# Experimental and computational approaches for deep metabolome annotation with application to the ecotoxicological model organism *Daphnia magna*

**DOI:** 10.1093/gigascience/giag055

**Published:** 2026-05-09

**Authors:** Thomas N Lawson, Martin R Jones, Andrew J Chetwynd, Elena Sostare, Stefan Weidt, Robert Mistrik, Warwick B Dunn, Ralf J M Weber, Mark R Viant

**Affiliations:** School of Biosciences, University of Birmingham, Edgbaston, Birmingham, B15 2TT, United Kingdom; Michabo Health Science Limited, Union House, 111 New Union Street, Coventry, CV1 2NT, United Kingdom; School of Biosciences, University of Birmingham, Edgbaston, Birmingham, B15 2TT, United Kingdom; School of Biosciences, University of Birmingham, Edgbaston, Birmingham, B15 2TT, United Kingdom; Phenome Centre Birmingham, University of Birmingham, Edgbaston, Birmingham, B15 2TT, United Kingdom; Michabo Health Science Limited, Union House, 111 New Union Street, Coventry, CV1 2NT, United Kingdom; Glasgow Polyomics, University of Glasgow, University Avenue, Glasgow, G12 8QQ, United Kingdom; HighChem, Mlynské nivy 5, 821 09 Bratislava, Slovakia; School of Biosciences, University of Birmingham, Edgbaston, Birmingham, B15 2TT, United Kingdom; Phenome Centre Birmingham, University of Birmingham, Edgbaston, Birmingham, B15 2TT, United Kingdom; School of Biosciences, University of Birmingham, Edgbaston, Birmingham, B15 2TT, United Kingdom; Phenome Centre Birmingham, University of Birmingham, Edgbaston, Birmingham, B15 2TT, United Kingdom; School of Biosciences, University of Birmingham, Edgbaston, Birmingham, B15 2TT, United Kingdom; Michabo Health Science Limited, Union House, 111 New Union Street, Coventry, CV1 2NT, United Kingdom; Phenome Centre Birmingham, University of Birmingham, Edgbaston, Birmingham, B15 2TT, United Kingdom

**Keywords:** metabolomics, mass spectrometry, deep metabolome annotation, galaxy platform, daphnia magna, model organism

## Abstract

**Background:**

Comprehensively characterizing the metabolomes of model organisms with high coverage and confidence is a critical step towards interpreting the metabolic basis of human and environmental health, yet there are formidable challenges involved in annotating metabolomes. A wide range of genotypes and phenotypes should be sampled with multiple complementary analytical approaches to cover the large and dynamic biochemical space they exhibit. In addition, multiple computational tools and approaches are required to annotate the metabolites from raw analytical data.

**Results:**

To address this, we developed the deep metabolome annotation (DMA) workflow. Applied to the ecological sentinel species, *Daphnia magna*, a pooled sample comprising 10 distinct strains exposed to both normal and stressed environmental conditions was extracted and systematically physicochemically separated via solid-phase extraction and liquid- and gas-chromatography prior to extensive multiple-stage mass spectrometric fragmentation, generating >8,000 raw data files, and supplemented by nuclear magnetic resonance spectroscopy. An extensive Galaxy-based computational approach was built to analyse these data, comprising >30 tools. The overall DMA efforts resulted in 8,181 annotated polar metabolites and lipids in *D. magna*, with the raw and processed data, tools, and annotations disseminated freely via public data repositories and a custom web-based interface to maximize reusability.

**Conclusions:**

The DMA workflow has generated one of the largest metabolome annotation datasets for any non-human model organism and provides the first in-depth characterization of the *D. magna* metabolome, serving as both a resource and a valuable catalyst for future DMA studies of other model organisms.

## Introduction

Large-scale efforts to map and catalogue both human and model organism genomes have been a fundamental driver of change in biological and biochemical research over the past few decades. The technological developments and resulting biological, biomedical, and environmental knowledge derived from such projects have helped underpin the modern era of biological sciences [1–4]. In contrast, our understanding of metabolic biochemistry (where we use the term “metabolites” here to represent the full spectrum of low molecular weight endogenous biochemicals from polar metabolites to lipids) has increased relatively minimally over the last half a century. Such knowledge must either be inferred from genome-scale metabolic reconstructions or, if measured experimentally, be limited to metabolites that can be annotated analytically (i.e., using metabolomics datasets). Ongoing improvements in both analytical and computational methods for metabolic annotation now allow for more extensive metabolite annotation coverage than what could be performed 10 years ago. However, although these developments are welcomed and are in part reflected by the increase in both studies featuring extensive metabolite annotation analysis [5–7] and the maturation of databases containing metabolites and relevant experimental data (Metabolights, Metabolomics Workbench, HMDB, GNPS, MoNA, MassBank, LipidBlast, and mzCloud [8–13]), for the majority of widely used model organisms, the metabolome knowledge is still severely lacking.

While the need for deeper metabolome knowledge of model organisms has been well established [14], the challenges are still considerable and multi-faceted. First, the biology: as the metabolome is driven by genes, the changing environment, and their interactions, achieving a comprehensive map of the breadth of a species’ metabolome requires a range of genotypes and phenotypes. Second, analytical chemistry: no single method is sufficient to cover the chemical space of a metabolome; hence, multiple physicochemical separations and detection techniques are required. Third, the computational challenges: as metabolomes are vast, specialized tools and workflows for data processing and metabolite annotation are required, together with resources for data and metadata management. Where possible, the data generated and software used should be Findable, Accessible, Interoperable, Reusable (FAIR), and scalable in order to support an anticipated further cascade of deep metabolome annotation (DMA) studies.

To address these challenges, we have developed and applied an experimental and computational workflow for extensively measuring the metabolome of model organisms, applied here to *Daphnia magna*. The International Metabolomics Society’s Model Organism Metabolomes task group [15] and the ongoing Precision Toxicology project [16] both highlight the crustacean *Daphnia* as a key model organism due to its importance as an indicator genus used to set ecotoxicological regulatory standards (SOR/2002–222), and from being extensively studied in the context of evolution and ecology [17–21], making it an excellent candidate for an in-depth investigation of its metabolome. Experimentally, the workflow involved culturing multiple strains of *D. magna* under normal and stressed conditions to provide a representative pooled sample for metabolome annotation. This sample underwent extensive extraction and physicochemical separation and (ultra)-high-performance liquid chromatography-high resolution tandem mass spectrometry ((U)HPLC-HRMS(/MS)) analysis. Concurrent fractionation yielded (semi-)purified metabolome fractions that underwent in-depth characterization by direct infusion-high resolution multiple stage mass spectrometry (DI-HRMS(/MS^*n*^)). Supplemental analyses by gas chromatography-electron ionization-high resolution mass spectrometry (GC-EI-HRMS) and 1- and 2-dimensional nuclear magnetic resonance spectroscopy (1D- & 2D-NMR), helped to ensure broad coverage of the physicochemical space of metabolites. A computational workflow was then developed and applied for processing, annotating, and managing the data and results, heavily utilizing the Galaxy Workflow platform [22]. The resulting metabolome annotations, data, metadata, and computational tools are disseminated through various channels (MetaboLights, GNPS, Galaxy, and a custom web portal named DMAdb) to ensure traceability and reusability.

**Figure 1:**
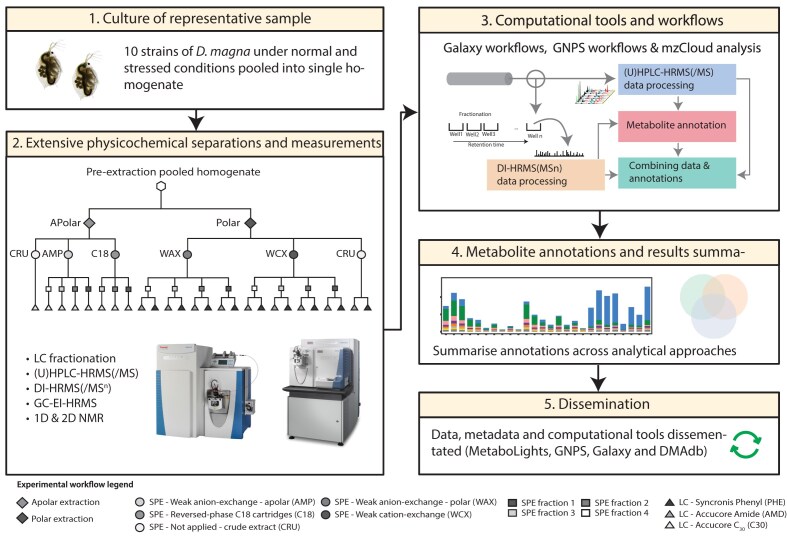
DMA workflow—conceptual overview and application to *D. magna*. (1) First a representative sample (applied to *D. magna* here) cultured under multiple conditions using distinct strains is homogenized into a single sample. (2) The pooled homogenized sample then undergoes extensive physicochemical separations (including polar and apolar extraction procedures, four types of solid-phase extraction (SPE)—each collecting 3–4 SPE fractions, three types of liquid chromatography (LC), and extensive LC fractionation). SPE fractions then undergo extensive analysis based on (U)HPLC-HRMS(/MS), followed by direct infusion-high resolution mass spectrometry with multiple-stage fragmentation (DI-HRMS(/MS^*n*^)). SPE fraction analyses are also supplemented with gas chromatography-electron ionization-high resolution mass spectrometry (GC-EI-HRMS) and 1- and 2-dimensional nuclear magnetic resonance spectroscopy (1D- & 2D-NMR). (3) Extensive computational tools and workflows were developed and applied to process and annotate the metabolite. The results are then summarized across the analytical workflow (4), and finally, the data, metadata and computational tools are disseminated to ensure traceability and reusability (5). Images of Thermo Scientific Q Exactive and Orbitrap Elite mass spectrometers by Thermo Fisher Scientific (Bremen), licensed under Creative Commons Attribution–ShareAlike 3.0 Unported (CC BY-SA 3.0), via Wikimedia Commons.

The combined extensive experimental and computational workflow, referred to here as the DMA workflow (see Fig. 1), has generated one of the largest metabolome annotation datasets for any single organism and provided the first in-depth characterization of the *D. magna* metabolome—a resource that is much needed to improve the interpretation of *Daphnia* biology and toxicology. The workflow presented here could also be adapted to support DMA studies in other model organisms.

## Methods

### DMA experimental workflow

#### Overview

**Figure 2:**
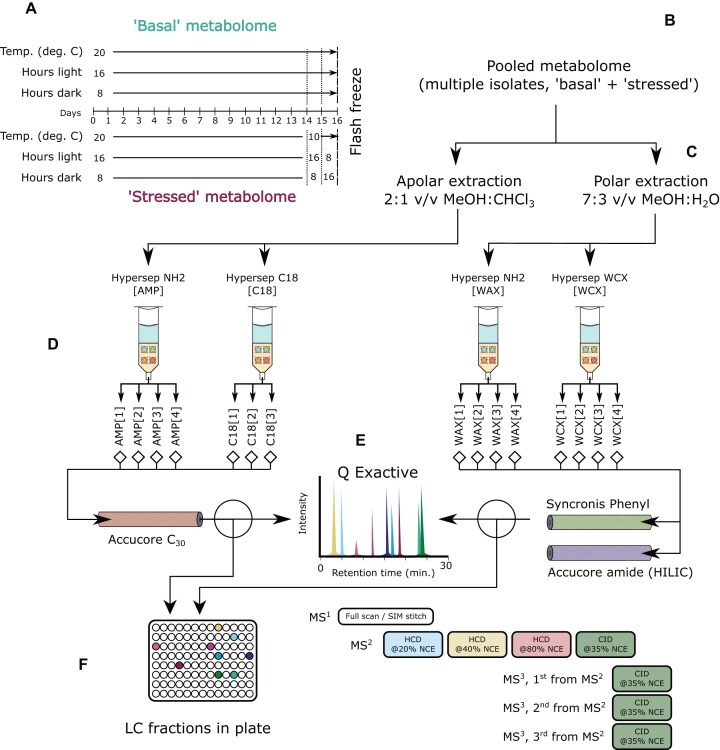
DMA experimental workflow for the physicochemical separation and measurement of *D. magna* metabolites. (A) Culturing: Culturing of 10 strains of a model organism under normal (basal) and stressed conditions was used to ensure a wide range of metabolites were present in (B) the pooled sample. For the DMA of *D. magna*, this involved flash-freezing in liquid nitrogen to quench metabolism, followed by homogenization and pooling into a single sample. (C) Liquid phase extraction: Two distinct liquid phase extractions were then performed on the pooled homogenate from step (B) for “polar” and “apolar” metabolites. (D) Solid-phase extraction (SPE): Four types of SPE were performed on the extracts from step (C), the polar extracts using weak anion-exchange (WAX) and weak cation-exchange SPE cartridges (WCX) and apolar extracts using weak anion-exchange (AMP) and reversed-phase C18 cartridges (C18). (E) (U)HPLC-HRMS(/MS): Three distinct (U)HPLC-HRMS(/MS) methods were applied using a Q-Exactive mass spectrometer: Accucore amide HILIC LC column (AMD) and Syncronis phenyl LC column (PHE) for analysis of metabolites from the polar arm of the workflow, and Accucore C30 LC column (C30) for the apolar extract and any SPE fractions derived from the apolar extract. (F) Fractionation and DI-HRMS(/MS^*n*^): The eluate from the LC columns in step (E) was fractionated into plates for subsequent extensive multiple-stage fragmentation (MS^*n*^) analysis applied (up to MS^3^), including at multiple collision energies and with technical replication.

An experimental workflow for extensive physicochemical separation and analytical measurement of metabolites has been developed for the analysis of model organisms—applied here to *D. magna*. An overview is provided in Fig. 2.

This extensive workflow, applied to both the *Daphnia* sample and a metabolite reference standard sample, was separated into 135 distinct experimental assays (see Supplementary Table S1). Of these assays, 103 corresponded to (U)HPLC-HRMS(/MS) and DI-HRMS(/MS^*n*^) analysis, generating 8,846 raw mass spectrometry files (5,430 files specifically corresponding to the *Daphnia* sample, with the remaining files corresponding to metabolite reference standards, extract blanks, or quality assurance measurements such as (U)HPLC-HRMS system equilibration). See Supplementary Table S2 for the full file list.

#### *Daphnia magna* culturing and sample preparation

The DMA experimental workflow is applied here to *D. magna*; however, the same considerations extend to DMA analyses of other model organisms. The workflow should take as input a set of samples that, ideally, span diverse genetic and environmental backgrounds and collectively reflect the full metabolic repertoire accessible to the organism under study. These samples are then pooled and homogenized to form a single complex sample matrix that constitutes an average of the constituent metabolomes.

For the DMA of *D. magna*, ca. 2,000 individual organisms were pooled and homogenized, generating a homogenate consisting of 10 distinct strains (Supplementary Table S3) exposed to two contrasting environmental conditions. A “basal” metabolome was represented by *D. magna* cultured under standard conditions (20 ± 2°C with a 16:8 hr light:dark ratio) for 14 days, followed by a further 48 hr under the same conditions. A “stressed” metabolome was represented by *D. magna* cultured for 14 days under standard conditions, followed by 24 hr at 10 ± 1°C with a 16:8 hr light:dark ratio, and then a further 24 hr at 10 ± 1°C with an 8:16 hr light:dark ratio. Under both conditions, *Daphnia* were maintained without food (algae) throughout the final 48 hr of culturing to minimize the presence of algae in the gut and prioritize measurement of metabolites derived from *Daphnia* rather than the food source. Further details are provided in Supplementary Section 1.5 and Tables S4 and S5.

#### Metabolite extraction from *D. magna* pooled sample

Metabolites were extracted from the pooled homogenate using two distinct liquid-phase extraction protocols: a “polar” extraction in which (predominantly polar through to moderately polar) metabolites were extracted using a solution comprising 71.4:28.6% v/v methanol:water, and an “apolar” extraction protocol in which metabolites (spanning moderately apolar through to highly apolar metabolites, including lipids) were extracted using a solution of 1:1 v/v methanol:chloroform, to which water was added to form a biphasic system comprising 2:2:1.8 v/v/v chloroform:methanol:water, from which the lower (apolar) layer was recovered. Polar extracts were dried in a centrifugal vacuum concentrator (Speedvac), while apolar extracts were dried under a stream of nitrogen gas. Further details are provided in Supplementary Section 1.6.

#### Solid-phase extraction-based fractionation of metabolite extracts

Constituents of the polar or apolar extract were independently fractionated over two solid-phase extraction (SPE) cartridges. The polar extract was fractionated using weak anion-exchange (WAX; aminopropyl) and weak cation-exchange cartridges (WCX; carboxylic acid), while the apolar extract was fractionated using weak anion-exchange (referred to as AMP to differentiate from the polar arm; aminopropyl) and reversed-phase C18 cartridges (C18). See Supplementary Section 1.7 and Figs S1 and S2 for further details. The resulting 15 SPE fractions and remaining unfractionated extracts (referred to as “crude” extract) were analysed by (U)HPLC-HRMS(/MS). A selected subset of SPE fractions, alongside crude extract, was also analysed by 1D- & 2D-NMR spectroscopy and GC-EI-HRMS to further expand the breadth of metabolome annotation.

#### (U)HPLC-HRMS(/MS), DI-HRMS(/MS^n^) and LC fractionation

Three distinct (U)HPLC-HRMS(/MS) methods were applied in both positive and negative ionization modes to analyse constituents of the polar and apolar crude extracts, and associated SPE fractions. Polar crude extract and polar SPE fractions were analysed by hydrophilic interaction liquid chromatography (HILIC) using an Accucore amide column (AMD; 2.1 × 100 mm, 2.6 µm solid core; Thermo Scientific), and reversed-phase liquid chromatography (RPLC) based on a Syncronis phenyl column (PHE; 2.1 × 100 mm, 1.7 µm; Thermo Scientific). Constituents of the apolar crude extract and apolar SPE fractions, meanwhile, were analysed by RPLC using an Accucore C30 column (C30) (2.1 × 100 mm, 2.6 µm solid-core particle, 150 Å; Thermo Scientific). All chromatographic separations were performed using a Dionex Ultimate 3000 liquid chromatography system. A Q Exactive mass spectrometer (Thermo Scientific), fitted with a heated electrospray ionization source, was used for HRMS(/MS) mass spectrometry analysis of metabolites eluted from LC columns. A passive flow-splitting tee-piece was installed between the LC column outlet and Q Exactive inlet to facilitate simultaneous collection of 20-second-wide LC fractions and associated HRMS(/MS) data. Each fraction was collected into independent wells of a deep well plate. LC fraction collection plates were dried in a centrifugal evaporator at the end of an analysis sequence.

Initial (U)HPLC-HRMS analyses were used to create inclusion and exclusion lists (i.e., *m/z* features of interest) to direct the subsequent data-dependent acquisition (DDA) of (U)HPLC-HRMS/MS data. In parallel, eluent from the LC columns was fraction-collected during mass spectral acquisition, which was then subject to extensive DI-HRMS(/MS^*n*^).

DI-HRMS(/MS^*n*^) analyses of resuspended LC fractions were performed using an Orbitrap Elite mass spectrometer (Thermo Scientific) using both higher energy collisional dissociation (HCD) and collision-induced dissociation (CID) at several levels of normalized collision energy (NCE). Specifically, HCD was performed at 20, 40, and 80% NCE, followed by CID at 35% NCE with multi-stage fragmentation up to MS^3^. In total, 2,305 LC fractions were analysed as part of the DMA of *D. magna*. The acquisition of DI-HRMS(/MS^*n*^) fragmentation data was directed via a predefined list of targeted *m/z* features, derived from prior DI-HRMS analysis of the same fraction.

For detailed information on the (U)HPLC-HRMS analytical setup and LC methods, including the fractionation procedure, as well as the data acquisition sequence and computational methods used to create inclusion/exclusion lists for targeting the most informative features for fragmentation data acquisition, see Supplementary Section 1.8.1 and Fig. S3. Additionally, for more details on the resuspension of LC fractions, the DI-HRMS(/MS^*n*^) analytical setup, data acquisition sequence, and the computational methods for DI-HRMS(/MS^*n*^) used to develop both inclusion/exclusion lists and instrument methods files that directed DI-HRMS^*n*^ data acquisition, see Supplementary Section 1.8.2 and Fig. S4.

#### (U)HPLC-HRMS(/MS) method optimization

The PHE and AMD (U)HPLC-HRMS(/MS) methods underwent optimization for DMA of *D. magna* aiming to maximize reproducibly detectable metabolic features while enabling reproducible fractionation for downstream analyses. The methodology for the optimization is detailed within the Supplementary Section 1.9, Fig. S5, and Tables S6–S8. The C30 (U)HPLC-HRMS(/MS) method was previously optimized for broad lipid profiling applications by Thermo Fisher Scientific; hence, no further optimization was pursued.

#### GC-EI-HRMS

GC-EI-HRMS was performed on the WAX and WCX fractions, as well as the crude polar extract, using a TriPlus RSH autosampler and TRACE 1310 gas chromatograph coupled to a Q Exactive mass spectrometer (Thermo Scientific), and an Extractabrite electron ionization/chemical ionization source. Further details are provided in Supplementary Section 1.10.

#### 1D- & 2D-NMR spectroscopy

The WAX and WCX SPE fractions were also analysed using a combination of 1D- & 2D-NMR spectroscopy experiments performed using a Bruker AVANCE III 600 MHz NMR spectrometer, equipped with a 1.7 mm TCI-Cryoprobe and operated at a proton frequency of 600.13 MHz. Each sample was measured using 1D proton nuclear overhauser effect NMR spectroscopy (1D-^1^H-NOESY) followed by 2D homonuclear ^1^H-^1^H (2D-JRes and TOCSY) and heteronuclear ^1^H-^13^C (HSQC) experiments to support annotation. Further details are provided in Supplementary Section 1.11.

### Computational tools and workflows for data processing, metabolite annotation, and data analysis

#### Overview

An extensive computational workflow utilizing the Galaxy platform has been developed to analyse the highly complex data acquired through the experimental DMA workflow. This computational workflow predominantly consists of an extensive Galaxy-based workflow, with additional annotations incorporated from external sources, i.e., mzCloud, GNPS workflows, GC-EI-HRMS annotations and 1D- & 2D-NMR annotations. A summary is provided in Supplementary Section S12 and Fig. S6.

The Galaxy workflow component generated 104 Galaxy histories (see Supplementary Table S1 for links to corresponding Galaxy history), 60 of which were used for the analysis of the *Daphnia* samples. Each Galaxy history contains a combined SQLite database containing all annotations and relevant (average) spectra across all assays.

#### Galaxy workflow details

**Figure 3:**
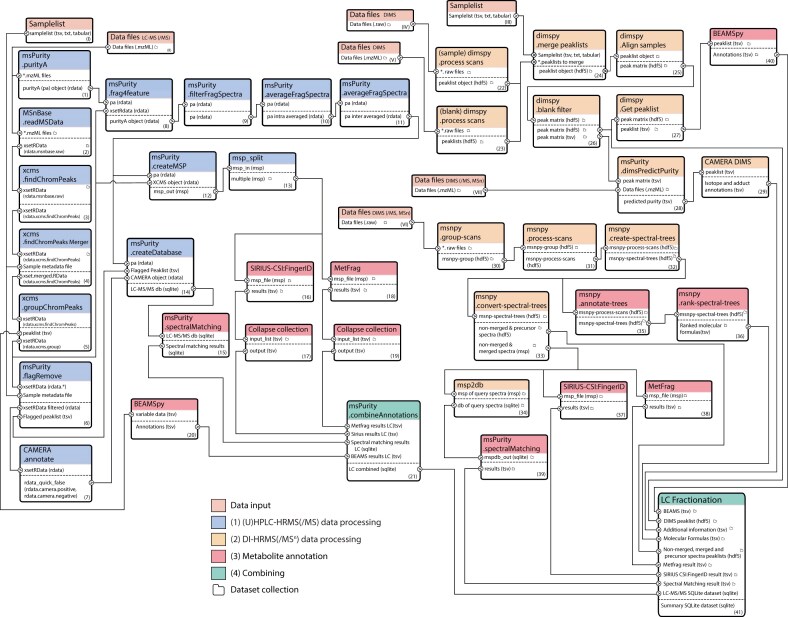
DMA data processing and metabolite annotation Galaxy workflow. Components are separated by colour into “Data input”, “(U)HPLC-HRMS(/MS) data processing”, “DI-HRMS(/MS^*n*^) data processing”, “metabolite annotation”, and “Combining”. See Supplementary Table S9 for description of each tool.

The Galaxy workflow (see Fig. 3) was designed specifically to process and perform metabolite annotation across the multiple data types produced by the DMA experimental workflow, including (U)HPLC-HRMS(/MS) and DI-HRMS(/MS*^n^*). Utilizing the high level of replication achieved from the workflow, averaging and filtering were performed on both the (U)HPLC-HRMS(/MS) and DI-HRMS(/MS*^n^*) data so that higher-quality, reproducible fragment peaks were retained for multiple complementary computational approaches to metabolite annotation.

The workflow is split into five components: “Data input”, “(U)HPLC-HRMS(/MS) data processing”, “DI-HRMS(/MS*^n^*) data processing”, “Metabolite annotation”, and “Combining”. A detailed description of all steps used in the workflow can be found in Supplementary Section 1.13— with individual schematics detailing the (U)HPLC-HRMS(/MS) (see Supplementary Fig. S7) and DI-HRMS(/MS^*n*^) fragmentation data processing (see Supplementary Fig. S8). See also Supplementary Table S9 for the description of all tools used and Table 1 for the location of code repositories for each Galaxy tool and underlying software.

**Table 1:** Galaxy tools—code availability.

Project name (biotools id)	Galaxy tools	Galaxy tool code home page	Underlying software code home page	Licence	Language
**Pre-existing software and Galaxy tools**					
MSnBase (biotools:msnbase)	MSnBase.readMSData	https://github.com/workflow4metabolomics/tools-metabolomics	https://www.bioconductor.org/packages/release/bioc/html/MSnbase.html	Underlying software: Artistic-2.0 Galaxy tool: GPL-3.0	R
XCMS (biotools:xcms)	xcms.findChromPeaks xcms.findChromPeaks Merger xcms.groupChromPeaks	https://github.com/workflow4metabolomics/tools-metabolomics	http://bioconductor.org/packages/release/bioc/html/xcms.html[60]	GPL (>= 2)	R
CAMERA (biotools:camera)	CAMERA.Annotate	https://github.com/workflow4metabolomics/tools-metabolomics	https://www.bioconductor.org/packages/release/bioc/html/CAMERA.html[61]	GPL (>= 2)	R
BEAMSpy (Not available)	BEAMSpy	https://github.com/computational-metabolomics/beamspy-galaxy	https://github.com/computational-metabolomics/beamspy https://more.bham.ac.uk/beams/	GPL-3.0	R
DIMSpy (biotools:dimspy)	dimspy.Process scans dimspy.merge peaklists dimspy.align samples dimspy.blank filter dimspy.Get peaklist	https://github.com/computational-metabolomics/dimspy-galaxy	https://github.com/computational-metabolomics/dimspy	GPL-3.0	Python
**Software and/or the Galaxy tool was developed by authors**					
^b^msPurity (biotools:mspurity)	msPurity.purityA msPurity.flagRemove msPurity.frag4feature msPurity.filterFragSpectra msPurity.averageFragSpectra msPurity.createMSP msPurity.createDatabase msPurity.spectralMatching msPurity.combineAnnotations msPurity.dimsPredictPurity	https://github.com/computational-metabolomics/mspurity-galaxy/	https://www.bioconductor.org/packages/release/bioc/html/msPurity.html[26]	GPL-3.0	R
^b^MSnPy (biotools:msnpy)	MSnPy.group-scans MSnPy.process-scans MSnPy.create-spectral-trees MSnPy.annotate-trees MSnPy.convert-spectral-trees	https://github.com/computational-metabolomics/msnpy-galaxy	https://github.com/computational-metabolomics/msnpy	GPL-3.0	Python
^b^msp2db (Not available)	msp2db	https://github.com/computational-metabolomics/dmatools-galaxy	https://github.com/computational-metabolomics/msp2db	GPL-3.0	Python
^b^CAMERA DIMS (Not available)	CAMERA DIMS	https://github.com/computational-metabolomics/dmatools-galaxy	https://github.com/computational-metabolomics/cameraDIMS	GPL (>= 2)	R
^a^SIRIUS CSI:FingerID (biotools:Sirius)	SIRIUS CSI:FingerID	https://github.com/computational-metabolomics/sirius-csifingerid-galaxy/	https://bio.informatik.uni-jena.de/software/sirius/[30]	Underlying software: GNU AGPL Galaxy tool: GPL-3.0	Java (and python for Galaxy wrapper)
^a^MetFrag (biotools:metfrag)	MetFrag	https://github.com/computational-metabolomics/metfrag-galaxy/	https://ipb-halle.github.io/MetFrag/[27–29]	Underlying software: GPL (>= 2) Galaxy tool: GPL-3.0	Java (and python for Galaxy wrapper)
^b^LC fractionation processor (Not available)	LC fractionation processor	https://github.com/computational-metabolomics/lcfrac-galaxy	https://github.com/computational-metabolomics/lcfrac-galaxy(all functionality within Galaxy tool)	GPL-3.0	Python
^b,c^ deconran (Not available)	deconrank	https://github.com/computational-metabolomics/dmatools-galaxy	https://github.com/computational-metabolomics/deconrank	GPL-3.0	Python

All tools and software described in table are operating system platform independent.

a Galaxy tool developed by authors.

b New (or updated) underlying software and Galaxy tool developed by authors.

c Not used directly in the annotation workflows but was used in the “directed acquisition workflows” described in Supplementary Section 1.8. The “msp2db”, “CAMERA DIMS”, “LC fractionation processor”, and “deconrank” are considered highly specific to this workflow and currently of limited general applicability, and therefore have not yet been registered in bio.tools.

The Galaxy workflow incorporates both new and existing tools, e.g., existing Workflow4Metabolomics XCMS Galaxy tools [23] for (U)HPLC-HRMS peak picking and processing; DIMSpy Galaxy tools [24, 25] for DI-HRMS data processing. New Galaxy tools developed for the DMA project include updated functionality from the msPurity R package [26] to filter and flag spectra, average fragmentation spectra, create MSP and SQLite files of (U)HPLC-HRMS(/MS) data, perform spectral matching, and combine metabolite annotations from multiple sources; the MSnPy Python package and Galaxy tools to process DI-HRMS(/MS*^n^*) data with both multiple-stage and multiple-energy fragmentation spectral trees, perform spectral averaging across trees, and annotate and rank spectral trees with molecular formulae; and the LC Fractionation Galaxy tool, which was created to combine all spectra and metabolite annotations from a DMA LC fractionation experiment. In addition, Galaxy wrappers have been created for the *in silico* fragmentation software MetFrag [27–29] and mass spectrometry data processing and metabolite annotation software SIRIUS CSI:FingerID [30].

This modular, multistage workflow ensures reusability across other studies. Additionally, a Galaxy instance [31] was created for conducting the Galaxy DMA analysis, with public access to the histories, data, and parameters.

#### mzCloud library search

All annotations derived from mzCloud were performed programmatically in batches using mzCloud proprietary software [32]. Each fragmentation scan was treated individually, and spectral matching was performed against the mzCloud database. The results were saved as an SQLite database with reference to both the query and library spectra of each annotation. The data were filtered to only include “endogenous” metabolites and spectral matches with a spectral similarity score >0.7.

#### GNPS library search

Fragmentation spectra were searched against the GNPS library spectra using the online workflow on the GNPS website [11, 33]. The precursor ion mass tolerance was set to 0.02 Da and a MS/MS fragment ion tolerance of 0.02 Da. Additionally, spectral matches were filtered to have an error of 10 ppm or less between the library precursor *m/z* and the query precursor *m/z*. Annotations were further restricted to spectral matches in which the library and query spectra were acquired on mass spectrometers operating in the same ionization mode.

#### GNPS molecular network analysis

A molecular network was generated using the online workflow on the GNPS website [11, 33]. The data were filtered to remove all fragment ions within ±17 Da of the precursor *m/z*. Fragmentation spectra were window-filtered by retaining only the top six fragment ions in the ±50 Da window across the spectrum. Both the precursor ion mass tolerance and fragment ion tolerance were set to 0.02 Da. A network was then constructed where edges were filtered based on having a cosine score >0.7 and more than two matched peaks. Further, edges between two nodes were only kept in the network if each of the nodes appeared in each other’s respective top 10 most similar nodes. Finally, the maximum size of a molecular family was set to 100, and the lowest scoring edges were removed from molecular families until the molecular family size was below this threshold. The fragmentation spectra in the network were then searched against GNPS’s spectral libraries. The library spectra were filtered in the same manner as the input data. All matches kept between network and library spectra were required to have a score >0.7 and at least two matched peaks. Further annotation was performed using the Dereplicator tool [34] and the MS2LDA [35] workflow to determine common mass motifs.

#### Combining and summarising all annotations

Five main sources of annotations were combined into a final list of metabolite annotations, including Galaxy workflow annotations, GNPS workflow annotations, mzCloud annotations, NMR annotations, and GC-EI-HRMS annotations. All data were combined into a single table encompassing annotations from all assays, and a final stage of filtering was performed as described in Supplementary Section 1.14.

All annotations were chemically classified using ClassyFire [36]; all reported terms at the “superclass”, “class”, and “subclass” levels are used as defined in the ClassyFire taxonomy.

#### Comparison to other metabolite databases

The final list of *D. magna* metabolites was compared with compound lists from KEGG [37–39], ChEBI [40], HMDB [8], and MTox700+ [41]. ChEBI was filtered to include only compounds with a known species origin. PhyloT was used to generate the species phylogenetic tree using the NCBI taxonomy [42]. Matching was based on compounds sharing the same partial InChIKey (i.e., the first block of the InChIKey that encodes the molecular skeleton).

QIAGEN Ingenuity Pathway Analysis (IPA, QIAGEN Inc.) was used to derive metabolite-pathway associations. A metabolite list containing PubChem, HMDB, and KEGG identifiers was imported into the IPA software. “Metabolomics core analysis” was then conducted using all mapped metabolites.

### Assessment of the computational and experimental DMA workflow with metabolite reference standards

Metabolite reference standards (see Supplementary Table S11) were analysed to evaluate the effectiveness of the overall DMA workflow, specifically the (U)HPLC-HRMS(/MS) component of the experimental workflow, including extraction, chromatographic separation, and mass spectrometric analysis. The same computational workflow was applied as used for the *D. magna* samples. The measured metabolite reference standards were then compared to the expected annotations for each assay by matching their partial InChIKeys.

### DMA database and web portal

**Figure 4:**
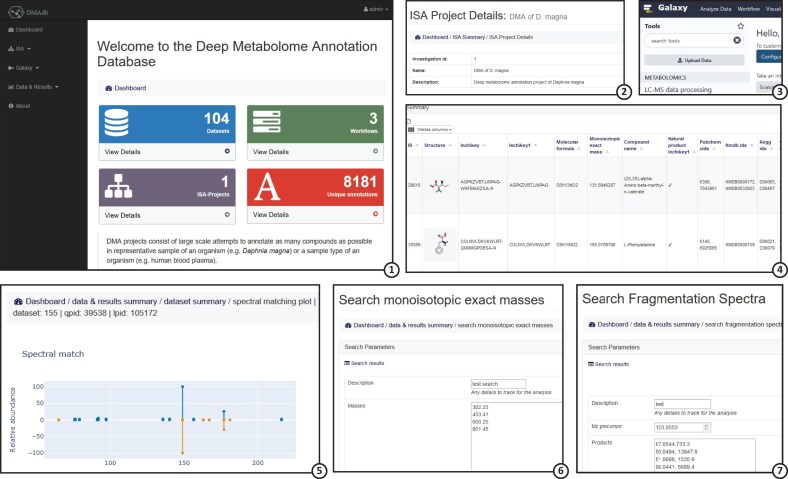
Overview of the DMA database and web portal (DMAdb) developed and implemented to organize, manage, and disseminate DMA data and associated metabolite annotations of *D. magna*. (1) Dashboard view of the DMAdb interface. (2) Investigation/Study/Assay (ISA) representation of the DMA data, capturing relevant ontologies, protocols, and experimental processes. (3) Data processing and annotation workflows performed via the Galaxy platform, with outputs uploaded to and stored within DMAdb. (4–5) Browsing and visualization of metabolite annotations and associated raw data files. (6–7) Search functionalities supporting exact mass queries and MS/MS-based spectral matching.

The DMA database and web portal (DMAdb [43]; Fig. 4) were developed to organize, manage, and disseminate DMA datasets and associated metabolite annotations. It was implemented using Django, a high-level Python web framework, and comprises multiple applications (i.e., django-gfiles, django-galaxy, and django-mogi [44]; see Availability of Supporting Source Code and Requirements for further details). Experimental datasets were structured in alignment to the Investigation/Study/Assay (ISA) framework [45]  to support standardized organization of experimental data and metadata. The DMAdb structure enabled consistent metadata capture and traceability between raw data, processing workflows, and metabolite annotations. DMAdb was primarily designed as an internal, administrator-restricted data management platform with Galaxy workflow integration. However, controlled public access to selected functionalities is provided to enable basic exploration and searching of DMA datasets, as described in the Results section.

## Results and discussion

### (U)HPLC-HRMS(/MS) method optimization

The PHE and AMD (U)HPLC-HRMS(/MS) methods were optimized to improve detection of reliable metabolic features and support reproducible fractionation. Full details and supporting figures are provided in Supplementary Section S2.1, Table S12, and Figs S9–S26.

### Summary of all *D. magna* metabolite annotations and compound classifications

In total, 8,181 unique metabolite annotations are reported from all experimental assays (including (U)HPLC-HRMS(/MS), DI-HRMS(/MS*^n^*), 1D- & 2D-NMR and GC-EI-HRMS), summarized in Supplementary Table S13. The combined annotations and compound classifications across all technologies and approaches are presented here, with further details, including specifics for each measurement technology, provided in Supplementary Sections 2.2–2.5 and Supplementary Tables S14–15 . In summary, the majority of annotations were reported for the (U)HPLC-HRMS(/MS) and DI-HRMS(/MS*^n^*) datasets, observing 8,132 unique annotations compared with four unique metabolites for GC-EI-HRMS and three for 1D- & 2D-NMR; see Venn diagram in Supplementary Fig. S27. Although the discrepancy in observed annotations across these analytical technologies appears large, with annotations derived from (U)HPLC-HRMS(/MS) and DI-HRMS(/MS^*n*^) dominating the counts, this is consistent with the analytical prioritization of these technologies and the comparatively more extensive computational interrogation applied to them.

**Figure 5:**
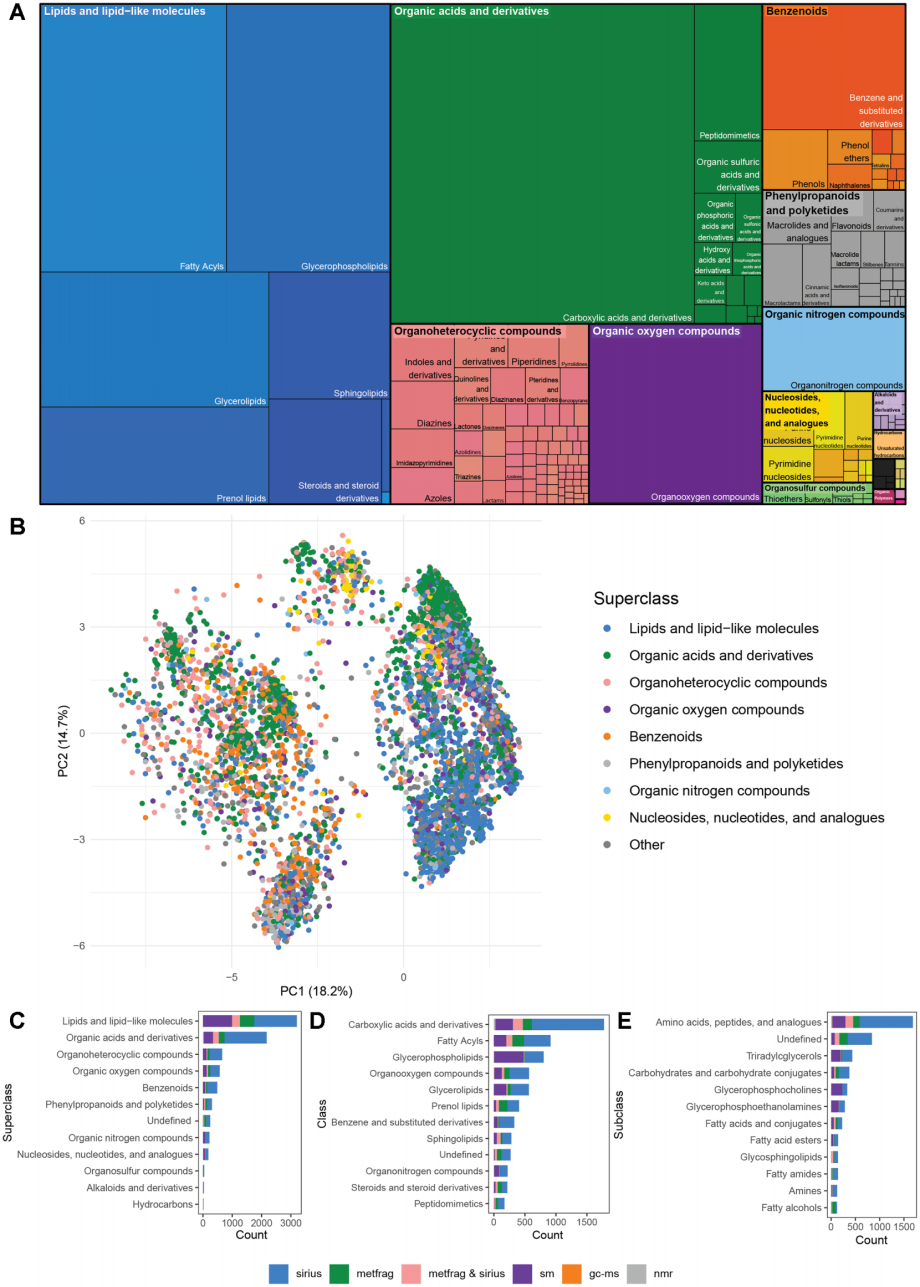
Compound classifications for the 8,181 *D. magna* annotated metabolites. (A) Treemap of metabolite “superclasses” and “classes” for the metabolite annotations. (B) Principal component analysis of PubChem fingerprints for all unique metabolite annotations: colour represents the superclass classification of the metabolite annotation. (C) Count of unique metabolite annotations for “superclass” compound classifications. Showing the top 12 “superclasses”, colour represents the annotation approach used (annotation was either derived using SIRIUS CSI:FingerID, MetFrag, SIRIUS CSI:FingerID & MetFrag, spectral matching, GC-EI-HRMS or 1D- & 2D-NMR). (D) Count of unique metabolite annotations for “class” compound classifications. Showing the top 12 “classes”, colour represents the annotation approach used (annotation was either derived using SIRIUS CSI:FingerID, MetFrag, SIRIUS CSI:FingerID & MetFrag, spectral matching, GC-EI-HRMS or 1D- & 2D-NMR). (E) Count of unique metabolite annotations for “subclass” compound classifications. Showing the top 12 “subclasses”, colour represents the annotation approach used (annotation was either derived from SIRIUS CSI:FingerID, MetFrag, SIRIUS CSI:FingerID & MetFrag, spectral matching, GC-EI-HRMS or 1D- & 2D-NMR).

Compound classification via ClassyFire was possible for 7,934 (97%) metabolites to at least the “Superclass” level. The remaining unclassified metabolites had either incompatible SMILES for ClassyFire or lacked a SMILES annotation from PubChem. See Fig. 5A for a treemap summarising all annotation superclasses and classes, demonstrating the diverse biochemical space observed. The chemical space was further explored using principal component analysis (PCA) of the PubChem molecular fingerprints, showing broad clustering of metabolites based on their structure, with no obvious outliers (see Fig. 5B).

The most common “superclass” observed was lipids and lipid-like molecules (3,202 uniquely annotated metabolites), followed by organic acids and derivatives (2,173); organoheterocyclic compounds (656); organic oxygen compounds (572); benzenoids (489); phenylpropanoids and polyketides (306); organic nitrogen compounds (221); nucleosides, nucleotides, and analogues (185); organosulfur compounds (44); alkaloids and derivatives (32); hydrocarbons (17); organophosphorus compounds (17); lignans, neolignans, and related compounds (11); and three other “superclasses” with six or fewer annotated metabolites. A total of 247 metabolites could not be classified to a “superclass” level.

The most common “class” classification observed was for carboxylic acids and derivatives (1,776) followed by fatty acyls (913); glycerophospholipids (803); organooxygen compounds (567); glycerolipids (564); prenol lipids (406); benzene and substituted derivatives (329); sphingolipids (282); organonitrogen compounds (221); steroids and steroid derivatives (217); peptidomimetics (170); phenols (72); macrolides and analogues (68); indoles and derivatives (67); organic sulphuric acids and derivatives (65); diazines (58); purine nucleosides (52); and 179 other “classes” with 46 or fewer annotated metabolites. A total of 269 metabolites could not be classified to a “class” level.

The most common “subclass” classification was for amino acids, peptides, and analogues (1,681) followed by triradylcglycerols (434); carbohydrates and carbohydrate conjugates (374); glycerophosphocholines (330); glycerophosphoethanolamines (282); fatty acids and conjugates (226); fatty acid esters (142); glycosphingolipids (141); fatty amides (140); amines (122); fatty alcohols (120); fatty acyl glycosides (97); ceramides (96); depsipeptides (92); lineolic acids and derivatives (87) and 338 other subclasses with 82 or fewer annotated metabolites. A total of 838 compounds could not be classified to a “subclass” level. The top 12 most common superclasses, classes and subclasses that have been annotated are shown in Fig. 5C–E.

We note that some of the metabolites annotated here are likely to originate from organisms other than *D. magna*. These may include bacteria (exogenous and microbiota), fungi or parasites that were potentially present in the non-axenic cultures used (despite daily visual inspections to identify and remove cultures with potential contamination), as well as residual algal feed material in the digestive tracts of the *D. magna*. Indeed, some metabolites were assigned compound superclasses typically associated with the plant kingdom, e.g., 306 phenylpropanoids and polyketides and 32 alkaloids and derivatives, though biosynthesis within the animal kingdom is known [46] and some of these annotations could potentially be “false positive” assignments. Metabolic flux analyses, or radio-labelled isotope tracer experiments, would likely be required to definitively determine the exact origins of each metabolite reported. Alternatively, sterile culturing conditions coupled with the use of dextran beads to purge algal feed material from the digestive tracts of *Daphnia* may prove helpful in focussing future DMA-like experiments on metabolites that are exclusively present in and/or produced by *D. magna*.

(U)HPLC-HRMS(/MS) and DI-HRMS(/MS*^n^*) annotations were predominantly derived from spectral matching, MetFrag and SIRIUS CSI:FingerID annotation approaches. SIRIUS CSI:FingerID produced the most unique annotations, with MetFrag and spectral matching having similar counts of unique annotations (see Supplementary Section 2.3 and Fig. S28 for further details). This finding should not be interpreted as identifying the most effective metabolite annotation method, as the number of annotations observed can change dramatically based on filtering criteria and the compound and spectral libraries used. Rather, this simply illustrates the origins of the annotations in this *D. magna* DMA project. The differing results between the annotation approaches do, however, caution against relying on only a single method, both in terms of the breadth of coverage of the tool used and the potential reliability of annotations.

By using a range of metabolite annotation approaches, the confidence in the resulting annotations can be adjusted according to user preference. For example, if only annotations reported by either MetFrag or spectral matching are considered (i.e., disregarding annotations derived only from SIRIUS CSI:FingerID that may yield more false positives amongst the large number of annotations reported), it would result in 3,591 annotations (or 3,601 if the 1D- & 2D-NMR and GC-EI-HRMS annotations are included). Alternatively, annotations can be filtered even more strictly by specifying that they should be observed with at least two of the three fragmentation data analysis approaches, resulting in 1,286 annotations (or 1,301 if all 1D- & 2D-NMR and GC-EI-HRMS annotations are also included). These subsets of annotations could be considered more reliable, though it is important to highlight that the full set of 8,181 annotations observed from all fragmentation-based annotation approaches was derived from sufficiently unique fragmentation spectra to yield this high number of unique metabolite annotations. While some annotations may be less reliable (i.e., from SIRIUS CSI:FingerID only), the number of unique fragmentation spectra demonstrates the richness of the *D. magna* metabolome.

Additionally, *de novo* molecular formula annotation using MSnPy was performed on the DI-HRMS(/MS^*n*^) data via the MSnPy spectral annotation functionality within the Galaxy workflow. The DI-HRMS(/MS^*n*^) data are particularly suited to this approach due to the high level of measurement replication at different collision energies and fragmentation levels. A total of 40,240 unique molecular formulae were annotated to an MSnPy rank of 1 (32,672 observed from positive ionization mode and 9,768 from negative mode). Ranking was based on the application of common “consistency” rules for filtering formulae and neutral losses [47], which use fragmentation tree consistency to evaluate and rank the most plausible molecular formulae. Only molecular formulae were included where there were 10 or fewer possible top-ranked candidates. It should be noted that multiple molecular formulae can share a rank of one and that, despite stringent filtering, false positives will be present. It is also worth noting that this stringent filtering will have excluded many annotations for higher-mass precursor ions, which tended to produce excessively large sets of candidate molecular formulae, thereby preventing a high number of false-positive annotations. This approach does not rely on prior knowledge of spectral libraries or compound databases and therefore provides a potentially less biased insight into the biochemistry of the metabolome, albeit limited to the level of molecular formula (i.e., non-structural) annotation.

### Metabolites and compound classes physicochemically separated by DMA experimental workflow

**Figure 6:**
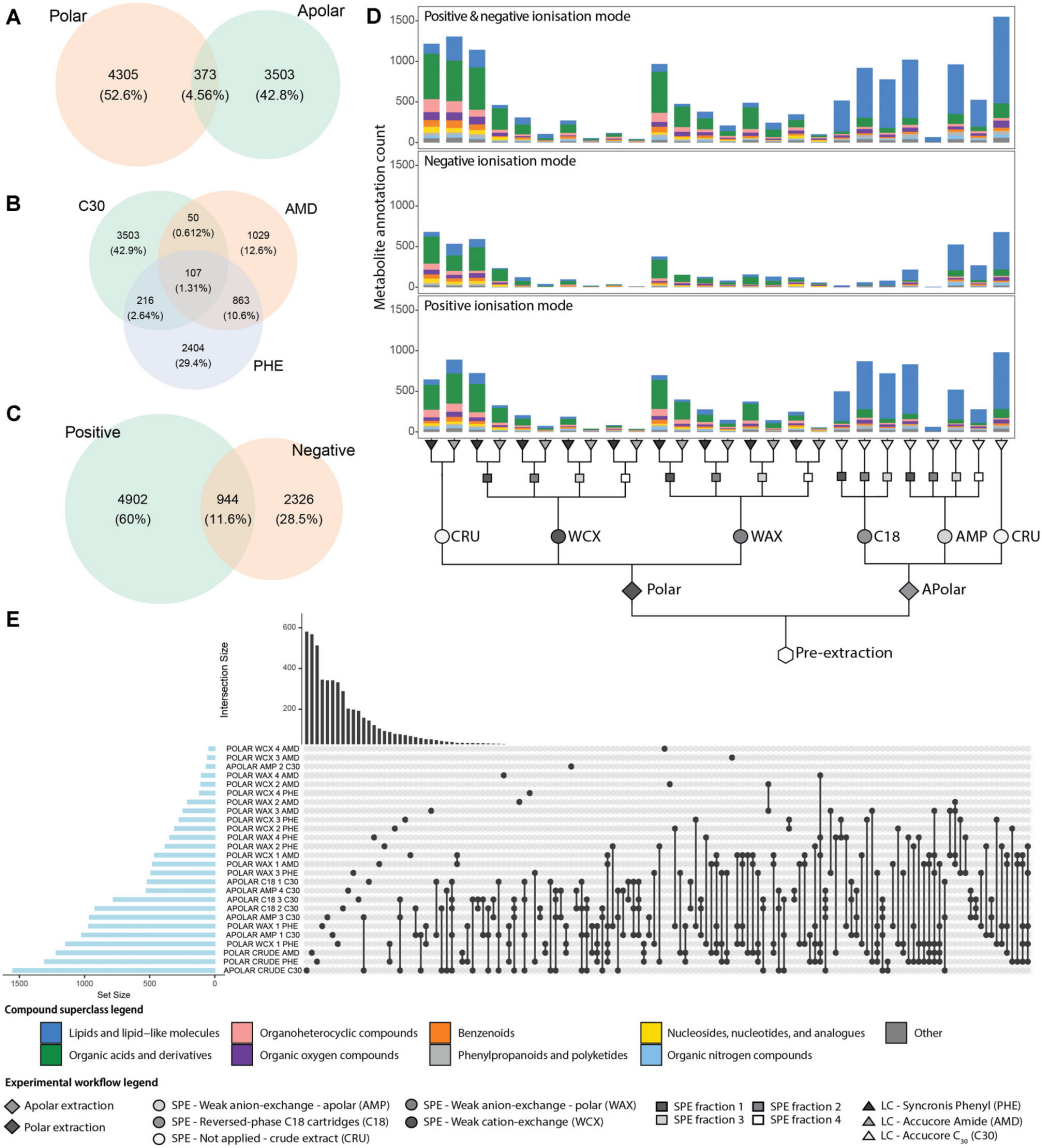
Contributions of extraction, SPE fractionation, LC separation, and mass spectrometric ionization methods to the number of metabolite annotations for *D. magna*. (A) Venn diagram of metabolite annotations observed across extraction approaches. (B) Venn diagram of metabolite annotations observed across all chromatography techniques. (C) Venn diagram of metabolite annotations observed across positive and negative ionization modes. (D) Count of metabolite annotations across experimental workflow components: bar charts shown for positive ionization mode, negative ionization mode and combined positive and negative ionization modes. Colour represents the superclass compound classification of the annotations. See thebottom of figure for colour code used for the compound superclass. (E) UpSet plot summarising the overlap of metabolite annotations between assays (positive and negative assays have been combined).

The extent of physicochemical separation achieved by the DMA experimental workflow was evaluated to determine the effectiveness, or potential redundancy, of components within the workflow (see Fig. 6).

When combining all annotations from either the polar or apolar arm of the workflow (see Fig. 6A), both arms generated a substantial number of unique metabolite annotations (with 4,305 and 3,503 metabolites unique to the polar and apolar arms respectively, and only 373 metabolites shared). This finding highlights the importance of the extraction procedure within the workflow and the necessity of both arms of the workflow to provide a comprehensive view of the metabolome. When combining all annotations from each chromatography approach used (see Fig. 6B), the C30 column yielded the highest number of metabolite annotations (3,876 in total, of which 3,503 were unique to the column). The PHE analysis resulted in 3,590 metabolite annotations (2,404 unique to the column), while analyses performed using the AMD column resulted in the lowest number of metabolite annotations (2,049, of which 1,029 were unique to this column).

Considering the ionization modes used (see Fig. 6C), analysis using positive ionization mode produced 5,846 metabolite annotations (4,902 were unique to this mode), compared to 3,270 metabolite annotations using negative ionization (2,326 unique to this mode). This finding evidences the need to include both ionization modes in the DMA workflow. Unsurprisingly, for both ionization modes the higher mass ranges (>600 Da) are dominated by lipids and lipid-like molecules, whereas for mass ranges <600 Da the organic acids and derivatives are most prominent, along with lipids and lipid-like molecules. Other superclasses with mass <600 Da include organoheterocyclic compounds; organic oxygen compounds; benzenoids; phenylpropanoids and polyketides; organic nitrogen compounds; and nucleosides, nucleotides, and analogues. Supplementary Fig. S29 shows the distributions of unique annotations against the exact mass of the annotation.

When examining each assay (see Fig. 6D), in all cases the analysis of the crude *D. magna* extract (without SPE fractionation) resulted in the highest number of annotations compared to the individual SPE fractions. This is to be expected, as the SPE fractions were intended to separate the metabolites according to their physicochemical properties and so by design will separate the metabolites across fractions. Figure 6D also demonstrates that lipids and lipid-like molecules can be seen to dominate the apolar arm, whereas organic acids and derivatives are the most prominent in the polar arm. This is also expected based on the chemistry of the liquid-phase extractions, solid-phase extractions and chromatography used, which favour apolar metabolites.

Figure 6E shows an UpSet plot detailing the overlap of annotations across assays (with positive and negative ionization modes combined). The most striking observation is the varying number of annotations per assay, with the SPE fractions from the AMD column providing the lowest number of annotations. This assessment could form a basis for a more streamlined, time-efficient workflow. Given that the measurements of the crude extracts yielded 581, 568 and 513 unique annotations (for apolar crude C30, polar crude AMD and polar crude PHE respectively) illustrates their valuable contribution to the DMA workflow.

To provide added confidence in the ability of the experimental and computational workflow to annotate the metabolome, an assessment of the workflow was also made using 48 chemical reference standards covering a wide biochemical space, including lipid and lipid-like molecules; organic acids and derivatives; organic oxygen compounds; organoheterocyclic compounds; nucleosides, nucleotides, and analogues; and organic nitrogen compounds. The spread of the compound classes across the experimental workflow mirrors what is observed in the *Daphnia* samples, where lipids and lipid-like molecules dominate the apolar arm and organic acids and derivatives being the most prominent in the polar arm. As the majority of metabolite reference standards were observed by the DMA workflow (89.6%), we deemed that both the experimental and computational workflows were sufficiently reliable at annotating a diverse range of metabolites to be used for annotating *D. magna* metabolome. See Supplementary Table S11, Figs S30 and S31, and Section 2.6 for further details.

### Comparison to other metabolite databases

**Figure 7:**
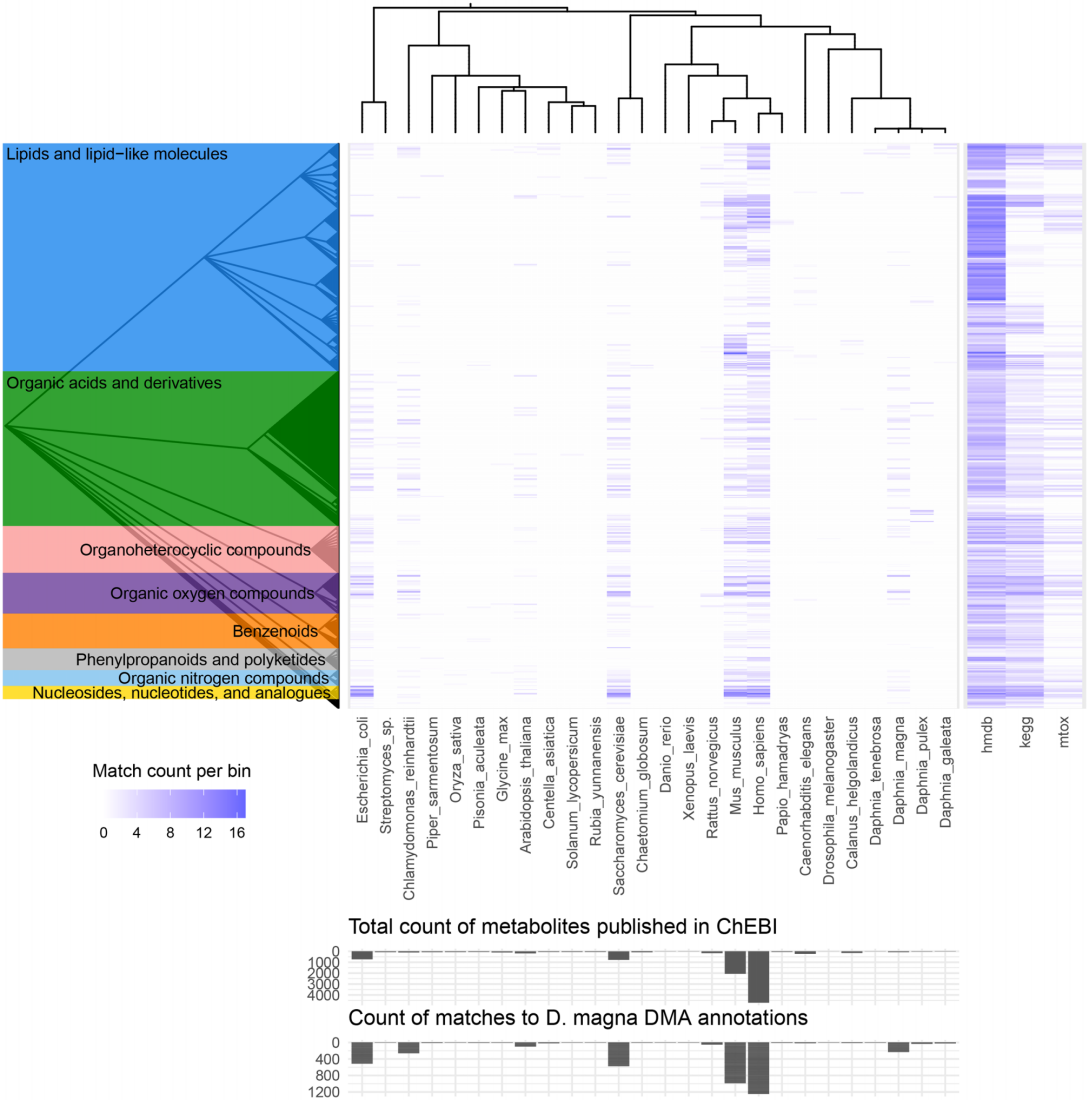
Overlap of *D. magna* metabolite annotations reported using the DMA workflow with known metabolites from other species. Left—all metabolite annotations from the DMA of *D. magna* presented in a hierarchical tree of superclasses, classes and subclasses. Top—phylogenetic tree of known metabolites from 26 species derived from ChEBI. Centre—heatmap of the counts of metabolites matched between the species derived from ChEBI with the *D. magna* metabolites from the DMA workflow (binned into sets of 25 compounds). Right—heatmap of matches observed between HMDB, KEGG and MTox700+ and the *D. magna* metabolite annotations reported using the DMA workflow. Bottom—counts of the metabolites published with ChEBI for each organism and below that the counts of the matches to DMA of *D. Magna* annotations.

The metabolite annotation results from the DMA of *D. magna* were compared to public resources of relevant metabolites from different species, as summarized in Fig. 7. Even given this relatively limited number of metabolites known for different organisms, it is readily apparent that some metabolites are widely shared across organisms due to the conservation of metabolism (i.e., phylometabolomics). From ChEBI, the top six species that overlap with *D. magna* DMA annotations are *Homo sapiens, Saccharomyces cerevisiae, Mus musculus, Escherichia coli, D. magna*, and *Chlamydomonas reinhardtii (a single-celled green algae)*. For *H. sapiens, M. musculus, S. cerevisiae*, and *E. coli*, this is partly explained by these species being the most represented within ChEBI. However, overlap with the previously known *D. magna* and algae metabolites (which are the diet of the cultured *D. magna*) adds confidence to the existing annotations and supports the effectiveness of the DMA workflow.

As *D. magna* is used internationally as an ecotoxicology test species, the metabolite annotations reported using the DMA workflow were compared to those in MTox700+, a metabolite list of toxicologically relevant metabolites derived from mammalian studies. Overlapping metabolites may help interpret the toxicological perturbations measured in *D. magna* metabolomics studies. A total of 293 of 722 metabolites were matched to a full InChIKey (or 346 of 722 if using the first section of the InChIKey).

In addition to the above, the 8,181 annotated metabolites were investigated to assess coverage of known pathways. While we acknowledge the limitations of this analysis due to the limited knowledge of pathways for *Daphnia*, preliminary analysis using the QIAGEN IPA software was still able to identify 113 pathways having ≥50% coverage with ≥3 measured metabolites (see Supplementary Fig. S32), supporting the relevance of the annotated metabolites for pathway-level toxicological interpretation.

### Molecular network analysis using GNPS

**Figure 8:**
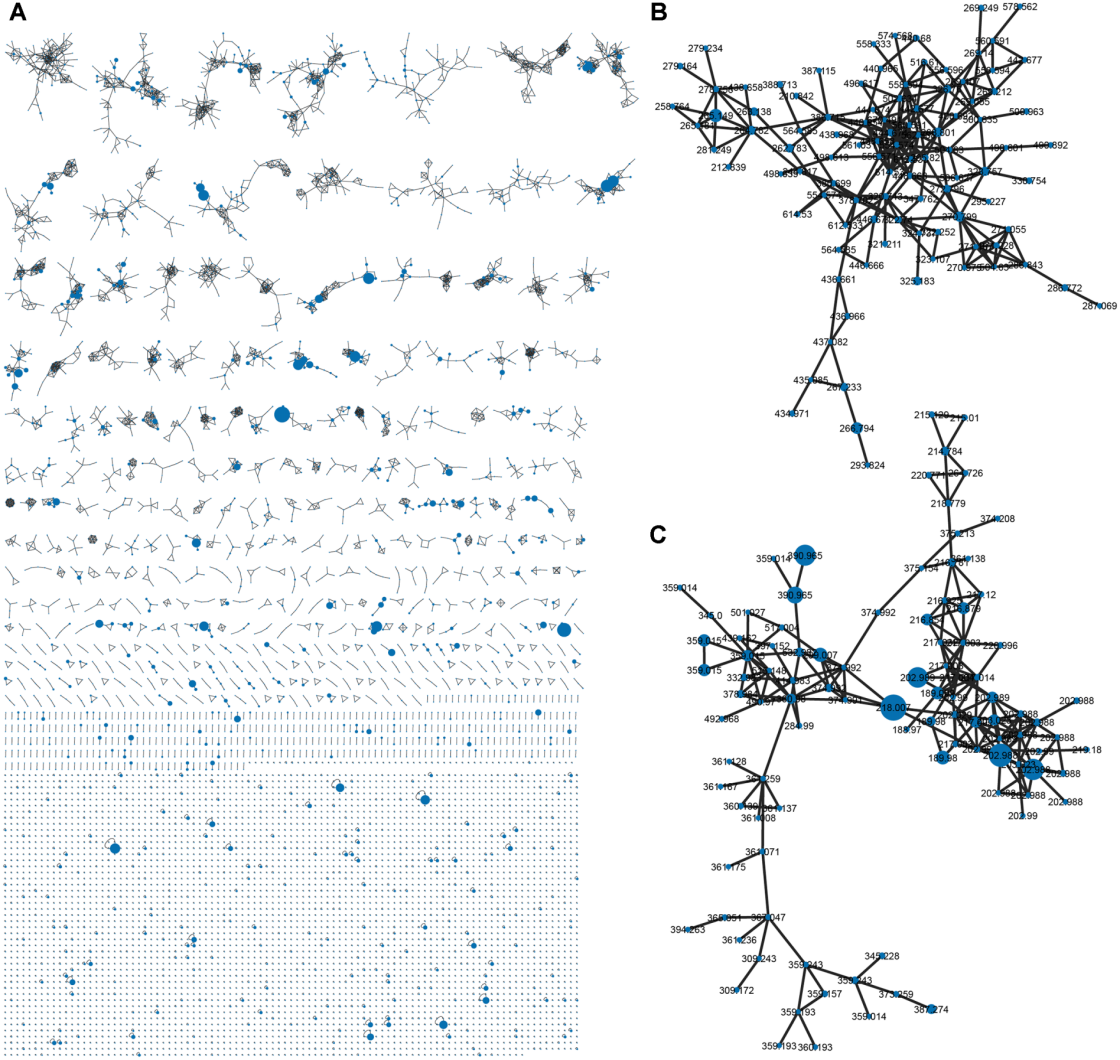
GNPS molecular network derived from *D. magna* DMA negative ionization fragmentation spectra. (A) Overview of negative ionization mode molecular networks generated from GNPS molecular network analysis, showing all 5,320 distinct clusters. The node size is proportional to the number of spectra that contribute to the node. The top 2 clusters (based on how many nodes were observed) are shown in more detail to highlight the precursor m/z associated with the node. (B) Largest cluster observed. (C) Second largest cluster observed.

Molecular networks were generated using the GNPS network analysis workflow, with classical molecular networking, MS2LDA, Dereplicator+ and MolNetEnhancer (see Fig. 8 for summary of negative ionization molecular networks and Supplementary Fig. S33 for positive ionization molecular networks). These networks provide an overall picture of the diversity of the fragmentation spectra collected (and thus the diversity of metabolites observed in *Daphnia*) without being wholly dependent on obtaining a compound or compound class annotation. The dataset exhibited a large diversity of fragmentation spectra (ca. 31,000 fragmentation clusters for positive ionization mode and ca. 5,000 distinct fragmentation clusters for negative ionization mode). A similar overview can be achieved using MS2LDA mass motifs, for which ca. 55,000 “mass motifs” are observed for positive ionization data and ca. 3,900 for the negative ionization data. However, only a small subset of the clusters could be annotated (e.g., ca. 1,000 clusters annotated via spectral matching for positive ionization spectra and ca. 200 clusters annotated for negative ionization spectra). The remaining unannotated spectra may reveal additional insights into the *Daphnia* metabolome as spectral libraries and computational approaches for annotation improve. In particular, the annotations could be improved with further integration with MetFrag and SIRIUS CSI:FingerID, but this was beyond the scope of this analysis. The spectral networks also demonstrate the potential for future analysis where multiple networks created from other model organism DMA projects could be compared for cross-species phylometabolomics analysis that would be driven by spectral similarity as opposed to being reliant on metabolite annotations.

### DMAdb: public access and functionalities

DMAdb [43] serves as a data management environment encompassing raw data, processing workflows, and metabolite annotations (Fig. 4). This integrated framework enhances reproducibility and supports transparent data provenance. By structuring DMA data according to the ISA model, DMAdb provides standardized representations of experimental datasets, thereby promoting interoperability and enabling structured exploration of associated metadata. Public users can access selected functionalities without registration, including browsing ISA-organized datasets and performing basic searches of metabolite annotation records. Registered users are provided with extended functionalities, including access to processed datasets at the individual assay level, derived from the corresponding Galaxy histories. Advanced functionalities further include batch-based exact mass searches across annotated metabolites and MS/MS spectral matching against processed and annotated fragmentation spectra. Although developed specifically for this study to support systematic investigation of the metabolic complexity of *D. magna*, DMAdb establishes a structured foundation for ongoing refinement and future expansion, offering a framework that can be adapted for use with additional model organisms.

## Conclusion

The extensive experimental and computational tools and workflows developed and applied here have generated one of the largest metabolite annotation datasets ever published and provide the first comprehensive list of metabolites thought to be present in the ecotoxicologically important model organism, *D. magna*. The reported 8,181 metabolites (1,301–3,601 if using more stringent filtering criteria), covering predominantly the endogenous *Daphnia* metabolites and potentially some amount of the algae food source and gut microbiome (e.g., the 306 phenylpropanoids and polyketides and the 32 alkaloids and derivatives), represent a significant step forward in understanding the metabolic complexity of *Daphnia*.

The dataset also provides a substantial resource of mass spectrometry fragmentation data focused on a single organism, which can be used for re-analysis and as a source of annotations when compared with other fragmentation datasets, e.g., for cross-species metabolome comparisons (phylometabolomics). Additionally, we have established Galaxy workflows and tools to process and annotate not only this dataset, but also other mass spectrometry fragmentation datasets. Reproducing this complex, multi-step workflow within Galaxy would require substantial effort, particularly for users unfamiliar with the workflow management platform or the specific configuration requirements of this study. However, the sharing of full Galaxy histories and workflows is intended to provide transparency and support methodological inspection and reuse where appropriate. We also note that SIRIUS CSI (version 4), as used here, is no longer publicly accessible, as it depended on an API endpoint that has since been deprecated and is no longer supported. Despite these caveats, the Galaxy tools developed here have already been applied in subsequent metabolomics studies, demonstrating their practical utility [48, 49].

We also present DMAdb as a data management environment for raw data, processing steps, and metabolite annotations, enabling controlled public access to ISA-organized datasets alongside extended functionality for registered users. Although developed specifically for this study, it establishes a potential foundation for the organization and exploration of future DMA datasets, with application beyond *D. magna*.

While our computational workflow is extensive, encompassing multiple techniques and approaches, it has several limitations. In particular, bias arises from the choice of software, software versions, parameter settings, library choice and filtering approaches, all of which influence the final set of reported metabolite annotations. The level of lenience applied in these choices affects the reliability of the annotations, and it is anticipated that some false positives are reported here, i.e., not actual endogenous *Daphnia* metabolites but structurally similar enough to generate an annotation. Purchasing thousands of metabolite reference standards to confirm the annotations is not currently feasible, due to the limited commercial availability of such standards. Also, although steps were taken to minimize background and contaminant signals, further work could explore more stringent criteria. As more metabolite annotations are reported for model organisms, and as their validity becomes established, the reliability of the annotations from the *Daphnia* DMA project can be further evaluated. Improvements to the computational workflow could include greater use of isotopic patterns and in-source fragmentation to reduce spurious annotations [50]; further integration of the underlying software packages into other mass spectrometry-based data analysis suites [51]; and integration with GNPS networking tools where networks could be generated from averaged spectra produced (e.g., via msPurity and MSnPy) rather than individual scans, followed by incorporation of annotations from MetFrag and SIRIUS CSI:FingerID.

The experimental methods described here provide a means to obtain a single homogenous sample representing an organism’s metabolome, which is then characterized through extensive physicochemical separation and bioanalytical measurements. The experimental workflow developed here can be applied in full or adapted in part, depending on the resources available. For example, where sample material is limited, a subset of the SPE methods and/or (U)HPLC-HRMS(/MS) approaches may be selected based on which metabolite classes are of interest, or for a more rapid DMA project, the SPE component could be omitted. We acknowledge that even with this extensive workflow there are limitations in how many metabolites can be fragmented, reducing the number of metabolites that can be annotated using fragmentation-based approaches. However, advances in mass spectrometry technologies may help to address this issue, e.g., the Thermo Scientific Orbitrap ID-X Tribrid mass spectrometer is capable of extensive MS^*n*^ analysis, while current generation Time of Flight/Astral instruments support MS/MS acquisition at up to 250 Hz.

The present study is unlikely to provide an exhaustive list of all possible metabolites present in *D. magna*. In part, this is due to limitations of current analytical platforms, which typically detect only a subset of an organism’s metabolome, e.g., pigment molecules are often difficult to detect using liquid chromatography-mass spectrometry. At the same time, while 10 *D. magna* strains were included in the present work to maximize the breadth of *D. magna* metabolome annotation achieved, and to capture genetic diversity across the species, many of these strains are derived from European *Daphnia* populations. *Daphnia magna* strains from diverse geographic regions (e.g., East Asia, South Africa, and North America) may very well produce a plethora of unique metabolites that are currently missing from the annotations described here. Accordingly, the DMA of *D. magna* should be expanded to include metabolite annotation data, collated using a comparable DMA workflow, both for *Daphnia* originating from a wider variety of geographic locations, and for *Daphnia* maintained under a greater range of environmental or exposure conditions. This would enhance the applicability of this work to *D. magna* metabolomics studies worldwide, while also facilitating exploration of the genetic and environmental basis of metabolic diversity across *D. magna* populations.

Despite the limitations described above, the DMA workflow described here and applied to *D. magna* provides both a resource and a valuable catalyst for future DMA studies of other model organisms.

## Availability of source code and requirements

Project name: dmagna-dma-paper

Project homepage: https://github.com/computational-metabolomics/dmagna-dma-paper

License: GPL-3.0 license

Operating system(s): Operating system independent

Package management: CRAN, Bioconductor and renv

Programming language: R

Hardware requirements: 4 CPU cores and 16 GB RAM

R (v4.4.3) [52] was used to summarize the annotations and generate Figs 5–7, as well as the supplementary summary figures. The code used for this is available via the “dmagna-dma-paper” GitHub repository, where all package requirements are detailed. Key R packages used include: ggplot2 (v3.5.2) [53], which was used throughout the analysis for the generation of plots; UpSetR (v1.4.0) [54] for UpSet plots; VennDiagram (v1.7.3) for Venn diagrams; Treemap (v2.4.4) for treemaps; ggtree (v3.14.0) [55], ape (v5.8.1) [56] and aplot (v0.2.8) [57] were used to generate the plots comparing the DMA of *D. magna* metabolites to the phylogenetic tree of relevant species and map to relevant databases and resources; ChemmineR [58] (v3.58.0) was used to extract the PubChem fingerprints and mol files from PubChem [59], principal component analysis (PCA) was then performed on these fingerprints with the R “prcomp” function. Additionally, this repository includes an example illustrating how data from the Galaxy histories may be accessed programmatically, including retrieval of XCMS peak matrices and the inspection of their relative peak intensities.

See Table 1 for availability of the Galaxy tools used and developed, and Supplementary Table S9 for details of each tool.

## Availability of resources and requirements

Project name: DMAdb

Project homepage: Web portal https://dmadb.bham.ac.uk/; Documentation https://dmadb.readthedocs.io/en/latest/getting-started.html

License: GPL-3.0 license

Operating system(s): Web portal accessibility is operating system independent; Linux is used for the deployment of the DMAdb web server.

Package management: Underlying Django applications are maintained on GitHub (dependencies can be managed via conda and PyPI)

Programming language: Underlying Django applications are developed primarily with Python, alongside HTML and JavaScript

Hardware requirements: Minimum requirements for deployment are 2 CPU cores and 4 GB RAM

DMAdb and web portal were developed using three Django applications (django-gfiles, django-galaxy, and django-mogi) specifically designed for metabolomics data organization with Galaxy and the ISA framework. The web portal and underlying software packages are freely accessible.

## Additional files

Supplementary information is available in the accompanying Word (.docx) file, with larger tables provided separately in the Excel (.xlsx) file (Supplementary Tables S1, S2, S11, and S13).

**Supplementary Section 1: Materials and methods—further details**

1.1: Summary of assays and files

1.2: Chemicals

1.3: Solvents and solutions

1.4: Consumables

1.5: *D. magna* culturing and sample preparation

1.6: Metabolite extraction from homogenized *D. magna* biomass

1.7: Solid-phase extraction-based fractionation of metabolite extracts

1.8: DMA (U)HPLC-HRMS(/MS), DI-HRMS(/MS*^n^*) and LC fractionation

1.9: (U)HPLC-HRMS(/MS) method optimization

1.10: GC-EI-HRMS

1.11: 1D- & 2D-NMR

1.12: DMA computational workflow overview

1.13: DMA Galaxy workflow

1.14: Combining and summarising all annotations

1.15: Assessment of the computational and experimental DMA workflow with metabolite reference standards

**Supplementary Section 2: Results—further details**

2.1: (U)HPLC-HRMS(/MS) method optimization

2.2: Summary of all DMA of *D. magna* annotations

2.3: (U)HPLC-HRMS(/MS) and DI-HRMS(/MS*^n^*) derived metabolite annotations

2.4: GC-EI-HRMS derived metabolite annotations

2.5: NMR derived metabolite annotations

2.6: Assessment of the computational and experimental DMA workflow with metabolite reference standards

2.7: Pathway analysis

2.8: Molecular network analysis using GNPS

**Supplementary tables:**

**Supplementary Table S1:** Assay summary.

**Supplementary Table S2:** (U)HPLC-HRMS(/MS) DI-HRMS(/MS*^n^*) data files.

**Supplementary Table S3:** *D. magna* strains.

**Supplementary Table S4:** High-hardness COMBO and modified high hardness COMBO medium.

**Supplementary Table S5:** Bold’s basal medium.

**Supplementary Table S6:** Liquid chromatography systems utilized in optimization of (U)HPLC-HRMS(/MS) methods.

**Supplementary Table S7:** Mass spectrometer operational parameters for optimization of (U)HPLC-HRMS(/MS) methods.

**Supplementary Table S8:** Liquid chromatography operational parameters for optimization of (U)HPLC-HRMS(/MS) methods.

**Supplementary Table S9:** Summary of Galaxy tools.

**Supplementary Table S10:** Summary of fragmentation spectra used for spectral matching with msPurity.

**Supplementary Table S11:** Metabolite reference standard summary.

**Supplementary Table S12:** Median and interquartile range of retention times for RDMFs recorded in DMA (U)HPLC-HRMS/MS method optimization experiments.

**Supplementary Table S13:** *D. magna* metabolite annotation summary.

**Supplementary Table S14:** GC-EI-HRMS derived metabolite annotations.

**Supplementary Table S15:** NMR derived metabolite annotations.

**Supplementary figures (methods)**

**Supplementary Fig. S1**: Solid-phase extraction-based fractionation of *D. magna* polar extract (WAX, weak anion-exchange; WCX, weak cation-exchange).

**Supplementary Fig. S2**: Solid-phase extraction-based fractionation of *D. magna* apolar extract (C18, a reversed phase-based fractionation procedure; AMP, a weak anion-exchange-based fractionation procedure.

**Supplementary Fig. S3:** Overview of the data acquisition workflow applied for (U)HPLC-HRMS(/MS) analysis and time-based fractionation of DMA samples.

**Supplementary Fig. S4:** Overview of the data acquisition workflow applied for DI-HRMS(/MS^*n*^) analysis of the DMA re-suspended LC fractionation samples.

**Supplementary Fig. S5**: Sample preparation for (U)HPLC-HRMS(/MS) method optimization.

**Supplementary Fig. S6:** Overview of computational analysis of DMA (U)HPLC-HRMS(/MS) and DI-HRMS(/MS*^n^*) LC fractionation experiments.

**Supplementary Fig. S7:** (U)HPLC-HRMS(/MS) data processing schematic for msPurity and XCMS.

**Supplementary Fig. S8:** DI-HRMS(/MS*^n^*) data processing schematic for MSnPy.

**Supplementary figures ((U)HPLC-HRMS(/MS) method optimization results)**

**Supplementary Figs S9–S26:** Multiple figures detailing the (U)HPLC-HRMS(/MS) method optimization. Includes two-dimensional density plot of reproducibly detectable metabolic features (RDMFs) for each method assessed and summary plots of the counts of RDMFs across the different methods.

**Supplementary figures (*D. magna* annotation results)**

**Supplementary Fig. S27:** Venn diagram of metabolite annotations observed for 1D- & 2D-NMR, GC-EI-HRMS, and (U)HPLC-HRMS(/MS) and DI-HRMS(/MS*^n^*) measurement techniques.

**Supplementary Fig. S28:** Venn diagram of metabolite annotations observed across computational annotation approach used.

**Supplementary Fig. S29:** Distribution of unique metabolite annotations across monoisotopic exact mass.

**Supplementary Fig. S30:** Assessment of the DMA experimental and computational workflow.

**Supplementary Fig. S31:** Summary of which annotation approach was able to identify each metabolite standard.

**Supplementary Fig. S32**: Summary of the top canonical pathways derived using QIAGEN Ingenuity Pathway Analysis (IPA) for all annotations obtained from the DMA of *D. magna*.

**Supplementary Fig. S33:** GNPS spectral network analysis (positive ionization mode).

## Abbreviations

1D- & 2D-NMR: 1- and 2-dimensional nuclear magnetic resonance; (U)HPLC-HRMS(/MS): (Ultra)high-performance liquid chromatography-high resolution tandem mass spectrometry; AMD: Accucore Amide liquid chromatography column; AMP: Weak anion-exchange SPE cartridges (apolar arm of workflow); C18: Reversed-phase C18 SPE cartridges; C30: Accucore C30 RPLC column (C30); CID: Collision-induced dissociation; DDA: Data-dependent acquisition; DI-HRMS(/MS*^n^*) : Direct infusion-high resolution mass spectrometry (with multiple-stage fragmentation); DMA: Deep metabolome annotation; DMAdb: Deep metabolome annotation database; GC-EI-HRMS: Gas chromatography-electron ionization-high resolution mass spectrometry; HCD: Higher energy collisional dissociation; HILIC: Hydrophilic interaction liquid chromatography; HRMS(/MS): High resolution mass spectrometry (with tandem mass spectrometry); NCE: Normalized collision energy; RPLC: Reverse-phase liquid chromatography; PHE: Syncronis Phenyl liquid chromatography column; SPE: Solid-phase extraction; WAX: Weak anion-exchange SPE cartridges (polar arm of workflow); WCX: Weak-cation exchange SPE cartridges.

## Supplementary Material

giag055_Supplemental_Files

giag055_Authors_Response_To_Reviewer_Comments_original_submission

giag055_GIGA-D-25-00453_original_submission

giag055_GIGA-D-25-00453_revision_1

giag055_Reviewer_1_Report_original_submission Reviewer 1 -- 11/21/2025

giag055_Reviewer_1_Report_revision_1 Reviewer 1 -- 3/27/2026

giag055_Reviewer_2_Report_original_submission Reviewer 2 -- 11/30/2025

## Data Availability

All raw (U)HPLC-HRMS(/MS) and DI-HRMS(/MS^*n*^) data and selected annotations supporting the results of this article are available through MetaboLights (MTBLS2273). Additionally, the mass spectrometry files containing mass spectrometry gas-phase fragmentation spectra used for the GNPS analysis of the *D. magna* sample (and not equilibration, blank or reference standard samples) are also available through GNPS MassIVE repository (MSV000094957). The assay format was simplified for MetaboLights and MassIVE into four assays (apolar positive, apolar negative, polar positive and polar negative). The Galaxy workflows, histories and details of each tool used in this project are available in the DMA Galaxy instance [31]. The Galaxy analysis performed used the following workflows: • **W1**: The full (U)HPLC-HRMS(/MS), DI-HRMS(/MS^*n*^) and LC fractionation workflow (described in Fig. 3) • **W2**: The (U)HPLC-HRMS(/MS) only workflow • **W3**: The (U)HPLC-HRMS(/MS) workflow used for the metabolite reference standard analysis. Supplementary Table S1 provides the links for the relevant histories and workflow used for each assay. The workflows are also made available on Workflowhub (W1 [63], W2 [64], and W3 [65]) These above resources cover the full Galaxy workflow analysis; however, re-running all analyses, particularly outside of the provided Galaxy instances, would require additional setup due to both the high computational resource demands of this large dataset, as well as software updates to some of the underlying Galaxy tool since the analysis in this manuscript was performed. As such, we also include an example workflow for the most broadly reusable portion of the (U)HPLC-HRMS(/MS) workflow [66] that can be executed on other public instances (e.g., Workflow4Metabolomics [67]). The GitHub repository “dmagna-dma-paper” (see the “Availability of Source Code and Requirements” section) additionally includes the consolidated annotation file spanning all assays that was used for figure generation and the derivation of summary information, along with details on accessing data from the associated Galaxy workflow histories. This repository also includes the numerical data used for all data analysis figures. Additionally, the DMAdb web portal [43] can also be used to access all the raw and processed (U)HPLC-HRMS(/MS) and DI-HRMS(/MS^*n*^) data as well as viewing and searching the fragmentation spectra and metabolite annotations.
