# Supplementary material for: Experimental and computational approaches for deep metabolome annotation with application to the ecotoxicological model organism Daphnia magna
Source: Gigascience. 2026 May 9;15:giag055. doi: 10.1093/gigascience/giag055 (PMC13254479; doi:10.1093/gigascience/giag055)
Supplement: giag055_Supplemental_Files [file giag055_supplemental_files.zip › DMA D. magna - supp info ed revised 01052026.docx]

**Experimental and computational approaches for deep metabolome annotation with application to ecotoxicological model organism *Daphnia magna***

**Supplemental information**

Thomas N. Lawson^1,2,†^, Martin R. Jones^1,†^, Andrew J. Chetwynd^1,3,α^, Elena Sostare^2^, Stefan Weidt^5^, Robert Mistrik^4,δ^, Warwick B. Dunn^1,3,§^, Ralf J. M. Weber^1,3,*^, Mark R. Viant^1,2,3,*^

^1^School of Biosciences, University of Birmingham, Edgbaston, Birmingham, B15 2TT, UK

^2^Michabo Health Science Limited, Union House, 111 New Union Street, Coventry, CV1 2NT, UK

^3^Phenome Centre Birmingham, University of Birmingham, Edgbaston, Birmingham, B15 2TT, UK

^4^HighChem, Mlynské nivy 5, 821 09 Bratislava, Slovakia

^5^Glasgow Polyomics, University of Glasgow, University Avenue, Glasgow, G12 8QQ, UK

^†^Joint first authors

Present addresses: ^α^Centre for Proteome Research, and ^§^Centre for Metabolomics Research, Department of Biochemistry, Cell and Systems Biology, Institute of Systems, Molecular and Integrative Biology, University of Liverpool, Liverpool, L69 7ZB, UK; ^δ^Bitmoderna, Leskova 11, 81104 Bratislava, Slovakia.

*Correspondence address: School of Biosciences, University of Birmingham, Edgbaston, Birmingham, B15 2TT, UK; E-mail: [m.viant@bham.ac.uk](mailto:m.viant@bham.ac.uk); E-Mail: [r.j.weber@bham.ac.uk](mailto:r.j.weber@bham.ac.uk)

**Table of contents**

[1 Supplemental – materials and methods 3](#_Toc228549309)

[1.1 Summary of assays and files 4](#_Toc228549310)

[1.2 Chemicals 4](#_Toc228549311)

[1.3 Solvents and solutions 4](#_Toc228549312)

[1.4 Consumables 5](#_Toc228549313)

[1.5 *D. magna* culturing and sample preparation 5](#_Toc228549314)

[1.6 Metabolite extraction from homogenised *D. magna* biomass 10](#_Toc228549315)

[1.7 Solid phase extraction-based fractionation of metabolite extracts 11](#_Toc228549316)

[1.8 DMA (U)HPLC-HRMS(/MS), DI-HRMS(/MS^n^) and LC fractionation 14](#_Toc228549317)

[1.9 (U)HPLC-HRMS(/MS) method optimisation 25](#_Toc228549318)

[1.10 GC-EI-HRMS 36](#_Toc228549319)

[1.11 1D- & 2D-NMR 38](#_Toc228549320)

[1.12 DMA computational workflow overview 40](#_Toc228549321)

[1.13 DMA Galaxy workflow 41](#_Toc228549322)

[1.14 Combining and summarising all annotations 60](#_Toc228549323)

[1.15 Assessment of the computational and experimental DMA workflow with metabolite reference standards 61](#_Toc228549324)

[2 Supplemental - results 62](#_Toc228549325)

[2.1 (U)HPLC-HRMS(/MS) method optimisation 63](#_Toc228549326)

[2.2 Summary of all DMA of D. magna annotations 89](#_Toc228549327)

[2.3 (U)HPLC-HRMS(/MS) and DI-HRMS(/MS^n^) derived metabolite annotations 90](#_Toc228549328)

[2.4 GC-EI-HRMS derived metabolite annotations 92](#_Toc228549329)

[2.5 NMR derived metabolite annotations 94](#_Toc228549330)

[2.6 Assessment of the computational and experimental DMA workflow with metabolite reference standards 101](#_Toc228549331)

[2.7 Pathway analysis 104](#_Toc228549332)

[2.8 Molecular network analysis using GNPS 105](#_Toc228549333)

[3 References 107](#_Toc228549334)

# Supplemental – materials and methods

## Summary of assays and files

See **Supplemental Table S1** (provided in separate excel file) for a summary of all experimental assays performed.

See **Supplemental Table S2** (provided in separate excel file) for the (ultra)-high-performance liquid chromatography-high resolution tandem mass spectrometry ((U)HPLC-HRMS(/MS)) and the direct infusion-high resolution mass spectrometry with multiple-stage fragmentation (DI-HRMS(/MS^n^)) data files used.

## Chemicals

Acetic acid (≥ 99.0% w/w, TraceSelect grade), ammonium acetate (≥ 99.99%, trace metals basis), ammonium formate (≥ 99.9% w/w trace metals basis), ammonium hydroxide (≥ 25.0% w/w), boric acid (98.5% for molecular biology), calcium chloride dihydrate (ReagentPlus, ≥ 99.0%), cobalt (II), nitrate hexahydrate (laboratory grade), copper (II) sulfate pentahydrate (laboratory grade), ethylenediaminetetraacetic acid tetra-sodium salt (≥ 99.0%), formic acid (≥ 98% w/w, TraceSelect grade), lithium chloride anhydrous (BioUltra for molecular biology), magnesium sulfate heptahydrate (BioXtra, ≥ 99.0%), manganese (II) chloride tetrahydrate (ReagentPlus, ≥ 99.0%), potassium phosphate dibasic (ACS reagent, ≥ 98%), potassium phosphate monobasic (≥ 99.0%), potassium chloride (ACS reagent, 99.0-100.5%), potassium hydroxide (≥85.0%, pellets), potassium iodide (BioUltra, ≥99.5%), rubidium chloride (≥99%, AT), sodium bicarbonate, sodium bromide (99%), sodium selenite, strontium chloride hexahydrate (ACS reagent, 99%), sulfuric acid and zinc (II) sulfate were all purchased from Sigma Aldrich. Iron (II) sulfate heptahydrate (≥ 99%) and sodium metasilicate nonahydrate were purchased from Acros Organics. Sodium molybdenum oxide dihydrate (ACS, 99.5-103.0% ) was sourced through Alfa Aesar. Sodium chloride (analytical reagent grade), sodium phosphate monobasic (anhydrous, Enzyme-grade (99%), and sodium phosphate dibasic (anhydrous, Bioreagent, ≥99.0%) were sources from Fisher Scientific. Sodium-3-(trimethylsilyl)-proprionate-2,2,3,3-d_4_ (98%) was purchased from Cambridge Isotope Laboratories Inc.

## Solvents and solutions

All solvents used in metabolite extractions, solid phase extractions, (U)HPLC-HRMS(/MS) and DI-HRMS(/MS^n^) analyses were of HPLC-grade or better: water (various vendors), methanol (various vendors), chloroform (Fisher Scientific, HPLC grade, 99.8% stabilised with amylenes), propan-2-ol (Fisher Scientific, Optima (U)HPLC-HRMS(/MS), 99.9%),.n-hexane (Acros Organisc, 97%+ for HPLC), diethyl ether (Sigma Aldrich, Chromasolv for HPLC, ≥99.9%, inhibitor free), ethanol (Fisher Scientific, HPLC-grade, ‘Ethanol absolute’), and phosphoric acid (Fluka Analytical, for HPLC (85-90%).

All mass spectrometer calibration solutions were purchased from Thermo Fisher Scientific.

## Consumables

HyperSep WCX (carboxylic acid), HyperSep WAX (aminopropyl) and HyperSep C18 SPE cartridges (3 mL, 500 mg), and Syncronis Phenyl (2.1 x 100 mm, 1.7 µm), Accucore Amide (2.1 x 100 m, 2.6 µm) and Accucore C30 (2.1 x 100 mm, 2.6 µm) LC columns, were provided by Fisher Scientific (Hemel Hempstead, UK).

Easy Pierce foil (20 µm) and 384-well polypropylene well plates (AB-Gene) were purchased from Thermo Scientific. Glass champagne vials (1.5 mL, clear, VZM-1509CC-100) and caps (9-425 Blue Screw Thread with PTFE/silicone/PTFE septa), used in NMR experiments, were purchased from Cronus. ACQUITY UPLC 700 µL 96-well deep well plates, used for LC fraction capture and storage, were purchased from Waters. Polypropylene microfuge tubes (1.5 mL, clear, Safe-Lock) were bought from Eppendorf. Aluminium self-adhesive well plate sealing tape was purchased from Corning.

## *D. magna* culturing and sample preparation

Stock cultures of ten *D. magna* isolates (see **Table S3**) were maintained in modified high-hardness COMBO medium (Baer and Goulden, 1998) (see **Table S4**) at 20 +/- 2 °C and under a 16:8 hr light:dark cycle, at a target density of 20 female *D. magna* per 1.2 L of medium. A concentrated isolate of *Chlorella vulgaris,* (equivalent to 0.5 mg carbon per litre and cultured under non-axenic conditions in Bold’s Basal medium – see **Table S5**) was supplied as feed material, at an equivalent daily dose of*:* 0.5 mL/day for days 1-2; 0.75 mL/day for days 3-7 and; 1 mL/day for days 8 onwards. Culture medium was replaced weekly, between which neonates and any unexpected male daphniids were carefully removed. Each *Daphnia* stock culture was derived from an existing stock culture by transferring twenty < 24 hr neonates (third brood or later) to freshly aerated culture medium. Under these conditions, cultures comprised almost exclusively parthenogenetic females. All cultures were visually inspected daily for potential bacterial or fungal contamination. Where contamination was evident or suspected, cultures were promptly discarded.

*D. magna* used in DMA experiments were derived from corresponding stock cultures by transferring 20 neonates (age < 24 hr, third brood or later) into fresh culturing medium. These experimental cultures were maintained as per stock cultures until day 14, at which point adult *D. magna* were transferred to fresh medium in the absence of food. Adults were then maintained without food for two days, under one of two conditions, either: 1) 20 +/- 2 °C with a 16:8 hr light:dark ratio for 48 hr (‘basal’ metabolome); or 2) 10 +/- 1 °C and with 16:8 hr light:dark ratio for 24 hr, then 10 +/- 1 °C and with 8:16 hr light:dark ratio for a further 24 hr (‘stressed’ metabolome). Thereafter, remaining female daphniids were collected and metabolically quenched.

To quench metabolic processes at the end of the culturing period, *Daphnia* were rapidly flash frozen in liquid nitrogen. To do so, *D. magna* cultures were first passed through a coarse gauze mesh to isolate *D. magna* from their culturing medium. *D. magna*, trapped on the mesh, were then blot dried with tissue paper to remove excess media and transferred to polypropylene tubes using a fine-haired paintbrush, wherein they were submerged in liquid nitrogen. Resulting samples were either used immediately or stored at -80 °C, until required. Excess stock culture daphniids used in DMA workflow optimisation experiments (“**Development samples”**), and daphniids included in the final DMA workflow application experiments (“**DMA samples”**), were all collected using this process.

Prior to metabolite extraction, *D. magna* samples were cryogenically cooled in liquid nitrogen and homogenised over dry ice using a ceramic mortar and pestle. Resulting homogenate was transferred to a pre-cooled 28 mL glass vial for metabolite extraction.

DMA samples were homogenised in two batches, with approximately 50% of DMA samples homogenised in each. Approximately 60% of each resulting homogenate was transferred to a single 28 mL glass vial for extraction of “polar” metabolites, with the remainder transferred to two separate 28 mL glass vials for extraction of “apolar” metabolites.

**Table S3**: *D. magna strains*

| **Geographic origin** | **Supplier** | **Strain name** |
| --- | --- | --- |
| Antwerp, Belgium | University of Antwerp. | AW |
| Birmingham, UK | University of Birmingham. | B1 |
| Birmingham, UK | University of Birmingham. | B2 |
| Kent, UK | Blades Biological – Commercial Supplier. | BD |
| California, USA | University of California, Berkeley. | BK |
| Minnesota, USA | US Environmental Protection Agency (EPA). | EPA |
| Reading, UK | University of Reading. | RD |
| Okazaki, Japan | Okazaki Institute for Integrative Science, National Institute of Natural Sciences. | NS |
| Alessandria, Italy | Universitá del Piemonte Orientale. | UPO |
| UK | Industrial Strain. | IS |

**Table S4**: High-hardness COMBO and modified high hardness COMBO medium

|  | **Compound** | **Stock (g/L)** | **Final medium** | | **Component**  **in final medium** | | | **Volume added per 1 L of medium** |
| --- | --- | --- | --- | --- | --- | --- | --- | --- |
|  |  |  | **mg/L** | **µmol/L** | **Symbol** | **mg/L** | **µmol/L** |  |
| Major stocks | CaCl2.2H2O | 110.28 | 110.28 | 750 | Ca | 30.1 | 750 | 1 mL |
|  |  |  |  |  | Cl | 53.2 | 1500 |  |
|  | MgSO4.7H2O | 55.45 [113.5] | 55.45 [113.5] | 225 [461] | Mg | 5.5 [11.2] | 225 [461] | 1 mL [1 mL] |
|  |  |  |  |  | SO4 | 21.6 [44.2] | 225 [461] |  |
|  | K2PO4 | 1.742 | 1.742 | 10 | K | 0.8 | 20 | 1 mL |
|  |  |  |  |  | P | 0.3 | 10 |  |
|  | NaNO3 | 17 | 17 | 200 | Na | 4.6 | 200 | 1 mL |
|  |  |  |  |  | NO3 | 12.4 | 200 |  |
|  | NaHCO3† | 126 | 126 | 1500 | Na | 34.5 | 1500 | 1 mL |
|  |  |  |  |  | CO3 | 90 | 1500 |  |
|  | Na2SiO3.9H2O | 28.42 | 28.42 | 100 | Na | 4.6 | 200 | 1 mL |
|  |  |  |  |  | Si | 2.8 | 100 |  |
|  | KCl | 5.96 | 5.96 | 80 | K | 3.1 | 80 | 1 mL |
|  |  |  |  |  | Cl | 2.8 | 80 |  |
|  | H3BO3 | 24 | 24 | 388 | B | 4.2 | 388 | 1 mL |
| ANIMATE ‡ | LiCl | 0.31 | 0.31 | 7.31 | Li | 0.05 | 7.313 | \|  \|  \|  \|  \|  \|  1 mL  \|  \|  \|  \|  \|  \| |
|  |  |  |  |  | Cl | 0.26 | 7.313 |  |
|  | RbCl | 0.07 | 0.07 | 0.58 | Rb | 0.05 | 0.579 |  |
|  |  |  |  |  | Cl | 0.02 | 0.579 |  |
|  | SrCl2.6H2O | 0.15 | 0.15 | 0.56 | Sr | 0.05 | 0.563 |  |
|  |  |  |  |  | Cl | 0.04 | 1.125 |  |
|  | NaBr | 0.016 | 0.016 | 0.16 | Na | 0.004 | 0.156 |  |
|  |  |  |  |  | Br | 0.0124 | 0.156 |  |
|  | KI | 0.0033 | 0.0033 | 0.02 | K | 0.0008 | 0.02 |  |
|  |  |  |  |  | I | 0.0025 | 0.02 |  |
| VIM Ŧ | *d-biotin* | 0.1042 | [0.0005] | [0.002] | - | - | - | \|  [0.5 mL]  \| |
|  | Cyanocobalamin (B12) | 0.1124 | [0.00055] | [0.0004] | - | - | - |  |
|  | Thiamine | 0.2 | [0.1] | [0.3] | - | - | - |  |
|  | Na2SeO3Ŧ | 0.04 | 0.002 | 0.0875 | Na | 0.008 | 0.348 | 50 µL |
|  |  |  |  |  | Se | 0.0275 | 0.348 |  |
|  |  |  |  |  |  |  |  |  |
| † Heat to dissolve; ‡ ANIMATE: Animal trace elements; Ŧ VIM: Vitamins – only added to high-hardness COMBO medium; * Sodium selenite was added to high hardness and modified high hardness COMBO medium to enhance Daphnia reproductive output (Keating and Dagbusan, 1984). Values in square braces are for high hardness COMBO medium only (i.e. distinct from the values for modified high hardness COMBO medium). All culturing media was prepared using 15 MΩ deionised water (dH2O), to which was added 1 ml of each major stock and 50 µl of sodium selenite, per one litre of prepared medium. All media were aerated for a minimum of ten hours before adjustment to pH 7.75 ± 0.1 using hydrochloric acid and sodium hydroxide. Finally, 1 ml of ANIMATE stock solution was added per litre of medium, immediately prior to use. | | | | | | | | |

**Table S5**: Bold’s basal medium

|  |  | **Stock solution** | |  | **Bold's Basal Medium (BBM)†** | |
| --- | --- | --- | --- | --- | --- | --- |
| **Solution No.** | **Formula** | **g/L** | **mmol/L** | **Volume added per 1 L BBM (mL)** | **g/L** | **mmol/L** |
| 1 | K_2_HPO_4_ | 7.5 | 43.1 | 10 | 0.075 | 0.43 |
| 2 | KH_2_PO_4_ | 17.5 | 128.6 | 10 | 0.175 | 1.29 |
| 3 | MgSO_4_.7H_2_O | 7.5 | 30.4 | 10 | 0.075 | 0.3 |
| 4 | NaNO_3_ | 25 | 294.2 | 10 | 0.25 | 2.94 |
| 5 | CaCl_2_.2H_2_O | 2.5 | 17 | 10 | 0.025 | 0.17 |
| 6 | NaCl | 2.5 | 42.8 | 10 | 0.025 | 0.43 |
| 7 | EDTA - Na_4_ | 50 | 131.5 | 1 | 0.5 | 1.32 |
|  | KOH | 31 | 552.5 |  | 0.31 | 5.52 |
| 8 | FeSO_4_.7H_2_O | 5 | 17.9 | 1 | 0.0498 | 0.18 |
|  | H_2_SO_4_ | 1 mL |  |  |  |  |
| 9 | H_3_BO_3_ | 11.4 | 184.7 | 1 | 0.1142 | 1.85 |
| 10 | ZnSO_4_.7H_2_O | 14.1 | 49.1 | 0.1 | 0.1412 | 0.49 |
| 11 | MnCl_2_.4H_2_O | 2.3 | 11.7 | 0.1 | 0.0232 | 0.12 |
| 12 | CuSO_4_.5H_2_O | 2.5 | 10.1 | 0.1 | 0.0252 | 0.1 |
| 13 | Co(NO_3_)_2_.6H_2_O | 0.8 | 2.7 | 0.1 | 0.008 | 0.03 |
| 14 | Na_2_MoO_4_­.2H_2_O | 1.9 | 7.9 | 0.1 | 0.0192 | 0.08 |
| † The pH of the final BBM media was maintained in the range 6.7 ± 0.3 (6.4-7.0) by drop-wise addition of either hydrochloric acid or sodium hydroxide. All BBM was autoclaved (121 °C) before use. | | | | | | |
|  |  |  |  |  |  |  |
|  |  |  |  |  |  |  |

## Metabolite extraction from homogenised *D. magna* biomass

Two distinct procedures were applied for the extraction of metabolites from homogenised *D. magna* biomass. The first, aimed at extracting polar through to moderately-polar metabolites, involved addition of ice-cold methanol, then ice-cold water, to homogenised *D. magna* biomass to yield a 2.5:1 v/v methanol:water solution (accounting for approximately 80% w/w water content of an adult *D. magna*)**,** at 15 parts solution per 1 part biomass (v/w). The extraction vial was capped, vortex-mixed for 30 seconds (40 Hz, Thermo Scientific) and centrifuged for 10 minutes at 3000 rpm (approximately 1600 *x g*) and 4 °C. Supernatant was carefully transferred to a clean, ice-cold 28 mL glass vial. This “polar extract” was distributed equally across 1.5 mL polypropylene microfuge tubes, such that each tube contained a proportion of the total extract equivalent to 20 *D. magna*. Polar extract aliquots were dried in a SpeedVac at 35 °C and then transferred to a -80 °C freezer for storage.

The “apolar extraction” procedure, derived from that described by Bligh and Dyer (1959) was applied to extract moderately-polar through to highly-apolar metabolites. Ice-cold chloroform was added to homogenate using a graduated glass pipette, followed by ice-cold methanol, to give final solvent ratio of 1:1 v/v chloroform:methanol and a final extraction ratio of 30 parts extraction solution per 1 part homogenate (assumptions as per the polar extraction procedure, detailed above). The extraction vial was then capped and vortex-mixed for 30 seconds (40 Hz), followed by centrifugation for 10 minutes at 4000 rpm (approximately 2800 x *g*) and 4 °C. The supernatant was transferred to an ice-cold, 28 mL glass vial using a glass Pasteur pipette. Next, ice-cold water was added to the supernatant (taking into account the approximate water content of daphniids) to induce phase separation, with the extraction solution consisting of 1:1:0.9 v/v/v chloroform:methanol:water. After vortex mixing for 30 seconds, vials were centrifuged at 3000 rpm (approximately 1600 x *g*) for 10 mins at 4 °C and then allowed to stand on a laboratory bench for 5 minutes at room temperature. Using a 500 µL glass gas-tight syringe (Hamilton), the upper (polar) and lower (apolar) layers were transferred to separate, pre-cooled 28 mL glass vials. The syringe was rinsed before and between transfer of each layer, first using ten full volumes of methanol and then ten full volumes of chloroform. The upper and lower layers were independently split into equivolume aliquots across glass vials and tubes, respectively, such that each contained the equivalent of 20 *D. magna.* The upper ‘polar’ layer aliquots were dried under vacuum at 35 °C (Speedvac, Thermo Scientific), while the lower ‘non-polar’ layer aliquots were dried under a gaseous stream of nitrogen and then capped after flushing with nitrogen gas. Dried aliquots were stored frozen at -80 °C.

For both the polar and apolar extraction procedures, methodological blank samples were prepared by replacing *D. magna* biomass with water. The volume of water added was such that it corresponded to 80% of the total wet weight of the extracted *D. magna* biomass (assuming one adult *D. magna* has a wet mass of 1.5 mg).

For DMA samples, dried extracts resulting from the “polar” and “apolar” extraction procedures were termed “crude” extracts.

## Solid phase extraction-based fractionation of metabolite extracts

Solid phase extraction (SPE) was used to fractionate metabolite extracts prior to analysis. The ‘polar extract’ was fractionated using weak anion-exchange (WAX; aminopropyl) and weak-cation exchange cartridges (WCX; carboxylic acid) SPE cartridges, while the ‘apolar extract’ was fractionated with aminopropyl weak anion-exchange (referred to as AMP to distinguish from the polar arm; aminopropyl) and reversed-phase C18 cartridges (C18). All SPE fractionation procedures were applied independently (i.e. not chained) using cartridges of 3 mL total volume and packed with 500 mg sorbent material. Solvents and solutions were passed through cartridges at approximately 1 mL/min.

A total of *n* dried extracts (maximum *n* = 10) were resuspended for SPE-based fractionation over a single SPE cartridge. Extracts, *x*, were ordered from *x* = 1 to *x* = *n*. In all but the final resuspension step, resuspension solvent or solution was added to *x* = 1, vortex mixed for 30 seconds and then transferred to *x* +1. This procedure was repeated until *x* = *n*, at which point, the resuspended extract was transferred to a glass collection vial (8 mL). In the final resuspension step, all *n* extracts received resuspension solvent, followed by vortex mixing for 30 seconds and centrifugation at 3000 x g for 10 min at 4 °C. The resulting supernatants were transferred to the collection vial. All solvents, solutions and extracts were kept of wet ice throughout the resuspension procedure.

**Figures S1** and **S2** provide an overview of each SPE-based fractionation procedure, including: sample resuspension procedures; volumes and types of solvents or solutions used, and; fractions resulting from each stage.

Fractions eluted from SPE cartridges were divided into equivolume aliquots, such that each contained metabolite quantities equivalent to ca. 20 *D. magna*. Aliquots for polar SPE extracts were transferred to polypropylene tubes and dried using a SpeedVac (Thermo Scientific) at 35 °C. Apolar fractions were transferred to 1.75 mL glass vials and dried under a gentle stream of gaseous nitrogen at room temperature. Dried fractions were stored frozen at -80 °C.


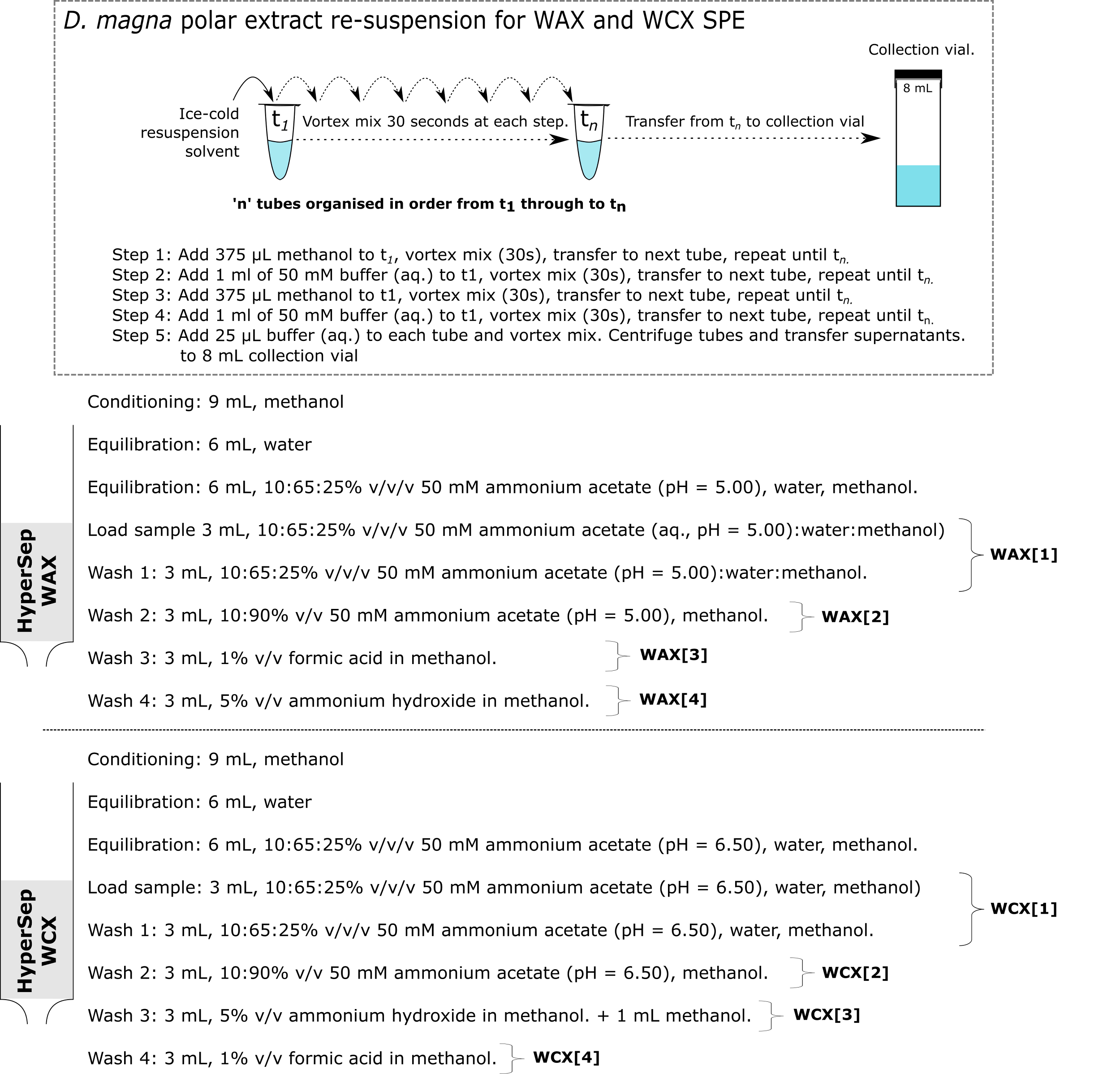


***Figure S1: Solid phase extraction-based fractionation of D. magna* polar extract *(WAX, weak anion-exchange; WCX, weak cation-exchange)***


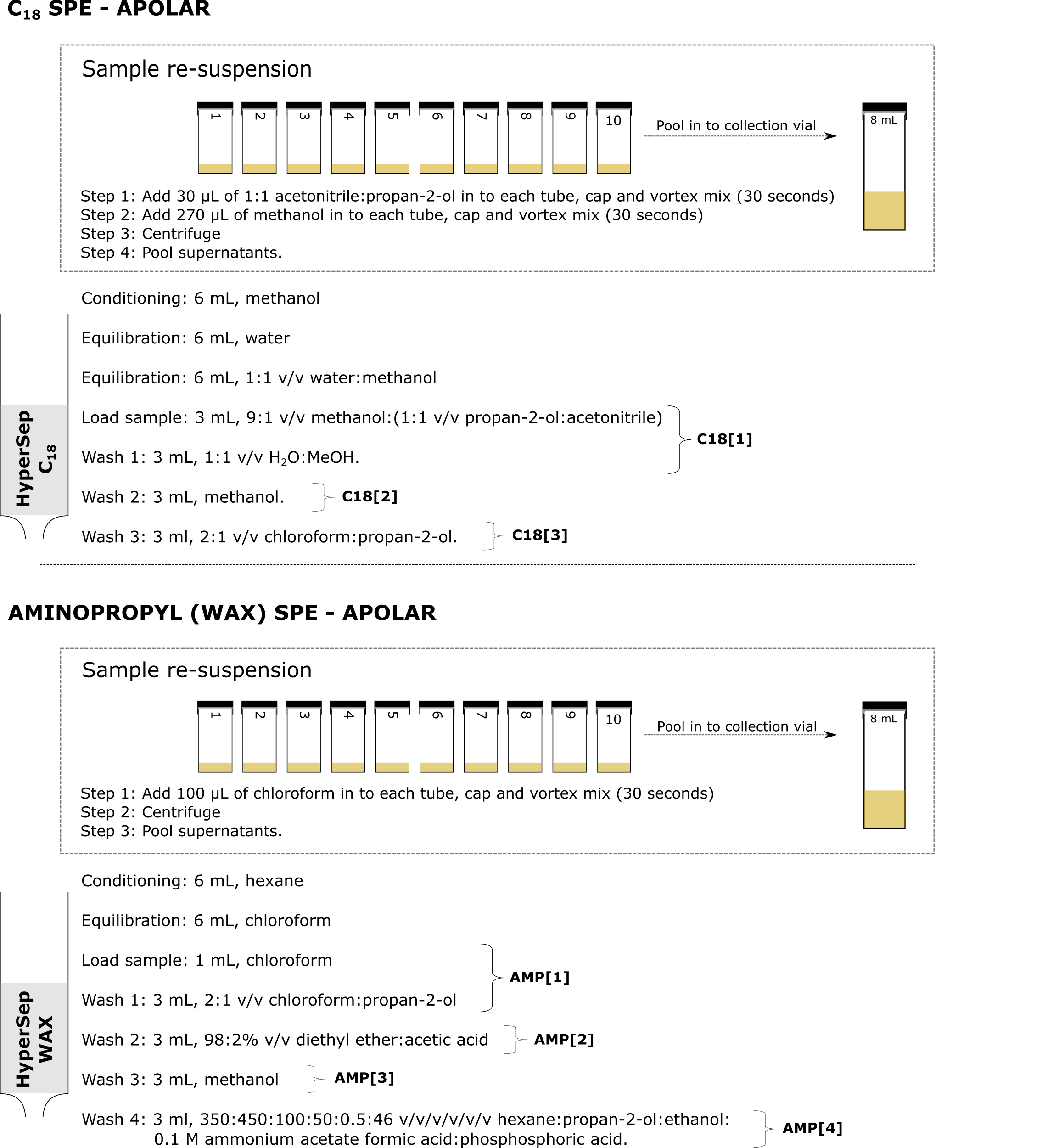


***Figure S2: Solid phase extraction-based fractionation of D. magna apolar extract (C18, a reversed phase-based fractionation procedure); AMP, a weak anion-exchange-based fractionation procedure***

## DMA (U)HPLC-HRMS(/MS), DI-HRMS(/MS^n^) and LC fractionation

### (U)HPLC-HRMS(/MS)

#### (U)HPLC-HRMS(/MS) analytical setup and chromatographic methods

Three distinct (U)HPLC-HRMS(/MS) methods were used for untargeted metabolomics analysis of metabolite extracts (‘crude’ extracts) and associated SPE fractions, derived from DMA experimental samples. All chromatographic separations were performed using an Ultimate 3000 liquid chromatograph with in-line degasser, column oven, and fraction collection-enabled autosampler modules (system ‘FC-LCsys’ in **Table S6**; 20 microliter loop and maintained at 10 °C). A Q Exactive mass spectrometer, fitted with a heated electrospray ionisation source, was used for high resolution-full scan (HRMS) and -tandem (HRMS/MS) mass spectrometry analyses of eluted metabolites. All injections were performed using a custom-written injection procedure, in which 10 µL of sample was injected between a plug of mobile phase A.

Samples were resuspended (on wet ice) by vortex mixing for approximately 30 seconds with ice-cold resuspension solution: for Syncronis Phenyl-based RPLC-HRMS(/MS) this was 120 µL mobile phase A; for Accuore Amide-based HILIC-HRMS(/MS) analyses this comprised 24 µL aqueous buffer (i.e. 100 mM ammonium acetate + 2% v/v acetic acid in water), plus 96 µL acetonitrile and 20 µL methanol (see **Supplemental section 1.9.3.3** for resuspension details); for Accucore C30-based RPLC-HRMS(/MS), this was 120 µL of 1:1 v/v acetonitrile:propan-2-ol. Samples were centrifuged at 21885 x g and 4 °C for 10 minutes, followed by transfer of supernatants to glass HPLC vials with glass inserts.

Throughout (U)HPLC-HRMS(/MS) analyses of each DMA sample type, LC fractions were automatically collected. This was achieved by installing a passive tee-piece device between the LC column outlet and mass spectrometer inlet, such that approximately 30-50% of post-column eluent was passed towards the mass spectrometer, while the remainder returned towards the LC autosampler’s dual injector/fraction collector assembly. The latter automated the collection of LC fractions into individual wells of a deep well plate (Eppendorf, 700 µL deep-well polypropylene plates), with each LC fraction spanning a 20 second window. Fraction collection commenced 15 seconds into each (U)HPLC-HRMS(/MS) analysis to allow sufficient time for external rinsing of the injector/fraction collector assembly with needle wash solution. Across replicate analyses of each DMA sample type, under each analysis configuration i.e. positive mode HRMS, negative mode HRMS, positive mode HRMS/MS, and negative mode HRMS/MS, LC fractions were pooled into individual wells of a single fraction collection plate (see **Figure S3**). Upon completion of each (U)HPLC-HRMS(/MS) analysis of each DMA sample type, the corresponding LC fraction collection plates were either dried immediately using a Speedvac system (40 °C), or were briefly stored refrigerated (ca. 4 °C; heat sealed at 174 °C for 2 s with 20 µm EasyPierce foil, Thermo Scientific) until the Speedvac system was available.

HPLC-HRMS(/MS) analyses of apolar extract and associated SPE fractions were performed using an Accucore C30 LC column (C30) (2.1 x 100 mm, 2.6 µm solid-core particle, 150 Å; Thermo Scientific). The column was maintained at 35 °C throughout, with metabolites eluted using a binary linear gradient supplied at 500 µL/min with the following profile (time in minutes, %B in parentheses): 0 (22%), 6 (60%), 14 (85%), 23 (100%), 26 (100%), 26.1 (22%), 30 (22%). Mobile phase A consisted of 49.5:49.5:1% v/v/v water:acetonitrile:buffer, mobile phase B consisted of 88:10:2% v/v/v propan-2-ol:acetonitrile:buffer, with buffer in each case comprising 99:1% v/v 100 mM ammonium acetate (aq.):acetic acid. Full-scan HRMS data were acquired with the following settings: 200-1200 m/z scan range; 70000 resolution (FWHM200m/z); 1e6 AGC target; 100 ms maximum ion injection time; 20 second chromatographic peak width. Data-dependent HRMS/MS scans were performed for the top-10 most abundant ions from each full-scan using the following parameters: minimum m/z fixed at 50 m/z; 35000 resolution (FWHM_200 m/z_); 1e5 AGC target; 1.3e4 intensity threshold; 1% underfill ratio; fixed first mass of 75 m/z; isolation window of 1 m/z and offset of 0 m/z; stepped normalized collision energies of 20, 30 and 40% in negative ionisation mode, and 25 and 30% in positive ionisation mode; dynamic exclusion of 8 seconds; apex trigger minimum of 4 seconds and maximum of 12 seconds; ‘pick others’ enabled; ‘peptide match’ off; ‘Exclude isotopes’ on; ‘charge exclusion’ off. The H-ESI source was operated as follows: sheath gas 20 arbitrary units (AU), auxiliary gas 5 AU, sweep gas 1 AU, spray voltage ±3 kV, S-lens 45%, capillary temperature 285 °C, and heater temperature 250 °C. S-lens RF level was set at 45%.

‘Polar extract’ and associated SPE fractions derived from DMA samples, underwent untargeted metabolomics analyses using two optimised (U)HPLC-HRMS(/MS) methods. The first, a reversed-phase UHPLC method, was based on a Syncronis Phenyl column (PHE) (2.1 x 100 mm (i.d. x L), 1.7 µm, Thermo Scientific) maintained at 40 °C. Metabolites were eluted from this column using a binary linear gradient supplied at 400 µL/min with the following profile (time in minutes, %B in parentheses): 0 (0%), 1.5 (0%), 22 (100%), 25 (100%), 26 (0%), 30 (0%). Mobile phases A consisted of 90:5:5% v/v/v water:methanol:buffer, while mobile phase B comprised 5:95% v/v buffer:methanol. In each case, buffer comprised an aqueous solution of 100 mM ammonium acetate adjusted to pH 5.8 using 100 mM acetic acid (aq.). The autosampler capillary was cleaned between injections using 100 µL of 80:20% v/v methanol:water solution (needle wash solution). With regards to HRMS(/MS) data acquisition, full-scan data were acquired between 100 and 1000 m/z at a resolution of 70000 (FWHM_200 m/z_) and with AGC target of 3e6, using a maximum ion injection time of 200 ms, S-lens RF 70%, and a single microscan. Data-dependent HRMS/MS data were collected for the top-3 most abundant ions in each full scan, with: ion intensity threshold ≥ 2e4; minimum m/z fixed at 50 m/z; 35000 resolution (FWHM_200m/z_); 2e5 AGC target; 100 ms maximum ion injection time; 1 m/z isolation window (0 m/z offset); stepped normalized collision energies of 30 and 50%; 8 second dynamic exclusion; ‘pick others’ enabled; apex trigger with minimum of 2 seconds and maximum of 6 seconds; ‘exclude isotopes’ on; ‘charge exclusion’ off; ‘peptide match’ off. The H-ESI source was operated as follows: sheath gas 20 arbitrary units (AU), auxiliary gas 5 AU, sweep gas 0 AU, spray voltage +3.5 kV in positive ionisation mode and -3 kV in negative ionisation mode, S-lens 70%, capillary temperature 320 °C, and heater temperature 200 °C. Chromatographic peak width was set to 15 seconds for full scan and HRMS(/MS) analyses in both positive and negative ionisation modes

The second optimised (U)HPLC-HRMS(/MS) method used for untargeted metabolomics analysis of the ‘polar extract’ of DMA samples and the associated SPE fractions, was based on an Accucore Amide HILIC column (2.1 x 100 mm (i.d. x L), 2.6 µm solid core, Thermo Scientific). The column was maintained at 35 °C throughout analyses, with metabolites eluted using a binary linear gradient, supplied at 400 µL/min, with the following profile (time in minutes, %B in parentheses): 0 (0%), 1.5 (0%), 21 (100%), 25 (100%), 26 (0%), 30 (0%). Mobile phase A consisted of 90:5:5% v/v/v acetonitrile:water:buffer, while mobile phase B comprised 50:45:5% v/v/v acetonitrile:water:buffer. The buffer in each mobile phase consisted of 98:2% v/v 100 mM ammonium acetate (aq.):acetic acid. The autosampler capillary was rinsed with 100 µL of 80:20% v/v water:acetonitrile between samples. Full-scan, data-dependent HRMS(/MS), and H-ESI source parameters were as per the Syncronis Phenyl-based method, described above, with the exception that: chromatographic peak width was set to 25 seconds in positive ionisation mode and 15 seconds in negative ionisation mode; the top-5 ions from each full scan underwent HRMS/MS analysis; the apex trigger range was set with a minimum of 6 seconds and maximum of 15 seconds.

All mobile phases used in optimisation and DMA experiments were prepared using solvents and additives of HPLC-grade, or better. Each mobile phase was degassed in an ultrasonic water bath prior to use.

A directed data acquisition methodology was employed in the DMA experimental workflow for the acquisition of data-dependent (U)HPLC-HRMS(/MS) data. Sometimes referred to as Nearline (Neumann et al., 2013), a hybrid of offline and online fragmentation acquisition, the approach constitutes semi-automated acquisition techniques that can be executed with sufficient speed to minimize shifts in data acquisition, thereby achieving greater efficiency compared to offline fragmentation acquisition. See **Supplemental Section 1.8.1.2-1.8.1.3** for full details.

The collected LC fractions then resuspended underwent extensive DI-HRMS(/MS^n^) analysis with a Thermo Fisher Obitrap Elite mass spectrometer.An initial DI-HRMS run being used to determine which features should be prioritised for fragmentation based on available sample volume and time constraints. See **Supplemental Sections 1.8.2** for full details.

#### (U)HPLC-HRMS(/MS) data acquisition sequence

U)HPLC-HRMS(/MS) analysis of each DMA sample extract and SPE fraction followed a defined analysis procedure, as depicted in **Figure S3**. At the start of each sequence, quality assurance samples (‘QA’ samples) consisting of polar or apolar extracts of *D. magna* ‘development samples’ (the former used for Syncronis Phenyl and Accucore amide based methods, the latter used for the Accucore C30 method) were repeatedly injected to equilibrate the (U)HPLC-HRMS(/MS) system, followed by injection of one or two solvent blanks to minimise potential carryover. Full-scan (U)HPLC-HRMS analyses of DMA samples were then commenced, first in positive and then negative ionisation mode, for both the blank and *D. magna* DMA sample type under consideration. Here, data acquisition followed the same pattern in both ionisation modes, with five consecutive injections of the blank DMA sample and five consecutive injections of the corresponding *D. magna* DMA sample, interspaced by two ‘QA’ samples and a solvent blank. A single sample blank was injected between blocks of positive and negative ionisation full-scan acquisitions. Additional solvent blank and ‘QA’ samples were also injected upon completion of full-scan acquisitions, prior to commencing data-dependent acquisition of HRMS/MS data (DDA-HRMS/MS) in both positive and negative ionisation modes. For DDA-HRMS/MS analyses, four consecutive injections of *D. magna* DMA sample were performed in each ionisation mode, with a single solvent blank injection between. To maximise the number of (U)HPLC-HRMS features selected for data-dependent HRMS/MS analysis, the first two and last two sample injections in each set of four DDA-HRMS/MS injections were configured with distinct inclusion and exclusion lists (see **Supplemental Section** **1.8.1.3** for further details).

‘QA’ samples injected throughout the analysis sequence were used to ensure stability of (U)HPLC retention times and HRMS signals (in terms of ppm error and intensity). Solvent blanks, meanwhile, were used both to minimise potential carryover and to allow sufficient time for ‘directed acquisition’ data processing required to generate the inclusion and exclusion lists used in DDA-HRMS/MS acquisitions.

#### (U)HPLC-HRMS(/MS) computational methods used for data acquisition

With a view to focussing on the most informative features to acquire fragmentation on, directed data acquisition was applied here to generate the inclusion and exclusion lists for the DDA-HRMS/MS injections. The approach was developed iteratively throughout the project, culminating in an implementation combining the following software packages: XCMS for feature picking (Smith et al., 2006), CAMERA (Kuhl et al., 2012) to annotate features based on their isotopes and adducts; msPurity (Lawson et al., 2017) for peak processing; the ‘Intervals’ R package for interval scheduling; and the Python package ‘deconrank’ ([github.com/computational-metabolomics/deconrank](https://github.com/computational-metabolomics/deconrank)) for deconvolution of features, ranking and assigning features for fragmentation.

The following key components, along with parameters – albeit with some variations as the workflow evolved throughout the project, were utilised: The raw data files were converted into mzML format using mzconvert (Proteowizard) into centroid configuration. Subsequently, XCMS was then used for feature detection using centWave peak picking algorithm. The parameters were typically set with ppm value of 5, subject to adjustment for specific assays. The signal-to-noise threshold was established at a minimum of 50, with a prefilter setting of (3, 100), integration set to 1, sigma at 3, mzdiff at 0.001, and peakWidth (low) set to 5 and peakWidth (high) between 20 and 40. For the grouping process in XCMS, the parameters included a minfrac between 0.5 and 0.6, mzwid set to 0.025, and bw set to 5. The blank and corresponding *Daphnia* grouped XCMS features were then compared and if a feature was observed in both blank and *Daphnia* samples, the intensity of the *Daphnia* samples was required to be 10 times that of the blank. The features were also filtered for the relative standard deviation of the retention time and for the *Daphnia* samples their intensity as well. A threshold of intensity of (e.g. 5000) was also applied to the *Daphnia* sample features. In addition, to further avoid acquiring fragmentation on blank features the exclusion list also included the top 100 most intense (pre-XCMS) peaks within the scan of each mzML across multiple bins within chromatographic run (e.g. 0-90, 90-660, 660-1230 and 1230-1800).The R package CAMERA employed to annotate features based on their isotopes and adducts, used an error tolerance of 2 ppm. A pre-established adduct ranking, determined by the propensity of adduct formation to fragment (e.g., [M+H]^+^ is ranked higher than [M+Na]^+^), was used to select each feature from within each feature cluster that is expected to yield the most informative fragmentation data for describing that feature cluster. In cases where features are tied, the feature with the highest intensity is selected. Additionally, the features must elute within the same time range as determined by CAMERA's peak cluster groups. This process generates a list of features ranked in order of preference for fragmentation. The full list of ranked adducts are listed within the deconrank package (negative ionisation adducts: [github.com/computational-metabolomics/deconrank/blob/master/deconrank/CAMERA_rules_NegFinal_PlusLi.csv](https://github.com/computational-metabolomics/deconrank/blob/master/deconrank/CAMERA_rules_NegFinal_PlusLi.csv); positive ionisation adducts: [github.com/computational-metabolomics/deconrank/blob/master/deconrank/CAMERA_rules_PosFinal_PlusLi.csv](https://github.com/computational-metabolomics/deconrank/blob/master/deconrank/CAMERA_rules_PosFinal_PlusLi.csv))

The final ranked list of Daphnia features was subsequently organized into two inclusion lists for each assay. Features were iteratively added to these lists, ensuring that the minimum number of overlapping intervals of 10 was not surpassed. Furthermore, a corresponding exclusion list was established, comprising features to be excluded based on the detected blank features from XCMS, the top 100 most intense blank features (per chromatographic bin described above) and any features present on the alternate inclusion list.


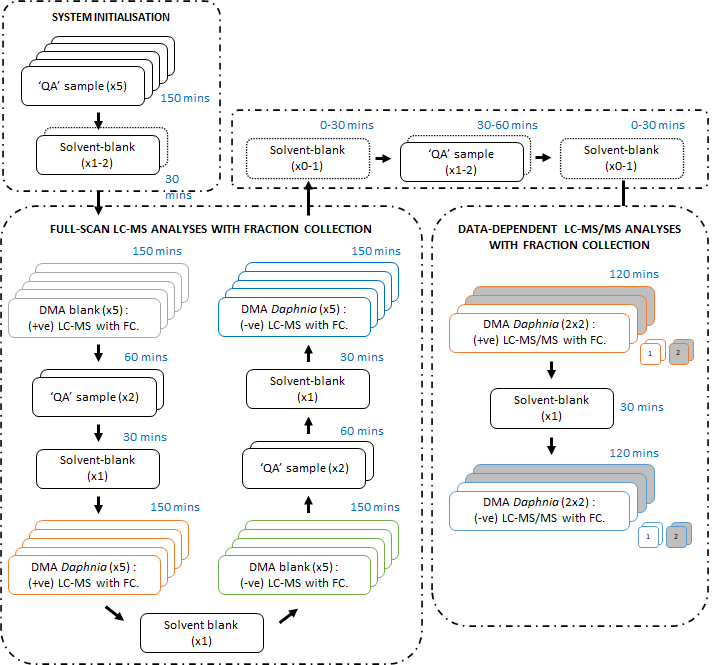


***Figure S3:*** ***Overview of the data acquisition workflow applied for (U)HPLC-HRMS(/MS) analysis and time-based fractionation of DMA samples.*** *“‘QA’ samples” were polar extracts (in the case of the Syncronis Phenyl and Accucore Amide methods) or apolar extractions (in the case of the Accucore C30 method) of D. magna development samples. “(+ve)” and “(-ve)” refer to positive and negative ionisation mode detection, respectively. “FC” means fraction collection. The numbered lists (1 and 2) in the box labelled “Data-dependent LC-MS/MS analysis with fraction collection” representing the inclusion and exclusion lists generated from the directed data acquisition (Nearline) approach.*

### DI-HRMS(/MS^n^)

#### Resuspension of LC fractions for DI-HRMS(/MS^n^) analyses

LC fractions were resuspended prior to DI-HRMS(/MS^n^) analyses. Here, 96-well deep well plates containing paired blank- and *Daphnia*-derived LC fractions (dry), were thawed over wet ice, followed by addition of 100 µL resuspension solution to each well. Resuspension solution was adjusted based on both the ionisation mode of DI-HRMS(/MS^n^) analyses, and the origin of the LC fractions, as follows: 0.25% v/v formic acid in 80:20% v/v methanol:water for analysis of polar LC fractions under positive ionisation conditions; 4:1 v/v methanol:25 mM ammonium acetate (unmodified, aq.) for analysis of polar LC fractions under negative ionisation conditions; 2:1 v/v 7.5 mM ammonium acetate in methanol:chloroform for analysis of apolar LC fractions under positive and negative ionisation conditions. Well plates were then covered with self-adhesive foil and vortex mixed for 15 minutes, before being returned to wet ice. Well contents were subsequently transferred to individual polypropylene PCR tubes (0.5 mL, Sarstedt), from which 15 µL aliquots were in turn transferred to individual wells of a polypropylene 384-well microtiter well plate (Eppendorf twin.tec^TM^) for DI-HRMS(/MS^n^) analyses. Blank-derived LC fractions were each loaded into a single well on the 384-well plate. *Daphnia*-derived LC fractions meanwhile were each loaded into three wells of the 384-well plate (one well for DI-HRMS analysis, one well for DI-HRMS(/MS^n^) analysis, and the third well serving as a backup). The 384-well plate was heat-sealed at 174 °C for 2 s using 20 µm EasyPierce^TM^ foil (Thermo Scientific) and finally loaded into the cooled (10 °C) plate holder of a Triversa NanoMate (Advion) in preparation for analysis. Where immediate analysis was not possible, 384-well plates were stored refrigerated until the first possible opportunity for analysis. PCR tubes were stored refrigerated (+4-5 °C) short-term, before transfer to a laboratory freezer (-20 °C) for longer-term storage of remaining LC fraction aliquots.

#### DI-HRMS(/MS^n^) analytical setup and data acquisition sequence

Resuspended LC fractions underwent DI-HRMS(/MS*^n^*) analyses using a Triversa NanoMate coupled to an LTQ-Orbitrap Elite mass spectrometer (Thermo Fisher Scientific), i.e. nano-electrospray ionisation-based direct infusion-HRMS(/MS*^n^*) analyses. Triversa operational parameters, configured within Chipsoft 8.3.3.1008, were consistent for all scan types: +1.7 kV (positive mode) or -1.7 kV (negative mode) spray voltage; 0.30 psi nitrogen gas pressure; 10 °C interface (plate holder) temperature; 10 µL injection volume; 0.5 µL post-sample air gap; and, with ‘headspace venting’ and ‘air gap before chip’ both enabled. Spray sensing was not used. Infusion flow rate was approximately 300 nL/min. The LTQ-Orbitrap Elite was operated in the profile acquisition mode with 240 °C ion transfer tube temperature and with S-lens RF level set to between 60 and 70%. An automated tuning procedure was applied prior to analyses, using manufacturer recommended calibration mixture. External mass calibration was also performed in accordance with manufacturer recommendations prior to all analyses.

The first step in DI-HRMS(/MS*^n^*) analyses was acquisition of full scan HRMS data for all LC fraction types – both blank- and Daphnia-derived – present on a 384-well plate. For LC fractions generated using the Syncronis Phenyl-based UHPLC-HRMS(/MS) method, full scan data were acquired over 1.5 minutes (inclusive of a 24 second spray stabilisation delay) with the following parameters: Orbitrap detection at 120000 resolution (FWHM_400m/z_), 3e6 AGC target, 50-1000 m/z scan range, 1000 ms maximum ion injection time and single microscan. For fractions generated via HILIC-HRMS(/MS) or C30-HRMS(/MS) methods (i.e. based on HPLC columns ‘AMD’ and ‘C30’, respectively), full scan data were acquired as overlapping Selected Ion Monitoring (SIM) scan windows (while also having an initial 30 s spray stabilisation delay, during which full scan spectra were acquired). Data from SIM scans was subsequently ‘stitched’ together, using a bespoke informatics pipeline, to yield pseudo-full scan spectra (Southam et al., 2017). For HILIC-HRMS(/MS) LC fractions (generated using column ‘AMD’), SIM scan windows were: 50-125, 105-180, 160-235, 215-290, 270-345, 325-400, 380-455, 435-510, 490-565, 545-620, 600-675, 655-730, 710-785, 765-840, 820-895, 875-950, 930-1005 and 985-1060 m/z. For C30-RPLC-HRMS(/MS) LC fractions (generated using column ‘C30’), SIM scan windows were: 190-265, 245-320, 300-375, 355-430, 410-485, 465-540, 520-595, 575-650, 630-705, 685-760, 740-815, 795-870, 850-925, 905-980, 960-1035, 1015-1090, 1070-1145, and 1125-1200 m/z. All SIM scan data were acquired with Orbitrap detection, using a target resolution of 240000 (FWHM_400m/z_) and AGC target of 5e5 (in both positive and negative ionisation modes). All other acquisition parameters were as per those used for DI-HRMS analyses of Syncronis Phenyl-derived LC fractions.

Full scan data, whether collected in full scan or SIM modes, was processed as described in **Supplemental Section** **1.8.2.3**, below. Resulting inclusion lists were used to guide data-dependent tandem (MS^2^) and multiple-stage (MS^3^) mass spectral analyses of *Daphnia*-derived LC fraction components, during subsequent DI-HRMS(/MS^n^) analyses. Herein, full scan data was acquired periodically throughout the infusion of each *Daphnia*-derived LC fraction aliquot, using the following parameters: Orbitrap detection at 120000 resolution (FWHM_400 m/z_) and 3e6 AGC target for LC fractions generated during Syncronis Phenyl-based RPLC-HRMS(/MS) analyses, and at 240000 resolution (FWHM_400 m/z_) with 5e5 AGC target for LC fractions generated from both HILIC-HRMS(/MS) and C30-RPLC-HRMS(/MS) analyses; scan range 50-1000 m/z, 50-1060 m/z and 190-1200 m/z for LC fractions generated during Syncronis Phenyl-based RPLC-HRMS(/MS), HILIC-HRMS(/MS), and C30-RPLC-HRMS(/MS) analyses, respectively; S-lens RF level between 60-70%; maximum ion injection time of 1000 ms; single microscan. For LC fractions generated during HILIC-HRMS(/MS) and C30-RPLC-HRMS(/MS) analyses, a data-dependent SIM scan followed each full scan (to better evaluate precursor ion purity), with the following parameters: Orbitrap detection at 120000 resolution (FWHM_400 m/z_), 5e5 AGC target, S-lens RF level between 60-70%; 10 microscans. Precursor ions selected from full scan events then underwent data-dependent (i.e. guided by an inclusion list) HCD-based fragmentation at three distinct normalised collision energy (NCE) values, specifically 20, 40 and 80%, using 0.1 ms activation time and with 50 m/z fixed lower scan-range limit. CID-based MS^n^ analyses (effected using the linear ion trap) were also attempted for the same precursor ions, with fragmentation energy set to 35% NCE and with 30 ms activation time and 0.25 activation Q. Up to the top-3 most intense CID-induced MS^2^ product ions, with m/z values < 98% of the original precursor ion, were targeted for CID-based MS^3^ analyses. All MS^2^ and MS^3^ scan types were performed in triplicate, using: 1 Da isolation window; 2e5 AGC target; Orbitrap detection at 120000 resolution (FWHM_400 m/z_); 1000 ms maximum ion injection time; dynamic exclusion enabled; minimum signal requirement of either 5000 counts (for LC fractions generated using column ‘PHE’) or 500 counts (for LC fractions generated using methods ‘AMD’ and ‘C30’); default charge state of 1. Dynamic exclusion settings were: repeat- and exclusion-time equal to the duration of DI-HRMS(/MS^n^) method section, maximum exclusion list size of 500 and exclusion width of < ±2.5 ppm. See **Figure S4** for a summary of the data acquisition workflow.

#### DI-HRMS(/MS*^n^*) computational methods used for data acquisition

The same principle used for the directed data acquisition (i.e. Nearline (Neumann et al., 2013)) for (U)HPLC-HRMS(/MS) workflow was also applied to DI-HRMS(/MS^n^) analysis to prioritise the most informative features to acquire fragmentation data on. The workflow was developed iteratively over the course of the project with a final implementation using the Python package ‘DIMSpy’ ([github.com/computational-metabolomics/dimspy](https://github.com/computational-metabolomics/dimspy)) for the peak processing or “feature picking”; adducts and isotope peaks putatively assigned using a modified version of the R package CAMERA ([github.com/computational-metabolomics/cameraDIMS](https://github.com/computational-metabolomics/cameraDIMS) ) (Kuhl et al., 2012); and the Python package ‘deconrank’ ([github.com/computational-metabolomics/deconrank](https://github.com/computational-metabolomics/deconrank)) used to rank and select features for fragmentation, as well as to schedule the chosen features into inclusion lists. Visual scripting was then used to automatically create mass spectrometry instrument methods based on the inclusion lists generated.

As mentioned, the approach was iteratively improved as the project was developed but bellow describes broadly the workflow and parameters used for all assays:

Full-scan HRMS data generated during DI-HRMS analysis of resuspended LC fractions, was extracted in centroid format from associated .RAW data. For data acquired using SIM-type scan windows (i.e. LC fractions generated via HILIC-HRMS/MS and C30-RPLC-HRMS/MS methods), SIM spectra were stitched together to generate pseudo full scan spectra. Resulting peak matrices, each containing the m/z value, intensity and signal-to-noise ratio (as defined by manufacturer algorithm) values for all peaks in all full scan spectra (whether generated using full scan or SIM scan acquisition modes) of a given file, were then filtered. In each matrix, peaks with SNR ≥ 3 underwent hierarchical clustering using typically 5 ppm overlap threshold applied to peak m/z value (exact ppm used dependent on the dataset analysed). Replicate and %RSD filters were then applied followed by an absolute-intensity ≥ 5000. Extract-blank and corresponding *Daphnia* peak matrices were subsequently compared. Peaks present in the both matrices, as defined by a 5 ppm overlap window, were removed from the latter if their intensity was less than 10-times that in the former, yielding a ‘blank filtered’ matrix. The msPurity R package was then used to calculate the anticipated precursor ion purity of the feature (defined as precursor ion intensity / total intensity of all ions within +/- 0.5 m/z units).

To each of these matrices was applied the adapted version of the CAMERA package (compatible with DI-MS datasets), facilitating ion-type and isotope annotations. Each feature could thus have no annotation, an adduct annotation, an isotope annotation, or both. From each resulting feature cluster, the feature expected to yield the most informative fragmentation spectrum was selected based on pre‑established adduct ranking rules (e.g. M+H⁺ ranked above M+Na⁺); in cases of ties, the most intense feature was selected. These selected features were designated as “first‑tier features,” with all other cluster members classified as “second‑tier features.”. All features (both first and second tiers) are then scored by peak intensity, full-scan anticipated precursor ion purity, ion-type annotation and number of other associated ions sharing the same candidate neutral molecular mass. Weightings factors summed to a total of 1 and were set as follows: ion type 0.3, intensity 0.3, purity 0.2 and adducts in group 0.2. A subset of the second-tier features (e.g. 50%) being included on the inclusion list but with a penalised overall score (i.e. given a lower priority for fragmentation). Additionally, hard thresholds were used to remove features where the anticipated precursor ion purity was very low (e.g. <0.05) and removal of features where they strongly thought to be isotopes (e.g. [M+1]+ [M+2]+).

Xcalibur (v3.0.63) method files for all DI-HRMS(/MS^n^) analyses were generated using an automated visual-scripting methodology to automate Windows graphical user interface (GUI) operations (using the python package pywinauto). All method files comprised two distinct sections, the first dedicated to HCD-based HRMS/MS data acquisition, and the second to ion-trap-facilitated CID MS^3^ data acquisition. The time duration of each section, and therefore the overall method duration (inclusive of a 30 s spray stabilisation delay period), were adapted based on the length of the inclusion list supplied and according to defined limits to keep each acquisition run within approximately 30 minutes. Within these prescribed limits, section 1 duration (i.e. HCD-based HRMS/MS acquisition) was set in accordance with: length of inclusion list multiplied by 10 s (duration required for triplicate analysis at three %NCE values). Duration of section 2 was set in accordance with: length of inclusion list multiplied by 0.33 (i.e. 33% of peaks considered) multiplied by 12 s (duration required for triplicate analysis at single NCE value via ion-trap).


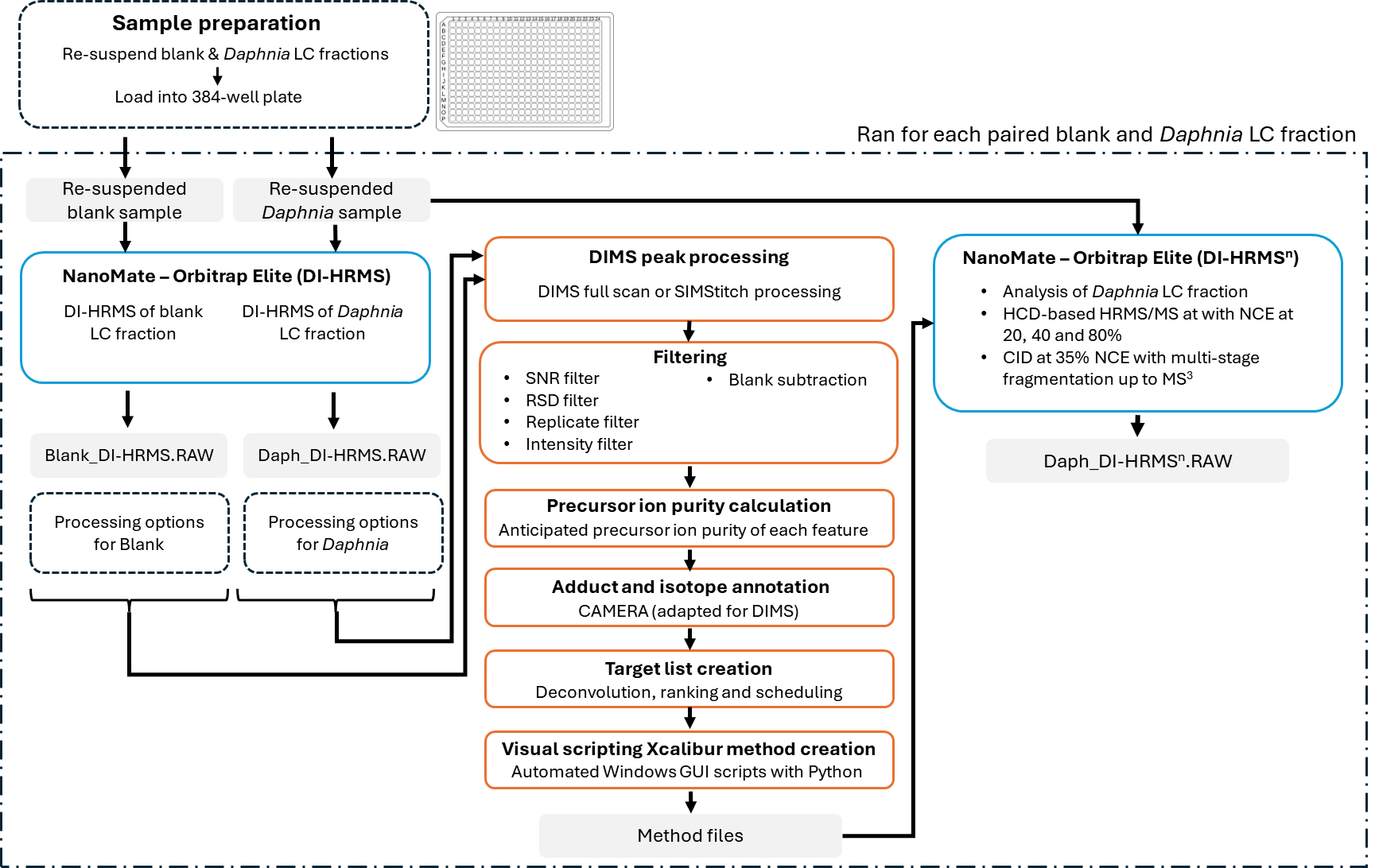


***Figure S4:*** ***Overview of the data acquisition workflow applied for DI-HRMS(/MS^n^) analysis of the DMA re-suspended LC fractionation samples.*** *Demonstrating how the re-suspended LC fractionation samples were analysed by DI-HRMS(/MS^n^). First DI-HRMS (Orbitrap Elite) analysis is performed on the paired re-suspended blank and Daphnia LC fractions to generate Thermo MS RAW files for each sample. These files are then peak processed with the resulting peak matrices filtered. Then the precursor ion purity is calculated and adduct and isotopes are annotated for the filtered features. A target list of features is then created prioritising features that will be the most informative to obtain fragmentation spectra on within restricted time limits for the acquisition. The target list of features is then used as input to generate Xcalibur methods files (the generation of which was automated using visual scripting with Python). The method files are then used as input for the analysis of the corresponding Daphnia LC fraction for both HCD-based HRMS/MS and CID-based multi-stage fragmentation.*

## (U)HPLC-HRMS(/MS) method optimisation

Two (U)HPLC-HRMS(/MS) methods were optimized for analysis and (U)HPLC-based fractionation of DMA *D. magna* ‘polar extracts’ and associated SPE fractions. The first, a reversed-phase liquid chromatography (RPLC) method, was based on a Syncronis Phenyl column (2.1 x 100 mm, 1.7 µm; Thermo Scientific). The second, a hydrophilic interaction liquid chromatography (HILIC) method, was established using an Accucore Amide column (2.1 x 100 mm, 2.6 µm; Thermo Scientific).

(U)HPLC-HRMS(/MS) method optimisation sought to maximise the number and chromatographic distribution of ‘reproducibly detectable metabolic features’ (**RDMFs**) recorded in WAX and WCX SPE fractions, derived from polar extracts of *Daphnia magna* “development samples” (see **Supplemental Section 1.5**), under both positive and negative ionisation conditions. RDMFs were defined as any (U)HPLC-HRMS feature that, across technical replicate injections, had percent relative standard deviation (%RSD) ≤ 15% with respect to intensity and ≤ 3% with respect to retention time, and that were not annotated as potential isotopes, adducts or background ions during data processing, i.e. after background filtering and spectral deconvolution. In so doing, the goal was to maximise the number of metabolites for which HRMS/MS data could be collected during (U)HPLC-HRMS/MS analysis of DMA samples, whilst also ensuring that (semi-)purified metabolites could be reproducibly and concurrently fractionated (i.e. collected into well plates) for downstream, in-depth direct infusion-HRMS*^n^* analyses as part of the DMA analytical workflow (see **Supplemental Section 2.1** for details).

To rationalise the number of optimisation experiments performed, SPE fractions were combined based on presumed overlap in the physicochemical properties of their constituent metabolites. Fractions WAX[1], WAX[2], WCX[3] and WCX[4] were combined to generate sample type ‘WAX12WCX34’, whose constituents were expected to be polar-to-moderately polar, including some with (inducible) cationic structural moieties (e.g. amines). Fractions WAX[3], WAX[4], WCX[1] and WCX[2], meanwhile, were combined to yield “WCX12WAX34” containing polar-to-moderately polar metabolites, some of which having anionic structural moieties (e.g. carboxy groups, sulfates etc). *Daphnia*-derived SPE fraction pools are hereafter referred to as ‘DAX12DCX34’ and ‘DCX12DAX34’, while corresponding extraction blank SPE fractions are termed ‘BAX12BCX34’ and ‘BCX12BAX34’.

Each phase of method optimisation used SPE fractions prepared from distinct sets of *D. magna* “development samples”. Hence, RDMF counts were comparable within an optimisation experiment, wherein a single set of “development samples” were used, but not necessarily between optimisation experiments where different sets may have been used.

**Table S6**: Liquid chromatography systems used for (U)HPLC-HRMS(/MS) method optimisation

| **System identifier^(2)(3)^** | **Component** | **Additional details** |
| --- | --- | --- |
| RS-LCsys1 | Ultimate^TM^ SRD-3600 | In-line degasser |
|  | Ultimate^TM^ HPG-3400RS | High-pressure binary gradient pump |
|  | Ultimate^TM^ TCC-3000RS | Thermostatted column compartment |
|  | Ultimate^TM^ WPS-3000TRS | Temperature controlled and fitted with 100 µL stainless steel split loop and 6-port high pressure switch valve |
| RS-LCsys2 | Ultimate^TM^ SR-3000 | In-line degasser |
|  | Ultimate^TM^ LPG-3400RS | Quaternary pump |
|  | Ultimate^TM^ TCC-3000RS | Thermostatted column compartment |
|  | Ultimate^TM^ WPS-3000TRS | Temperature controlled autosampler unit fitted with a 25 µL syringe, 25 µL stainless steel split loop and 6-port high pressure switch valve |
| FC-LCsys^(1)^ | Ultimate^TM^ SRD-3600 | Six-channel in-line degasser |
|  | Ultimate^TM^ DGP-3600RS | Dual-gradient pump unit with ternary solvent-mixing capabilities |
|  | Ultimate^TM^ FLM-3100 | Thermostatted flow control manager (column oven) |
|  | Ultimate^TM^ WPS-3000TFC-ANALYTICAL | Temperature-controlled fraction collection-enabled autosampler fitted with 20 µL nanoViper pulled-loop, 2.4 µL needle, 6.4 µL bridge tubing, 25 µL syringe and 50 µL buffer tubing (all constructed from PEEK) |

^(1)^ Injections on ‘FC-LCsys’ were performed using a custom-written ‘User Defined Program’, in which a 10 µL sample plug was injected at the centre of a loading solvent plug of volume 10 µL, with composition typically matching mobile-phase start conditions[MJ1] .

^(2)^ All LC systems were configured, from injection loop through to mass spectral detector, with Viper (stainless-steel) or nanoViper (PEEK) capillaries.

^(3)^ All autosampler modules, irrespective of analytical method applied, were operated at 10 °C, with samples loaded in clear-glass 300 µL fixed-insert autosampler vials, sealed using pre-assembled threaded caps with PTFE-silicone septa[MJ2]

**Table S7**: Mass spectrometer operational parameters for optimisation of (U)HPLC-HRMS(/MS) methods

|  | **Mass spectrometer scan parameters** | | | | | | **MS source parameters** | | | | | | | | | | |
| --- | --- | --- | --- | --- | --- | --- | --- | --- | --- | --- | --- | --- | --- | --- | --- | --- | --- |
| **Method ID** | **System** | **Scan range (m/z)** | **Full-scan** | | **MS/MS** | | **Gas (AU)** | | | **Aux. gas temp. (°C)** | **Spray voltage (kV)** | **S-lens level (%)** | **Ion transfer tube temp. (°C)** | **Position** | | |  |
|  |  |  | **Resolut-ion*** | **AGC** | **Resolut-ion** | **AGC** | **Sheath** | **Aux.** | **Sweep** |  |  |  |  | **x-axis** | **y-axis** | **z-axis** |  |
| Ph-CO-1 | Q Exactive | 100-1000 | 70000 | 3e6 | NA | NA | 40 | 15 | 0 | 300 | Pos: +3.5,  Neg: -3 | 70 | 320 | +0.5 | C | 1.5 |  |
| Ph-CO-2 |  |  |  |  |  |  |  |  |  |  |  |  |  |  |  |  |  |
| Ph-CO-3 |  |  |  |  |  |  |  |  |  |  |  |  |  |  |  |  |  |
| Ph-CO-4 |  |  |  |  |  |  |  |  |  |  |  |  |  |  |  |  |  |
| Ph-FO-1 |  |  |  |  |  |  |  |  |  |  |  |  |  |  |  |  |  |
| Ph-FO-2 |  |  |  |  |  |  |  |  |  |  |  |  |  |  |  |  |  |
| Ph-FO-3 |  |  |  |  |  |  |  |  |  |  |  |  |  |  |  |  |  |
| AA-CO-1 | Q Exactive | 100-1000 | 70000 | 3e6 | NA | NA | 40 | 15 | 0 | 200 | Pos: +3.5, Neg: -3 | 70 | 320 | +0.5 | C | 1.25 |  |
| AA-CO-2 |  |  |  |  |  |  |  |  |  |  |  |  |  |  |  |  |  |
| AA-CO-3 |  |  |  |  |  |  |  |  |  |  |  |  |  |  |  |  |  |
| AA-FO-1* |  |  |  |  |  |  |  |  |  | 300 |  |  |  |  |  | 1.5 |  |
| AA-FO-1** | Q Exactive Focus |  |  |  |  |  | Pos: 40, Neg: 20 | Pos: 15, Neg: 5 |  | Pos: 300, Neg: 200 | Pos: +3.5,   Neg: -4 |  |  |  |  | 1.75 |  |
| AA-FO-2 |  |  |  |  |  |  |  |  |  |  |  |  |  |  |  |  |  |
| AA-FO-3 |  |  |  |  |  |  |  |  |  |  |  |  |  |  |  |  |  |
| AA-FO-4 |  |  |  |  |  |  |  |  |  |  |  |  |  |  |  |  |  |
| AA-FO-5 |  |  |  |  |  |  |  |  |  |  |  |  |  |  |  |  |  |
| AA-FO-6 |  |  |  |  |  |  |  |  |  |  |  |  |  |  |  |  |  |
| C30 | Q Exactive | 200-1200 | 70000 | 1e6 | 35000 | 1e5 | 40 | 15 | 1 | 250 | Pos: +3,   Neg: -3 | 45 | 285 | +0.5 | C | 1.75 |  |

| * parameters used for phases 2 and 3 of HILIC-HRMS(/MS) method optimisation |
| --- |
| ** parameters used for phase 4 of HILIC-HRMS(/MS) method and for DMA experiments. |

**Table S8**: Liquid chromatography operational parameters for optimisation of (U)HPLC-HRMS(/MS) methods

| **Column** | **Method ID** | **A-phase** | **B-phase** | **Gradient (%B / time / flow rate)** | **Column temp. (°C)** | **Syringe wash** | **Inj. vol. (µL)** | **LC system** | **AS temp. (°C)** |
| --- | --- | --- | --- | --- | --- | --- | --- | --- | --- |
| Syncronis Phenyl | Ph-CO-1 | 0.1% v/v FA in H2O | 0.1% v/v FA in MeOH | 5/ 5/ 95/ 95/ 5/ 5% B at 0/ 1.5/ 22/ 25/ 26/ 30 min at 0.4 mL/min | 40 | 80:20% v/v MeOH:H_2_O | 10 | RS-LCsys1 | 10 |
|  | Ph-CO-2 | 0.1% v/v FA in H2O | 0.1% v/v FA in ACN |  |  |  |  |  |  |
|  | Ph-CO-3 | 95:5% v/v H2O:100 mM AmAc buffer (aq., pH 5.80) - final pH 5.8 | 5:95% v/v 100 mM AmAc buffer (aq., pH 5.80):MeOH |  |  |  |  |  |  |
|  | Ph-CO-4 | 95:5% v/v H2O:100 mM AmAc buffer (aq., pH 5.80) - final pH 5.8 | 5:95% v/v 100 mM AmAc buffer (aq., pH 5.80):ACN |  |  |  |  |  |  |
|  | Ph-FO-1 | 90:5:5% v/v/v H2O:MeOH:100 mM AmAc buffer (aq., pH 5.80) | 5:95% v/v 100 mM AmAc buffer (aq., pH 5.80):MeOH | 0/ 0/ 100/ 100/ 0/ 0% B at 0/ 1.5/ 22/ 25/ 26/ 30 min at 0.4 mL/min |  |  | 10* | FC-LCsys |  |
|  | Ph-FO-2 |  |  | 0 /0 / 70/ 100/ 100/ 0/ 0% B at 0 / 1.5/ 22/ 22.1/ 25/ 26/ 30 min at 0.4 mL/min |  |  |  |  |  |
|  | Ph-FO-3 |  |  | 0/ 0/ 100/ 100/ 100/ 0/ 0/ 0% B at 0/ 1.5/ 22/ 22.1/ 25/ 26/ 29.9/ 30 min at 0.3/ 0.3/ 0.3/ 0.4/ 0.4/ 0.4/ 0.4/0.3 mL/min |  |  |  |  |  |
| Accucore amide | AA-CO-1 | 90:10% v/v ACN:50 mM AmFm buffer (aq., pH 3.00) | 40:50:10% v/v/v ACN:H_2_O:50 mM AmFm buffer (aq., pH 3.00) | 0/ 0/ 100/ 100/ 100/ 100/ 100/ 0/ 0/ 0% B at 0/ 1.5/ 21/ 23/ 23.01/ 25/ 25.01/ 26/ 30/ 30.1 min with 0.2/ 0.2/ 0.2/ 0.2/ 0.3/ 0.3/ 0.4/ 0.4/ 0.4/ 0.2 mL/min | 30 | 80:20% v/v H2O:MeCN | 10 | RS-LCsys1 | 10 |
|  | AA-CO-2 | 90:10% v/v ACN:50 mM 50 mM AmAc buffer (aq., pH 6.63) | 40:50:10% v/v/v ACN:H2O:50 mM AmAc buffer (aq., pH 6.63) |  |  |  |  |  |  |
|  | AA-CO-3 | 90:10% v/v ACN:50 mM 50 mM AmAc buffer (aq., pH 5.80) | 40:50:10% v/v/v ACN:H2O:50 mM AmAc buffer (aq., pH 5.80) |  |  |  |  |  |  |
|  | AA-FO-1 | 90:5:5% v/v ACN:H2O:100 mM AmAc buffer (aq., pH 6.63) | 40:55:5% v/v/v ACN:H2O:100 mM AmAc buffer (aq., pH unmodified at approximately 6.9) | 0/ 0/ 100/ 100/ 100/ 100/ 100/ 0/ 0/ 0 / 0% B at 0/ 1.5/ 21/ 23/ 23.01/ 25/ 25.01/ 26/ 30/ 30.1/ 35 min with 0.2/ 0.2/ 0.2/ 0.2/ 0.3/ 0.3/ 0.4/ 0.4/ 0.4/ 0.2/ 0.2 mL/min |  |  | 10* | FC-Lcsys and RS-Lcsys2 |  |
|  | AA-FO-2 | 90:5:5% v/v/v ACN:H2O:100 mM AmAc (unmodified) | 40:55:5% v/v/v ACN:H2O:100 mM AmAc (aq., pH unmodified at approximately 6.9) | 0/ 0/ 100/ 100/ 0/ 0% B at 0/ 1.5/ 21/ 25/ 26/ 30 min at 0.4 mL/min |  |  | 5 | RS-Lcsys2 |  |
|  | AA-FO-3 |  | 60:35:5% v/v/v ACN:H2O:100 mM AmAC (aq., pH unmodified at approximately 6.9) |  |  |  |  |  |  |
|  | AA-FO-4 | 90:5:5% v/v/v ACN:H2O:100 mM AmAc (pH 5.8) | 40:55:5% v/v/v ACN:H2O:100 mM AmAc (pH 5.8) |  |  |  |  |  |  |
|  | AA-FO-5 |  | 60:35:5% v/v/v ACN:H2O:100 mM AmAc (pH 5.8) |  |  |  |  |  |  |
|  | AA-FO-6 | 90:5:5% v/v/v ACN:H2O:100 mM AmAc + 2% HOAc | 60:35:5% v/v/v ACN:H2O:100 mM AmAc + 2% HOAc |  |  |  |  |  |  |
| Accucore C_30_ | C30-Eval | 10 mM AmFm in 50% ACN + 0.1% FA | 2 mM AmFm in ACN:IPA:H2O 10:88:2% (v/v/v) + 0.02% FA | 22/60/85/100/100/22/22% B at 0/6/14/23/26/26.1/30 minutes at 0.4 mL/min | 35 | 47.5:47.5:5% v/v/v ACN:IPA:H2O | 10* | FC-LCsys | 10 |

* 20 µL injection in which 10 µL of sample is injected between a loading solvent plug (mobile phase A).

### Sample preparation for (U)HPLC-HRMS(/MS) method optimisation

SPE fraction pools used for (U)HPLC-HRMS/MS method optimisation were resuspended to a total volume of 100 µL. The resuspension solution used was equivalent in composition to the (U)HPLC gradient start conditions, unless otherwise specified. In practice, all SPE fractions to be combined were thawed and maintained on wet-ice throughout the resuspension process (see **Figure S5**, below). Thereafter, 100 µL of ice-cold resuspension solution was added into the microfuge tube containing the SPE fraction with lowest elution order number within a given pool, e.g. DAX[1] in ‘DAX12DCX34’ and DCX[1] in ‘DCX12DCX34’. The tube was then vortex mixed for 30 seconds, before centrifugation at 21885 x *g* and 4 °C for 5 mins. The resulting supernatant was entirely transferred to the next highest-numbered fraction, again followed by vortex mixing, centrifugation and transfer – a procedure repeated until reaching the final fraction within a pooling group. Pooled SPE fraction samples, i.e. WAX12WCX34 and WCX12WAX34, were finally centrifuged for 10 minutes at 4 °C and 21885 x *g*, before transfer to 300 µL clear-glass autosampler vials for analysis.


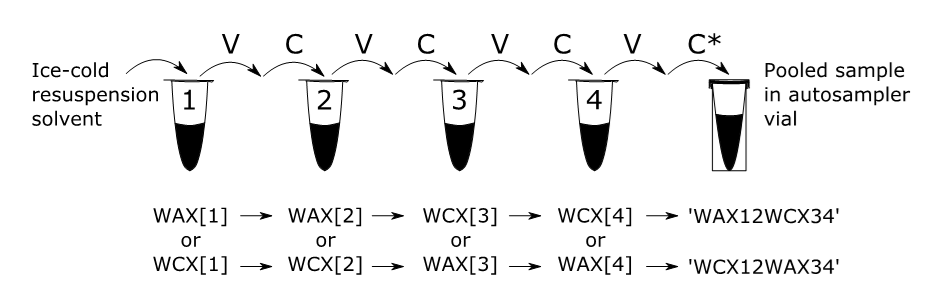


***Figure S5: Sample preparation for (U)HPLC-HRMS(/MS) method optimisation. V: vortex mix, C: centrifuge, C*: extended centrifugation period, prior to loading in to autosampler vial.***

Quality assurance (QA) samples were prepared by resuspending thawed polar extracts of *D. magna* development samples (see **Supplemental Section 1.5**, above) in 100 µL of ice-cold resuspension solution. QA samples were vortex mixed for 30 s and then centrifuged for 10 min at 21885 x g and 4 °C. The resulting supernatant was transferred to a glass autosampler vial for analysis. Extraction blank ‘QA’ samples were likewise prepared. *D. magna* QA samples were injected repeatedly at the start of each (U)HPLC-HRMS(/MS) analysis sequence to initialise the LC-MS system, and then intermittently throughout each sequence to monitor system performance. Extract blank QA samples were likewise injected, generally towards the start and end of each sequence, to monitor the extent of LC-MS background signal.

### Optimisation of a Syncronis Phenyl-based reserved phase UHPLC-HRMS(/MS) method for untargeted analysis of DMA ‘polar’ extracts and associated SPE fractions

#### Phase one

Phase one of method optimisation, termed “coarse optimisation” (CO), for the Syncronis Phenyl-based RPLC method, involved exploration of the impact of different organic solvent types and mobile phase modifiers on the number and chromatographic distribution of RDMFs. Four methods were trialled. Methods ‘Ph-CO-1’ and ‘Ph-CO-2’ used acetonitrile as organic solvent, while methods ‘Ph-CO-3’ and ‘Ph-CO-4’ used methanol. Methods ‘Ph-CO-1’ and ‘Ph-CO-4’ included 0.1% v/v formic acid as mobile phase modifier, whereas methods ‘Ph-CO-2’ and ‘Ph-CO-3’ included 5 mM ammonium acetate buffer (5 mM final concentration, aqueous buffer pH 5.8) in each mobile phase. All other chromatography and mass spectrometry settings were kept constant across methods, as summarised in **Tables S7** and **S8**.

Samples DAX12DCX34 and DCX12DAX34 were analysed by consecutive quadruplicate injection in positive ionisation mode, and by consecutive triplicate injection in negative ionisation mode. Between each sample type, a single injection of corresponding blank SPE fraction pool was performed, followed by an injection of a *D. magna* ‘QA’ sample.

#### Phase two

Phase two of optimising the Syncronis Phenyl-based RPLC method involved fine-tuning of the eluent gradient shape and flow rate of the best-performing method from the first phase of optimisation, method ‘Ph-CO-3’. Herein, the primary objective was to maximise the chromatographic spread of RDMFs across the available chromatographic elution time to maximise the purity of DMA LC fractions while minimising HRMS(/MS) spectral complexity.

Method ‘Ph-CO-3’ was updated to use a lower initial percent of mobile phase B: 0/0/100/100/0/0% B at 0/1.5/22/25/26/30 min, using 400 µL/min flow rate throughout. This updated method was termed ‘Ph-FO-1’. From this reference method, two additional methodological modifications were explored, the first (‘Ph-FO-2’) utilising a two-step-binary gradient elution profile (0/0/70/100/100/0/0% B at 0/1.5/22/22.1/25/26/30 min) at the original 400 µL/min flow rate, and the second (‘Ph-FO-3’) implementing a reduced mobile phase flow rate of 300 µL/min throughout the gradient ramp period (minutes 0 to 22), before returning to 400 µL/min for the remainder of the elution period.

For each method, quintuplicate injections were performed of both DAX12DCX34 and DCX12DAX34 sample types. Corresponding blank SPE fraction pools were injected in triplicate after their respective *Daphnia* equivalents, followed by a single or duplicate injection of a *D. magna* ‘QA’ sample. This analysis order was applied first in positive and then in negative ionisation modes.

### Optimisation of an Accucore Amide-based hydrophilic interaction liquid chromatography (HILIC)-HRMS(/MS) method for untargeted analysis of DMA ‘polar’ extracts and associated SPE fractions

#### Phase one

Phase one of optimising an Accucore Amide-based HILIC-HRMS(/MS) method for DMA involved exploration of the impact, on RDMF counts and distributions, of altering the pH and additive type of the aqueous buffer used to prepare the binary-gradient mobile phases. As summarised in **Table S8**, method ‘AA-CO-1’ used a 50 mM ammonium formate buffer (aq.) adjusted to pH 3.0 through dropwise addition of 50 mM ammonium formate into 50 mM formic acid (aq.). Method ‘AA-CO-2’ used a 50 mM ammonium acetate solution (aq.), pH unmodified (approximately pH 6.6). Method ‘AA-CO-3’, meanwhile, used a 50 mM ammonium acetate buffer (aq.) adjusted to pH 5.8 using 50 mM acetic acid (aq.). In all methods, mobile phase ‘A’ comprised 90:10% v/v acetonitrile:aqueous buffer, while mobile phase ‘B’ comprised 40:50:10% v/v/v acetonitrile:water:aqueous buffer.

Owing to limited solubility of dried SPE fractions in 100% mobile phase A, a modified version of the SPE-fraction resuspension procedure described in **Supplemental Section 1.9.1** (above) was applied. At each stage of the resuspension process (i.e. for each SPE fraction included in an SPE fraction pool), the following steps were applied: transfer resuspension solution into the microfuge tube containing the dried SPE fraction; vortex mix for 60 seconds; sonicate twice for 10 seconds; vortex mix for a further 30 seconds; sonicate for 30 seconds; vortex mix for a 30 seconds and; finally, centrifuge for 5 minutes at 21885 x g and 4 °C. This procedure is hereafter referred to as ‘AA-Resus-1’. All sonication steps were performed using an ultrasonic bath operated at ambient temperature. Total resuspension volume was 100 µL.

Samples DAX12DCX34 and DCX12DAX34 were each analysed in quadruplicate, in both positive and negative ionisation modes, followed by corresponding extract blank samples. One or two *D. magna* ‘QA’ samples were injected after the latter.

#### Phase two

Phase two of Accucore Amide-based HILIC-HRMS(/MS) method optimisation sought to evaluate the impact of sample resuspension on the number and diversity of RDMFs. Hence, three SPE fraction resuspension strategies were trialled. The first, “AA-Resus-1”, followed the procedure outlined in phase one of HILIC-HRMS(/MS) method optimisation (**Supplemental Section 1.9.3.1**, above) – this served as the reference method. The second, “AA-Resus-2”, replicated the procedure for “AA-Resus-1” but used a pre-mixed solution of 80:20% v/v acetonitrile:dimethyl sulfoxide (DMSO) for resuspension. The final resuspension method evaluated, ‘AA-Resus-3’, used two solutions for resuspension, the first being 50 µL ice-cold 100 mM ammonium acetate (pH unmodified) and the second being 50 µL ice-cold acetonitrile. For both, the resuspension procedure followed that of “AA-Resus-1”, with the final supernatants combined. For all resuspension methods evaluated, the resuspended sample was vortex mixed, centrifuged (4 °C for 10 minutes at 21885 x g) and transferred into an autosampler vial, prior to analysis. Resulting SPE fraction pools were analysed using system ‘LC-FCsys’, (see TableS6, above), in combination with a Q Exactive mass spectrometer. Chromatographic conditions matched those for method ‘AA-CO-3’, save for an additional 5-minute hold at 100% mobile phase A that was added to the end of the method for re-equilibration purposes – hereafter termed method ‘AA-FO-1’. Mobile phase ‘A’ was used as loading solvent for the plugged sample injection procedure.

All SPE fraction pools prepared using a given resuspension protocol were analysed consecutively, in both positive and then negative ionisation modes. DAX12DCX34 and DCX12DAX34 fraction pools were injected in triplicate, each followed by duplicate injection of corresponding BAX12BCX34 and BCX12BAX34 samples. *D. magna* QA samples were injected singly, or in duplicate, following each set of blank SPE fraction pool injections, to monitor and equilibrate the (U)HPLC-HRMS(/MS) system.

#### Phase three

Phase three of optimizing an Accucore Amide-based HILIC method for DMA involved further evaluation of the impact of the SPE fraction resuspension procedure on RDMF counts and distributions. Method “AA-Resus-4” was based on “AA-Resus-3” but used 20 µL of 100 mM ammonium acetate buffer (aq., pH unmodified) for the first part of resuspension, and 80 µL acetonitrile for the second. Following each vortex mixing step depicted in **Figure S5**, above, the SPE fraction undergoing resuspension was centrifuged for 5 minutes at 21885 x *g* and 4 °C, prior to transfer of the resulting supernatant into the next tube. The final supernatants generated using both 20 µL 100 mM ammonium acetate buffer and 80 µL acetonitrile, were combined into a single tube. An additional 20 µL methanol was spiked into this tube to avoid phase separation, followed by vortex mixing, centrifugation (5 minutes at 21885 x g and 4 °C) and transfer of the supernatant into a glass autosampler vial.

For comparison, additional SPE fraction pool samples were prepared as per “AA-Resus-1” (see **Supplemental Section** **1.9.3.2**, above) – the leading resuspension method from prior optimisation experiments.

Both DAX12DCX34 and DCX12DAX34 were each analysed in quadruplicate, followed by corresponding extract blanks (BAX12BCX34 and BCX12BAX34, respectively), in both positive and negative ionisation modes using methods ‘AA-FO-1’ (see **Supplemental Section 1.9.3.2**).

#### Phase four

Phase four of optimizing an Accucore Amide-based HILIC-HRMS(/MS) method for DMA involved re-evaluating the method’s chromatographic conditions. This was undertaken due to substantial changes implemented for sample resuspension during earlier phases of method optimisation, which were anticipated to impact both the type and concentration of metabolites accessible through this method. A total of six chromatographic conditions were therefore trialled, as outlined in **Table S7** and **Table S8**, using system ‘LC-RSsys1’ (see **Table S6**) in combination with a Q Exactive Focus mass spectrometer. Method ‘AA-FO-1’ (see **Supplemental** **Section 1.9.3.2**) was transferred to this system with a 10 µL injection volume defined (no longer using a plugged solvent injection procedure). Methods ‘AA-FO-2’ through ‘AA-FO-6’ all used a 5 µL injection volume. For methods ‘AA-FO-1’ and ‘AA-FO-2’, injection volume (10 versus 5 µL) and flow rate (200 versus 400 µL min^-1^) were the sole technical differences between the methods. Method ‘AA-FO-3’ replicated ‘AA-FO-2’, except for a reduction in the aqueous content of mobile phase B (strong mobile phase) to 40% v/v. Methods ‘AA-FO-4’ and ‘AA-FO-5’ were replicas of methods ‘AA-FO-2’ and ‘AA-FO-3’, respectively, albeit with the aqueous buffer used for mobile phase preparation having been adjusted to pH 5.8 using 100 mM acetic acid. Method ‘AA-FO-6’ was likewise a replica of method ‘AA-FO-2’, albeit with the aqueous buffer used to prepare mobile phases comprising 100 mM ammonium acetate spiked with acetic acid to 2% v/v. Methods ‘AA-FO-[3-6]’ all also used a higher linear flow rate of 400 µL/min.

Across all methods, SPE fraction re-suspension followed closely the two-step procedure described for ‘AA-Resus-4’ (see **Supplemental Section 1.9.3.3**, above), differing only in that 24 µL of aqueous buffer and 96 µL of acetonitrile were used, followed by addition of 10 µL methanol for biphase disruption. Owing to failure to dissipate the resulting biphase in sample vials for methods ‘AA-FO-4 ‘, ‘AA-FO-5’ and ‘AA-FO-6’, it was necessary to spike in an additional 10 µL methanol into the SPE fraction pools.

### Data processing for (U)HPLC-HRMS(/MS) method optimisation

Data files from (U)HPLC-HRMS(/MS) method optimisation were converted to .mzML format using msconvert (Proteowizard). XCMS was used to read and extract data from .mzML files and to perform feature detection using the centWave peak picking algorithm with the following parameters: ppm = 5, signal to noise threshold = 100, prefilter = (3, 100), sigma = 3, mzdiff = 0.001, peakWidth (low) = 5 and peakWidth (high) = 20. XCMS’s group.density function was then used to group metabolic features within and across sample groups, using the following parameters: ‘bw’ = 5, ‘minfrac’ = 0.6, ‘minsamp’ = 1, ‘mzwid’ = 0.025, ‘max’ = 50. Adduct and isotope annotations were assigned to features in the resulting data matrix using the CAMERA package, with the following functions and parameters applied: function ‘groupFWHM’ used ‘perfwhm’ = 0.6, and ‘sigma’ = 6; function ‘groupCorr’ used ‘cor_eic_th’ = 0.75, ‘calcIso’ = 0, ‘calcCiS’ = 0, ‘calcCaS’ = 0, ‘graphMethod’ = hcs, and ‘pval’ = 0.05; function ‘findIsotopes’ used ‘intval’ = maxo, ‘maxcharge’ = 3, ‘minfrac’ = 0.5, ‘maxiso’ = 4, ‘mzabs’ = 0.01, and ‘ppm’ = 5; function ‘findAdducts’ used ‘max_peaks’ = 100, ‘multiplier’ = 3, ‘mzabs’ = 0.015, and ‘ppm’ = 5. Spectral deconvolution was subsequently performed within Python as per **Supplemental Section 1.8.1.2.**

## GC-EI-HRMS

Aliquots of the ‘polar extract’ and associated SPE fractions derived from DMA experimental samples, were analysed by gas chromatography-electron ionisation-high resolution mass spectrometry (GC-EI-HRMS). Prior to analysis, each sample underwent a two-step methoximation-silylation derivatization procedure, as follows: 1) add 50 µL of 20 mg/mL O-methoxyamine hydrochloride in pyridine (99.8%) to sample; 2) vortex mix for 30 seconds; 3) heat sample to 80 °C and maintain for 15 minutes; 4) bring sample to room temperature; 5) add 50 µL of N-methyl-N-trimethylsilyltrifluoroacetamide (MSTFA) containing 1% trimethylchlorosilane (TMCS); 6) vortex mix for 20 seconds; 7) heat sample to 80 °C and maintain for 15 minutes; 8) bring sample to room temperature; 9) spike 5 µL of n-alkane retention index solution comprising 0.6 mg/mL of each of decane, dodecane, pentadecane, nonadecane, docosane, pentacosane and nonacosane, in pyridine; 10) vortex mix for 20 seconds; 11) centrifuge at 15800 x *g* and room temperature for 15 minutes; 12) transfer 50 µL of supernatant to 300 µL fixed-insert, amber-glass GC automsampler vial and crimp seal using pre-assembled caps with PTFE septum. All heating was performed using a Reacti-Therm I heat block (Thermo Scientific). All solutions were handled with positive displacement pipettes fitted with disposable glass, microdispenser capillaries. Between transfer procedures, pipettes were rinsed with *n*-hexane and wiped with lint-free tissue paper to minimise cross contamination.

Aliquots of crude polar extracts of *D. magna* ‘development samples’ were prepared according to the above procedure and used for GC-EI-HRMS system equilibration and as intra-study QC samples.

GC-EI-HRMS analyses were performed using a Thermo Scientific TriPlus RSH autosampler coupled, via a wool-lined heated injection port (280 °C; 1:100 split ratio), to a Thermo Scientific TRACE 1310 gas chromatograph and, in turn, to a Q Exactive GC-Orbitrap mass spectrometer, via an Extractabrite electron ionisation/chemical ionisation source. Samples were injected at a volume of 1 µL and separated over a TG-5SilMS capillary column (30 m length x 0.25 mm internal diameter x 0.25 µm film thickness; fitted with 15 cm integrated, non-functionalised guard; selectivity comparable to 5% diphenyl/95% dimethylpolysiloxane columns) using helium as carrier gas (1 mL/min) and with the following temperature gradient elution program: 0/4/12.5/20.5/30 min at 70/70/320/320/70 °C. Column eluates were passed through an MS transfer line (250 °C) in to a heated electron-ionization (EI) source (230 °C), wherein ionisation was induced through interaction with an electron ‘beam’ operated at 70 eV. Positive-mode, full-scan (50-650 m/z) mass spectral data acquisition was performed at 60000 resolution (FWHM 200 m/z) and with AGC target of 1e6, using a dedicated GC-Orbitrap mass spectrometer. Data were not acquired during the first five minutes of elution. Lock-masses of 207.0324 m/z, 281.0511 m/z and 355.0699 m/z were included for internal calibration purposes. The mass spectral system was externally mass-calibrated < 24 hours prior to data acquisition.

Xcalibur software was used to construct and thereafter automate the GC-EI-HRMS analysis sequence. Prior to analysis of DMA SPE fractions, five QA sample injections were performed for the purpose of system stabilisation. Thereafter, DMA SPE fractions were analysed at random, with QA samples injected every fourth sample for system monitoring purposes.

GC-EI-HRMS data were processed using TraceFinder v.4.1(beta), including peak-picking, deconvolution and spectral library lookup. Processing parameters were set as follows: accurate mass tolerance of 5 ppm; signal to noise ratio threshold of 10; minimum and maximum retention times bounds were 5.3 and 20.0 min, respectively; TIC intensity threshold of 100000; ion overlap window of 98%; ‘use all ions’ enabled; retention alignment window of 5 seconds and; library search type set to ‘normal’ with ‘golmdb’ selected.

Peak annotations were made through matching of full-scan mass spectra to the NIST 2014 HRAM and GOLMdb libraries, as well as to an internal GC-EI-HRMS library constructed under the same temperature programme as described above, using authentic chemical standards. Annotations did not take into consideration the retention time, nor indices, of the extracted mass spectral features due to software limitations. Quantifier ions were automatically selected via the TraceFinder software.

Annotations were filtered to retain only those with dot-product match scores > 700 (equivalent to the 0.7 dot product cosine threshold used elsewhere in this paper). The threshold has been previously reported as a general guide for considering an acceptable match, where 700-800 is considered a fair match, 800-900 a good match and greater than 900 considered a very good match (Garreta-Lara et al., 2016).

## 1D- & 2D-NMR

SPE fractions derived from DMA polar metabolite extracts were analysed by 1D- & 2D-NMR spectroscopy. *Daphnia* SPE fractions, with corresponding blanks, were thawed over wet ice for 10 minutes before addition of 60 μL of 0.1 M phosphate buffer (pH 7.0, 9:1% v/v HPLC-grade water:deuterium oxide) containing 0.5 mM trimethylsilylpropanoic acid (chemical shift calibrant). Tubes were vortex mixed for 30 seconds, followed by centrifugation at 15,000 rpm and 4 °C, for 10 minutes. For SPE fraction WCX[3], it was necessary to agitate the sample for 60 s in an ultrasonic bath to promote dissolution of the pelleted material, followed by centrifugation. Thereafter, 50 μL of each supernatant was transferred to an individual, clear-glass champagne vial (1.5 mL, Cronus) and stored capped (PTFE-silicone-PTFE, Cronus) in a laboratory refrigerator (5 ± 1 °C) before robot-assisted transfer into 1.7 mm NMR tubes (1.7 x 103.5 mm; SampleJet). Finally, samples were loaded and stored in a cooled (6 °C) NMR SampleJet autosampler for analysis. All NMR analyses were performed at the Biomolecular NMR facility Birmingham, United Kingdom, using a Bruker AVANCE III 600 MHz NMR spectrometer equipped with a 1.7 mm TCI-Cryoprobe (1H, 13C, 19F) and operated at a proton frequency of 600.13 MHz.

A combination of 1D and 2D-NMR experiments were used to study SPE fractions. Each fraction was initially profiled by 1D proton nuclear overhauser effect NMR spectroscopy (1D-^1^H-NOESY). The 1D-^1^H-NOESY pulse sequence (Bruker noesygppr1d pulse sequence) included water suppression and consisted of the following parameters: mixing time 10 ms, spectral width 11.98 ppm, number of points 32k, relaxation delay 4 s and number of scan 128. Total acquisition time per experiment amounted to 14 minutes.

To support metabolite annotation, 2D homonuclear ^1^H-^1^H (2D-JRes and TOCSY) and heteronuclear ^1^H-^13^C (HSQC) experiments were performed. For 2D ^1^H-^1^H J-Resolved (J-Res) NMR experiments, data were acquired using 8 transients for 32 increments, each collected with 16k data points and with a spectral width of 12 ppm in the direct dimension F2 (chemical shift axis) and 50 Hz in the indirect dimension F1 (spin-spin coupling axis) for a total acquisition time of 24 minutes. 2D 1H-1H TOCSY spectra were acquired using a DIPSI-2 (Shaka et al., 1988) mixing sequence with the following parameters: 140 ms acquisition time, 65 ms mixing time, 11.98 ppm spectral width and relaxation delay 2s, number of scans 16 and 512 increments corresponding to a total acquisition time of 2 hours and 34 minutes. 2D 1H-13C Heteronuclear Single Quantum Coherence spectroscopy (HSQC) experiments (Davis et al., 1991) were carried out using a 65 ms acquisition time with GARP 13C decoupling and a 1.5 s relaxation delay. A total of 64 transients were averaged for each of the 256 increments resulting in an acquisition time of 3 hours and 35 minutes.

To shorten the acquisition time of total correlation spectroscopy (TOCSY) and HSQC experiments, a Non-Uniform Sampling (NUS) algorithm (Multi-dimensional Decomposition (Orekhov and Jaravine, 2011)) was applied. Here, only a subset of data points were acquired in a semi-randomised manner. For both experiment types, the amount of sparse sampling was set to 40%.

Automated tuning, matching and shimming was performed prior to data acquisition. Temperature gradients and radiation damping occasionally necessitated manual shimming. Such were the difficulties in achieving an effective shim on the aforementioned NMR system, that polar-arm SPE fractions for the metabolite reference standard mixture were analysed using a Bruker AVANCE III HD 600 MHz IVDr validated NMR spectrometer, fitted with a room temperature double resonance broadband probe (Bruker 5mm BBI).

NMR data analysis was performed using the TopSpin software suite (v.3.5pl2, Bruker BioSpin Germany). For assignment of identities to spectral signals, data were compared to various NMR databases (SpinCouple (Kikuchi et al., 2016)), the Human Metabolome Database (Wishart et al., 2007), the Birmingham Metabolomics Library (Ludwig et al., 2012) and Biological Magnetic Resonance Data Bank (Hoch et al., 2022) and to the metabolite standard mixture components. For putatively annotated compounds, further experiments were performed involving spiking of reference compounds into solution.

## DMA computational workflow overview

The DMA workflow predominantly consists of the Galaxy workflow, however annotations are also incorporated from external sources (i.e. mzCloud, GNPS workflows, NMR and GC-MS). See **Figure S6** for overview for the full overview of the computational workflow.


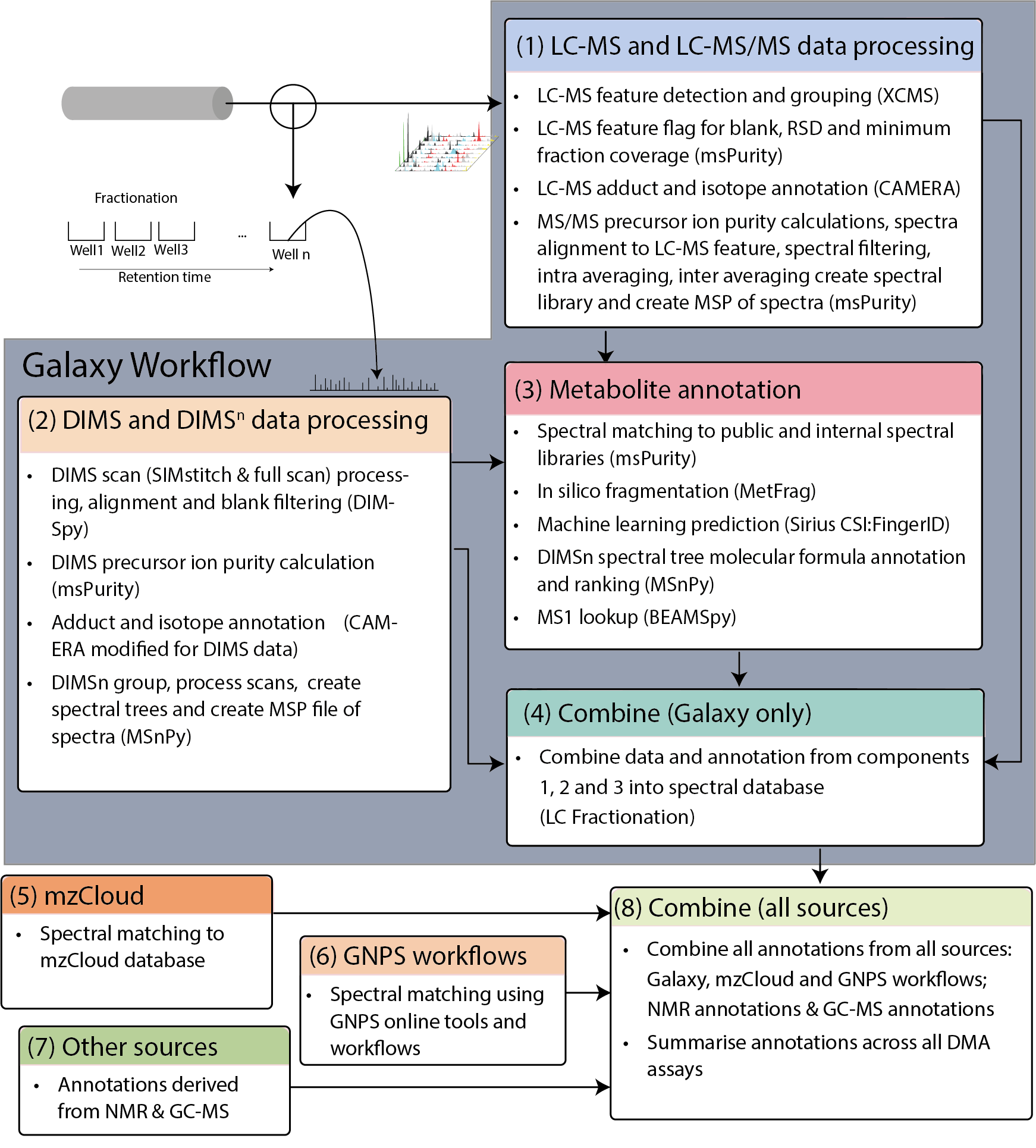


***Figure S6: Overview of computational analysis of DMA (U)HPLC-HRMS(/MS) and DI-HRMS(/MS^n^) LC fractionation experiments****. Conceptual schematic of the data processing, metabolite annotation, data management and additional data analysis techniques and approaches used.*

## DMA Galaxy workflow

### Overview

The “DMA Galaxy Workflow” is split into 5 components: “Data input”, “(U)HPLC-HRMS(/MS) data processing”, “DI-HRMS(/MS^n^) data processing”, “Metabolite annotation” and the “Combining” component.

The “Data input” workflow component consists of either the RAW mass spectrometry files, the equivalent mzML file or the sample metadata for each file.

The **“(U)HPLC-HRMS(/MS) data processing”** workflow component uses the Workflow4Metabolomics XCMS Galaxy tools (Giacomoni et al., 2015; Smith et al., 2006) for chromatographic feature picking and grouping of (U)HPLC-HRMS features between multiple files. Galaxy wrappers of the msPurity R package are also used to flag and calculate quality metrics for MS1 features (i.e. flag blank features, calculate RSD of the intensities and retention time of the MS1 data), calculate precursor ion purity of the fragmentation spectra, align fragmentation spectra to the XCMS MS1 features, filter fragmentation data based on signal to noise, average fragmentation spectra within files (intra-averaging), average fragmentation spectra across files (inter-averaging), combine the (U)HPLC-HRMS(/MS) based analysis into a single SQLite database and create MSP files of the averaged fragmentation spectra. Additionally the CAMERA Galaxy tool (Giacomoni et al., 2015; Kuhl et al., 2012) is used as means to determine adducts and isotopes.

The **“DI-HRMS(/MS^n^) data processing”** workflow component uses the Galaxy wrappers for the DIMSpy tools ([github.com/computational-metabolomics/dimspy-galaxy](https://github.com/computational-metabolomics/dimspy-galaxy)) to process the DI-HRMS data - including averaging and grouping spectra within and across files and blank subtraction. Galaxy wrappers of the MSnPy tools are also used to process the fragmentation component of the DI-HRMS(/MS^n^) data – including grouping related scans, processing and averaging the m/z and intensities from each scan group, creating spectral and fragmentation trees of each group and creating MSP files of the averaged fragmentation spectra. Additionally, the msPurity Galaxy tools are used to calculate the precursor ion purity and CAMERA DIMS Galaxy tool is used as means to determine adducts and isotopes.

The **“Metabolite annotation”** workflow components use the Galaxy wrappers of msPurity for spectral matching; the Galaxy wrapper for SIRIUS-CSI:FingerID (Dührkop et al., 2019) for predicting structure based on fragmentation trees and a machine learning model derived from public mass spectrometry datasets; and the Galaxy wrapper for MetFrag (Ruttkies et al., 2019, 2016; Wolf et al., 2010) that annotates features using *in silico* prediction of mass spectral fragmentation patterns. Annotation of the MS1 features is also performed using the Galaxy wrapper for BEAMSpy - using a neutral mass lookup of each feature to a compound database (HMDB) based on a neutral mass calculated from a predefined list of adducts.

The **“Combining”** workflow component uses the msPurity combineAnnotation tools and the LC Fractionation Galaxy tool to combine all the annotations and processed data into a single SQLite database. The (U)HPLC-HRMS(/MS) data is first combined using the msPurity combineAnnotation tool, where all annotations from the (U)HPLC-HRMS(/MS) dataset (i.e. spectral matching, MetFrag, SIRIUS CSI:FingerID and MS1 neutral mass lookup) are aligned based on their InChiKeys. Scores from each approach are weighted based on predetermined weights and the annotations are ranked for each annotated feature. The “LC Fractionation” Galaxy tool is then used when fractionation data is available and combines all the DI-HRMS(/MS^n^) data and annotations in the same way as the combineAnnotation but additionally aligns features observed in the DI-HRMS(/MS^n^) fractions to the (U)HPLC-HRMS features.

Galaxy workflows and workflow histories available at <https://dma.galaxy.bham.ac.uk/>. Full parameters for each tool used can be found in the histories and key parameters are also detailed in the individual sections of the methods here.

### Galaxy tool summary

**Table S9:** Summary of Galaxy tools

| **Tool Name** | **Underlying software of tool** | **Fig 3. DMA Galaxy Work-**  **flow step(s)** | **Description of tool** |
| --- | --- | --- | --- |
| **LC-MS and LC-MS/MS data processing** | | | |
| MSnBase.readMSData | MSnBase R Package | 2 | Read in mzML files using the MSnBase R package to be used for XCMS processing |
| xcms.findChromPeaks | XCMS R package | 3 | Data processing tool for feature detection of mass spectrometry datasets |
| xcms.findChromPeaks Merger | XCMS R package | 4 | Data processing tool to merge multiple xcms.xcmsSet data objects into a single object |
| xcms.groupChromPeaks | XCMS R package | 5 | Data processing tool for grouping mass spectrometry peaks between different files |
| CAMERA  .Annotate | CAMERA R package | 7 | Annotation tool for adducts and isotopes of LC-MS data |
| **msPurity.purityA | msPurity R package | 1 | Assess the precursor ion purity for fragmentation spectra acquired from either a LC-MS/MS or DI-MS(/MS*^n^*) experiment |
| **msPurity.flagRemove | msPurity R package | 6 | Tool to flag XCMS grouped peaks from the xcmsSet object based on various thresholds (e.g. RSD of intensity and retention time). The peaks can then be removed from the xcmsSet object and regrouped |
| **msPurity.frag4feature | msPurity R package | 8 | Assign fragmentation spectra (MS/MS) stored within a purityA class object to grouped features within an XCMS xset object. |
| **msPurity  .filterFragSpectra | msPurity R package | 9 | Flag and filter features based on signal-to-noise ratio, relative abundance, intensity threshold and precursor ion purity of precursor. |
| **msPurity  .averageFragSpectra | msPurity R package | 10, 11 | Average and filter fragmentation spectra for each XCMS feature using an msPurity purityA object. The tool uses the msPurity functions averageAllFragSpectra, averageIntraFragSpectra and averageInterFragSpectra. |
| **msPurity.  createMSP | msPurity R package | 12 | This tool will extract the MS/MS spectra data from an msPurity object into a MSP file |
| **msPurity.  createDatabase | msPurity R package | 14 | Create SQLite database of LC-MS/MS dataset |
| **DI-HRMS(/MS^n^) data processing** | | | |
| dimspy  .Process scans | dimspy python Package | 22,23 | Process Scans (and SIM-Stitch) - Read, filter and average MS scans |
| dimspy  .merge peaklists | dimspy python package | 24 | Merge peaklists produced by the tools 'Process scans (and SIM-Stitch)' or 'Replicate filter' |
| dimspy  .align samples | dimspy python package | 25 | Align Samples - Align peaks across Peaklists |
| dimspy.  blank filter | dimspy python package | 26 | Blank Filter - Remove 'blank' peaks from the biological mass spectra |
| dimspy.  Get peaklist | dimspy python package | 27 | Export a tsv file of the peaklist |
| *CAMERA DIMS | CAMERA R package (modified to handle DIMS data) | 29 | Modification of the R package CAMERA to work on DI-MS data |
| **msPurity  .dimsPredictPurity(single) | msPurity R package | 28 | Calculate the anticipated precursor ion purity from a DI-MS dataset |
| **MSnPy  Group Scans | MSnPy python package | 30 | Group fragmentation events from DI-MS(/MS^n^) data |
| **MSnPy  Process Scans | MSnPy python package | 31 | Read, filter and average DI-MS(/MS^n^) scans |
| **MSnPy  Create Spectral Trees | MSnPy python package | 32 | Create spectral trees from processed scan data derived from DI-MS(/MS^n^) data |
| **MSnPy  Convert Spectral Trees | MSnPy python package | 33 | Convert spectral trees to MSP files or dimspy PeakList objects |
| **Metabolite annotation** | | | |
| BEAMSpy | BEAMSpy python package | 20, 40 | Birmingham mEtabolite Annotation for Mass SpectroMetry - for MS1 metabolite annotations and spectral annotations (e.g. adducts and isotopes) |
| **msPurity  .spectralMatching | msPurity R package | 15, 39 | Perform spectral matching to spectral libraries for an LC-MS/MS dataset. |
| *SIRIUS CSI:FingerID | SIRIUS CSI:FingerID | 16, 37 | Run the metabolite annotation software SIRIUS CSI:FingerID on MSP files |
| *MetFrag | MetFrag | 18, 38 | Run the metabolite annotation software MetFrag on MSP files |
| **MSnPy  Annotate Spectral Trees | MSnPy python package | 35 | Annotate and/or filter spectral trees derived from DI-MS(/MS^n^) data |
| **MSnPy  Rank Spectral Trees | MSnPy python package | 36 | Rank annotated spectral trees derived from DI-MS(/MS^n^) data |
| **Combining** | | | |
| **msPurity.combineAnnotations | msPurity R package | 21 | Combine, score and rank metabolite annotation results |
| **LC fractionation processor | lcfrac python package | 41 | Combine and process spectra and metabolite annotation results from an LC-MS(/MS) & DI-MS(/MS^n^) fractionation experiment |
| **Miscellaneous** | | | |
| **msp_split | Python script | 13 | Split an MSP file into *n* number of files |
| **msp2db | msp2db python package | 34 | create an SQLite database from MSP files |
| **msPurity.purityX | msPurity R package | ***NA | Calculate the anticipated precursor ion purity from a LC-MS XCMS dataset |
| **deconrank | Deconrank python package | ***NA | Deconvolute adducts and isotopes then score and rank for fragmentation |

**Footnotes**: *New Galaxy tool developed by authors. **New underlying software and Galaxy tool developed by authors. ***Not used directly as part of the metabolite annotation workflow shown in **Figure 3** of the main paper. The abbreviations of liquid chromatography mass spectrometry (LC-MS), liquid chromatography tandem mass spectrometry (LC-MS/MS), direction infusion mass spectrometry (DI-MS) and direct infusion mass spectrometry with multi stage fragmentation (DI-MS(/MS^n^)) are used here when describing approaches that do not require ultra-high performance liquid chromatography (UHPLC) or high resolution mass spectrometry (HRMS).

### (U)HPLC-HRMS(/MS) data processing

#### MSnBase and XCMS

(U)HPLC-HRMS(/MS) mzML files were first processed with MSnBase.readMSData Galaxy tool that creates MSnBase R datatypes that are compatible with the subsequent XCMS tools.

The XCMS Galaxy tool xcms.findChromPeaks is then used to perform (U)HPLC-HRMS feature detection, the CentWave algorithm was used and the following parameters were used - ppm: 11.5, peakwidth: “3, 30”, mzdiff: -0.00375 and snrthres: 10.

The Galaxy tool xcms.findChromPeaks is ran as a data collection where each MSnBase.readMSData processed mzML file is ran independently to one another. The resulting xcms.findChromPeaks outputs are then merged into a single object and appropriate sample metadata applied using the xcms.findChromPeaks merger Galaxy tool.

The Galaxy tool xcms.groupChromPeaks was then used to group the chromatographic features across each of the files using the PeakDensity approach was used with the following parameters - bw: 0.25, minFraction: 0.5, minSamples: 1 and binSize: 0.0157.

The parameters for xcms.findChromPeaks and xcms.groupChromPeaks determined by running IPO (Libiseller et al., 2015) optimisation of the XCMS parameters on a subset the (U)HPLC-HRMS data files for the majority of the individual assays and taking the medium parameters for each value.

#### msPurity ((U)HPLC-HRMS(/MS))

(U)HPLC-HRMS(/MS) data processing was done primarily through the Galaxy tools of msPurity that has been substantially updated since the original publication.

Within the DMA galaxy workflow, using the Galaxy tool msPurity.purityA the precursor ion purity was calculated for all the fragmentation spectra from the (U)HPLC-HRMS(/MS) mzML files (linear interpolation to derive the precursor ion purity score with, features less than 5% of the peak were removed and C13 isotope peaks were disregarded).

The Galaxy tool msPurity.frag4feature was then used to map the fragmentation spectra to the XCMS chromatographic features using a tolerance of 10 ppm to match between the fragmentation spectra precursor and XCMS features.

The Galaxy tool msPurity.filterFragSpectra was then used to calculate signal-to-noise ratio of all fragmentation spectra. It should be noted though that no features were removed at this stage as further filtering was performed based on averaged spectra in the next step in the workflow.

The Galaxy tool msPurity.averageFragSpectra was then used to average the fragmentation spectra associated with XCMS chromatographic features. The tool was run twice, first to average within each file (intra-averaging) and then again to average across files (inter-averaging). The averaging was performed on the *m/z* values using hierarchical clustering with a cutoff of 5 ppm where the averaged fragment peak consists of the median intensity and median *m/z* value of the contributing peaks that are averaged. Fragment peaks were removed that were not present in at least 50% of the scans (for intra-averaging) or at least 50% of files (for inter averaging). See **Figure S7** for schematic of data processing and averaging strategy.


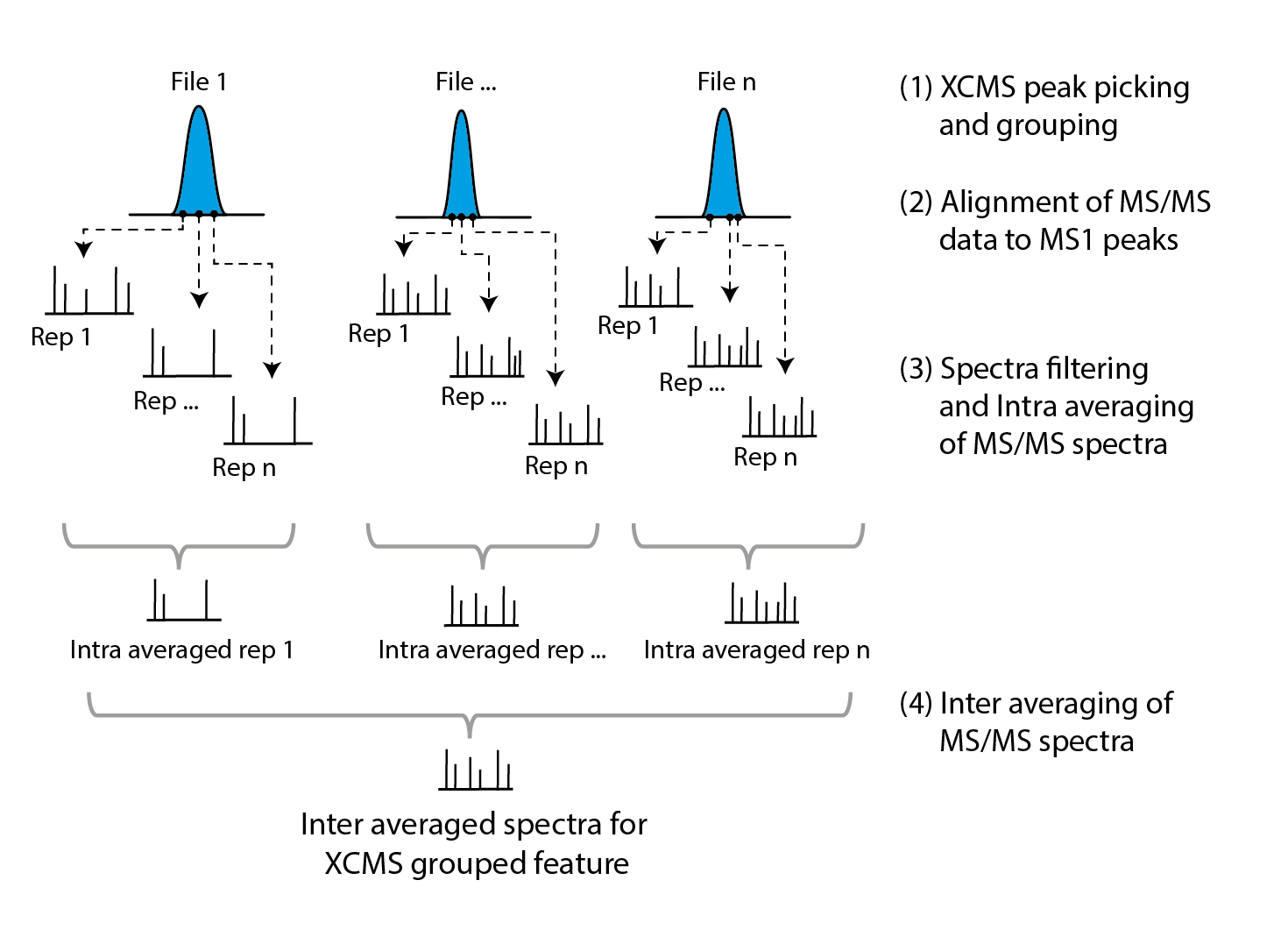


***Figure S7: (U)HPLC-HRMS(/MS) data processing schematic for msPurity and XCMS.*** *Schematic showing how (U)HPLC-HRMS(/MS) data within a file is averaged (intra-averaging) and across a file is averaged (inter-averaging) using msPurity and XCMS.*

The Galaxy tool msPurity.createMSP was then used to create MSP files for the averaged spectra that in turn is used with the MetFrag and SIRIUS CSI:FingerID Galaxy tools, The Galaxy tool msPurity.createDatabase tool was used to create an SQLite database of the (U)HPLC-HRMS(/MS) dataset (schema - <https://bioconductor.org/packages/release/bioc/vignettes/msPurity/inst/doc/msPurity-spectral-database-vignette.html>) - the SQLite database is used as input for the (U)HPLC-HRMS(/MS) spectral matching and used to map the DI-HRMS(/MS^n^) data to the (U)HPLC-HRMS(/MS) data.

Additionally, the Galaxy tool ‘msPurity.flagRemove’ was used to calculate the RSD of the intensity and RSD of the retention time for each feature the blank and non-blank (i.e. “samples”) files, flags any features that are not observed in less than a defined minimum fraction of features and flag features that are determined as blanks. We not here that no features were removed at this stage as we intended to obtain all annotations regardless of the feature quality. Instead, blank filtering was performed after the Galaxy workflow at the final combining stage (see section S1.14)

Both the Galaxy tools of msPurity and the underlying R package are developed and maintained by the authors.

#### CAMERA

The Galaxy tool CAMERA.Annotate was used to annotate isotopes and adducts in the LC-MS/MS data using a threshold of 5 ppm and absolute *m/z* error of 0.015, and using the correlation inside samples for peak grouping.

### DI-HRMS(/MS^n^) data processing

#### DIMSpy

DI-HRMS processing was done via the Galaxy tools of the DIMSpy Python package. For each assay, the workflow processed each of the DI-HRMS raw files generated from every well of the fractionation experiment.

The DIMSpy.Process Scan tool was used to process the scans and perform hierarchical clustering to average the spectra across scans (i.e. the same technique that was used for averaging the fragmentation spectra). The DIMSpy.Process tool was setup to either process SIMstitch DI-HRMS data or full scan DI-HRMS(/MS^n^) data. Each processing and averaging is performed on each raw file independently and then merged into a single object using the DIMSpy.Merge peaklist tool. A signal to noise threshold of 3 (based on the ThermoFisher noise value extracted from the .RAW file).

We note that the *Daphnia* samples and blank samples were processed using slightly different settings, with the *Daphnia* samples used a 5 ppm tolerance to average across scans and the blank samples used a 2 ppm tolerance. The lower ppm used for blank samples was used as an attempt to provide more robust blank subtraction, the difference however is thought to have minimal overall impact and in any future use of the workflow using the same ppm tolerance between *Daphnia* samples and blank samples would be suggested.

The DIMSpy.Align Samples Galaxy tool is then used to align the blank and *Daphnia* samples together based on a ppm tolerance of 5 ppm, and then blank subtraction of peaks can be performed using the DIMSpy.Blank filter Galaxy tool, again based on a ppm tolerance of 5 and a *Daphnia* feature required to be 10 times the intensity of the blank sample feature.

The Galaxy tool DIMSpy.Get peaklists was used to create a data collection of the processed peaklist as text files and a data collection of the peaklists as hdf5.

Both the Galaxy tools of DIMSpy and the underlying Python package are developed and maintained by the authors.

#### MSnPy

DI-HRMS(/MS^n^) processing was conducted via several Galaxy tools that cover the functionalities of the MSnPy python package. For each assay, the workflow processed each of the DI-HRMS(/MS^n^) raw files generated from every well of the analytical fractionation experiment.

The first stage is to group scans that are replicates of one another and map the relationship between scans (e.g. replicates of different collision energies, types and MS levels) using the MSnPy.Group scans Galaxy tool. Next the Galaxy tool MSnPy.Process-scans is used to average the features within each scan group (using the same hierarchical clustering approach as used in DIMSpy and msPurity for averaging) with a ppm tolerance of 5. The averaged scans are then used to create spectral tree networks using the Galaxy tool MSnPy.create-spectral-trees. The averaged fragmentation data of these trees are then exported using the “MSnPy.convert-spectral-trees” tool. This provides outputs of the averaged spectra in multiple configurations: merged fragmentation spectra across all collision energies and MS levels; merged fragmentation spectra but keeping each energy collision energy independent and MS level independent; and finally just the precursors that were targeted for the fragmentation. The outputs are available in hdf5 and MSP formats. See **Figure S8** for a schematic of the data processing and averaging strategy used with MSnPy.

Both the Galaxy tools of MSnPy and the underlying Python packages for DI-HRMS(/MS^n^) data processing are developed and maintained by the authors.


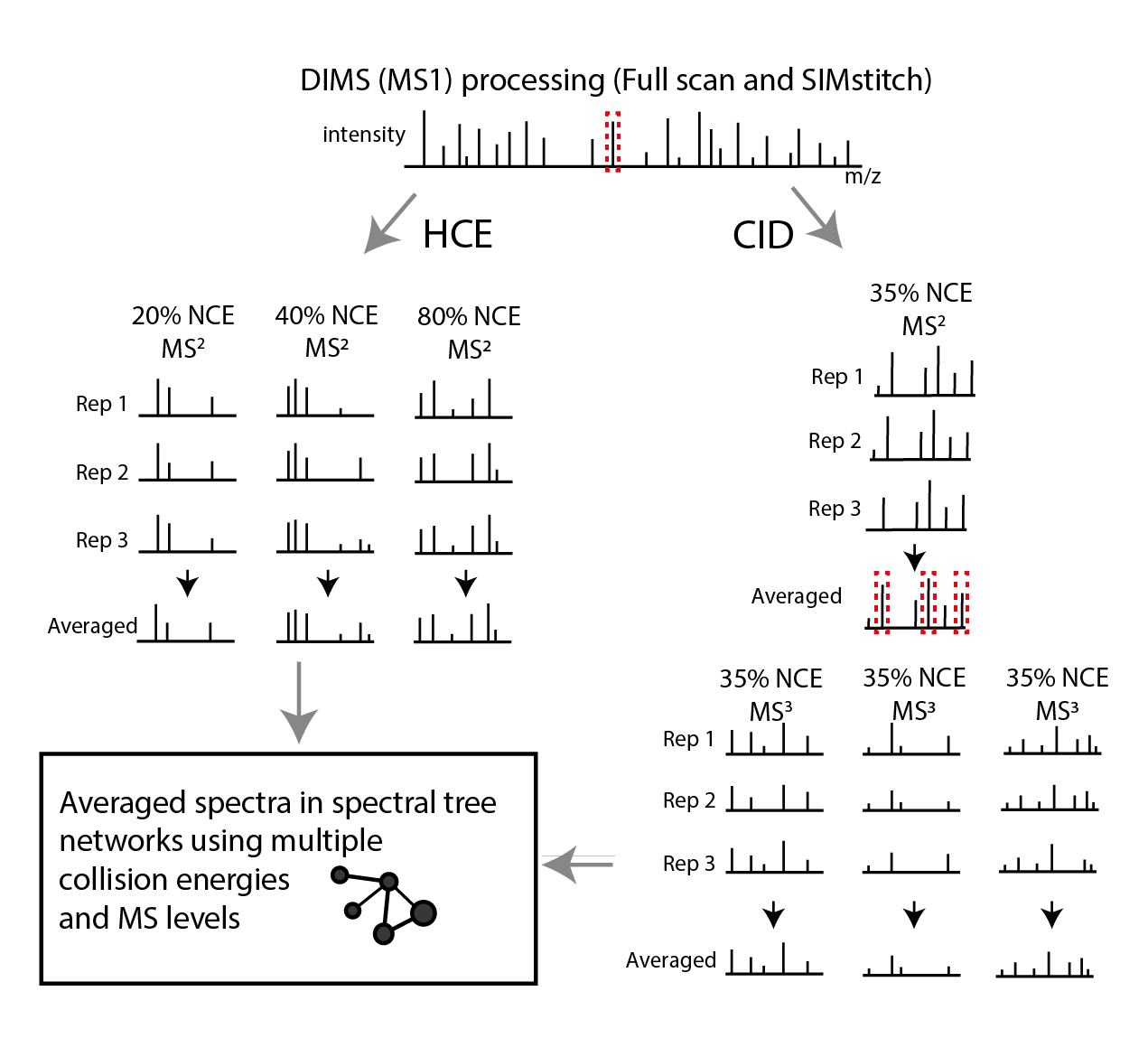


***Figure S8: DI-HRMS(/MS^n^) data processing schematic for MSnPy.*** *Schematic of how DI-HRMS(/MS^n^) data is averaged. HCE and CID data was collected within the same run and is initially processed and averaged separately and then merged at different levels for the creation of relevant spectra for annotation purposes. HCE = Higher energy collisional dissociation. CID = Collision induced dissociation. NCE = normalised collision energy.*

#### msPurity (DI-HRMS(/MS^n^))

The precursor ion purity was calculated for all the MS^1^ features using the msPurity.dimsPredictPurity Galaxy tool for all features observed from the DIMSpy peaklist generated for every well of the fractionation experiment for each assay.

It should be noted that the precursor ion purity scores used for the DI-HRMS data using the dimsPredictiPurity tool were recalculated for some assays to assess different parameters used from those in the originally submitted workflow. For clarity a separate Galaxy history has been created with the suffix (DI-HRMS predicted precursor ion purity) for each DI-HRMS based assay and within each of these histories are the final DI-HRMS precursor ion purity scores used for filtering the metabolite annotations.

Both the Galaxy tool msPurity.dimsPredictPurity and underlying R package are developed and maintained by the authors.

#### msp2db

To perform spectral matching of the DI-HRMS(/MS^n^) data we first need to generate an SQLite database of the query spectra to be used as input for msPurity.spectralMatching. This is done via the msp2db Galaxy tool for all the MS^n^Py-generated MSP files of the DI-HRMS(/MS^n^) data, generated for every well of the fractionation experiment for each assay.

Both the Galaxy tool msp2db and underlying python package are developed and maintained by the authors.

#### CAMERA-DIMS

Adduct and isotopes were checked using a modified version CAMERA to work on DI-HRMS data using the Galaxy tool CAMERA-DIMS for all the DIMSpy peaklist generated for every well of the fractionation experiment for each assay.

The Galaxy tool CAMERA-DIMS was developed by, and maintained by, the authors.

### Metabolite annotation

#### msPurity spectralMatching

The spectral matching for both (U)HPLC-HRMS(/MS) and the DI-HRMS(/MS^n^) data was performed using Galaxy tool msPurity.spectralMatching.

The library SQLite database used consist of 667,163 fragmentation spectra with 229,936 unique compounds (based on a unique InChiKey) was generated using the package msp2db - using all of the MoNA mass spectrometry database as of 23rd April 2021. The database also included an internal library of mass spectral fragmentation data collected in-house at Phenome Centre Birmingham.

The library spectra was filtered by ionisation type and only included the following instruments types: APCI-ITFT, APCI-ITTOF, CE-ESI-TOF, ESI-ITFT, ESI-ITTOF, ESI-QFT, ESI-QTOF, ESI-TOF, in source CID, in-silico QTOF, Ion trap, LC-APCI-ITFT, LC-APCI-QTOF, LC-APCI-Q, LC-ESI-IT, LC-ESI-ITFT, LC-ESI-ITTOF, LC-ESI-Q, LC-ESI-QFT, LC-ESI-QIT, LC-ESI-QQ, LC-ESI-QTOF, LC-ESI-TOF, LC-Q-TOF/MS, LC-QTOF, Linear Ion Trap, LIT, MALDI-QIT, MALDI-TOF, MALDI-TOFTOF, Orbitrap, QIT, QIT-FT, QIT-TOF, QqQ, Q-TOF, Quattro_QQQ, QTOF, Flow-injection QqQ/MS, LC-APPI-QQ, LC-ESI-QQQ and MALDI-QITTOF. Some of the fragmentation spectra did not have an instrument type defined; in those cases the instrument names were checked and spectra with relevant instruments were included.

After filtering for instrument types, the database consists of 648,243 fragmentation spectra with 221,575 unique compounds (based on a unique InChiKey).

Weighted vectors of the intensity and m/z were used for the spectral matching comparison (see equation 1) - for all analysis in this paper the weighting was as per the MassBank (Horai et al. 2010) approach, i.e. x = 0.5 and y = 2. The dot product cosine (equation 2 – where $Q$ represents the query spectra and $L$ represents the library spectra), was then calculated. Other similarity metrices were calculated (i.e. reverse dot product cosine and composite dot-product cosine) but not used as part of the final filtering and ranking of annotations.

| $w = [peak\_intensity]^{x} \times[mz]^{y}$ | (1) |
| --- | --- |
| $dpc = \frac{\sum w_{Q}\times w_{L}}{\sqrt{\sum w_{Q}^{2}\times\sum w_{L}^{2}}}$ | (2) |

For the LC-MS/MS spectral matching the msPurity.spectralMatching used the output of the msPurity.createDatabase tool and spectral matching was performed on all inter-averaged fragmentation spectra.

For the the DI-HRMS(/MS^n^) spectral matching the msPurity.spectralMatching used the output from msp2db that created an SQLite database of the averaged fragmentation spectra for each collision energy and was ran for every well of the fractionation experiment for each assay.

The precursor of the library and the query spectra need to be within +/- 5 ppm of one another.

Both the Galaxy tools of msPurity and the underlying R package described above are developed and maintained by the authors.

Mass spectral libraries used are available via github (github.com/computational-metabolomics/msp2db/releases/tag/v0.0.14-mona-23042021).

**Table S10**: Summary of fragmentation spectra used for spectral matching with msPurity

| **MSP files origin** | **Original source** | **Spectra count** | | | **Unique Compound count** | | |
| --- | --- | --- | --- | --- | --- | --- | --- |
|  |  | **Pos** | **Neg** | **All** | **Pos** | **Neg** | **all** |
| MoNA | embl-mcf | 692 | 601 | 1293 | 256 | 321 | 431 |
|  | fahfa | NA | 4290 | 4290 | NA | 4290 | 4290 |
|  | fiehn_hilic | 1712 | 1348 | 3060 | 980 | 813 | 1219 |
|  | fiehn_plasma | 4439 | 4216 | 8655 | 377 | 295 | 594 |
|  | gnps | 19554 | 4247 | 23801 | 10356 | 2747 | 10983 |
|  | hmdb | 2088 | 1074 | 3162 | 672 | 271 | 923 |
|  | lipidblast | 143342 | 342454 | 485796 | 110833 | 154770 | 197527 |
|  | massbank | 40040 | 19062 | 59102 | 5350 | 3934 | 7226 |
|  | metabobase | NA | 1254 | 1254 | NA | 290 | 290 |
|  | pathogen_box | 392 | NA | 392 | 392 | NA | 392 |
|  | respect | 3879 | 2495 | 6374 | 1149 | 907 | 1511 |
|  | riken_ims_oxidized_phospholipids | NA | 386 | 386 | NA | 386 | 386 |
|  | vaniya_fiehn_natural_products_library | 31944 | 12992 | 44936 | 2677 | 2464 | 2768 |
| Internal library | METASCI_HILIC | 682 | 676 | 1358 | 460 | 538 | 695 |
|  | METASCI_RP | 923 | 733 | 1656 | 514 | 442 | 634 |
|  | PCB_HILIC | 523 | 196 | 719 | 238 | 123 | 263 |
|  | PCB_LIPIDS | 326 | 257 | 583 | 154 | 159 | 214 |
|  | PCB_LIPIDS_IPA | 321 | 251 | 572 | 145 | 157 | 213 |
|  | PCB_RP | 454 | 400 | 854 | 230 | 187 | 289 |
| **Total** | | 251311 | 396932 | 648243 | 127953 | 168207 | 221575 |
| **Total (without LipidBlast in silico)** | | 107969 | 54478 | 162447 | 17150 | 13951 | 24589 |

#### MetFrag

Metabolite annotation using MetFrag was performed using the Galaxy tool of the same name. MetFrag is a combinatorial *in silico* fragmentation tool, that generates *in silico* spectra from potential matches of a hypothetical neutral masses or molecular formula of the fragmentation precursor ion.

When annotating the (U)HPLC-HRMS(/MS) data within the DMA workflow, the MetFrag Galaxy tool takes as input the MSP of the inter-averaged fragmentation spectra generated from msPurity.createMSP. When annotating DI-HRMS(/MS^n^) data from the DMA workflow, the MetFrag Galaxy tool takes as input the MSP of the combined and averaged spectra from multiple collision energies and MS levels, generated from MSnPy.convert-spectral-trees tools. This was run for every well of the fractionation experiment for each assay.

The analysis in this paper used a PostgreSQL database of all the compounds within PubChem to search against to generate the *in-silico* spectra. An overall weighted score was used of the following: Fragmentor score (which scores the fragmentation based on the intensities, m/z values and bond energies of the matches between the predicted spectra and the query spectra); the OfflineMetFusionScore (based on a predefined spectral library within MetFrag); and the Suspect list score (which ranks annotations against a list of suspected compounds – in our cases a list of natural products). The weights used for the DMA workflow were: FragmentScore: 0.2, OfflineMetFusionScore: 0.3 and SuspectListScore: 0.5. The scores were chosen based on the default values of the Galaxy tool based on the community development of the tool (FragmentScore: 0.4, OfflineMetFusionScore: 0.6 and SuspectListScore: 1) but scaled to equal 1 to easier integrate with other scores. Whilst the combining approach used here is convenient, further MetFrag analysis beyond this paper would benefit of using the scores independent as the overall score is somewhat biassed to both the suspect list and offline metfusion score and led to a rather harsh cutoff being used for final filtering of MetFrag (>0.95) to ensure we were always using a reliable “FragmentScore”.

We also note that use of the PubChem structural database for MetFrag means that the annotations are not restricted to existing metabolome knowledgebases – however this can lead to annotations where there is no prior evidence of either the compound occurring naturally, or of it having been detected using a mass spectrometer. As such, the use of MetFrag’s “SuspectedListScore” and the “OfflineMetFusionScore” means that compounds that have either previously been found as a natural product or that have previously been reported in MassBank, are prioritised, as these were considered more likely to be an observable metabolite in *D. magna*. In addition, the final filtering of MetFrag (>0.95) essentially forces any final reported annotation derived solely from MeFrag to have been on the “SuspectedListScore” and have a high scoring “OfflineMetFusionScore”.

The mass deviation for the database search was set at 5 ppm and the fragment peak match deviation with an absolute mass deviation of 0.001 Da.

The following adducts were considered for positive ionisation mode: [M+H]^+^,[M+Na]^+^,[M+NH_4_]^+^ and [M+K]^+^. The following adducts were considered for negative ionisation mode: [M-H]-,[M+Cl]-,[M+HCOO]^-^,[M+CH_3_COO]^-^ and [M-H+CH_3_COOH]^-^.

The MetFrag Galaxy tool is developed and maintained by the authors (with help within the Galaxy community).

#### SIRIUS CSI:FingerID

SIRIUS is a mass spectrometry analysis and annotation GUI and CLI software that can be used to perform isotope analysis, fragmentation trees analysis and predict metabolite structures using CSI:FingerID. The Galaxy tool SIRIUS CSI:FingerID, provides a wrapper of the SIRIUS CLI specifically to perform the fragmentation tree creation, CSI:FingerID metabolite annotation and the CANOPUS metabolite class annotation

When annotating the (U)HPLC-HRMS(/MS) data within the DMA workflow, the SIRIUS CSI:FingerID Galaxy tool takes as input the MSP of the inter-averaged fragmentation spectra generated from msPurity.createMSP. When annotating the DI-HRMS(/MS^n^) data from the DMA workflow, the SIRIUS CSI:FingerID tool takes in as input the combined and averaged spectra at each collision energy, converted into MSP format from the from the MSnPy Galaxy tool MSnPy.convert-spectra-trees tool and was run for every well of the fractionation experiment, for each assay.

The annotation workflow used included the “all biological” compound database for the CSI:FingerID database, specifying for Orbitrap based analysis and with 5 ppm mass deviation used. For each fragmentation spectra SIRIUS CSI:FingerID generates a rank list of potential structures - annotated to a partial InChiKey.

The following adducts were considered for positive ionisation mode: [M+H]^+^,[M+Na]^+^,[M+NH_4_]^+^ and [M+K]^+^. The following adducts were considered for negative ionisation mode: [M-H]-,[M+Cl]-,[M+HCOO]^-^,[M+CH_3_COO]^-^ and [M-H+CH_3_COOH]^-^.

The SIRIUS CSI:FingerID Galaxy tool was primarily developed by the authors (with help within the Galaxy community) and is maintained by the authors.

#### BEAMSpy

The BEAMSpy Galaxy tool was used to annotate metabolites detected during DI-HRMS(/MS^n^) and (U)HPLC-HRMS analyses. The BEAMSpy tool uses a calculated neutral mass of each MS1 feature and searches this against a Metabolite library of compounds. As there is no *D. magna* list of metabolites to search against, HMDB was used instead as it provides one of the larger resources of known metabolites. Note that no annotation was reported in this paper that was solely dependent on BEAMSpy metabolite annotation of MS1-only data.

When annotating the (U)HPLC-HRMS data with BEAMSpy within the DMA workflow, the BEAMSpy Galaxy tool takes as input a text (.tsv) file of the XCMS grouped chromatographic features (from the xcms.groupChemPeaks Galaxy tool). When annotating the DI-HRMS(/MS*^n^*) data from the DMA workflow, BEAMSpy Galaxy tool takes as input a text (.tsv) file containing DI-HRMS features generated by the DIMSpy.Get peaklist Galaxy tool. This was run for every well of the fractionation experiment for each assay.

#### MSnPy molecular formula annotation

*De novo* metabolite annotation to a molecular formula (i.e. without a predefined compound database) of the DI-HRMS(/MS^n^) data was done via the MSnPy.annotate-trees Galaxy tool. Due to the multiple collision energies and MS levels collected, the DI-HRMS(/MS^n^) data provided an opportunity to calculate more accurate *de novo* molecular formula annotations of mass spectrometry features than would usually be possible with (U)HPLC-HRMS(/MS) data. The MSnPy.annotate-trees used a ppm tolerance of 5, applied a heuristic rules method to calculate the molecular formula, and used the pre-calculated molecular formula database – mfdb (<https://mfdb.bham.ac.uk>) – consisting of precomputed molecular formulas based on the assumption that the compound would comprise the following chemical elements (CHNOPS) within the measured mass range of the mass spectrometer. The “MSnPy.rank-spectral-trees”Galaxy tool is then used to rank the annotated trees based on the number of neutral losses explained by the molecular formula annotation of the annotated tree. The MSnPy molecular formula annotation and ranking was ran on every well of the fractionation experiment for each assay.

Additionally, it should be noted that, when the annotations described here were summarised in the main paper, only molecular formulae were included in instances where there were ten or fewer top-ranked candidates.

The MSnPy molecular formula annotation Galaxy tool and underlying python package was developed by and maintained by the authors.

### Combining

#### msPurity combineAnnotation

All (U)HPLC-HRMS(/MS) derived annotations and processed data are combined into a single SQLite database using the msPurity.combineAnnotation Galaxy tool, where the database generated at an earlier stage (msPurity.createDatabase) and updated via the spectral matching analysis (msPurity.spectralMatching) is combined with the annotation results from MetFrag, SIRIUS CSI:FingerID and BEAMSpy of the (U)HPLC-HRMS(/MS) data.

In addition to the annotation approaches above, we also calculated a “biological similarity metric”. Here, a filtered list of HMDB compounds was generated in which entries determined to be exogenous metabolites were removed. The chemical structures of these retained HMDB metabolites was then compared against all structures available in PubChem, to generate tanimoto scores. For Pubchem structures found to be dissimilar to the filtered list of HMDB metabolites based on the calculated Tanimoto scores, i.e. not similar to any ‘endogenous’ compound reported in the largest metabolome database, these were less likely to be accurate annotations for HRMS/MSn spectra – or should at least be deprioritised as candidate annotations.

Compounds are aligned across all approaches based on matching InChIKeys (Heller and McNaught, 2009) and the annotations for specific (U)HPLC-HRMS features are ranked based on a combined score of all the annotation approaches where each approach is given a weight. The following weightings were used for the Galaxy Annotation Workflow: spectral-matching: 0.45, metfrag: 0.15, SIRIUS-CSI:fingerID: 0.25 and MS1 lookup: 0.1 and biosimilarity score 0.05.

Weightings were selected by evaluating annotation performance on the MTBLS749 MetaboLights study using a combination of empirical testing and expert judgment. Sensitivity and specificity were calculated following Chao et al. (2020) to guide selection, alongside expert considerations: spectral matching was prioritized as the most reliable source, with MS/MS-based tools like MetFrag and SIRIUS given greater weight than approaches lacking fragmentation data. Weight combinations that didn’t align with these principles were excluded, and the final set demonstrating the best combined sensitivity and specificity was chosen.

Although the approach of selecting the weights here is subjective, being heavily dependent on both the choice of metabolites to calculate the sensitivity and specificity and the “expert” considerations, following the assessment of the (U)HPLC-HRMS(/MS) component of the experimental & computational DMA workflow, wherein we could annotate 89.6% of reference standards when filtering for the 1st top ranked metabolite – we deemed the weightings sufficient to use.

The msPurity.combineAnnotation tool used the following SQLite database of PubChem compounds: https://doi.org/10.5281/zenodo.7756132The msPurity.combineAnnotation Galaxy tool and underlying R package was developed by and maintained by the authors.

#### LC-Fractionation processor tool

Both the (U)HPLC-HRMS(/MS) and the DI-HRMS(/MS*^n^*) derived annotations and processed data are combined into a single SQLite database using the Galaxy tool “LC Fractionation processor”. This takes as input the combined (U)HPLC-HRMS(/MS) annotations from the msPurity.combineAnnotation Galaxy tool as well as for every well of the fractionation experiment for each assay: the DI-HRMS peaklists from DIMSpy, the DI-HRMS(/MS^n^) peaklists from MSnPy, the DI-HRMS(/MS^n^) MetFrag annotations, the DI-HRMS(/MS^n^) SIRIUS CSI:FingerID annotations, the DI-HRMS(/MS^n^) spectral matching annotations, the DI-HRMS BEAMSpy MS^1^ annotations and the molecular formula annotations from MSnPy.

Compound annotations were aligned for each DI-HRMS feature using the same approach as msPurity.comebinAnnotation and used the same weights: spectral-matching: 0.45, metfrag: 0.15, SIRIUS-CSI:fingerID: 0.25 and MS1 lookup: 0.1 and biosimilarity score 0.05.

The “LC fractionation processor” Galaxy tool used the following SQLite database of PubChem compounds: https://doi.org/10.5281/zenodo.7756132

The “LC fractionation processor” Galaxy tool was developed by, and is maintained by, the authors.

### Galaxy workflow reproducibility

The Workflows can be viewed directly from our Galaxy instance <https://dma.galaxy.bham.ac.uk/>.

Manual inspection of Galaxy histories was performed and in multiple cases jobs needed to be either re-run due to faults with API calls to web-services, cluster and server related errors, timeout errors, or specific raw files being problematic. In cases where the job could not be repeated successfully, the failed job output was filtered from its dataset collection and the remaining jobs in the history were restarted. All manual interactions are recorded in each of the analysis Galaxy histories.

Spectral matching was repeated with msPurity.spectralMatching in each history to include a wider range of instrument types and library spectra. The subsequent tools msPurity.combineAnnotations and the LC-Fractionation processor tool were repeated to include the updated spectral matching annotations.

The LC-Fractionation tool was repeated several times in each history whilst issues with problematic files and bugs were resolved. The redundant LC fractionation result files have been deleted from the history (but the metadata can still be viewed for traceability). The LC-Fractionation tool is the last step in the workflow so repeating the analysis has no effect on any other tool in the workflow.

## Combining and summarising all annotations

Five main sources of annotations were combined into a final list of Metabolite annotations: Galaxy workflow annotations, GNPS workflow annotations, mzCloud annotations, NMR annotations and GC-EI-HRMS annotations. All data were combined into a single table encompassing all annotations across every assay.

Entries in this table were filtered to only include annotations that were ranked as the top 1 annotation with either a spectral matching dot product cosine score >0.7, at least 2 shared peaks between library and query spectra, and at least 10% of peaks explained; or be ranked 1st from SIRIUS CSI:FingerID; or have a MetFrag score >0.95. All Galaxy workflow annotations also had to be derived from fragmentation spectra acquired from a precursor ion with “precursor ion purity” >0.5.

Any annotations where the mass difference between the query precursor *m/z* and the library precursor *m/z* for GNPS and mzCloud was above 10 ppm were disregarded. The SIRIUS CSI:FingerID annotations were also filtered to remove any annotations where the calculated neutral mass of the query was not within 10 ppm of the annotation. This is already done within the Galaxy workflow annotations for spectral matching within *msPurity.spectralMatching* and within the MetFrag tool. From all the approaches only the top ranked annotations were used for the final summary of results.

Following the above filtering, annotations derived from mzCloud and the Galaxy workflow could still have more than one annotation per feature (i.e. when there are multiple top 1 ranked annotations). If all the annotations within the top 1 ranked annotations had the same partial InChiKey (i.e., the first block of the InChiKey that encodes the molecular skeleton) and only differed in stereochemistry, one representation of the structure was carried forward. If the annotations that were top 1 ranked had different molecular formulae, the annotation with the lowest ppm error between the theoretical neutral monoisotopic mass and the calculated monoisotopic mass of the feature (based on the adduct used for the annotation) was selected. Following these steps, if the annotation could still not be distilled to a single metabolite annotation (unique InChiKey) the annotation was not included in the final list of metabolites.

The LC-MS annotations for the *Daphnia* assays were further quality-flagged by extracting the XCMS peaklist objects from the Galaxy workflow and applying blank filtering. For each feature, if a corresponding peak was detected in the blank sample, the intensity in the daphnia samples was required to be at least 10-fold higher than that in the blank for the feature to be retained. All LC-MS-based annotations were required to either be directly linked to an XCMS feature that passed the blank-filtering threshold (when using the Galaxy workflow); or if the annotation was generated through the mzCloud or GNPS workflows, such that both the precursor m/z (+/-10 ppm) and the XCMS feature *m/z* (+/-10 ppm) overlapped and fall within the retention time window defined for a valid XCMS feature. All annotations were chemically classified using ClassyFire (Djoumbou Feunang et al., 2016).

## Assessment of the computational and experimental DMA workflow with metabolite reference standards

Metabolite reference standards were analysed to evaluate the effectiveness of the overall DMA workflow, specifically the (U)HPLC-HRMS(/MS) component.

See **Supplemental Table S11** (provided in separate excel file) for a summary of the metabolite reference standards used.

# Supplemental - results

## (U)HPLC-HRMS(/MS) method optimisation

(U)HPLC-HRMS(/MS) method optimisation was undertaken using Syncronis Phenyl and Accucore Amide LC columns. Here, the objective was to maximise both the number and chromatographic distribution of reproducibly detectable metabolic features (RDMFs) accessible during DMA of *Daphnia magna* ‘crude’ polar extract and associated SPE fractions.

### Syncronis Phenyl (PHE)

#### Phase one

Phase one of optimising a Syncronis Phenyl-based UHPLC-HRMS(/MS) method for DMA involved exploration of the impact of altering mobile phase organic solvent (methanol or acetonitrile) and additive (0.1% v/v formic acid or 5 mM ammonium acetate) types on RDMF counts and chromatographic distributions.

**Figures S9** and **S10** provide representative examples of the two-dimensional distribution (*m/z* versus retention time) of RDMFs detected using methods ‘Ph-CO-[1-4]’. As demonstrated in the marginal boxplots of these figures, the m/z distribution of RDMFs across methods was found to be highly similar, though clear differences existed with respect to the chromatographic distribution of RDMFs. Methods ‘Ph-CO-3’ and ‘Ph-CO-4’, both of which used methanol as mobile phase organic solvent, had superior (i.e. wider) distribution of RDMFs across the chromatographic elution window compared to methods ‘Ph-CO-1’ and ‘Ph-CO-2’, where acetonitrile was used as organic solvent. Median retention times for RDMFs (and associated interquartile ranges) are summarised in **Table S12**, with significantly greater values recorded for methods ‘Ph-CO-3’ and Ph-CO-4’ compared to ‘Ph-CO-1’ and ‘Ph-CO-2’. The lower eluotropic strength of methanol compared to acetonitrile under reversed phase LC conditions, coupled with methanol’s lack of *π*-orbital electrons that could disrupt *π*-*π* interactions between metabolites and stationary phase moieties, likely contributed to these observations.

The total number of RDMFs recorded using methods ‘Ph-CO-[1-4]’ is summarised in **Figure S11**. Method ‘Ph-CO-3’ consistently outperformed all other methods in this regard, irrespective of the sample type or ionisation mode combination considered. Interestingly, all methods yielded more RDMFs when SPE fraction pool DCX12DAX34 was analysed compared to DAX12DCX34, potentially indicating a greater number of anionic versus cationic metabolites in the polar extracts of *Daphnia magna* (though differences in detection sensitivity and selectivity for anionic and cationic metabolites cannot be excluded as contributory factors in this observation).

Overall, method ‘Ph-CO-3’ outperformed all other methods evaluated during phase one of Syncronis Phenyl-based (U)HPLC-HRMS(/MS) method optimisation, both in terms of the total number and chromatographic distribution of RDMFs. The next most-performant method, method ‘Ph-CO-4’, offered similar performance metrics to ‘Ph-CO-3’, however upon closer inspection of raw chromatographic data (not shown) it was found that peak widths were substantially wider for many of the most intense features recorded during negative ionisation mode analyses. These wider peak widths would have proven deleterious to maximising the number of features for which HRMS/MS data could be acquired, and for post-column fractionation during DMA experiments. Method ‘Ph-CO-3’ was therefore taken forward for further optimisation.

**Table S12**: Median and interquartile range of retention times for RDMFs recorded in DMA (U)HPLC-HRMS/MS method optimisation experiments

|  | **RDMF retention time metrics (minutes)** | | | | | | | |
| --- | --- | --- | --- | --- | --- | --- | --- | --- |
| **SPE fraction pool** | **DAX12DCX34** | | | | **DCX12DAX34** | | | |
| **Ionisation mode** | **Positive** | | **Negative** | | **Positive** | | **Negative** | |
| **Method name** | **Median** | **IQR** | **Median** | **IQR** | **Median** | **IQR** | **Median** | **IQR** |
| Ph-CO-1 | 6.3 | 5.3 | 5.3 | 5.8 | 6.6 | 6.9 | 5.9 | 6.9 |
| Ph-CO-2 | 5.7 | 6.0 | 5.0 | 5.3 | 7.0 | 5.1 | 6.1 | 4.8 |
| Ph-CO-3 | 8.6 | 9.6 | 7.8 | 9.0 | 9.1 | 9.9 | 8.7 | 8.5 |
| Ph-CO-4 | 7.0 | 11.2 | 6.2 | 8.2 | 10.3 | 9.6 | 9.0 | 8.4 |
| Ph-FO-1 | 7.6 | 12.5 | 5.1 | 9.7 | 6.5 | 11.9 | 6.3 | 9.5 |
| Ph-FO-2 | 1.2 | 11.6 | 0.9 | 5.5 | 6.8 | 15.4 | 5.6 | 11.3 |
| Ph-FO-3 | 3.1 | 11.7 | 1.5 | 8.6 | 6.7 | 11.8 | 6.6 | 10.4 |
| AA-CO-1 | 2.4 | 5.1 | 2.5 | 5.0 | 3.1 | 5.7 | 2.7 | 5.9 |
| AA-CO-2 | 2.4 | 3.6 | 2.3 | 3.9 | 4.3 | 5.7 | 4.4 | 5.8 |
| AA-CO-3 | 2.8 | 4.4 | 2.6 | 4.2 | 4.1 | 5.4 | 4.0 | 5.4 |
| AA-Resus-1 | 3.9 | 7.5 | 6.6 | 7.8 | 4.2 | 8.2 | 7.1 | 8.3 |
| AA-Resus-2 | 3.0 | 6.7 | 5.0 | 7.3 | 2.6 | 6.8 | 3.1 | 6.7 |
| AA-Resus-3 | 8.8 | 10.6 | 9.3 | 10.2 | 11.2 | 11.6 | 11.5 | 5.4 |
| AA-Resus-4 | 7.5 | 8.7 | 9.1 | 8.0 | 8.8 | 9.9 | 10.2 | 4.6 |
| AA-FO-1 | 3.0 | 8.2 | 2.6 | 6.6 | 7.6 | 9.4 | 8.2 | 9.3 |
| AA-FO-2 | 5.5 | 6.8 | 5.5 | 6.6 | 6.9 | 7.3 | 6.3 | 7.0 |
| AA-FO-3 | 4.6 | 9.2 | 7.6 | 9.4 | 7.1 | 9.7 | 7.3 | 9.2 |
| AA-FO-4 | 5.1 | 7.4 | 5.9 | 6.9 | 6.9 | 8.1 | 7.2 | 7.5 |
| AA-FO-5 | 4.8 | 7.8 | 5.3 | 7.9 | 6.4 | 9.6 | 5.8 | 9.6 |
| AA-FO-6 | 6.4 | 9.5 | 6.0 | 9.5 | 7.0 | 9.7 | 7.0 | 9.6 |

Footnotes: IQR - interquartile range; RDMF - reproducibly detectable metabolic feature (see section 1.9 for details)


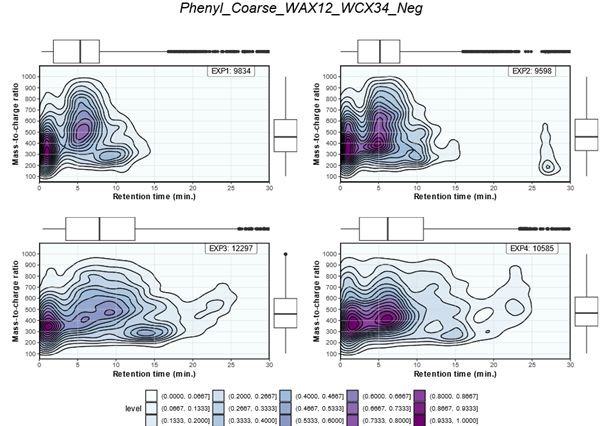


***Figure S9****: 2-dimensional density plot of reproducibly detectable metabolic features detected in sample DAX12DCX34 (a pool of Daphnia magna polar extract SPE fractions) under negative ionisation mode conditions using methods ‘Ph-CO-1’ (****top-left****), ‘Ph-CO-2’ (****top-right****), ‘Ph-CO-3’ (****bottom-left****) and ‘Ph-CO-4’ (****bottom-right****). Text boxes in the upper-right corner of each subplot indicate the total RDMFs.*

*
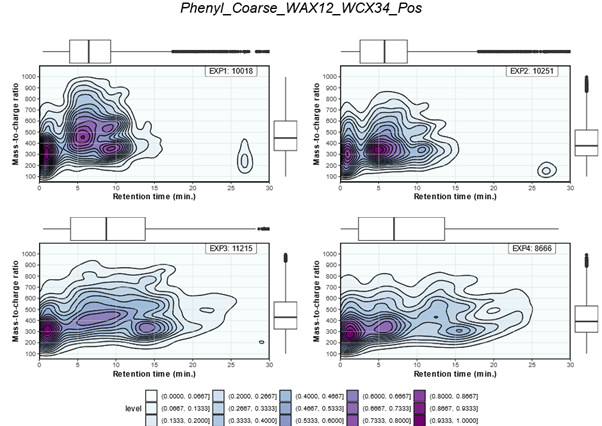
****Figure S10****: 2-dimensional density plot of reproducibly detectable metabolic features detected in sample DAX12DCX34 (a pool of Daphnia magna polar extract SPE fractions) under positive ionisation mode conditions using methods ‘Ph-CO-1’ (****top-left****), ‘Ph-CO-2’ (****top-right****), ‘Ph-CO-3’ (****bottom-left****) and ‘Ph-CO-4’ (****bottom-right****). Text boxes in the upper-right corner of each subplot indicate the total RDMFs.*


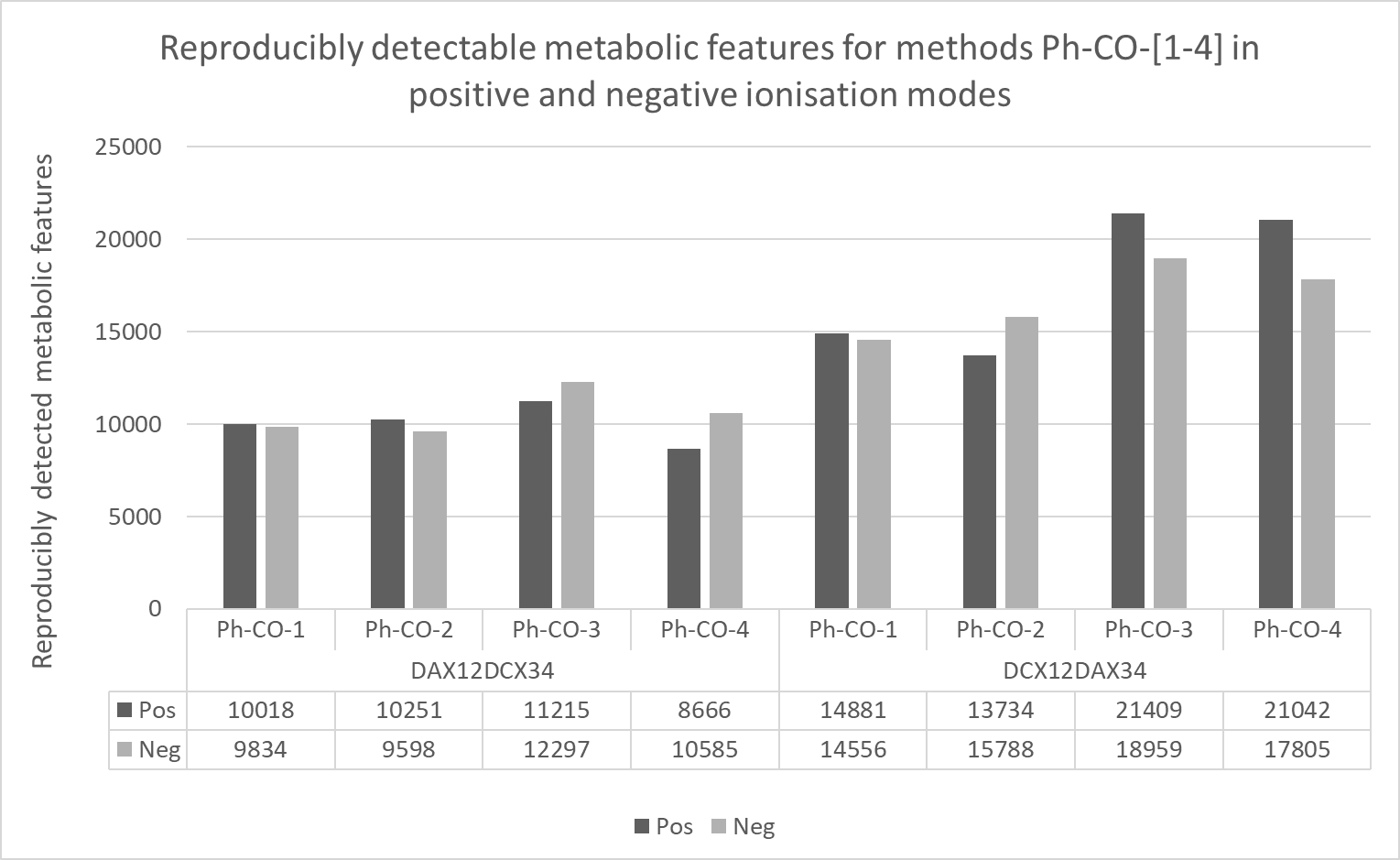


***Figure S11****: Count of reproducibly detectable metabolic features (RDMFs) detected in both positive (pos) and negative (neg) ionisation modes using methods ‘Ph-CO-[1-4]’ as part of phase one of Syncronis Phenyl-based (U)HPLC-HRMS(/MS) method optimisation.*

#### Phase two

Phase two of optimising a Syncronis Phenyl-based UHPLC-HRMS(/MS) method for DMA experiments, involved further refinement of the most performant method from the first phase of optimisation, method ‘Ph-CO-3’. Three methods, ‘Ph-FO-1’, ‘Ph-FO-2’ and ‘Ph-FO-3’ – each a partially adapted version of method ‘Ph-CO-3’ (see **Table S7** for details) – were therefore evaluated based on RDMF counts (**Figure S12**) and chromatographic distributions (**Figures S13** and **S14**; representative examples of the 2-dimensional distribution (*m/z* versus retention time) of recorded RDMFs) resulting from positive and negative ionisation mode analyses of samples DAX12DCX34 and DCX12DAX34.

Based on the sum total of RDMF counts recorded across each sample type and ionisation mode combination, as individually summarised in **Figure S12,** method ‘Ph-FO-1’ was found to outperform methods ‘Ph-FO-2’ and ‘Ph-FO-3’, yielding 19687 RDMFs in total compared to 16379 and 16205 RDMFs for methods ‘Ph-FO-2’ and ‘Ph-FO-3’, respectively (note, no dereplication was applied to account for features detected under multiple conditions). This result is underpinned by the greater number of RDMFs recorded using method ‘Ph-FO-1’, compared to ‘Ph-FO-2’ and ‘Ph-FO-3’, for all ionisation mode and sample type combinations considered, except for positive mode analysis of sample DCX12DAX34. Method ‘Ph-FO-1’ was therefore identified as the best performing method in terms of absolute RDMF counts.

With regards to the chromatographic distribution of the RDMFs recorded using methods ‘Ph-FO-[1-3]’, median and interquartile range values for RDMF retention times were highly similar across all methods for analysis of DCX12DAX34, as summarised in **Table S12**. Under both positive and negative ionisation conditions, median retention times for RDMFs were, respectively: 6.5 (11.9) and 6.3 (9.5) minutes for method ‘Ph-FO-1’; 6.8 (15.4) and 5.64 (11.3) minutes for method ‘Ph-FO-2’, and; 6.7 (11.8) and 6.6 (10.4) minutes for method ‘Ph-FO-3 (interquartile ranges in parentheses). Method ‘Ph-FO-2’, with its larger interquartile range values in both positive and negative ionisation modes, was therefore found to offer superior separation of RDMFs in DCX12DAX34.

Median RDMF retention time values for analysis of DAX12DCX34 were strikingly different to those recorded for DCX12DAX34: 7.6 (12.5) and 5.1 (9.7) minutes for ‘Ph-FO-1’; 1.2 (11.6) and 0.9 (5.5) minutes for ‘Ph-FO-2’, and; 3.1 (11.7) and 1.5 (8.6) minutes for ‘Ph-FO-3’ (interquartile ranges in parentheses). For methods ‘Ph-FO-2’ and ‘Ph-FO-3’ therefore, the first 50% of eluted RDMFs appeared to be highly congested at the start of the chromatographic elution space, a wholly undesirable situation for downstream application in the DMA analytical workflow, wherein extensive metabolome fractionation was sought prior to in-depth DI-HRMS(/MS^n^) analyses. The exact causes of lower median RDMF retention time values for methods ‘Ph-FO-2’ and ‘Ph-FO-3’ were unclear. In part, this could potentially have been linked to the on-average wider peak widths for these methods, being 11.51 s (10.02 s) and 14.42 s (10.77 s) respectively, as compared to method ‘Ph-FO-1’ at 10.91 s (5.86 s) (parenthesis are IQRs). These wider peak widths may have deleteriously impacted the effectiveness of computational peak picking, resulting in an artificially lower number of RDMFs than were truly present. Nevertheless, even if peaks were accurately detected, their broadness would have been highly undesirable for application in the DMA analytical workflow. Method ‘Ph-FO-1’, with its superior RDMF counts and acceptable RDMF distributions, was therefore selected for inclusion in the DMA analytical workflow.


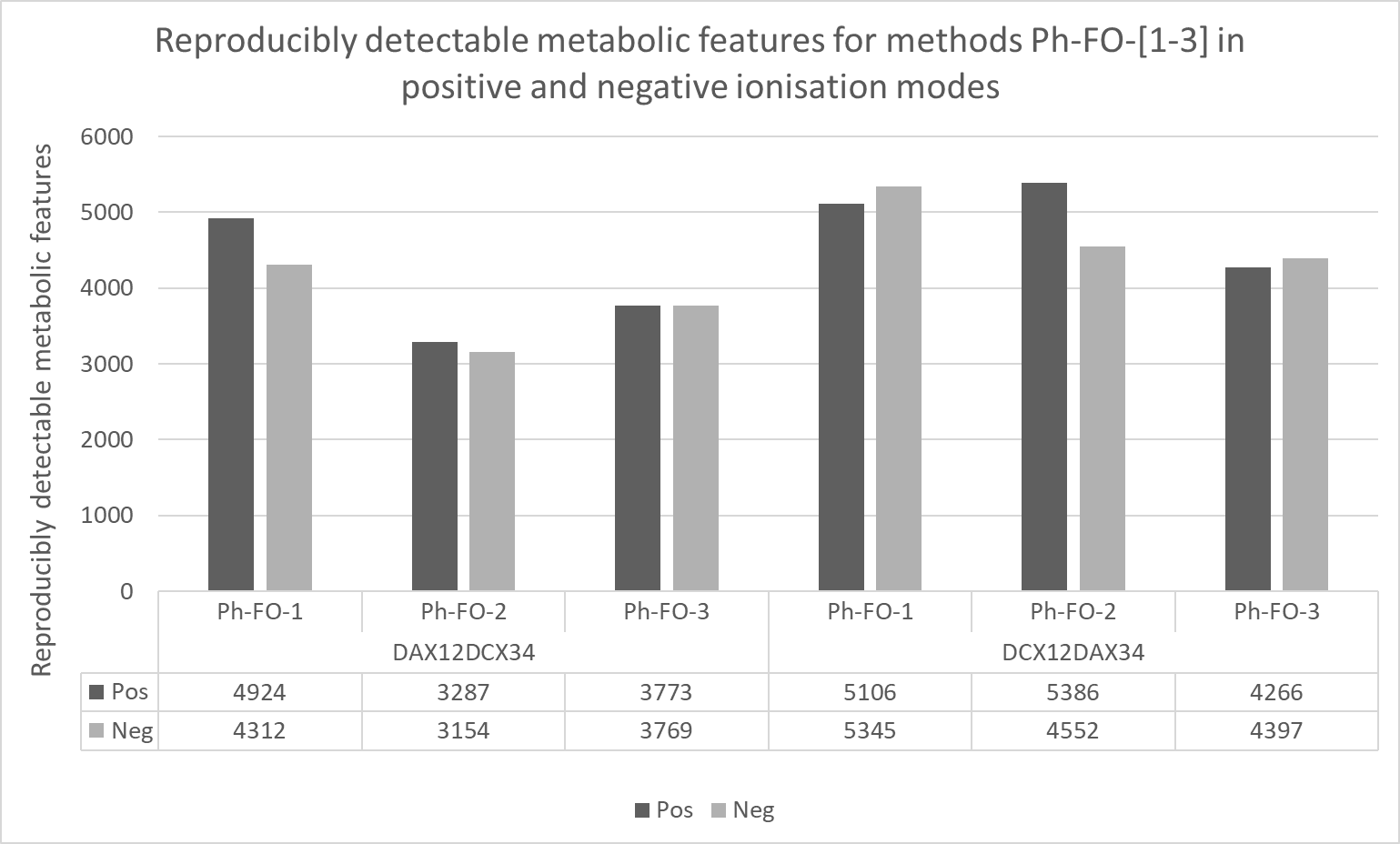


***Figure S12****: Counts of reproducibly detectable metabolic features (RDMFs) detected in both positive (‘pos’) and negative (‘neg’) ionisation modes using methods ‘Ph-FO-[1-3]’ during phase two of Syncronis Phenyl-based (U)HPLC-HRMS(/MS) method optimisation.*


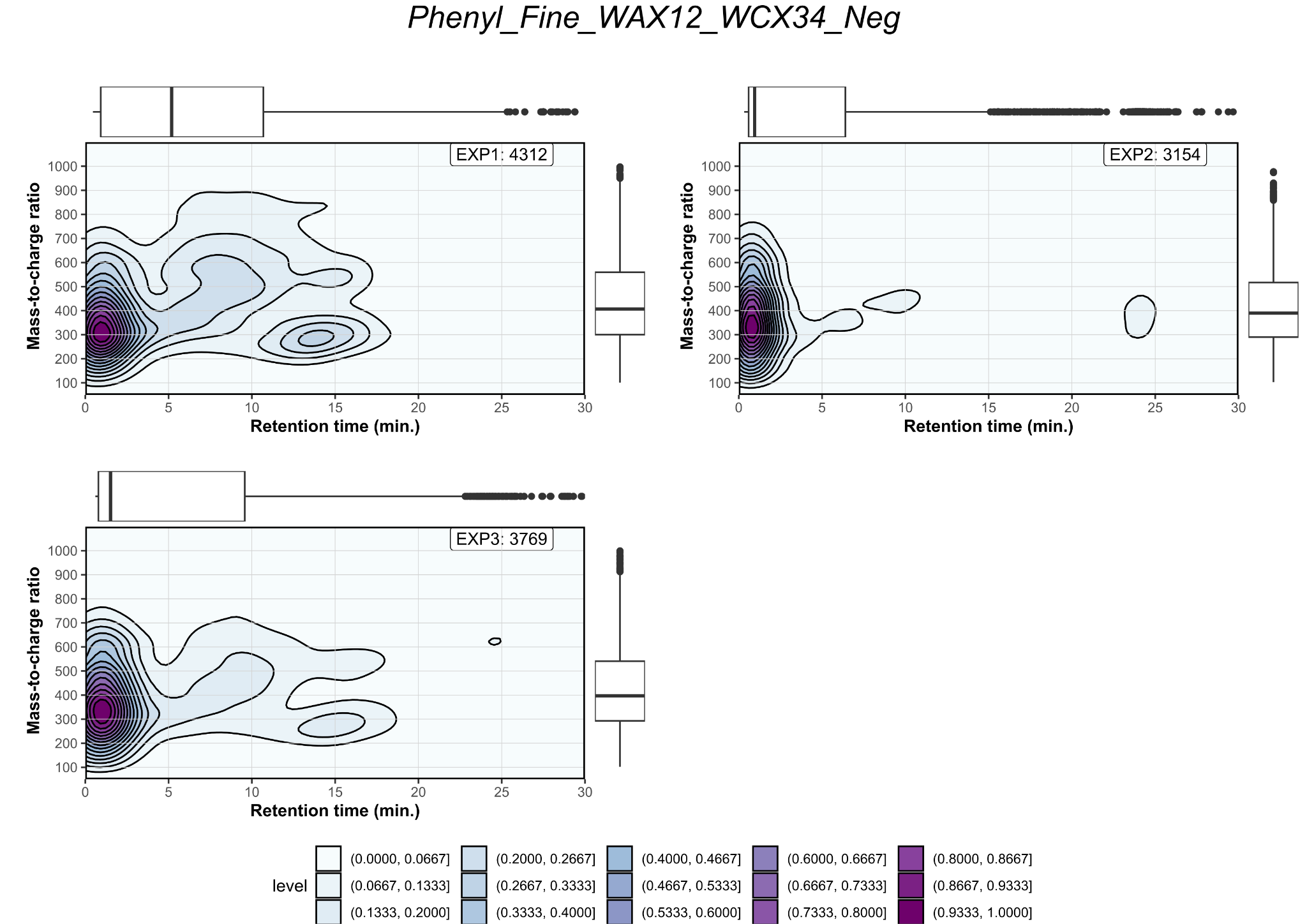


***Figure S13****: 2-dimensional density plot of reproducibly detectable metabolic features detected in sample DAX12DCX34 (a pool of Daphnia magna polar extract SPE fractions) under negative ionisation mode conditions using methods ‘Ph-FO-1’ (****top-left****), ‘Ph-FO-2’ (****top-right****) and ‘Ph-FO-3’ (****bottom-left****). Text boxes in the upper-right corner of each subplot indicate the total RDMFs.*


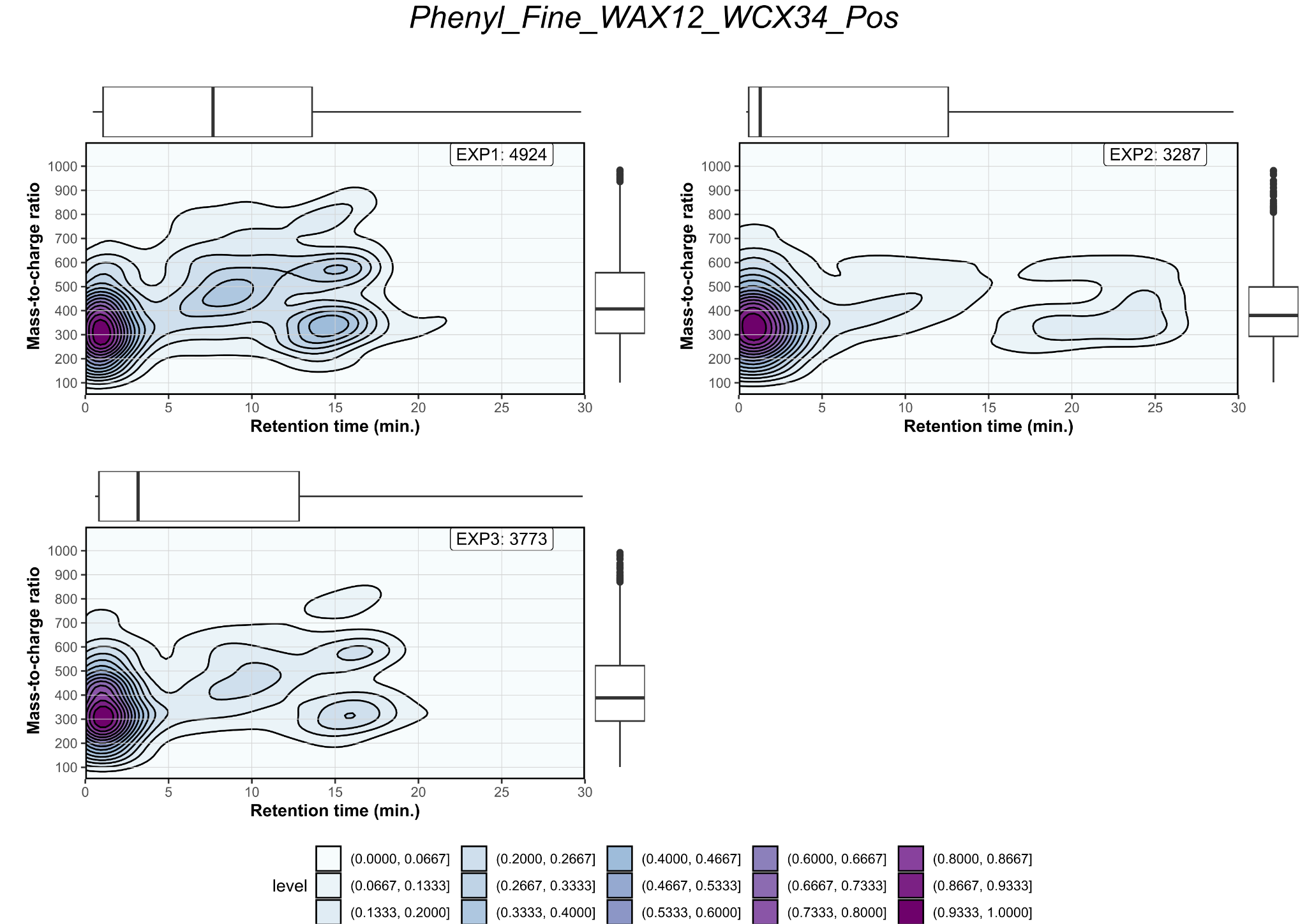


***Figure S14****: 2-dimensional density plot of reproducibly detectable metabolic features detected in sample DAX12DCX34 (a pool of Daphnia magna polar extract SPE fractions) under positive ionisation mode conditions using methods ‘Ph-FO-1’ (****top-left****), ‘Ph-FO-2’ (****top-right****) and ‘Ph-FO-3’ (****bottom-left****). Text boxes in the upper-right corner of each subplot indicate the total RDMFs.*

### Accucore Amide (AMD)

#### Phase one

Phase one of optimising an Accucore Amide-based HILIC-HRMS(/MS) method for DMA, involved exploration of the impact of coarse shifts in mobile phase pH (spanning column operational limits) and additive type on RDMF counts and distributions.

**Figure S15**, below, summarises the count of RDMFs recorded in samples DAX12DCX34 and DCX12DAX34, when analysed by methods ‘AA-CO-[1-3]’ under positive and negative ionisation mode conditions. Sum totals of RDMFs across each combination of sample type and ionisation mode were: 13368 for method ‘AA-CO-1’ (pH 3.0), 12957 for method ‘AA-CO-2’ (pH 6.6), and 13476 for method ‘AA-CO-3’ (pH 5.8). Based on these values, no single method configuration (i.e. mobile phase pH and additive type combination) could be regarded as significantly more performant than any of the other methods tested. Likewise, the individual RDMF values for each combination of ionisation mode and sample type, as presented in **Figure S15**, revealed that no single analytical configuration was substantially superior. Greater numbers of RDMFs were however, evidently recorded when positive mode ionisation was applied, indicating potentially distinct chromatographic distributions of anionic and cationic metabolites.

The chromatographic distribution of RDMFs recorded using methods ‘AA-CO-[1-3]’ (see **Figures S16** and **S17** for representative examples of the 2-dimensional (*m/z* versus retention time) distribution of RDMFs recorded with these methods) provided additional insights into the relative similarities and differences of each method. As summarised in **Table S12**, median retention times for RDMFs detected in sample DCX12DAX34 were found to be markedly lower for method ‘AA-CO-1’, at 3.1 min in positive mode and 2.7 min in negative mode, compared to methods ‘AA-CO-2’ or ‘AA-CO-3’, where corresponding medians were 4.3 and 4.4 min, and 4.1 and 4.0 min, respectively. For the same sample, interquartile range values of RDMF retention times were similar, being 5.7, 5.7 and 5.4 min in positive mode, and 5.9, 5.8 and 5.4 min in negative mode, for methods ‘AA-CO-1’, ‘AA-CO-2’, and ‘AA-CO-3’, respectively. Together, these values indicated that lower chromatographic resolution was achieved for the first 50% of RDMFs eluted using method ‘AA-CO-1’, compared to methods ‘AA-CO-2’ and ‘AA-CO-3’. Conversely, the larger span of time between the median and upper quartile of RDMF retention times for method ‘AA-CO-1’, indicated potential superior separation of metabolites during this elution period compared to ‘AA-CO-2’ and ‘AA-CO-3’. A possible explanation for this observation is that sample DCX12DAX34, by virtue of its constituent SPE fractions, may have been enriched with anionic metabolites. Under the acidic elution conditions of method ‘AA-CO-1’ (pH 3.0), dissociation of any weakly acidic constituents may have been fully or partially suppressed, in so doing potentially reducing their capacity to interact with the HILIC stationary phase and/or to partition into the aqueous-rich layer thereon, resulting in decreased retention times. With rapid elution of these components, the latter part of the chromatogram may appear to offer greater metabolite separation, due simply to fewer metabolic features remaining to be eluted.

The chromatographic distribution of RDMFs recorded in sample DAX12DCX34 were, unlike those in sample DCX12DAX34, highly similar across methods ‘AA-CO-[1-3]’. Median retention time values of RDMFs were 2.4, 2.4 and 2.8 mins in positive ionisation mode, and 2.5, 2.3 and 2.6 mins in negative ionisation mode, respectively. Corresponding interquartile ranges were 5.1, 3.6 and 4.4 mins, and 5.0, 3.9 and 4.2 mins, indicating slightly superior separation of metabolites by method ‘AA-CO-1’.

Given that methods ‘AA-CO-[1-3]’ achieved similar performance in terms of both the distribution and number of RDMFs recorded, method ‘AA-CO-3’ was ultimately selected for further optimisation on the basis that its operational pH, pH 5.8, may facilitate ionisation (and thus promote retention under HILIC conditions) of a broad range of both anionic and cationic metabolites.

***Figure S15 -*** *Counts of reproducibly detectable metabolic features (RDMFs) detected in samples DAX12DCX34 and DCX12DAX34 using methods ‘AA-CO-[1-3]’, under both positive (‘pos’) and negative (‘neg’) ionisation mode conditions, during phase one of Accucore Amide-based HILIC-HRMS(/MS) method optimisation.*


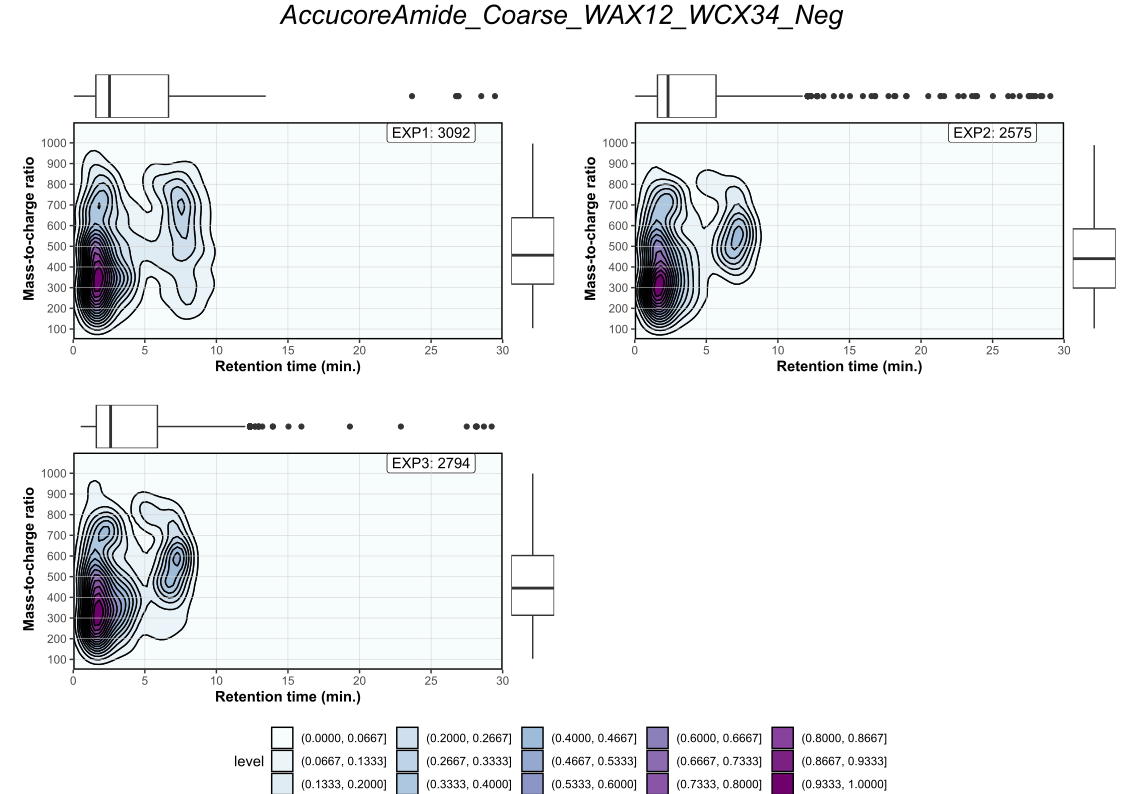


***Figure S16****: 2-dimensional density plot of reproducibly detectable metabolic features (RDMFs) detected through negative ionisation mode analysis of sample DAX12DCX34 using methods ‘AA-CO-1’ (****top-left****), ‘AA-CO-2’ (****top-right****) and ‘AA-CO-3 (****bottom-left****) during phase one of Accucore Amide-based HILIC-HRMS(/MS) method optimisation. Text boxes in the upper-right corner of each subplot indicate the total RDMFs.*


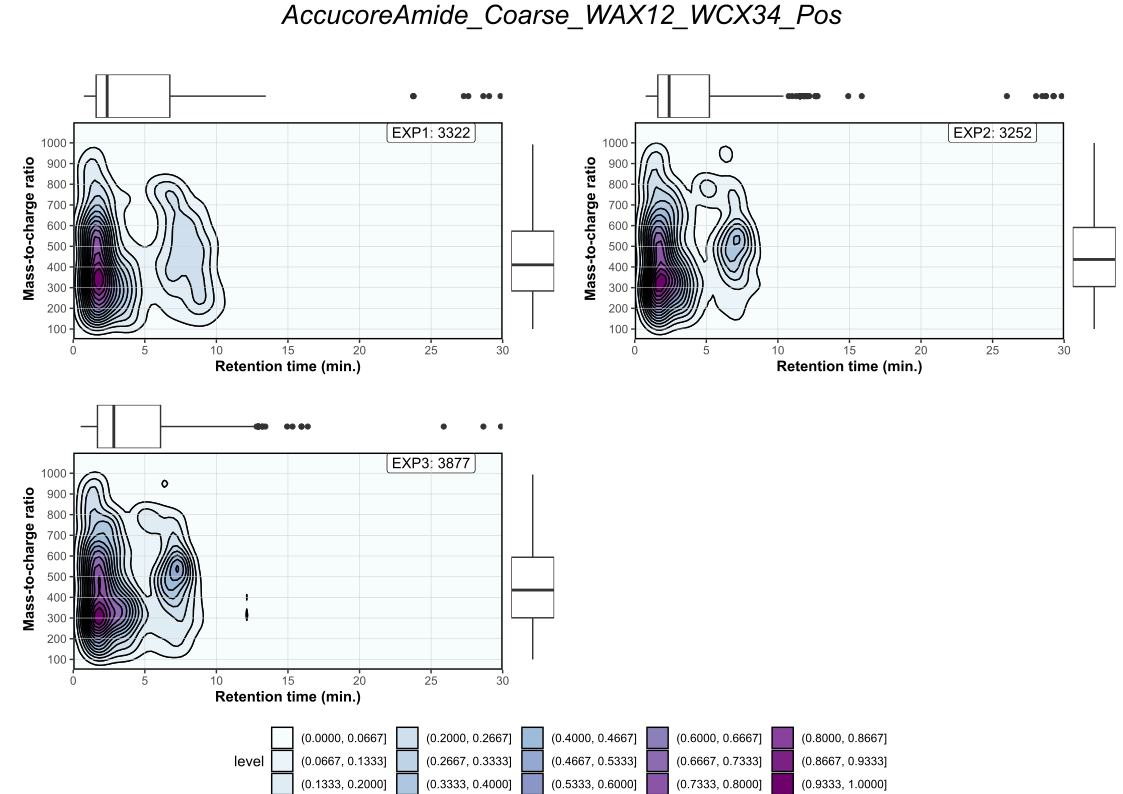


***Figure S17:*** *2-dimensional density plot of reproducibly detectable metabolic features (RDMFs) detected through positive ionisation mode analysis of sample DAX12DCX34 using methods ‘AA-CO-1’ (****top-left****), ‘AA-CO-2’ (****top-right****) and ‘AA-CO-3 (****bottom-left****) during phase one of Accucore Amide-based HILIC-HRMS(/MS) method optimisation. Text boxes in the upper-right corner of each subplot indicate the total RDMFs.*

#### Phase two

Resuspension of dried SPE fractions proved a considerable challenge when establishing an effective HILIC-HRMS(/MS) method for DMA. Indeed, during the first phase of HILIC-HRMS(/MS) method optimisation, SPE fraction resuspension required multiple bursts in an ultrasonic bath, interspersed by vigorous vortex mixing, to achieve effective dissolution of the dried pellets. The high salt content of dried SPE fractions, coupled with the potentially poor solubility of ‘polar’ metabolites in 90% v/v ice-cold acetonitrile:aqueous buffer, were hypothesised as major contributory factors in this regard.

Phase two of HILIC-HRMS(/MS) method optimisation sought to improve the efficiency (i.e. making the process faster and reducing the likelihood of promoting metabolite degradation) and effectiveness (i.e. ensuring as many metabolites as possible are resuspended) of the procedures used for SPE fraction resuspension, prior to DMA by HILIC-HRMS(/MS). A single analytical method, adapted from method ‘AA-CO-3’ (see **Supplemental Section 1.9.3.2**), was used to evaluate three distinct resuspension processes, ‘AA-Resus-[1-3]’.

**Figure S18**, below, demonstrates the number of RDMFs recorded in samples DAX12DCX34 and DCX12DAX34 following application of the three resuspension methods tested, ‘AA-RESUS-[1-3]’. Sum totals of RDMFs across sample type and ionisation mode were: 14789 for ‘AA-Resus-1’, 10580 for ‘AA-Resus-2’, and 8947 for ‘AA-Resus-3’. Method ‘AA-Resus-1’ – a replica of the method used for fraction resuspension during the first phase of HILIC-HRMS(/MS) method development (see **Supplemental Section 1.9.3.1**) – thus permitted detection of the greatest number of RDMFs. This remained true when considering RDMFs counts for each combination of sample type or MS ionisation mode.

Distributions of RDMFs differed considerably for each of the resuspension methods, with median RDMF retention times consistently lower for ‘AA Resus-2’ and consistently greater for ‘AA-Resus-3’, as summarised in **Table S12**. Each method, therefore, appeared to demonstrate differing selectivity with respect to the types of metabolic constituents resuspended and effectively separated under HILIC conditions.

Comparison of RDMF distributions for methods ‘AA-Resus 1’ and ‘AA-Resus-3’ (see **Figures** **S19** and **S20**, below) revealed 48.5 to 62.7% fewer early-eluting (i.e. less-polar or more apolar) RDMFs – here defined as RDMFs with retention times less than 5 * LC retention factor – for ‘AA-Resus-3’. The lower volume of ACN used for ‘AA-Resus-3’ resuspensions likely underpinned this observation. For later eluting RDMFs – defined as RDMFs with retention times > 5 * LC retention factor – ‘AA-Resus-3’ yielded 42.2% and 45.3% fewer RDMFs than ‘AA-Resus-1’ from analysis of DAX12DCX34 under negative and positive ionisation conditions, respectively. Similarly, 16.4% fewer RDMFs were recorded using ‘AA-Resus-3’ compared to ‘AA-resus-1’ when analysing sample DCX12DAX34 in positive ionisation mode, though 10.2% more RDMFs were recorded during negative ionisation mode analyses of the same sample, following ‘AA-Resus-3’.

Comparison of the chromatographic distributions of RDMFs for methods ‘AA-Resus-1’ and ‘AA-Resus-2’ (see **Figures S19** and **S20**, below), revealed 10.7 to 26.5% fewer early-eluting RDMFs (i.e. retention time < 5 * LC retention factor) for ‘AA-Resus-2’. The number of later-eluting RDMFs (i.e. retention time > 5 * LC retention factor) was also reduced relative to ‘AA-Resus-1’, with 25.5-27.1% fewer recorded RDMFs during analysis of DAX12DCX34, and 43.5% (positive mode) and 56.2% (negative mode) fewer during analysis of DCX12DAX34. These results build upon the conclusions of Ruta et al. (2010) who, using a limited panel of pre-dissolved probe molecules, suggested that comparable HILIC performance could be achieved where either 80:20% v/v ACN:DMSO or 100% v/v A-phase injection solvents were employed. In the present study, resuspension of dried polar extracts using 80:20% v/v ACN:DMSO mixtures was found to be substantially less effective for sample resuspension than using 100% mobile phase A. Indeed, given that ‘AA-Resus-2’ (resuspension using 80:20% v/v ACN:DMSO) employed the same sonication and vortex mixing procedures used for ‘AA-Resus-1’, but resulted in substantially fewer and less-well distributed RDMFs across all analyses conducted, ‘AA-Resus-2’ was ultimately excluded from further consideration as a method for metabolite resuspension in the DMA workflow.

A mismatch between sample injection solvent and initial LC eluent composition is known to adversely impact chromatographic performance. Chauve et al., (2010) highlight this as ‘the most important cause of performance loss and peak distortion’ in HILIC analyses, leading to peak splitting, tailing and fronting effects, particularly for earlier-eluting peaks. Nevertheless, due to poor solubility of various polar metabolites in ice-cold organic solvent (typically >90% v/v acetonitrile for HILIC analyses), such as those contained in the SPE fractions analysed in this work, mismatches are required to ensure effective solute dissolution. Use of a plugged sample injection procedure (such as that used to good effect by Johnson et al., (2010)), permits partial dilution of aqueous injection solvent – in this work reducing aqueous content from approximately 50% to 30% v/v. Given that method ‘AA-Resus-3’ yielded distinctly fewer early-eluting peaks than ‘AA-Resus-1’, a further reduction in the aqueous composition of the sample injection plug was speculated to be of importance for effective DMA. Indeed, Heaton and McCalley (2016) concluded that >20% v/v aqueous content should be avoided in the sample injection plug of HILIC-based separations, if early eluting peak shape is of importance. Hence, with a view to further improving the effectiveness of HILIC-HRMS(/MS) for DMA, methods ‘AA-Resus-3’ was carried forward for further optimisation in phase three of HILIC-HRMS(/MS) method optimisation, wherein method ‘AA-Resus-1’ was used to benchmark performance.

***Figure S18:*** *Counts of reproducibly detectable metabolic features (RDMFs) detected in both positive (‘pos’) and negative (‘neg’) ionisation modes using methods ‘AA-RESUS-[1-3]’ during phase two of Accucore Amide-based HILIC-HRMS(/MS) method optimisation.*


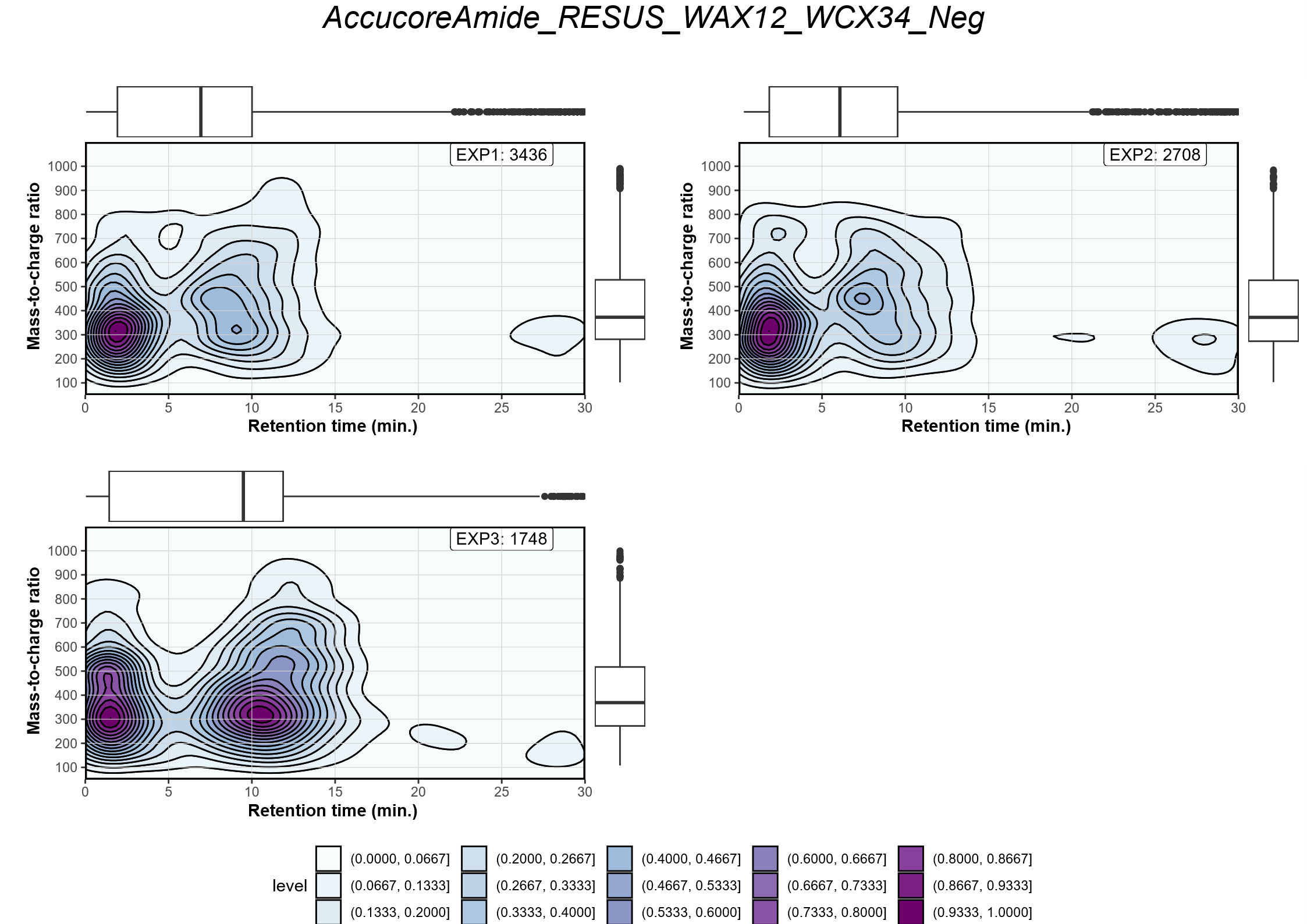


***Figure S19:*** *2-dimensional density plot of reproducibly detectable metabolic features (RDMFs) detected through negative ionisation mode analysis of sample DAX12DCX34 using methods ‘AA-Resus-1’ (****top-left****), ‘AA-Resus-2’ (****top-right****) and ‘AA-Resus-3 (****bottom-left****), during phase two of Accucore Amide-based HILIC-HRMS(/MS) method optimisation. Text boxes in the upper-right corner of each subplot indicate the total RDMFs.*


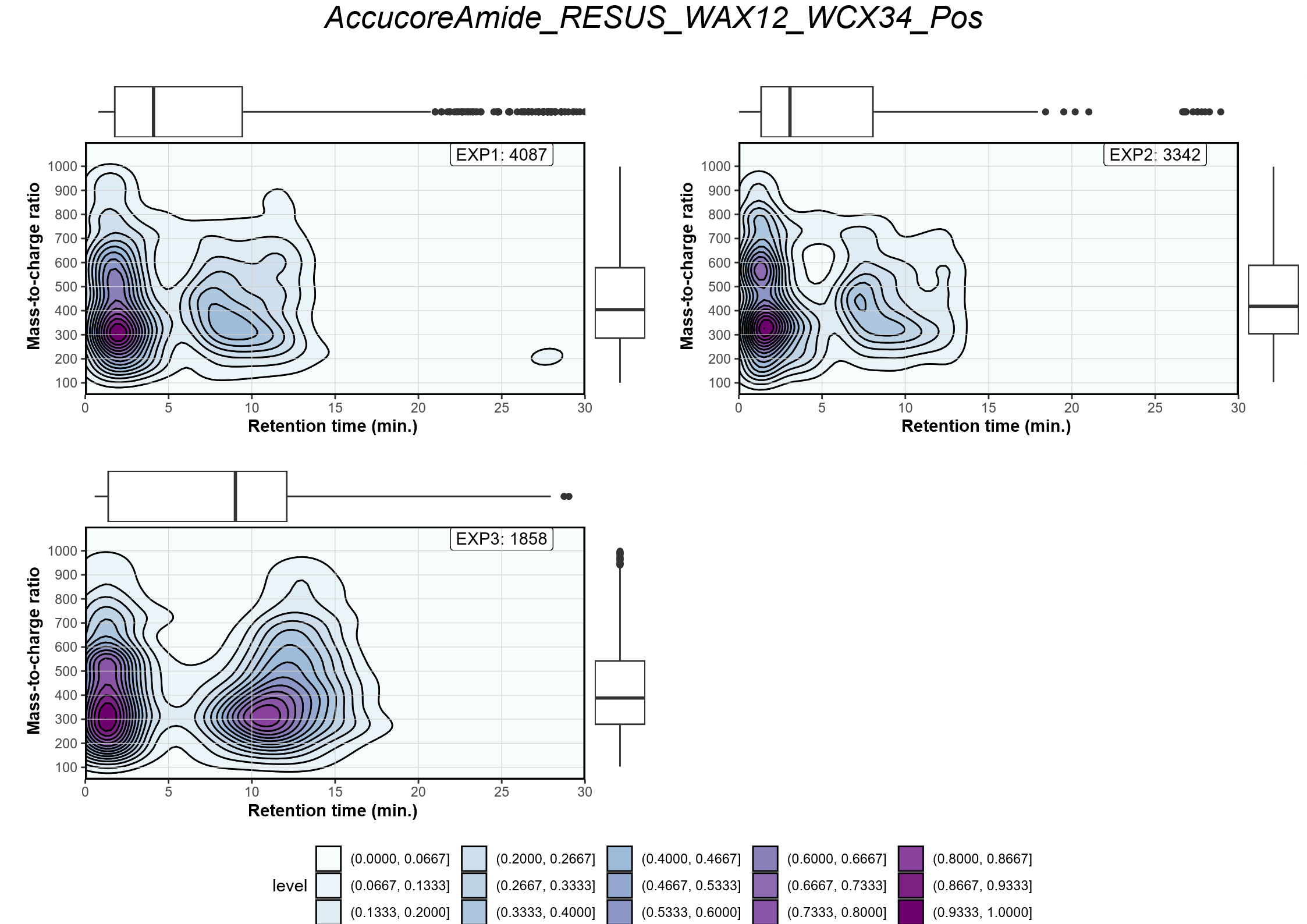


***Figure S20:*** *2-dimensional density plot of reproducibly detectable metabolic features (RDMFs) detected through positive ionisation mode analysis of sample DAX12DCX34 using methods ‘AA-Resus-1’ (****top-left****), ‘AA-Resus-2’ (****top-right****) and ‘AA-Resus-3 (****bottom-left****), during phase two of Accucore Amide-based HILIC-HRMS(/MS) method optimisation. Text boxes in the upper-right corner of each subplot indicate the total RDMFs.*

#### Phase three

Phase three of HILIC-HRMS(/MS) method optimisation sought to further improve the procedures used for resuspension of polar extracts and associated SPE fractions in the DMA workflow, to maximise the quantity and quality of resulting HILIC-HRMS(/MS) data. Two distinct resuspension methods were accordingly trialled. Method ‘AA-Resus-1’ used mobile phase A as resuspension solvent, whereas method ‘AA-Resus-4’ used 20 µL 100 mM ammonium acetate buffer (aq., pH unadjusted) followed by 80 µL acetonitrile.

Application of ‘AA-Resus-4’ to the resuspension of samples DAX12DCX34 and DCX12DAX34 resulted in formation of biphasic solutions. Neither warming these solutions to room temperature, nor increasing their acetonitrile or water proportions, served to disrupt their biphasic state. Spiking of 20 µL ice-cold methanol did however dissipate the biphase, as evidenced by the visibly uniform distribution of coloured metabolome constituents (presumably pigments, such as carotenoids) throughout resulting monophasic solutions. Method ‘AA-Resus-4’ thereby yielded solutions of 20% greater volume than ‘AA-Resus-1’, i.e. metabolites were potentially more diluted.

Based solely on the number of RDMFs recorded using resuspension methods ‘AA-Resus-1’ and ‘AA-Resus-4’, as summarised in **Figure S21,** below, neither resuspension procedure was found to be distinctly more performant for HILIC-HRMS(/MS)-based DMA. Sums of RDMFs counts across each sample type and ionisation mode combination were similar for each method, yielding 6741 and 7001 RDMFs, respectively. Method ‘AA-Resus-1’ did however outperform method ‘AA-Resus-4’ in terms of the number of RDMFs recorded in sample DAX12DCX34 under both positive and negative ionisation conditions, though the opposite was true for analysis of sample DCX12DAX34.

Inspection of the chromatographic distribution of RDMFs recorded using methods ‘AA-Resus-1’ and ‘AA-Resus-4’, as demonstrated in **Figures S22** and **S23,** below, revealed striking differences between the two methods. The early-eluting region of resulting chromatograms (defined as per **Supplemental Section 2.1.2.2**; equating to < 4.4 to 4.7 minutes) contained between 50 and 67% fewer RDMFs when ‘AA-Resus-4’ was used for sample resuspension as opposed to ‘AA-Resus-1’. Conversely, the later eluting regions (> 4.4 to 4.7 minutes) contained 66.7 and 107.1% more RDMFs for sample DAX12DCX34, and 248 and 363.7% (positive and negative mode values, respectively) for sample DCX12DAX34, when ‘AA-Resus-4’ was used for sample resuspension instead of ‘AA-Resus-1’.

Given that a HILIC-HRMS(/MS) method was included in the DMA workflow to facilitate HRMS(/MS) analysis of as many (predominantly) polar metabolites as possible, resuspension method ‘AA-Resus-4’, which yielded data on more later-eluting (and thus presumably more polar) metabolites, was selected as the method of choice for sample resuspension in the DMA workflow. Those metabolites missed by method ‘AA-Resus-4’, i.e. those detected in the early elution window of the HILIC-HRMS(/MS) method, were assumed to have more apolar characteristics and thus likely better suited to analysis under reversed-phase conditions (such as those established in **Supplemental Sections 2.1.1.1** and **2.1.1.2**).

***Figure S21:*** *Counts of reproducibly detectable metabolic features (RDMFs) detected in both positive (‘pos’) and negative (‘neg’) ionisation modes using methods ‘AA-Resus-1’ and ‘AA-Resus-4’ during phase three of Accucore Amide-based HILIC-HRMS(/MS) method optimisation.*


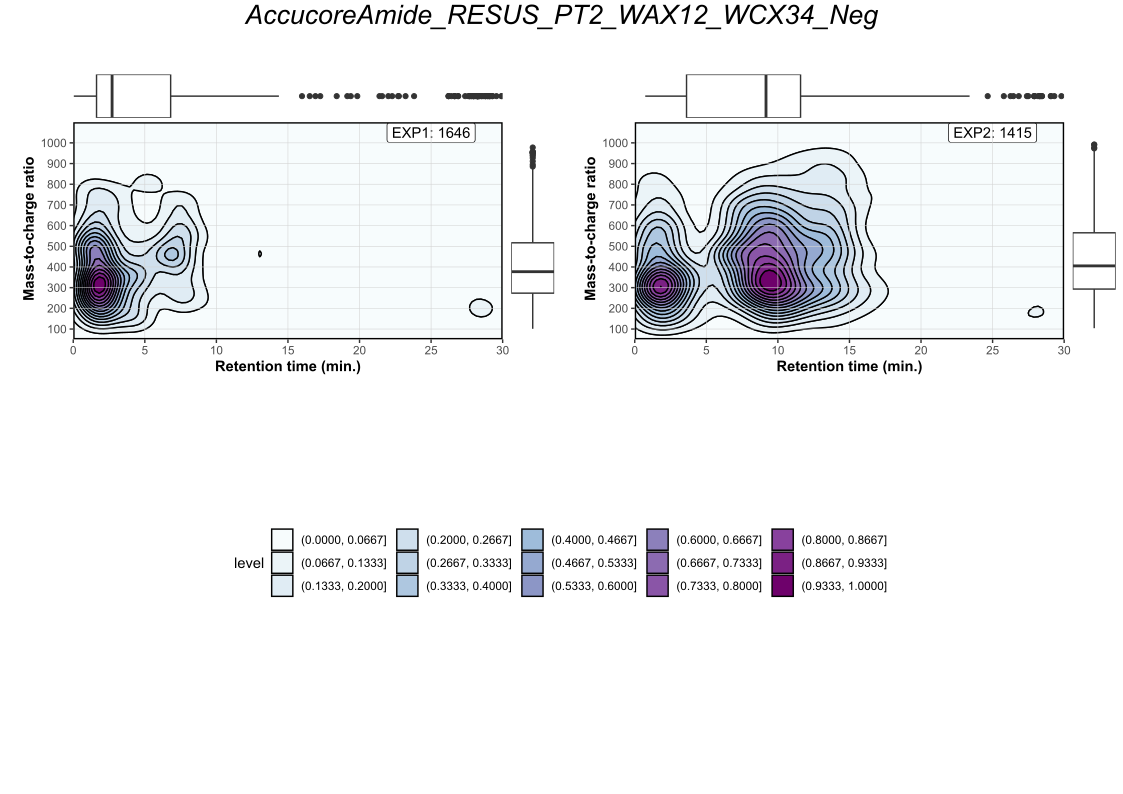


***Figure S22:*** *2-dimensional density plot of reproducibly detectable metabolic features (RDMFs) detected through negative ionisation mode analysis of sample DAX12DCX34 using methods ‘AA-Resus-1’ (****left****) and ‘AA-Resus-4’ (****right****), during phase three of Accucore Amide-based HILIC-HRMS(/MS) method optimisation. Text boxes in the upper-right corner of each subplot indicate the total RDMFs.*


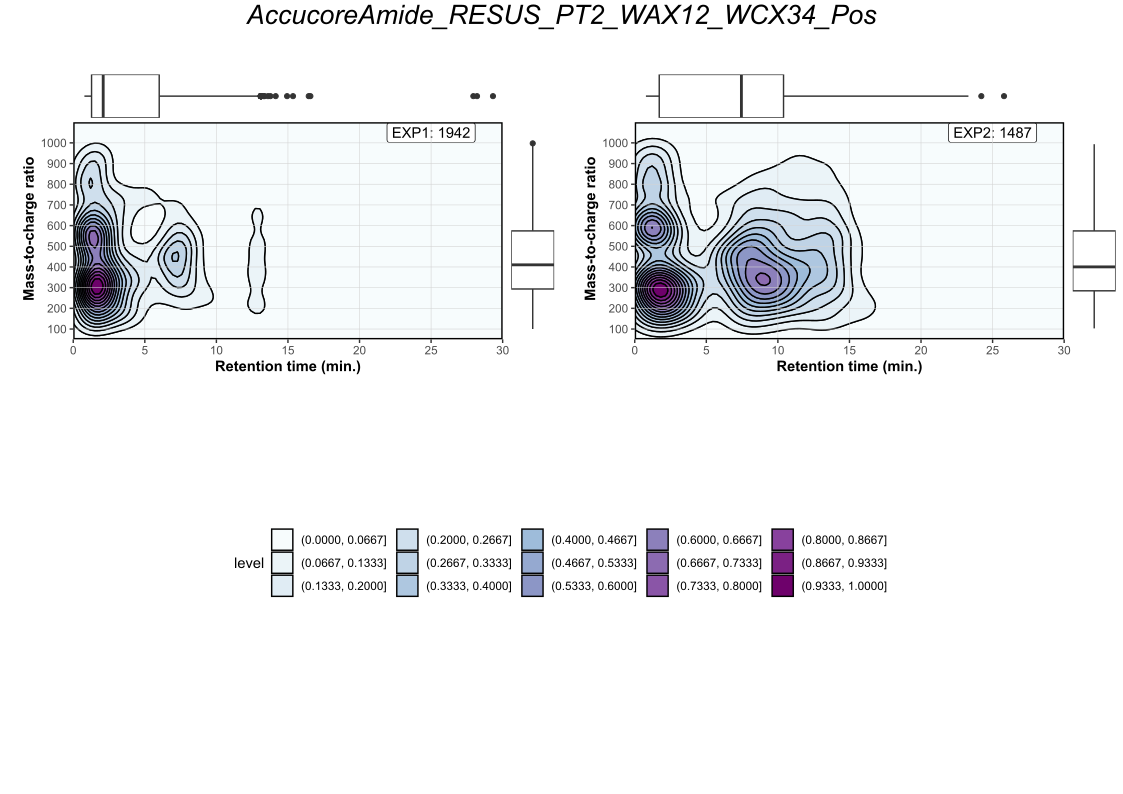


***Figure S23:*** *2-dimensional density plot of reproducibly detectable metabolic features (RDMFs) detected through positive ionisation mode analysis of sample DAX12DCX34 using methods ‘AA-Resus-1’ (****left****) and ‘AA-Resus-4’ (****right****), during phase three of Accucore Amide-based HILIC-HRMS(/MS) method optimisation. Text boxes in the upper-right corner of each subplot indicate the total RDMFs.*

#### Phase four

The fourth and final phase of optimising a HILIC-HRMS(/MS) method for DMA, involved broad reappraisal of the conditions used to effect HILIC separations. This was undertaken in response to the significant changes made to the HILIC-HRMS(/MS) sample resuspension protocol in prior optimisation steps, which substantially altered the types of metabolites accessible to this method. Six methods were ultimately trialled, methods ‘AA-FO-[1-6]’.

Counts of RDMFs recorded using methods ‘AA-FO-[1-6]’ are summarised in **Figure S24**, below. Strikingly, analyses conducted using method ‘AA-FO-1’ – a partially modified version of the most performant HILIC-HRMS(/MS) method from earlier optimisation experiments (see **Supplemental Sections 1.9.3 and 2.1.2.1-2.1.2.3** for method details) – resulted in between 34.1 and 53.4% fewer RDMFs than method ‘AA-FO-2’. The latter was a replica of method ‘AA-FO-1’, except for injection volume having been halved and flow rate doubled. The substantially lower RDMF counts for ‘AA-FO-1’ are hypothesised to stem from use of an overly large injection volume, which can deleteriously impact HILIC performance, including through peak fronting, tailing and, in extreme cases, complete peak loss (Chauve et al., 2010; Heaton and McCalley, 2016; Ruta et al., 2010; Vorkas et al., 2015). Such effects have been proposed to arise, as in RPLC, due to the differences in relative strengths and viscosities of the injection and mobile phase solutions (Heaton and McCalley, 2016). The lower injection volume of ‘AA-FO-2’, in combination with higher flow rate, would have led to greater dilution of the injection solvent and thereby likely helped to minimise solvent mismatch effects, resulting in improved chromatographic performance. For this reason, a 5 µL injection volume and 400 µL min^-1^ flow rate were used in the final DMA HILIC-HRMS(/MS) method.

Comparison of the sum total of RDMFs recorded (across each sample type and ionisation mode) using methods ‘AA-FO-4’ and ‘AA-FO-2’, or methods ‘AA-FO-5’ and ‘AA-FO-3’, revealed that substantially more RDMFs were recorded where pH-adjusted ammonium acetate buffers (aq., pH 5.8; as in methods ‘AA-FO-4’ and ‘AA-FO-5’) were used for mobile phase preparation and sample resuspension, rather than unmodified ammoniate acetate solutions (as in methods ‘AA-FO-2’ and ‘AA-FO-3’). Sum totals were 16869 and 14993, and 14105 and 12776, respectively. Methods ‘AA-FO-4’ and ‘AA-FO-2’, and methods ‘AA-FO-5’ and ‘AA-FO-3’ were each equivalent, except for the aqueous buffer used. It is noted that methods ‘AA-FO-4’ and ‘AA-FO-5’ also required 10 µL of additional methanol to be spiked into resuspended samples to maintain a monophasic state, meaning samples were also somewhat more dilute than those analysed by methods ‘AA-FO-2’ and ‘AA-FO-3’.

Through comparison of RDMFs counts for methods ‘AA-FO-4’ and ‘AA-FO-5’, and methods ‘AA-FO-2’ and ‘AA-FO-3’, it was observed that the rate at which aqueous volume percentage increased throughout the gradient elution program impacted both the total number and distribution of RDMFs recorded. Unexpectedly, it was the higher aqueous ramp rates applied in methods ‘AA-FO-2’ and ‘AA-FO-4’, wherein mobile phase ‘B’ constituted 60% v/v water, that gave rise to the greater number of RDMFs. In agreement with expectations however, the shallower ramp rates of aqueous volume fraction in methods ‘AA-FO-3’ or ‘AA-FO-5’ resulted in wider distributions of RDMFs, as shown in **Figures** **S25** and **S26**, below. A trade off therefore existed with respect to selecting an ‘optimal’ set of parameters for HILIC separations. Fortuitously, method ‘AA-FO-6’ afforded an excellent compromise, yielding the second highest RDMF sum total across all evaluated methods (15293 RDMFs recorded across all sample types and ionisation modes) – second only to ‘AA-FO-4’ (16869 RDMFs across all sample types and ionisation mode). Methods ‘AA-FO-6’ also consistently gave rise to the widest distribution of RDMFs, as evidenced by the interquartile range of RDMF retention times (see **Table S12**). Method ‘AA-FO-6’ was therefore selected as the HILIC-HRMS(/MS) method for inclusion in the DMA analytical workflow.

***Figure S24:*** *Counts of reproducibly detectable metabolic features (RDMFs) detected in both positive (‘pos’) and negative (‘neg’) ionisation modes using methods ‘AA-FO-[1-6]’ during phase four of Accucore Amide-based HILIC-HRMS(/MS) method optimisation.*


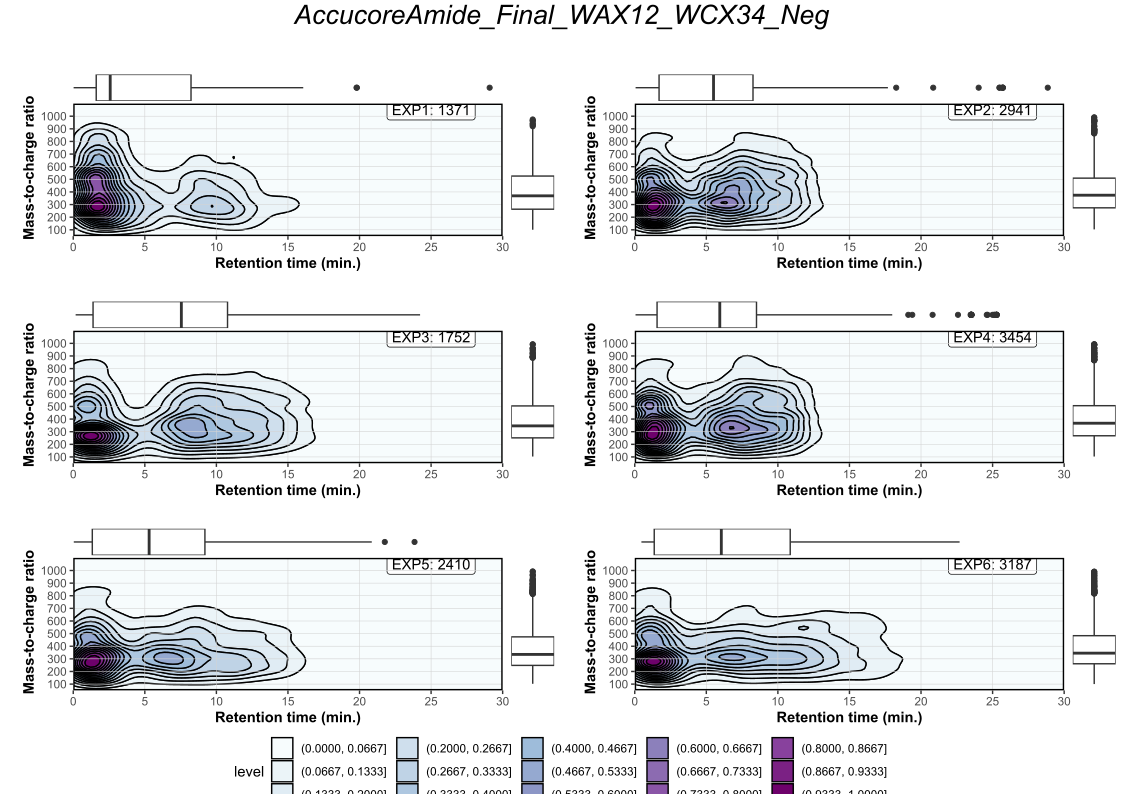


***Figure S25****: 2-dimensional density plot of reproducibly detectable metabolic features (RDMFs) detected through negative ionisation mode analysis of sample DAX12DCX34 using methods ‘AA-FO-1’ (****top-left****), ‘AA-FO-2 (****top-right****), ‘AA-FO-3’ (****centre-left****), ‘AA-FO-4’ (****centre-right****), ‘AA-FO-5’ (****bottom-left****) and ‘AA-FO-6’ (****bottom-right****) during phase four of Accucore Amide-based HILIC-HRMS(/MS) method optimisation. Text boxes in the upper-right corner of each subplot indicate the total RDMFs.*


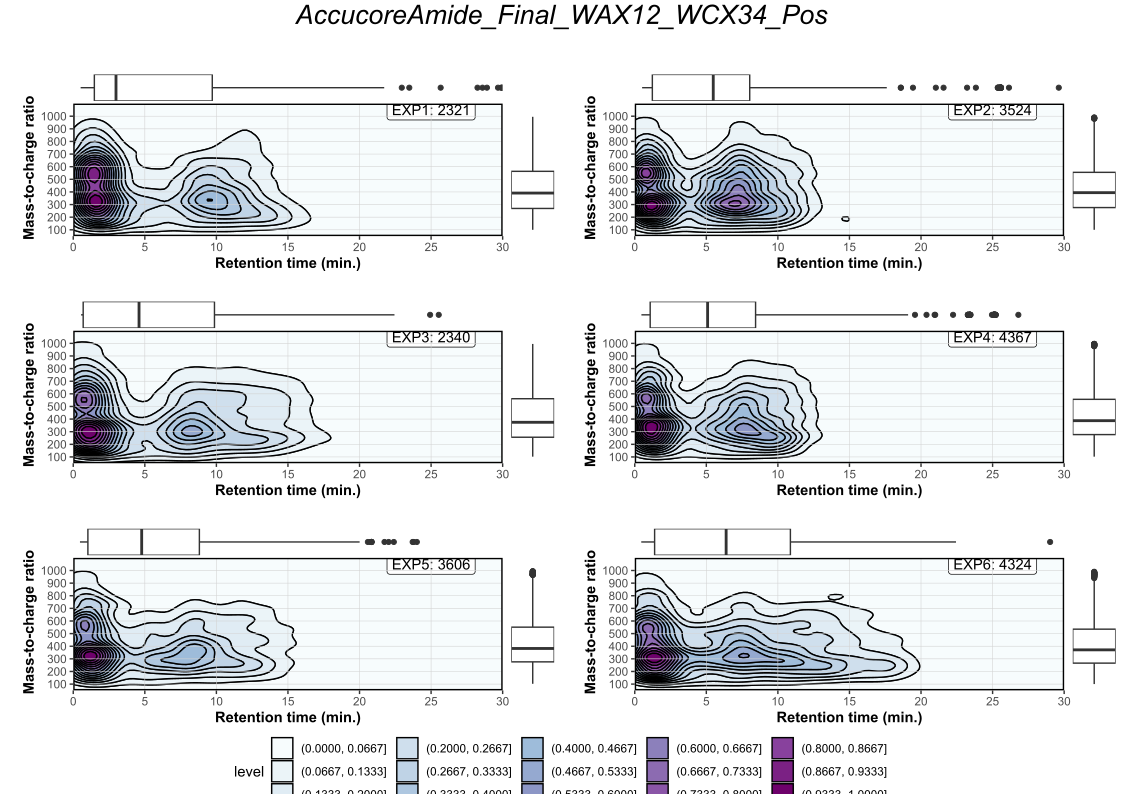


***Figure S26****: : 2-dimensional density plot of reproducibly detectable metabolic features (RDMFs) detected through positive ionisation mode analysis of sample DAX12DCX34 using methods ‘AA-FO-1’ (****top-left****), ‘AA-FO-2 (****top-right****), ‘AA-FO-3’ (****centre-left****), ‘AA-FO-4’ (****centre-right****), ‘AA-FO-5’ (****bottom-left****) and ‘AA-FO-6’ (****bottom-right****) during phase four of Accucore Amide-based HILIC-HRMS(/MS) method optimisation. Text boxes in the upper-right corner of each subplot indicate the total RDMFs.*

## Summary of all DMA of D. magna annotations

See **Supplementary Table S13** (provided in separate excel file) for a summary of all annotations for *D. magna.*


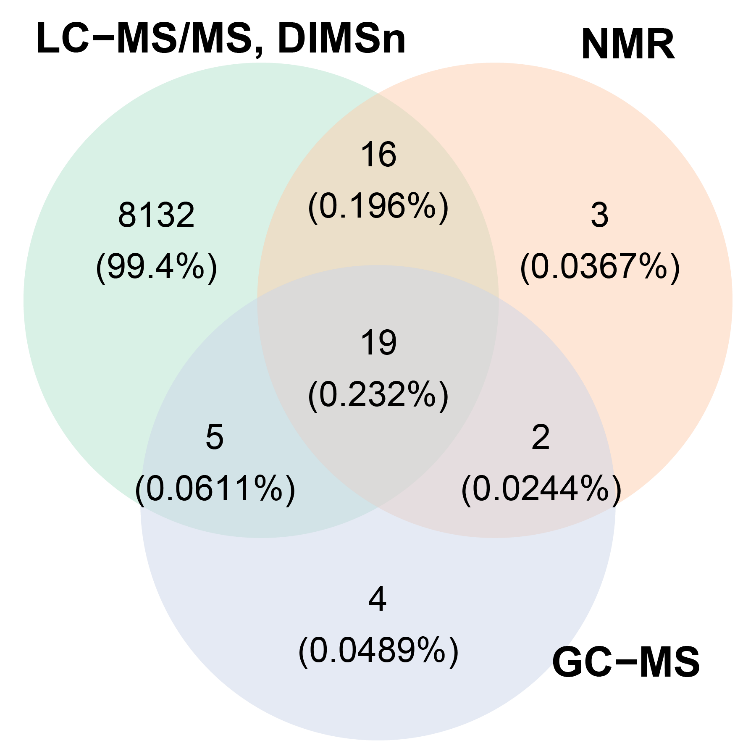


***Figure S27: Venn diagram of metabolite annotations observed for 1D- & 2D-NMR, GC-EI-HRMS and (U)HPLC-HRMS(/MS) and DI-HRMS(/MS^n^) measurement techniques***

## (U)HPLC-HRMS(/MS) and DI-HRMS(/MS^n^) derived metabolite annotations

We report 8,172 MSI level 2 annotated metabolites across all (U)HPLC-HRM(/MS) & DI-HRMS(/MS^n^) assays.

(U)HPLC-HRMS(/MS) & DI-HRMS(/MS^n^) annotations were derived from three main computational annotation approaches: spectral matching, SIRIUS CSI:FingerID and MetFrag. SIRIUS CSI:FingerID consistently provided more annotations than any other approach (5,817 unique annotations in total, 4,581 of which were unique to the approach), followed by MetFrag (1,951 unique annotations, 1,075 unique to the approach) and then spectral matching (1,880 unique annotations, 1,230 unique to the approach). The higher number of annotations observed from SIRIUS CSI:FingerID is partly down to the filtering used for this approach (choosing the top ranked annotation) whereas MetFrag and Spectral Matching enabled much more stringent filtering criteria to be applied based on the multiple scoring outputs. We note that SIRIUS CSI;FingerID now has the COSMOS score but that was not implemented within Galaxy Workflow tool at the time of analysis. Additionally, with the version of SIRIUS used, the COSMOS score was only provided for the top ranked CSI:FingerID annotation so could not be applied to all annotations derived from SIRIUS CSI:FingerID Galaxy tool. The primary reason for MetFrag not exceeding the number of annotations of the other approaches is due to the very stringent filtering criteria on the MetFrag annotations (chosen based on manual inspection of annotations observed at different thresholds of the MetFrag score) and the use of a wide range of libraries for spectral matching.

Spectral matching was performed using 3 different workflows or tools: The “Galaxy workflow” approach – where spectral matching was performed via the Galaxy workflow tool msPurity.spectralMatching applied to averaged fragmentation spectra; The “mzCloud” approach – spectral matching performed directly from the raw mass spectrometry files against the mzCloud spectral database; and the “GNPS workflow” approach – where the mzML files are searched against the GNPS public spectral databases. The “Galaxy workflow” spectral matching approach gave 1,044 unique annotations (784 unique to the approach), the GNPS workflow spectral matching approach gave 822 unique annotations (532 unique to the approach) and the mzCloud approach gave 415 unique annotations (255 unique to the approach). The Galaxy workflow and GNPS workflow were expected to have a higher number of annotations due to the larger spectral databases used (derived from various sources and instrument types) whereas the mzCloud library uses a smaller but more specific library for Thermo Scientific’s instruments, including Orbitrap mass spectrometers (i.e. the same instrument type used for the experimental analyses performed here).

Annotation to a full InChiKey is difficult using only mass spectrometry fragmentation analysis without additional chromatography investigations and can be challenging to distinguish between the stereochemistry encoded in the second component of an InChiKey. If we only consider the first component of the InChiKey (encoding the “molecular skeleton” of the structure) we observe 7,667 annotations from the (U)HPLC-MS/MS & DI-HRMS(/MS^n^) data sets.


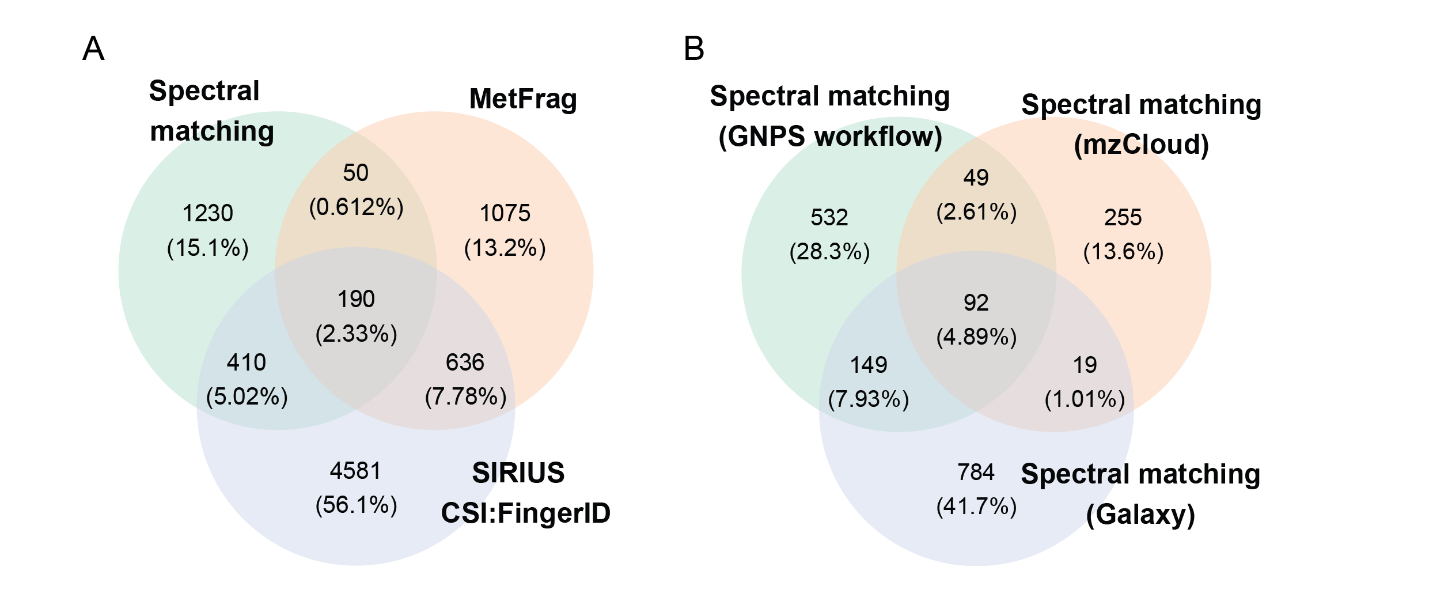


***Figure S28: Venn diagram of metabolite annotations observed across computational annotation approach used.*** *A) Comparison of spectral matching, MetFrag and SIRIUS CSI:FingerID. B) Comparison of the annotations specifically from the three spectral matching approaches used.*


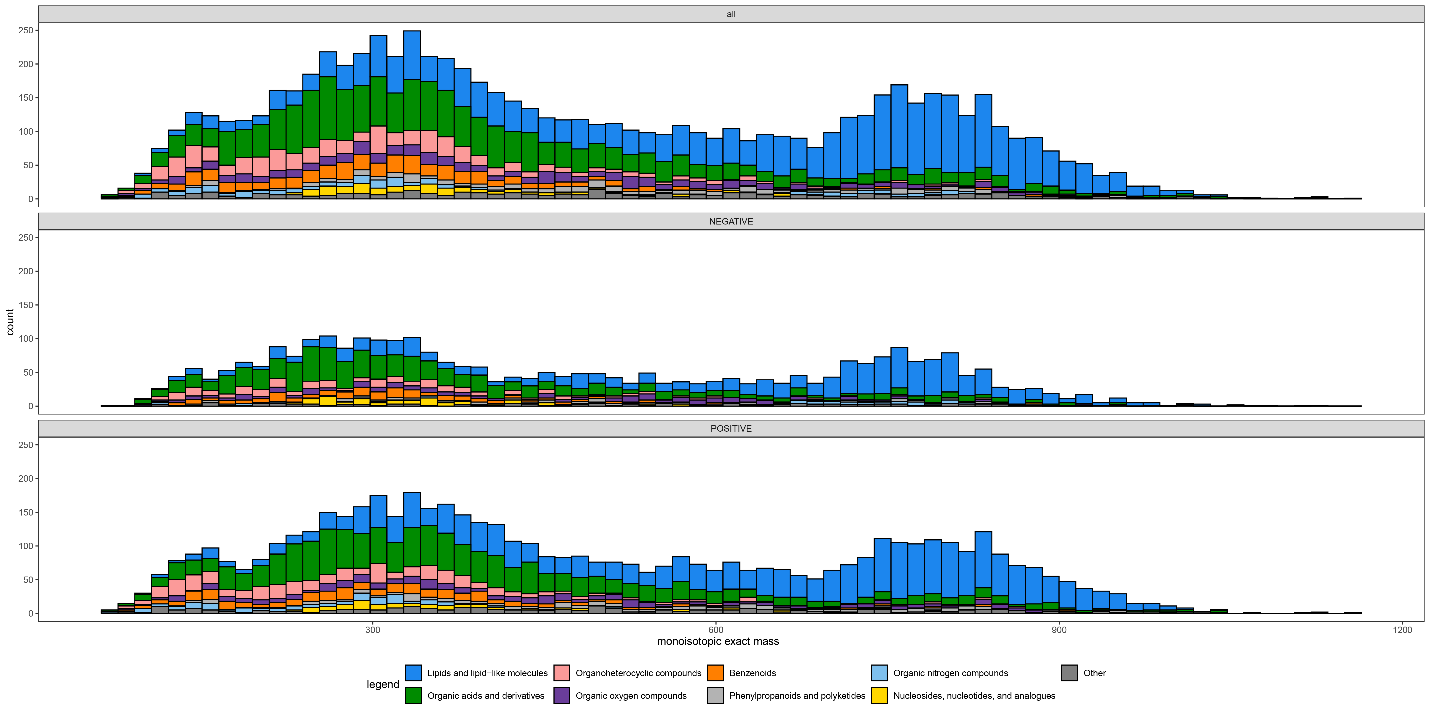


***Figure S29: Distribution of unique metabolite annotations across monoisotopic exact mass:*** *Histograms shown for positive ionisation mode, negative ionisation mode and the combined annotation of positive and negative ionisation mode. Colour represents the superclass compound classification of the annotations.*

## GC-EI-HRMS derived metabolite annotations

GC-EI-HRMS analysis of the crude polar extract and polar SPE fractions lead to the putative annotation of 31 metabolites (MSI level 2). The GC-EI-HRMS annotations included 19 organic acids and derivatives, including 15 of the 21 proteinogenic amino acids present in eukaryotes (as well as several derivatives thereof); 2 purine nucleotides; 2 pyrimidine nucleotides; 1 glycerophospholipids, 1 steroid (cholesterol), 1 amine (putrescine), 1 carbohydrate (D-glucose), 1 cyclohexanols (myo-inositol); 1 indole derivative (L-tryptophan), 1 glycoside (methyl-galactoside) and 1 inorganic compound (sulfuric acid). The 4 organic metabolites unique to the GC-EI-HRMS analysis were: Myo-inositol, 2-oxopentanoic acid, methyl-galactoside and 2-aminobutanoic acid.

**Table S14:** GC-EI-HRMS derived metabolite annotations

| **Compound** | **Quantifier ion (m/z)** | **Retention time (min.)** | **SI/Dot product score** | **SPE fractions** |
| --- | --- | --- | --- | --- |
|  |  |  |  |  |
| 2-aminobutanoic acid | 130.1047 | 7.84 | 772 | WAX[1], WCX[1], Crude |
|  |  |  |  |  |
| 2-oxopentanoic acid | 73.04683 | 10.13 | 714 | WAX[1], WAX[2], WAX[3], WAX[4], WCX[1], Crude |
|  |  |  |  |  |
| Adenosine 5'-monophosphate | 169.0679 | 17.53 | 757 | WAX[4], WCX[1], Crude |
|  |  |  |  |  |
| Asparagine | 73.04683 | 11.38 | 804 | WAX[1], WCX[1], WCX[4], Crude |
|  |  |  |  |  |
| Cholesterol | 129.073 | 18.13 | 858 | WAX[2], WCX[1], Crude |
|  |  |  |  |  |
| D-glucose | 73.04683 | 12.63 | 852 | WAX[1]. WCX[1], Crude |
|  |  |  |  |  |
| Glycerol-3-phosphate | 299.0713 | 11.87 | 830 | WAX[1], WAX[4], WCX[1], Crude |
|  |  |  |  |  |
| Glycine | 174.113 | 9.01 | 827 | WAX[1], WCX[1], Crude |
|  |  |  |  |  |
|  | 102.0735 | 7.33 | 798 |  |
|  |  |  |  |  |
| Inosine | 217.1074 | 15.65 | 801 | WAX[1], WCX[1], Crude |
|  |  |  |  |  |
| Lactic acid | 149.0449 | 6.61 | 786 | WAX[1], WAX[2], WAX[3], WCX[1], Crude |
|  |  |  |  |  |
| L-alanine | 116.089 | 7.12 | 849 | WAX[1], WCX[1], Crude |
|  |  |  |  |  |
| L-cystine | 218.1025 | 14.52 | 718 | WAX[1], WCX[1], Crude |
|  |  |  |  |  |
| L-glutamic acid | 246.1339 | 11.08 | 817 | WAX[1], WAX[3], WCX[1], Crude |
|  |  |  |  |  |
| L-glutamine | 156.0837 | 11.97 | 786 | WAX[1], WCX[1], Crude |
|  |  |  |  |  |
| L-isoleucine | 158.136 | 8.89 | 858 | WAX[1], WCX[1], Crude |
|  |  |  |  |  |
| L-leucine | 158.1359 | 8.72 | 735 | WAX[1], WCX[1], Crude |
|  |  |  |  |  |
| L-threonine | 73.04683 | 9.57 | 883 | WAX[1], WCX[1], Crude |
|  |  |  |  |  |
| L-tryptophan | 79.05426 | 14.2 | 762 | WAX[1], WCX[1], WCX[2], Crude |
|  |  |  |  |  |
| L-tyrosine | 218.1026 | 12.86 | 857 | WAX[1], WCX[1], Crude |
|  |  |  |  |  |
| L-valine | 144.1203 | 8.24 | 869 | WAX[1], WCX[1], Crude |
|  |  |  |  |  |
| Lysine | 174.113 | 12.77 | 723 | WAX[1], WCX[1], WCX[3], Crude |
|  |  |  |  |  |
| L-5-oxoproline | 156.0839 | 10.51 | 840 | WAX[1], WAX[3], WCX[1], Crude |
|  |  |  |  |  |
| L-proline | 142.1046 | 8.94 | 858 | WAX[1], WCX[1], Crude |
|  |  |  |  |  |
| Methyl-galactoside | 204.0997 | 12.54 | 744 | WAX[1], WCX[1], Crude |
|  |  |  |  |  |
| Myo-inositol | 217.1074 | 13.62 | 790 | WAX[1], WCX[1], Crude |
|  |  |  |  |  |
| Phenylalanine | 218.1021 | 11.17 | 881 | WAX[1], WCX[1], Crude |
|  |  |  |  |  |
| Putrescine | 174.1129 | 11.41 | 839 | WAX[1], WAX[2], WCX[1], WCX[4], Crude |
|  |  |  |  |  |
| Sulfuric acid | 147.0657 | 7.71 | 796 | WAX[4], WCX[1], Crude |
|  |  |  |  |  |
| Serine | 116.0528 | 8.59 | 726 | WAX[1], WCX[1], Crude |
|  |  |  |  |  |
|  | 204.1236 | 9.38 | 851 |  |
|  |  |  |  |  |
| Uridine | 73.04683 | 12.27 | 833 | WAX[1], WAX[4], WCX[1], Crude |
|  |  |  |  |  |
| Uridine 5'-monophosphate | 169.0679 | 16.67 | 714 | WAX[4], WCX[1], Crude |
|  |  |  |  |  |

## NMR derived metabolite annotations

1D and 2D NMR analysis of the crude polar extract and polar SPE fractions lead to the identification of 39 metabolites (MSI level 1) and putative annotation of 2 metabolites. The NMR annotations included 24 organic acids and derivatives; 4 pyrimidine nucleotides; 2 purine nucleosides; 3 carbohydrates; 4 organic nitrogen compounds; 1 imidazopyrimidine (hypoxanthine); 1 indole derivative (L-tryptophan); 1 glycerophosphocholine (GlyceroPhosphoCholine - GPCho) and 1 benzenoid (diaminopropane) were observed. The 3 metabolites unique to the NMR analysis were: inosinic acid (IMP), anserine and diaminopropane.

**Table S15:** NMR derived metabolite annotations

| Peak | Metabolites | Group | δ 1H  (ppm) | Multiplicity: J (Hz) | Assignment data | Fraction(s) |
| --- | --- | --- | --- | --- | --- | --- |
| 1 | L-Alanine | β-CH_3_ | 1.48 | d | ^1^H, HSQC (18.99), TOCSY (3.79) | WCX1, WAX1 |
|  |  | α-CH | 3.79 | q | ^1^H, HSQC (53.38), TOCSY (1.48) |  |
| 2 | Anserine | β-CH_2_(Ala) | 2.69 | m | ^1^H, JRES, TOCSY (3.20) | WCX2 |
|  |  | β-CH_2_ (His) | 3.05 | dd | ^1^H, JRES, TOCSY (3.20, 4.48, 7.11) |  |
|  |  | α-CH_2_(Ala) | 3.20 | m | ^1^H, JRES, TOCSY (2.68, 4.48, 7.11) |  |
|  |  | N-CH_3_ | 3.78 | s | ^1^H, JRES |  |
|  |  | α-CH(His) | 4.48 | dd | ^1^H, JRES, TOCSY (ND*) |  |
|  |  | CH-5(His) | 7.11 | d | ^1^H, JRES, TOCSY (3.05, 3.20, 8.28) |  |
|  |  | CH-2(His) | 8.28 | d | ^1^H, JRES, TOCSY (7.11) |  |
| 3 | L-Arginine/Phosphoarginine | γ-CH_2_ | 1.68 | m | ^1^H, HSQC (26.40), TOCSY (1.92, 3.25) | WCX1, WCX2, WCX3, WCX4 ,WAX1, WAX4 |
|  |  | β-CH_2_ | 1.92 | m | ^1^H, HSQC (30.40), TOCSY (1.68, 3.25, 3.76) |  |
|  |  | δ-CH_2_ | 3.25 | t | ^1^H, HSQC (43.37), TOCSY (1.68, 1.92, 3.76) |  |
|  |  | α-CH | 3.76 | t | ^1^H, HSQC (57.21), TOCSY (1.68, 1.92, 3.25) |  |
| 4 | L-Asparagine | β-CH_2_ | 2.88 | m | ^1^H, HSQC (37.36), TOCSY (2.95, 4.01) | WCX1, WAX1 |
|  |  | β’-CH_2_ | 2.95 | m | ^1^H, HSQC (37.43) , TOCSY (2.88, 4.01) |  |
|  |  | α-CH | 4.01 | dd | ^1^H, HSQC (54.16), TOCSY (2.88, 2.95) |  |
| 5 | L-Aspartic acid | β-CH_2_ | 2.69 | dd | ^1^H, TOCSY (2.78, 3.90) | WCX1, WAX3 |
|  |  | β’-CH_2_ | 2.78 | dd | ^1^H, TOCSY (2.69, 3.90) |  |
|  |  | α-CH | 3.90 | dd | ^1^H, TOCSY (2.69, 2.78) |  |
| 6 | Betaine | CH_3_ | 3.26 | s | ^1^H, HSQC (56.09) | WCX1, WAX1 |
|  |  | CH_2_ | 3.89 | s | ^1^H, HSQC (68.72) |  |
| 7 | Choline | CH_3_ | 3.22 | s | ^1^H, HSQC (56.66) | WCX1, WCX4, WAX1 |
|  |  | β-CH_2_ | 3.51 | dd | ^1^H, HSQC (70.16), TOCSY (4.06) |  |
|  |  | α-CH_2_ | 4.06 | ddd | ^1^H, HSQC (58.52), TOCSY (3.51) |  |
| 8 | L-Cystine | CH_2_ | 3.20 | dd | ^1^H, HSQC (40.56), TOCSY (3.39, 4.12) | WCX1, WAX1 |
|  |  | CH_2_ | 3.39 | dd | ^1^H, HSQC (40.71), TOCSY (3.20, 4.12) |  |
|  |  | CH | 4.12 | dd | ^1^H, HSQC (56.19), TOCSY (3.20, 3.39) |  |
| 9 | Diaminopropane | β-CH_2_ | 2.06 | m | ^1^H, HSQC (27.86), TOCSY (3.11) | WCX3, WCX4, WAX1 |
|  |  | α-CH_2_ | 3.11 | t | ^1^H, HSQC (39.59), TOCSY (2.06) |  |
| 10 | Fatty acids  (FA) | CH_3_ | 0.90 | t | ^1^H, TOCSY (1.30) | WCX1, WCX2, WCX3, WCX4, WAX1, WAX2, WAX3, WAX4 |
|  |  | (n)CH_2_ | 1.27 | Broad | ^1^H, TOCSY (1.58, 2.25) |  |
|  |  | (1)CH_2_ | 1.58 | Broad | ^1^H, TOCSY (1.27, 2.25) |  |
|  |  | (2)CH_2_ | 2.25 | Broad | ^1^H, TOCSY (1.27, 1.58) |  |
| 11 | γ-AminoButyric Acid  (GABA) | β-CH_2_ | 1.89 | m | ^1^H, JRES, spiking, TOCSY (2.29, 3.02) | WAX1 |
|  |  | α-CH_2_ | 2.29 | t | ^1^H, JRES, spiking, TOCSY (1.89, 3.02) |  |
|  |  | γ-CH_2_ | 3.02 | t | ^1^H, JRES, spiking, TOCSY (1.89, 2.29) |  |
| 12 | α-Glucose | CH-4 | 3.40 | m | ^1^H, HSQC (72.29), TOCSY | WCX1, WAX1 |
|  |  | CH-2 | 3.54 | dd | ^1^H, HSQC(74.26), TOCSY |  |
|  |  | CH-3 | 3.72 | m | ^1^H, HSQC (75.7), TOCSY |  |
|  |  | CH-6;  CH’-6 | 3.83 | m | ^1^H, HSQC (63.45), TOCSY |  |
|  |  | CH-5 | 3.83 | m | ^1^H, HSQC (74.13), TOCSY |  |
|  |  | CH-1 | 5.23 | d | ^1^H, HSQC (94.80), TOCSY |  |
| 13 | β-Glucose | CH-2 | 3.24 | dd | ^1^H, HSQC (76.98), TOCSY | WCX1, WAX1 |
|  |  | CH-4 | 3.40 | m | ^1^H, HSQC (72.29), TOCSY |  |
|  |  | CH-3; CH-5 | 3.47 | m | ^1^H,HSQC (78.61), TOCSY |  |
|  |  | CH-6 | 3.72 | m | ^1^H, HSQC (63.38), TOCSY |  |
|  |  | CH’-6 | 3.89 | dd | ^1^H, HSQC (63.42), TOCSY |  |
|  |  | CH-1 | 4.64 | d | ^1^H, HSQC (98.64), TOCSY(ND*) |  |
| 14 | L-Glutamic acid | β-CH_2_ | 2.06 | m | ^1^H, TOCSY (2.36, 3.77) | WCX1, WAX1, WAX3 |
|  |  | β’-CH_2_ | 2.14 | m | ^1^H, TOCSY (2.36, 3.77) |  |
|  |  | γ-CH_2_ | 2.36 | m | ^1^H, HSQC (36.12), TOCSY (2.10, 3.77) |  |
|  |  | α-CH | 3.77 | dd | ^1^H, HSQC (57.53), TOCSY (2.10, 2.36) |  |
| 15 | L-Glutamine | β-CH_2_ | 2.14 | m | ^1^H, HSQC (29.15), TOCSY (2.44, 3.77) | WCX1, WAX1 |
|  |  | γ-CH_2_ | 2.44 | m | ^1^H, HSQC (33.52), TOCSY (2.14, 3.77) |  |
|  |  | α-CH | 3.77 | t | ^1^H, HSQC (57.28), TOCSY (2.14, 2.44) |  |
| 16 | Glycerol | CH_2_ | 3.57 | m | ^1^H, HSQC (65.41), TOCSY (3.66, 3.79) | WCX1, WAX1 |
|  |  | CH_2_’ | 3.66 | m | ^1^H, HSQC (65.26), TOCSY (3.57, 3.79) |  |
|  |  | CH | 3.79 | tt | ^1^H, HSQC (74.95), TOCSY (3.57, 3.66) |  |
| 17 | GlyceroPhosphoCholine  (GPCho) | CH_3_ | 3.22 | s | ^1^H, HSQC (56.66), | WCX1, WAX1 |
|  |  | CH_2_ | 3.63 | m | ^1^H, HSQC (64.63), TOCSY (3.91, 4.33) |  |
|  |  | CH ; CH_2_ | 3.91 | m | ^1^H, HSQC (69.16), TOCSY (3.63) |  |
|  |  | CH_2_ | 4.33 | m | ^1^H, HSQC (62.13), TOCSY (3.63) |  |
| 18 | L-Glycine | α-CH_2_ | 3.56 | s | ^1^H, HSQC (44.31) | WCX1, WAX1 |
| 19 | L-Histidine | β-CH_2_ | 3.18 | dd | ^1^H, HSQC (30.24), TOCSY (3.26, 4.01) | WCX1, WAX1 |
|  |  | β-CH_2_ | 3.26 | dd | ^1^H, HSQC (30.24), TOCSY (3.18, 4.01) |  |
|  |  | α-CH | 4.01 | dd | ^1^H, HSQC (57.44), TOCSY (3.18, 3.26, 7.11 ) |  |
|  |  | δ-CH | 7.11 | d | ^1^H, HSQC (119.98), TOCSY (4.01) |  |
|  |  | ε-CH | 7.93 | d | ^1^H, HSQC (138.41) |  |
| 20 | Hypoxanthine | CH | 7.96 | s | ^1^H, HSQC (144.51) | WCX1 |
|  |  | CH | 8.11 | s | ^1^H, HSQC (140.15) |  |
| 21 | L-Isoleucine | δ-CH_3_ | 0.93 | t | ^1^H, HSQC (13.68), TOCSY(1.01, 1.47, 1.98) | WCX1, WAX1 |
|  |  | γ-CH_3_ | 1.01 | d | ^1^H, HSQC (17.27), TOCSY(0.93, 1.47, 1.98) |  |
|  |  | γ-CH_2_ | 1.47 | m | ^1^H, HSQC (27.11, TOCSY(0.93, 1.01, 1.98) |  |
|  |  | β-CH | 1.98 | m | ^1^H, HSQC (38.68), TOCSY (0.93, 1.01, 1.47) |  |
|  |  | α-CH | 3.68 | d | ^1^H, HSQC (62.44), TOCSY(1.01, 1.47, 1.98) |  |
| 22 | Inosine | CH_2_ | 3.84 | dd | ^1^H, HSQC (ND), TOCSY(4.26, 4.43) | WCX1, WAX1 |
|  |  | CH_2_ | 3.91 | dd | ^1^H, HSQC (ND), TOCSY(4.26, 4.43) |  |
|  |  | CH | 4.26 | dd | ^1^H, HSQC (88.39), TOCSY(3.91, 4.43, 4.8, 6.06) |  |
|  |  | CH | 4.43 | dd | ^1^H, HSQC (73.11), TOCSY(3.91, 4.26, 4.8, 6.06) |  |
|  |  | CH | 4.8* | s | ^1^H, HSQC (ND), TOCSY(ND) |  |
|  |  | CH | 6.06 | d | ^1^H, HSQC (91.05), TOCSY(4.26, 4.43, 4.8) |  |
|  |  | CH | 8.19 | s | ^1^H, HSQC (ND), TOCSY |  |
|  |  | CH | 8.31 | s | ^1^H, HSQC (142.94) |  |
| 23 | Inosinic acid (IMP) | CH_2_ | 4.02 | m | ^1^H, HSQC (66.35), TOCSY (4.37, 4.51) | WCX1, WAX1, WAX4 |
|  |  | CH | 4.37 | m | ^1^H, HSQC (87.31), TOCSY (4.02, 4.51) |  |
|  |  | CH | 4.51 | m | ^1^H, HSQC (73.37), TOCSY (ND*) |  |
|  |  | CH | 6.15 | d | ^1^H, HSQC (89.80), TOCSY (4.51) |  |
|  |  | CH | 8.24 | s | ^1^H, HSQC (149.20) |  |
|  |  | CH | 8.55 | s | ^1^H, HSQC (142.72) |  |
| 24 | L-Lactic acid | CH_3_ | 1.33 | d | ^1^H, HSQC (22.98), TOCSY (4.11) | WCX1, WAX1, WAX2, WAX3 |
|  |  | CH | 4.11 | q | ^1^H, HSQC (71.31), TOCSY (1.33) |  |
| 25 | L-Leucine | δ-CH_3_ | 0.95 | t | ^1^H, HSQC (23.83), TOCSY (1.71, 3.75) | WCX1, WAX1 |
|  |  | δ-CH_3_ | 0.98 | t | ^1^H, HSQC (24.77), TOCSY (1.71, 3.75) |  |
|  |  | γ-CH | 1.69 | m | ^1^H, HSQC (42.59), TOCSY (0.97, 3.75) |  |
|  |  | β-CH_2_ | 1.73 | m | ^1^H, HSQC (42.59), TOCSY (0.97, 3.75) |  |
|  |  | α-CH | 3.75 | m | ^1^H, HSQC (56.34), TOCSY (0.97, 1.71) |  |
| 26 | L-Lysine | γ-CH_2_ | 1.47 | m | ^1^H, HSQC(24.15), TOCSY (1.72, 1.91, 3.02, 3.77 ) | WCX1, WCX2, WCX3, WAX1 |
|  |  | δ-CH_2_ | 1.72 | m | ^1^H, HSQC (29.30), TOCSY (1.47, 1.91, 3.02 ) |  |
|  |  | β-CH_2_ | 1.91 | m | ^1^H, HSQC (32.75), TOCSY (1.47, 1.72, 3.77 ) |  |
|  |  | ε-CH_2_ | 3.02 | t | ^1^H, HSQC (42.12), TOCSY (1.47, 1.72, 1.91) |  |
|  |  | α-CH | 3.77 | t | ^1^H, HSQC (57.28), TOCSY (1.47,1.72, 1.91 ) |  |
| 27 | L-Methionine | CH_3_-S | 2.12 | s | ^1^H, HSQC (32.76) | WCX1 |
|  |  | β-CH_2_ | 2.15 | m | ^1^H, TOCSY (2.64, 3.86) |  |
|  |  | δ-CH_2_ | 2.64 | t | ^1^H, HSQC (31.49), TOCSY (2.15, 3.86) |  |
|  |  | α-CH | 3.86 | dd | ^1^H, HSQC (56.81), TOCSY (2.64, 2.15) |  |
| 28 | L-Ornithine | γ-CH_2_ | 1.81 | m | ^1^H, HSQC (ND), TOCSY (1.93, 3.05, 3.77), spiking | WAX1 |
|  |  | β-CH_2_ | 1.93 | m | ^1^H, HSQC (30.43), TOCSY (1.81, 3.05, 3.77), spiking |  |
|  |  | δ-CH_2_ | 3.05 | t | ^1^H, HSQC (41.84), TOCSY (1.81, 1.93, 3.77), spiking |  |
|  |  | α-CH | 3.77 | t | ^1^H, HSQC (57.28), TOCSY (1.83, 1.93, 3.05), spiking |  |
| 29 | L-Phenylalanine | β-CH_2_ | 3.14 | m | ^1^H, HSQC (39.15), TOCSY (4.00 ) | WCX1, WAX1, WAX3 |
|  |  | α-CH | 4.00 | dd | ^1^H, HSQC (58.85), TOCSY (3.14) |  |
|  |  | δ-CH | 7.33 | d | ^1^H, HSQC (132.16), TOCSY (7.38, 7.43) |  |
|  |  | ζ-CH | 7.38 | m | ^1^H, HSQC (130.44), TOCSY (7.33, 7.43 ) |  |
|  |  | ε-CH | 7.43 | m | ^1^H, HSQC (132.00), TOCSY (7.33, 7.38) |  |
| 30 | PhosphorylCholine  (PCho) | CH_3_ | 3.22 | s | ^1^H, HSQC (39.15) | WCX1, WAX1, WAX4 |
|  |  | β-CH_2_ | 3.59 | t | ^1^H, HSQC (69.32), TOCSY (4.18 ) |  |
|  |  | α-CH_2_ | 4.18 | ddd | ^1^H, HSQC (60.88), TOCSY (3.59 ) |  |
| 31 | Putrescine | β-CH_2_ | 1.73 | m | ^1^H, HSQC (26.80) , TOCSY (3.03) | WCX1, WAX1 |
|  |  | α-CH_2_ | 3.03 | t | ^1^H, HSQC (41.81) , TOCSY (1.73) |  |
| 32 | L-Serine | α-CH | 3.84 | dd | ^1^H, HSQC (59.16), TOCSY (3.96) | WCX1, WAX1 |
|  |  | β-CH_2_ | 3.96 | m | ^1^H, HSQC (63.22), TOCSY (3.84) |  |
| 33 | L-Threonine | γ-CH_3_ | 1.33 | d | ^1^H, HSQC (22.27), TOCSY (3.61, 4.25) | WCX1, WAX1 |
|  |  | α-CH | 3.61 | d | ^1^H, HSQC (63.38), TOCSY (1.33, 4.25) |  |
|  |  | β-CH | 4.25 | m | ^1^H, HSQC (69.01), TOCSY (1.33, 3.61) |  |
| 34 | L-Tryptophan | β-CH | 3.29 | dd | ^1^H, HSQC (29.14), TOCSY (3.47, 4.06) | WCX1, WAX1, WAX3 |
|  |  | β'-CH | 3.47 | dd | ^1^H, HSQC (29.14), TOCSY (3.29, 4.06) |  |
|  |  | α-CH | 4.06 | dd | ^1^H, HSQC (57.9), TOCSY (3.29, 3.47) |  |
|  |  | ζ'-CH | 7.20 | m | ^1^H, HSQC (122.15), TOCSY (7.28, 7.53, 7.73) |  |
|  |  | ε-CH | 7.28 | m | ^1^H, HSQC (124.81), TOCSY (7.20, 7.53, 7.73) |  |
|  |  | δ-CH | 7.33 | s | ^1^H, HSQC (128.09), TOCSY (2.28, 3.62) |  |
|  |  | ζ-CH | 7.53 | d | ^1^H, HSQC (114.81), TOCSY (7.20, 7.28, 7.73) |  |
|  |  | η-CH | 7.73 | d | ^1^H, HSQC (121.22), TOCSY (7.20, 7.28, 7.53) |  |
| 35 | L-Tyrosine | β-CH_2_ | 3.05 | dd | ^1^H, HSQC (38.37), TOCSY(3.17, 3.94) | WCX1, WAX1, WAX3 |
|  |  | β'-CH_2_ | 3.17 | dd | ^1^H, HSQC (38.37), TOCSY (3.05, 3.94) |  |
|  |  | α-CH | 3.94 | dd | ^1^H, HSQC (59.00), TOCSY (3.05, 3.17) |  |
|  |  | ε-CH | 6.89 | m | ^1^H, HSQC (118.72), TOCSY (7.19) |  |
|  |  | δ-CH | 7.19 | m | ^1^H, HSQC (133.57), TOCSY (6.89) |  |
| 36 | Uridine | CH2 | 3.81 | dd | ^1^H, JRES, TOCSY (3.91, 4.13, 4.23) | WCX1, WAX1 |
|  |  | CH2’ | 3.91 | dd | ^1^H, JRES, TOCSY (3.81, 4.13, 4.23) |  |
|  |  | CH | 4.13 | m | ^1^H, JRES, TOCSY (3.81, 3.91, 4.23) |  |
|  |  | CH | 4.23 | dd | ^1^H, JRES, TOCSY (3.81, 3.91, 4.13, 5.9) |  |
|  |  | CH | 4.35 | dd | ^1^H, JRES, TOCSY (4.13, 4.23, 5.9) |  |
|  |  | CH | 5.89 | d | ^1^H, JRES, TOCSY (4.13, 4.23, 4.35, 7.97) |  |
|  |  | CH | 5.91 | d | ^1^H, JRES, TOCSY (4.13, 4.23, 4.35, 7.97) |  |
|  |  | CH | 7.97 | d | ^1^H, JRES, TOCSY (5.90) |  |
| 37 | Uridine MonoPhosphate  (UMP) | CH_2_ | 3.97 | m | ^1^H, HSQC (ND), TOCSY (4.25, 4.33, 4.39) | WCX1, WAX1, WAX4 |
|  |  | CH | 4.25 | m | ^1^H, HSQC (ND), TOCSY (3.97, 4.33, 4.39, 5.98) |  |
|  |  | CH | 4.33 | t | ^1^H, HSQC (72.60), TOCSY (3.97, 4.25, 4.39, 5.98) |  |
|  |  | CH | 4.39 | t | ^1^H, HSQC (76.67), TOCSY (3.97, 4.25, 4.33, 5.98) |  |
|  |  | CH | 5.97 | m | ^1^H, HSQC (105.43), TOCSY (4.25, 4.33, 4.39) |  |
|  |  | CH | 5.98 | m | ^1^H, HSQC (91.20), TOCSY (4.25, 4.33, 4.39) |  |
|  |  | CH | 8.08 | d | ^1^H, HSQC (ND), TOCSY (5.98) |  |
| 38 | Uridine DiPhosphate  (UDP) | CH_2_ | 4.21 | dd | ^1^H, HSQC (ND), TOCSY (4.27, 4.38, 4.42) | WCX1, WAX4 |
|  |  | CH | 4.27 | m | ^1^H, HSQC (ND), TOCSY (4.21, 4.38, 4.42) |  |
|  |  | CH | 4.38 | t | ^1^H, HSQC (ND), TOCSY (4.21, 4.27, 4.42) |  |
|  |  | CH | 4.42 | t | ^1^H, HSQC (91.39), TOCSY (4.21, 4.27, 4.38) |  |
|  |  | CH | 5.96 | s | ^1^H, HSQC (ND) |  |
|  |  | CH | 5.97 | d | ^1^H, HSQC (105.32), TOCSY ( 7.98) |  |
|  |  | CH | 7.98 | d | ^1^H, HSQC (ND), TOCSY (5.96, 5.97) |  |
| 39 | UDP-N-acetylglucosamine | CH3 | 2.07 | s | ^1^H, HSQC (ND), JRES | WCX1 |
|  |  | CH | 3.56 | dd | ^1^H, HSQC (ND), TOCSY (3.80, 3.87, 3.92, 4.00) |  |
|  |  | CH | 3.80 | m | ^1^H, HSQC (ND), TOCSY (3.56, 3.87,3.92, 4.00) |  |
|  |  | CH2 | 3.87 | dd | ^1^H, HSQC (ND), TOCSY (3.56, 3.80, 3.92) |  |
|  |  | CH | 3.92 | m | ^1^H, HSQC (ND), TOCSY (3.56, 3.80, 3.87, 4.00) |  |
|  |  | CH | 4.00 | ddd | ^1^H, HSQC (56.03), TOCSY (3.56, 3.8, 3.92, 5.51) |  |
|  |  | CH2 | 4.19 | m | ^1^H, HSQC (67.76), TOCSY (4.37, 5.96) |  |
|  |  | CH2’ | 4.24 | m | ^1^H, HSQC (67.76), TOCSY (4.37, 5.96) |  |
|  |  | CH | 4.29 | m | ^1^H, HSQC (85.89), TOCSY (4.26, 5.96) |  |
|  |  | CH | 4.37 | m | ^1^H, HSQC (76.67), TOCSY (4.26, 5.96) |  |
|  |  | CH | 5.51 | dd | ^1^H, HSQC (97.30), TOCSY (3.56, 3.80, 4.00) |  |
|  |  | CH | 5.95 | d | ^1^H, HSQC (105.43), TOCSY (4.37, 7.94) |  |
|  |  | CH | 5.97 | d | ^1^H, HSQC (91.20), TOCSY (4.37, 7.94) |  |
|  |  | CH | 7.94 | d | ^1^H, HSQC (ND), TOCSY (5.96) |  |
| 40 | L-Valine | γ-CH_3_ | 0.99 | d | ^1^H, HSQC (19.45), TOCSY (2.28, 3.62) | WCX1, WAX1 |
|  |  | γ'-CH_3_ | 1.04 | d | ^1^H, HSQC (20.70), TOCSY (2.28, 3.62) |  |
|  |  | β-CH | 2.28 | m | ^1^H, HSQC (31.96), TOCSY (1.02, 3.62) |  |
|  |  | α-CH | 3.62 | d | ^1^H, HSQC (63.38), TOCSY (1.02, 2.28) |  |

*: Not detected because of the water suppression

## Assessment of the computational and experimental DMA workflow with metabolite reference standards

Metabolite reference standards were analysed using the DMA workflow to check that they were separated and annotated correctly – see **Figures S30-31**. The reference standards covered a wide biochemical space including lipid and lipid-like molecules; organic acids and derivatives; organic oxygen compounds; organoheterocyclic compounds; nucleosides, nucleotides, and analogues; and organic nitrogen compounds.

Forty-three of the 48 (89.6%) reference standards were successfully annotated as top ranked. For the apolar components of the workflow, the majority of annotations were for lipid and lipid-like molecules, while for the polar component of the workflow the chemical space was more diverse, with lipid and lipid-like molecules, organic acids and derivatives, alongside other superclasses and classes of compounds observed. The five missing standards, when only using the top ranked metabolite annotations, were: Adenosine triphosphate; pyruvate; 1,2-dioctadecanoyl-sn-glycero-3-phospho-L-serine (PS 18:0/18:0); Nicotinamide adenine dinucleotide phosphate; and spermine.

Our observations indicate that the majority of the missing metabolites were phosphorylated. Enhancements to the workflow could potentially increase their coverage, as recent advancements in liquid chromatography (LC) hardware and column technology offer significant improvements for the analysis of phosphorylated compounds and other compounds that interact with metal surfaces in the LC flow path (DeLano et al., 2021; Gilar et al., 2021; McCalley, 2022).

Spectral matching was able to annotate 39 out of the 48 (81.3 %) reference standard compounds. SIRIUS CSI:FingerID was able to annotate 39 out of the 48 (81.3%) and MetFrag was able to annotate 25 out of the 48 (52.1). The lower percentage of annotation from MetFrag is in part due to the high threshold (>0.95 MetFrag weighted score) used for the final filtering of the MetFrag annotations.

See **Supplemental Table S11** (provided in separate excel file) for a summary of the reference standard annotations.

***
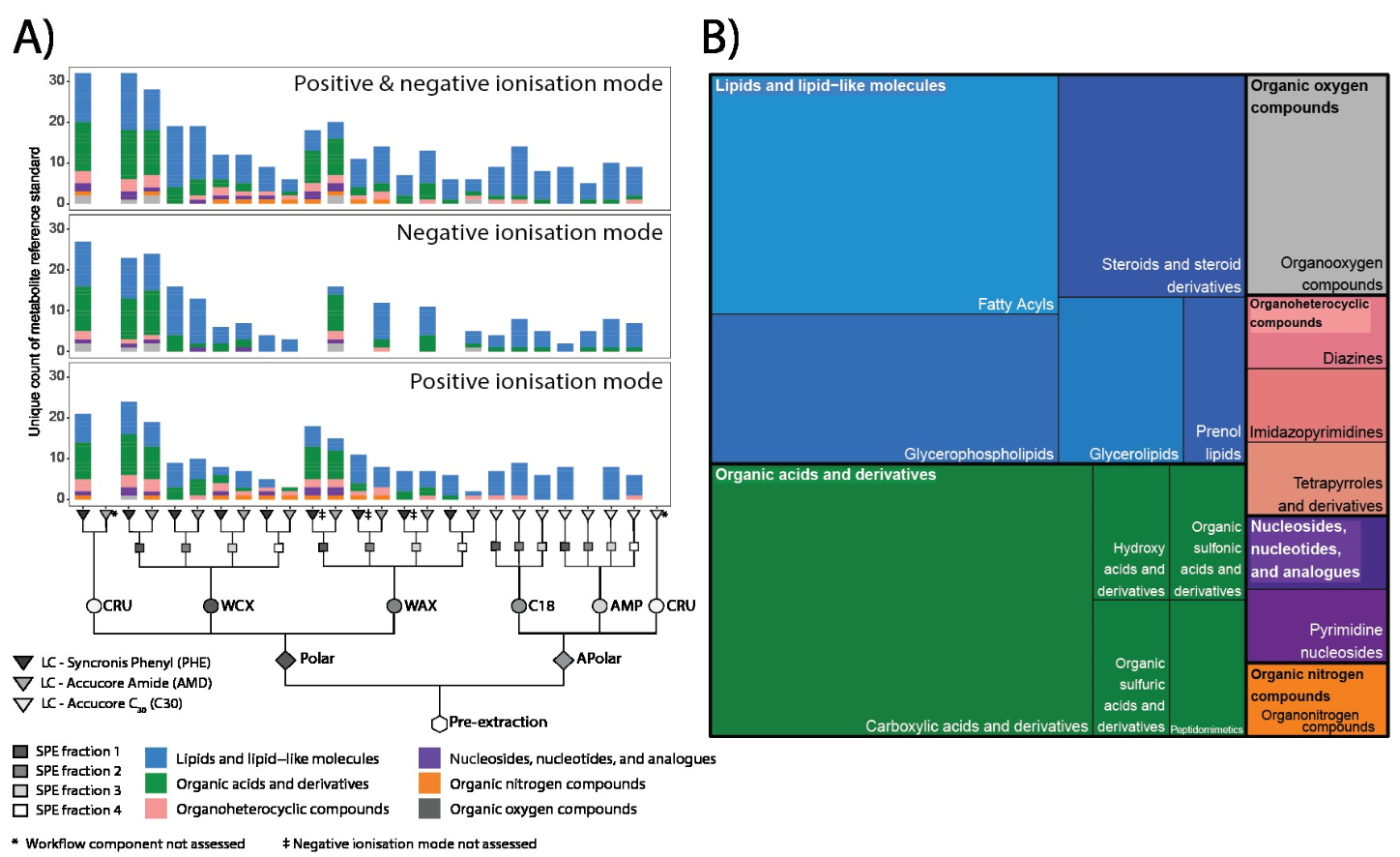
Figure S30: Assessment of the DMA experimental and computational workflow.***  ***A)*** *Count of matched reference standards across the different components of the experimental workflow confirming that, for example, organic acids and derivatives were detected in the polar arm while the apolar arm predominantly detected lipids and lipid-like molecules.* ***B)*** *Treemap of metabolite standard compound classes and superclasses.*


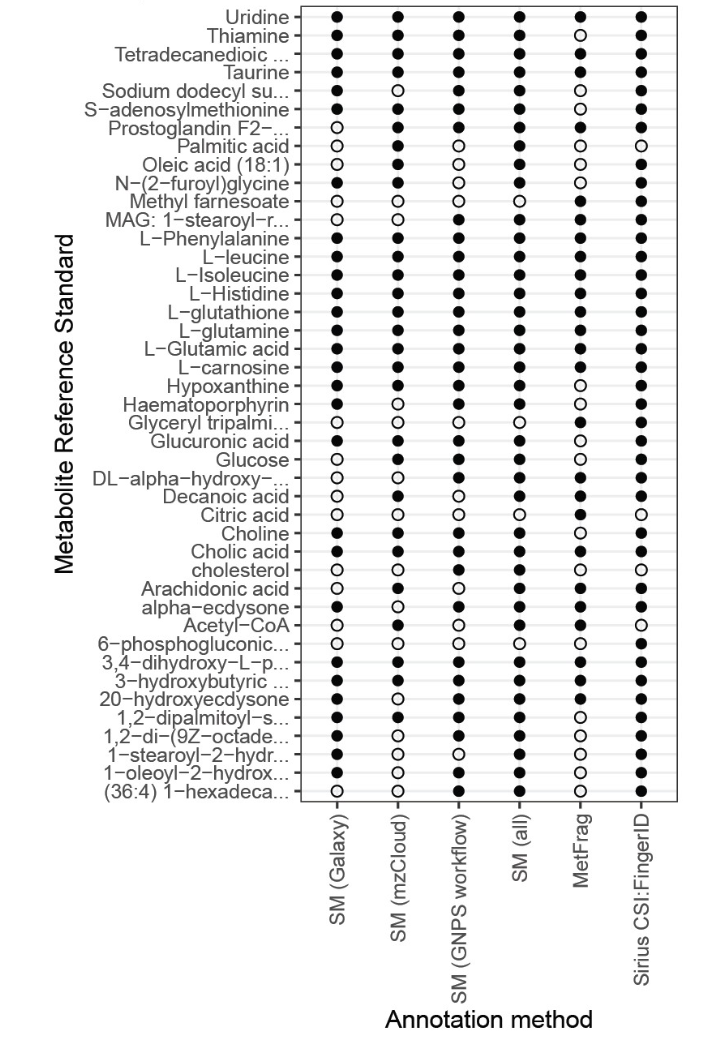


***Figure S31: Summary of which annotation approach was able to identify each metabolite standard.*** *Black circles represent a successful annotation to the standard and white/empty circles represent where the annotation was unsuccessful. SM = spectral matching; SM (all) = a successful annotation using any of the spectral matching approaches.*

## Pathway analysis


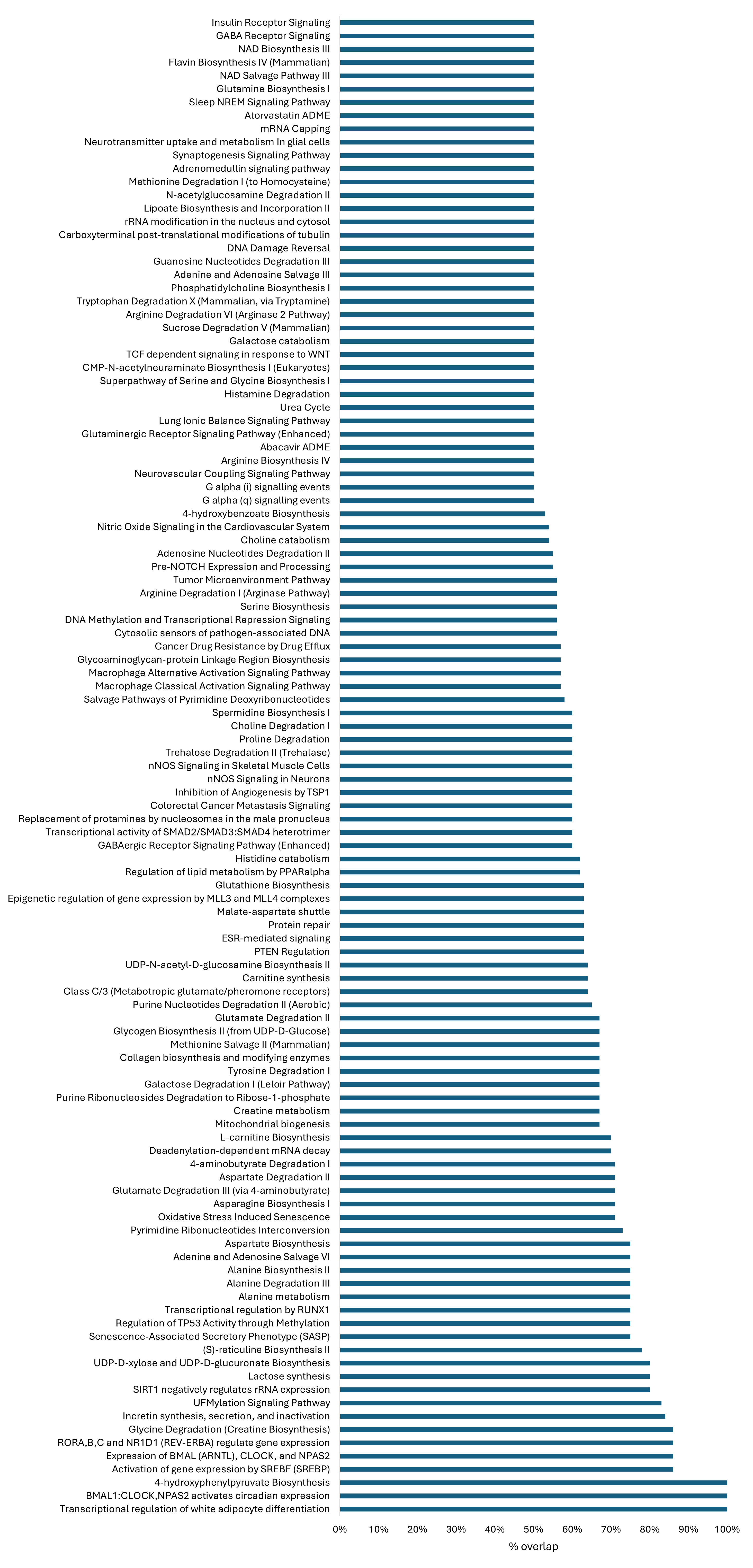


***Figure S32: Summary of the top canonical pathways derived using QIAGEN Ingenuity Pathway Analysis (IPA) for all annotations obtained from the DMA of D. magna.*** *Pathway coverage is shown as a percentage at the top and the numbers on the right represent metabolites in the particular pathway. Only showing pathways >=3 metabolites (measured) and >=50% coverage.*

## Molecular network analysis using GNPS

Spectral networks generated using the GNPS network analysis workflow were performed across all of the *D. magna* data files separated into positive and negative ionisation data.

For the positive ionisation mode the network contains 616,499 spectra of which 78,353 of the spectra are annotated using the GNPS spectral matching libraries. The GNPS workflow determined 31,323 distinct clusters of the network (including singlets) of which 1,052 had some level of annotation to GNPS spectral matching libraries. MS2LDA analysis on the positive ionisation network determined 47,853 mass motifs.

For the negative ionisation mode the network contains 165,430 spectra of which 17,666 of the spectra are annotated using the GNPS spectral matching libraries. The GNPS workflow determined 5,320 distinct clusters of the network (including singlets) of which 187 had some level of annotation to GNPS spectral matching libraries. MS2LDA analysis on the negative ionisation network determined 8,681 mass motifs.

**
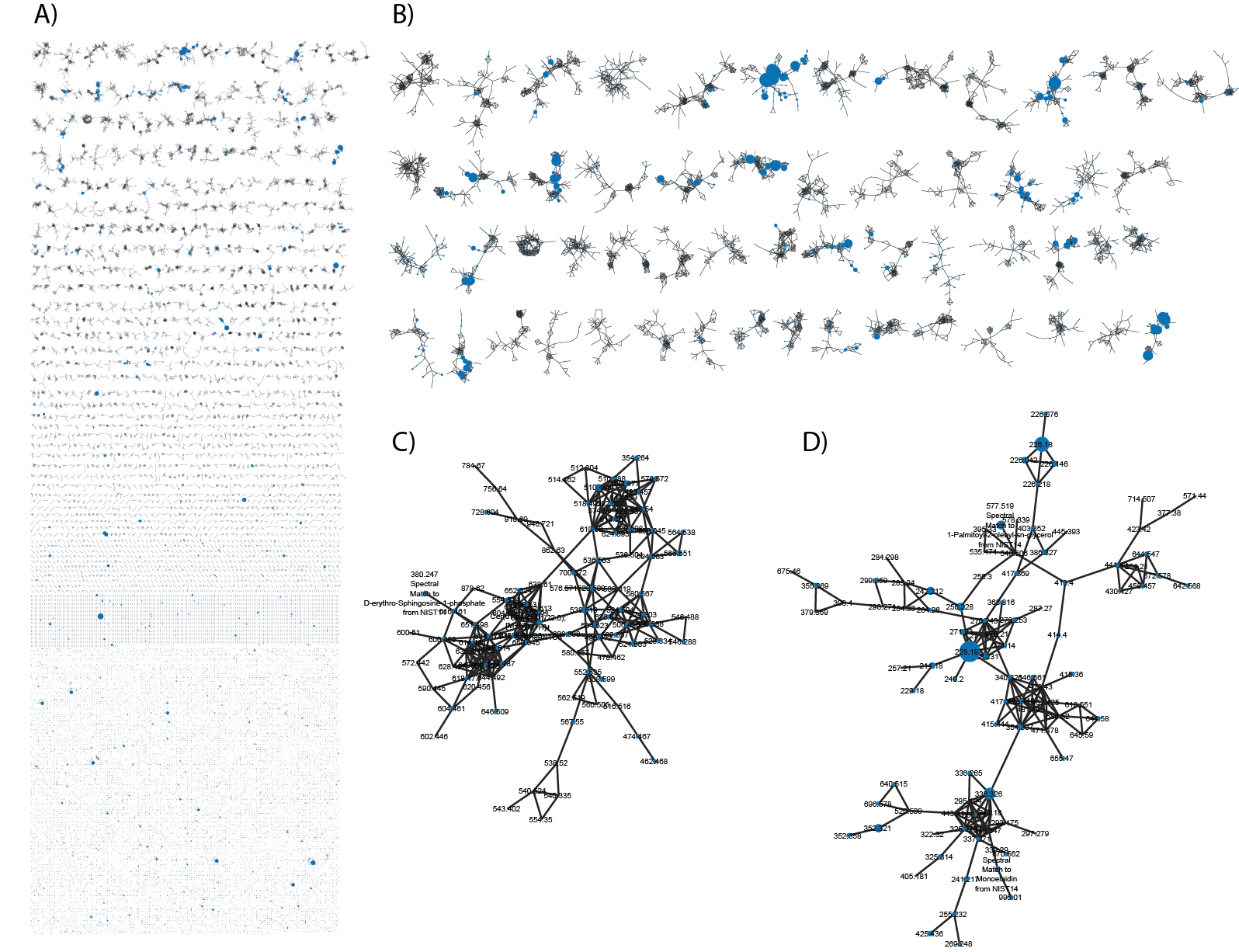
**

**Figure S33: GNPS spectral network analysis (positive ionisation mode).** A) Overview of positive ionisation mode spectral networks generated from GNPS molecular network analysis showing all 31,323 distinct clusters. B) the top 55 clusters (based on how many nodes were observed). C) and D) show the top 2 clusters (again based on how many nodes were observed) which further display the precursor m/z associated with the node and the compound name of any spectral annotation derived from spectral matching within GNPS. For A), the largest cluster observed, two spectral matching annotations were observed for D-erythro-Sphingosine-1-phosphate and Cer(d18:1/22:0). For B), the second largest cluster observed, two spectral matching annotations for Monoelaidin and 1-Palmitoyl-2-oleoyl-sn-glycerol.

# References

Baer, K.N., Goulden, C.E., 1998. Evaluation of a high-hardness COMBO medium and frozen algae for Daphnia magna. Ecotoxicology and environmental safety 39, 201–206. https://doi.org/10.1006/eesa.1997.1627

Bligh, E.G., Dyer, W.J., 1959. A rapid method of total lipid extraction and purification. Canadian Journal of Biochemistry and Physiology 37, 911–917.

Chao, A., Al-Ghoul, H., McEachran, A.D., Balabin, I., Transue, T., Cathey, T., Grossman, J.N., Singh, R.R., Ulrich, E.M., Williams, A.J., Sobus, J.R., 2020. In silico MS/MS spectra for identifying unknowns: a critical examination using CFM-ID algorithms and ENTACT mixture samples. Anal Bioanal Chem 412, 1303–1315. https://doi.org/10.1007/s00216-019-02351-7

Chauve, B., Guillarme, D., Cléon, P., Veuthey, J.-L., 2010. Evaluation of various HILIC materials for the fast separation of polar compounds. Journal of separation science 33, 752–764.

Davis, A.L., Laue, E.D., Keeler, J., Moskau, D., Lohman, J., 1991. Absorption-mode two-dimensional NMR spectra recorded using pulsed field gradients. Journal of Magnetic Resonance (1969) 94, 637–644. https://doi.org/10.1016/0022-2364(91)90154-L

DeLano, M., Walter, T.H., Lauber, M.A., Gilar, M., Jung, M.C., Nguyen, J.M., Boissel, C., Patel, A.V., Bates-Harrison, A., Wyndham, K.D., 2021. Using Hybrid Organic–Inorganic Surface Technology to Mitigate Analyte Interactions with Metal Surfaces in UHPLC. Anal. Chem. 93, 5773–5781. https://doi.org/10.1021/acs.analchem.0c05203

Djoumbou Feunang, Y., Eisner, R., Knox, C., Chepelev, L., Hastings, J., Owen, G., Fahy, E., Steinbeck, C., Subramanian, S., Bolton, E., Greiner, R., Wishart, D.S., 2016. ClassyFire: automated chemical classification with a comprehensive, computable taxonomy. J Cheminform 8, 61. https://doi.org/10.1186/s13321-016-0174-y

Dührkop, K., Fleischauer, M., Ludwig, M., Aksenov, A.A., Melnik, A.V., Meusel, M., Dorrestein, P.C., Rousu, J., Böcker, S., 2019. SIRIUS 4: a rapid tool for turning tandem mass spectra into metabolite structure information. Nat Methods 16, 299–302. https://doi.org/10.1038/s41592-019-0344-8

Garreta-Lara, E., Campos, B., Barata, C., Lacorte, S., Tauler, R., 2016. Metabolic profiling of Daphnia magna exposed to environmental stressors by GC–MS and chemometric tools. Metabolomics 12, 86. https://doi.org/10.1007/s11306-016-1021-x

Giacomoni, F., Le Corguille, G., Monsoor, M., Landi, M., Pericard, P., Petera, M., Duperier, C., Tremblay-Franco, M., Martin, J.-F., Jacob, D., Goulitquer, S., Thevenot, E.A., Caron, C., 2015. Workflow4Metabolomics: a collaborative research infrastructure for computational metabolomics. Bioinformatics 31, 1493–1495. https://doi.org/10.1093/bioinformatics/btu813

Gilar, M., DeLano, M., Gritti, F., 2021. Mitigation of analyte loss on metal surfaces in liquid chromatography. Journal of Chromatography A 1650, 462247. https://doi.org/10.1016/j.chroma.2021.462247

Heaton, J.C., McCalley, D.V., 2016. Some factors that can lead to poor peak shape in hydrophilic interaction chromatography, and possibilities for their remediation. Journal of Chromatography A 1427, 37–44. https://doi.org/10.1016/j.chroma.2015.10.056

Heller, S.R., McNaught, A.D., 2009. The IUPAC International Chemical Identifier (InChI). Chemistry International 31, 7–9. https://doi.org/10.1515/ci.2009.31.1.7

Hoch, J.C., Baskaran, K., Burr, H., Chin, J., Eghbalnia, H.R., Fujiwara, T., Gryk, M.R., Iwata, T., Kojima, C., Kurisu, G., Maziuk, D., Miyanoiri, Y., Wedell, J.R., Wilburn, C., Yao, H., Yokochi, M., 2022. Biological Magnetic Resonance Data Bank. Nucleic Acids Research 51, D368–D376. https://doi.org/10.1093/nar/gkac1050

Johnson, J.R., Karlsson, D., Dalene, M., Skarping, G., 2010. Determination of aromatic amines in aqueous extracts of polyurethane foam using hydrophilic interaction liquid chromatography and mass spectrometry. Analytica Chimica Acta 678, 117–123. https://doi.org/10.1016/j.aca.2010.08.020

Kikuchi, J., Tsuboi, Y., Komatsu, K., Gomi, M., Chikayama, E., Date, Y., 2016. SpinCouple: Development of a Web Tool for Analyzing Metabolite Mixtures via Two-Dimensional J-Resolved NMR Database. Anal Chem 88, 659–665. https://doi.org/10.1021/acs.analchem.5b02311

Kuhl, C., Tautenhahn, R., Böttcher, C., Larson, T.R.R., Neumann, S., Bo, C., Larson, T.R.R., Neumann, S., 2012. CAMERA: An integrated strategy for compound spectra extraction and annotation of liquid chromatography/mass spectrometry data sets. Anal. Chem. 84, 283–289. https://doi.org/10.1021/ac202450g

Lawson, T.N., Weber, R.J.M., Jones, M.R., Chetwynd, A.J., Rodriguez Blanco, G.A., Di Guida, R., Viant, M.R., Dunn, W.B., 2017. msPurity: Automated evaluation of precursor ion purity for mass spectrometry based fragmentation in metabolomics. Anal. Chem. 89, acs.analchem.6b04358. https://doi.org/10.1021/acs.analchem.6b04358

Libiseller, G., Dvorzak, M., Kleb, U., Gander, E., Eisenberg, T., Madeo, F., Neumann, S., Trausinger, G., Sinner, F., Pieber, T., others, 2015. IPO: a tool for automated optimization of XCMS parameters. BMC bioinformatics 16, 118.

Ludwig, C., Easton, J.M., Lodi, A., Tiziani, S., Manzoor, S.E., Southam, A.D., Byrne, J.J., Bishop, L.M., He, S., Arvanitis, T.N., Günther, U.L., Viant, M.R., 2012. Birmingham Metabolite Library: a publicly accessible database of 1-D 1H and 2-D 1H J-resolved NMR spectra of authentic metabolite standards (BML-NMR). Metabolomics 8, 8–18. https://doi.org/10.1007/s11306-011-0347-7

McCalley, D.V., 2022. Influence of metals in the column or instrument on performance in hydrophilic interaction liquid chromatography. Journal of Chromatography A 1663, 462751. https://doi.org/10.1016/j.chroma.2021.462751

Neumann, S., Thum, A., Böttcher, C., 2013. Nearline acquisition and processing of liquid chromatography-tandem mass spectrometry data. Metabolomics 9, 84–91. https://doi.org/10.1007/s11306-012-0401-0

Orekhov, V.Y., Jaravine, V.A., 2011. Analysis of non-uniformly sampled spectra with multi-dimensional decomposition. Progress in nuclear magnetic resonance spectroscopy 59, 271–292. https://doi.org/10.1016/j.pnmrs.2011.02.002

Ruta, J., Rudaz, S., McCalley, D.V., Veuthey, J.-L., Guillarme, D., 2010. A systematic investigation of the effect of sample diluent on peak shape in hydrophilic interaction liquid chromatography. Journal of Chromatography A 1217, 8230–8240. https://doi.org/10.1016/j.chroma.2010.10.106

Ruttkies, C., Neumann, S., Posch, S., 2019. Improving MetFrag with statistical learning of fragment annotations. BMC Bioinformatics 20, 376. https://doi.org/10.1186/s12859-019-2954-7

Ruttkies, C., Schymanski, E.L., Wolf, S., Hollender, J., Neumann, S., 2016. MetFrag relaunched: Incorporating strategies beyond in silico fragmentation. J Cheminform 8, 1–16. https://doi.org/10.1186/s13321-016-0115-9

Shaka, A., Lee, C., Pines, A., 1988. Iterative schemes for bilinear operators; application to spin decoupling. Journal of Magnetic Resonance (1969) 77, 274–293.

Smith, C.A., Want, E.J., Maille, G.O., Abagyan, R., Siuzdak, G., 2006. XCMS : Processing Mass Spectrometry Data for Metabolite Profiling Using Nonlinear Peak Alignment , Matching , and Identification. Anal. Chem. 78, 779–787. https://doi.org/10.1021/ac051437y

Southam, A.D., Weber, R.J.M., Engel, J., Jones, M.R., Viant, M.R., 2017. A complete workflow for high-resolution spectral-stitching nanoelectrospray direct-infusion mass-spectrometry-based metabolomics and lipidomics. Nature Protocols 12, 310–328. https://doi.org/10.1038/nprot.2016.156

Vorkas, P.A., Isaac, G., Anwar, M.A., Davies, A.H., Want, E.J., Nicholson, J.K., Holmes, E., 2015. Untargeted UPLC-MS Profiling Pipeline to Expand Tissue Metabolome Coverage: Application to Cardiovascular Disease. Anal. Chem. 87, 4184–4193. https://doi.org/10.1021/ac503775m

Wishart, D.S., Tzur, D., Knox, C., Eisner, R., Guo, A.C., Young, N., Cheng, D., Jewell, K., Arndt, D., Sawhney, S., Fung, C., Nikolai, L., Lewis, M., Coutouly, M.-A., Forsythe, I., Tang, P., Shrivastava, S., Jeroncic, K., Stothard, P., Amegbey, G., Block, D., Hau, D.D., Wagner, J., Miniaci, J., Clements, M., Gebremedhin, M., Guo, N., Zhang, Y., Duggan, G.E., Macinnis, G.D., Weljie, A.M., Dowlatabadi, R., Bamforth, F., Clive, D., Greiner, R., Li, L., Marrie, T., Sykes, B.D., Vogel, H.J., Querengesser, L., 2007. HMDB: the Human Metabolome Database. Nucleic acids research 35, D521--6. https://doi.org/10.1093/nar/gkl923

Wolf, S., Schmidt, S., Müller-Hannemann, M., Neumann, S., 2010. In silico fragmentation for computer assisted identification of metabolite mass spectra. BMC Bioinformatics 11, 148. https://doi.org/10.1186/1471-2105-11-148
