# Supplementary material for: Experimental and computational approaches for deep metabolome annotation with application to the ecotoxicological model organism Daphnia magna
Source: Gigascience. 2026 May 9;15:giag055. doi: 10.1093/gigascience/giag055 (PMC13254479; doi:10.1093/gigascience/giag055)
Supplement: giag055_GIGA-D-25-00453_original_submission [file giag055_giga-d-25-00453_original_submission.pdf]

GigaScience

Experimental and computational approaches for deep metabolome annotation with application to the ecotoxicological model organism Daphnia magna

--Manuscript Draft--

Manuscript Number:	GIGA-D-25-00453
Full Title:	Experimental and computational approaches for deep metabolome annotation with application to the ecotoxicological model organism Daphnia magna
Article Type:	Research
Funding Information:	Natural Environment Research Council (NE/L002493/1) — Dr Thomas N Lawson<br>Natural Environment Research Council (NE/J017442/1) — Dr Martin R Jones<br>Wellcome Trust (202952/Z/16/Z) — Dr Ralf J. M Weber<br>H2020 European Institute of Innovation and Technology (965406) — Professor Mark R Viant
Abstract:	Background: Comprehensively characterising the metabolomes of model organisms with high coverage and confidence is a critical step towards interpreting the metabolic basis of human and environmental health, yet there are formidable challenges involved in annotating metabolomes. A wide range of genotypes and phenotypes should be sampled with multiple complementary analytical approaches to cover the large and dynamic biochemical space they exhibit. In addition, multiple computational tools and approaches are required to annotate the metabolites from raw analytical data.<br>Results: To address this, we developed the Deep Metabolome Annotation (DMA) workflow. Applied to the ecological sentinel species, Daphnia magna, one pooled sample comprising ten distinct genotypes exposed to both normal and stressed environmental conditions was extracted and systematically physicochemically separated via solid-phase extraction, liquid- and gas-chromatography prior to extensive multiple-stage mass spectrometric fragmentation, generating more than 8,000 raw data files, and supplemented by nuclear magnetic resonance spectroscopy. An extensive Galaxy-based computational approach was built to analyse these data, comprising over 30 tools. The overall DMA efforts resulted in 8,577 annotated polar metabolites and lipids in D. magna, with the raw and processed data, tools and annotations disseminated freely via public data repositories and a custom web-based interface to maximise reusability and facilitate transferability.<br>Conclusions: The DMA workflow has generated one of the largest metabolome annotation datasets for any non-human model organism and provides the first in-depth characterisation of the D. magna metabolome – providing both a resource and a valuable catalyst for future deep metabolome annotation studies of other model organisms.
Corresponding Author:	Mark Viant<br>University of Birmingham<br>Birmingham, UNITED KINGDOM
Corresponding Author Secondary Information:	
Corresponding Author's Institution:	University of Birmingham
Corresponding Author's Secondary Institution:	
First Author:	Martin R Jones
First Author Secondary Information:	
Order of Authors:	Martin R Jones<br>Thomas N Lawson<br>Andrew J Chetwynd

Powered by Editorial Manager® and ProduXion Manager® from Aries Systems Corporation

	Elena Sostare
	Stefan Weidt
	Robert Mistrik
	Warwick B Dunn
	Ralf J. M Weber
	Mark R Viant
**Order of Authors Secondary Information:**	
**Additional Information:**	
**Question**	**Response**
Are you submitting this manuscript to a special series or article collection?	No
**Experimental design and statistics**<br><br>Full details of the experimental design and statistical methods used should be given in the Methods section, as detailed in our Minimum Standards Reporting Checklist. Information essential to interpreting the data presented should be made available in the figure legends.<br><br>Have you included all the information requested in your manuscript?	Yes
**Resources**<br><br>A description of all resources used, including antibodies, cell lines, animals and software tools, with enough information to allow them to be uniquely identified, should be included in the Methods section. Authors are strongly encouraged to cite Research Resource Identifiers (RRIDs) for antibodies, model organisms and tools, where possible.<br><br>Have you included the information requested as detailed in our Minimum Standards Reporting Checklist?	Yes
**Availability of data and materials**<br><br>All datasets and code on which the	Yes

conclusions of the paper rely must be either included in your submission or deposited in publicly available repositories (where available and ethically appropriate), referencing such data using a unique identifier in the references and in the "Availability of Data and Materials" section of your manuscript.<br><br>Have you have met the above requirement as detailed in our Minimum Standards Reporting Checklist?	
GigaScience has policies and guidelines in place for the use of generative AI-writing tools such as ChatGPT. If you have used such writing tools to assist with writing the manuscript this must be declared and cited in the text. Authors should not list AI-writing tools and other AI-assisted technologies as an author or co-author and should acknowledge that they are fully responsible for text generated or refined by AI-writing tools.<p><br><br>A summary of use (particularly in the introduction or among methods) needs to be included at the end of the paper, and the outputs should also be included as a supplementary file hosted in GigaDB or other open repositories. Please read our guidelines for more information. <p><br><br>By submitting to GigaScience, you are aware of the journal's AI-writing tools policy, and if you have declared use of such tools below, you have acknowledged this where appropriate in your manuscript and have made a summary of use and outputs available. <p><br><b>AI-assisted writing tools have been used in the preparation of this manuscript?	No

Powered by Editorial Manager® and ProduXion Manager® from Aries Systems Corporation

Click here to access/download;Manuscript;DMA D. magna - manuscript - 28-10-2025.docx

Experimental and computational approaches for deep metabolome annotation with application to the ecotoxicological model organism *Daphnia magna*

Martin R. Jones[1,†], Thomas N. Lawson[1,2,†], Andrew J. Chetwynd[1,3,α], Elena Sostare[2], Stefan Weidt[5], Robert Mistrik[4,δ], Warwick B. Dunn[1,3,§], Ralf J. M. Weber[1,3,*], Mark R. Viant[1,2,3,*]

[1]School of Biosciences, University of Birmingham, Edgbaston, Birmingham, B15 2TT, UK
[2]Michabo Health Science Limited, Union House, 111 New Union Street, Coventry, CV1 2NT, UK
[3]Phenome Centre Birmingham, University of Birmingham, Edgbaston, Birmingham, B15 2TT, UK
[4]HighChem, Mlynské nivy 5, 821 09 Bratislava, Slovakia
[5]Glasgow Polyomics, University of Glasgow, University Avenue, Glasgow, G12 8QQ, UK

[†]Joint first authors
Present addresses: [α]Centre for Proteome Research, and [§]Centre for Metabolomics Research, Department of Biochemistry, Cell and Systems Biology, Institute of Systems, Molecular and Integrative Biology, University of Liverpool, Liverpool, L69 7ZB, UK; [δ]Bitmoderna, Leskova 11, 81104 Bratislava, Slovakia.

*Correspondence address: School of Biosciences, University of Birmingham, Edgbaston, Birmingham, B15 2TT, UK; E-mail: m.viant@bham.ac.uk; E-Mail: r.j.weber@bham.ac.uk

Abstract

Background: Comprehensively characterising the metabolomes of model organisms with high coverage and confidence is a critical step towards interpreting the metabolic basis of human and environmental health, yet there are formidable challenges involved in annotating metabolomes. A wide range of genotypes and phenotypes should be sampled with multiple complementary analytical approaches to cover the large and dynamic biochemical space they exhibit. In addition, multiple computational tools and approaches are required to annotate the metabolites from raw analytical data.

Results: To address this, we developed the Deep Metabolome Annotation (DMA) workflow. Applied to the ecological sentinel species, *Daphnia magna,* one pooled sample comprising ten distinct genotypes exposed to both normal and stressed environmental conditions was extracted and systematically physicochemically separated via solid-phase extraction, liquid- and gas-chromatography prior to extensive multiple-stage mass spectrometric fragmentation, generating more than 8,000 raw data files, and supplemented by nuclear magnetic resonance spectroscopy. An extensive Galaxy-based computational approach was built to analyse these data, comprising over 30 tools. The overall DMA efforts resulted in 8,577 annotated polar metabolites and lipids in *D. magna,* with the raw and processed data, tools and annotations disseminated freely via public data repositories and a custom web-based interface to maximise reusability and facilitate transferability.

Conclusions: The DMA workflow has generated one of the largest metabolome annotation datasets for any non-human model organism and provides the first in-depth characterisation of the *D. magna* metabolome – providing both a resource and a valuable catalyst for future deep metabolome annotation studies of other model organisms.

Table of contents

1 Introduction 4
2 Methods 7
2.1 Deep metabolome annotation experimental workflow 7
2.1.1 Overview 7
2.1.2 D. magna culturing and sample preparation 7
2.1.3 Metabolite extraction from D. magna pooled sample 7
2.1.4 Solid phase extraction-based fractionation of metabolite extracts 8
2.1.5 (U)HPLC-HRMS(/MS), DI-HRMS(/MS^n) and LC fractionation 8
2.1.6 (U)HPLC-HRMS(/MS) method optimisation 9
2.1.7 GC-EI-HRMS 9
2.1.8 1D & 2D NMR spectroscopy 9
2.2 Computational tools and workflows for data processing, metabolite annotation and data analysis 11
2.2.1 Overview 11
2.2.2 Galaxy workflow details 11
2.2.3 mzCloud library search 13
2.2.4 GNPS library search 13
2.2.5 GNPS molecular network analysis 13
2.2.6 Combining and summarising all annotations 13
2.2.7 Comparison to other metabolite databases 14
2.3 Assessment of the computational and experimental DMA workflow with metabolite reference standards 14
2.4 Deep metabolome annotation database (DMAdb) 14
3 Results and discussion 15
3.1 (U)HPLC-HRMS(/MS) method optimisation 15
3.2 Summary of all *D. magna* metabolite annotations and compound classifications 15
3.3 Metabolites and compound classes physicochemically separated by DMA experimental workflow 20
3.4 Comparison to other metabolite databases 23
3.5 Molecular network analysis using GNPS 25
3.6 Deep metabolome annotation database (DMAdb) 27
4 Conclusion 28
5 Code availability 30
6 Additional files 33
7 Abbreviations 36
8 Acknowledgements 36
9 Author contributions 37
10 Funding 37
11 Data availability 38
12 Competing interests 38
13 References 38
14 Author notes 42

1 Introduction

Large-scale efforts to map and catalogue both human and model organism genomes have been a fundamental driver of change in biological and biochemical research over the past few decades. The technological developments and resulting biological, biomedical and environmental knowledge derived from such projects have helped underpin the modern era of biological sciences [1–4]. In contrast, our understanding of metabolic biochemistry (where we use the term metabolites here to represent the full spectrum of low molecular weight endogenous biochemicals from polar metabolites to lipids) has increased relatively minimally over the last half-a-century. Such knowledge must either be inferred from genome-scale metabolic reconstructions or, if measured experimentally, is limited to metabolites that can be annotated analytically (i.e. using metabolomics datasets). Ongoing improvements in both analytical and computational methods for metabolic annotation now allow for more extensive metabolite annotation coverage than what could be performed 10 years ago. However, although these developments are welcomed and are in part reflected by the increase in both studies featuring extensive metabolite annotation analysis [5–7] and the maturation of databases containing metabolites and relevant experimental data (Metabolights, Metabolomics Workbench, HMDB, GNPS, MoNA, MassBank, LipidBlast and mzCloud [8–13], for the majority of widely used model organisms the metabolome knowledge is still severely lacking.

Whilst the need for deeper metabolome knowledge of model organisms has been well established [14], the challenges are still considerable and multi-faceted. First, the biology: as the metabolome is driven by genes, the changing environment, and their interactions, achieving a comprehensive map of the breadth of a species' metabolome requires a range of genotypes and phenotypes. Second, analytical chemistry: no single method is sufficient to cover the chemical space of a metabolome, hence multiple physicochemical separations and detection techniques are required. Third, the computational challenges: as metabolomes are vast, specialised tools and workflows for data processing and metabolite annotation are required, together with resources for data and metadata management. Where possible, the data generated and software used should be Findable, Accessible, Interoperable, Reusable (FAIR) and scalable in order to support an anticipated further cascade of deep metabolome annotation studies.

To address these challenges, we have developed and applied an experimental and computational workflow for extensively measuring the metabolome of model organisms, applied here to *Daphnia magna*. The International Metabolomics Society's Model Organism Metabolomes task group [15] and the on-going Precision Toxicology project [16] both highlight the crustacean *Daphnia* as a key model organism due to its importance as an indicator genus used to set ecotoxicological regulatory standards (SOR/2002-222), and from being extensively studied in the context of evolution and ecology [17–21], making it an excellent candidate for an in-depth investigation of its metabolome. Experimentally, the workflow involved culturing multiple genotypically distinct strains of *D. magna* under normal and stressed conditions to provide a representative pooled sample for metabolome annotation. This sample underwent extensive extraction and physicochemical separation and (ultra)-high-performance liquid chromatography-high resolution tandem mass spectrometry ((U)HPLC-HRMS(/MS)) analysis. Concurrent fractionation yielded (semi-) purified metabolome fractions that underwent in-depth characterisation by direct infusion-

high resolution multiple stage mass spectrometry (DI-HRMS(MS^n)), and supplemental analysis by gas chromatography-electron ionisation-high resolution mass spectrometry (GC-EI-HRMS) and 1- and 2-dimensional nuclear magnetic resonance spectroscopy (1D- & 2D-NMR), ensuring broad coverage of the physicochemical space of metabolites were measured. A computational workflow was then developed and applied for processing, annotating and managing the data and results, heavily utilising the Galaxy Workflow platform [22]. The resulting metabolome annotations, data, metadata and computational tools are disseminated through various channels (MetaboLights, GNPS, Galaxy and a custom web portal, named DMAdb) to ensure traceability and reusability.

The combined extensive experimental and computational workflow, referred to here as the Deep Metabolome Annotation (DMA) workflow (see **Figure 1**), has generated one of the largest metabolome annotation datasets for any single organism and provides the first in-depth characterisation of the *D. magna* metabolome – a resource that is much needed to improve the interpretation of *Daphnia* biology and toxicology. This workflow can be redeployed for deep metabolome annotation of other model organisms.

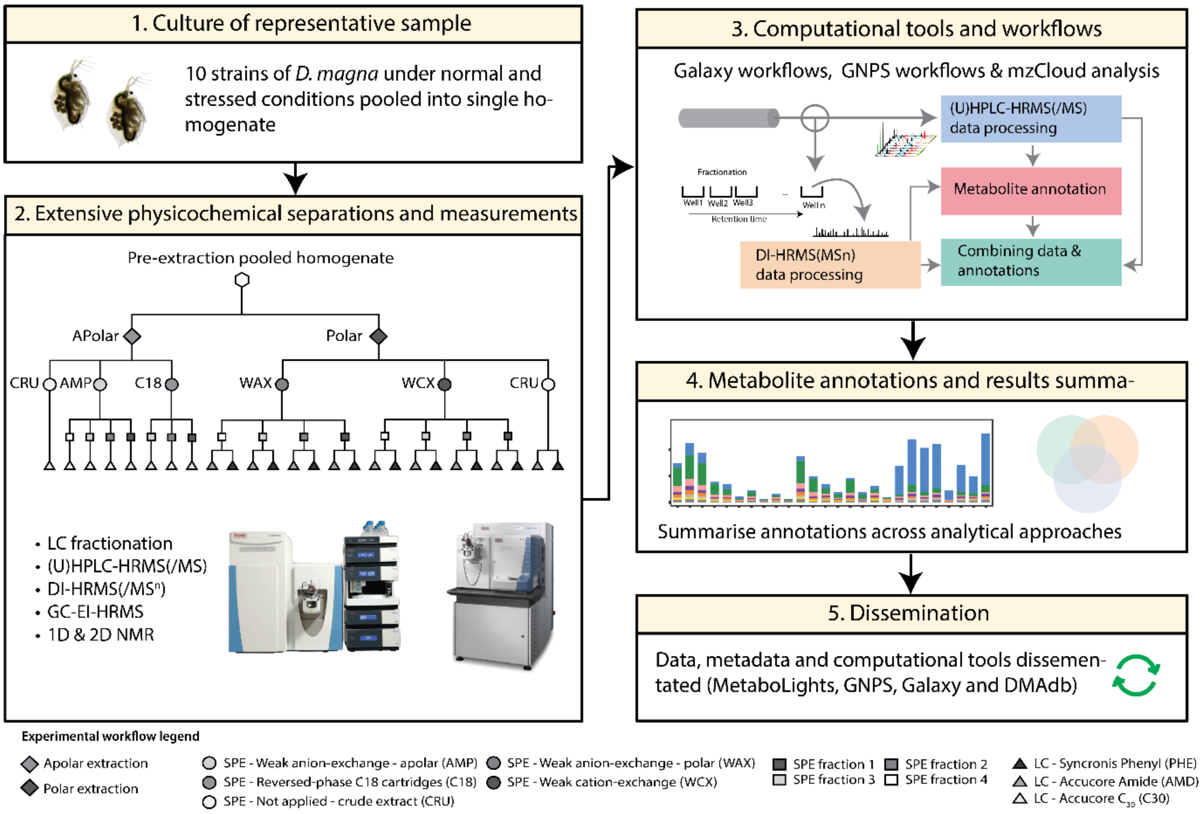

Figure 1: Deep Metabolome Annotation (DMA) workflow - conceptual overview and application to D. magna
1) *First a representative sample (applied to D. magna here) cultured under multiple conditions using genotypically distinct strains is homogenised into a single sample.* ***2)*** *The pooled homogenised sample then undergoes extensive physicochemical separations (including polar and apolar extraction procedures; four types of solid phase extraction (SPE) – each collecting 3-4 SPE fraction; three types of liquid chromatography (LC); and extensive LC fractionation). Followed by extensive analysis using (ultra)-high-performance liquid chromatography-high resolution tandem mass spectrometry ((U)HPLC-HRMS(/MS)), direct infusion-high resolution mass spectrometry with multiple-stage fragmentation (DI-HRMS(MS^n))) and supplemented with gas chromatography-electron ionisation-high resolution mass spectrometry (GC-EI-HRMS) and 1- and 2-dimensional nuclear magnetic resonance spectroscopy (1D- & 2D-NMR).* ***3)*** *Extensive computational tools and workflows*

were developed and applied to process and annotate the metabolite. The results are then summarised across the analytical workflow ***(4)*** *and finally the data, metadata and computational tools are disseminated to ensure traceability and reusability* ***(5)****.*

ALT TEXT: Graphical representation of the four stages of the deep metabolome annotation workflow.

2 Methods

2.1 Deep metabolome annotation experimental workflow

2.1.1 Overview

An experimental workflow for extensive physicochemical separation and analytical measurement of metabolites has been developed for the analysis of model organisms – applied here to *D. magna*. An overview is provided in Figure 2.

This extensive workflow, applied to both the *Daphnia* sample and a metabolite reference standard sample, was separated into 135 distinct experimental assays (see **Supplemental Table S1**). Of these assays, 103 corresponded to (U)HPLC-HRMS(/MS) and DI-HRMS(/MS^n) analysis, generating 8,846 raw mass spectrometry files (5,430 files specifically measuring the *Daphnia* sample with the remaining files either for metabolite reference standards, blanks or quality assurance measurements such as mass spectrometer equilibration). See **Supplemental Table S2** for the full file list.

2.1.2 D. magna culturing and sample preparation

The DMA experimental workflow is applied here to *D. magna*, but the same considerations apply for other potential model organism DMA analyses. The workflow should take as input a set of samples that, ideally, span diverse genetic and environmental backgrounds and collectively reflect the full metabolic repertoire accessible to the organism under study. These samples are then pooled and homogenised to form a single complex sample matrix that constitutes an average of the constituent metabolomes.

For the DMA of *D. magna*, ca. 2000 individual organisms were pooled and homogenised, generating a homogenate consisting of ten genotypically distinct strains (**Supplemental Table S3)** exposed to two contrasting environmental conditions. A ‘basal’ metabolome was represented by *D. magna* cultured under standard conditions (20 +/- 2 °C with a 16:8 hr light:dark ratio) for 14 days, followed by a further 48 hr under the same conditions. A ‘stressed’ metabolome was represented by *D. magna* cultured for 14 days under standard conditions, followed by 24 hr at 10 +/- 1 °C with 16:8 hr light:dark ratio, and then a further 24 hr at 10 +/- 1 °C with 8:16 hr light:dark ratio. Under both conditions, *Daphnia* were maintained without food (algae) throughout the final 48 hr of culturing, to minimise the presence of algae in the gut and prioritise measurement of metabolites derived from *Daphnia* rather than the food source. Further details are provided in **Supplemental Section 1.5** and **Supplemental Tables S4-5**.

2.1.3 Metabolite extraction from D. magna pooled sample

Metabolites were extracted from the pooled homogenate using two distinct liquid-phase extraction protocols: a ‘polar’ extraction in which (predominantly polar through to moderately-polar polar) metabolites were extracted using a solution comprising 71.4:28.6% v/v methanol:water, and; an ‘apolar’ extraction protocol in which metabolites (spanning moderately-apolar through to highly apolar metabolites, including lipids) were extracted using a solution of 1:1 v/v methanol:chloroform, to which water was added to form a biphasic system comprising 2:2:1.8 v/v/v chloroform:methanol:water, from which the lower (apolar)

layer was recovered. Polar extracts were dried in a centrifugal vacuum concentrator (Speedvac), while apolar extracts were dried under a stream of nitrogen gas. Further details are provided in **Supplemental Section 1.6**.

2.1.4 Solid phase extraction-based fractionation of metabolite extracts

Constituents of the polar or apolar extract were independently fractionated over two solid-phase extraction (SPE) cartridges. The polar extract was fractionated using weak anion-exchange (WAX; aminopropyl) and weak cation-exchange cartridges (WCX; carboxylic acid), while the apolar extract was fractionated using weak anion-exchange (referred to as AMP to differentiate from the polar arm; aminopropyl) and reversed-phase C18 cartridges (C18). See **Supplemental Section 1.7** and **Supplemental Figures S1-2** for further details. The resulting 15 SPE fractions and remaining unfractionated extracts (referred to as “crude” extract) were analysed by (U)HPLC-HRMS(/MS). A selected subset of SPE fractions, alongside crude extract, were also analysed by 1D- & 2D-NMR spectroscopy and GC-EI-HRMS, to further expand the breadth of metabolome annotation.

2.1.5 (U)HPLC-HRMS(/MS), DI-HRMS(/MS^n) and LC fractionation

Three distinct (U)HPLC-HRMS(/MS) methods were applied in both positive and negative ionisation modes to analyse constituents of the polar and apolar crude extracts, and associated SPE fractions. Polar crude extract and polar SPE fractions were analysed by hydrophilic interaction liquid chromatography (HILIC) using an Accucore amide column (AMD; 2.1 x 100 mm, 2.6 µm solid core; Thermo Scientific), and reversed-phase liquid chromatography (RPLC) based on a Syncronis phenyl column (PHE; 2.1 x 100 mm, 1.7 µm; Thermo Scientific). Constituents of the apolar crude extract and apolar SPE fractions, meanwhile, were analysed by RPLC using an Accucore C30 column (C30) (2.1 x 100 mm, 2.6 µm solid-core particle, 150 Å; Thermo Scientific). All chromatographic separations were performed using a Dionex Ultimate 3000 liquid chromatography system. A Q Exactive mass spectrometer (Thermo Scientific), fitted with heated electrospray ionisation source, was used for HRMS(/MS) mass spectrometry analysis of metabolites eluted from LC columns. A passive flow splitting tee-piece was installed between the LC column outlet and Q Exactive inlet to facilitate simultaneous collection of 20-second-wide LC fractions and associated HRMS(/MS) data. Each fraction was collected into independent wells of a deep well plate. LC fraction collection plates were dried in a centrifugal evaporator at the end of an analysis sequence.

Initial (U)HPLC-HRMS analyses were used to create inclusion and exclusion lists (i.e. *m/z* features of interest) to direct the subsequent data dependent acquisition (DDA) of (U)HPLC-HRMS/MS data. In parallel, eluent from the LC columns were fraction-collected during mass spectral acquisition, which were then subject to extensive DI-HRMS(/MS^n).

DI-HRMS(/MS^n) analyses of resuspended LC fractions were performed using an Orbitrap Elite mass spectrometer (Thermo Scientific) using both higher energy collisional dissociation (HCD) and collision-induced dissociation (CID) at several levels of normalised collision energy (NCE). Specifically, HCD was performed at 20, 40 and 80% NCE, followed by CID at 35% NCE with multi-stage fragmentation up to MS^3. In total, 2,305 LC fractions were analysed as part of the DMA of *D. magna*. The acquisition of DI-HRMS(/MS^n) fragmentation

data was directed via a predefined list of targeted *m/z* features, derived from prior DI-HRMS analysis of the same fraction.

For detailed information on the (U)HPLC-HRMS analytical setup and LC methods, including the fractionation procedure, as well as the data acquisition sequence and computational methods used to create inclusion/exclusion lists for targeting the most informative features for fragmentation data acquisition (See **Supplemental Sections 1.8.1 and Supplemental Figure S3**). Additionally, for more details on the resuspension of LC fractions, the DI-HRMS(/MS^n) analytical setup, data acquisition sequence, and the computational methods for DI-HRMS(/MS^n) used to develop both inclusion and exclusion lists and instrument methods files that directed DI-HRMS^n data acquisition (See **Supplemental Sections 1.8.2** and **Supplemental Figure S4**).

2.1.6 (U)HPLC-HRMS(/MS) method optimisation

The PHE and AMD (U)HPLC-HRMS(/MS) methods underwent optimisation for DMA of *D. magna* aiming to maximize reproducibly detectable metabolic features while enabling reproducible fractionation for downstream analyses. The methodology for the optimisation is detailed within the **Supplemental Section 1.9, Figure S5** and **Supplemental Tables S6-S8.** The C30 (U)HPLC-HRMS(/MS) method was previously optimised for broad lipid profiling applications by Thermo Fisher Scientific, hence no further optimisation was pursued.

2.1.7 GC-EI-HRMS

GC-EI-HRMS was performed on the WAX and WCX fractions, as well as the crude polar extract, using a TriPlus RSH autosampler and TRACE 1310 gas chromatograph coupled to a Q Exactive mass spectrometer (Thermo Scientific), and an Extractabrite electron ionisation/chemical ionisation source. Further details are provided in **Supplemental Section 1.10**.

2.1.8 1D & 2D NMR spectroscopy

The WAX and WCX SPE fractions were also analysed using a combination of 1D and 2D-NMR spectroscopy experiments performed using a Bruker AVANCE III 600 MHz NMR spectrometer, equipped with a 1.7 mm TCI-Cryoprobe and operated at a proton frequency of 600.13 MHz. Each sample was measured using 1D proton nuclear overhauser effect NMR spectroscopy (1D-^{1}H-NOESY) followed by 2D homonuclear ^{1}H-^{1}H (2D-JRes and TOCSY) and heteronuclear ^{1}H-^{13}C (HSQC) experiments to support annotation. Further details are provided in **Supplemental Section 1.11**.

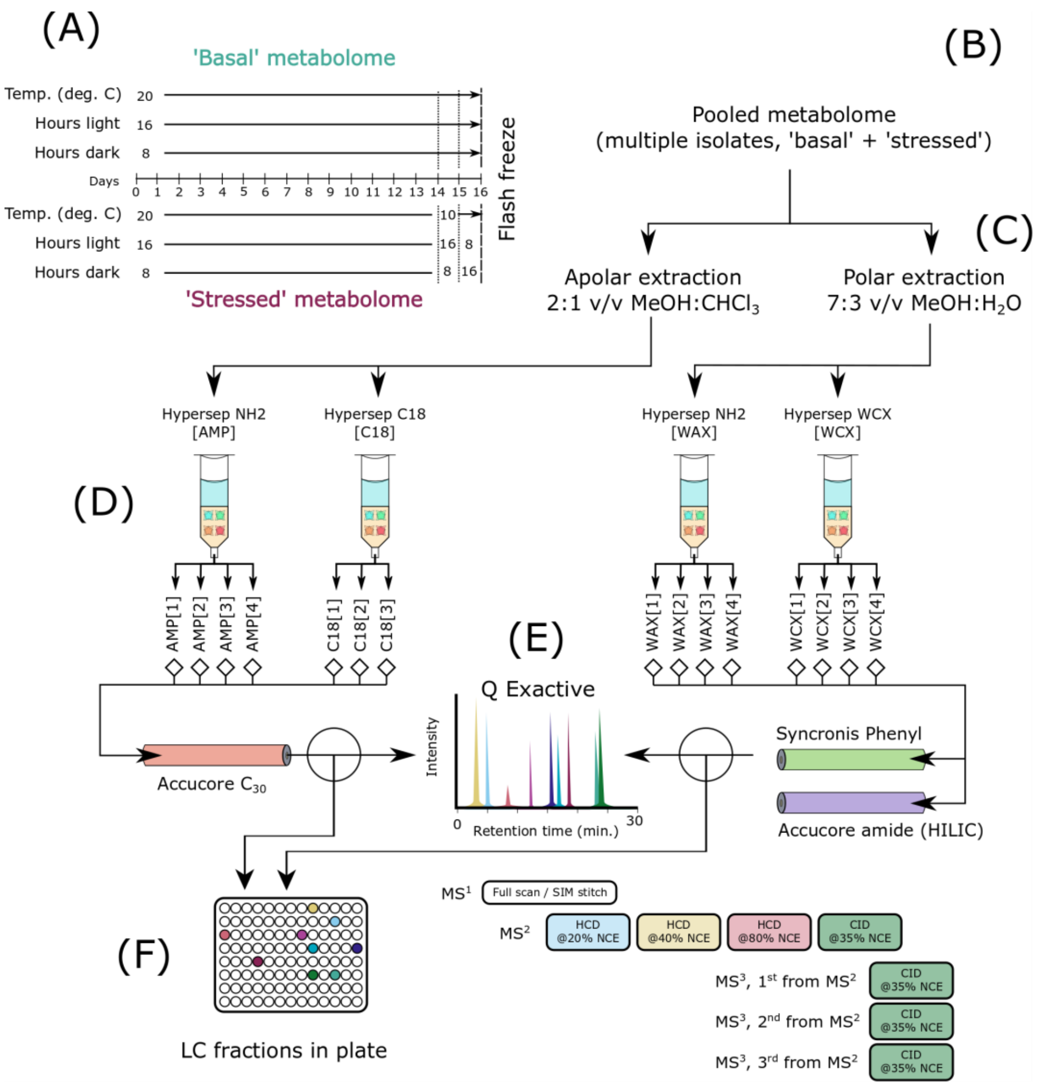

Figure 2: Deep metabolome annotation experimental workflow for the physicochemical separation and measurement of *D. magna* metabolites. *A)* *Culturing: Culturing of ten genotypically distinct strains of a model organism under normal (basal) and stressed conditions was used to ensure a wide range of metabolites were present in* ***B)*** *the pooled sample. For the DMA of D. magna this involved flash freezing in liquid nitrogen to quench metabolism, followed by homogenisation and pooling into a single sample.* ***C)*** *Liquid phase extraction: Two distinct liquid phase extractions were then performed on the pooled homogenate from step B), for 'polar' and 'apolar' metabolites.* ***D)*** *Solid phase extraction: Four types of solid phase extraction (SPE) were performed on the extracts from step C), the polar extracts using weak anion-exchange (WAX) and weak-cation exchange SPE cartridges (WCX) and apolar extracts using weak anion-exchange (AMP) and reversed-phase C18 cartridges (C18).* ***E)*** *(U)HPLC-HRMS(/MS): Three distinct (U)HPLC-HRMS(/MS) methods were applied using a Q-Exactive mass spectrometer: Accucore amide HILIC LC column (AMD) and Syncronis phenyl LC column (PHE) for analysis of metabolites from the polar arm of the workflow, and Accucore C30 LC column (C30) for the apolar extract and any SPE fractions derived from the apolar extract.* ***F)*** *Fractionation and DI-HRMS(/MS^n): The eluate from the LC columns in step E) was fractionated into plates for subsequent extensive multiple-stage fragmentation (MS^n) analysis applied (up to MS^3), including at multiple collision energies and with technical replication.*

ALT TEXT: Graphical representation of the Deep Metabolome Annotation experimental workflow.

2.2 Computational tools and workflows for data processing, metabolite annotation and data analysis

2.2.1 Overview

An extensive computational workflow utilising the Galaxy platform has been developed to analyse the highly complex data acquired through the experimental DMA workflow. This computational workflow predominantly consists of an extensive Galaxy-based workflow, with additional annotations incorporated from external sources i.e., mzCloud, GNPS workflows, GC-EI-HRMS annotations and 1D & 2D NMR annotations. A summary is provided in **Supplemental Section S12** and **Supplemental Figure S6**.

The Galaxy workflow component generated 104 Galaxy histories (see **Supplemental Table S1** for links to corresponding Galaxy history), 60 of which were used for the analysis of the *Daphnia* samples. Each Galaxy history contains a combined SQLite database containing all annotations and relevant (average) spectra across all assays.

2.2.2 Galaxy workflow details

The Galaxy workflow (see **Figure 3**) was designed specifically to process and perform metabolite annotation across the multiple data types produced by the DMA experimental workflow, including (U)HPLC-HRMS(/MS) and DI-HRMS(/MS^n). Utilising the high level of replication achieved from the DMA experimental workflow, averaging and filtering was performed on both the (U)HPLC-HRMS(/MS) and DI-HRMS(/MS^n) data so that higher quality reproducible fragment peaks were used for multiple complementary computational approaches to metabolite annotation.

The workflow is split into five components: “Data input”, “(U)HPLC-HRMS(/MS) data processing”, “DI-HRMS(/MS^n) data processing”, “Metabolite annotation” and “Combining”. Detailed description of all steps used in the workflow can be found in **Supplemental Section 1.13 –** with individual schematics detailing the (U)HPLC-HRMS(/MS) (See **Supplemental Figure S7**) and DI-HRMS(/MS^n) fragmentation data processing (See **Supplemental Figure S7**). See also **Supplemental Table S9** for the description of all tools used and **Table 1** for the location of code repositories for each Galaxy tool and underlying software.

The Galaxy workflow incorporates both new and existing tools, e.g. existing Workflow4Metabolomics XCMS Galaxy tools [23] for (U)HPLC-HRMS peak picking and processing; DIMSpy Galaxy tools [24] (https://github.com/computational-metabolomics/dimspy-galaxy) for DI-HRMS data processing. New Galaxy tools developed for the DMA project include updated functionality from the msPurity R package [25] to filter and flag spectra, average fragmentation spectra, create MSP and SQLite files of (U)HPLC-HRMS(/MS) data, perform spectral matching, and combine metabolite annotations from multiple sources; the MSnPy python package and Galaxy tools to process DI-HRMS(/MS^n) data with both multiple-stage and multiple energy fragmentation spectral trees, perform spectral averaging across trees, and annotate and rank spectral trees with molecular formulae; and the LC Fractionation Galaxy tool which was created to combine all spectra and metabolite annotations from a DMA LC fractionation experiment. In addition, Galaxy wrappers have been created for the *in silico* fragmentation software MetFrag [26–28] and

mass spectrometry data processing and metabolite annotation software SIRIUS-CSI:FingerID [29].

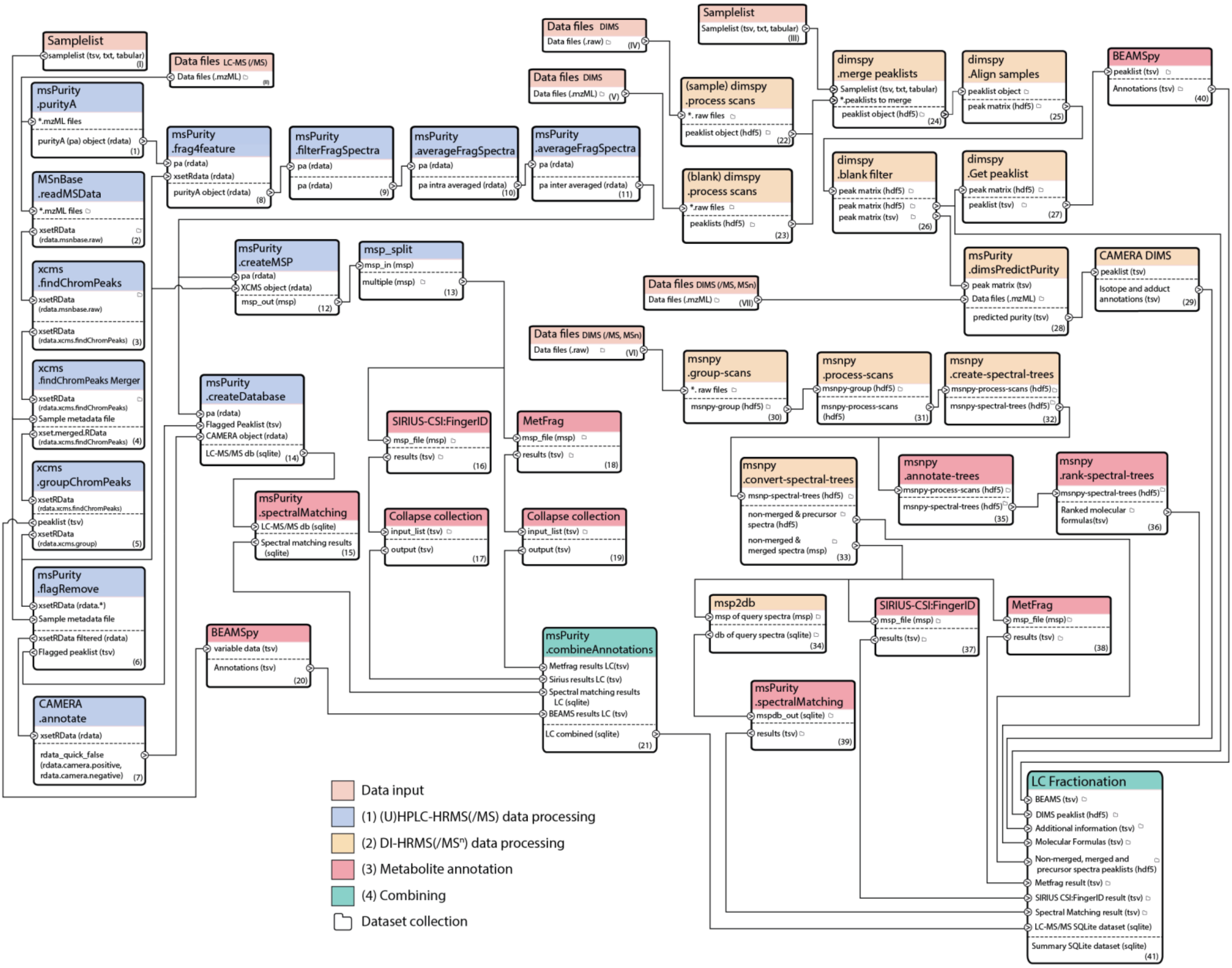

This modular, multistage workflow ensures reusability across other studies. Additionally, a public Galaxy instance (https://dma.galaxy.bham.ac.uk) was created for executing the Galaxy DMA analysis, with access to the histories, data and parameters.

Figure 3: Deep Metabolome Annotation data processing and metabolite annotation Galaxy workflow. *Components are separated by colour into "Data input", "(U)HPLC-HRMS(/MS) data processing", "DI-HRMS(/MS^n) data processing", "metabolite annotation" and "Combining". See* ***Supplemental Table S4*** *for description of each tool.*

ALT TEXT: Graphical representation of the deep metabolome annotation Galaxy workflow.

2.2.3 mzCloud library search

All annotations derived from mzCloud (https://www.mzcloud.org/) were performed programmatically in batches using mzCloud proprietary software. Each fragmentation scan was treated individually, and spectral matching was performed against the mzCloud database. The results were saved as an SQLite database with reference to both the query and library spectra of each annotation. The data were filtered to only include “endogenous” metabolites and spectral matches with dot product cosine score > 0.7.

2.2.4 GNPS library search

Fragmentation spectra were searched against the GNPS library spectra using the online workflow (https://ccms-ucsd.github.io/GNPSDocumentation/) on the GNPS website (http://gnps.ucsd.edu). The precursor ion mass tolerance was set to 0.02 Da and a MS/MS fragment ion tolerance of 0.02 Da. Additionally, spectral matches were filtered to have an error of ≤ 10 ppm between the library precursor m/z and the query precursor *m/z*, and annotations were filtered to only include spectral matching results for which the library and query spectra were derived from a mass spectrometer using the same ionisation mode.

2.2.5 GNPS molecular network analysis

A molecular network was created using the online workflow (https://ccms-ucsd.github.io/GNPSDocumentation/) on the GNPS website (http://gnps.ucsd.edu) [11]. The data was filtered to remove all fragment ions within ± 17 Da of the precursor *m/z*. Fragmentation spectra were window filtered by retaining only the top 6 fragment ions in the ± 50 Da window throughout the spectrum. Both the precursor ion mass tolerance and fragment ion tolerance were set to 0.02 Da. A network was then created where edges were filtered based on having a cosine score >0.7 and more than two matched peaks. Further, edges between two nodes were only kept in the network if each of the nodes appeared in each other's respective top 10 most similar nodes. Finally, the maximum size of a molecular family was set to 100, and the lowest scoring edges were removed from molecular families until the molecular family size was below this threshold. The fragmentation spectra in the network were then searched against GNPS’s spectral libraries. The library spectra were filtered in the same manner as the input data. All matches kept between network and library spectra were required to have a score above 0.7 and at least 2 matched peaks. Further annotation was performed using the Dereplicator tool [30] and the MS2LDA [31] workflow to determine common mass motifs.

2.2.6 Combining and summarising all annotations

Five main sources of annotations were combined into a final list of Metabolite annotations containing: Galaxy workflow annotations, GNPS workflow annotations, mzCloud annotations, NMR annotations and GC-EI-HRMS annotations. All data were combined into a single table encompassing all annotations across every assay and a final stage of filtering was performed to as described in **Supplemental Section 1.14.**

All annotations were chemically classified using ClassyFire [32].

2.2.7 Comparison to other metabolite databases

The final list of *D. magna* metabolites were compared to compound lists from KEGG [33–35], ChEBI [36], HMDB [8] and MTox700+ [37]. ChEBI was filtered for only those compounds with a known species origin. Phylotree (https://phylot.biobyte.de/index.cgi) was used to generate the species phylogenetic tree using the NCBI taxonomy. Matching was based on compounds sharing the same partial InChiKey (i.e. the first block of the InChiKey that encodes the molecular skeleton).

QIAGEN Ingenuity Pathway Analysis (IPA, QIAGEN Inc.) was used to derive metabolite-pathway associations. A metabolite list containing PubChem, HMDB and KEGG identifiers was imported into IPA. 'Metabolomics core analysis' was then conducted using all mapped metabolites, with the IPA database serving as a reference set.

2.3 Assessment of the computational and experimental DMA workflow with metabolite reference standards

Metabolite reference standards (see **Supplemental Table S11)** were analysed to evaluate the effectiveness of the overall DMA workflow, specifically the (U)HPLC-HRMS(/MS) component of the experimental workflow, including extraction, chromatographic separation, and mass spectrometric analysis. The same computational workflow was applied as used for the *D. magna* samples. Next, the measured metabolite reference standards were compared to the expected annotations, for each assay, by matching the partial InChiKey (i.e., first block).

2.4 Deep metabolome annotation database (DMAdb)

The DMA database (DMAdb) was implemented using a Python-Django framework and consists of a web portal and underlying database designed for data management and analysis. While established for the *D. magna* results, it also serves as a prototype for future DMA projects. The DMAdb allows users to organise DMA experiments within the ISA framework, supports interactions with Galaxy, enabling the metabolite annotations to be viewed and searched through a graphical user interface. The codebase for the DMAdb web portal was developed as three Django applications (django-gfiles, django-galaxy and django-mogi) specifically designed for metabolomics data organisation with Galaxy and the ISA framework (see https://dmadb.readthedocs.io/en/latest/).

3 Results and discussion

3.1 (U)HPLC-HRMS(/MS) method optimisation

The PHE and AMD (U)HPLC-HRMS(/MS) methods were optimized to improve detection of reliable metabolic features and support reproducible fractionation. Full details and supporting figures are provided in **Supplemental Section 2.1, Supplemental Table S12** and **Figures S9-S26**.

3.2 Summary of all *D. magna* metabolite annotations and compound classifications

In total, 8,577 unique metabolite annotations are reported from all experimental assays (including (U)HPLC-HRMS(/MS), DI-HRMS(/MS^n), 1D- & 2D-NMR and GC-EI-HRMS), summarised in **Supplemental Table S13**. The combined annotations and compound classifications across all technologies and approaches are presented here, with further details including specifics for each measurement technology provided in **Supplemental sections 2.2-2.5.** In summary, the majority of annotations were reported for the (U)HPLC-HRMS(/MS) and DI-HRMS(/MS^n) datasets, observing 8,528 unique annotations versus just four unique metabolites for GC-EI-HRMS and 3 for 1D- & 2D-NMR (see Venn diagram in **Supplemental Figure S27**). This can be explained in part by the relative sizes of each dataset.

Compound classification via ClassyFire was possible for 8,324 (97%) metabolites to at least the level of “Superclass”. The remaining unclassified metabolites had either incompatible SMILES for ClassyFire or lacked a SMILES annotation from PubChem. See **Figure 4A** for a treemap summarising all annotation superclasses and classes, demonstrating the diverse biochemical space observed. The chemical space was further explored using principal component analysis (PCA) of the PubChem molecular fingerprints, showing broad clustering of metabolites based on their structure, with no obvious outliers (see **Figure 4B**).

The most common “superclass” observed was for lipids and lipid-like molecules (3,391 uniquely annotated metabolites) followed by organic acids and derivatives (2,223); organoheterocyclic compounds (677); organic oxygen compounds (604); benzenoids (530); phenylpropanoids and polyketides (329); organic nitrogen compounds (230); nucleosides, nucleotides, and analogues (193); organosulfur compounds (45); alkaloids and derivatives (39); hydrocarbons (21); organophosphorus compounds (17); lignans, neolignans and related compounds (15); and five other “superclasses” with six or fewer annotated metabolites. A total of 253 metabolites could not be classified to a “superclass” level.

The most common “class” classification observed was for carboxylic acids and derivatives (1,809) followed by fatty acyls (984); glycerophospholipids (828); organooxygen compounds (598); glycerolipids (588); prenol lipids (446); benzene and substituted derivatives (356); sphingolipids (292); steroids and steroid derivatives (235); organonitrogen compounds (230); peptidomimetics (171); phenols (78); indoles and derivatives (69); macrolides and analogues (69); organic sulfuric acids and derivatives (68); purine nucleosides (52); and 185 other “classes" with 49 or fewer annotated metabolites. A total of 278 metabolites could not be classified to a “class” level.

The most common “subclass” classification was for amino acids, peptides, and analogues (1,711) followed by triradylcglycerols (450); carbohydrates and carbohydrate conjugates (397); glycerophosphocholines (334); glycerophosphoethanolamines (295); fatty acids and conjugates (253); fatty acid esters (150); glycosphingolipids (143); fatty amides (141); fatty alcohols (133); amines (131); ceramides (103); fatty acyl glycosides (101); linoleic acids and derivatives (93) and 343 other subclasses with 93 or less annotated metabolites. A total of 873 compounds could not be classified to a “subclass” level. The top 12 most common superclasses, classes and subclasses that have been annotated are shown in **Figure 4C-E.**

We note that whilst the *Daphnia* were starved of their usual algae food source for 48 hours prior to freezing for sampling, we still observe annotations for compound superclasses that are primarily derived from plant origin (e.g. the 329 phenylpropanoids and polyketides and the 39 alkaloids and derivatives). Whilst some of these metabolites could potentially be endogenous in *Daphnia (*e.g. biosynthesis within the animal kingdom is known [38]), a more plausible explanation may be that the gut of the *Daphnia* was not entirely free of algae (and any associated microbiome), and the plant-specific metabolites were still being metabolised. Another possibility is that these are potentially false positive annotations, where the actual endogenous *Daphnia* metabolite is structurally similar enough to the plant derived metabolite annotation. The use of reference standards would be required to definitively rule this out.

(U)HPLC-HRMS(/MS) and DI-HRMS(/MS^n) annotations were predominantly derived from spectral matching, MetFrag and SIRIUS CSI:FingerID annotation approaches. SIRIUS CSI:FingerID produced the most unique annotations with MetFrag and spectral matching having similar counts of unique annotations (see **Supplemental Section 2.3 and Supplemental Figure S28** for further details). This finding should not be interpreted as identifying the most effective metabolite annotation method, as the number of annotations observed can change dramatically based on filtering criteria and the compound and spectral libraries used. Rather, this simply illustrates the origins of the annotations in this *D. magna* DMA project. The differing results between the annotation approaches does however caution against relying on only a single method, both in terms of the breadth of coverage of the tool used and the potential reliability of annotations.

By using a range of metabolite annotation approaches, the confidence in the resulting annotations can be adjusted to user preference. For example, if only annotations reported by either MetFrag or spectral matching are considered (i.e., disregarding annotations derived only from SIRIUS CSI:FingerID that may yield more false positives amongst the very large number of annotations reported), it would result in 3,883 annotations (or 3,893 if the 1D- & 2D-NMR and GC-EI-HRMS annotations are included). Alternatively, annotations can be filtered even more strictly by specifying that they should be observed with at least two of the three fragmentation data analysis approaches, resulting in 1,350 annotations (or 1,365 if all 1D- & 2D-NMR and GC-EI-HRMS annotations are also included). These subsets of annotations could be considered more reliable, though it is important to highlight that the full set of 8,577 annotations observed from all fragmentation based annotation approaches were derived from sufficiently unique fragmentation spectra to derive this high number of unique metabolite annotations. While some annotations may be less reliable (i.e., from SIRIUS CSI:FingerID only), the number of unique fragmentation spectra demonstrates the richness of the *D. magna* metabolome.

Additionally, de novo molecular formula annotation using MSnPy was performed on the DI-HRMS(/MS^n) data via the MSnPy spectral annotation functionality within the Galaxy workflow. The DI-HRMS(/MS^n) data are particularly suited to this approach due to the high level of measurement replication at different collision energies and fragmentation levels. A total of 40,240 unique molecular formulae were annotated to an MSnPy rank of 1 (32,672 derived from positive ionisation mode and 9,768 from negative mode). Ranking was based on the application of common “consistency” rules for filtering formulae and neutral losses [39], which use fragmentation tree consistency to evaluate and rank the most plausible molecular formulae. Only molecular formulae were included where there were 10 or fewer possible top-ranked candidates. It should be noted that multiple molecular formulae can share a rank of one and that, despite stringent filtering, false positives will be present. It is also worth noting that this stringent filtering will have excluded many annotations for higher-mass precursor ions, which tended to produce excessively large sets of candidate molecular formulae, thereby preventing a high number of false-positive annotations. This approach does not rely on prior knowledge of spectral libraries or compound databases and therefore provides a potentially less biased insight into the biochemistry of the metabolome, albeit limited to the level of molecular formula (i.e., non-structural) annotation.

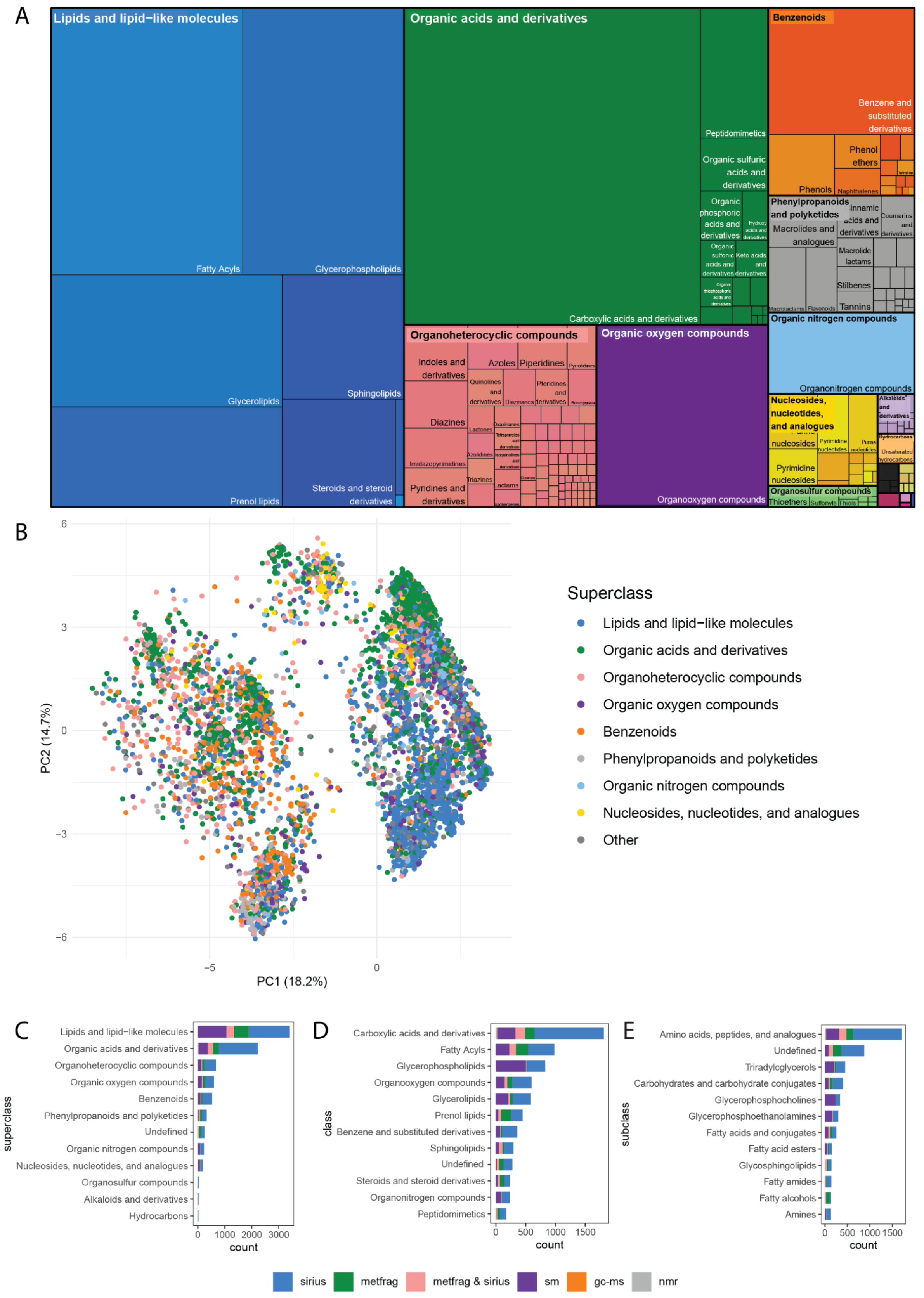

Figure 4: Compound classifications for the 8,577 D. magna deeply annotated metabolites. A) *Treemap of metabolite 'superclasses' and 'classes' for the metabolite annotations.* ***B)*** *Principal component analysis of PubChem fingerprints for all unique metabolite annotations: colour represents the superclass classification of the*

metabolite annotation. ***C)*** *Count of unique metabolite annotations for 'subclass' compound classifications. Showing the top 12 'subclasses'****,*** *colour represents the annotation approach used (annotation was either derived using SIRIUS CSI:FingerID, MetFrag, SIRIUS CSI:FingerID & MetFrag, Spectral matching, GC-EI-HRMS or 1D- & 2D-NMR).* ***D)*** *Count of unique metabolite annotations for 'class' compound classifications. Showing the top 12 'classes'****,*** *colour represents the annotation approach used (annotation was either derived using SIRIUS CSI:FingerID, MetFrag, SIRIUS CSI:FingerID & MetFrag, Spectral matching, GC-EI-HRMS or 1D- & 2D-NMR).* ***E)*** *Count of unique metabolite annotations for 'superclass' compound classifications. Showing the top 12 'superclasses', colour represents the annotation approach used (annotation was either derived using from SIRIUS CSI:FingerID, MetFrag, SIRIUS CSI:FingerID & MetFrag, Spectral matching, GC-EI-HRMS or 1D- & 2D-NMR).*

ALT TEXT: Graphs summarising the different compound classifications of metabolites annotated through deep metabolome annotation of D. magna

3.3 Metabolites and compound classes physicochemically separated by DMA experimental workflow

The extent to which the DMA experimental workflow physicochemically separated metabolites was evaluated to determine the effectiveness, or potential redundancy, of components within the workflow (see **Figure 5**).

When combining all annotations from either the polar or apolar arm of the workflow (see **Figure 5A**), both arms generated a substantial number of unique metabolite annotations (with 4,495 and 3,632 metabolites unique to the polar and apolar arms respectively, and only 450 metabolites shared). This finding highlights the importance of the extraction procedure within the workflow and the necessity of both arms of the workflow to provide a comprehensive view of the metabolome. When combining all annotations from each chromatography approach used (see **Figure 5B**), the C30 column yielded the highest number of metabolite annotations (4,082 in total, of which 3,632 were unique to the column). The PHE analysis resulted in 3,771 metabolite annotations (2,466 unique to column) while analyses performed using the AMD column resulted in the lowest number of metabolite annotations (2,226, of which 1,171 were unique to this column).

Considering the ionisation modes used (see **Figure 5C**), analysis using positive ionisation mode produced 6,099 metabolite annotations (5,078 were unique to this mode), compared to 3,490 metabolite annotations using negative ionisation (2,469 were unique). This finding evidences the need to include both ionisation modes in the DMA workflow. Unsurprisingly, for both ionisation modes the higher mass ranges (>600 Da) are dominated by lipids and lipid-like molecules, whereas for mass ranges <600 Da the organic acids and derivatives are most prominent, along with lipids and lipid-like molecules. Other superclasses with mass <600 Da include organoheterocyclic compounds; organic oxygen compounds; benzenoids; phenylpropanoids and polyketides; organic nitrogen compounds; and nucleosides, nucleotides, and analogues. **Supplemental Figure S29** shows the distributions of unique annotations against the exact mass of the annotation.

When examining each assay (see **Figure 5D**), in all cases the analysis of the crude *D. magna* extract (without SPE fractionation) resulted in the highest number of annotations compared to the individual SPE fractions. This is to be expected as the SPE fractions were intended to separate the metabolites according to their physicochemical properties and so by design will separate the metabolites across fractions. **Figure 5D** also demonstrates that lipids and lipid-like molecules can be seen to dominate the apolar arm, whereas organic acids and derivatives are the most prominent in the polar arm. This is also expected based on the chemistry of the liquid-phase extractions, solid-phase extractions and chromatography used, which favour apolar metabolites.

Figure 5E shows an UpSet plot detailing the overlap of annotations across assays (with positive and negative ionisation modes combined). The most striking observation is the dissimilar number of annotations per assay, with the SPE fractions from the AMD column providing the lowest number of annotations. This assessment could form a basis for a more streamlined, time-efficient workflow. Given that the measurements of the crude extracts yielded 577, 565 and 507 unique annotations (for apolar crude C30, polar crude AMD and polar crude PHE respectively) illustrates their valuable contribution to the DMA workflow.

To provide added confidence in the ability of the experimental and computational workflows ability to annotate the metabolome an assessment of the workflow was also made using 48 chemical reference standards covering a wide biochemical space including lipid and lipid-like molecules; organic acids and derivatives; organic oxygen compounds; organoheterocyclic compounds; nucleosides, nucleotides, and analogues; and organic nitrogen compounds. The spread of the compound classes across the experimental workflow mirrors what is observed in the *Daphnia* samples, where lipids and lipid-like molecules dominate the apolar arm and organic acids and derivatives being the most prominent in the polar arm. As the majority of metabolite reference standards were observed by the DMA workflow (89.6%), we deemed that both the experimental and computational workflows were sufficiently reliable at annotating a diverse range of metabolites to be used for annotating *D. magna* metabolome. See **Supplemental Table S11**, **Supplemental Figure S30-31** and **Supplemental Section 2.6** for further details.

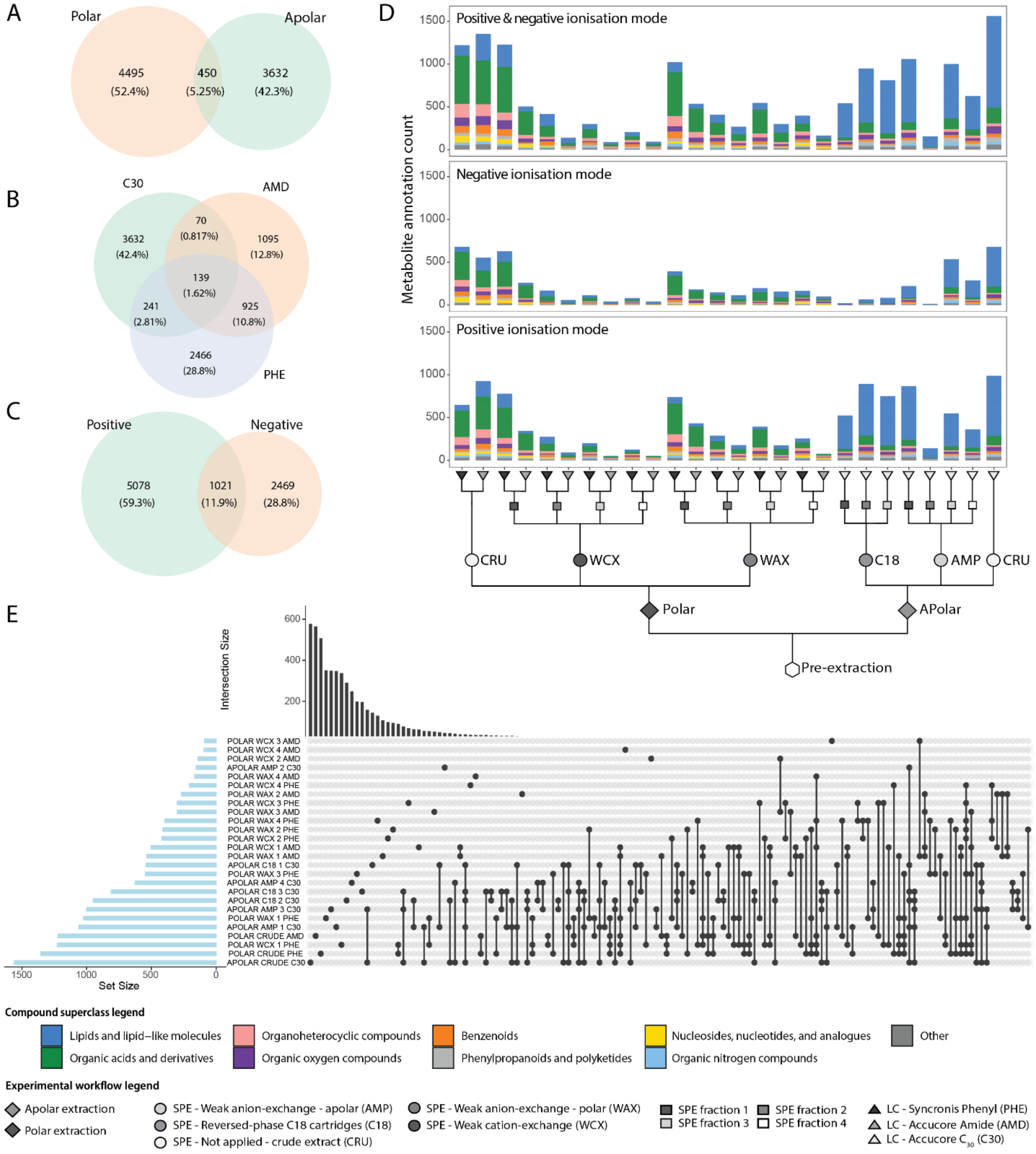

Figure 5: Contributions of extraction, SPE fractionation, LC separation and mass spectrometric ionisation methods to the number of metabolite annotations for D. magna. A) *Count of metabolite annotations across experimental workflow components: bar charts shown for positive ionisation mode, negative ionisation mode and combined positive and negative ionisation modes. Colour represents the superclass compound classification of the annotations. See bottom of figure for colour code used for compound superclass.* ***B)*** *UpSet plot summarising the overlap of metabolite annotations between assays (positive and negative assays have been combined).* ***C)*** *Venn diagram of metabolite annotations observed across extraction approaches.* ***D)*** *Venn diagram of metabolite annotations observed across all chromatography techniques.* ***E)*** *Venn diagram of metabolite annotations observed across positive and negative ionisation modes.*

ALT TEXT: Graphs summarising the number of metabolite annotations resulting from each experimental component of the D. magna deep metabolome annotation.

3.4 Comparison to other metabolite databases

The annotation results from the DMA of *D. magna* were compared to public resources of relevant metabolites from different species, as summarised in **Figure 7**. Even considering this relatively limited number of metabolites known for different organisms, it is readily apparent that some metabolites are widely shared across organisms due to the conservation of metabolism (i.e., phylometabolomics). From ChEBI, the top six species that overlap with *D. magna* DMA annotations are *Homo sapiens*, *Saccharomyces cerevisiae*, *Mus musculus, Escherichia coli, D. magna* and *Chlamydomonas reinhardtii (a single celled green algae)*. For *H. sapiens, M. musculus*, *S. cerevisiae* and *E. coli* this is in part explained by these species being the most represented within ChEBI. However, overlap with the previously known *D. magna* and algae metabolites (which are the diet of the cultured *D. magna*) adds confidence to both the existing annotations and the effectiveness of the DMA workflow.

As *D. magna* is used internationally as an ecotoxicology test species, the metabolite annotations reported using the DMA workflow were compared to those in MTox700+, a metabolite list of toxicologically-relevant metabolites derived from mammalian studies. Overlapping metabolites could be used to help interpret the toxicological perturbations measured in *D. magna* metabolomics studies. A total of 311 of 722 metabolites were matched to a full InChiKey (or 370 of 722 if using the first section of the InChiKey).

In addition to the above, the 8,577 annotated metabolites were investigated to assess coverage of known pathways. Whilst we acknowledge the limitations of this analysis due to the limited knowledge of pathways for *Daphnia*, preliminary analysis using the QIAGEN IPA software were still able to identify 56 molecular pathways ($p \leq 0.05$, Fisher's exact test), with 45 pathways having coverage over 50% (see **Supplemental Figure S32**), suggesting new knowledge of the *Daphnia* metabolome may enable deeper toxicological insights.

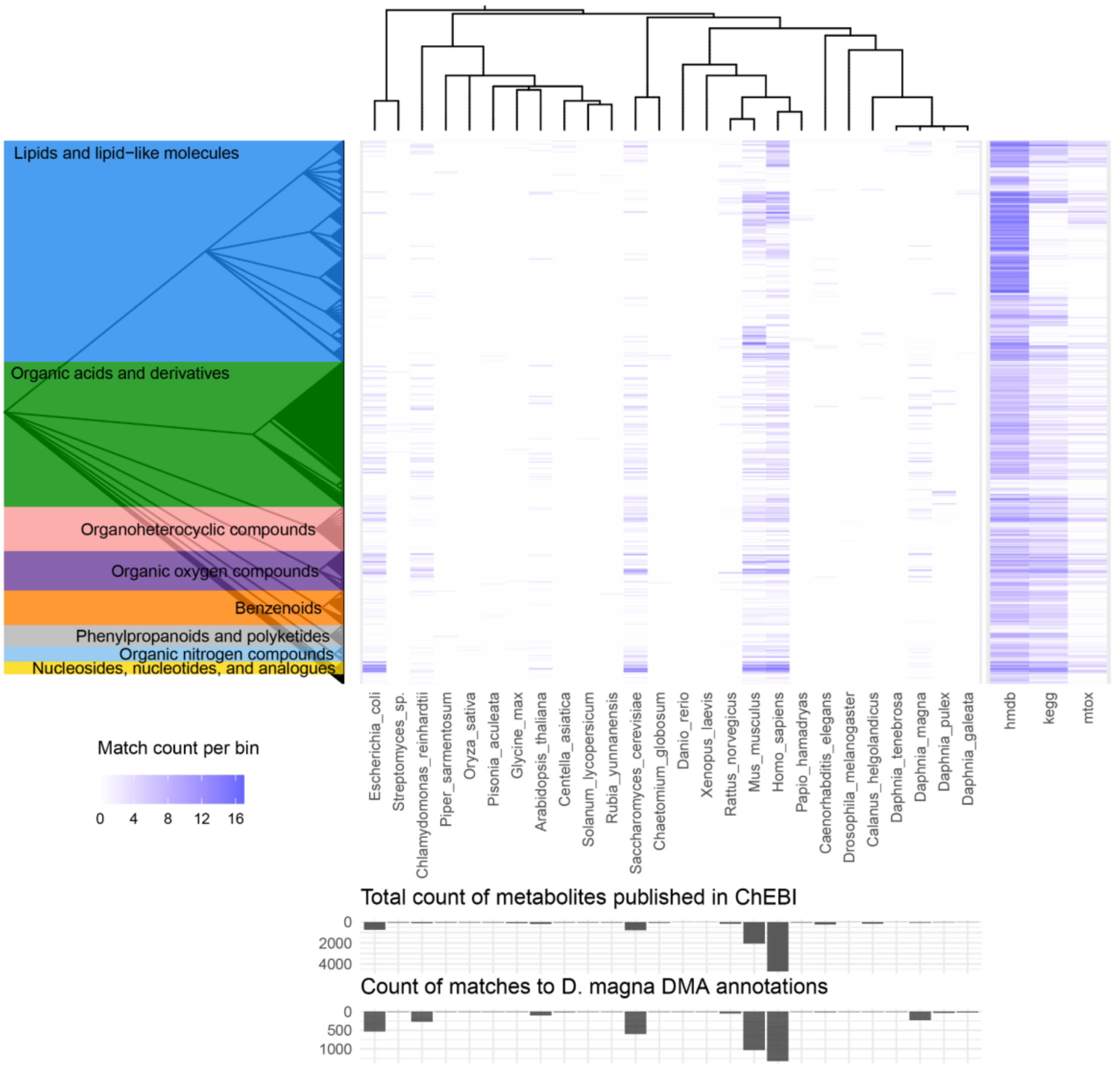

***Figure 6: Overlap of D. magna metabolite annotations reported using the DMA workflow with known metabolites from other species. Left** – all metabolite annotations from the DMA of D. magna presented in a hierarchical tree of superclasses, classes and subclasses. **Top** – phylogenetic tree of known metabolites from 26 species derived from ChEBI. **Centre** – heatmap of the counts of metabolites matched between the species derived from ChEBI with the D. magna metabolites from the DMA workflow (binned into sets of 25 compounds). **Right –** heatmap of matches observed between HMDB, KEGG and MTox700+ and the D. magna metabolite annotations reported using the DMA workflow. **Bottom –** counts of the metabolites published with ChEBI for each organism and below that the counts of the matches to DMA of D. Magna annotations.*

ALT TEXT: Graphs showing how the D. magna deep metabolome annotations overlap across other species from known metabolomes.

3.5 Molecular network analysis using GNPS

Molecular networks were generated using the GNPS network analysis workflow, with classical molecular networking, MS2LDA, Dereplicator+ and MolNetEnhancer (see **Figure 7** for summary of negative ionisation molecular networks and **Supplemental Figure S33** for positive ionisation molecular networks). These networks provide an overall picture of the diversity of the fragmentation spectra collected (and thus the diversity of metabolites observed in *Daphnia*) while not being wholly dependent on obtaining a compound or compound class annotation. The dataset collected exhibited a large diversity of fragmentation spectra (ca. 31,000 fragmentation clusters for the positive ionisation and ca. 5,000 distinct fragmentation clusters for negative ionisation mode). A similar overview can be achieved using MS2LDA mass-motifs, for which ca. 55,000 “mass motifs” are observed for positive ionisation data and ca. 3,900 for the negative ionisation data. However, only a small subset of the clusters could be annotated (e.g., ca. 1,000 clusters annotated via spectral matching for positive ionisation spectra and ca. 200 clusters annotated for negative ionisation spectra). The remaining unannotated spectra may reveal additional insights into the *Daphnia* metabolome as spectral libraries and computational approaches for annotation improve. In particular, the annotations could be improved with further integration with MetFrag and Sirius CSI:FingerID, but this was beyond the scope of this analysis. The spectral networks also demonstrate the potential for future analysis where multiple networks created from other model organism DMA projects could be compared for cross-species phylometabolomics analysis that would be driven by spectral similarity as opposed to being reliant on metabolite annotations.

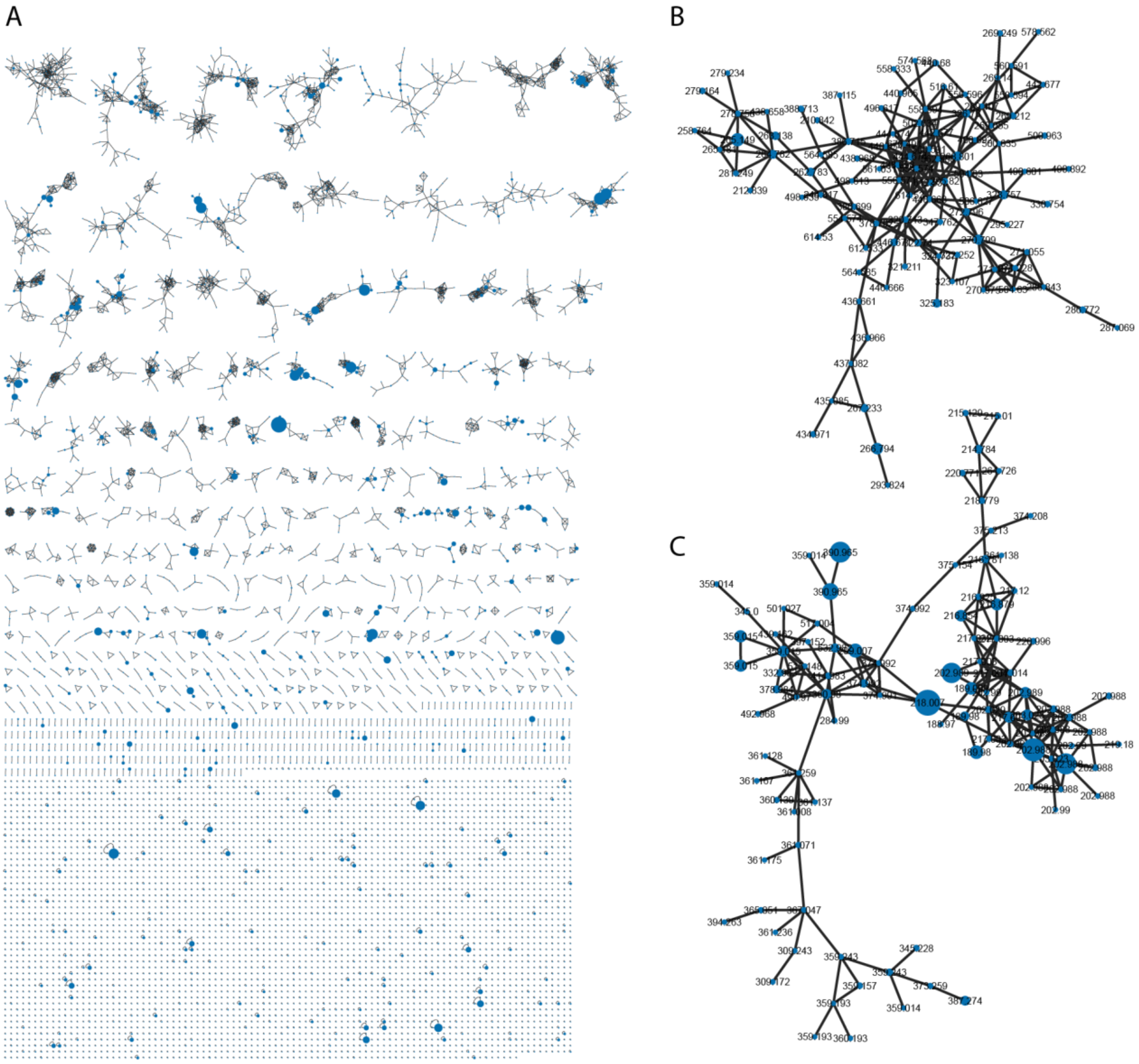

Figure 7. GNPS molecular network derived from D. magna deep metabolome annotation negative ionisation data. A) *O*verview of negative ionisation mode molecular networks generated from GNPS molecular network analysis, showing all 5,320 distinct clusters. The node size is proportional to the number of spectra that contribute to the node. The top 2 clusters (based on how many nodes were observed) are shown in more detail to highlight the precursor *m/z* associated with the node. **B)** Largest cluster observed. **C)** Second largest cluster observed.

ALT TEXT: Networks of the negative ionisation mass spectrometry fragmentation data for the D.magna deep metabolome annotations.

3.6 Deep metabolome annotation database (DMAdb)

As a means to internally organise and process the DMA annotations, and to serve as a proof of concept for how deep metabolome annotations could be disseminated in an interactive manner, DMAdb was developed. Accessible via https://dmadb.bham.ac.uk, users can access the data and results through the “ISA (investigation, study & assay)” section or the “Data & Results” section. The (U)HPLC-HRMS(/MS) and DI-HRMS(/MS^n) mass spectrometry raw data are available to download, the metabolite annotations for each assay can be explored, the overall metabolite annotations across all assays are summarised, and functionality is provided to both search by monoisotopic exact mass across the annotated compounds and to search the fragmentation spectra from all assays.

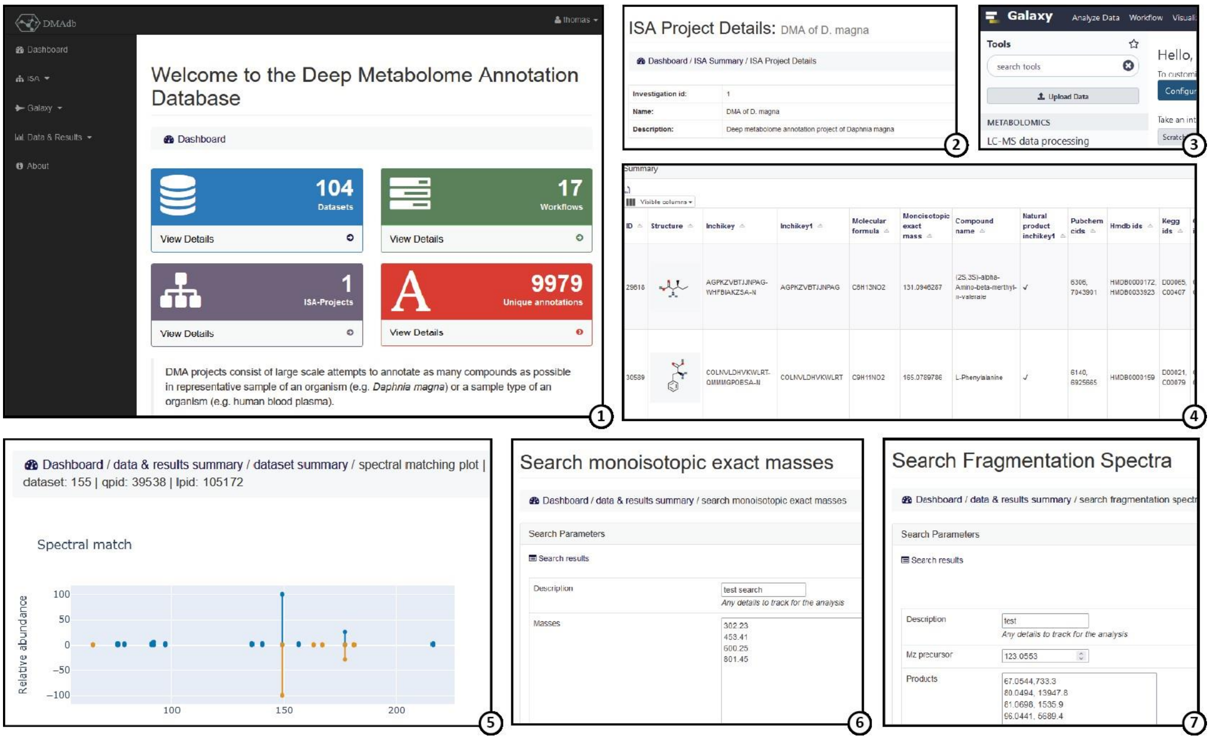

Figure 8 The Deep Metabolome Annotation database (DMAdb) - overview of its web interface and functionalities. *1) Dashboard for the DMAdb. 2) Multiple projects can be handled within the (Investigation/Study/Assay) ISA framework where relevant ontologies, protocols and processes can be recorded. 3) Data processing and annotation analysis was performed via the Galaxy platform (i.e., tools and workflows) and the resulting outputs were uploaded and stored in DMAdb. 4) The metabolite annotations and raw data files can be browsed (4), visualised (5), and searched either by one or more monoisotopic exact masses (6) or via fragmentation spectra (7).*

ALT TEXT: Summary of the different web interfaces of the Deep Metabolome Annotation database (DMAdb).

4 Conclusion

The extensive experimental and computational tools and workflows developed and applied here have generated one of the largest metabolite annotation datasets ever published and provide the first comprehensive list of metabolites thought to be present in the ecotoxicologically important model organism, *D. magna*. The reported 8,577 metabolites (1,365 to 3,893 if using more stringent filtering criteria), covering predominantly the endogenous *Daphnia* metabolites and potentially some amount of the algae food source and gut microbiome (e.g. the 329 phenylpropanoids and polyketides and the 39 alkaloids and derivatives*),* is a significant step forward in understanding the metabolic complexity of *Daphnia*. The dataset generated here also provides a large resource of mass spectrometry fragmentation data focused on a single organism, which can be used for both re-analysis and as a source of annotations when compared to other fragmentation datasets, e.g. for cross-species metabolome comparisons (phylometabolomics). Additionally, we have established Galaxy workflows and tools to process and annotate not just this dataset, but other mass spectrometry fragmentation datasets. Indeed, as many of the tools have already been made public, the benefit of the Galaxy tools developed here have already been demonstrated [40,41].

While our computational workflow is extensive, encompassing multiple techniques and approaches, we do acknowledge some challenges. In particular, the bias arising from the choice of software, software parameters, weightings, library choice and filtering approaches used and their impact on the final set of reported metabolite annotations. The lenience set for these parameters and choices does impact the reliability of the annotations, and it is anticipated that some false positive annotations are reported here, i.e., not actual endogenous *Daphnia* metabolites but structurally similar enough to generate an annotation. Purchasing thousands of metabolite reference standards to confirm the annotations is not currently possible, due to the limited commercial availability of such standards. Also, whilst steps were put in place to minimise influence of background or contaminant signals, further work could investigate more stringent criteria. As more metabolite annotations are described for model organisms, assuming that they are definitive, the reliability of the annotations from the *Daphnia* DMA project can be tested. Improvements to the computational workflow could include a more extensive use of isotopes and in-source fragmentation to filter out spurious annotations [42]; further integration of the underlying software packages into other mass spectrometry-based data analysis suites [43]; and further integration with GNPS networking tools where potentially the networks could be generated on the averaged spectra generated via msPurity and MSnPy rather than individual scans, and then incorporating the annotations generated from MetFrag and Sirius CSI:FingerID.

The experimental methods described here provide a means to obtain a single homogenous sample representing an organism's metabolome that is then characterised through extensive physicochemical separation and bioanalytical measurements. The experimental workflow developed here could be reused in full or in part, depending on the resources available. For example, where sample material is limited a subset of the SPE methods and/or (U)HPLC-HRMS(/MS) approaches could be used based on which metabolite classes are of interest, or for a more rapid DMA project the SPE component could be removed from the workflow. We acknowledge that even with this extensive workflow there are limitations in how many metabolites were able to be fragmented, reducing the number of metabolites that can be

annotated using fragmentation-based approaches. However, advancements in mass spectrometry technologies can help to address this issue, e.g. the Thermo Scientific Orbitrap ID-X Tribrid mass spectrometer is capable of extensive MS^n analysis, while current generation Time of Flight / Astral instruments support MS/MS acquisition at up to 250 Hz. Even with these limitations, the DMA workflow described here and applied to *D. magna* provides both a resource and a valuable catalyst for future deep metabolome annotation studies of model organisms.

5 Code availability

The Galaxy workflows, histories and details of each tool used in this project are available in https://dma.galaxy.bham.ac.uk/histories/list_published and https://dma.galaxy.bham.ac.uk/workflows/list_published (workflows also available to download via - https://github.com/computational-metabolomics/dmagna-dma-galaxy-workflows under GPL-3.0 licence). These resources cover the full Galaxy workflow analysis; however, re-running all analyses, particularly outside of the provided Galaxy instances, would require additional setup due both the high computational resource demands of this large dataset, as well as software updates to some of the underlying Galaxy tool since the analysis in this manuscript was performed. See **Table 1** for availability of the Galaxy tools used and developed, and **Supplemental Table S9** for details of each tool.

Table 1: Galaxy tools - code availability

Project name	Galaxy tools	Galaxy tool code home page	Underlying software code home page	Licence	Language
Pre-existing software and Galaxy tools					
MSnBase	MSnBase.readMSData	https://github.com/workflow4metabolomics/tools-metabolomics	https://www.bioconductor.org/packages/release/bioc/html/MSnbase.html	Underlying software: Artistic-2.0<br>Galaxy tool: GPL-3.0	R
XCMS	xcms.findChromPeaks<br>xcms.findChromPeaks Merger<br>xcms.groupChromPeaks	https://github.com/workflow4metabolomics/tools-metabolomics	http://bioconductor.org/packages/release/bioc/html/xcms.html<br><br>[44]	GPL (>= 2)	R
CAMERA	CAMERA<br>.Annotate	https://github.com/workflow4metabolomics/tools-metabolomics	https://www.bioconductor.org/packages/release/bioc/html/CAMERA.html<br><br>[45]	GPL (>= 2)	R
BEAMSpy	BEAMSpy	https://github.com/computational-metabolomics/beamspy-galaxy	https://github.com/computational-metabolomics/beamspy<br><br>https://more.bham.ac.uk/beams/	GPL-3.0	R
DIMSpy	dimspy.Process scans<br>dimspy.merge peaklists<br>dimspy.align samples<br>dimspy.blank filter<br>dimspy.Get peaklist	https://github.com/computational-metabolomics/dimspy-galaxy	https://github.com/computational-metabolomics/dimspy	GPL-3.0	Python
Software and / or the Galaxy tool was developed by authors					
**msPurity	msPurity.purityA<br>msPurity.flagRemove<br>msPurity.frag4feature<br>msPurity.filterFragSpectra<br>msPurity.averageFragSpectra<br>msPurity.createMSP<br>msPurity.createDatabase<br>msPurity.spectralMatching<br>msPurity.combineAnnotations<br>msPurity.dimsPredictPurity	https://github.com/computational-metabolomics/mspurity-galaxy/	https://www.bioconductor.org/packages/release/bioc/html/msPurity.html<br><br>[25]	GPL-3.0	R

**MSnPy	MSnPy.group-scans<br>MSnPy.process-scans<br>MSnPy.create-spectral-trees<br>MSnPy.annotate-trees<br>MSnPy.convert-spectral-trees	https://github.com/computational-metabolomics/msnpy-galaxy	https://github.com/computational-metabolomics/msnpy	GPL-3.0	Python
**msp2db	msp2db	https://github.com/computational-metabolomics/dmatools-galaxy	https://github.com/computational-metabolomics/msp2db	GPL-3.0	Python
**CAMERA DIMS	CAMERA DIMS	https://github.com/computational-metabolomics/dmatools-galaxy	https://github.com/computational-metabolomics/cameraDIMS	GPL (>= 2)	R
*SIRIUS CSI:FingerID	SIRIUS CSI:FingerID	https://github.com/computational-metabolomics/sirius-csifingerid-galaxy/	https://bio.informatik.uni-jena.de/software/sirius/<br><br>[29]	Underlying software: GNU AGPL<br>Galaxy tool: GPL-3.0	Java (and python for Galaxy wrapper)
*MetFrag	MetFrag	https://github.com/computational-metabolomics/metfrag-galaxy/	https://ipb-halle.github.io/MetFrag/<br><br>[26–28]	Underlying software: GPL (>= 2)<br>Galaxy tool: GPL-3.0	Java (and python for Galaxy wrapper)
**LC fractionation processor	LC fractionation processor	https://github.com/computational-metabolomics/lcfrac-galaxy	https://github.com/computational-metabolomics/lcfrac-galaxy<br><br>(all functionality within Galaxy tool)	GPL-3.0	Python
**[§]deconrank	deconrank	https://github.com/computational-metabolomics/dmatools-galaxy	https://github.com/computational-metabolomics/deconrank	GPL-3.0	Python

Footnotes: All tools and software described in table are operating system platform independent. *Galaxy tool developed by authors. **New underlying software and Galaxy tool developed by authors. [§]Not used directly in the annotation workflows but was used in the "directed acquisition workflows" described in **Supplemental Section 1.8.** Each of the new tools provided here provide example test data to trial the functionality and analysis.

R (v4.4.3) was used for summarising the annotations and generating **Figures 5-7** and the supplemental summary figures. The code used for this is available via Github (https://github.com/computational-metabolomics/dmagna-dma-paper) available under the GPL-3.0 licence – operating system – platform independent. All packages requirements are detailed with the repository and we summarise some of the key packages used here: The R package ggplot2 (v3.5.2) [46] was used throughout the analysis for the generation of plots; UpSetR (v1.4.0) [47] was used to generate UpSet plots; VennDiagram (v1.7.3) was used to generate Venn diagrams; Treemap (v2.4.4) was used to generate treemaps; ggtree (v3.14.0) [48], ape (v5.8.1) [49] and aplot (v0.2.8) [50] were used to generate the plots comparing the DMA of *D. magna* metabolites to the phylogenetic tree of relevant species and map to relevant databases and resources; ChemmineR [51] (v3.58.0) was used to extract the PubChem fingerprints from the PubChem mol files from PubChem [52], principal component analysis (PCA) was then performed on these fingerprints with the R "prcomp" function.

Additionally, the packages and code used to create the DMAdb site (https://dmadb.bham.ac.uk) are freely available (Project name: DMAdb; Project home page: https://dmadb.readthedocs.io/en/latest/getting-started.html; Operating system(s): Platform independent; Programming language: Python; License: GPL-3.0).

6 Additional files

Supplemental information is available in the accompanying Word (.docx) file, with larger tables provided separately in the Excel (.xlsx) file (**Supplemental Tables S1, S2, S11** and **S13**).

Supplemental section 1: Materials and methods - further details

- **1.1: Summary of assays and files**
- **1.2: Chemicals**
- **1.3: Solvents and solutions**
- **1.4: Consumables**
- **1.5: *D. magna* culturing and sample preparation**
- **1.6: Metabolite extraction from homogenised D. magna biomass**
- **1.7: Solid phase extraction-based fractionation of metabolite extracts**
- **1.8: DMA (U)HPLC-HRMS(/MS), DI-HRMS(MS^n) and LC fractionation**
- **1.9: (U)HPLC-HRMS(/MS) method optimisation**
- **1.10: GC-EI-HRMS**
- **1.11: 1D- & 2D-NMR**
- **1.12: DMA computational workflow overview**
- **1.13: DMA Galaxy workflow**
- **1.14: Combining and summarising all annotations**
- **1.15: Assessment of the computational and experimental DMA workflow with metabolite reference standards**

Supplemental section 2: Results - further details

- **2.1: (U)HPLC-HRMS(/MS) method optimisation**
- **2.2: Summary of all DMA of *D. magna* annotations**
- **2.3: (U)HPLC-HRM(/MS) and DI-HRMS(MS^n) derived metabolite annotations**
- **2.4: GC-EI-HRMS derived metabolite annotations**
- **2.5: NMR derived metabolite annotations**
- **2.6: Assessment of the computational and experimental DMA workflow with metabolite reference standards**
- **2.7: Pathway analysis**
- **2.8: Molecular network analysis using GNPS**

Supplemental Tables:

- **Table S1:** Assay Summary
- **Table S2:** (U)HPLC-HRMS(/MS) DI-HRMS(MS^n) data files
- **Table S3:** *D. magna* cultures
- **Table S4:** High-hardness COMBO and modified high hardness COMBO medium
- **Table S5:** Bold's basal medium
- **Table S6:** Liquid chromatography systems utilised in optimisation of (U)HPLC-HRMS(/MS) methods

- **Table S7:** Mass spectrometer operational parameters for optimisation of (U)HPLC-HRMS(/MS) methods
- **Table S8:** Liquid chromatography operational parameters for optimisation of (U)HPLC-HRMS(/MS) methods
- **Table S9:** Summary of Galaxy tools
- **Table S10:** Summary of fragmentation spectra used for spectral matching with msPurity
- **Table S11:** Metabolite reference standard summary
- **Table S12:** Median and interquartile range of retention times for RDMFs recorded in DMA (U)HPLC-HRMS/MS method optimisation experiments
- **Table S13:** *D. magna* metabolite annotation summary
- **Table S14:** GC-EI-HRMS derived metabolite annotations
- **Table S15:** NMR derived metabolite annotations

Supplemental figures (methods)

- **Figure S1**: Solid phase extraction-based fractionation of *D. magna* polar extract (WAX, weak anion-exchange; WCX, weak cation-exchange)
- **Figure S2**: Solid phase extraction-based fractionation of *D. magna* apolar extract (C18, a reversed phase-based fractionation procedure; AMP, a weak anion-exchange-based fractionation procedure
- **Figure S3:** Overview of the data acquisition workflow applied for (U)HPLC-HRMS(/MS) analysis and time-based fractionation of DMA samples
- **Figure S4:** Overview of the data acquisition workflow applied for DI-HRMS(/MS^n) analysis of the DMA re-suspended LC fractionation samples.
- **Figure S5**: Sample preparation for (U)HPLC-HRMS(/MS) method optimisation
- **Figure S6:** Overview of computational analysis of DMA (U)HPLC-HRMS(/MS) and DI-HRMS(MS^n) LC fractionation experiments
- **Figure S7:** (U)HPLC-HRMS(/MS) data processing schematic for msPurity and XCMS
- **Figure S8:** DI-HRMS(MS^n) data processing schematic for MSnPy

Supplemental figures ((U)HPLC-HRMS(/MS) method optimisation results)

- **Figure S9-26:** Multiple figures detailing the (U)HPLC-HRMS(/MS) method optimisation. Includes 2-dimensional density plot of reproducibly detectable metabolic features (RDMFs) for each method assessed and summary plots of the counts of RDMFS across the different methods.

Supplemental figures (*D. magna* annotation results)

- **Figure S27:** Venn diagram of metabolite annotations observed for 1D- & 2D-NMR, GC-EI-HRMS and (U)HPLC-HRMS(/MS) and DI-HRMS(MS^n) measurement techniques
- **Figure S28:** Venn diagram of metabolite annotations observed across computational annotation approach used.

- **Figure S29:** Distribution of unique metabolite annotations across monoisotopic exact mass
- **Figure S30:** Assessment of the DMA experimental and computational workflow
- **Figure S31:** Summary of which annotation approach was able to identify each metabolite standard
- **Figure S32**: Summary of the top canonical pathways derived using QIAGEN Ingenuity Pathway Analysis (IPA) for all annotations obtained from the DMA of D. magna
- **Figure S33:** GNPS spectral network analysis (positive ionisation mode).

7 Abbreviations

(U)HPLC-HRMS(/MS)	(Ultra)high-performance liquid chromatography-high resolution tandem mass spectrometry
1D- & 2D-NMR	1- and 2-dimensional nuclear magnetic resonance
AMD	Accucore Amide liquid chromatography column
AMP	Weak anion-exchange SPE cartridges (apolar arm of workflow)
C18	Reversed-phase C18 SPE cartridges
C30	Accucore C30 RPLC column (C30)
CID	Collision-induced dissociation
DDA	Data dependent acquisition
DI-HRMS(/MS^n)	Direct infusion-high resolution mass spectrometry (with multiple-stage fragmentation)
DMA	Deep metabolome annotation
DMAdb	Deep metabolome annotation database
GC-EI-HRMS	Gas chromatography-electron ionisation-high resolution mass spectrometry
HCD	Higher energy collisional dissociation
HILIC	Hydrophilic interaction liquid chromatography
HRMS(/MS)	High resolution mass spectrometry (with tandem mass spectrometry)
NCE	Normalised collision energy
RPLC	Reverse-phase liquid chromatography
PHE	Syncronis Phenyl liquid chromatography column
SPE	Solid-phase extraction
WAX	Weak anion-exchange SPE cartridges (polar arm of workflow)
WCX	Weak-cation exchange SPE cartridges

8 Acknowledgements

We would like to thank several current and former Thermo Fisher Scientific scientists for their helpful advice, including Martin Hornshaw, David Peake, Amanda Souza, Ioanna Ntai and Tim Stratton, as well as Anthony Edge and Alex Adam who co-supervised MRJ's iCASE PhD studentship. We are also grateful to Peter Li from *Gigascience* who co-supervised TNL's iCASE PhD studentship and provided early guidance related to Galaxy and database development. We also thank John Colbourne for his insightful feedback provided throughout his co-supervision of both MRJ's and TNL's PhDs. We thank Karl Burgess for his oversight

as Head of Metabolomics at Glasgow Polyomics, University of Glasgow where the GC-EI-HRMS measurements were performed. Thanks also to the Galaxy community for helpful discussions and contributions regarding the Galaxy tools and workflow development, and to Dominic Wilson and Andrew Edmonds from the Research Software Group, part of Advanced Research Computing at the University of Birmingham (https://www.birmingham.ac.uk/bear-software) for help with setting up the IT infrastructure for data storage, DMAdb and the Galaxy platform. Finally, we are particularly grateful to Clement Heude (no longer in the field) and the Biomolecular NMR Facility team at the University of Birmingham for the NMR measurements.

9 Author contributions

MRJ: Investigation [lead], Methodology [lead], Conceptualization [equal], Formal analysis [equal], Software [equal], Visualisation [equal], Data curation [equal], Writing – original draft [lead] and Writing – review & editing [equal].

TNL: Software [lead], Formal analysis [lead], Visualisation [lead], Data curation [lead], Writing – original draft [lead], Writing – review & editing [equal], Conceptualization [equal], Investigation [supporting] and Methodology [supporting].

AJC: Investigation [supporting], Methodology [supporting], and Writing – review & editing [supporting].

ES: Formal analysis [supporting], Visualisation [supporting], and Writing – review & editing [supporting].

SW: Investigation [supporting], Methodology [supporting], Formal analysis [supporting] and Writing – review & editing [supporting].

RM: Supervision [supporting] and Writing – review & editing [supporting].

WD: Supervision [supporting], Conceptualization [supporting], and Writing – review & editing [supporting].

RJMW: Supervision [equal]; Software [equal], Conceptualization [equal], Writing – original draft [equal], Writing – review & editing [equal]; Methodology [supporting], Formal analysis [supporting] and Data curation [supporting].

MRV: Supervision [lead]; Conceptualization [equal]; Writing – original draft [equal] and Writing – review & editing [equal].

10 Funding

This work was supported financially through two NERC CASE PhD studentships at the University of Birmingham with GigaScience (NE/L002493/1 – CENTA: Central England NERC Training Alliance; TNL) and Thermo Fisher Scientific (NE/J017442/1; MRJ). The work was also funded through the Wellcome Trust research grant “MetaboFlow” (202952/Z/16/Z; TNL, MRJ, RJMW, MRV) and funding from the European Union’s Horizon 2020 Research and Innovation programme under Grant Agreement No. 965406 “PrecisionTox” (TNL, MRJ,

RJMW, ES, MRV). This output reflects only the authors' views and the European Union cannot be held responsible for any use that may be made of the information contained therein.

11 Data availability

All raw (U)HPLC-HRMS(/MS) and DI-HRMS(/MSn) data and selected annotations supporting the results of this article are available through MetaboLights (MTBLS2273). Additionally, the mass spectrometry files containing mass spectrometry gas-phase fragmentation spectra used for the GNPS analysis of the *D. magna* sample (and not equilibration, blank or reference standard samples) are also available through GNPS MassIVE repository (MSV000094957). The assay format was simplified for MetaboLights and MassIVE into four assays (apolar positive, apolar negative, polar positive and polar negative).

Mass spectral libraries used for the Galaxy workflow are available via github (github.com/computational-metabolomics/msp2db/releases/tag/v0.0.14-mona-23042021).

The DMAdb web portal can also be used to access all of the raw and processed (U)HPLC-HRMS(/MS) and DI-HRMS(/MSn) data as well as viewing and searching the fragmentation spectra and metabolite annotations.

12 Competing interests

The authors declare no competing interests.

13 References

1. Keane TM, Goodstadt L, Danecek P, White MA, Wong K, Yalcin B, et al.. Mouse genomic variation and its effect on phenotypes and gene regulation. *Nature*. 2011; doi: 10.1038/nature10413.

2. Dunham I, Kundaje A, Aldred SF, Collins PJ, Davis C a., Doyle F, et al.. An integrated encyclopedia of DNA elements in the human genome. *Nature*. 2012; doi: 10.1038/nature11247.

3. Hood L, Rowen L. The human genome project: big science transforms biology and medicine. *Genome Med*. 2013; doi: 10.1186/gm483.

4. The 1000 Genomes Project Consortium, Corresponding authors, Auton A, Abecasis GR, Steering committee, Altshuler DM, et al.. A global reference for human genetic variation. *Nature*. 2015; doi: 10.1038/nature15393.

5. Moco S, Buescher JM. Metabolomics: going deeper, going broader, going further. *Cell-Wide Identification of Metabolite-Protein Interactions*. Springer; :155–78 2022;

6. Puris E, Kouřil Š, Najdekr L, Auriola S, Loppi S, Korhonen P, et al.. Metabolomic, Lipidomic and Proteomic Characterisation of Lipopolysaccharide-induced Inflammation Mouse Model. *Neuroscience*. 2022; doi: 10.1016/j.neuroscience.2022.05.030.

7. Ramabulana A-T, Petras D, Madala NE, Tugizimana F. Mass spectrometry DDA parameters and global coverage of the metabolome: Spectral molecular networks of momordica cardiospermoides plants. *Metabolomics*. 2023; doi: 10.1007/s11306-023-01981-4.

8. Wishart DS, Tzur D, Knox C, Eisner R, Guo AC, Young N, et al.. HMDB: the Human Metabolome Database. *Nucleic acids research*. 2007; doi: 10.1093/nar/gkl923.

9. Horai H, Arita M, Kanaya S, Nihei Y, Ikeda T, Suwa K, et al.. MassBank: A public repository for sharing mass spectral data for life sciences. *J Mass Spectrom*. Wiley Online Library; 2010; doi: 10.1002/jms.1777.

10. Kind T, Liu K-H, Lee DY, DeFelice B, Meissen JK, Fiehn O. LipidBlast in silico tandem mass spectrometry database for lipid identification. *Nat Methods*. 2013; doi: 10.1038/nmeth.2551.

11. Wang M, Carver JJ, Phelan VV, Sanchez LM, Garg N, Peng Y, et al.. Sharing and community curation of mass spectrometry data with Global Natural Products Social Molecular Networking. *Nat Biotechnol*. 2016; doi: 10.1038/nbt.3597.

12. Haug K, Salek RM, Conesa P, Hastings J, de Matos P, Rijnbeek M, et al.. MetaboLights--an open-access general-purpose repository for metabolomics studies and associated meta-data. *Nucleic acids research*. 2013; doi: 10.1093/nar/gks1004.

13. Sud M, Fahy E, Cotter D, Azam K, Vadivelu I, Burant C, et al.. Metabolomics Workbench: An international repository for metabolomics data and metadata, metabolite standards, protocols, tutorials and training, and analysis tools. *Nucleic Acids Res*. 2015; doi: 10.1093/nar/gkv1042.

14. Viant MR, Kurland IJ, Jones MR, Dunn WB. How close are we to complete annotation of metabolomes? *Current Opinion in Chemical Biology*. Elsevier BV; 2017; doi: 10.1016/j.cbpa.2017.01.001.

15. Edison AS, Hall RD, Junot C, Karp PD, Kurland IJ, Mistrik R, et al.. The time is right to focus on model organism metabolomes. *Metabolites*. 2016; doi: 10.3390/metabo6010008.

16. . The Precision Toxicology initiative. *Toxicology Letters*. 2023; doi: 10.1016/j.toxlet.2023.05.004.

17. Weismann A. Beiträge zur Naturgeschichte der Daphnoiden. W. Engelmann;

18. Ebert D. Introduction to the ecology, epidemiology, and evolution of parasitism in Daphnia. National Center for Biotechnology Information (US);

19. Ebert D. Daphnia as a versatile model system in ecology and evolution. *EvoDevo*. 2022; doi: 10.1186/s13227-022-00199-0.

20. Lampert W. Daphnia: Model herbivore, predator and prey. *Polish Journal of Ecology*. 54:607–202006;

21. Colbourne JK, Pfrender ME, Gilbert D, Thomas WK, Tucker A, Oakley TH, et al.. The ecoresponsive genome of Daphnia pulex. *Science (New York, NY)*. 2011; doi: 10.1126/science.1197761.

22. Afgan E, Baker D, Batut B, van den Beek M, Bouvier D, Čech M, et al.. The Galaxy platform for accessible, reproducible and collaborative biomedical analyses: 2018 update. *Nucleic Acids Research*. 2018; doi: 10.1093/nar/gky379.

23. Giacomoni F, Le Corguille G, Monsoor M, Landi M, Pericard P, Petera M, et al.. Workflow4Metabolomics: a collaborative research infrastructure for computational metabolomics. *Bioinformatics*. 2015; doi: 10.1093/bioinformatics/btu813.

24. Southam AD, Weber RJM, Engel J, Jones MR, Viant MR. A complete workflow for high-resolution spectral-stitching nanoelectrospray direct-infusion mass-spectrometry-based metabolomics and lipidomics. *Nature Protocols*. 2017; doi: 10.1038/nprot.2016.156.

25. Lawson TN, Weber RJM, Jones MR, Chetwynd AJ, Rodriguez Blanco GA, Di Guida R, et al.. msPurity: Automated evaluation of precursor ion purity for mass spectrometry based fragmentation in metabolomics. *Anal Chem*. 2017; doi: 10.1021/acs.analchem.6b04358.

26. Ruttkies C, Schymanski EL, Wolf S, Hollender J, Neumann S. MetFrag relaunched: Incorporating strategies beyond in silico fragmentation. *J Cheminform*. Springer International Publishing; 2016; doi: 10.1186/s13321-016-0115-9.

27. Ruttkies C, Neumann S, Posch S. Improving MetFrag with statistical learning of fragment annotations. *BMC Bioinformatics*. 2019; doi: 10.1186/s12859-019-2954-7.

28. Wolf S, Schmidt S, Müller-Hannemann M, Neumann S. In silico fragmentation for computer assisted identification of metabolite mass spectra. *BMC Bioinformatics*. BioMed Central Ltd; 2010; doi: 10.1186/1471-2105-11-148.

29. Dührkop K, Fleischauer M, Ludwig M, Aksenov AA, Melnik AV, Meusel M, et al.. SIRIUS 4: a rapid tool for turning tandem mass spectra into metabolite structure information. *Nat Methods*. 2019; doi: 10.1038/s41592-019-0344-8.

30. Mohimani H, Gurevich A, Mikheenko A, Garg N, Nothias L-F, Ninomiya A, et al.. Dereplication of peptidic natural products through database search of mass spectra. *Nature Chemical Biology*. 2017; doi: 10.1038/nchembio.2219.

31. Wandy J, Zhu Y, van der Hooft JJJ, Daly R, Barrett MP, Rogers S. Ms2lda.org: web-based topic modelling for substructure discovery in mass spectrometry. Stegle O, editor. *Bioinformatics*. 2018; doi: 10.1093/bioinformatics/btx582.

32. Djoumbou Feunang Y, Eisner R, Knox C, Chepelev L, Hastings J, Owen G, et al.. ClassyFire: automated chemical classification with a comprehensive, computable taxonomy. *J Cheminform*. 2016; doi: 10.1186/s13321-016-0174-y.

33. Kanehisa M, Goto S. KEGG: Kyoto Encyclopedia of Genes and Genomes. *Nucleic Acids Research*. Vol. 28:27–302000;

34. Kanehisa M. Toward understanding the origin and evolution of cellular organisms. *Protein Science*. 2019; doi: 10.1002/pro.3715.

35. Kanehisa M, Furumichi M, Sato Y, Kawashima M, Ishiguro-Watanabe M. KEGG for taxonomy-based analysis of pathways and genomes. *Nucleic Acids Research*. 2023; doi: 10.1093/nar/gkac963.

36. Hastings J, de Matos P, Dekker A, Ennis M, Harsha B, Kale N, et al.. The ChEBI reference database and ontology for biologically relevant chemistry: enhancements for 2013. *Nucleic acids research*. 2013; doi: 10.1093/nar/gks1146.

37. Sostare E, Lawson TN, Saunders LR, Colbourne JK, Weber RJM, Sobanski T, et al.. Knowledge-Driven Approaches to Create the MTox700+ Metabolite Panel for Predicting Toxicity. *Toxicological Sciences*. 2022; doi: 10.1093/toxsci/kfac007.

38. Braekman JC, Daloze D, Pasteels J. Alkaloids in animals. *Alkaloids: Biochemistry, Ecology, and Medicinal Applications*. Springer; p. 349–78.

39. Böcker S, Rasche F. Towards de novo identification of metabolites by analyzing tandem mass spectra. *Bioinformatics*. 2008; doi: 10.1093/bioinformatics/btn270.

40. Peters K, Herman S, Khoonsari PE, Burman J, Neumann S, Kultima K. Metabolic drift in the aging nervous system is reflected in human cerebrospinal fluid. *Sci Rep*. 2021; doi: 10.1038/s41598-021-97491-1.

41. Colas L, Royer A-L, Massias J, Raux A, Chesneau M, Kerleau C, et al.. Urinary metabolomic profiling from spontaneous tolerant kidney transplanted recipients shows enrichment in tryptophan-derived metabolites. *eBioMedicine*. 2022; doi: 10.1016/j.ebiom.2022.103844.

42. Eysseric E, Gagnon C, Segura PA. Identifying congeners and transformation products of organic contaminants within complex chemical mixtures in impacted surface waters with a top-down non-targeted screening workflow. *Science of The Total Environment*. 2022; doi: 10.1016/j.scitotenv.2022.153540.

43. Rainer J, Vicini A, Salzer L, Stanstrup J, Badia JM, Neumann S, et al.. A Modular and Expandable Ecosystem for Metabolomics Data Annotation in R. *Metabolites*. 2022; doi: 10.3390/metabo12020173.

44. Smith CA, Want EJ, Maille GO, Abagyan R, Siuzdak G. XCMS : Processing Mass Spectrometry Data for Metabolite Profiling Using Nonlinear Peak Alignment , Matching , and Identification. *Anal Chem*. 2006; doi: 10.1021/ac051437y.

45. Kuhl C, Tautenhahn R, Böttcher C, Larson TRR, Neumann S, Bo C, et al.. CAMERA: An integrated strategy for compound spectra extraction and annotation of liquid chromatography/mass spectrometry data sets. *Anal Chem*. ACS Publications; 2012; doi: 10.1021/ac202450g.

46. Wickham H. ggplot2: Elegant graphics for data analysis. Springer-Verlag New York;

47. Conway JR, Lex A, Gehlenborg N. UpSetR: An R package for the visualization of intersecting sets and their properties. *Bioinformatics*. 2017; doi: 10.1093/bioinformatics/btx364.

48. Yu G. Using ggtree to visualize data on tree-like structures. *Current protocols in bioinformatics*. Wiley Online Library; 69:e962020;

49. Paradis E, Schliep K. ape 5.0: an environment for modern phylogenetics and evolutionary analyses in R. Schwartz R, editor. *Bioinformatics*. 2019; doi: 10.1093/bioinformatics/bty633.

50. Xu S, Wang Q, Wen S, Li J, He N, Li M, et al.. aplot: Simplifying the creation of complex graphs to visualize associations across diverse data types. *The Innovation*. 2025; doi: 10.1016/j.xinn.2025.100958.

51. Cao Y, Charisi A, Cheng L-C, Jiang T, Girke T. ChemmineR: a compound mining framework for R. *Bioinformatics*. 2008; doi: 10.1093/bioinformatics/btn307.

52. Kim S, Chen J, Cheng T, Gindulyte A, He J, He S, et al.. PubChem 2025 update. *Nucleic Acids Research*. 2025; doi: 10.1093/nar/gkae1059.

14 Author notes

Martin R. Jones and Thomas N. Lawson contributed equally to this article.

Publisher’s notes.

© The Author(s) 2025. Published by Oxford University Press GigaScience.

This is an Open Access article distributed under the terms of the Creative Commons Attribution License (https://creativecommons.org/licenses/by/4.0/), which permits unrestricted reuse, distribution, and reproduction in any medium, provided the original work is properly cited.

Click here to access/download;Figure;fig1.pdf

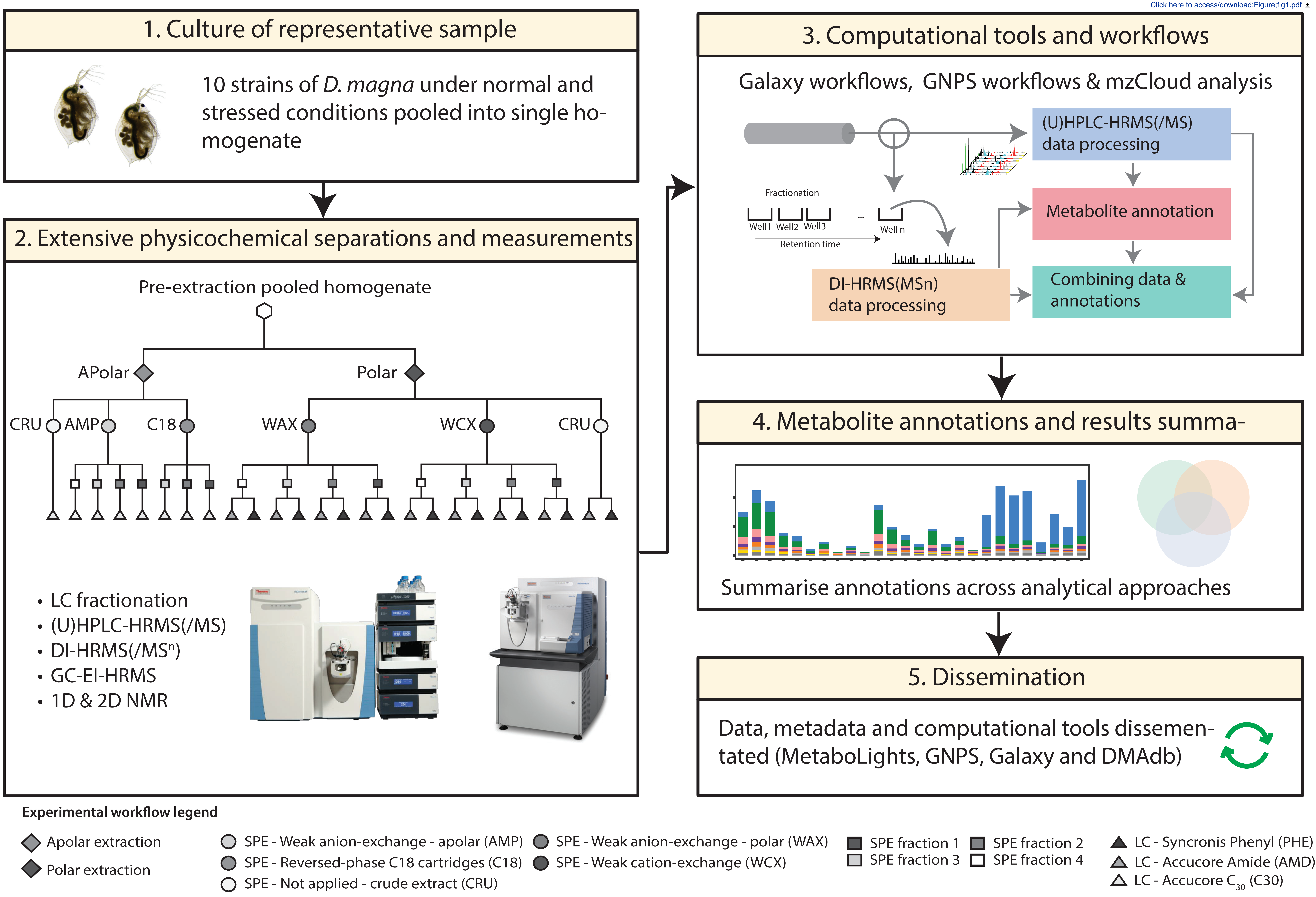

Click here to access/download;Figure;fig2.pdf

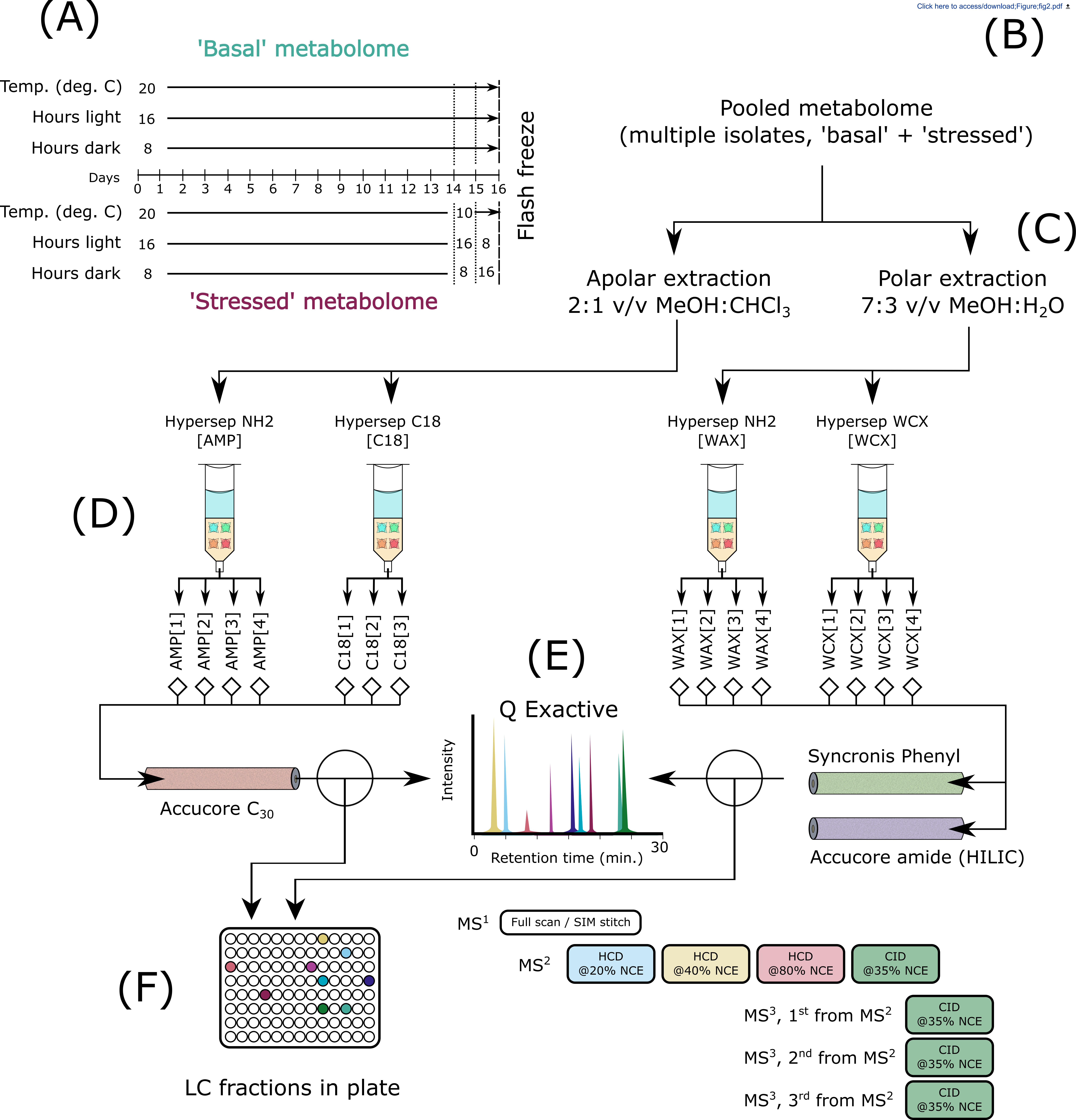

Click here to access/download;Figure;fig3.pdf

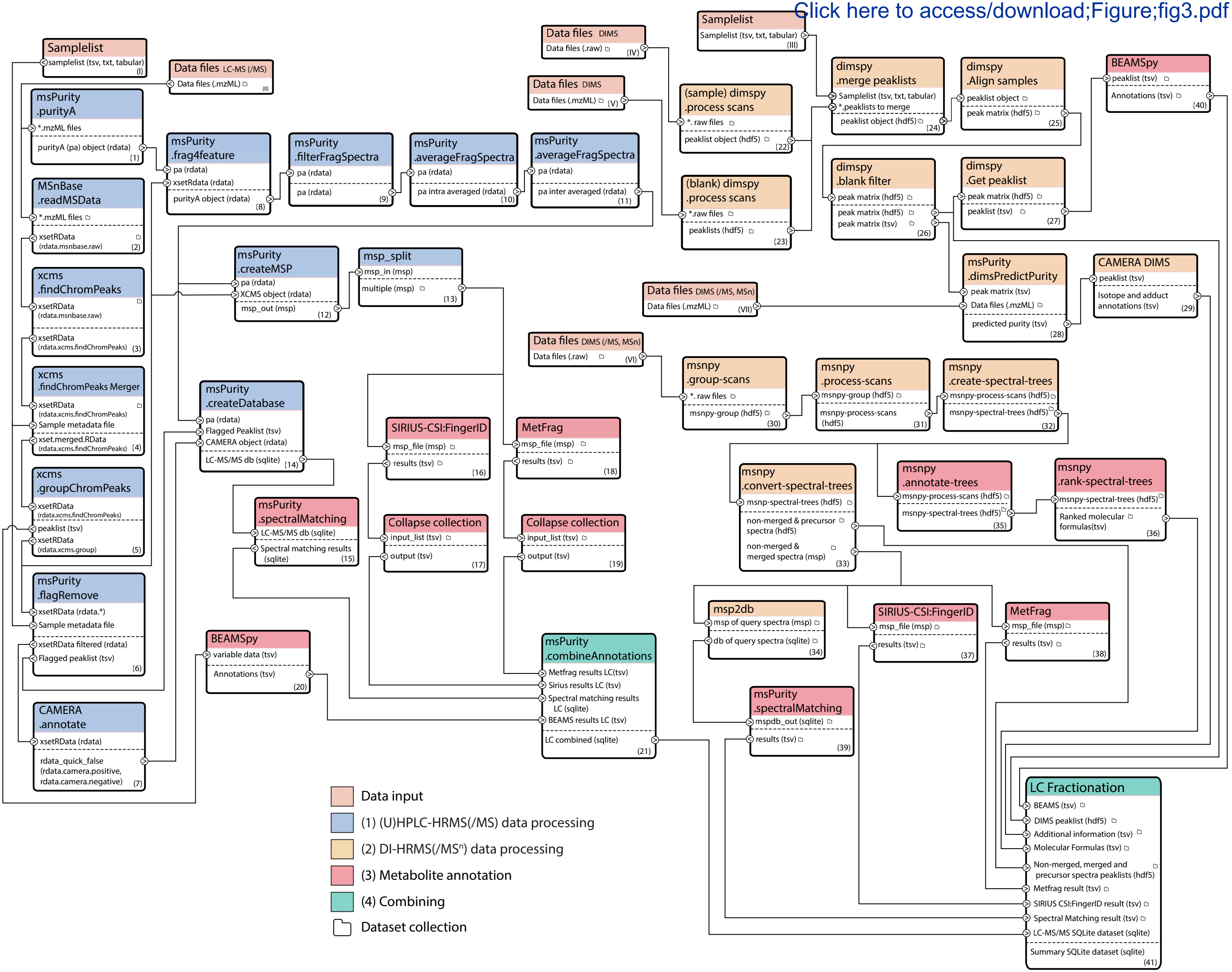

Click here to access/download;Figure;fig4.png

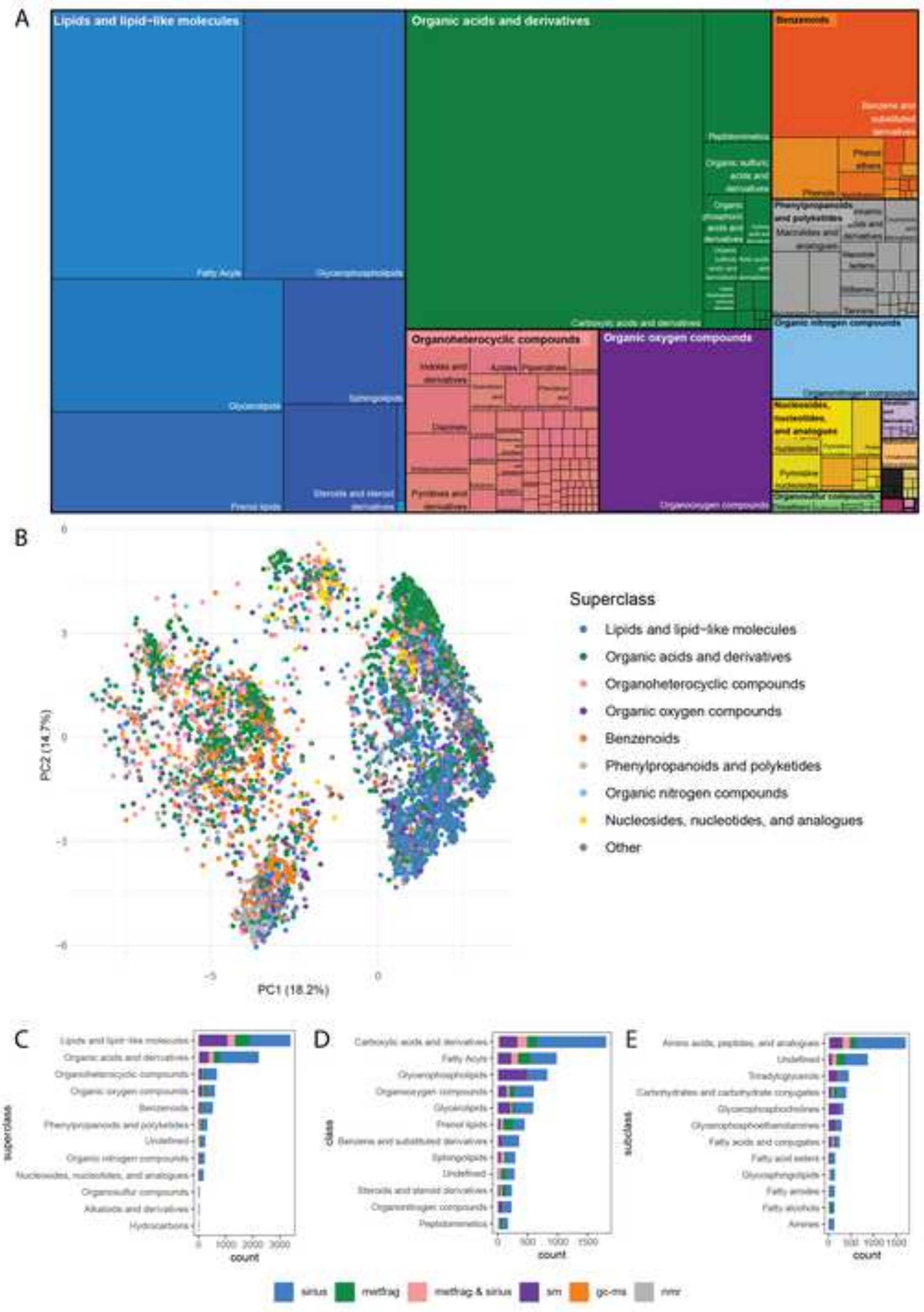

Click here to access/download;Figure;fig5.png

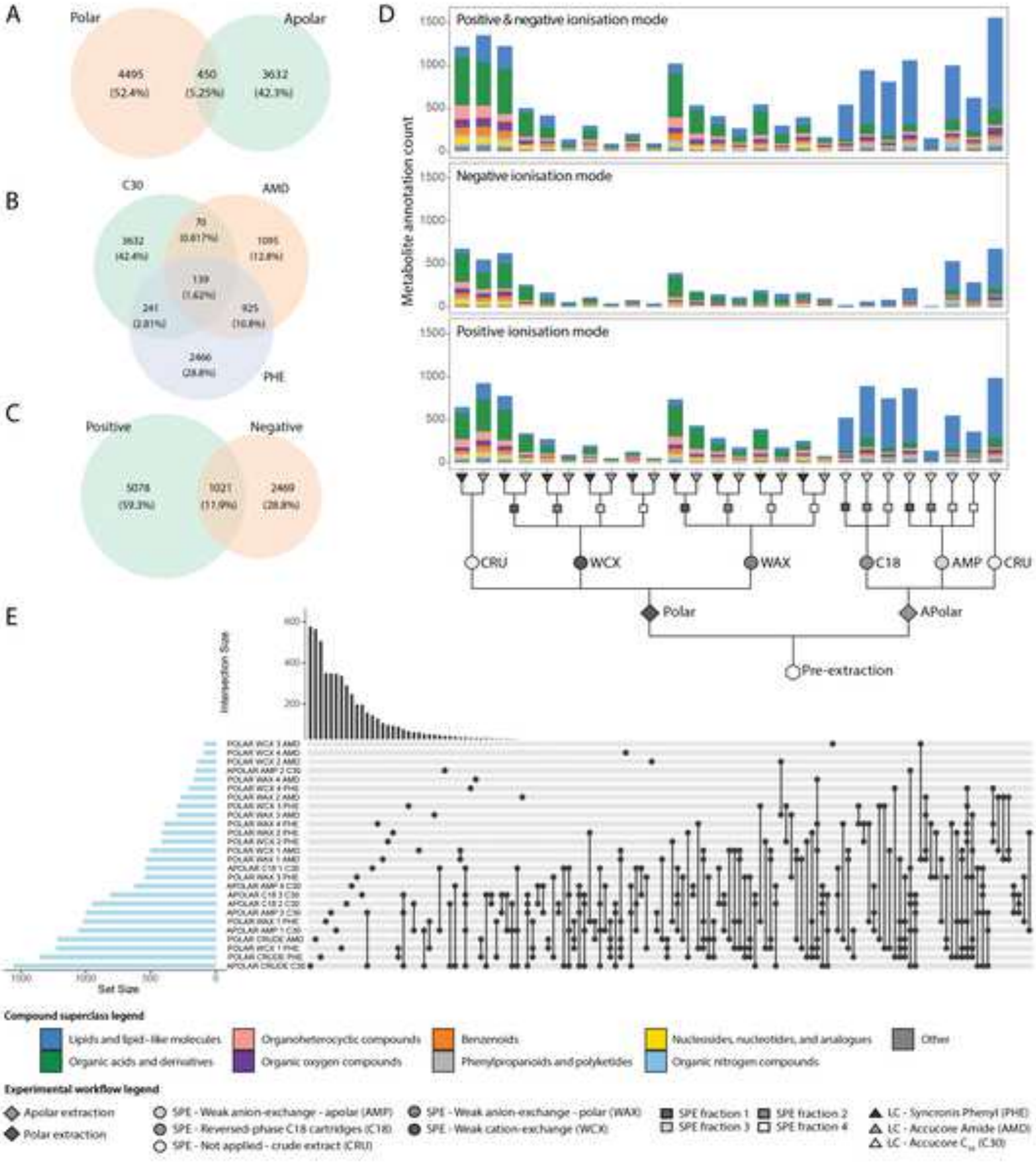

Click here to access/download;Figure;fig6.png

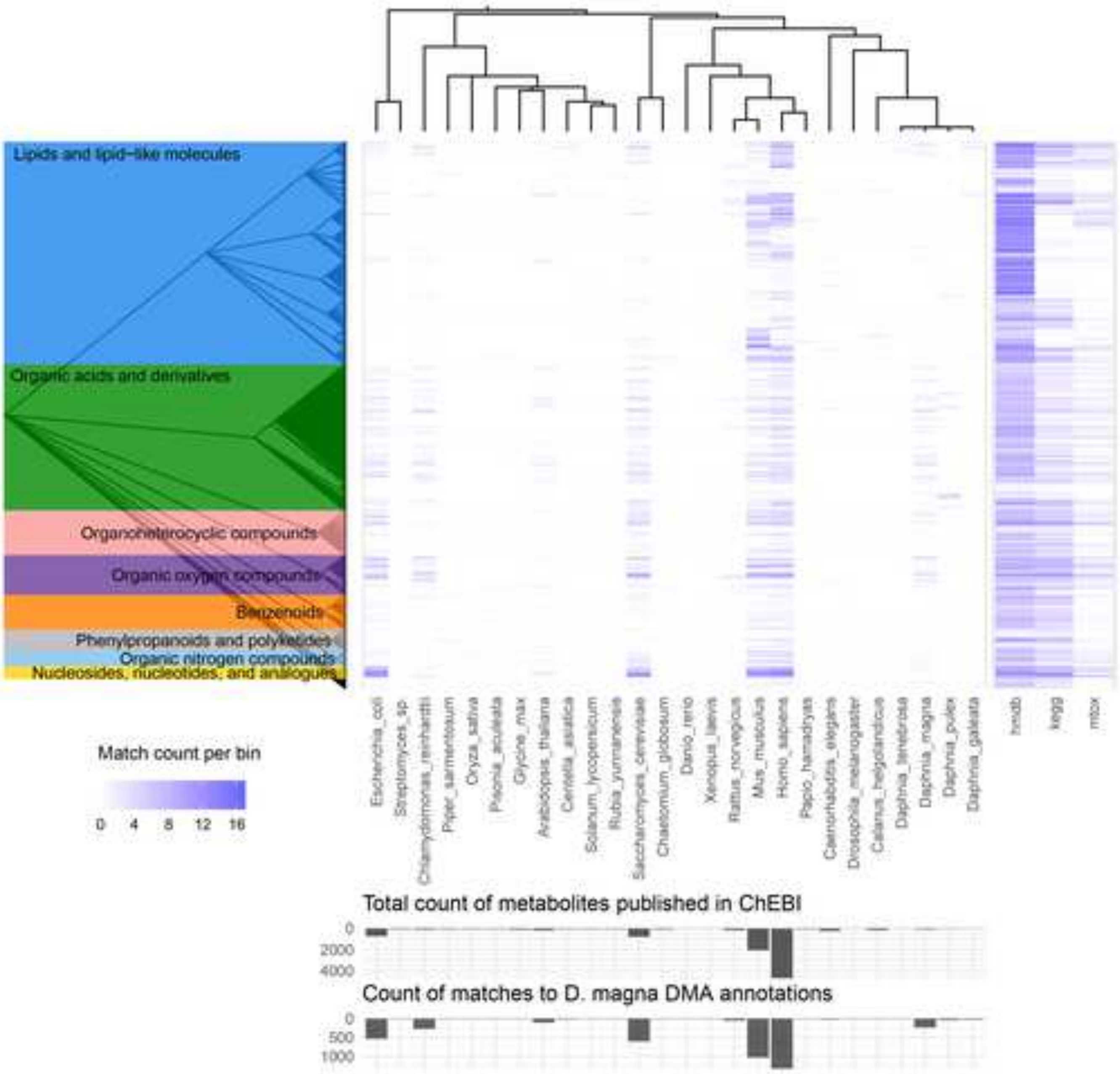

Click here to access/download;Figure;fig7.png

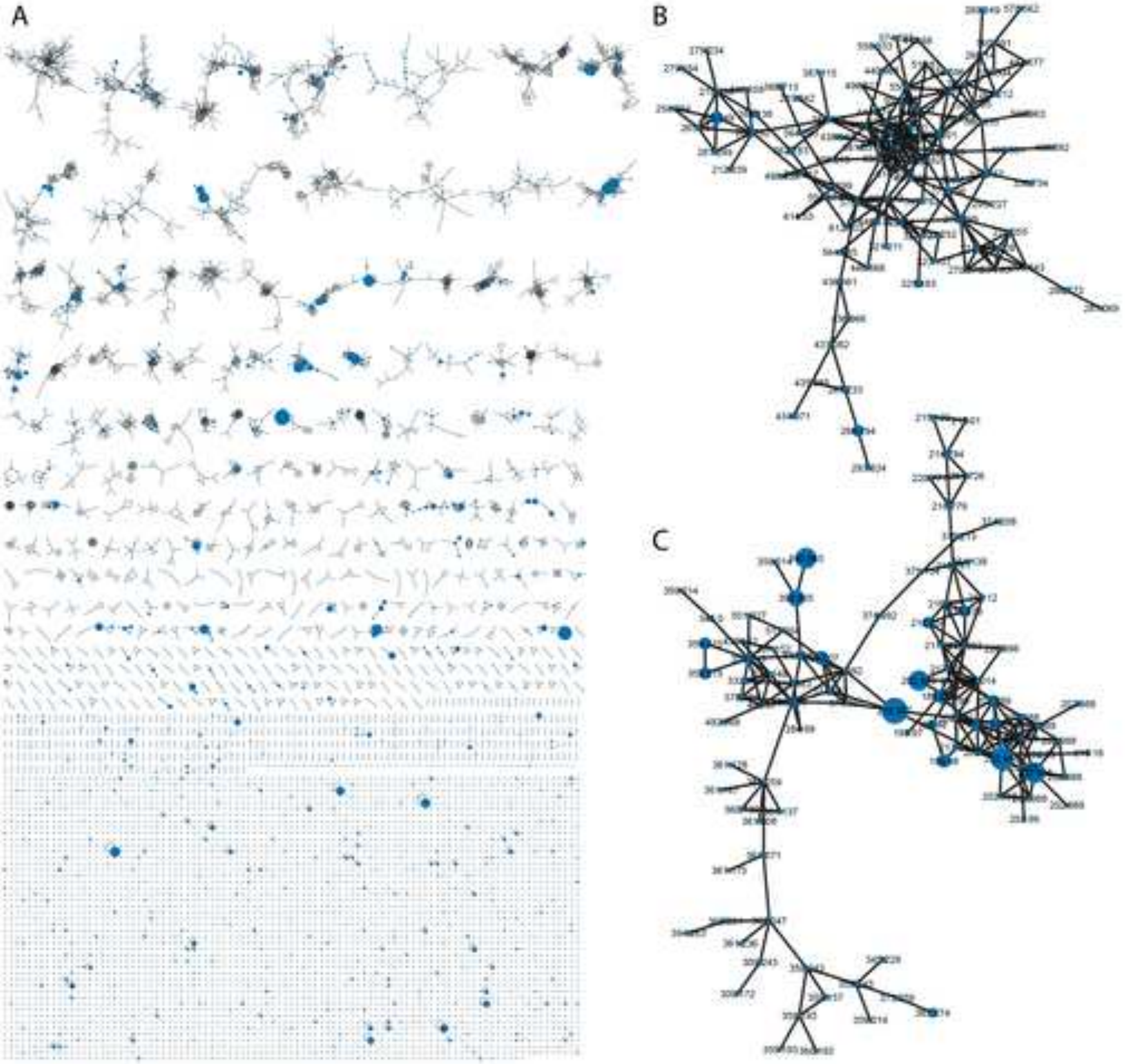

Click here to access/download Figure fig8.pdf

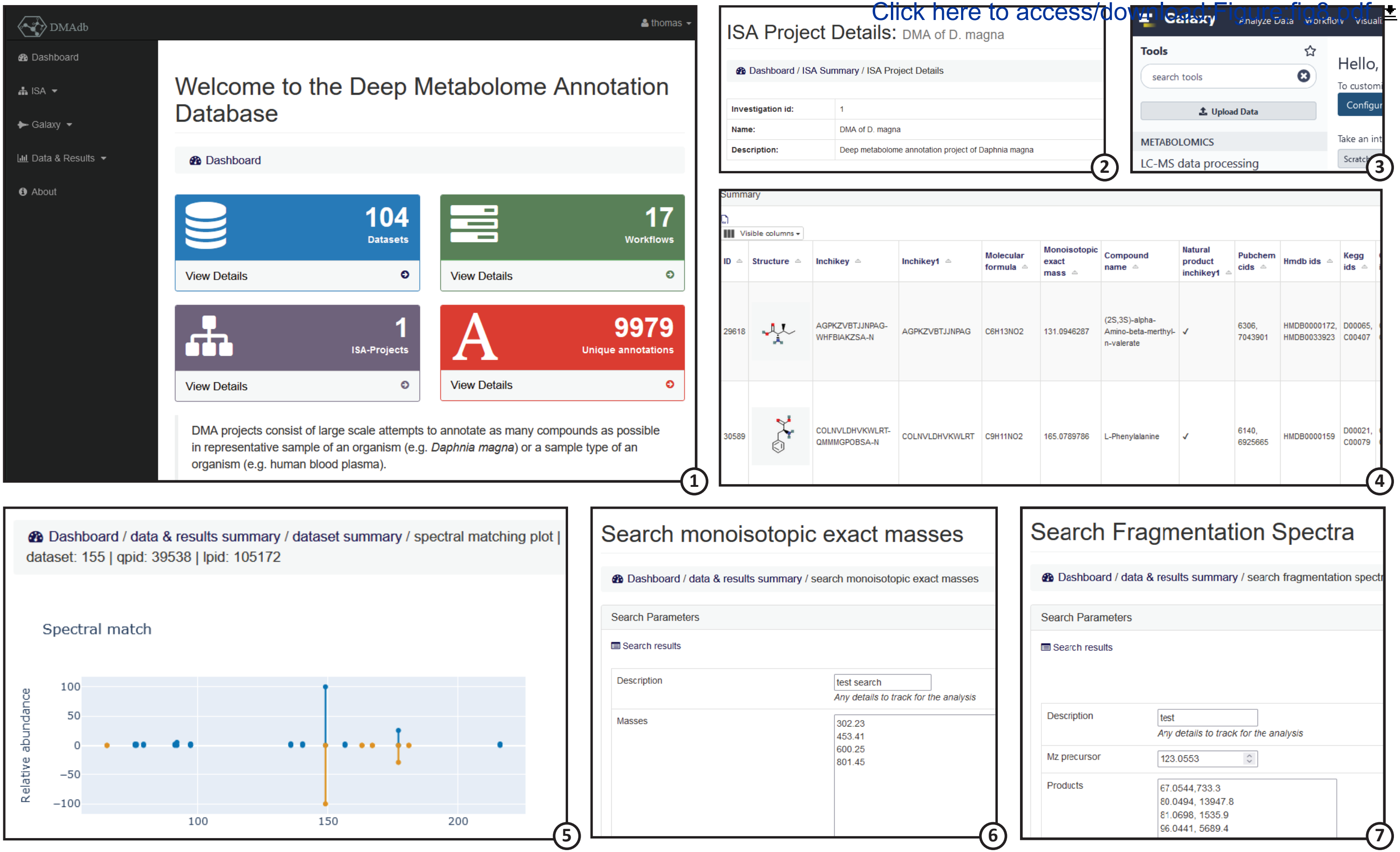

Click here to access/download
Supplementary Material
DMA D. magna - supp info - 28-10-2025.docx

Click here to access/download
Supplementary Material
DMA D. magna - supp tables - 28-10-2025.xlsx
