# Supplementary material for: Experimental and computational approaches for deep metabolome annotation with application to the ecotoxicological model organism Daphnia magna
Source: Gigascience. 2026 May 9;15:giag055. doi: 10.1093/gigascience/giag055 (PMC13254479; doi:10.1093/gigascience/giag055)
Supplement: giag055_GIGA-D-25-00453_revision_1 [file giag055_giga-d-25-00453_revision_1.pdf]

GigaScience

# Experimental and computational approaches for deep metabolome annotation with application to the ecotoxicological model organism Daphnia magna

--Manuscript Draft--

| | |
|---|---|
| Manuscript Number: | GIGA-D-25-00453R1 |
| Full Title: | Experimental and computational approaches for deep metabolome annotation with application to the ecotoxicological model organism Daphnia magna |
| Article Type: | Research |
| Funding Information: | Natural Environment Research Council (NE/L002493/1) — Dr Thomas N Lawson<br>Natural Environment Research Council (NE/J017442/1) — Dr Martin R Jones<br>Wellcome Trust (202952/Z/16/Z) — Dr Ralf J. M Weber<br>H2020 European Institute of Innovation and Technology (965406) — Professor Mark R Viant |
| Abstract: | Background: Comprehensively characterising the metabolomes of model organisms with high coverage and confidence is a critical step towards interpreting the metabolic basis of human and environmental health, yet there are formidable challenges involved in annotating metabolomes. A wide range of genotypes and phenotypes should be sampled with multiple complementary analytical approaches to cover the large and dynamic biochemical space they exhibit. In addition, multiple computational tools and approaches are required to annotate the metabolites from raw analytical data.<br>Results: To address this, we developed the Deep Metabolome Annotation (DMA) workflow. Applied to the ecological sentinel species, Daphnia magna, one pooled sample comprising ten distinct strains exposed to both normal and stressed environmental conditions was extracted and systematically physicochemically separated via solid-phase extraction, liquid- and gas-chromatography prior to extensive multiple-stage mass spectrometric fragmentation, generating more than 8,000 raw data files, and supplemented by nuclear magnetic resonance spectroscopy. An extensive Galaxy-based computational approach was built to analyse these data, comprising over 30 tools. The overall DMA efforts resulted in 8,181 annotated polar metabolites and lipids in D. magna, with the raw and processed data, tools and annotations disseminated freely via public data repositories and a custom web-based interface to maximise reusability.<br>Conclusions: The DMA workflow has generated one of the largest metabolome annotation datasets for any non-human model organism and provides the first in-depth characterisation of the D. magna metabolome – providing both a resource and a valuable catalyst for future deep metabolome annotation studies of other model organisms. |
| Corresponding Author: | Mark Viant<br>University of Birmingham<br>Birmingham, UNITED KINGDOM |
| Corresponding Author Secondary Information: | |
| Corresponding Author's Institution: | University of Birmingham |
| Corresponding Author's Secondary Institution: | |
| First Author: | Thomas N Lawson |
| First Author Secondary Information: | |
| Order of Authors: | Thomas N Lawson<br>Martin R Jones<br>Andrew J Chetwynd |

Powered by Editorial Manager® and ProduXion Manager® from Aries Systems Corporation

| | |
|---|---|
| | Elena Sostare |
| | Stefan Weidt |
| | Robert Mistrik |
| | Warwick B Dunn |
| | Ralf J. M Weber |
| | Mark R Viant |
| **Order of Authors Secondary Information:** | |
| **Response to Reviewers:** | Please refer to the file "DMA D. magna – comments addressed 28022026.docx" for detailed responses to the editor and reviewers.<br><br>We have also included updated versions of the manuscript and supplementary document with track changes enabled in Word. |
| **Additional Information:** | |
| **Question** | **Response** |
| Are you submitting this manuscript to a special series or article collection? | No |
| **Experimental design and statistics**<br><br>Full details of the experimental design and statistical methods used should be given in the Methods section, as detailed in our Minimum Standards Reporting Checklist. Information essential to interpreting the data presented should be made available in the figure legends.<br><br>Have you included all the information requested in your manuscript? | Yes |
| **Resources**<br><br>A description of all resources used, including antibodies, cell lines, animals and software tools, with enough information to allow them to be uniquely identified, should be included in the Methods section. Authors are strongly encouraged to cite Research Resource Identifiers (RRIDs) for antibodies, model organisms and tools, where possible.<br><br>Have you included the information requested as detailed in our Minimum Standards Reporting Checklist? | Yes |

Powered by Editorial Manager® and ProduXion Manager® from Aries Systems Corporation

| | |
|---|---|
| **Availability of data and materials**<br><br>All datasets and code on which the conclusions of the paper rely must be either included in your submission or deposited in publicly available repositories (where available and ethically appropriate), referencing such data using a unique identifier in the references and in the "Availability of Data and Materials" section of your manuscript.<br><br>Have you have met the above requirement as detailed in our Minimum Standards Reporting Checklist? | Yes |
| GigaScience has policies and guidelines in place for the use of generative AI-writing tools such as ChatGPT. If you have used such writing tools to assist with writing the manuscript this must be declared and cited in the text. Authors should not list AI-writing tools and other AI-assisted technologies as an author or co-author and should acknowledge that they are fully responsible for text generated or refined by AI-writing tools.<p><br><br>A summary of use (particularly in the introduction or among methods) needs to be included at the end of the paper, and the outputs should also be included as a supplementary file hosted in GigaDB or other open repositories. Please <a href=https://academic.oup.com/gigascience/pages/editorial_policies_and_reporting_standards target="_new" > read our guidelines for more information. </a> <p><br><br>By submitting to GigaScience, you are aware of the journal's AI-writing tools policy, and if you have declared use of such tools below, you have acknowledged this where appropriate in your manuscript and have made a summary of use and outputs available. </b><p><br><b>AI-assisted writing tools have been used in the preparation of this manuscript? | No |

Powered by Editorial Manager® and ProduXion Manager® from Aries Systems Corporation

Manuscript Click here to access/download;Manuscript;DMA D. magna - manuscript ed revised 01052026.docx

# Experimental and computational approaches for deep metabolome annotation with application to the ecotoxicological model organism *Daphnia magna*

Thomas N. Lawson[1,2,†], Martin R. Jones[1,†], Andrew J. Chetwynd[1,3,α], Elena Sostare[2], Stefan Weidt[5], Robert Mistrik[4,δ], Warwick B. Dunn[1,3,§], Ralf J. M. Weber[1,3,*], Mark R. Viant[1,2,3,*]

[1]School of Biosciences, University of Birmingham, Edgbaston, Birmingham, B15 2TT, UK
[2]Michabo Health Science Limited, Union House, 111 New Union Street, Coventry, CV1 2NT, UK
[3]Phenome Centre Birmingham, University of Birmingham, Edgbaston, Birmingham, B15 2TT, UK
[4]HighChem, Mlynské nivy 5, 821 09 Bratislava, Slovakia
[5]Glasgow Polyomics, University of Glasgow, University Avenue, Glasgow, G12 8QQ, UK

[†]Joint first authors
Present addresses: [α]Centre for Proteome Research, and [§]Centre for Metabolomics Research, Department of Biochemistry, Cell and Systems Biology, Institute of Systems, Molecular and Integrative Biology, University of Liverpool, Liverpool, L69 7ZB, UK; [δ]Bitmoderna, Leskova 11, 81104 Bratislava, Slovakia.

*Correspondence address: School of Biosciences, University of Birmingham, Edgbaston, Birmingham, B15 2TT, UK; E-mail: m.viant@bham.ac.uk; E-Mail: r.j.weber@bham.ac.uk

Andrew Chetwynd 0000-0001-6648-6881
Elena Sostare 0000-0002-9879-0818
Mark Viant 0000-0001-5898-4119
Martin Jones 0000-0003-4176-4819
Ralf Weber 0000-0002-8796-4771
Robert Mistrik 0009-0006-5472-6933
Stefan Weidt 0000-0003-1127-9214
Thomas Lawson 0000-0002-5915-7980
Warwick Dunn 0000-0001-6924-0027

## Abstract

**Background:** Comprehensively characterising the metabolomes of model organisms with high coverage and confidence is a critical step towards interpreting the metabolic basis of human and environmental health, yet there are formidable challenges involved in annotating metabolomes. A wide range of genotypes and phenotypes should be sampled with multiple complementary analytical approaches to cover the large and dynamic biochemical space they exhibit. In addition, multiple computational tools and approaches are required to annotate the metabolites from raw analytical data.

**Results:** To address this, we developed the Deep Metabolome Annotation (DMA) workflow. Applied to the ecological sentinel species, *Daphnia magna,* a pooled sample comprising ten distinct strains exposed to both normal and stressed environmental conditions was extracted and systematically physicochemically separated via solid-phase extraction, liquid- and gas-chromatography prior to extensive multiple-stage mass spectrometric fragmentation, generating more than 8,000 raw data files, and supplemented by nuclear magnetic resonance spectroscopy. An extensive Galaxy-based computational approach was built to analyse these data, comprising over 30 tools. The overall DMA efforts resulted in 8,181 annotated polar metabolites and lipids in *D. magna,* with the raw and processed data, tools and annotations disseminated freely via public data repositories and a custom web-based interface to maximise reusability.

**Conclusions:** The DMA workflow has generated one of the largest metabolome annotation datasets for any non-human model organism and provides the first in-depth characterisation of the *D. magna* metabolome, serving as both a resource and a valuable catalyst for future deep metabolome annotation studies of other model organisms.

# 1 Introduction

Large-scale efforts to map and catalogue both human and model organism genomes have been a fundamental driver of change in biological and biochemical research over the past few decades. The technological developments and resulting biological, biomedical and environmental knowledge derived from such projects have helped underpin the modern era of biological sciences [1], [2], [3], [4]. In contrast, our understanding of metabolic biochemistry (where we use the term metabolites here to represent the full spectrum of low molecular weight endogenous biochemicals from polar metabolites to lipids) has increased relatively minimally over the last half a century. Such knowledge must either be inferred from genome-scale metabolic reconstructions or, if measured experimentally, is limited to metabolites that can be annotated analytically (i.e. using metabolomics datasets). Ongoing improvements in both analytical and computational methods for metabolic annotation now allow for more extensive metabolite annotation coverage than what could be performed 10 years ago. However, although these developments are welcomed and are in part reflected by the increase in both studies featuring extensive metabolite annotation analysis [5], [6], [7] and the maturation of databases containing metabolites and relevant experimental data (Metabolights, Metabolomics Workbench, HMDB, GNPS, MoNA, MassBank, LipidBlast and mzCloud [8], [9], [10], [11], [12], [13]), for the majority of widely used model organisms the metabolome knowledge is still severely lacking.

Whilst the need for deeper metabolome knowledge of model organisms has been well established [14], the challenges are still considerable and multi-faceted. First, the biology: as the metabolome is driven by genes, the changing environment, and their interactions, achieving a comprehensive map of the breadth of a species' metabolome requires a range of genotypes and phenotypes. Second, analytical chemistry: no single method is sufficient to cover the chemical space of a metabolome, hence multiple physicochemical separations and detection techniques are required. Third, the computational challenges: as metabolomes are vast, specialised tools and workflows for data processing and metabolite annotation are required, together with resources for data and metadata management. Where possible, the data generated and software used should be Findable, Accessible, Interoperable, Reusable (FAIR) and scalable in order to support an anticipated further cascade of deep metabolome annotation studies.

To address these challenges, we have developed and applied an experimental and computational workflow for extensively measuring the metabolome of model organisms, applied here to *Daphnia magna*. The International Metabolomics Society's Model Organism Metabolomes task group [15] and the ongoing Precision Toxicology project [16] both highlight the crustacean *Daphnia* as a key model organism due to its importance as an indicator genus used to set ecotoxicological regulatory standards (SOR/2002-222), and from being extensively studied in the context of evolution and ecology [17], [18], [19], [20], [21], making it an excellent candidate for an in-depth investigation of its metabolome. Experimentally, the workflow involved culturing multiple strains of *D. magna* under normal and stressed conditions to provide a representative pooled sample for metabolome annotation. This sample underwent extensive extraction and physicochemical separation and (ultra)-high-performance liquid chromatography-high resolution tandem mass spectrometry ((U)HPLC-HRMS(/MS)) analysis. Concurrent fractionation yielded (semi-) purified metabolome fractions that underwent in-depth characterisation by direct infusion-

high resolution multiple stage mass spectrometry (DI-HRMS(/MS$^n$)). Supplemental analyses by gas chromatography-electron ionisation-high resolution mass spectrometry (GC-EI-HRMS) and 1- and 2-dimensional nuclear magnetic resonance spectroscopy (1D- & 2D-NMR), helped to ensure broad coverage of the physicochemical space of metabolites. A computational workflow was then developed and applied for processing, annotating and managing the data and results, heavily utilising the Galaxy Workflow platform [22]. The resulting metabolome annotations, data, metadata and computational tools are disseminated through various channels (MetaboLights, GNPS, Galaxy and a custom web portal, named DMAdb) to ensure traceability and reusability.

The combined extensive experimental and computational workflow, referred to here as the Deep Metabolome Annotation (DMA) workflow (see **Figure 1**), has generated one of the largest metabolome annotation datasets for any single organism and provides the first in-depth characterisation of the *D. magna* metabolome – a resource that is much needed to improve the interpretation of *Daphnia* biology and toxicology. This workflow can also be redeployed for deep metabolome annotation of other model organisms.

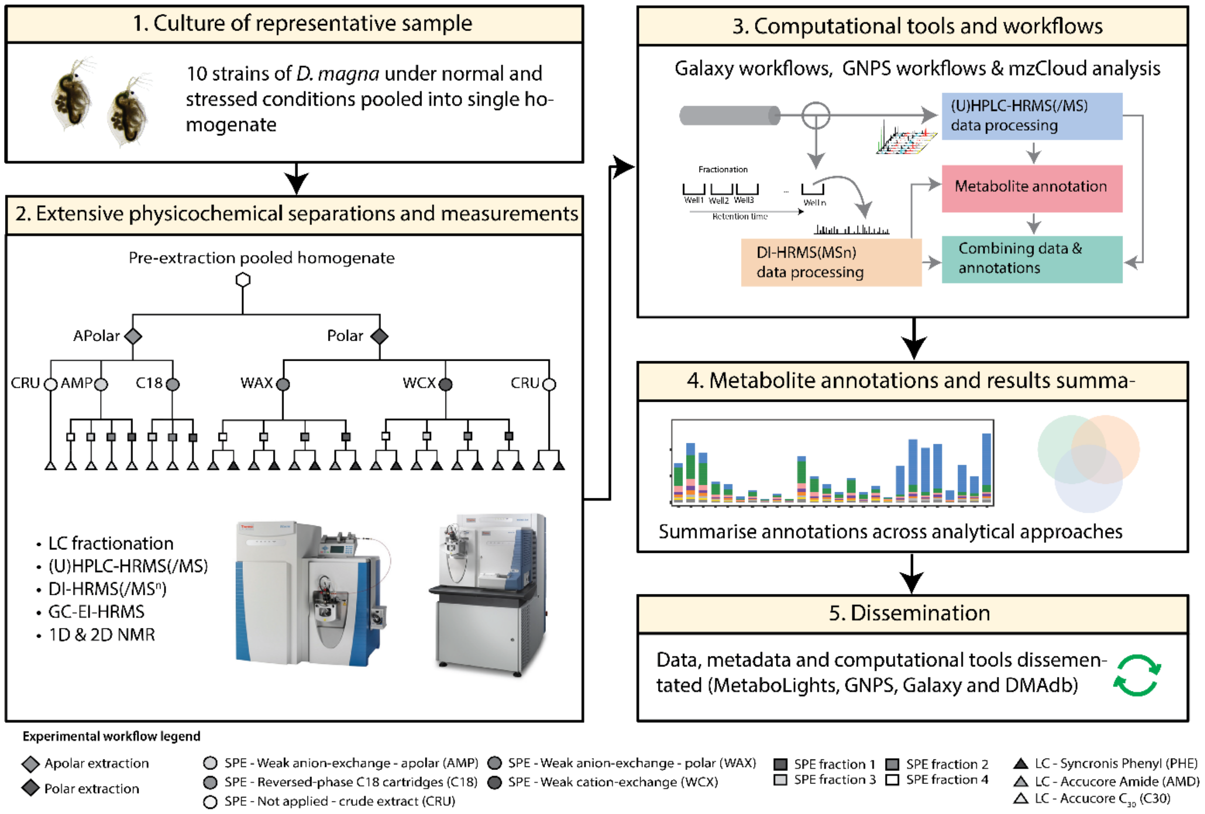


***Figure 1: Deep Metabolome Annotation (DMA) workflow - conceptual overview and application to D. magna***
***1)*** *First a representative sample (applied to D. magna here) cultured under multiple conditions using distinct strains are homogenised into a single sample.* ***2)*** *The pooled homogenised sample then undergoes extensive physicochemical separations (including polar and apolar extraction procedures; four types of solid phase extraction (SPE) – each collecting 3-4 SPE fractions; three types of liquid chromatography (LC); and extensive LC fractionation). SPE fractions then undergo extensive analysis based on (ultra)-high-performance liquid chromatography-high resolution tandem mass spectrometry ((U)HPLC-HRMS(/MS)), followed by direct infusion-high resolution mass spectrometry with multiple-stage fragmentation (DI-HRMS(/MS$^n$)). SPE fraction analyses are also supplemented with gas chromatography-electron ionisation-high resolution mass spectrometry (GC-EI-*

*HRMS) and 1- and 2-dimensional nuclear magnetic resonance spectroscopy (1D- & 2D-NMR).* ***3)*** *Extensive computational tools and workflows were developed and applied to process and annotate the metabolite. The results are then summarised across the analytical workflow* ***(4)*** *and finally the data, metadata and computational tools are disseminated to ensure traceability and reusability* ***(5)****. Images of Thermo Scientific Q Exactive and Orbitrap Elite mass spectrometers by Thermo Fisher Scientific (Bremen), licensed under Creative Commons Attribution–ShareAlike 3.0 Unported (CC BY-SA 3.0), via Wikimedia Commons.*
*ALT TEXT: Graphical representation of the four stages of the deep metabolome annotation workflow.*

# 2 Methods

## 2.1 Deep metabolome annotation experimental workflow

### *2.1.1 Overview*

An experimental workflow for extensive physicochemical separation and analytical measurement of metabolites has been developed for the analysis of model organisms – applied here to *D. magna*. An overview is provided in **Figure 2**.

This extensive workflow, applied to both the *Daphnia* sample and a metabolite reference standard sample, was separated into 135 distinct experimental assays (see **Supplemental Table S1**). Of these assays, 103 corresponded to (U)HPLC-HRMS(/MS) and DI-HRMS(/MS$^n$) analysis, generating 8,846 raw mass spectrometry files (5,430 files specifically corresponding to the *Daphnia* sample with the remaining files corresponding for metabolite reference standards, extract blanks, or quality assurance measurements such as (U)HPLC-HRMS system equilibration). See **Supplemental Table S2** for the full file list.

### *2.1.2 D. magna culturing and sample preparation*

The DMA experimental workflow is applied here to *D. magna*; however, the same considerations extend to DMA analyses of other model organisms. The workflow should take as input a set of samples that, ideally, span diverse genetic and environmental backgrounds and collectively reflect the full metabolic repertoire accessible to the organism under study. These samples are then pooled and homogenised to form a single complex sample matrix that constitutes an average of the constituent metabolomes.

For the DMA of *D. magna*, ca. 2,000 individual organisms were pooled and homogenised, generating a homogenate consisting of ten distinct strains (**Supplemental Table S3)** exposed to two contrasting environmental conditions. A 'basal' metabolome was represented by *D. magna* cultured under standard conditions (20 +/- 2 °C with a 16:8 hr light:dark ratio) for 14 days, followed by a further 48 hr under the same conditions. A 'stressed' metabolome was represented by *D. magna* cultured for 14 days under standard conditions, followed by 24 hr at 10 +/- 1 °C with 16:8 hr light:dark ratio, and then a further 24 hr at 10 +/- 1 °C with 8:16 hr light:dark ratio. Under both conditions, *Daphnia* were maintained without food (algae) throughout the final 48 hr of culturing, to minimise the presence of algae in the gut and prioritise measurement of metabolites derived from *Daphnia* rather than the food source. Further details are provided in **Supplemental Section 1.5** and **Supplemental Tables S4-5**.

### *2.1.3 Metabolite extraction from D. magna pooled sample*

Metabolites were extracted from the pooled homogenate using two distinct liquid-phase extraction protocols: a 'polar' extraction in which (predominantly polar through to moderately-polar) metabolites were extracted using a solution comprising 71.4:28.6% v/v methanol:water, and; an 'apolar' extraction protocol in which metabolites (spanning moderately-apolar through to highly apolar metabolites, including lipids) were extracted using a solution of 1:1 v/v methanol:chloroform, to which water was added to form a biphasic system comprising 2:2:1.8 v/v/v chloroform:methanol:water, from which the lower (apolar) layer was recovered. Polar extracts were dried in a centrifugal vacuum concentrator

(Speedvac), while apolar extracts were dried under a stream of nitrogen gas. Further details are provided in **Supplemental Section 1.6**.

#### *2.1.4 Solid phase extraction-based fractionation of metabolite extracts*

Constituents of the polar or apolar extract were independently fractionated over two solid-phase extraction (SPE) cartridges. The polar extract was fractionated using weak anion-exchange (WAX; aminopropyl) and weak cation-exchange cartridges (WCX; carboxylic acid), while the apolar extract was fractionated using weak anion-exchange (referred to as AMP to differentiate from the polar arm; aminopropyl) and reversed-phase C18 cartridges (C18). See **Supplemental Section 1.7** and **Supplemental Figures S1-2** for further details. The resulting 15 SPE fractions and remaining unfractionated extracts (referred to as "crude" extract) were analysed by (U)HPLC-HRMS(/MS). A selected subset of SPE fractions, alongside crude extract, were also analysed by 1D- & 2D-NMR spectroscopy and GC-EI-HRMS, to further expand the breadth of metabolome annotation.

#### *2.1.5 (U)HPLC-HRMS(/MS), DI-HRMS(/MS$^n$) and LC fractionation*

Three distinct (U)HPLC-HRMS(/MS) methods were applied in both positive and negative ionisation modes to analyse constituents of the polar and apolar crude extracts, and associated SPE fractions. Polar crude extract and polar SPE fractions were analysed by hydrophilic interaction liquid chromatography (HILIC) using an Accucore amide column (AMD; 2.1 x 100 mm, 2.6 µm solid core; Thermo Scientific), and reversed-phase liquid chromatography (RPLC) based on a Syncronis phenyl column (PHE; 2.1 x 100 mm, 1.7 µm; Thermo Scientific). Constituents of the apolar crude extract and apolar SPE fractions, meanwhile, were analysed by RPLC using an Accucore C30 column (C30) (2.1 x 100 mm, 2.6 µm solid-core particle, 150 Å; Thermo Scientific). All chromatographic separations were performed using a Dionex Ultimate 3000 liquid chromatography system. A Q Exactive mass spectrometer (Thermo Scientific), fitted with heated electrospray ionisation source, was used for HRMS(/MS) mass spectrometry analysis of metabolites eluted from LC columns. A passive flow splitting tee-piece was installed between the LC column outlet and Q Exactive inlet to facilitate simultaneous collection of 20-second-wide LC fractions and associated HRMS(/MS) data. Each fraction was collected into independent wells of a deep well plate. LC fraction collection plates were dried in a centrifugal evaporator at the end of an analysis sequence.

Initial (U)HPLC-HRMS analyses were used to create inclusion and exclusion lists (i.e. *m/z* features of interest) to direct the subsequent data dependent acquisition (DDA) of (U)HPLC-HRMS/MS data. In parallel, eluent from the LC columns were fraction-collected during mass spectral acquisition, which were then subject to extensive DI-HRMS(/MS$^n$).

DI-HRMS(/MS$^n$) analyses of resuspended LC fractions were performed using an Orbitrap Elite mass spectrometer (Thermo Scientific) using both higher energy collisional dissociation (HCD) and collision-induced dissociation (CID) at several levels of normalised collision energy (NCE). Specifically, HCD was performed at 20, 40 and 80% NCE, followed by CID at 35% NCE with multi-stage fragmentation up to MS$^3$. In total, 2,305 LC fractions were analysed as part of the DMA of *D. magna*. The acquisition of DI-HRMS(/MS$^n$) fragmentation data was directed via a predefined list of targeted *m/z* features, derived from prior DI-HRMS analysis of the same fraction.

For detailed information on the (U)HPLC-HRMS analytical setup and LC methods, including the fractionation procedure, as well as the data acquisition sequence and computational methods used to create inclusion/exclusion lists for targeting the most informative features for fragmentation data acquisition, see **Supplemental Sections 1.8.1 and Supplemental Figure S3**. Additionally, for more details on the resuspension of LC fractions, the DI-HRMS(/$MS^n$) analytical setup, data acquisition sequence, and the computational methods for DI-HRMS(/$MS^n$) used to develop both inclusion/exclusion lists and instrument methods files that directed DI-$HRMS^n$ data acquisition, see **Supplemental Sections 1.8.2** and **Supplemental Figure S4**.

### *2.1.6 (U)HPLC-HRMS(/MS) method optimisation*

The PHE and AMD (U)HPLC-HRMS(/MS) methods underwent optimisation for DMA of *D. magna* aiming to maximize reproducibly detectable metabolic features while enabling reproducible fractionation for downstream analyses. The methodology for the optimisation is detailed within the **Supplemental Section 1.9, Figure S5** and **Supplemental Tables S6-S8.** The C30 (U)HPLC-HRMS(/MS) method was previously optimised for broad lipid profiling applications by Thermo Fisher Scientific, hence no further optimisation was pursued.

### *2.1.7 GC-EI-HRMS*

GC-EI-HRMS was performed on the WAX and WCX fractions, as well as the crude polar extract, using a TriPlus RSH autosampler and TRACE 1310 gas chromatograph coupled to a Q Exactive mass spectrometer (Thermo Scientific), and an Extractabrite electron ionisation/chemical ionisation source. Further details are provided in **Supplemental Section 1.10**.

### *2.1.8 1D & 2D NMR spectroscopy*

The WAX and WCX SPE fractions were also analysed using a combination of 1D and 2D-NMR spectroscopy experiments performed using a Bruker AVANCE III 600 MHz NMR spectrometer, equipped with a 1.7 mm TCI-Cryoprobe and operated at a proton frequency of 600.13 MHz. Each sample was measured using 1D proton nuclear overhauser effect NMR spectroscopy (1D-$^1$H-NOESY) followed by 2D homonuclear $^1$H-$^1$H (2D-JRes and TOCSY) and heteronuclear $^1$H-$^{13}$C (HSQC) experiments to support annotation. Further details are provided in **Supplemental Section 1.11**.

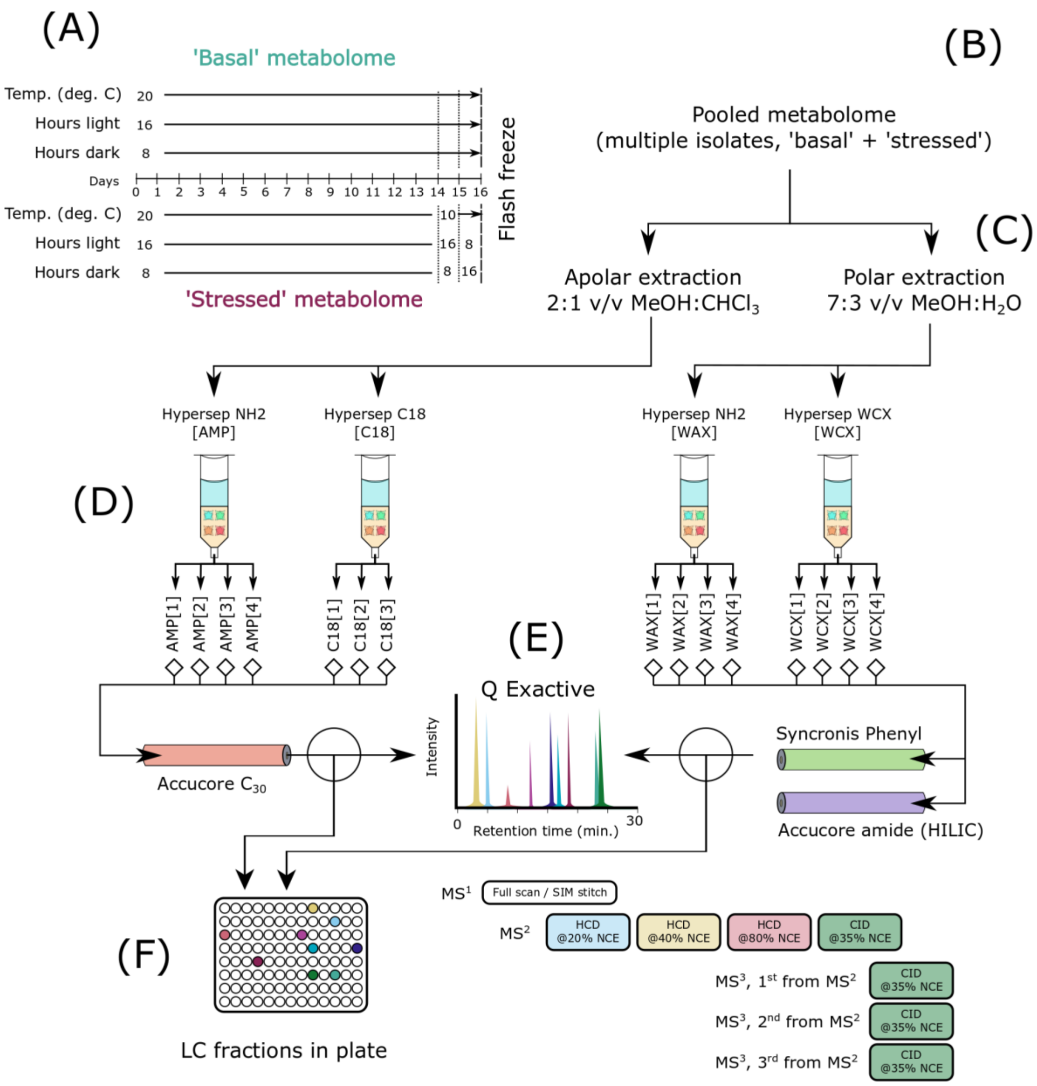


**Figure 2: Deep metabolome annotation experimental workflow for the physicochemical separation and measurement of *D. magna* metabolites. *A)*** *Culturing: Culturing of ten strains of a model organism under normal (basal) and stressed conditions was used to ensure a wide range of metabolites were present in* ***B)*** *the pooled sample. For the DMA of D. magna this involved flash freezing in liquid nitrogen to quench metabolism, followed by homogenisation and pooling into a single sample.* ***C)*** *Liquid phase extraction: Two distinct liquid phase extractions were then performed on the pooled homogenate from step B), for 'polar' and 'apolar' metabolites.* ***D)*** *Solid phase extraction: Four types of solid phase extraction (SPE) were performed on the extracts from step C), the polar extracts using weak anion-exchange (WAX) and weak-cation exchange SPE cartridges (WCX) and apolar extracts using weak anion-exchange (AMP) and reversed-phase C18 cartridges (C18).* ***E)*** *(U)HPLC-HRMS(/MS): Three distinct (U)HPLC-HRMS(/MS) methods were applied using a Q-Exactive mass spectrometer: Accucore amide HILIC LC column (AMD) and Syncronis phenyl LC column (PHE) for analysis of metabolites from the polar arm of the workflow, and Accucore C30 LC column (C30) for the apolar extract and any SPE fractions derived from the apolar extract.* ***F)*** *Fractionation and DI-HRMS(/MS$^n$): The eluate from the LC columns in step E) was fractionated into plates for subsequent extensive multiple-stage fragmentation (MS$^n$) analysis applied (up to MS$^3$), including at multiple collision energies and with technical replication.*
*ALT TEXT: Graphical representation of the Deep Metabolome Annotation experimental workflow.*

## 2.2 Computational tools and workflows for data processing, metabolite annotation and data analysis

### *2.2.1 Overview*

An extensive computational workflow utilising the Galaxy platform has been developed to analyse the highly complex data acquired through the experimental DMA workflow. This computational workflow predominantly consists of an extensive Galaxy-based workflow, with additional annotations incorporated from external sources i.e., mzCloud, GNPS workflows, GC-EI-HRMS annotations and 1D & 2D NMR annotations. A summary is provided in **Supplemental Section S12** and **Supplemental Figure S6**.

The Galaxy workflow component generated 104 Galaxy histories (see **Supplemental Table S1** for links to corresponding Galaxy history), 60 of which were used for the analysis of the *Daphnia* samples. Each Galaxy history contains a combined SQLite database containing all annotations and relevant (average) spectra across all assays.

### *2.2.2 Galaxy workflow details*

The Galaxy workflow (see **Figure 3**) was designed specifically to process and perform metabolite annotation across the multiple data types produced by the DMA experimental workflow, including (U)HPLC-HRMS(/MS) and DI-HRMS(/MS$^n$). Utilising the high level of replication achieved from the workflow, averaging and filtering were performed on both the (U)HPLC-HRMS(/MS) and DI-HRMS(/MS$^n$) data so that higher-quality, reproducible fragment peaks were retained for multiple complementary computational approaches to metabolite annotation.

The workflow is split into five components: “Data input”, “(U)HPLC-HRMS(/MS) data processing”, “DI-HRMS(/MS$^n$) data processing”, “Metabolite annotation” and “Combining”. A detailed description of all steps used in the workflow can be found in **Supplemental Section 1.13 –** with individual schematics detailing the (U)HPLC-HRMS(/MS) (See **Supplemental Figure S7**) and DI-HRMS(/MS$^n$) fragmentation data processing (See **Supplemental Figure S8**). See also **Supplemental Table S9** for the description of all tools used and **Table 1** for the location of code repositories for each Galaxy tool and underlying software.

The Galaxy workflow incorporates both new and existing tools, e.g. existing Workflow4Metabolomics XCMS Galaxy tools [23] for (U)HPLC-HRMS peak picking and processing; DIMSpy Galaxy tools [24], [25] for DI-HRMS data processing. New Galaxy tools developed for the DMA project include updated functionality from the msPurity R package [26] to filter and flag spectra, average fragmentation spectra, create MSP and SQLite files of (U)HPLC-HRMS(/MS) data, perform spectral matching, and combine metabolite annotations from multiple sources; the MSnPy python package and Galaxy tools to process DI-HRMS(/MS$^n$) data with both multiple-stage and multiple energy fragmentation spectral trees, perform spectral averaging across trees, and annotate and rank spectral trees with molecular formulae; and the LC Fractionation Galaxy tool which was created to combine all spectra and metabolite annotations from a DMA LC fractionation experiment. In addition, Galaxy wrappers have been created for the *in silico* fragmentation software MetFrag [27], [28], [29] and mass spectrometry data processing and metabolite annotation software SIRIUS CSI:FingerID [30].

This modular, multistage workflow ensures reusability across other studies. Additionally, a Galaxy instance [31] was created for conducting the Galaxy DMA analysis, with public access to the histories, data and parameters.

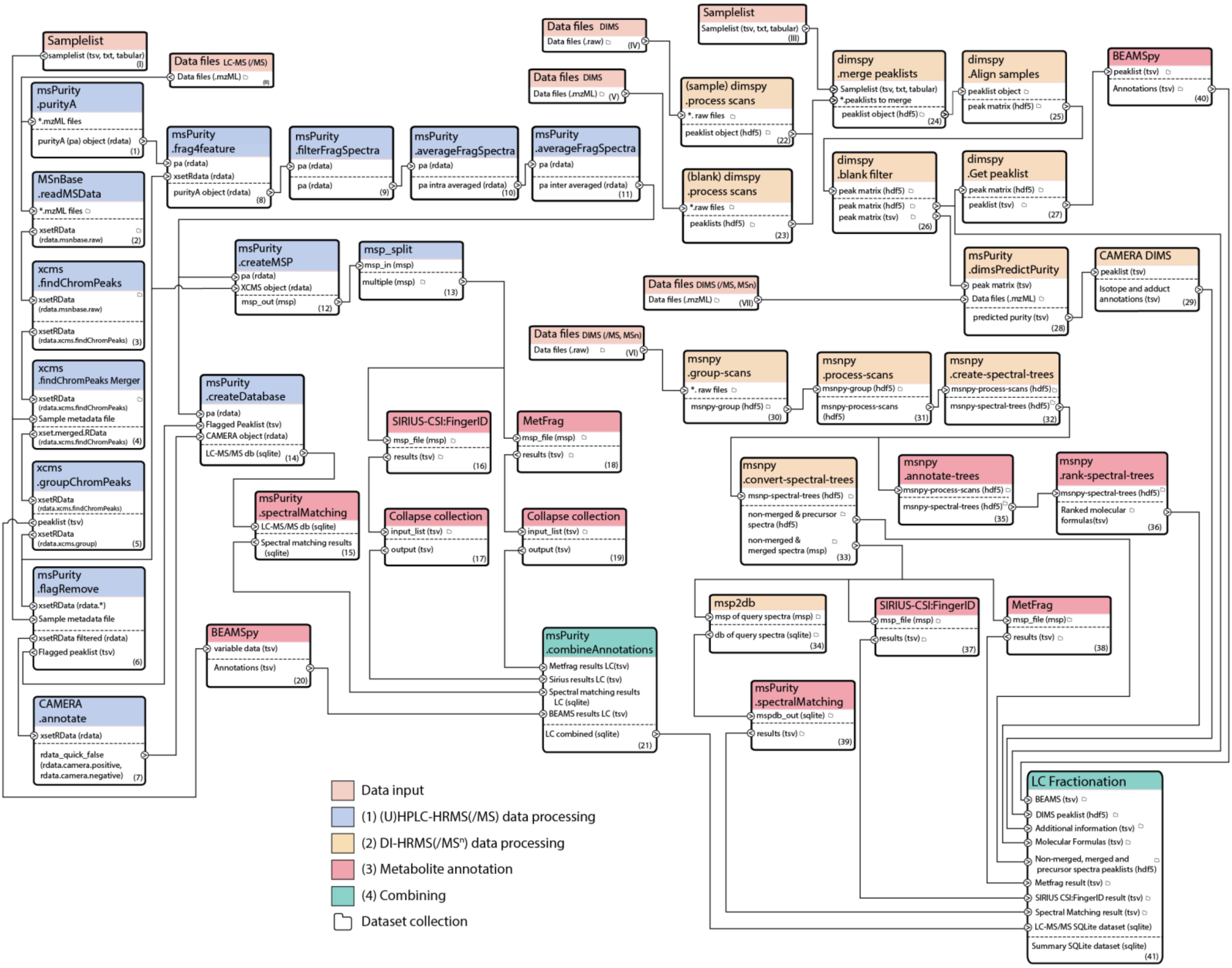


***Figure 3: Deep Metabolome Annotation data processing and metabolite annotation Galaxy workflow.*** *Components are separated by colour into "Data input", "(U)HPLC-HRMS(/MS) data processing", "DI-HRMS(/MS$^n$) data processing", "metabolite annotation" and "Combining". See* ***Supplemental Table S9*** *for description of each tool.*

*ALT TEXT: Graphical representation of the deep metabolome annotation Galaxy workflow.*

### *2.2.3 mzCloud library search*

All annotations derived from mzCloud were performed programmatically in batches using mzCloud proprietary software [32]. Each fragmentation scan was treated individually, and spectral matching was performed against the mzCloud database. The results were saved as an SQLite database with reference to both the query and library spectra of each annotation. The data were filtered to only include “endogenous” metabolites and spectral matches with a spectral similarity score greater than 0.7.

### *2.2.4 GNPS library search*

Fragmentation spectra were searched against the GNPS library spectra using the online workflow on the GNPS website [11], [33]. The precursor ion mass tolerance was set to 0.02 Da and a MS/MS fragment ion tolerance of 0.02 Da. Additionally, spectral matches were filtered to have an error of 10 ppm or less between the library precursor *m/z* and the query precursor *m/z*. Annotations were further restricted to spectral matches in which the library and query spectra were acquired on mass spectrometers operating in the same ionisation mode.

### *2.2.5 GNPS molecular network analysis*

A molecular network was generated using the online workflow on the GNPS website [11], [33]. The data were filtered to remove all fragment ions within ±17 Da of the precursor *m/z*. Fragmentation spectra were window-filtered by retaining only the top six fragment ions in the ±50 Da window across the spectrum. Both the precursor ion mass tolerance and fragment ion tolerance were set to 0.02 Da. A network was then constructed where edges were filtered based on having a cosine score greater than 0.7 and more than two matched peaks. Further, edges between two nodes were only kept in the network if each of the nodes appeared in each other's respective top ten most similar nodes. Finally, the maximum size of a molecular family was set to 100, and the lowest scoring edges were removed from molecular families until the molecular family size was below this threshold. The fragmentation spectra in the network were then searched against GNPS’s spectral libraries. The library spectra were filtered in the same manner as the input data. All matches kept between network and library spectra were required to have a score greater than 0.7 and at least two matched peaks. Further annotation was performed using the Dereplicator tool [34] and the MS2LDA [35] workflow to determine common mass motifs.

### *2.2.6 Combining and summarising all annotations*

Five main sources of annotations were combined into a final list of Metabolite annotations, including Galaxy workflow annotations, GNPS workflow annotations, mzCloud annotations, NMR annotations and GC-EI-HRMS annotations. All data were combined into a single table encompassing annotations from all assays and a final stage of filtering was performed as described in **Supplemental Section 1.14.**

All annotations were chemically classified using ClassyFire [36]; all reported terms at the “superclass”, “class”, and “subclass” levels are used as defined in the ClassyFire taxonomy.

### *2.2.7 Comparison to other metabolite databases*

The final list of *D. magna* metabolites were compared with compound lists from KEGG [37], [38], [39], ChEBI [40], HMDB [8] and MTox700+ [41]. ChEBI was filtered to include only compounds with a known species origin. PhyloT was used to generate the species phylogenetic tree using the NCBI taxonomy [42]. Matching was based on compounds sharing the same partial InChIKey (i.e. the first block of the InChIKey that encodes the molecular skeleton).

QIAGEN Ingenuity Pathway Analysis (IPA, QIAGEN Inc.) was used to derive metabolite-pathway associations. A metabolite list containing PubChem, HMDB and KEGG identifiers was imported into the IPA software. ‘Metabolomics core analysis’ was then conducted using all mapped metabolites.

## 2.3 Assessment of the computational and experimental DMA workflow with metabolite reference standards

Metabolite reference standards (see **Supplemental Table S11)** were analysed to evaluate the effectiveness of the overall DMA workflow, specifically the (U)HPLC-HRMS(/MS) component of the experimental workflow, including extraction, chromatographic separation, and mass spectrometric analysis. The same computational workflow was applied as used for the *D. magna* samples. Next, the measured metabolite reference standards were compared to the expected annotations, for each assay, by matching the partial InChIKey.

## 2.4 Deep metabolome annotation database and web portal (DMAdb)

The DMA database and web portal (DMAdb [43]; **Figure 4**) was developed to organise, manage, and disseminate DMA datasets and associated metabolite annotations. It was implemented using Django, a high-level Python web framework, and comprises multiple applications (i.e. django-gfiles, django-galaxy, and django-mogi [44], see Availability of Supporting Source Code and Requirements for further details). Experimental datasets were structured using the Investigation/Study/Assay (ISA) framework to support standardised organisation of experimental data and metadata. This structure enables consistent metadata capture, facilitates interoperability with community standards, and enables traceability between raw data, processing workflows, and metabolite annotations. DMAdb was primarily designed as an internal, administrator-restricted data management platform with Galaxy workflow integration. However, controlled public access to selected functionalities is provided to enable basic exploration and searching of DMA datasets, as described in the Results section.

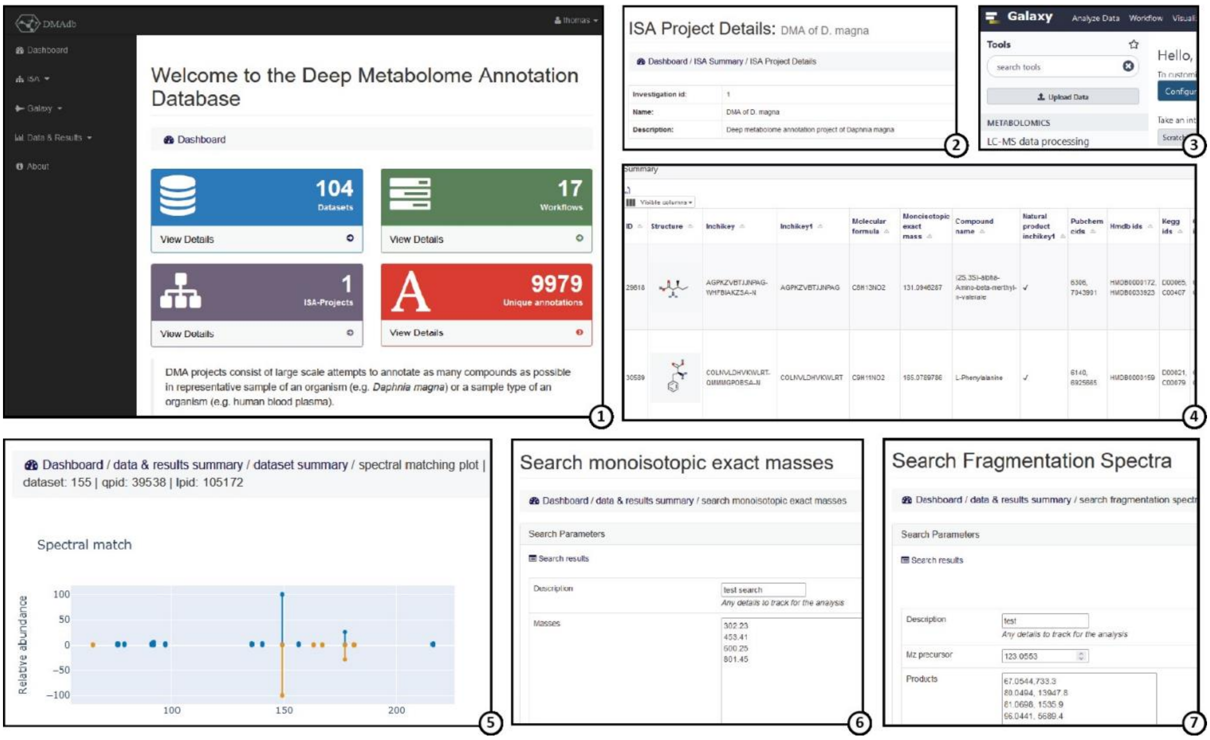


***Figure 4. Overview of the Deep Metabolome Annotation database and web portal (DMAdb) developed and implemented to organise, manage, and disseminate DMA data and associated metabolite annotations of Daphnia magna.*** *(1) Dashboard view of the DMAdb interface. (2) Investigation/Study/Assay (ISA) representation of the DMA data, capturing relevant ontologies, protocols, and experimental processes. (3) Data processing and annotation workflows performed via the Galaxy platform, with outputs uploaded to and stored within DMAdb. (4–5) Browsing and visualisation of metabolite annotations and associated raw data files. (6–7) Search functionalities supporting exact mass queries and MS/MS-based spectral matching.*

*ALT TEXT: Screenshots of the different web interfaces of the Deep Metabolome Annotation database and web portal (DMAdb).*

# 3 Results and discussion

## 3.1 (U)HPLC-HRMS(/MS) method optimisation

The PHE and AMD (U)HPLC-HRMS(/MS) methods were optimised to improve detection of reliable metabolic features and support reproducible fractionation. Full details and supporting figures are provided in **Supplemental Section 2.1, Supplemental Table S12** and **Figures S9-S26**.

## 3.2 Summary of all *D. magna* metabolite annotations and compound classifications

In total, 8,181 unique metabolite annotations are reported from all experimental assays (including (U)HPLC-HRMS(/MS), DI-HRMS(/MS$^{n}$), 1D- & 2D-NMR and GC-EI-HRMS), summarised in **Supplemental Table S13**. The combined annotations and compound classifications across all technologies and approaches are presented here, with further details including specifics for each measurement technology provided in **Supplemental sections 2.2-2.5.** In summary, the majority of annotations were reported for the (U)HPLC-HRMS(/MS) and DI-HRMS(/MS$^{n}$) datasets, observing 8,132 unique annotations compared with four unique metabolites for GC-EI-HRMS and three for 1D- & 2D-NMR; see Venn diagram in **Supplemental Figure S27**. Although the discrepancy in observed annotations across these analytical technologies appears large, with annotations derived from (U)HPLC-HRMS(/MS) and DI-HRMS(/MS$^{n}$) dominating the counts, this is consistent with the analytical prioritisation of these technologies and the comparatively more extensive computational interrogation applied to them.

Compound classification via ClassyFire was possible for 7,934 (97%) metabolites to at least the “Superclass” level. The remaining unclassified metabolites had either incompatible SMILES for ClassyFire or lacked a SMILES annotation from PubChem. See **Figure 5A** for a treemap summarising all annotation superclasses and classes, demonstrating the diverse biochemical space observed. The chemical space was further explored using principal component analysis (PCA) of the PubChem molecular fingerprints, showing broad clustering of metabolites based on their structure, with no obvious outliers (see **Figure 5B**).

The most common “superclass” observed was lipids and lipid-like molecules (3,202 uniquely annotated metabolites) followed by organic acids and derivatives (2,173); organoheterocyclic compounds (656); organic oxygen compounds (572); benzenoids (489); phenylpropanoids and polyketides (306); organic nitrogen compounds (221); nucleosides, nucleotides, and analogues (185); organosulfur compounds (44); alkaloids and derivatives (32); hydrocarbons (17); organophosphorus compounds (17); lignans, neolignans, and related compounds (11); and three other “superclasses” with six or fewer annotated metabolites. A total of 247 metabolites could not be classified to a “superclass” level.

The most common “class” classification observed was for carboxylic acids and derivatives (1,776) followed by fatty acyls (913); glycerophospholipids (803); organooxygen compounds (567); glycerolipids (564); prenol lipids (406); benzene and substituted derivatives (329); sphingolipids (282); organonitrogen compounds (221); steroids and steroid derivatives (217); peptidomimetics (170); phenols (72); macrolides and analogues (68); indoles and derivatives

(67); organic sulfuric acids and derivatives (65); diazines (58); purine nucleosides (52); and 179 other “classes" with 46 or fewer annotated metabolites. A total of 269 metabolites could not be classified to a “class” level.

The most common “subclass” classification was for amino acids, peptides, and analogues (1,681) followed by triradylcglycerols (434); carbohydrates and carbohydrate conjugates (374); glycerophosphocholines (330); glycerophosphoethanolamines (282); fatty acids and conjugates (226); fatty acid esters (142); glycosphingolipids (141); fatty amides (140); amines (122); fatty alcohols (120); fatty acyl glycosides (97); ceramides (96); depsipeptides (92); lineolic acids and derivatives (87) and 338 other subclasses with 82 or fewer annotated metabolites. A total of 838 compounds could not be classified to a “subclass” level. The top 12 most common superclasses, classes and subclasses that have been annotated are shown in **Figure 5C-E.**

We note that some of the metabolites annotated here are likely to originate from organisms other than *D. magna*. These may include bacteria (exogenous and microbiota), fungi or parasites that were potentially present in the non-axenic cultures used (despite daily visual inspections to identify and remove cultures with potential contamination), as well as residual algal feed material in the digestive tracts of the *D. magna*. Indeed, some metabolites were assigned compound superclasses typically associated with the plant kingdom, e.g. 306 phenylpropanoids and polyketides and 32 alkaloids and derivatives, though biosynthesis within the animal kingdom is known [41] and some of these annotations could potentially be ‘false positive’ assignments. Metabolic flux analyses, or radio-labelled isotope tracer experiments, would likely be required to definitively determine the exact origins of each metabolite reported. Alternatively, sterile culturing conditions coupled with the use of dextran beads to purge algal feed material from the digestive tracts of *Daphnia* may prove helpful in focussing future DMA-like experiments on metabolites that are exclusively present in and/or produced by *D. magna*.

(U)HPLC-HRMS(/MS) and DI-HRMS(/MS$^n$) annotations were predominantly derived from spectral matching, MetFrag and SIRIUS CSI:FingerID annotation approaches. SIRIUS CSI:FingerID produced the most unique annotations with MetFrag and spectral matching having similar counts of unique annotations (see **Supplemental Section 2.3 and Supplemental Figure S28** for further details). This finding should not be interpreted as identifying the most effective metabolite annotation method, as the number of annotations observed can change dramatically based on filtering criteria and the compound and spectral libraries used. Rather, this simply illustrates the origins of the annotations in this *D. magna* DMA project. The differing results between the annotation approaches does, however, caution against relying on only a single method, both in terms of the breadth of coverage of the tool used and the potential reliability of annotations.

By using a range of metabolite annotation approaches, the confidence in the resulting annotations can be adjusted according to user preference. For example, if only annotations reported by either MetFrag or spectral matching are considered (i.e., disregarding annotations derived only from SIRIUS CSI:FingerID that may yield more false positives amongst the large number of annotations reported), it would result in 3,591 annotations (or 3,601 if the 1D- & 2D-NMR and GC-EI-HRMS annotations are included). Alternatively, annotations can be filtered even more strictly by specifying that they should be observed

with at least two of the three fragmentation data analysis approaches, resulting in 1,286 annotations (or 1,301 if all 1D- & 2D-NMR and GC-EI-HRMS annotations are also included). These subsets of annotations could be considered more reliable, though it is important to highlight that the full set of 8,181 annotations observed from all fragmentation-based annotation approaches were derived from sufficiently unique fragmentation spectra to yield this high number of unique metabolite annotations. While some annotations may be less reliable (i.e., from SIRIUS CSI:FingerID only), the number of unique fragmentation spectra demonstrates the richness of the *D. magna* metabolome.

Additionally, de novo molecular formula annotation using MSnPy was performed on the DI-HRMS(/$MS^n$) data via the MSnPy spectral annotation functionality within the Galaxy workflow. The DI-HRMS(/$MS^n$) data are particularly suited to this approach due to the high level of measurement replication at different collision energies and fragmentation levels. A total of 40,240 unique molecular formulae were annotated to an MSnPy rank of 1 (32,672 observed from positive ionisation mode and 9,768 from negative mode). Ranking was based on the application of common “consistency” rules for filtering formulae and neutral losses [45], which use fragmentation tree consistency to evaluate and rank the most plausible molecular formulae. Only molecular formulae were included where there were 10 or fewer possible top-ranked candidates. It should be noted that multiple molecular formulae can share a rank of one and that, despite stringent filtering, false positives will be present. It is also worth noting that this stringent filtering will have excluded many annotations for higher-mass precursor ions, which tended to produce excessively large sets of candidate molecular formulae, thereby preventing a high number of false-positive annotations. This approach does not rely on prior knowledge of spectral libraries or compound databases and therefore provides a potentially less biased insight into the biochemistry of the metabolome, albeit limited to the level of molecular formula (i.e., non-structural) annotation.

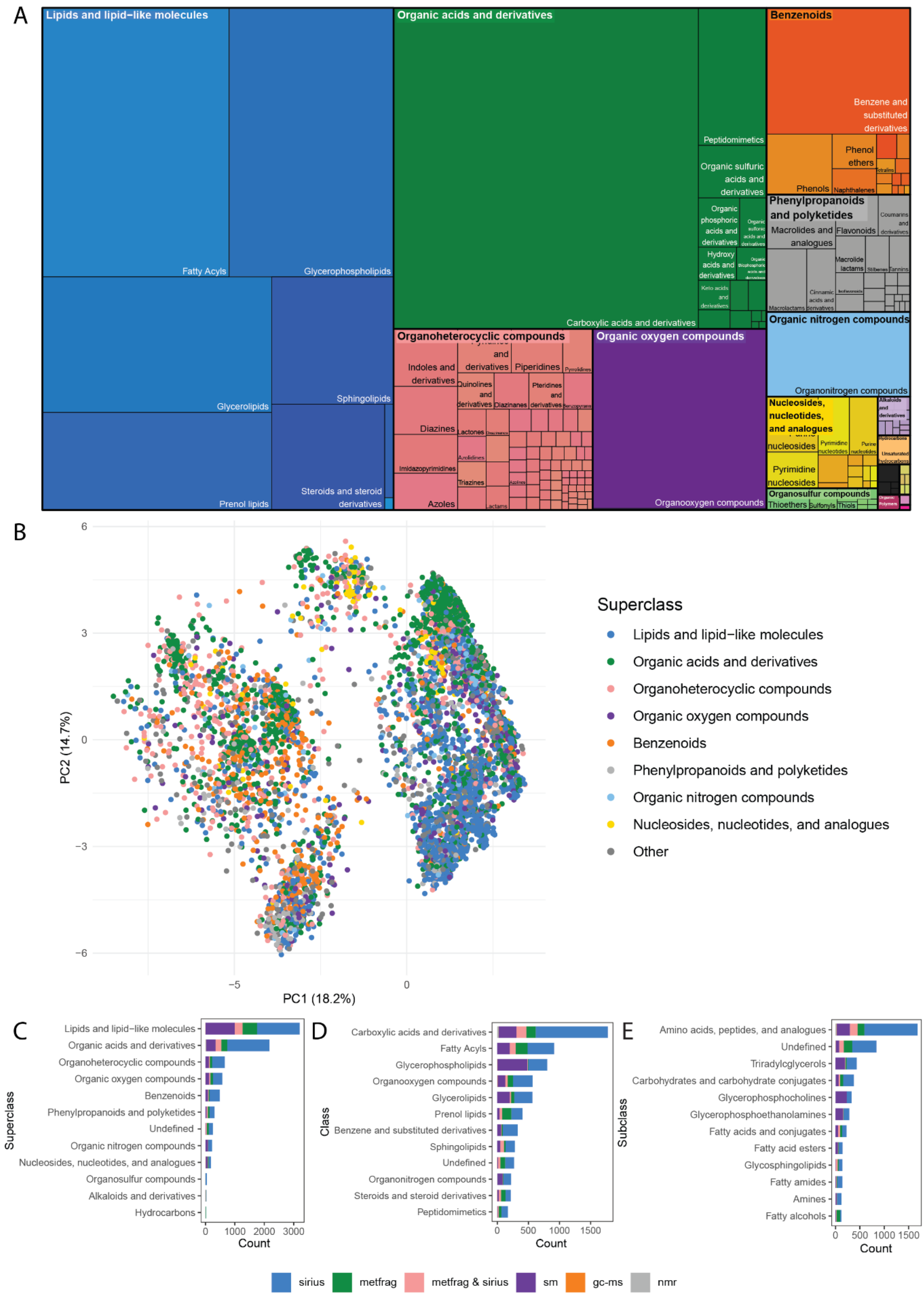


***Figure 5: Compound classifications for the 8,181 D. magna annotated metabolites. A)*** *Treemap of metabolite 'superclasses' and 'classes' for the metabolite annotations.* ***B)*** *Principal component analysis of*

*PubChem fingerprints for all unique metabolite annotations: colour represents the superclass classification of the metabolite annotation.* ***C)*** *Count of unique metabolite annotations for 'superclass' compound classifications. Showing the top 12 'superclasses'****,*** *colour represents the annotation approach used (annotation was either derived using SIRIUS CSI:FingerID, MetFrag, SIRIUS CSI:FingerID & MetFrag, Spectral matching, GC-EI-HRMS or 1D- & 2D-NMR).* ***D)*** *Count of unique metabolite annotations for 'class' compound classifications. Showing the top 12 'classes'****,*** *colour represents the annotation approach used (annotation was either derived using SIRIUS CSI:FingerID, MetFrag, SIRIUS CSI:FingerID & MetFrag, Spectral matching, GC-EI-HRMS or 1D- & 2D-NMR).* ***E)*** *Count of unique metabolite annotations for 'subclass' compound classifications. Showing the top 12 'subclasses', colour represents the annotation approach used (annotation was either derived from SIRIUS CSI:FingerID, MetFrag, SIRIUS CSI:FingerID & MetFrag, Spectral matching, GC-EI-HRMS or 1D- & 2D-NMR).*

*ALT TEXT: Graphs summarising the different compound classifications of metabolites annotated through deep metabolome annotation of D. magna*

---

## 3.3 Metabolites and compound classes physicochemically separated by DMA experimental workflow

The extent of physicochemical separation achieved by the DMA experimental workflow was evaluated to determine the effectiveness, or potential redundancy, of components within the workflow (see **Figure 6**).

When combining all annotations from either the polar or apolar arm of the workflow (see **Figure 6A**), both arms generated a substantial number of unique metabolite annotations (with 4,305 and 3,503 metabolites unique to the polar and apolar arms respectively, and only 373 metabolites shared). This finding highlights the importance of the extraction procedure within the workflow and the necessity of both arms of the workflow to provide a comprehensive view of the metabolome. When combining all annotations from each chromatography approach used (see **Figure 6B**), the C30 column yielded the highest number of metabolite annotations (3,876 in total, of which 3,503 were unique to the column). The PHE analysis resulted in 3,590 metabolite annotations (2,404 unique to the column) while analyses performed using the AMD column resulted in the lowest number of metabolite annotations (2,049, of which 1,029 were unique to this column).

Considering the ionisation modes used (see **Figure 6C**), analysis using positive ionisation mode produced 5,846 metabolite annotations (4,902 were unique to this mode), compared to 3,270 metabolite annotations using negative ionisation (2,326 unique to this mode). This finding evidences the need to include both ionisation modes in the DMA workflow. Unsurprisingly, for both ionisation modes the higher mass ranges (>600 Da) are dominated by lipids and lipid-like molecules, whereas for mass ranges <600 Da the organic acids and derivatives are most prominent, along with lipids and lipid-like molecules. Other superclasses with mass <600 Da include organoheterocyclic compounds; organic oxygen compounds; benzenoids; phenylpropanoids and polyketides; organic nitrogen compounds; and nucleosides, nucleotides, and analogues. **Supplemental Figure S29** shows the distributions of unique annotations against the exact mass of the annotation.

When examining each assay (see **Figure 6D**), in all cases the analysis of the crude *D. magna* extract (without SPE fractionation) resulted in the highest number of annotations compared to the individual SPE fractions. This is to be expected as the SPE fractions were intended to separate the metabolites according to their physicochemical properties and so by design will separate the metabolites across fractions. **Figure 6D** also demonstrates that lipids and lipid-like molecules can be seen to dominate the apolar arm, whereas organic acids and derivatives are the most prominent in the polar arm. This is also expected based on the chemistry of the liquid-phase extractions, solid-phase extractions and chromatography used, which favour apolar metabolites.

**Figure 6E** shows an UpSet plot detailing the overlap of annotations across assays (with positive and negative ionisation modes combined). The most striking observation is the varying number of annotations per assay, with the SPE fractions from the AMD column providing the lowest number of annotations. This assessment could form a basis for a more streamlined, time-efficient workflow. Given that the measurements of the crude extracts yielded 581, 568 and 513 unique annotations (for apolar crude C30, polar crude AMD and polar crude PHE respectively) illustrates their valuable contribution to the DMA workflow.

To provide added confidence in the ability of the experimental and computational workflow's ability to annotate the metabolome an assessment of the workflow was also made using 48 chemical reference standards covering a wide biochemical space including lipid and lipid-like molecules; organic acids and derivatives; organic oxygen compounds; organoheterocyclic compounds; nucleosides, nucleotides, and analogues; and organic nitrogen compounds. The spread of the compound classes across the experimental workflow mirrors what is observed in the *Daphnia* samples, where lipids and lipid-like molecules dominate the apolar arm and organic acids and derivatives being the most prominent in the polar arm. As the majority of metabolite reference standards were observed by the DMA workflow (89.6%), we deemed that both the experimental and computational workflows were sufficiently reliable at annotating a diverse range of metabolites to be used for annotating *D. magna* metabolome. See **Supplemental Table S11**, **Supplemental Figure S30-31** and **Supplemental Section 2.6** for further details.

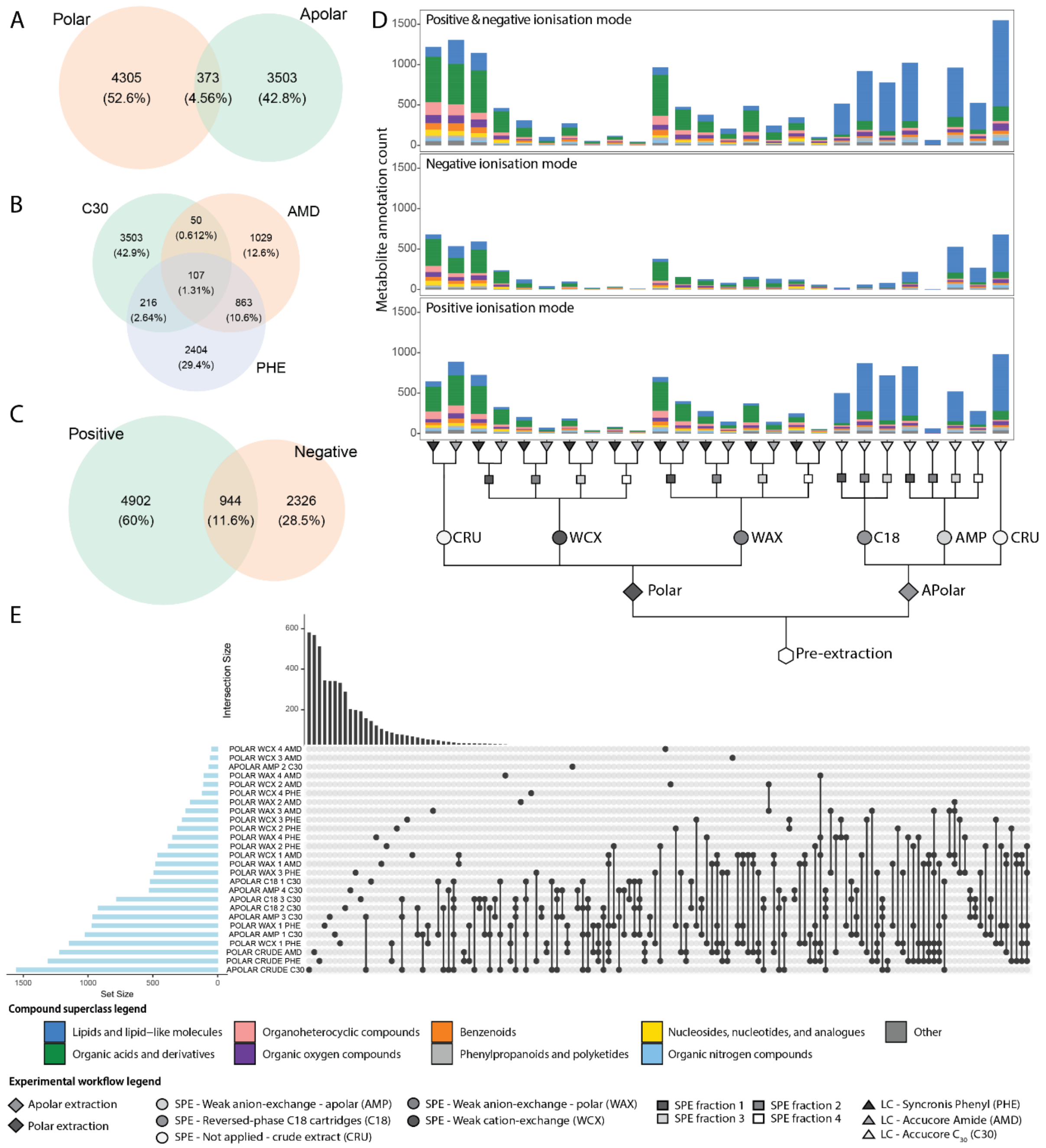


***Figure 6: Contributions of extraction, SPE fractionation, LC separation and mass spectrometric ionisation methods to the number of metabolite annotations for D. magna. A)*** *Venn diagram of metabolite annotations observed across extraction approaches.* ***B)*** *Venn diagram of metabolite annotations observed across all chromatography techniques.* ***C)*** *Venn diagram of metabolite annotations observed across positive and negative ionisation modes.* ***D)*** *Count of metabolite annotations across experimental workflow components: bar charts shown for positive ionisation mode, negative ionisation mode and combined positive and negative ionisation modes. Colour represents the superclass compound classification of the annotations. See bottom of figure for colour code used for compound superclass.* ***E)*** *UpSet plot summarising the overlap of metabolite annotations between assays (positive and negative assays have been combined).*

*ALT TEXT: Graphs summarising the number of metabolite annotations resulting from each experimental component of the D. magna deep metabolome annotation.*

## 3.4 Comparison to other metabolite databases

The metabolite annotation results from the DMA of *D. magna* were compared to public resources of relevant metabolites from different species, as summarised in **Figure 7**. Even given this relatively limited number of metabolites known for different organisms, it is readily apparent that some metabolites are widely shared across organisms due to the conservation of metabolism (i.e., phylometabolomics). From ChEBI, the top six species that overlap with *D. magna* DMA annotations are *Homo sapiens*, *Saccharomyces cerevisiae*, *Mus musculus, Escherichia coli, D. magna* and *Chlamydomonas reinhardtii (a single-celled green algae)*. For *H. sapiens, M. musculus*, *S. cerevisiae* and *E. coli* this is partly explained by these species being the most represented within ChEBI. However, overlap with the previously known *D. magna* and algae metabolites (which are the diet of the cultured *D. magna*) adds confidence to the existing annotations and supports the effectiveness of the DMA workflow.

As *D. magna* is used internationally as an ecotoxicology test species, the metabolite annotations reported using the DMA workflow were compared to those in MTox700+, a metabolite list of toxicologically-relevant metabolites derived from mammalian studies. Overlapping metabolites may help interpret the toxicological perturbations measured in *D. magna* metabolomics studies. A total of 293 of 722 metabolites were matched to a full InChIKey (or 346 of 722 if using the first section of the InChIKey).

In addition to the above, the 8,181 annotated metabolites were investigated to assess coverage of known pathways. Whilst we acknowledge the limitations of this analysis due to the limited knowledge of pathways for *Daphnia*, preliminary analysis using the QIAGEN IPA software was still able to identify 113 pathways having ≥ 50% coverage with ≥ 3 measured metabolites (see **Supplemental Figure S32**), supporting the relevance of the annotated metabolites for pathway-level toxicological interpretation.

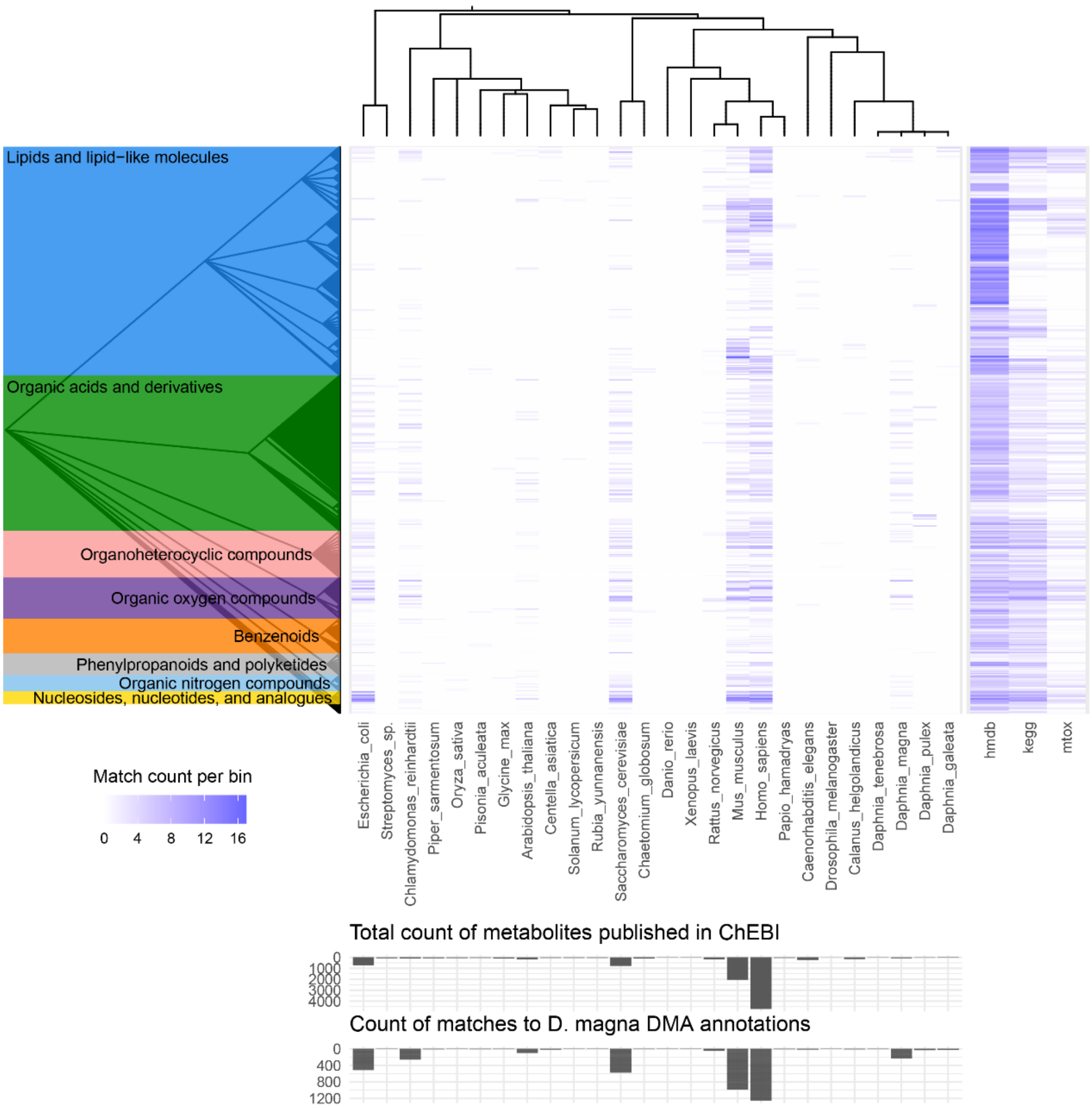


***Figure 7: Overlap of D. magna metabolite annotations reported using the DMA workflow with known metabolites from other species. Left** – all metabolite annotations from the DMA of D. magna presented in a hierarchical tree of superclasses, classes and subclasses. **Top** – phylogenetic tree of known metabolites from 26 species derived from ChEBI. **Centre** – heatmap of the counts of metabolites matched between the species derived from ChEBI with the D. magna metabolites from the DMA workflow (binned into sets of 25 compounds). **Right –** heatmap of matches observed between HMDB, KEGG and MTox700+ and the D. magna metabolite annotations reported using the DMA workflow. **Bottom –** counts of the metabolites published with ChEBI for each organism and below that the counts of the matches to DMA of D. Magna annotations.*

*ALT TEXT: Graphs showing how the D. magna deep metabolome annotations overlap across other species from known metabolomes.*

## 3.5 Molecular network analysis using GNPS

Molecular networks were generated using the GNPS network analysis workflow, with classical molecular networking, MS2LDA, Dereplicator+ and MolNetEnhancer (see **Figure 8** for summary of negative ionisation molecular networks and **Supplemental Figure S33** for positive ionisation molecular networks). These networks provide an overall picture of the diversity of the fragmentation spectra collected (and thus the diversity of metabolites observed in *Daphnia*) without being wholly dependent on obtaining a compound or compound class annotation. The dataset exhibited a large diversity of fragmentation spectra (ca. 31,000 fragmentation clusters for positive ionisation mode and ca. 5,000 distinct fragmentation clusters for negative ionisation mode). A similar overview can be achieved using MS2LDA mass-motifs, for which ca. 55,000 “mass motifs” are observed for positive ionisation data and ca. 3,900 for the negative ionisation data. However, only a small subset of the clusters could be annotated (e.g., ca. 1,000 clusters annotated via spectral matching for positive ionisation spectra and ca. 200 clusters annotated for negative ionisation spectra). The remaining unannotated spectra may reveal additional insights into the *Daphnia* metabolome as spectral libraries and computational approaches for annotation improve. In particular, the annotations could be improved with further integration with MetFrag and SIRIUS CSI:FingerID, but this was beyond the scope of this analysis. The spectral networks also demonstrate the potential for future analysis where multiple networks created from other model organism DMA projects could be compared for cross-species phylometabolomics analysis that would be driven by spectral similarity as opposed to being reliant on metabolite annotations.

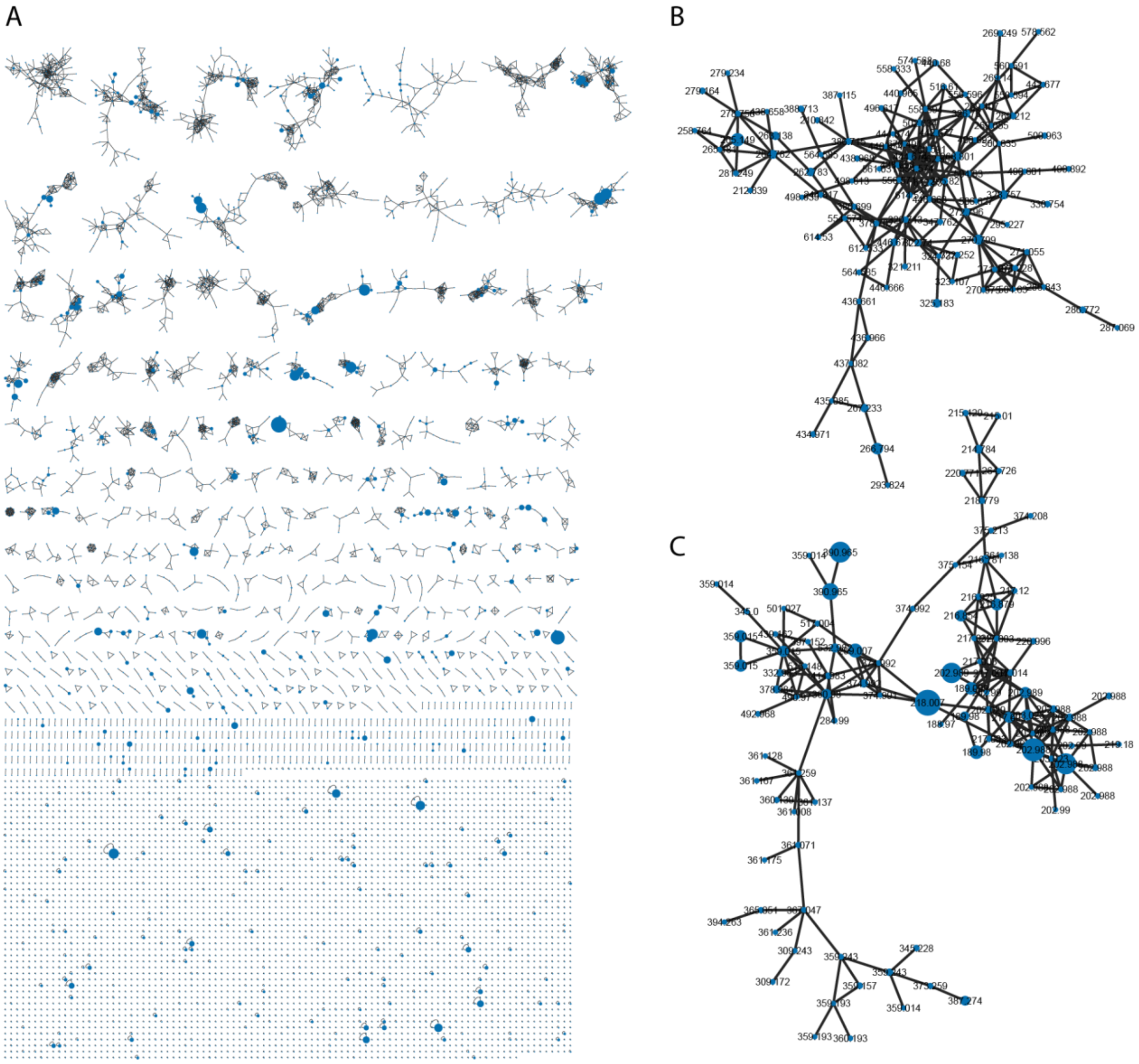


***Figure 8. GNPS molecular network derived from D. magna deep metabolome annotation negative ionisation fragmentation spectra. A)*** Overview of negative ionisation mode molecular networks generated from GNPS molecular network analysis, showing all 5,320 distinct clusters. The node size is proportional to the number of spectra that contribute to the node. The top 2 clusters (based on how many nodes were observed) are shown in more detail to highlight the precursor *m/z* associated with the node. **B)** Largest cluster observed. **C)** Second largest cluster observed.

*ALT TEXT: Networks of the negative ionisation mass spectrometry fragmentation data for the D.magna deep metabolome annotations.*

## 3.6 DMAdb: Public access and functionalities

DMAdb [43] serves as a data management environment encompassing raw data, processing workflows, and metabolite annotations (**Figure 4**). This integrated framework enhances reproducibility and supports transparent data provenance. By structuring DMA data according to the ISA model, DMAdb provides standardised representations of experimental datasets, thereby promoting interoperability and enabling structured exploration of associated metadata. Public users can access selected functionalities without registration, including browsing ISA-organised datasets and performing basic searches of metabolite annotation records. Registered users are provided with extended functionalities, including access to processed datasets at the individual assay level, derived from the corresponding Galaxy histories. Advanced functionalities further include batch-based exact mass searches across annotated metabolites and MS/MS spectral matching against processed and annotated fragmentation spectra. Although developed specifically for this study to support systematic investigation of the metabolic complexity of *D. magna*, DMAdb establishes a structured foundation for ongoing refinement and future expansion, offering a framework that can be adapted for use with additional model organisms.

## 4 Conclusion

The extensive experimental and computational tools and workflows developed and applied here have generated one of the largest metabolite annotation datasets ever published and provide the first comprehensive list of metabolites thought to be present in the ecotoxicologically important model organism, *D. magna*. The reported 8,181 metabolites (1,301 to 3,601 if using more stringent filtering criteria), covering predominantly the endogenous *Daphnia* metabolites and potentially some amount of the algae food source and gut microbiome (e.g. the 306 phenylpropanoids and polyketides and the 32 alkaloids and derivatives*),* represent a significant step forward in understanding the metabolic complexity of *Daphnia*.

The dataset also provides a substantial resource of mass spectrometry fragmentation data focused on a single organism, which can be used for re-analysis and as a source of annotations when compared with other fragmentation datasets, e.g. for cross-species metabolome comparisons (phylometabolomics). Additionally, we have established Galaxy workflows and tools to process and annotate not only this dataset, but also other mass spectrometry fragmentation datasets. Reproducing this complex, multi-step workflow within Galaxy would require substantial effort, particularly for users unfamiliar with the workflow management platform or the specific configuration requirements of this study. However, the sharing of full Galaxy histories and workflows is intended to provide transparency and support methodological inspection and reuse where appropriate. We also note that SIRIUS CSI (version 4), as used here, is no longer publicly accessible, as it depended on an API endpoint that has since been deprecated and is no longer supported. Despite these caveats, the Galaxy tools developed here have already been applied in subsequent metabolomics studies, demonstrating their practical utility [46], [47].

We also present DMAdb as a data management environment for raw data, processing steps, and metabolite annotations, enabling controlled public access to ISA-organised datasets alongside extended functionality for registered users. Although developed specifically for this study, it establishes a potential foundation for the organisation and exploration of future DMA datasets, with application beyond *D. magna.*

While our computational workflow is extensive, encompassing multiple techniques and approaches, it has several limitations. In particular, bias arises from the choice of software, software versions, parameter settings, library choice and filtering approaches, all of which influence the final set of reported metabolite annotations. The level of lenience applied in these choices affects the reliability of the annotations, and it is anticipated that some false positives are reported here, i.e., not actual endogenous *Daphnia* metabolites but structurally similar enough to generate an annotation. Purchasing thousands of metabolite reference standards to confirm the annotations is not currently feasible, due to the limited commercial availability of such standards. Also, although steps were taken to minimise background and contaminant signals, further work could explore more stringent criteria. As more metabolite annotations are reported for model organisms, and as their validity becomes established, the reliability of the annotations from the *Daphnia* DMA project can be further evaluated. Improvements to the computational workflow could include greater use of isotopic patterns and in-source fragmentation to reduce spurious annotations [48]; further integration of the underlying software packages into other mass spectrometry-based data analysis suites [49];

and integration with GNPS networking tools where networks could be generated from averaged spectra produced (e.g. via msPurity and MSnPy) rather than individual scans, followed by incorporation of annotations from MetFrag and SIRIUS CSI:FingerID.

The experimental methods described here provide a means to obtain a single homogenous sample representing an organism's metabolome, which is then characterised through extensive physicochemical separation and bioanalytical measurements. The experimental workflow developed here can be applied in full or adapted in part, depending on the resources available. For example, where sample material is limited, a subset of the SPE methods and/or (U)HPLC-HRMS(/MS) approaches may be selected based on which metabolite classes are of interest, or for a more rapid DMA project the SPE component could be omitted. We acknowledge that even with this extensive workflow there are limitations in how many metabolites can be fragmented, reducing the number of metabolites that can be annotated using fragmentation-based approaches. However, advances in mass spectrometry technologies may help to address this issue, e.g. the Thermo Scientific Orbitrap ID-X Tribrid mass spectrometer is capable of extensive $MS^n$ analysis, while current generation Time of Flight / Astral instruments support MS/MS acquisition at up to 250 Hz.

The present study is unlikely to provide an exhaustive list of all possible metabolites present in *D. magna*. In part, this is due to limitations of current analytical platforms, which typically detects only a subset of an organism's metabolome, e.g. pigment molecules are often difficult to detect using liquid chromatography mass spectrometry. At the same time, while ten *D. magna* strains were included in the present work to maximise the breadth of *D. magna* metabolome annotation achieved, and to capture genetic diversity across the species, many of these strains are derived from European *Daphnia* populations. *D. magna* strains from diverse geographic regions (e.g. East Asia, South Africa, and North America), may very well produce a plethora of unique metabolites that are currently missing from the annotations described here. Accordingly, the DMA of *D. magna* should be expanded to include metabolite annotation data, collated using a comparable DMA workflow, both for *Daphnia* originating from a wider variety of geographic locations, and for *Daphnia* maintained under a greater range of environmental or exposure conditions. This would enhance the applicability of this work to *D. magna* metabolomics studies worldwide, while also facilitating exploration of the genetic and environmental basis of metabolic diversity across *D. magna* populations.

Despite the limitations described above, the DMA workflow described here and applied to *D. magna* provides both a resource and a valuable catalyst for future deep metabolome annotation studies of other model organisms.

# 5 Availability of Source Code and Requirements

Project name: dmagna-dma-paper

Project homepage: https://github.com/computational-metabolomics/dmagna-dma-paper

License: GPL-3.0 license

Operating system(s): Operating system independent

Package management: CRAN, Bioconductor and renv

Programming language: R

Hardware requirements: 4 CPU cores and 16 GB RAM

See **Table 1** for availability of the Galaxy tools used and developed, and **Supplemental Table S9** for details of each tool.

R (v4.4.3) [50] was used to summarise the annotations and generate **Figures 5-7**, as well as the supplementary summary figures. The code used for this is available via the "dmagna-dma-paper" GitHub repository, where all package requirements are detailed. Key R packages used include: ggplot2 (v3.5.2) [51], which was used throughout the analysis for the generation of plots; UpSetR (v1.4.0) [52] for UpSet plots; VennDiagram (v1.7.3) for Venn diagrams; Treemap (v2.4.4) for treemaps; ggtree (v3.14.0) [53], ape (v5.8.1) [54] and aplot (v0.2.8) [55] were used to generate the plots comparing the DMA of *D. magna* metabolites to the phylogenetic tree of relevant species and map to relevant databases and resources; ChemmineR [56] (v3.58.0) was used to extract the PubChem fingerprints and mol files from PubChem [57], principal component analysis (PCA) was then performed on these fingerprints with the R "prcomp" function. Additionally, this repository includes an example illustrating how data from the Galaxy histories may be accessed programmatically, including retrieval of XCMS peak matrices and the inspection of their relative peak intensities.

# 6 Availability of Resources and Requirements

Project name: DMAdb – Documentation (and Django packages details)

Project homepage: Web portal https://dmadb.bham.ac.uk/; Documentation https://dmadb.readthedocs.io/en/latest/getting-started.html

License: GPL-3.0 license

Operating system(s): Linux

Package management: PyPi

Programming language: Python

Hardware requirements: 2 CPU cores and 4 GB RAM

DMAdb and web portal were developed using three Django applications (django-gfiles, django-galaxy and django-mogi) specifically designed for metabolomics data organisation with Galaxy and the ISA framework. The packages and code used are freely available.

**Table 1:** Galaxy tools - code availability

| Project name (biotools id) | Galaxy tools | Galaxy tool code home page | Underlying software code home page | Licence | Language |
|---|---|---|---|---|---|
| Pre-existing software and Galaxy tools | | | | | |
| MSnBase (biotools:msnbase) | MSnBase.readMSData | https://github.com/workflow4metabolomics/tools-metabolomics | https://www.bioconductor.org/packages/release/bioc/html/MSnbase.html | Underlying software: Artistic-2.0<br>Galaxy tool: GPL-3.0 | R |
| XCMS (biotools:xcms) | xcms.findChromPeaks<br>xcms.findChromPeaks Merger<br>xcms.groupChromPeaks | https://github.com/workflow4metabolomics/tools-metabolomics | http://bioconductor.org/packages/release/bioc/html/xcms.html<br><br>[58] | GPL (>= 2) | R |
| CAMERA (biotools:camera) | CAMERA<br>.Annotate | https://github.com/workflow4metabolomics/tools-metabolomics | https://www.bioconductor.org/packages/release/bioc/html/CAMERA.html<br><br>[59] | GPL (>= 2) | R |
| BEAMSpy (Not available) | BEAMSpy | https://github.com/computational-metabolomics/beamspy-galaxy | https://github.com/computational-metabolomics/beamspy<br><br>https://more.bham.ac.uk/beams/ | GPL-3.0 | R |
| DIMSpy (biotools:dimspy) | dimspy.Process scans<br>dimspy.merge peaklists<br>dimspy.align samples<br>dimspy.blank filter<br>dimspy.Get peaklist | https://github.com/computational-metabolomics/dimspy-galaxy | https://github.com/computational-metabolomics/dimspy | GPL-3.0 | Python |
| Software and / or the Galaxy tool was developed by authors | | | | | |
| †msPurity (biotools:mspurity) | msPurity.purityA<br>msPurity.flagRemove<br>msPurity.frag4feature<br>msPurity.filterFragSpectra<br>msPurity.averageFragSpectra<br>msPurity.createMSP<br>msPurity.createDatabase<br>msPurity.spectralMatching<br>msPurity.combineAnnotations<br>msPurity.dimsPredictPurity | https://github.com/computational-metabolomics/mspurity-galaxy/ | https://www.bioconductor.org/packages/release/bioc/html/msPurity.html<br><br>[26] | GPL-3.0 | R |
| †MSnPy (biotools:msnpy) | MSnPy.group-scans<br>MSnPy.process-scans<br>MSnPy.create-spectral-trees<br>MSnPy.annotate-trees<br>MSnPy.convert-spectral-trees | https://github.com/computational-metabolomics/msnpy-galaxy | https://github.com/computational-metabolomics/msnpy | GPL-3.0 | Python |
| †msp2db (Not available) | msp2db | https://github.com/computational-metabolomics/dmatools-galaxy | https://github.com/computational-metabolomics/msp2db | GPL-3.0 | Python |
| †CAMERA DIMS (Not available) | CAMERA DIMS | https://github.com/computational-metabolomics/dmatools-galaxy | https://github.com/computational-metabolomics/cameraDIMS | GPL (>= 2) | R |

| | | | | | |
|---|---|---|---|---|---|
| *SIRIUS CSI:FingerID (biotools:Sirius) | SIRIUS CSI:FingerID | https://github.com/computational-metabolomics/sirius-csifingerid-galaxy/ | https://bio.informatik.uni-jena.de/software/sirius/<br><br>[30] | Underlying software: GNU AGPL<br>Galaxy tool: GPL-3.0 | Java (and python for Galaxy wrapper) |
| *MetFrag (biotools:metfrag) | MetFrag | https://github.com/computational-metabolomics/metfrag-galaxy/ | https://ipb-halle.github.io/MetFrag/<br><br>[27], [28], [29] | Underlying software: GPL (>= 2)<br>Galaxy tool: GPL-3.0 | Java (and python for Galaxy wrapper) |
| †LC fractionation processor (Not available) | LC fractionation processor | https://github.com/computational-metabolomics/lcfrac-galaxy | https://github.com/computational-metabolomics/lcfrac-galaxy<br><br>(all functionality within Galaxy tool) | GPL-3.0 | Python |
| †§deconrank (Not available) | deconrank | https://github.com/computational-metabolomics/dmatools-galaxy | https://github.com/computational-metabolomics/deconrank | GPL-3.0 | Python |

**Footnotes**: All tools and software described in table are operating system platform independent. *Galaxy tool developed by authors. †New (or updated) underlying software and Galaxy tool developed by authors. §Not used directly in the annotation workflows but was used in the “directed acquisition workflows” described in **Supplemental Section 1.8.** The “msp2db”, “CAMERA DIMS”, “LC fractionation processor” and “deconrank” are considered highly specific to this workflow and currently of limited general applicability, and therefore have not yet been registered in bio.tools.

# 7 Additional files

Supplemental information is available in the accompanying Word (.docx) file, with larger tables provided separately in the Excel (.xlsx) file (**Supplemental Tables S1, S2, S11** and **S13**).

**Supplemental section 1: Materials and methods - further details**

- **1.1: Summary of assays and files**
- **1.2: Chemicals**
- **1.3: Solvents and solutions**
- **1.4: Consumables**
- **1.5: *D. magna* culturing and sample preparation**
- **1.6: Metabolite extraction from homogenised D. magna biomass**
- **1.7: Solid phase extraction-based fractionation of metabolite extracts**
- **1.8: DMA (U)HPLC-HRMS(/MS), DI-HRMS(/MS$^n$) and LC fractionation**
- **1.9: (U)HPLC-HRMS(/MS) method optimisation**
- **1.10: GC-EI-HRMS**
- **1.11: 1D- & 2D-NMR**
- **1.12: DMA computational workflow overview**
- **1.13: DMA Galaxy workflow**
- **1.14: Combining and summarising all annotations**
- **1.15: Assessment of the computational and experimental DMA workflow with metabolite reference standards**

**Supplemental section 2: Results - further details**

- **2.1: (U)HPLC-HRMS(/MS) method optimisation**
- **2.2: Summary of all DMA of *D. magna* annotations**
- **2.3: (U)HPLC-HRMS(/MS) and DI-HRMS(/MS$^n$) derived metabolite annotations**
- **2.4: GC-EI-HRMS derived metabolite annotations**
- **2.5: NMR derived metabolite annotations**
- **2.6: Assessment of the computational and experimental DMA workflow with metabolite reference standards**
- **2.7: Pathway analysis**
- **2.8: Molecular network analysis using GNPS**

**Supplemental Tables:**

- **Table S1:** Assay Summary
- **Table S2:** (U)HPLC-HRMS(/MS) DI-HRMS(/MS$^n$) data files
- **Table S3:** *D. magna* strains
- **Table S4:** High-hardness COMBO and modified high hardness COMBO medium
- **Table S5:** Bold's basal medium
- **Table S6:** Liquid chromatography systems utilised in optimisation of (U)HPLC-HRMS(/MS) methods

- **Table S7:** Mass spectrometer operational parameters for optimisation of (U)HPLC-HRMS(/MS) methods
- **Table S8:** Liquid chromatography operational parameters for optimisation of (U)HPLC-HRMS(/MS) methods
- **Table S9:** Summary of Galaxy tools
- **Table S10:** Summary of fragmentation spectra used for spectral matching with msPurity
- **Table S11:** Metabolite reference standard summary
- **Table S12:** Median and interquartile range of retention times for RDMFs recorded in DMA (U)HPLC-HRMS/MS method optimisation experiments
- **Table S13:** *D. magna* metabolite annotation summary
- **Table S14:** GC-EI-HRMS derived metabolite annotations
- **Table S15:** NMR derived metabolite annotations

**Supplemental figures (methods)**

- **Figure S1**: Solid phase extraction-based fractionation of *D. magna* polar extract (WAX, weak anion-exchange; WCX, weak cation-exchange)
- **Figure S2**: Solid phase extraction-based fractionation of *D. magna* apolar extract (C18, a reversed phase-based fractionation procedure; AMP, a weak anion-exchange-based fractionation procedure
- **Figure S3:** Overview of the data acquisition workflow applied for (U)HPLC-HRMS(/MS) analysis and time-based fractionation of DMA samples
- **Figure S4:** Overview of the data acquisition workflow applied for DI-HRMS(/MS$^n$) analysis of the DMA re-suspended LC fractionation samples.
- **Figure S5**: Sample preparation for (U)HPLC-HRMS(/MS) method optimisation
- **Figure S6:** Overview of computational analysis of DMA (U)HPLC-HRMS(/MS) and DI-HRMS(/MS$^n$) LC fractionation experiments
- **Figure S7:** (U)HPLC-HRMS(/MS) data processing schematic for msPurity and XCMS
- **Figure S8:** DI-HRMS(/MS$^n$) data processing schematic for MSnPy

**Supplemental figures ((U)HPLC-HRMS(/MS) method optimisation results)**

- **Figure S9-26:** Multiple figures detailing the (U)HPLC-HRMS(/MS) method optimisation. Includes 2-dimensional density plot of reproducibly detectable metabolic features (RDMFs) for each method assessed and summary plots of the counts of RDMFs across the different methods.

**Supplemental figures (*D. magna* annotation results)**

- **Figure S27:** Venn diagram of metabolite annotations observed for 1D- & 2D-NMR, GC-EI-HRMS and (U)HPLC-HRMS(/MS) and DI-HRMS(/MS$^n$) measurement techniques
- **Figure S28:** Venn diagram of metabolite annotations observed across computational annotation approach used.

- **Figure S29:** Distribution of unique metabolite annotations across monoisotopic exact mass
- **Figure S30:** Assessment of the DMA experimental and computational workflow
- **Figure S31:** Summary of which annotation approach was able to identify each metabolite standard
- **Figure S32**: Summary of the top canonical pathways derived using QIAGEN Ingenuity Pathway Analysis (IPA) for all annotations obtained from the DMA of D. magna
- **Figure S33:** GNPS spectral network analysis (positive ionisation mode).

# 8 Abbreviations

| | |
|---|---|
| (U)HPLC-HRMS(/MS) | (Ultra)high-performance liquid chromatography-high resolution tandem mass spectrometry |
| 1D- & 2D-NMR | 1- and 2-dimensional nuclear magnetic resonance |
| AMD | Accucore Amide liquid chromatography column |
| AMP | Weak anion-exchange SPE cartridges (apolar arm of workflow) |
| C18 | Reversed-phase C18 SPE cartridges |
| C30 | Accucore C30 RPLC column (C30) |
| CID | Collision-induced dissociation |
| DDA | Data dependent acquisition |
| DI-HRMS(/MS$^n$) | Direct infusion-high resolution mass spectrometry (with multiple-stage fragmentation) |
| DMA | Deep metabolome annotation |
| DMAdb | Deep metabolome annotation database |
| GC-EI-HRMS | Gas chromatography-electron ionisation-high resolution mass spectrometry |
| HCD | Higher energy collisional dissociation |
| HILIC | Hydrophilic interaction liquid chromatography |
| HRMS(/MS) | High resolution mass spectrometry (with tandem mass spectrometry) |
| NCE | Normalised collision energy |
| RPLC | Reverse-phase liquid chromatography |
| PHE | Syncronis Phenyl liquid chromatography column |
| SPE | Solid-phase extraction |
| WAX | Weak anion-exchange SPE cartridges (polar arm of workflow) |
| WCX | Weak-cation exchange SPE cartridges |

# 9 Acknowledgements

We would like to thank several current and former Thermo Fisher Scientific scientists for their helpful advice, including Martin Hornshaw, David Peake, Amanda Souza, Ioanna Ntai and Tim Stratton, as well as Anthony Edge and Alex Adam who co-supervised MRJ's iCASE PhD studentship. We are also grateful to Peter Li from *Gigascience* who co-supervised TNL's iCASE PhD studentship and provided early guidance related to Galaxy and database development. We also thank John Colbourne for his insightful feedback provided throughout his co-supervision of both MRJ's and TNL's PhDs. We thank Karl Burgess for his oversight

as Head of Metabolomics at Glasgow Polyomics, University of Glasgow where the GC-EI-HRMS measurements were performed. Thanks also to the Galaxy community for helpful discussions and contributions regarding the Galaxy tools and workflow development, and to Dominic Wilson and Andrew Edmonds from the Research Software Group, part of Advanced Research Computing at the University of Birmingham [60] for help with setting up the IT infrastructure for data storage, DMAdb and the Galaxy platform. Finally, we are particularly grateful to Clement Heude (no longer in the field) and the Biomolecular NMR Facility team at the University of Birmingham for the NMR measurements.

## 10 Author contributions

**TNL:** Formal analysis [lead], Software [lead], Visualisation [lead], Data curation [lead], Writing – original draft [lead], Writing – review & editing [equal], Conceptualization [equal], Investigation [supporting] and Methodology [supporting].

**MRJ:** Investigation [lead], Methodology [lead], Conceptualization [equal], Formal analysis [equal], Software [equal], Visualisation [equal], Data curation [equal], Writing – original draft [lead] and Writing – review & editing [equal].

**AJC:** Investigation [supporting], Methodology [supporting], and Writing – review & editing [supporting].

**ES:** Formal analysis [supporting], Visualisation [supporting], and Writing – review & editing [supporting].

**SW:** Investigation [supporting], Methodology [supporting], Formal analysis [supporting] and Writing – review & editing [supporting].

**RM:** Supervision [supporting] and Writing – review & editing [supporting].

**WD:** Supervision [supporting], Conceptualization [supporting], and Writing – review & editing [supporting].

**RJMW:** Supervision [equal]; Software [equal], Conceptualization [equal], Writing – original draft [equal], Writing – review & editing [equal]; Methodology [supporting], Formal analysis [supporting] and Data curation [supporting].

**MRV:** Supervision [lead]; Conceptualization [equal]; Writing – original draft [equal] and Writing – review & editing [equal].

## 11 Funding

This work was supported financially through two NERC CASE PhD studentships at the University of Birmingham with GigaScience (NE/L002493/1 – CENTA: Central England NERC Training Alliance; TNL) and Thermo Fisher Scientific (NE/J017442/1; MRJ). The work was also funded through the Wellcome Trust research grant “MetaboFlow” (202952/Z/16/Z; TNL, MRJ, RJMW, MRV) and funding from the European Union’s Horizon 2020 Research and Innovation programme under Grant Agreement No. 965406 “PrecisionTox” (TNL, MRJ, RJMW, ES, MRV). This output reflects only the authors’ views and the European Union

cannot be held responsible for any use that may be made of the information contained therein.

# 12 Data availability

All raw (U)HPLC-HRMS(/MS) and DI-HRMS(/MSn) data and selected annotations supporting the results of this article are available through MetaboLights (MTBLS2273). Additionally, the mass spectrometry files containing mass spectrometry gas-phase fragmentation spectra used for the GNPS analysis of the *D. magna* sample (and not equilibration, blank or reference standard samples) are also available through GNPS MassIVE repository (MSV000094957). The assay format was simplified for MetaboLights and MassIVE into four assays (apolar positive, apolar negative, polar positive and polar negative).

The Galaxy workflows, histories and details of each tool used in this project are available in the DMA Galaxy instance [31]. The Galaxy analysis performed used the following workflows:

- **W1**: The full (U)HPLC-HRMS(/MS), DI-HRMS(/MS$^n$) and LC fractionation workflow (described in **Figure 3**)
- **W2**: The (U)HPLC-HRMS(/MS) only workflow
- **W3**: The (U)HPLC-HRMS(/MS) workflow used for the metabolite reference standard analysis.

**Supplemental Table S1** provides the links for the relevant histories and workflow used for each assay.

The workflows are also made available on Workflowhub (W1 [61]; W2 [62] and W3 [63])

These above resources cover the full Galaxy workflow analysis; however, re-running all analyses, particularly outside of the provided Galaxy instances, would require additional setup due to both the high computational resource demands of this large dataset, as well as software updates to some of the underlying Galaxy tool since the analysis in this manuscript was performed. As such, we also include an example workflow for the most broadly reusable portion of the (U)HPLC-HRMS(/MS) workflow [64] that can be executed on other public instances (e.g. Workflow4Metabolomics [65]).

The GitHub repository “dmagna-dma-paper” (Section 5) additionally includes the consolidated annotation file spanning all assays that was used for figure generation and the derivation of summary information, along with details on accessing data from the associated Galaxy workflow histories. This repository also includes the numerical data used for all data analysis figures.

Additionally, the DMAdb web portal [43] can also be used to access all the raw and processed (U)HPLC-HRMS(/MS) and DI-HRMS(/MSn) data as well as viewing and searching the fragmentation spectra and metabolite annotations.

# 13 Competing interests

The authors declare no competing interests.

# 14 References


[1] T. M. Keane, L. Goodstadt, P. Danecek, M. A. White, K. Wong, B. Yalcin, *et al.*, Mouse genomic variation and its effect on phenotypes and gene regulation, *Nature*, vol. 477, no. 7364, pp. 289–294, Sep. 2011, doi: 10.1038/nature10413.

[2] I. Dunham, A. Kundaje, S. F. Aldred, P. J. Collins, C. a. Davis, F. Doyle, *et al.*, An integrated encyclopedia of DNA elements in the human genome, *Nature*, vol. 489, no. 7414, pp. 57–74, 2012, doi: 10.1038/nature11247.

[3] L. Hood and L. Rowen, The human genome project: big science transforms biology and medicine, *Genome Med*, vol. 5, no. 9, p. 79, 2013, doi: 10.1186/gm483.

[4] The 1000 Genomes Project Consortium, Corresponding authors, A. Auton, G. R. Abecasis, Steering committee, D. M. Altshuler, *et al.*, A global reference for human genetic variation, *Nature*, vol. 526, no. 7571, pp. 68–74, Oct. 2015, doi: 10.1038/nature15393.

[5] S. Moco and J. M. Buescher, Metabolomics: going deeper, going broader, going further, *Cell-Wide Identification of Metabolite-Protein Interactions*, pp. 155–178, 2022.

[6] E. Puris, Š. Kouřil, L. Najdekr, S. Auriola, S. Loppi, P. Korhonen, *et al.*, Metabolomic, Lipidomic and Proteomic Characterisation of Lipopolysaccharide-induced Inflammation Mouse Model, *Neuroscience*, vol. 496, pp. 165–178, Aug. 2022, doi: 10.1016/j.neuroscience.2022.05.030.

[7] A.-T. Ramabulana, D. Petras, N. E. Madala, and F. Tugizimana, Mass spectrometry DDA parameters and global coverage of the metabolome: Spectral molecular networks of momordica cardiospermoides plants, *Metabolomics*, vol. 19, no. 3, p. 18, Mar. 2023, doi: 10.1007/s11306-023-01981-4.

[8] D. S. Wishart, D. Tzur, C. Knox, R. Eisner, A. C. Guo, N. Young, *et al.*, HMDB: the Human Metabolome Database, *Nucleic acids research*, vol. 35, no. Database issue, pp. D521--6, Jan. 2007, doi: 10.1093/nar/gkl923.

[9] H. Horai, M. Arita, S. Kanaya, Y. Nihei, T. Ikeda, K. Suwa, *et al.*, MassBank: A public repository for sharing mass spectral data for life sciences, *J. Mass Spectrom.*, vol. 45, no. 7, pp. 703–714, 2010, doi: 10.1002/jms.1777.

[10] T. Kind, K.-H. Liu, D. Y. Lee, B. DeFelice, J. K. Meissen, and O. Fiehn, LipidBlast in silico tandem mass spectrometry database for lipid identification. *Nat Methods*, vol. 10, no. 8, pp. 755–758, Aug. 2013, doi: 10.1038/nmeth.2551.

[11] M. Wang, J. J. Carver, V. V. Phelan, L. M. Sanchez, N. Garg, Y. Peng, *et al.*, Sharing and community curation of mass spectrometry data with Global Natural Products Social Molecular Networking, *Nat Biotechnol*, vol. 34, no. 8, pp. 828–837, Aug. 2016, doi: 10.1038/nbt.3597.

[12] K. Haug, R. M. Salek, P. Conesa, J. Hastings, P. de Matos, M. Rijnbeek, *et al.*, MetaboLights--an open-access general-purpose repository for metabolomics studies and associated meta-data., *Nucleic acids research*, vol. 41, no. Database issue, pp. D781--6, Jan. 2013, doi: 10.1093/nar/gks1004.

[13] M. Sud, E. Fahy, D. Cotter, K. Azam, I. Vadivelu, C. Burant, *et al.*, Metabolomics Workbench: An international repository for metabolomics data and metadata, metabolite standards, protocols, tutorials and training, and analysis tools, *Nucleic Acids Res*, vol. 44, no. D1, p. gkv1042, 2015, doi: 10.1093/nar/gkv1042.

[14] M. R. Viant, I. J. Kurland, M. R. Jones, and W. B. Dunn, How close are we to complete annotation of metabolomes?, *Current Opinion in Chemical Biology*, vol. 36, pp. 64–69, Feb. 2017, doi: 10.1016/j.cbpa.2017.01.001.

[15] A. S. Edison, R. D. Hall, C. Junot, P. D. Karp, I. J. Kurland, R. Mistrik, *et al.*, The time is right to focus on model organism metabolomes, *Metabolites*, vol. 6, no. 1, p. 8, 2016, doi: 10.3390/metabo6010008.

[16] The Precision Toxicology initiative, *Toxicology Letters*, vol. 383, pp. 33–42, Jul. 2023, doi: 10.1016/j.toxlet.2023.05.004.

[17] A. Weismann, *Beiträge zur Naturgeschichte der Daphnoiden*, vol. 2. W. Engelmann, 1876.
[18] D. Ebert, *Introduction to the ecology, epidemiology, and evolution of parasitism in Daphnia*. National Center for Biotechnology Information (US), 2005.
[19] D. Ebert, Daphnia as a versatile model system in ecology and evolution, *EvoDevo*, vol. 13, no. 1, p. 16, Aug. 2022, doi: 10.1186/s13227-022-00199-0.
[20] W. Lampert, Daphnia: Model herbivore, predator and prey, *Polish Journal of Ecology*, vol. 54, no. 4, pp. 607–620, 2006.
[21] J. K. Colbourne, M. E. Pfrender, D. Gilbert, W. K. Thomas, A. Tucker, T. H. Oakley, *et al.*, The ecoresponsive genome of Daphnia pulex, *Science (New York, N.Y.)*, vol. 331, no. 6017, pp. 555–61, Feb. 2011, doi: 10.1126/science.1197761.
[22] E. Afgan, D. Baker, B. Batut, M. van den Beek, D. Bouvier, M. Čech, *et al.*, The Galaxy platform for accessible, reproducible and collaborative biomedical analyses: 2018 update, *Nucleic Acids Research*, vol. 46, no. W1, pp. W537–W544, Jul. 2018, doi: 10.1093/nar/gky379.
[23] F. Giacomoni, G. Le Corguille, M. Monsoor, M. Landi, P. Pericard, M. Petera, *et al.*, Workflow4Metabolomics: a collaborative research infrastructure for computational metabolomics, *Bioinformatics*, vol. 31, no. 9, pp. 1493–1495, May 2015, doi: 10.1093/bioinformatics/btu813.
[24] A. D. Southam, R. J. M. Weber, J. Engel, M. R. Jones, and M. R. Viant, A complete workflow for high-resolution spectral-stitching nanoelectrospray direct-infusion mass-spectrometry-based metabolomics and lipidomics, *Nature Protocols*, vol. 12, no. 2, pp. 310–328, Feb. 2017, doi: 10.1038/nprot.2016.156.
[25] Weber RJM, Zhou J., *DIMSpy: Python package for processing direct- infusion mass spectrometry-based metabolomics and lipidomics data*. (Apr. 2020). Zenodo. doi: 10.5281/zenodo.3764169.
[26] T. N. Lawson, R. J. M. Weber, M. R. Jones, A. J. Chetwynd, G. A. Rodriguez Blanco, R. Di Guida, *et al.*, msPurity: Automated evaluation of precursor ion purity for mass spectrometry based fragmentation in metabolomics, *Anal. Chem.*, vol. 89, no. 4, p. acs.analchem.6b04358, 2017, doi: 10.1021/acs.analchem.6b04358.
[27] C. Ruttkies, E. L. Schymanski, S. Wolf, J. Hollender, and S. Neumann, 'MetFrag relaunched: Incorporating strategies beyond in silico fragmentation', *J Cheminform*, vol. 8, no. 1, pp. 1–16, 2016, doi: 10.1186/s13321-016-0115-9.
[28] C. Ruttkies, S. Neumann, and S. Posch, Improving MetFrag with statistical learning of fragment annotations, *BMC Bioinformatics*, vol. 20, no. 1, p. 376, Dec. 2019, doi: 10.1186/s12859-019-2954-7.
[29] S. Wolf, S. Schmidt, M. Müller-Hannemann, and S. Neumann, In silico fragmentation for computer assisted identification of metabolite mass spectra, *BMC Bioinformatics*, vol. 11, no. 1, p. 148, 2010, doi: 10.1186/1471-2105-11-148.
[30] K. Dührkop, M. Fleischauer, M. Ludwig, A. A. Aksenov, A. V. Melnik, M. Meusel, *et al.*, SIRIUS 4: a rapid tool for turning tandem mass spectra into metabolite structure information, *Nat Methods*, vol. 16, no. 4, pp. 299–302, Apr. 2019, doi: 10.1038/s41592-019-0344-8.
[31] Galaxy instance for the Deep metabolome annotation of (*D. magna*). https://dma.galaxy.bham.ac.uk/. Accessed: 17 Apr 2026.
[32] HighChem LLC. mzCloud: Advanced mass spectral database. https://www.mzcloud.org/. Accessed 12 Feb 2026.
[33] Global Natural Products Social Molecular Networking (GNPS). GNPS: Global natural products social molecular networking. https://gnps.ucsd.edu. Accessed 12 Feb 2026.
[34] H. Mohimani, A. Gurevich, A. Mikheenko, N. Garg, L.-F. Nothias, A. Ninomiya, *et al.*, Dereplication of peptidic natural products through database search of mass spectra, *Nature Chemical Biology*, vol. 13, no. 1, pp. 30–37, Jan. 2017, doi: 10.1038/nchembio.2219.
[35] J. Wandy, Y. Zhu, J. J. J. van der Hooft, R. Daly, M. P. Barrett, and S. Rogers, Ms2lda.org: web-based topic modelling for substructure discovery in mass

spectrometry, *Bioinformatics*, vol. 34, no. 2, pp. 317–318, Jan. 2018, doi: 10.1093/bioinformatics/btx582.

[36] Y. Djoumbou Feunang, R. Eisner, C. Knox, L. Chepelev, J. Hastings, G. Owen, *et al.*, ClassyFire: automated chemical classification with a comprehensive, computable taxonomy, *J Cheminform*, vol. 8, no. 1, p. 61, Dec. 2016, doi: 10.1186/s13321-016-0174-y.

[37] M. Kanehisa and S. Goto, KEGG: Kyoto Encyclopedia of Genes and Genomes, *Nucleic Acids Research*, vol. Vol. 28, no. 1, pp. 27–30, Jan. 2000.

[38] M. Kanehisa, Toward understanding the origin and evolution of cellular organisms, *Protein Science*, vol. 28, no. 11, pp. 1947–1951, Nov. 2019, doi: 10.1002/pro.3715.

[39] M. Kanehisa, M. Furumichi, Y. Sato, M. Kawashima, and M. Ishiguro-Watanabe, KEGG for taxonomy-based analysis of pathways and genomes, *Nucleic Acids Research*, vol. 51, no. D1, pp. D587–D592, Jan. 2023, doi: 10.1093/nar/gkac963.

[40] J. Hastings, P. de Matos, A. Dekker, M. Ennis, B. Harsha, N. Kale, *et al.*, The ChEBI reference database and ontology for biologically relevant chemistry: enhancements for 2013., *Nucleic acids research*, vol. 41, no. Database issue, pp. D456--63, Jan. 2013, doi: 10.1093/nar/gks1146.

[41] E. Sostare, T. N. Lawson, L. R. Saunders, J. K. Colbourne, R. J. M. Weber, T. Sobanski, *et al.*, Knowledge-Driven Approaches to Create the MTox700+ Metabolite Panel for Predicting Toxicity, *Toxicological Sciences*, vol. 186, no. 2, pp. 208–220, Mar. 2022, doi: 10.1093/toxsci/kfac007.

[42] Biobyte Solutions GmbH, PhyloT: Phylogenetic tree generator. https://phylot.biobyte.de/index.cgi. Accessed 12 Feb 2026.

[43] DMAdb: Deep metabolome annotation database (*D. magna*). https://dmadb.bham.ac.uk/. Accessed 17 Apr 2026.

[44] DMAdb docs: Deep metabolome annotation database (*D. magna*) – documentation and Django package details. https://dmadb.readthedocs.io/en/latest/getting-started.html. Accessed: 17 Apr 2026.

[45] S. Böcker and F. Rasche, Towards de novo identification of metabolites by analyzing tandem mass spectra, *Bioinformatics*, vol. 24, no. 16, pp. 49–55, 2008, doi: 10.1093/bioinformatics/btn270.

[46] K. Peters, S. Herman, P. E. Khoonsari, J. Burman, S. Neumann, and K. Kultima, Metabolic drift in the aging nervous system is reflected in human cerebrospinal fluid, *Sci Rep*, vol. 11, no. 1, p. 18822, Sep. 2021, doi: 10.1038/s41598-021-97491-1.

[47] L. Colas, A.-L. Royer, J. Massias, A. Raux, M. Chesneau, C. Kerleau, *et al.*, Urinary metabolomic profiling from spontaneous tolerant kidney transplanted recipients shows enrichment in tryptophan-derived metabolites, *eBioMedicine*, vol. 77, p. 103844, Mar. 2022, doi: 10.1016/j.ebiom.2022.103844.

[48] E. Eysseric, C. Gagnon, and P. A. Segura,‘Identifying congeners and transformation products of organic contaminants within complex chemical mixtures in impacted surface waters with a top-down non-targeted screening workflow, *Science of The Total Environment*, vol. 822, p. 153540, May 2022, doi: 10.1016/j.scitotenv.2022.153540.

[49] J. Rainer, A. Vicini, L. Salzer, J. Stanstrup, J. M. Badia, S. Neumann, *et al.*, A Modular and Expandable Ecosystem for Metabolomics Data Annotation in R, *Metabolites*, vol. 12, no. 2, p. 173, Feb. 2022, doi: 10.3390/metabo12020173.

[50] R Core Team, R: A language and environment for statistical computing, R Foundation for Statistical Computing, Vienna, Austria, manual, https://www.R-project.org/. Accessed 17 Apr 2026.

[51] H. Wickham, *ggplot2: Elegant graphics for data analysis*. Springer-Verlag New York. https://ggplot2.tidyverse.org. Accessed 17 Apr 2026.

[52] J. R. Conway, A. Lex, and N. Gehlenborg, UpSetR: An R package for the visualization of intersecting sets and their properties, *Bioinformatics*, vol. 33, no. 18, pp. 2938–2940, 2017, doi: 10.1093/bioinformatics/btx364.

[53] G. Yu, Using ggtree to visualize data on tree-like structures, *Current protocols in bioinformatics*, vol. 69, no. 1, p. e96, 2020.

[54] E. Paradis and K. Schliep, ape 5.0: an environment for modern phylogenetics and evolutionary analyses in R, *Bioinformatics*, vol. 35, no. 3, pp. 526–528, Feb. 2019, doi: 10.1093/bioinformatics/bty633.
[55] S. Xu, Q. Wang, S. Wen, J. Li, N. He, M. Li, *et al.*, aplot: Simplifying the creation of complex graphs to visualize associations across diverse data types, *The Innovation*, p. 100958, May 2025, doi: 10.1016/j.xinn.2025.100958.
[56] Y. Cao, A. Charisi, L.-C. Cheng, T. Jiang, and T. Girke, ChemmineR: a compound mining framework for R, *Bioinformatics*, vol. 24, no. 15, pp. 1733–1734, Aug. 2008, doi: 10.1093/bioinformatics/btn307.
[57] S. Kim, J. Chen, T. Cheng, A. Gindulyte, J. He, S. He, *et al.*, PubChem 2025 update, *Nucleic Acids Research*, vol. 53, no. D1, pp. D1516–D1525, Jan. 2025, doi: 10.1093/nar/gkae1059.
[58] C. A. Smith, E. J. Want, G. O. Maille, R. Abagyan, and G. Siuzdak, XCMS : Processing Mass Spectrometry Data for Metabolite Profiling Using Nonlinear Peak Alignment , Matching , and Identification, *Anal. Chem.*, vol. 78, no. 3, pp. 779–787, 2006, doi: 10.1021/ac051437y.
[59] C. Kuhl, R. Tautenhahn, C. Böttcher, T. R. R. Larson, S. Neumann, C. Bo, *et al.*, CAMERA: An integrated strategy for compound spectra extraction and annotation of liquid chromatography/mass spectrometry data sets, *Anal. Chem.*, vol. 84, no. 1, pp. 283–289, Jan. 2012, doi: 10.1021/ac202450g.
[60] University of Birmingham, BEAR software: Birmingham environment for academic research. https://www.birmingham.ac.uk/bear-software. Accessed: 12 Feb 2026.
[61] DMA (*D. magna*) workflow W1: (U)HPLC-HRMS(/MS), DI-HRMS(/MSn), and LC fractionation workflow. WorkflowHub. https://workflowhub.eu/workflows/2084. Accessed 17 Apr 2026.
[62] DMA (D. magna) workflow W2: (U)HPLC-HRMS(/MS) only workflow. WorkflowHub. https://workflowhub.eu/workflows/2085. Accessed 17 Apr 2026.
[63] DMA (D. magna) workflow W3: (U)HPLC-HRMS(/MS) workflow used for metabolite reference standard analysis. WorkflowHub. https://workflowhub.eu/workflows/2086. Accessed 17 Apr 2026.
[64] DMA (D. magna) example workflow for (U)HPLC-HRMS(/MS). WorkflowHub. https://workflowhub.eu/workflows/2083. Accessed 17 Apr 2026.
[65] Workflow4Metabolomics. Workflow4Metabolomics Galaxy instance. https://workflow4metabolomics.usegalaxy.fr/. Accessed 17 Apr 2026.

# 15 Author notes

Martin R. Jones and Thomas N. Lawson contributed equally to this article.

Publisher’s notes.

© The Author(s) 2026. Published by Oxford University Press GigaScience.

This is an Open Access article distributed under the terms of the Creative Commons Attribution License (https://creativecommons.org/licenses/by/4.0/), which permits unrestricted reuse, distribution, and reproduction in any medium, provided the original work is properly cited.

Click here to access/download;Figure;fig1_28022026.pdf

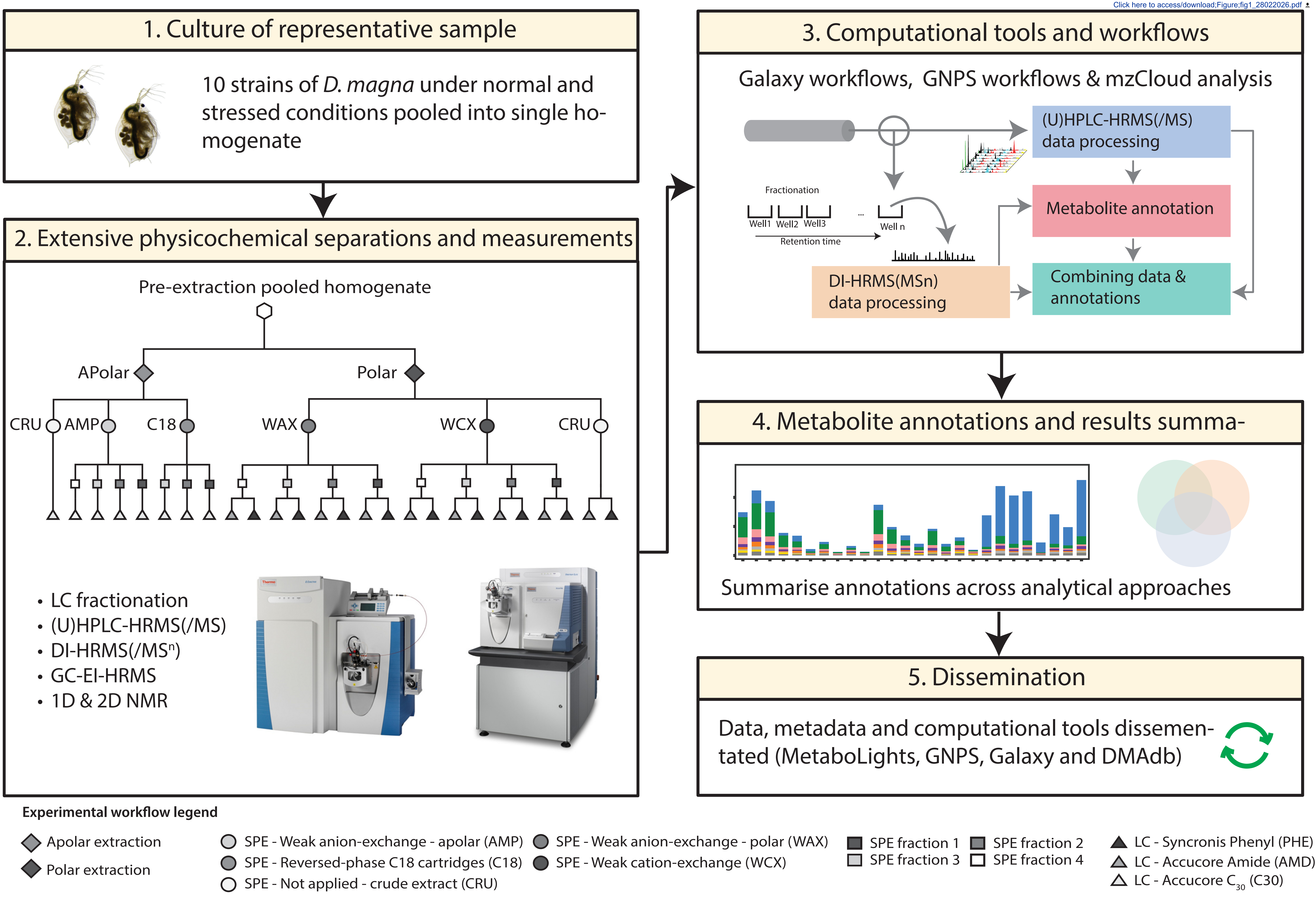

Click here to access/download;Figure;fig2_28022026.pdf

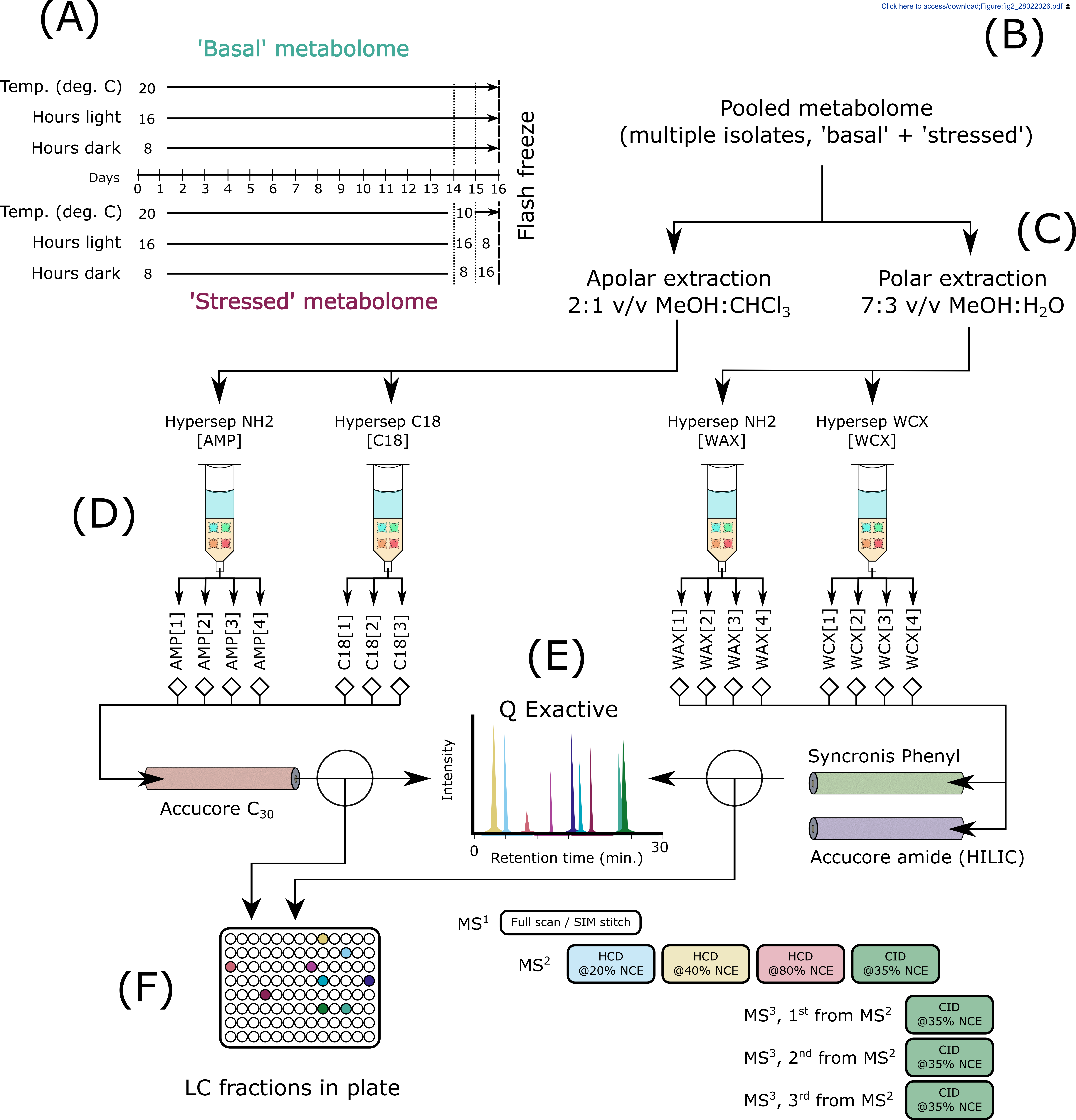

Click here to access/download;Figure;fig3_28022026.pdf

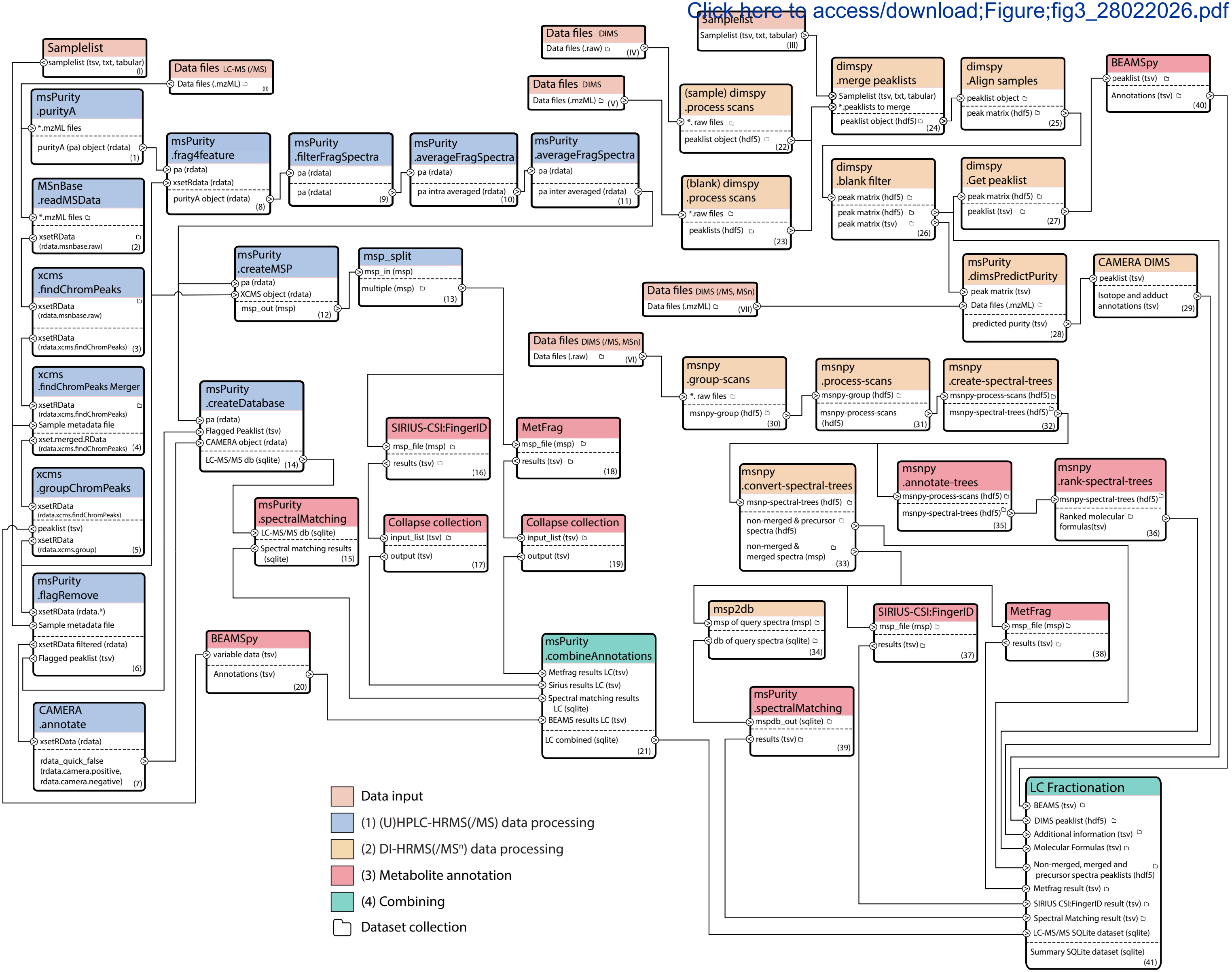

Click here to access/download;Figure;fig4_28022026.pdf

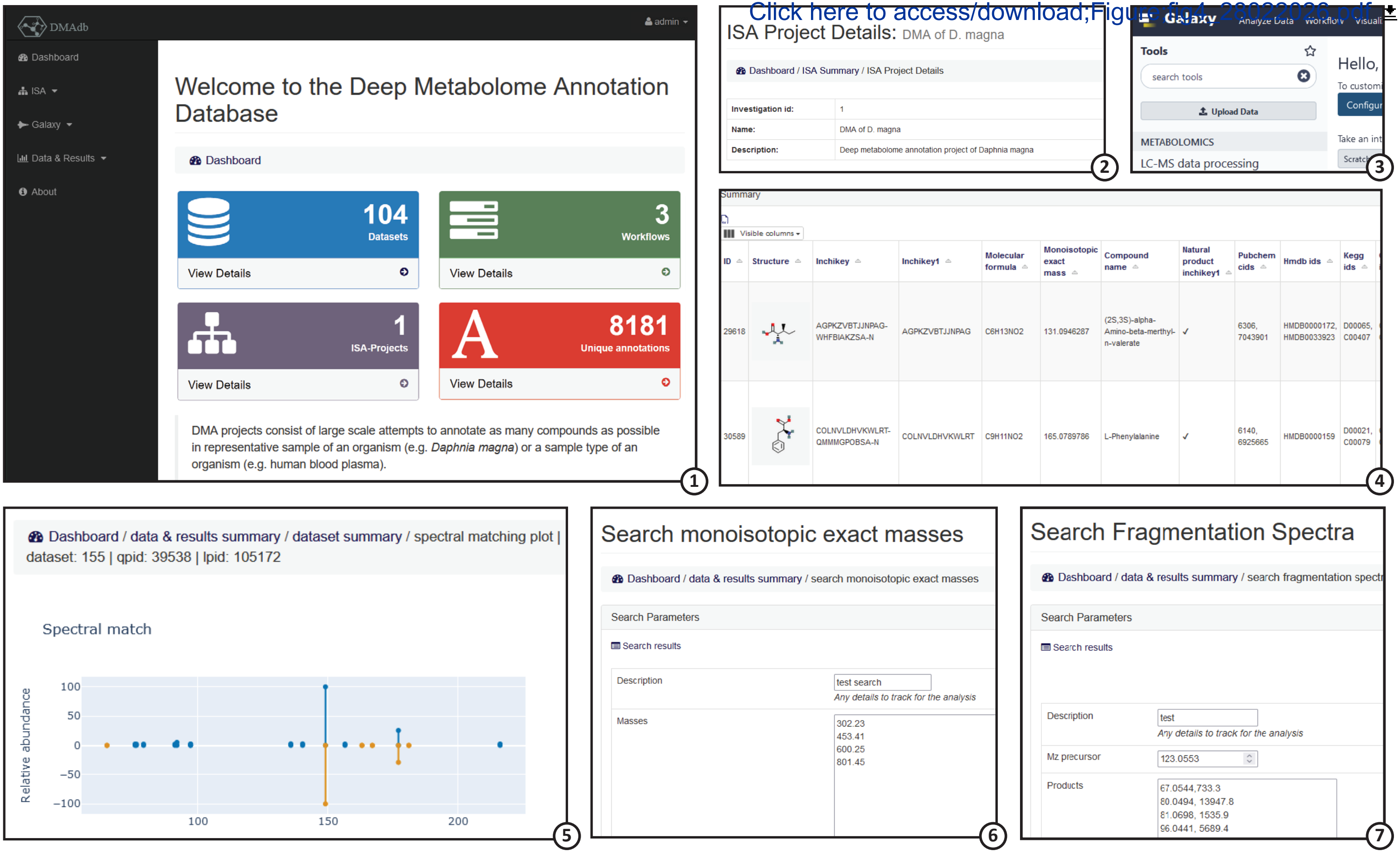

Click here to access/download;Figure;fig5_28022026.png

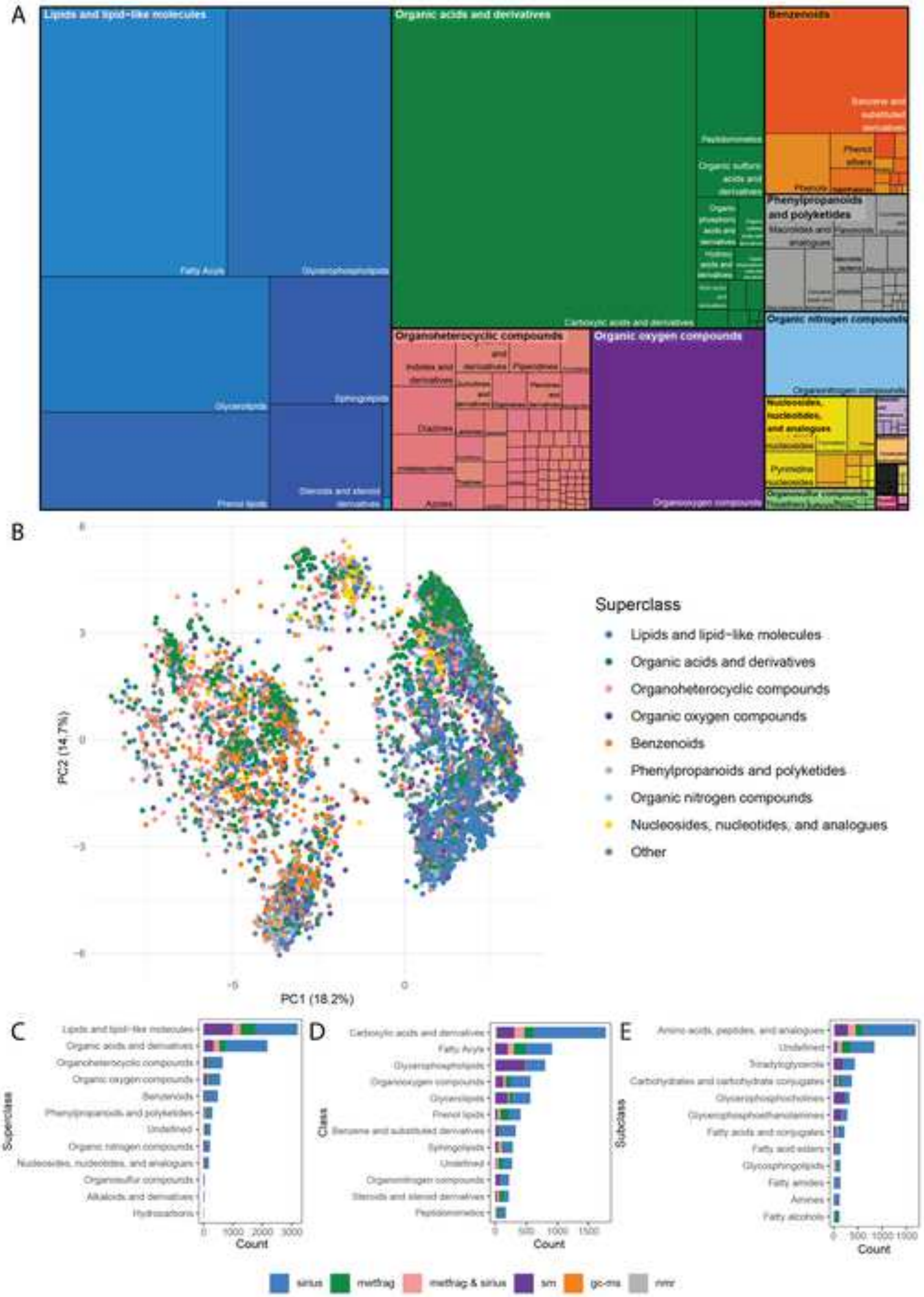

Click here to access/download;Figure;fig6_28022026.png

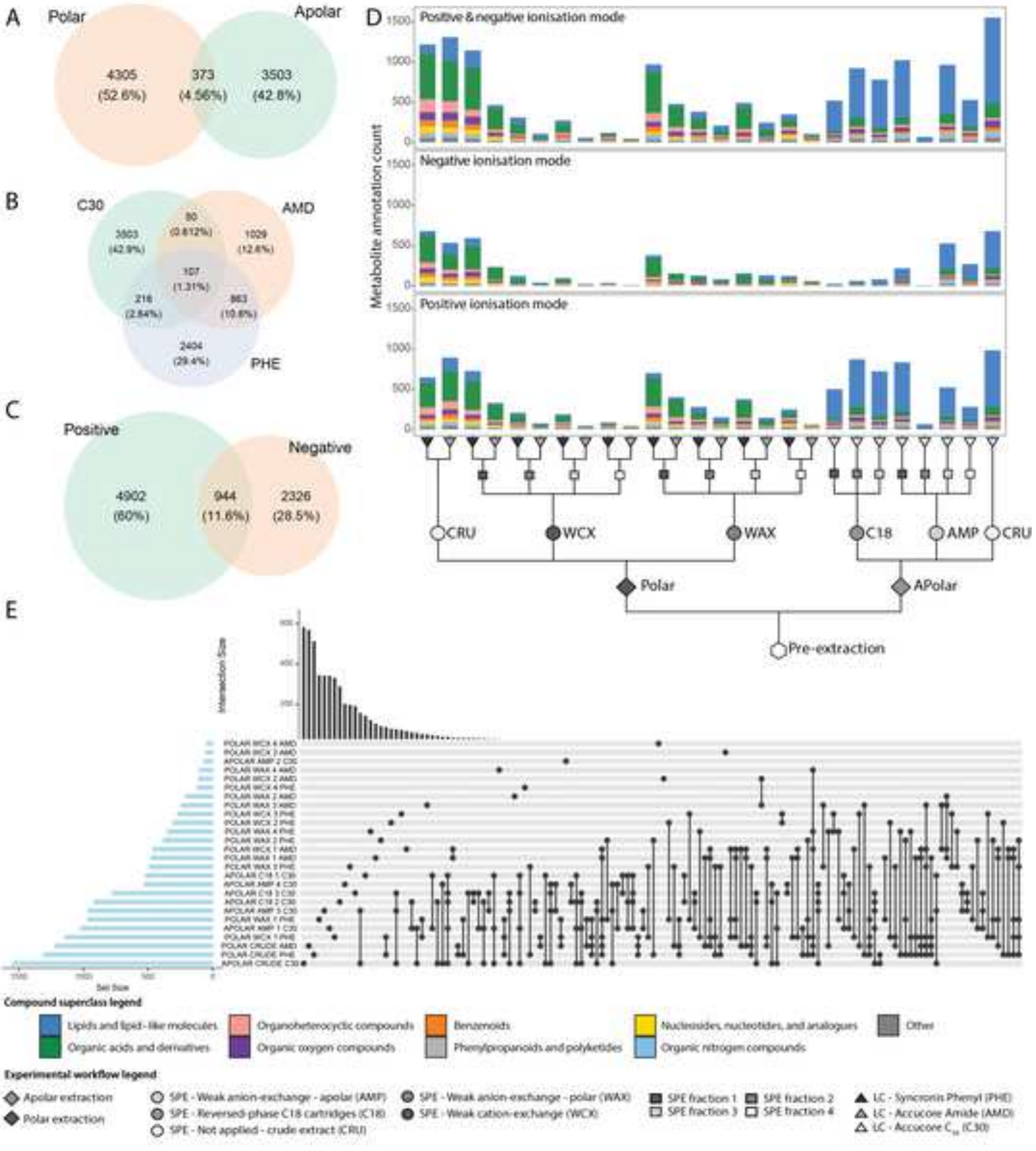

Click here to access/download;Figure;fig7_28022026.png

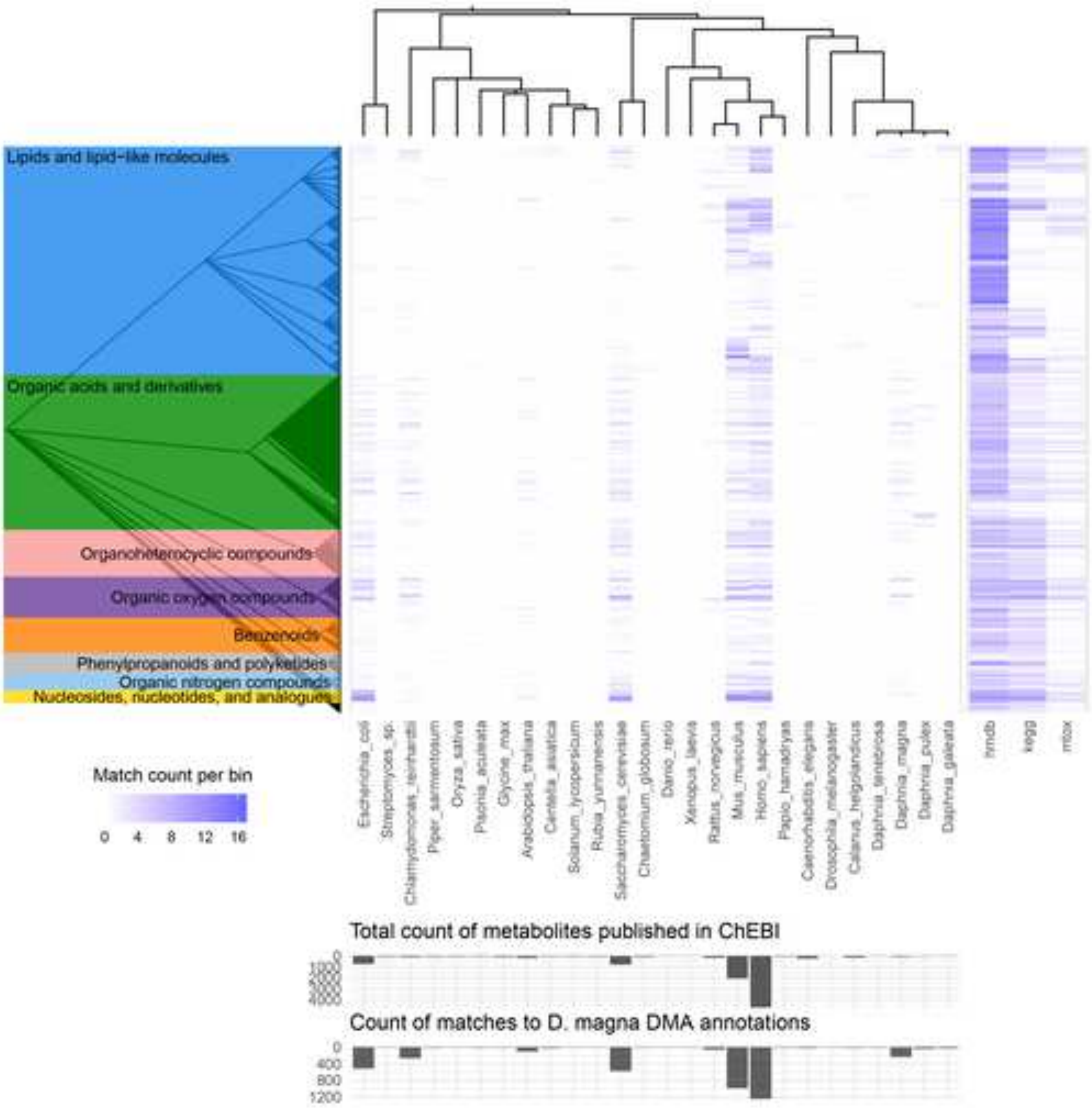

Click here to access/download;Figure;fig8_28022026.png

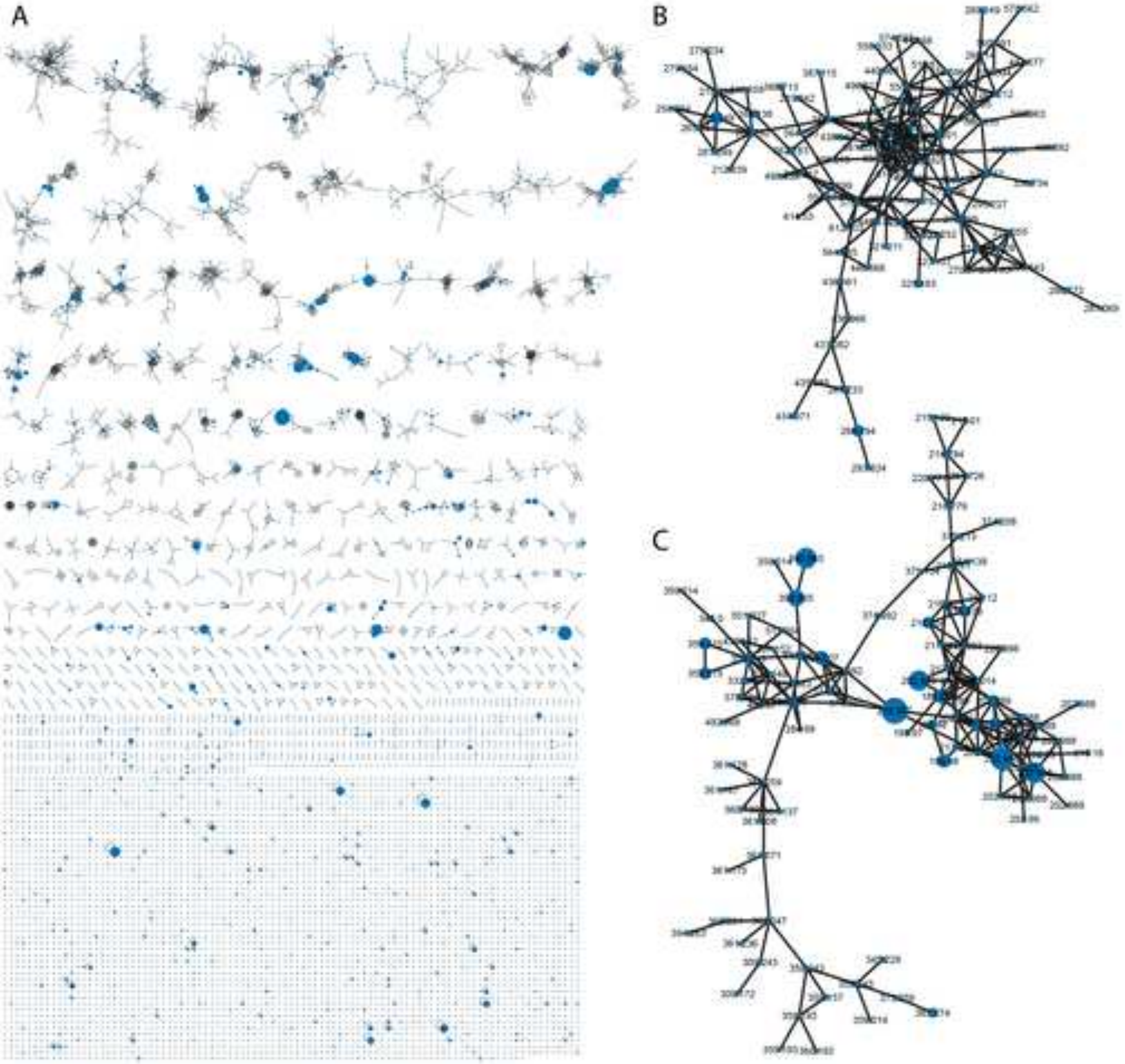

Click here to access/download
**Supplementary Material**
DMA D. magna - supp tables revised 28022026.xlsx

Click here to access/download
**Supplementary Material**
DMA D. magna - supp info ed revised 01052026.docx

GIGA-D-25-00453
Dear Yannan,

We thank you and the reviewers for the careful review of our manuscript *"Experimental and computational approaches for deep metabolome annotation with application to the ecotoxicological model organism Daphnia magna"* (GIGA-D-25-00453). We appreciate the constructive and insightful comments, all of which have helped us to improve the clarity and quality of our work and manuscript.

We have revised the manuscript accordingly. Below, we provide a point-by-point response to all reviewer and editorial comments. For each point, we describe the revision made or provide clarification where appropriate. All changes in the manuscript have been clearly marked by track changes.

We are confident that the changes made have resolved all concerns and improved the manuscript accordingly.

We also note, following further consideration of the author's contributions to the manuscript preparation, we have decided to list Thomas N. Lawson as the first author. Thomas N. Lawson and Martin R. Jones should still be regarded as joint first authors.

Please let us know if there are any formal steps required to implement this change.

In addition, two instrument images included in the workflow figure (Figure 1) are sourced from Wikimedia Commons under the Creative Commons Attribution–ShareAlike 3.0 Unported (CC BY-SA 3.0) licence, with appropriate attribution provided in the figure legend. Please let us know if any further clarification is required regarding third-party material.

Best regards,

Assistant Professor Ralf Weber & Professor Mark Viant
(Joint corresponding authors)

**Point-by-Point Responses**

**Editor**

Register new software tools in bio.tools and SciCrunch (to obtain RRIDs), and register workflows in WorkflowHub.

- Where applicable and feasible, we have registered primary, reusable software tools in bio.tools and included the corresponding RRIDs in the revised manuscript, with associated DOIs cited in the *Methods* and *Data Availability* sections.
- The complete set of Galaxy workflows has been uploaded to WorkflowHub (and available on dma.galaxy.bham.ac.uk, or are also available from the following repository https://github.com/computational-metabolomics/dmagna-dma-galaxy-workflows);
- Please note that execution of these workflows is currently limited to users with direct access to dma.galaxy.bham.ac.uk. Installation on other Galaxy instances is possible but requires minor configuration for optimal performance. For example, local paths to specific databases may need to be defined to avoid excessive web API calls. In addition, a small number of tools used for MS-fragmentation processing of LC-fractionation data in one workflow are bespoke and are not available through the Galaxy ToolShed; however, they can be manually installed from the corresponding GitHub repository (see Table 1 in manuscript). The version of SIRIUS CSI:FingerID used within the Galaxy workflow is no longer publicly accessible, as it relied on an API endpoint that is no longer supported for CSI:FingerID v4. Access now requires explicit approval from the developers. Limitations have been clarified in the main manuscript.
- We have also deposited an additional workflow, representing the most broadly reusable portion of the LC-MS/MS-based annotation workflow, which can be executed on the public Workflow4metabolomics Galaxy instance (https://workflow4metabolomics.usegalaxy.fr, free to anyone to sign up for an account).

All URLs must be converted into references and removed from the main text (except in the Abstract and Code Availability).

- All web links have been moved into the reference list and assigned numbered citations. The reference order has been updated accordingly.
- Multiple URLs remain within the “Code Availability” section, including the DMAdb prototype resource (dmadb.bham.ac.uk), in accordance with the journal’s guidance that URLs may appear in this section. As this resource is not a primary focus of the manuscript, it has not been included in the Abstract. Although it would most naturally be described within the Methods, it has been placed in “Code Availability” to ensure compliance with the journal instructions.

Ensure all author ORCIDs are updated.

- All authors have ORCIDs registered within the submission portal. Please can we confirm with the editor that all authors will receive a submission notice where they can check their ORCID details.

## Reviewer 1

We thank the Reviewer 1 for their detailed and constructive feedback, particularly regarding data accessibility, pathway analysis, and website functionality, which has helped us significantly improve the manuscript.

Firstly, during the review process we identified that the data analysis could be improved by incorporating additional checks to ensure that annotated features were not derived from extract blank samples. As a result, the final annotations and corresponding figures presented in the manuscript have been revised, with 4.6% of annotations removed. Method details in the Supplemental Information have been updated to reflect this

As a resource, it will be more useful if feature annotation from each method is made accessible. That is, when another lab generates data using a similar method, the m/z and retention time can be used for their annotation. As is, the supplemental tables and the website only provide compound centered results, which are not clearly linked to experimental data. Annotation per experiment method will also help others assess annotation confidence and how to best combine annotations, which are, as the authors rightfully acknowledged, not fully evaluated. Concentration levels, even just signal intensity as proxy, would be very useful to others. The project website hosts a searchable table, but direct dataset downloads should be available and important.

- Whilst we encourage any full re-annotation of the data to be performed directly from the raw data, we have now clarified how various feature and annotation details can be accessed.
- The full list of annotation details for every analytical assay and computational approach is available via the GitHub repository (https://github.com/computational-metabolomics/dmagna-dma-paper; see zip file).
- For each Galaxy workflow history, the complete feature lists and annotations can be explored in detail. A practical starting point for examining LC-MS features (including intensity values, which may serve as a limited proxy for concentration) is the XCMS RData objects and associated peaklists. URLs to each Galaxy history are provided in Supplementary Table 1. Additionally, we have added the URLs to the corresponding XCMS RData files and peaklists for each history in the above GitHub repository, along with an R script demonstrating how XCMS dataset features can be downloaded from Galaxy and inspected alongside the full list of annotations (see example_feature_check.R). This detail has been added to the “Code Availability” section.
- Additionally, for every assay, the Galaxy workflow history contains an SQLite database generated by either the “*LC-MS/MS fractionation processor*” or “*msPurity.combineAnnotations*”. These databases include references to fragmentation spectra and the former captures the full details of the LC-MS/MS fractionation and DIMSn analyses. Admittedly, these resources are relatively complex and require

additional setup to maximise their utility for the community; however, we still make them accessible within the Galaxy histories for those interested.
- We anticipate that these revisions will enhance the usability of the resources and facilitate the evaluation of the annotations

It will also be important to archive the high-quality features without annotation. As the authors have invested so much in generating the data, listing the unknown features will make it possible for them to become known in the future.

- As described above, for every assay method, we have clarified how to download the information for all features, not just those annotated.
- We note that this work was primarily focused on metabolite annotations. However, in future work, particularly as additional data are collected from other organisms and sample types, we aim to explore fragmentation data and high-quality yet unannotated features in greater detail using mass spectrometry fragmentation networks, which showed initial promise in the results presented here. To facilitate this, the data have been uploaded to GNPS (as described in the Data Availability section), where they are freely accessible for further exploration.

It is understood that readers can reproduce such features and annotation by running the various Galaxy histories. But this reproducibility requires a high level of commitment to access a Galaxy server and look up many granular details, where the underlying software functions have many design limitations. This makes reproducibility for the sake of reproducibility, not necessarily for scientific progress. We appreciate the efforts from the authors, but wish that the paper includes some discussion of the older software tools versus newer options.

- We agree that reproducing a complex, multi-step workflow within Galaxy can require substantial effort, particularly for users unfamiliar with the platform or with the specific configuration requirements of this study. Our intention in sharing the full Galaxy histories and workflows is to provide transparency and enable methodological inspection and reuse where appropriate, rather than to imply that full re-execution will always be practical or necessary for scientific progress.
- To improve accessibility and practical reuse, we have provided a simplified version of the workflow focused on LC-MS/MS analysis via the Workflow4Metabolomics Galaxy instance, where it can be readily applied by the metabolomics community.
- We also note that several of the Galaxy tools developed for this work were made publicly available prior to publication and have already been applied in metabolomics studies, see references in Conclusion section.
- Additionally, in response to the reviewer's suggestion, we have expanded the Conclusion section to acknowledge limitations associated with Galaxy-based reproducibility for large and specialised workflows and discuss limitations of specific software versions used and the potential advantages of newer or updated tools.

The IPA pathway analysis appears to be incorrect, because Fisher's exact test is for overrepresentation of a category/pathway. This work concerns how the annotated metabolites

map to metabolic pathways, not involving an overrepresentation test. For this, one can use a generic pathway definition from an open resource. IPA is not needed but adds complications.

- We’ve removed Fisher's exact test results and replaced them with a summary of how the annotated metabolites map to pathways within IPA software. We appreciate the point that this type of mapping can also be carried out using open-source pathway resources, and we agree that these provide useful alternatives. We nonetheless consider IPA to be an appropriate (and widely used) choice for pathway analysis and so have still included its usage.
- The IPA pathway results paragraph has been updated slightly to ensure the final sentence clearly reflects the intended interpretation.

The website, https://dmadb.bham.ac.uk/, is nice. But we have spotted a few glitches. For some functions, errors returned that user privilege is not granted. Filtering worked for some values, not others. E.g. Error 502 Gateway Error when 390-399 was applied to mono mass filtering.

- We thank the reviewer for their positive comments on DMAdb.
- We have revised the manuscript to clarify that DMAdb, including its Django-based applications, was primarily developed as a platform to support the local organisation, analysis, and public dissemination of metabolite annotations, associated datasets, and related experimental metadata. While the current implementation represents a focused and evolving resource, it provides a foundational framework for the continued expansion, refinement, and interactive exploration of future DMA and related metabolomics datasets.
- In line with this positioning, we have expanded the description of DMAdb in the Methods section to better emphasise its role and functionality. We have also revised the Results section to ensure a more accurate and balanced representation of the overall resource.
- We further clarify that users who log in via a Google account can access additional functionality, including batch mass searches and MS/MS-based querying.
- Finally, we have addressed the technical issues identified by the reviewer, including filtering inconsistencies and gateway errors, to ensure stable and reliable browsing and basic exploration of the data and annotations.

It'd be nice to mention in the main text of how the NMR data overlap with MS data.

We have expanded the corresponding section in the main text to more clearly describe the overlap between the NMR and MS datasets. However, we have intentionally kept this discussion concise and did not dwell extensively on detailed comparisons between NMR, GC–MS, and LC–MS, as the analytical dataset is strongly weighted toward LC–MS measurements. Consequently, the comparative depth that could be meaningfully provided across platforms is limited.

Under section 3.4, reference to Figure 7 should be Figure 6.

- Corrected

The stress conditions in this paper were rather limited. It'd be good to add to discussions how this may be broadened in the future. How to link to genomics would be worthy discussions too.

- We have added text to the Conclusion section to acknowledge this limitation and to highlight the potential for novel *D. magna* metabolome discoveries through deep metabolome annotation of a wider variety of *D. magna* isolates, both in terms of geographic origins (and related genetic variation) and their environmental exposure conditions.

**Reviewer 2**

We are grateful to Reviewer 2 for their thoughtful and constructive comments, which provided valuable biological context and helped improve the clarity and scope of the study.

Firstly, during the revision process we identified that the data analysis could be further improved by implementing additional checks to ensure that annotated metabolic features were not derived from blank extract samples. As a result, the final set of annotations and the corresponding figures in the manuscript have been updated, with 4.6% of annotations removed following this refinement. Method details in the Supplemental Information have been updated to reflect this.

While this comment is too late now (the study is already done), it would have been good to use a proper method for gut evacuation of the animals. Starving is known not to do this. Daphnia digest slowly. For gut evacuation, feeding the Daphnia with dextran beats, replaces the entire gut content with these beats. This would reduce the problem with metabolites potentially derived from the food (Chlorella algae) and microbiota (fungi and bacteria).

- We agree that starvation does not fully evacuate the gut and appreciate the reviewer's suggestion. We have incorporated this point into our interpretation of the origin of the metabolite annotations in Section 3.2.

Where the Daphnia checked for parasites before the 10 genotypes were used? Many D. magna clone harbour parasites in their gut epithelium.

- We confirm that the Daphnia were examined for visible gut-parasite infections before selecting the ten genotypes. This information has now been explicitly added to the Methods in the Supplemental Information.

I appreciate that the authors tried to diversify across D. magna genotypes, but the sample of 10 D. magna clones used seems a bit arbitrary. Most strain collections around the world, use central European D. magna clones, e.g. the NIES clone from Japan is from Europe (via the US).

But I think this is ok, as most work on this model species is done in Europe anyway. Future studies may include confirmed isolates from the East Asian, South African and North American D. magna lineage.

- We have added text to the conclusion to acknowledge this.

The references cited need some curation:
Book chapter are often incorrectly formatted and incomplete.
Several references miss the year of publication (e.g. ref. 17, 18, 20, 32, 38, 46), other miss the doi (e.g ref. 6, most book chapters).
Incorrect formatting in ref. 16.

- We thank the reviewer for carefully noting the issues in the reference list. We had initially used the “GigaScience” Zotero citation style, which appears to contain formatting inconsistencies. We have now switched to an alternative citation format and carefully checked and corrected all references. We also note that some older book references do not have DOIs available.

Section 2.1.3, third line Type: "polar polar"

- Corrected.

# Experimental and computational approaches for deep metabolome annotation with application to the ecotoxicological model organism *Daphnia magna*

Thomas N. Lawson[1,2,†], Martin R. Jones[1,†], ~~Thomas N. Lawson[1,2,†],~~ Andrew J. Chetwynd[1,3,α], Elena Sostare[2], Stefan Weidt[5], Robert Mistrik[4,δ], Warwick B. Dunn[1,3,§], Ralf J. M. Weber[1,3,*], Mark R. Viant[1,2,3,*]

[1]School of Biosciences, University of Birmingham, Edgbaston, Birmingham, B15 2TT, UK
[2]Michabo Health Science Limited, Union House, 111 New Union Street, Coventry, CV1 2NT, UK
[3]Phenome Centre Birmingham, University of Birmingham, Edgbaston, Birmingham, B15 2TT, UK
[4]HighChem, Mlynské nivy 5, 821 09 Bratislava, Slovakia
[5]Glasgow Polyomics, University of Glasgow, University Avenue, Glasgow, G12 8QQ, UK

[†]Joint first authors
Present addresses: [α]Centre for Proteome Research, and [§]Centre for Metabolomics Research, Department of Biochemistry, Cell and Systems Biology, Institute of Systems, Molecular and Integrative Biology, University of Liverpool, Liverpool, L69 7ZB, UK; [δ]Bitmoderna, Leskova 11, 81104 Bratislava, Slovakia.

*Correspondence address: School of Biosciences, University of Birmingham, Edgbaston, Birmingham, B15 2TT, UK; E-mail: m.viant@bham.ac.uk; E-Mail: r.j.weber@bham.ac.uk

## Abstract

**Background:** Comprehensively characterising the metabolomes of model organisms with high coverage and confidence is a critical step towards interpreting the metabolic basis of human and environmental health, yet there are formidable challenges involved in annotating metabolomes. A wide range of genotypes and phenotypes should be sampled with multiple complementary analytical approaches to cover the large and dynamic biochemical space they exhibit. In addition, multiple computational tools and approaches are required to annotate the metabolites from raw analytical data.

**Results:** To address this, we developed the Deep Metabolome Annotation (DMA) workflow. Applied to the ecological sentinel species, *Daphnia magna,* one pooled sample comprising ten distinct strainsgenotypes exposed to both normal and stressed environmental conditions was extracted and systematically physicochemically separated via solid-phase extraction, liquid- and gas-chromatography prior to extensive multiple-stage mass spectrometric fragmentation, generating more than 8,000 raw data files, and supplemented by nuclear magnetic resonance spectroscopy. An extensive Galaxy-based computational approach was built to analyse these data, comprising over 30 tools. The overall DMA efforts resulted in 8,5778,181 annotated polar metabolites and lipids in *D. magna,* with the raw and processed data, tools and annotations disseminated freely via public data repositories and a custom web-based interface to maximise reusability and facilitate transferability.

**Conclusions:** The DMA workflow has generated one of the largest metabolome annotation datasets for any non-human model organism and provides the first in-depth characterisation of the *D. magna* metabolome – providing both a resource and a valuable catalyst for future deep metabolome annotation studies of other model organisms.

# Table of contents

1 Introduction 5
2 Methods 8
2.1 Deep metabolome annotation experimental workflow 8
2.1.1 Overview 8
2.1.2 D. magna culturing and sample preparation 8
2.1.3 Metabolite extraction from D. magna pooled sample 8
2.1.4 Solid phase extraction-based fractionation of metabolite extracts 9
2.1.5 (U)HPLC-HRMS(/MS), DI-HRMS(/MS$^n$) and LC fractionation 9
2.1.6 (U)HPLC-HRMS(/MS) method optimisation 10
2.1.7 GC-EI-HRMS 10
2.1.8 1D & 2D NMR spectroscopy 10
2.2 Computational tools and workflows for data processing, metabolite annotation and data analysis 12
2.2.1 Overview 12
2.2.2 Galaxy workflow details 12
2.2.3 mzCloud library search 14
2.2.4 GNPS library search 14
2.2.5 GNPS molecular network analysis 14
2.2.6 Combining and summarising all annotations 14
2.2.7 Comparison to other metabolite databases 15
2.3 Assessment of the computational and experimental DMA workflow with metabolite reference standards 15
2.4 Deep Metabolome Annotation Database and Web Portal (DMAdb) 15
3 Results and discussion 17
3.1 (U)HPLC-HRMS(/MS) method optimisation 17
3.2 Summary of all *D. magna* metabolite annotations and compound classifications 17
3.3 Metabolites and compound classes physicochemically separated by DMA experimental workflow 23
3.4 Comparison to other metabolite databases 26
3.5 Molecular network analysis using GNPS 29
3.6 DMAdb: Public Access and Functionalities 33
4 Conclusion 35
5 Code availability 38
6 Additional files 41
7 Abbreviations 44
8 Acknowledgements 44
9 Author contributions 45
10 Funding 45
11 Data availability 46
12 Competing interests 46
13 References 46
14 Author notes 50
~~1 Introduction 4~~
~~2 Methods 7~~
~~2.1 Deep metabolome annotation experimental workflow 7~~
~~2.1.1 Overview 7~~
~~2.1.2 D. magna culturing and sample preparation 7~~
~~2.1.3 Metabolite extraction from D. magna pooled sample 7~~
~~2.1.4 Solid phase extraction-based fractionation of metabolite extracts 8~~
~~2.1.5 (U)HPLC-HRMS(/MS), DI-HRMS(/MSn) and LC fractionation 8~~
~~2.1.6 (U)HPLC-HRMS(/MS) method optimisation 9~~

**Formatted:** Default Paragraph Font, Check spelling and grammar

**Formatted:** Default Paragraph Font, Check spelling and grammar

**Formatted:** Default Paragraph Font, Check spelling and grammar

**Formatted:** Default Paragraph Font, Check spelling and grammar

**Formatted:** Default Paragraph Font, Check spelling and grammar

**Formatted:** Default Paragraph Font, Check spelling and grammar

**Formatted:** Default Paragraph Font, Check spelling and grammar

**Formatted:** Default Paragraph Font, Check spelling and grammar

**Formatted:** Default Paragraph Font, Check spelling and grammar

**Formatted:** Default Paragraph Font, Check spelling and grammar

**Formatted:** Default Paragraph Font, Check spelling and grammar

**Formatted:** Default Paragraph Font, Check spelling and grammar

**Formatted:** Default Paragraph Font, Check spelling and grammar

**Formatted:** Default Paragraph Font, Check spelling and grammar

**Formatted:** Default Paragraph Font, Check spelling and grammar

**Formatted:** Default Paragraph Font, Check spelling and grammar

**Formatted:** Default Paragraph Font, Check spelling and grammar, Not Superscript/ Subscript

**Formatted:** Default Paragraph Font, Check spelling and grammar

**Formatted:** Default Paragraph Font, Check spelling and grammar

**Formatted:** Default Paragraph Font, Check spelling and grammar

2.1.7 GC-EI-HRMS 9
2.1.8 1D & 2D NMR spectroscopy 9
2.2 Computational tools and workflows for data processing, metabolite annotation and data analysis 11
2.2.1 Overview 11
2.2.2 Galaxy workflow details 11
2.2.3 mzCloud library search 13
2.2.4 GNPS library search 13
2.2.5 GNPS molecular network analysis 13
2.2.6 Combining and summarising all annotations 13
2.2.7 Comparison to other metabolite databases 14
2.3 Assessment of the computational and experimental DMA workflow with metabolite reference standards 14
2.4 Deep Metabolome Annotation Database and Web Portal (DMAdb) 14
3 Results and discussion 16
3.1 (U)HPLC-HRMS(/MS) method optimisation 16
3.2 Summary of all *D. magna* metabolite annotations and compound classifications 16
3.3 Metabolites and compound classes physicochemically separated by DMA experimental workflow 21
3.4 Comparison to other metabolite databases 24
3.5 Molecular network analysis using GNPS 26
3.6 DMAdb: Public Access and Functionalities 28
4 Conclusion 28
5 Code availability 31
6 Additional files 34
7 Abbreviations 37
8 Acknowledgements 37
9 Author contributions 38
10 Funding 38
11 Data availability 39
12 Competing interests 39
13 References 39
14 Author notes 42

Formatted ...
Formatted ...
Formatted ...
Formatted ...
Formatted ...
Formatted ...
Formatted ...
Formatted ...
Formatted ...
Formatted ...
Formatted ...
Formatted ...
Formatted ...
Formatted ...
Formatted ...
Formatted ...
Formatted ...
Formatted ...
Formatted ...
Formatted ...
Formatted ...
Formatted ...
Formatted ...
Formatted ...
Formatted ...
Formatted ...
Formatted ...
Formatted ...
Formatted ...
Formatted ...
Formatted ...
Formatted ...
Formatted ...
Formatted ...
Formatted ...
Formatted ...
Formatted ...
Formatted ...
Formatted ...
Formatted ...
Formatted ...
Formatted ...
Formatted ...
Formatted ...
Formatted ...
Formatted ...
Formatted ...
Formatted ...
Formatted ...
Formatted ...
Formatted ...
Formatted ...
Formatted ...
Formatted ...
Formatted ...
Formatted ...

# 1 Introduction

Large-scale efforts to map and catalogue both human and model organism genomes have been a fundamental driver of change in biological and biochemical research over the past few decades. The technological developments and resulting biological, biomedical and environmental knowledge derived from such projects have helped underpin the modern era of biological sciences [1], [2], [3], [4]. In contrast, our understanding of metabolic biochemistry (where we use the term metabolites here to represent the full spectrum of low molecular weight endogenous biochemicals from polar metabolites to lipids) has increased relatively minimally over the last half-a-century. Such knowledge must either be inferred from genome-scale metabolic reconstructions or, if measured experimentally, is limited to metabolites that can be annotated analytically (i.e. using metabolomics datasets). Ongoing improvements in both analytical and computational methods for metabolic annotation now allow for more extensive metabolite annotation coverage than what could be performed 10 years ago. However, although these developments are welcomed and are in part reflected by the increase in both studies featuring extensive metabolite annotation analysis [5], [6], [7] and the maturation of databases containing metabolites and relevant experimental data (Metabolights, Metabolomics Workbench, HMDB, GNPS, MoNA, MassBank, LipidBlast and mzCloud [8], [9], [10], [11], [12], [13], for the majority of widely used model organisms the metabolome knowledge is still severely lacking.

Whilst the need for deeper metabolome knowledge of model organisms has been well established [14], the challenges are still considerable and multi-faceted. First, the biology: as the metabolome is driven by genes, the changing environment, and their interactions, achieving a comprehensive map of the breadth of a species' metabolome requires a range of genotypes and phenotypes. Second, analytical chemistry: no single method is sufficient to cover the chemical space of a metabolome, hence multiple physicochemical separations and detection techniques are required. Third, the computational challenges: as metabolomes are vast, specialised tools and workflows for data processing and metabolite annotation are required, together with resources for data and metadata management. Where possible, the data generated and software used should be Findable, Accessible, Interoperable, Reusable (FAIR) and scalable in order to support an anticipated further cascade of deep metabolome annotation studies.

To address these challenges, we have developed and applied an experimental and computational workflow for extensively measuring the metabolome of model organisms, applied here to *Daphnia magna*. The International Metabolomics Society's Model Organism Metabolomes task group [15] and the on-going Precision Toxicology project [16] both highlight the crustacean *Daphnia* as a key model organism due to its importance as an indicator genus used to set ecotoxicological regulatory standards (SOR/2002-222), and from being extensively studied in the context of evolution and ecology [17], [18], [19], [20], [21], making it an excellent candidate for an in-depth investigation of its metabolome. Experimentally, the workflow involved culturing multiple ~~genotypically distinct~~ strains of *D. magna* under normal and stressed conditions to provide a representative pooled sample for metabolome annotation. This sample underwent extensive extraction and physicochemical separation and (ultra)-high-performance liquid chromatography-high resolution tandem mass spectrometry ((U)HPLC-HRMS(/MS)) analysis. Concurrent fractionation yielded (semi-)purified metabolome fractions that underwent in-depth characterisation by direct infusion-

high resolution multiple stage mass spectrometry (DI-HRMS(MS$^n$)). ~~, and s~~Supplemental ~~analysis~~ analyses by gas chromatography-electron ionisation-high resolution mass spectrometry (GC-EI-HRMS) and 1- and 2-dimensional nuclear magnetic resonance spectroscopy (1D- & 2D-NMR), ~~ensuring~~ helped to ensure broad coverage of the physicochemical space of metabolites ~~were measured~~. A computational workflow was then developed and applied for processing, annotating and managing the data and results, heavily utilising the Galaxy Workflow platform [22]. The resulting metabolome annotations, data, metadata and computational tools are disseminated through various channels (MetaboLights, GNPS, Galaxy and a custom web portal, named DMAdb) to ensure traceability and reusability.

The combined extensive experimental and computational workflow, referred to here as the Deep Metabolome Annotation (DMA) workflow (see **Figure 1**), has generated one of the largest metabolome annotation datasets for any single organism and provides the first in-depth characterisation of the *D. magna* metabolome – a resource that is much needed to improve the interpretation of *Daphnia* biology and toxicology. This workflow can ~~be~~ also be redeployed for deep metabolome annotation of other model organisms.

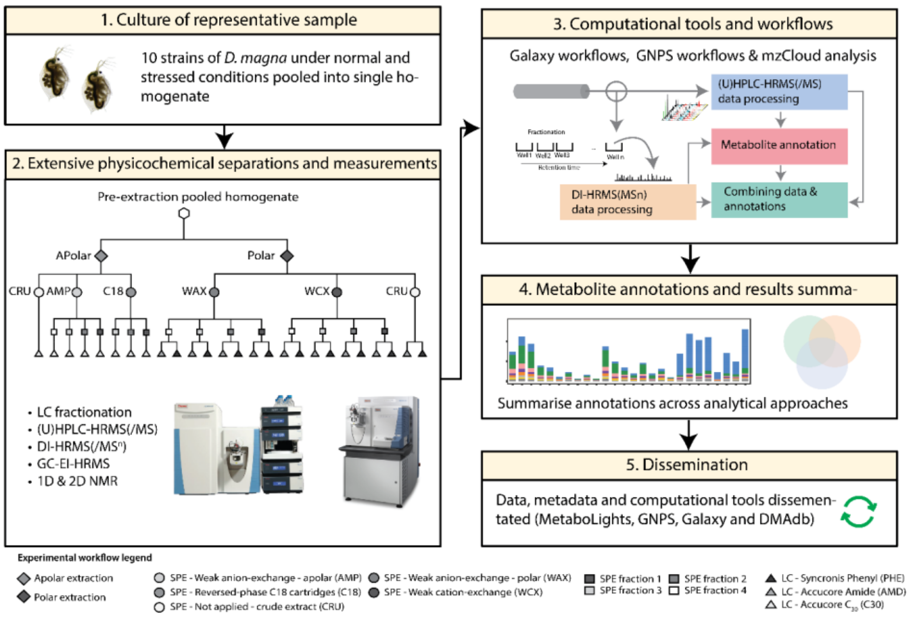

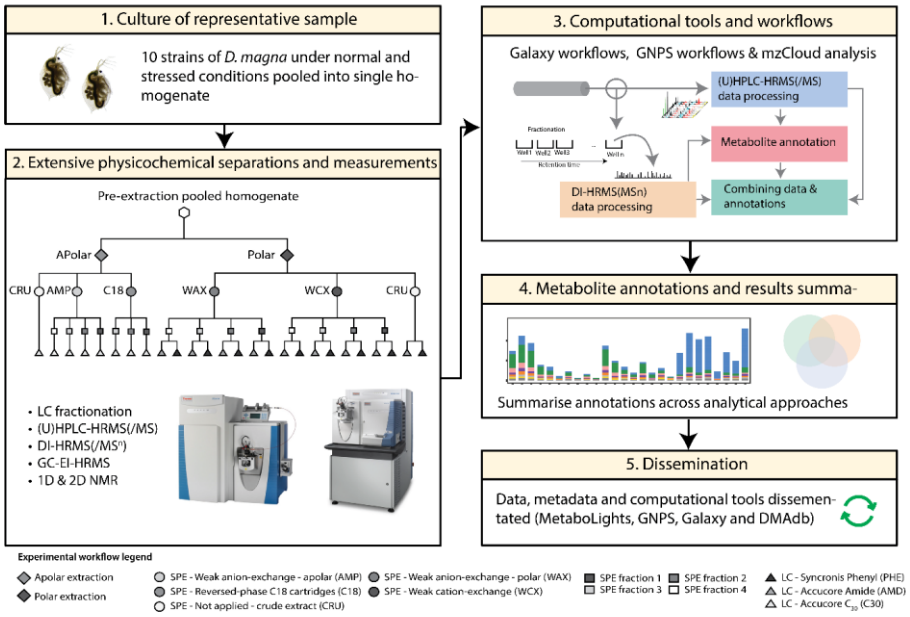


***Figure 1: Deep Metabolome Annotation (DMA) workflow - conceptual overview and application to D. magna***

***1)*** *First a representative sample (applied to D. magna here) cultured under multiple conditions using ~~genotypically~~ distinct strains ~~is~~are homogenised into a single sample.* ***2)*** *The pooled homogenised sample then undergoes extensive physicochemical separations (including polar and apolar extraction procedures; four types of solid phase extraction (SPE) – each collecting 3-4 SPE fractions; three types of liquid chromatography (LC); and extensive LC fractionation). ~~Followed by extensive analysis using~~SPE fractions then undergo extensive analysis based on (ultra)-high-performance liquid chromatography-high resolution tandem mass spectrometry ((U)HPLC-HRMS(/MS)), followed by~~,~~ direct infusion-high resolution mass spectrometry with multiple-stage fragmentation (DI-HRMS(MS$^n$))~~)~~. SPE fraction analyses are also ~~and~~ supplemented with gas chromatography-electron ionisation-high resolution mass spectrometry (GC-EI-HRMS) and 1- and 2-dimensional nuclear magnetic resonance spectroscopy (1D- & 2D-NMR).* ***3)*** *Extensive computational tools and workflows were developed and applied to process and annotate the metabolite. The results are then summarised across the analytical workflow* ***(4)*** *and finally the data, metadata and computational tools are disseminated to ensure traceability and reusability* ***(5)****. Images of Thermo Scientific Q Exactive and Orbitrap Elite mass spectrometers by Thermo Fisher Scientific (Bremen), licensed under Creative Commons Attribution–ShareAlike 3.0 Unported (CC BY-SA 3.0), via Wikimedia Commons.*

*ALT TEXT: Graphical representation of the four stages of the deep metabolome annotation workflow.*

**Formatted:** Font: Italic

**Formatted:** Font: Italic

# 2 Methods

## 2.1 Deep metabolome annotation experimental workflow

### *2.1.1 Overview*

An experimental workflow for extensive physicochemical separation and analytical measurement of metabolites has been developed for the analysis of model organisms – applied here to *D. magna*. An overview is provided in **Figure 2**.

Formatted: Font: Bold

This extensive workflow, applied to both the *Daphnia* sample and a metabolite reference standard sample, was separated into 135 distinct experimental assays (see **Supplemental Table S1**). Of these assays, 103 corresponded to (U)HPLC-HRMS(/MS) and DI-HRMS(/MS$^n$) analysis, generating 8,846 raw mass spectrometry files (5,430 files specifically measuring the *Daphnia* sample with the remaining files either for metabolite reference standards, blanks or quality assurance measurements such as (U)HPLC-HRMS system mass spectrometer equilibration). See **Supplemental Table S2** for the full file list.

### *2.1.2 D. magna culturing and sample preparation*

The DMA experimental workflow is applied here to *D. magna*, but the same considerations apply for other potential model organism DMA analyses. The workflow should take as input a set of samples that, ideally, span diverse genetic and environmental backgrounds and collectively reflect the full metabolic repertoire accessible to the organism under study. These samples are then pooled and homogenised to form a single complex sample matrix that constitutes an average of the constituent metabolomes.

For the DMA of *D. magna*, ca. 2000 individual organisms were pooled and homogenised, generating a homogenate consisting of ten genotypically distinct strains (**Supplemental Table S3)** exposed to two contrasting environmental conditions. A ‘basal’ metabolome was represented by *D. magna* cultured under standard conditions (20 +/- 2 °C with a 16:8 hr light:dark ratio) for 14 days, followed by a further 48 hr under the same conditions. A ‘stressed’ metabolome was represented by *D. magna* cultured for 14 days under standard conditions, followed by 24 hr at 10 +/- 1 °C with 16:8 hr light:dark ratio, and then a further 24 hr at 10 +/- 1 °C with 8:16 hr light:dark ratio. Under both conditions, *Daphnia* were maintained without food (algae) throughout the final 48 hr of culturing, to minimise the presence of algae in the gut and prioritise measurement of metabolites derived from *Daphnia* rather than the food source. Further details are provided in **Supplemental Section 1.5** and **Supplemental Tables S4-5**.

### *2.1.3 Metabolite extraction from D. magna pooled sample*

Metabolites were extracted from the pooled homogenate using two distinct liquid-phase extraction protocols: a ‘polar’ extraction in which (predominantly polar through to moderately-polar polar) metabolites were extracted using a solution comprising 71.4:28.6% v/v methanol:water, and; an ‘apolar’ extraction protocol in which metabolites (spanning moderately-apolar through to highly apolar metabolites, including lipids) were extracted using a solution of 1:1 v/v methanol:chloroform, to which water was added to form a biphasic system comprising 2:2:1.8 v/v/v chloroform:methanol:water, from which the lower (apolar)

layer was recovered. Polar extracts were dried in a centrifugal vacuum concentrator (Speedvac), while apolar extracts were dried under a stream of nitrogen gas. Further details are provided in **Supplemental Section 1.6**.

#### *2.1.4 Solid phase extraction-based fractionation of metabolite extracts*

Constituents of the polar or apolar extract were independently fractionated over two solid-phase extraction (SPE) cartridges. The polar extract was fractionated using weak anion-exchange (WAX; aminopropyl) and weak cation-exchange cartridges (WCX; carboxylic acid), while the apolar extract was fractionated using weak anion-exchange (referred to as AMP to differentiate from the polar arm; aminopropyl) and reversed-phase C18 cartridges (C18). See **Supplemental Section 1.7** and **Supplemental Figures S1-2** for further details. The resulting 15 SPE fractions and remaining unfractionated extracts (referred to as "crude" extract) were analysed by (U)HPLC-HRMS(/MS). A selected subset of SPE fractions, alongside crude extract, were also analysed by 1D- & 2D-NMR spectroscopy and GC-EI-HRMS, to further expand the breadth of metabolome annotation.

#### *2.1.5 (U)HPLC-HRMS(/MS), DI-HRMS(/MS$^n$) and LC fractionation*

Three distinct (U)HPLC-HRMS(/MS) methods were applied in both positive and negative ionisation modes to analyse constituents of the polar and apolar crude extracts, and associated SPE fractions. Polar crude extract and polar SPE fractions were analysed by hydrophilic interaction liquid chromatography (HILIC) using an Accucore amide column (AMD; 2.1 x 100 mm, 2.6 µm solid core; Thermo Scientific), and reversed-phase liquid chromatography (RPLC) based on a Syncronis phenyl column (PHE; 2.1 x 100 mm, 1.7 µm; Thermo Scientific). Constituents of the apolar crude extract and apolar SPE fractions, meanwhile, were analysed by RPLC using an Accucore C30 column (C30) (2.1 x 100 mm, 2.6 µm solid-core particle, 150 Å; Thermo Scientific). All chromatographic separations were performed using a Dionex Ultimate 3000 liquid chromatography system. A Q Exactive mass spectrometer (Thermo Scientific), fitted with heated electrospray ionisation source, was used for HRMS(/MS) mass spectrometry analysis of metabolites eluted from LC columns. A passive flow splitting tee-piece was installed between the LC column outlet and Q Exactive inlet to facilitate simultaneous collection of 20-second-wide LC fractions and associated HRMS(/MS) data. Each fraction was collected into independent wells of a deep well plate. LC fraction collection plates were dried in a centrifugal evaporator at the end of an analysis sequence.

Initial (U)HPLC-HRMS analyses were used to create inclusion and exclusion lists (i.e. *m/z* features of interest) to direct the subsequent data dependent acquisition (DDA) of (U)HPLC-HRMS/MS data. In parallel, eluent from the LC columns were fraction-collected during mass spectral acquisition, which were then subject to extensive DI-HRMS(/MS$^n$).

DI-HRMS(/MS$^n$) analyses of resuspended LC fractions were performed using an Orbitrap Elite mass spectrometer (Thermo Scientific) using both higher energy collisional dissociation (HCD) and collision-induced dissociation (CID) at several levels of normalised collision energy (NCE). Specifically, HCD was performed at 20, 40 and 80% NCE, followed by CID at 35% NCE with multi-stage fragmentation up to MS$^3$. In total, 2,305 LC fractions were analysed as part of the DMA of *D. magna*. The acquisition of DI-HRMS(/MS$^n$) fragmentation

data was directed via a predefined list of targeted *m/z* features, derived from prior DI-HRMS analysis of the same fraction.

For detailed information on the (U)HPLC-HRMS analytical setup and LC methods, including the fractionation procedure, as well as the data acquisition sequence and computational methods used to create inclusion/exclusion lists for targeting the most informative features for fragmentation data acquisition, ~~(S~~see **Supplemental Sections 1.8.1 and Supplemental Figure S3**~~)~~. Additionally, for more details on the resuspension of LC fractions, the DI-HRMS(/MS$^n$) analytical setup, data acquisition sequence, and the computational methods for DI-HRMS(/MS$^n$) used to develop both inclusion/~~ and ~~exclusion lists and instrument methods files that directed DI-HRMS$^n$ data acquisition, ~~(S~~see **Supplemental Sections 1.8.2** and **Supplemental Figure S4**~~)~~.

### 2.1.6 (U)HPLC-HRMS(/MS) method optimisation

The PHE and AMD (U)HPLC-HRMS(/MS) methods underwent optimisation for DMA of *D. magna* aiming to maximize reproducibly detectable metabolic features while enabling reproducible fractionation for downstream analyses. The methodology for the optimisation is detailed within the **Supplemental Section 1.9, Figure S5** and **Supplemental Tables S6-S8.** The C30 (U)HPLC-HRMS(/MS) method was previously optimised for broad lipid profiling applications by Thermo Fisher Scientific, hence no further optimisation was pursued.

### 2.1.7 GC-EI-HRMS

GC-EI-HRMS was performed on the WAX and WCX fractions, as well as the crude polar extract, using a TriPlus RSH autosampler and TRACE 1310 gas chromatograph coupled to a Q Exactive mass spectrometer (Thermo Scientific), and an Extractabrite electron ionisation/chemical ionisation source. Further details are provided in **Supplemental Section 1.10**.

### 2.1.8 1D & 2D NMR spectroscopy

The WAX and WCX SPE fractions were also analysed using a combination of 1D and 2D-NMR spectroscopy experiments performed using a Bruker AVANCE III 600 MHz NMR spectrometer, equipped with a 1.7 mm TCI-Cryoprobe and operated at a proton frequency of 600.13 MHz. Each sample was measured using 1D proton nuclear overhauser effect NMR spectroscopy (1D-$^1$H-NOESY) followed by 2D homonuclear $^1$H-$^1$H (2D-JRes and TOCSY) and heteronuclear $^1$H-$^{13}$C (HSQC) experiments to support annotation. Further details are provided in **Supplemental Section 1.11**.

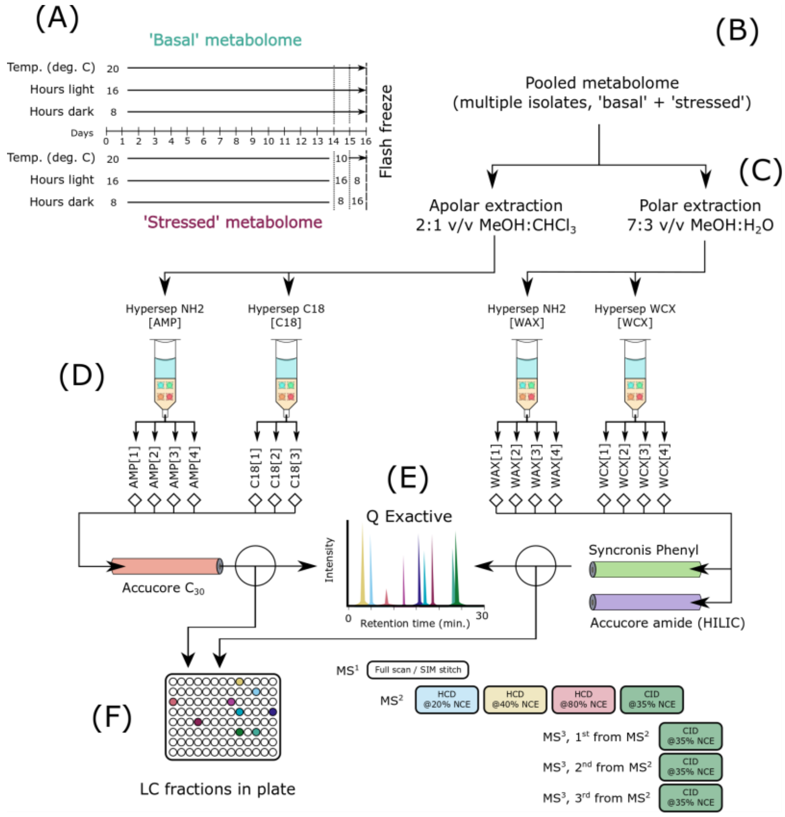


**Figure 2: Deep metabolome annotation experimental workflow for the physicochemical separation and measurement of *D. magna* metabolites.** ***A)*** *Culturing: Culturing of ten ~~genotypically distinct~~ strains of a model organism under normal (basal) and stressed conditions was used to ensure a wide range of metabolites were present in* ***B)*** *the pooled sample. For the DMA of D. magna this involved flash freezing in liquid nitrogen to quench metabolism, followed by homogenisation and pooling into a single sample.* ***C)*** *Liquid phase extraction: Two distinct liquid phase extractions were then performed on the pooled homogenate from step B), for 'polar' and 'apolar' metabolites.* ***D)*** *Solid phase extraction: Four types of solid phase extraction (SPE) were performed on the extracts from step C), the polar extracts using weak anion-exchange (WAX) and weak-cation exchange SPE cartridges (WCX) and apolar extracts using weak anion-exchange (AMP) and reversed-phase C18 cartridges (C18).* ***E)*** *(U)HPLC-HRMS(/MS): Three distinct (U)HPLC-HRMS(/MS) methods were applied using a Q-Exactive mass spectrometer: Accucore amide HILIC LC column (AMD) and Syncronis phenyl LC column (PHE) for analysis of metabolites from the polar arm of the workflow, and Accucore C30 LC column (C30) for the apolar extract and any SPE fractions derived from the apolar extract.* ***F)*** *Fractionation and DI-HRMS(/$MS^n$): The eluate from the LC columns in step E) was fractionated into plates for subsequent extensive multiple-stage fragmentation ($MS^n$) analysis applied (up to $MS^3$), including at multiple collision energies and with technical replication.*

*ALT TEXT: Graphical representation of the Deep Metabolome Annotation experimental workflow.*

## 2.2 Computational tools and workflows for data processing, metabolite annotation and data analysis

### *2.2.1 Overview*

An extensive computational workflow utilising the Galaxy platform has been developed to analyse the highly complex data acquired through the experimental DMA workflow. This computational workflow predominantly consists of an extensive Galaxy-based workflow, with additional annotations incorporated from external sources i.e., mzCloud, GNPS workflows, GC-EI-HRMS annotations and 1D & 2D NMR annotations. A summary is provided in **Supplemental Section S12** and **Supplemental Figure S6**.

The Galaxy workflow component generated 104 Galaxy histories (see **Supplemental Table S1** for links to corresponding Galaxy history), 60 of which were used for the analysis of the *Daphnia* samples. Each Galaxy history contains a combined SQLite database containing all annotations and relevant (average) spectra across all assays.

### *2.2.2 Galaxy workflow details*

The Galaxy workflow (see **Figure 3**) was designed specifically to process and perform metabolite annotation across the multiple data types produced by the DMA experimental workflow, including (U)HPLC-HRMS(/MS) and DI-HRMS(/MS$^n$). Utilising the high level of replication achieved from the DMA experimental workflow, averaging and filtering was performed on both the (U)HPLC-HRMS(/MS) and DI-HRMS(/MS$^n$) data so that higher quality reproducible fragment peaks were used for multiple complementary computational approaches to metabolite annotation.

The workflow is split into five components: “Data input”, “(U)HPLC-HRMS(/MS) data processing”, “DI-HRMS(/MS$^n$) data processing”, “Metabolite annotation” and “Combining”. Detailed description of all steps used in the workflow can be found in **Supplemental Section 1.13 –** with individual schematics detailing the (U)HPLC-HRMS(/MS) (See **Supplemental Figure S7**) and DI-HRMS(/MS$^n$) fragmentation data processing (See **Supplemental Figure S87**). See also **Supplemental Table S9** for the description of all tools used and **Table 1** for the location of code repositories for each Galaxy tool and underlying software.

The Galaxy workflow incorporates both new and existing tools, e.g. existing Workflow4Metabolomics XCMS Galaxy tools [23] for (U)HPLC-HRMS peak picking and processing; DIMSpy Galaxy tools [24], [25] ~~(https://github.com/computational-metabolomics/dimspy-galaxy)~~ for DI-HRMS data processing. New Galaxy tools developed for the DMA project include updated functionality from the msPurity R package [26] to filter and flag spectra, average fragmentation spectra, create MSP and SQLite files of (U)HPLC-HRMS(/MS) data, perform spectral matching, and combine metabolite annotations from multiple sources; the MSnPy python package and Galaxy tools to process DI-HRMS(/MS$^n$) data with both multiple-stage and multiple energy fragmentation spectral trees, perform spectral averaging across trees, and annotate and rank spectral trees with molecular formulae; and the LC Fractionation Galaxy tool which was created to combine all spectra and metabolite annotations from a DMA LC fractionation experiment. In addition, Galaxy wrappers have been created for the *in silico* fragmentation software MetFrag [27], [28], [29]

and mass spectrometry data processing and metabolite annotation software SIRIUS -CSI:FingerID [30].

This modular, multistage workflow ensures reusability across other studies. Additionally, a public Galaxy instance (https://dma.galaxy.bham.ac.uk) was created for executing the Galaxy DMA analysis, with public access to the histories, data and parameters.

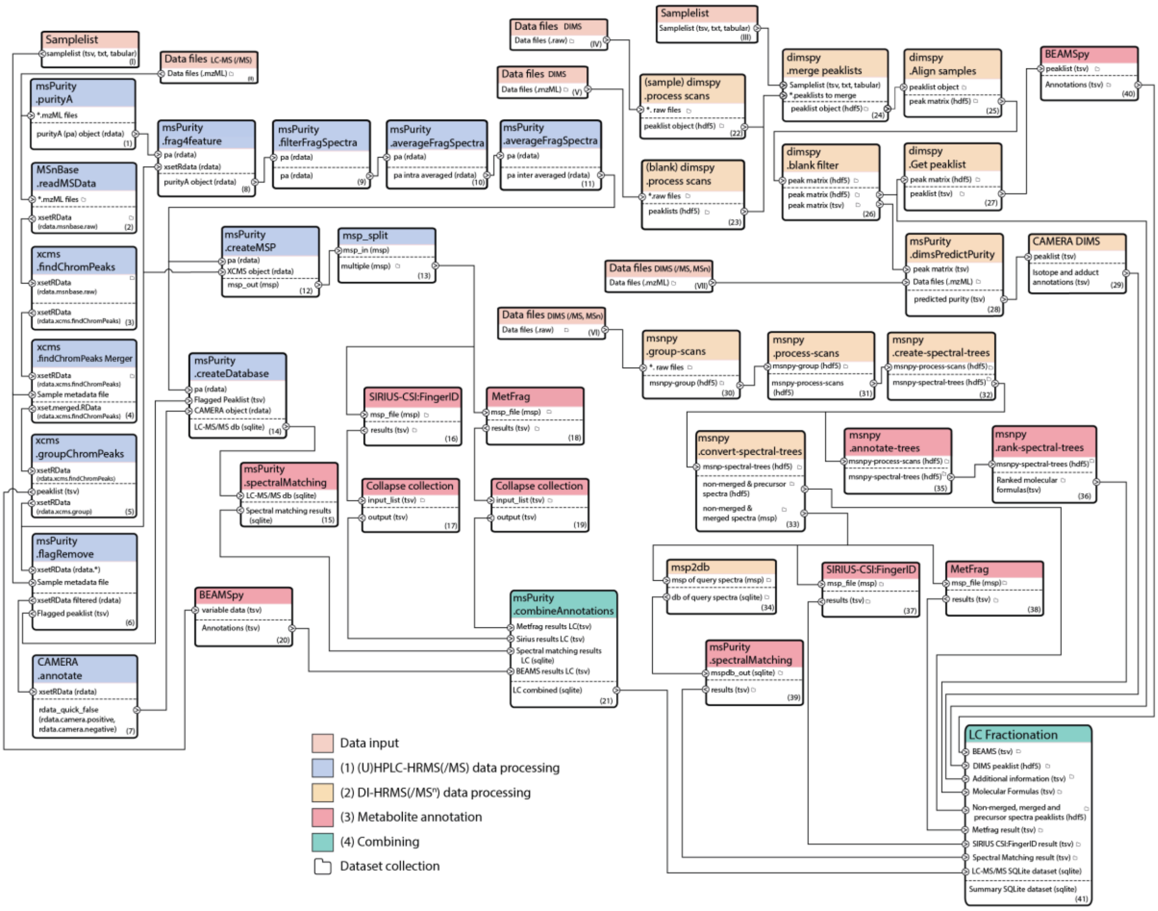


***Figure 3: Deep Metabolome Annotation data processing and metabolite annotation Galaxy workflow.*** *Components are separated by colour into "Data input", "(U)HPLC-HRMS(/MS) data processing", "DI-HRMS(/MS$^n$) data processing", "metabolite annotation" and "Combining". See* ***Supplemental Table S94*** *for description of each tool.*

*ALT TEXT: Graphical representation of the deep metabolome annotation Galaxy workflow.*

### 2.2.3 mzCloud library search

All annotations derived from mzCloud (https://www.mzcloud.org/) were performed programmatically in batches using mzCloud proprietary software [31]. Each fragmentation scan was treated individually, and spectral matching was performed against the mzCloud database. The results were saved as an SQLite database with reference to both the query and library spectra of each annotation. The data were filtered to only include “endogenous” metabolites and spectral matches with dot product cosine score > 0.7.

### 2.2.4 GNPS library search

Fragmentation spectra were searched against the GNPS library spectra using the online workflow (https://ccms-ucsd.github.io/GNPSDocumentation/) on the GNPS website (http://gnps.ucsd.edu) [11], [32]. The precursor ion mass tolerance was set to 0.02 Da and a MS/MS fragment ion tolerance of 0.02 Da. Additionally, spectral matches were filtered to have an error of ≤ 10 ppm between the library precursor m/z and the query precursor *m/z*, and annotations were filtered to only include spectral matching results for which the library and query spectra were derived from a mass spectrometer using the same ionisation mode.

### 2.2.5 GNPS molecular network analysis

A molecular network was created using the online workflow (https://ccms-ucsd.github.io/GNPSDocumentation/) on the GNPS website (http://gnps.ucsd.edu) [11], [32]. The data was filtered to remove all fragment ions within ± 17 Da of the precursor *m/z*. Fragmentation spectra were window filtered by retaining only the top 6 fragment ions in the ± 50 Da window throughout the spectrum. Both the precursor ion mass tolerance and fragment ion tolerance were set to 0.02 Da. A network was then created where edges were filtered based on having a cosine score >0.7 and more than two matched peaks. Further, edges between two nodes were only kept in the network if each of the nodes appeared in each other's respective top 10 most similar nodes. Finally, the maximum size of a molecular family was set to 100, and the lowest scoring edges were removed from molecular families until the molecular family size was below this threshold. The fragmentation spectra in the network were then searched against GNPS’s spectral libraries. The library spectra were filtered in the same manner as the input data. All matches kept between network and library spectra were required to have a score above 0.7 and at least 2 matched peaks. Further annotation was performed using the Dereplicator tool [33] and the MS2LDA [34] workflow to determine common mass motifs.

### 2.2.6 Combining and summarising all annotations

Five main sources of annotations were combined into a final list of Metabolite annotations containing: Galaxy workflow annotations, GNPS workflow annotations, mzCloud annotations, NMR annotations and GC-EI-HRMS annotations. All data were combined into a single table encompassing all annotations across every assay and a final stage of filtering was performed to as described in **Supplemental Section 1.14.**

All annotations were chemically classified using ClassyFire [35].

### 2.2.7 Comparison to other metabolite databases

The final list of *D. magna* metabolites were compared to compound lists from KEGG [36], [37], [38], ChEBI [39], HMDB [8] and MTox700+ [40]. ChEBI was filtered for only those compounds with a known species origin. PhyloT~~tree~~ ~~(https://phylot.biobyte.de/index.cgi)~~ was used to generate the species phylogenetic tree using the NCBI taxonomy [41]. Matching was based on compounds sharing the same partial InChI~~i~~Key (i.e. the first block of the InCh~~i~~IKey that encodes the molecular skeleton).

QIAGEN Ingenuity Pathway Analysis (IPA, QIAGEN Inc.) was used to derive metabolite-pathway associations. A metabolite list containing PubChem, HMDB and KEGG identifiers was imported into the IPA software. 'Metabolomics core analysis' was then conducted using all mapped metabolites.~~, with the IPA database serving as a reference set.~~

## 2.3 Assessment of the computational and experimental DMA workflow with metabolite reference standards

Metabolite reference standards (see **Supplemental Table S11)** were analysed to evaluate the effectiveness of the overall DMA workflow, specifically the (U)HPLC-HRMS(/MS) component of the experimental workflow, including extraction, chromatographic separation, and mass spectrometric analysis. The same computational workflow was applied as used for the *D. magna* samples. Next, the measured metabolite reference standards were compared to the expected annotations, for each assay, by matching the partial InChI~~i~~Key (i.e., first block).

## 2.4 Deep m~~M~~etabolome a~~A~~nnotation d~~D~~atabase and w~~W~~eb p~~P~~ortal (DMAdb)

~~The DMA database (DMAdb) was implemented using a Python-Django framework and consists of a web portal and underlying database designed for data management and analysis. While established for the *D. magna* results, it also serves as a prototype for future DMA projects. The DMAdb allows users to organise DMA experiments within the ISA framework, supports interactions with Galaxy, enabling the metabolite annotations to be viewed and searched through a graphical user interface. The codebase for the DMAdb web portal was developed as three Django applications (django-gfiles, django-galaxy and django-mogi) specifically designed for metabolomics data organisation with Galaxy and the ISA framework (see https://dmadb.readthedocs.io/en/latest/).~~The DMA database and web portal (DMAdb; **Figure 4**) was developed to organise, manage, and disseminate DMA datasets and associated metabolite annotations. It was implemented using Django, a high-level Python web framework, and comprises multiple applications (i.e. django-gfiles, django-galaxy, and django-mogi, see Code Availability). Experimental datasets were structured using the Investigation/Study/Assay (ISA) framework to support standardised organisation of experimental data and metadata. This structure enables consistent metadata capture, facilitates interoperability with community standards, and enables traceability between raw data, processing workflows, and metabolite annotations. DMAdb was primarily designed as an internal, administrator-restricted data management platform with Galaxy workflow integration. However, controlled public access to selected functionalities is provided to enable basic exploration and searching of DMA datasets, as described in the Results

**Formatted:** Font: Bold

section.

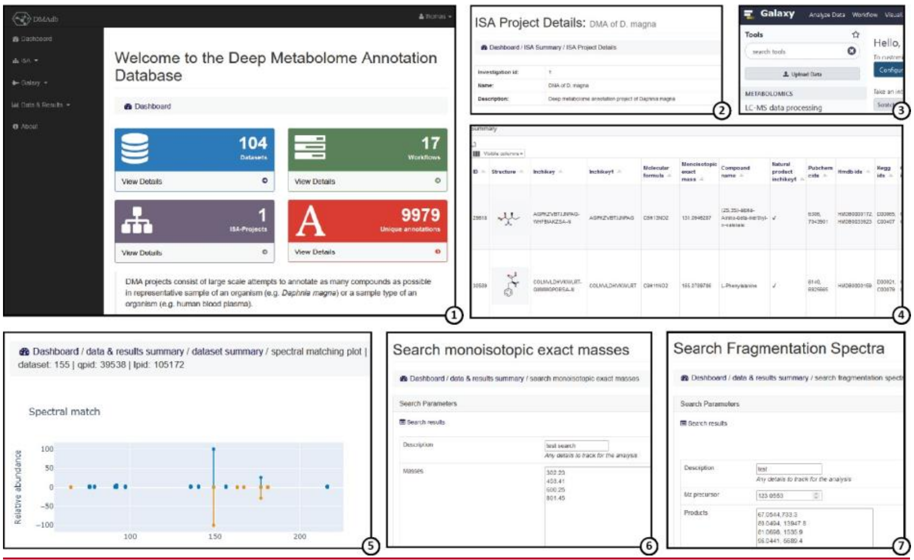


***Figure 4. Overview of the Deep Metabolome Annotation database and web portal (DMAdb) developed and implemented to organise, manage, and disseminate DMA data and associated metabolite annotations of Daphnia magna.*** *(1) Dashboard view of the DMAdb interface. (2) Investigation/Study/Assay (ISA) representation of the DMA data, capturing relevant ontologies, protocols, and experimental processes. (3) Data processing and annotation workflows performed via the Galaxy platform, with outputs uploaded to and stored within DMAdb. (4–5) Browsing and visualisation of metabolite annotations and associated raw data files. (6–7) Search functionalities supporting exact mass queries and MS/MS-based spectral matching.*

*ALT TEXT: Summary of the different web interfaces of the Deep Metabolome Annotation database and web interface (DMAdb).*

# 3 Results and discussion

## 3.1 (U)HPLC-HRMS(/MS) method optimisation

The PHE and AMD (U)HPLC-HRMS(/MS) methods were optimis~~z~~ed to improve detection of reliable metabolic features and support reproducible fractionation. Full details and supporting figures are provided in **Supplemental Section 2.1, Supplemental Table S12** and **Figures S9-S26**.

## 3.2 Summary of all *D. magna* metabolite annotations and compound classifications

In total, 8,181~~577~~ unique metabolite annotations are reported from all experimental assays (including (U)HPLC-HRMS(/MS), DI-HRMS(/MS$^n$), 1D- & 2D-NMR and GC-EI-HRMS), summarised in **Supplemental Table S13**. The combined annotations and compound classifications across all technologies and approaches are presented here, with further details including specifics for each measurement technology provided in **Supplemental sections 2.2-2.5.** In summary, the majority of annotations were reported for the (U)HPLC-HRMS(/MS) and DI-HRMS(/MS$^n$) datasets, observing 8,~~52~~132~~8~~ unique annotations versus just four unique metabolites for GC-EI-HRMS and three~~3~~ for 1D- & 2D-NMR~~ ()~~,; ~~-~~see Venn diagram in **Supplemental Figure S27**~~)~~. Although the discrepancy in observed annotations across these analytical technologies appears large, with annotations derived from (U)HPLC-HRMS(/MS) and DI-HRMS(/MS$^n$) dominating the counts, this is consistent with the analytical prioritisation of these technologies and the comparatively more extensive computational interrogation applied to them.~~This can be explained in part by the relative sizes of each dataset.~~

Compound classification via ClassyFire was possible for~~ ~~ 7,934 (97%) ~~8,324 (97%)~~ metabolites to at least the level of "Superclass". The remaining unclassified metabolites had either incompatible SMILES for ClassyFire or lacked a SMILES annotation from PubChem. See **Figure 5~~4~~A** for a treemap summarising all annotation superclasses and classes, demonstrating the diverse biochemical space observed. The chemical space was further explored using principal component analysis (PCA) of the PubChem molecular fingerprints, showing broad clustering of metabolites based on their structure, with no obvious outliers (see **Figure 5~~4~~B**).

The most common "superclass" observed was for lipids and lipid-like molecules (3,202~~391~~ uniquely annotated metabolites) followed by organic acids and derivatives (2,~~223~~173); organoheterocyclic compounds (656~~77~~); organic oxygen compounds (~~604~~572); benzenoids (~~530~~489); phenylpropanoids and polyketides (~~329~~306); organic nitrogen compounds (~~230~~221); nucleosides, nucleotides, and analogues (1~~93~~85); organosulfur compounds (4~~5~~4); alkaloids and derivatives (32~~9~~); hydrocarbons (~~21~~17); organophosphorus compounds (17); lignans, neolignans and related compounds (1~~5~~1); and ~~fi~~three~~ve~~ other "superclasses" with six or fewer annotated metabolites. A total of 247~~53~~ metabolites could not be classified to a "superclass" level.

The most common "class" classification observed was for carboxylic acids and derivatives (1,776~~809~~) followed by fatty acyls (~~984~~913); glycerophospholipids (~~828~~803); organooxygen

**Formatted:** Not Highlight

**Formatted:** Not Highlight

**Formatted:** Not Highlight

**Formatted:** Not Highlight

**Formatted:** Not Highlight

**Formatted:** Not Highlight

**Formatted:** Not Highlight

**Formatted:** Not Highlight

**Formatted:** Not Highlight

**Formatted:** Not Highlight

**Formatted:** Not Highlight

**Formatted:** Not Highlight

**Formatted:** Not Highlight

**Formatted:** Not Highlight

**Formatted:** Not Highlight

**Formatted:** Not Highlight

**Formatted:** Not Highlight

**Formatted:** Not Highlight

compounds (56798); glycerolipids (56488); prenol lipids (44606); benzene and substituted derivatives (35629); sphingolipids (29282); steroids and steroid derivatives (235); organonitrogen compounds (23021); steroids and steroid derivatives (217); peptidomimetics (1710); phenols (782); macrolides and analogues (68); indoles and derivatives (697); macrolides and analogues (69); organic sulfuric acids and derivatives (685); diazines (58); purine nucleosides (52); and 179185 other "classes" with 469 or fewer annotated metabolites. A total of 26978 metabolites could not be classified to a "class" level.

The most common" "subclass" classification was for amino acids, peptides, and analogues (1,711681) followed by triradylcglycerols (45034); carbohydrates and carbohydrate conjugates (37497); glycerophosphocholines (3340); glycerophosphoethanolamines (28295); fatty acids and conjugates (25326); fatty acid esters (15042); glycosphingolipids (1431); fatty amides (1401); amines (122); fatty alcohols (13320); fatty acyl glycosides (97); amines (131); ceramides (96103); fatty acyl glycosides (101); linoleic acids and derivatives depsipeptides (9923); lineolic acids and derivatives (87) and 33843 other subclasses with 8293 or less annotated metabolites. A total of 83873 compounds could not be classified to a "subclass" level. The top 12 most common superclasses, classes and subclasses that have been annotated are shown in **Figure 54C-E.**

We note that whilst the *Daphnia* were starved of their usual algae food source for 48 hours prior to freezing for sampling, we still observe annotations for compound superclasses that are primarily derived from plant origin (e.g. the 329 phenylpropanoids and polyketides and the 39 alkaloids and derivatives). Whilst some of these metabolites could potentially be endogenous in *Daphnia* (e.g. biosynthesis within the animal kingdom is known [41]), a more plausible explanation may be that the gut of the *Daphnia* was not entirely free of algae (and any associated microbiome), and the plant-specific metabolites were still being metabolised. Another possibility is that these are potentially false positive annotations, where the actual endogenous *Daphnia* metabolite is structurally similar enough to the plant derived metabolite annotation. The use of reference standards would be required to definitively rule this out.

We note that some of the metabolites annotated here are likely to originate from organisms other than *D. magna*. These may include bacteria (exogenous and microbiota), fungi or parasites that were potentially present in the non-axenic cultures used (despite daily visual inspections to identify and remove cultures with potential contamination), as well as residual algal feed material in the digestive tracts of the *D. magna*. Indeed, some metabolites were assigned compound superclasses typically associated with the plant kingdom, e.g. 306 phenylpropanoids and polyketides and 32 alkaloids and derivatives, though biosynthesis within the animal kingdom is known [41] and some of these annotations could potentially be 'false positive' assignments. Metabolic flux analyses, or radio-labelled isotope tracer experiments, would likely be required to definitively determine the exact origins of each metabolite reported. Alternatively, sterile culturing conditions coupled with the use of dextran beads to purge algal feed material from the digestive tracts of *Daphnia*, may prove helpful in focussing future DMA-like experiments on metabolites that are exclusively present in and/or produced by *D. magna*.

(U)HPLC-HRMS(/MS) and DI-HRMS(/MS$^n$) annotations were predominantly derived from spectral matching, MetFrag and SIRIUS CSI:FingerID annotation approaches. SIRIUS CSI:FingerID produced the most unique annotations with MetFrag and spectral matching

**Formatted:** Not Highlight
**Formatted:** Not Highlight
**Formatted:** Not Highlight
**Formatted:** Not Highlight
**Formatted:** Not Highlight
**Formatted:** Not Highlight
**Formatted:** Not Highlight
**Formatted:** Not Highlight
**Formatted:** Not Highlight
**Formatted:** Not Highlight
**Formatted:** Not Highlight
**Formatted:** Not Highlight
**Formatted:** Not Highlight
**Formatted:** Not Highlight
**Formatted:** Not Highlight
**Formatted:** Not Highlight
**Formatted:** Not Highlight
**Formatted:** Not Highlight
**Formatted:** Not Highlight
**Formatted:** Not Highlight
**Formatted:** Not Highlight
**Formatted:** Not Highlight
**Formatted:** Not Highlight
**Formatted:** Not Highlight
**Formatted:** Not Highlight
**Formatted:** Not Highlight
**Formatted:** Not Highlight
**Formatted:** Not Highlight
**Formatted:** Not Highlight
**Formatted:** Font: Italic

having similar counts of unique annotations (see **Supplemental Section 2.3 and Supplemental Figure S28** for further details). This finding should not be interpreted as identifying the most effective metabolite annotation method, as the number of annotations observed can change dramatically based on filtering criteria and the compound and spectral libraries used. Rather, this simply illustrates the origins of the annotations in this *D. magna* DMA project. The differing results between the annotation approaches does however caution against relying on only a single method, both in terms of the breadth of coverage of the tool used and the potential reliability of annotations.

By using a range of metabolite annotation approaches, the confidence in the resulting annotations can be adjusted to user preference. For example, if only annotations reported by either MetFrag or spectral matching are considered (i.e., disregarding annotations derived only from SIRIUS CSI:FingerID that may yield more false positives amongst the very large number of annotations reported), it would result in 3,591 ~~3,883~~ annotations (or 3,601 ~~3,893~~ if the 1D- & 2D-NMR and GC-EI-HRMS annotations are included). Alternatively, annotations can be filtered even more strictly by specifying that they should be observed with at least two of the three fragmentation data analysis approaches, resulting in 1,286 ~~1,350~~ annotations (or 1,301 ~~1,365~~ if all 1D- & 2D-NMR and GC-EI-HRMS annotations are also included). These subsets of annotations could be considered more reliable, though it is important to highlight that the full set of 8,181 ~~8,577~~ annotations observed from all ~~fragmentation based~~fragmentation-based annotation approaches were derived from sufficiently unique fragmentation spectra to derive this high number of unique metabolite annotations. While some annotations may be less reliable (i.e., from SIRIUS CSI:FingerID only), the number of unique fragmentation spectra demonstrates the richness of the *D. magna* metabolome.

Additionally, de novo molecular formula annotation using MSnPy was performed on the DI-HRMS(/$MS^n$) data via the MSnPy spectral annotation functionality within the Galaxy workflow. The DI-HRMS(/$MS^n$) data are particularly suited to this approach due to the high level of measurement replication at different collision energies and fragmentation levels. A total of 40,240 unique molecular formulae were annotated to an MSnPy rank of 1 (32,672 observed~~derived~~ from positive ionisation mode and 9,768 from negative mode). Ranking was based on the application of common “consistency” rules for filtering formulae and neutral losses [42], which use fragmentation tree consistency to evaluate and rank the most plausible molecular formulae. Only molecular formulae were included where there were 10 or fewer possible top-ranked candidates. It should be noted that multiple molecular formulae can share a rank of one and that, despite stringent filtering, false positives will be present. It is also worth noting that this stringent filtering will have excluded many annotations for higher-mass precursor ions, which tended to produce excessively large sets of candidate molecular formulae, thereby preventing a high number of false-positive annotations. This approach does not rely on prior knowledge of spectral libraries or compound databases and therefore provides a potentially less biased insight into the biochemistry of the metabolome, albeit limited to the level of molecular formula (i.e., non-structural) annotation.

**Formatted:** Not Highlight

**Formatted:** Space Before: 12 pt, After: 12 pt

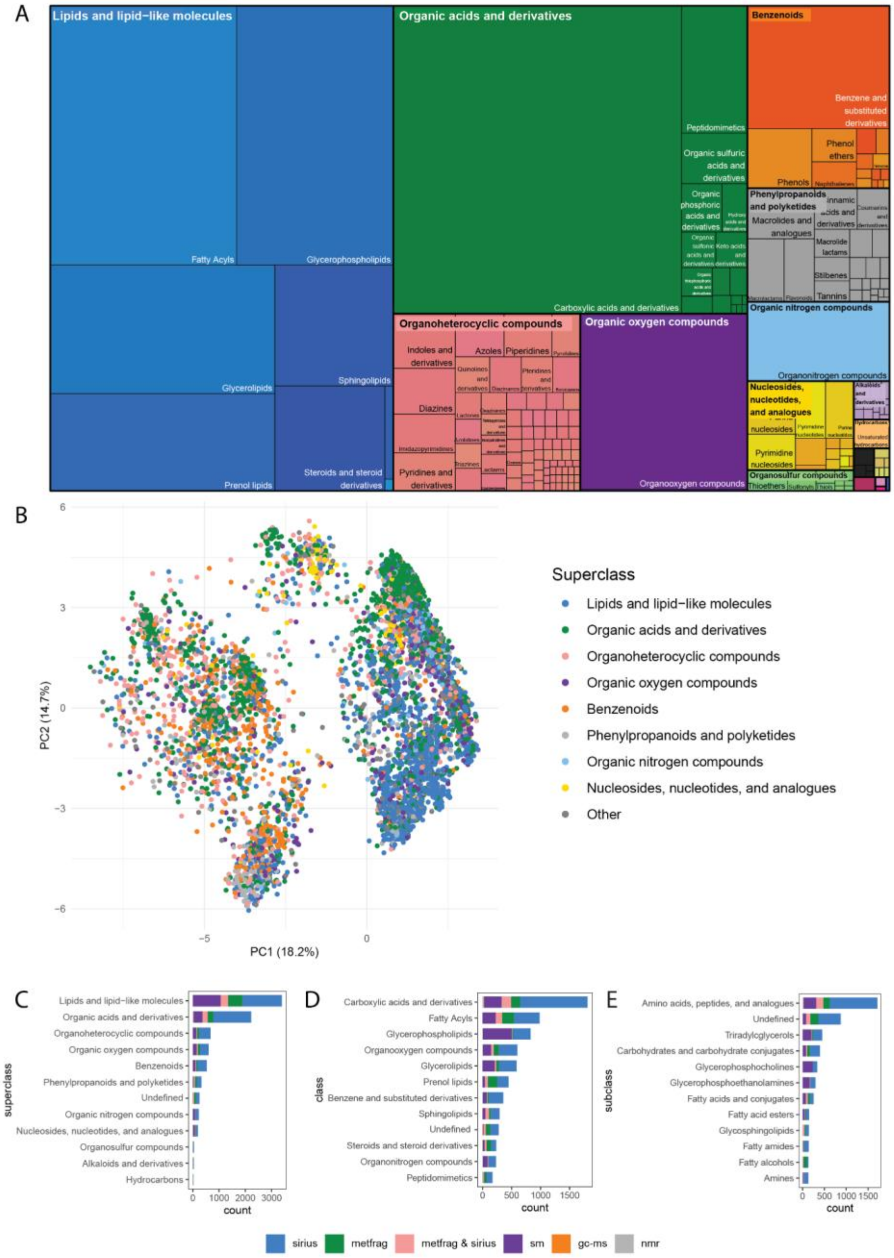

A
Lipids and lipid−like molecules
Fatty Acyls
Glycerophospholipids
Glycerolipids
Sphingolipids
Prenol lipids
Steroids and steroid derivatives
Organic acids and derivatives
Peptidomimetics
Organic sulfuric acids and derivatives
Carboxylic acids and derivatives
Organoheterocyclic compounds
Indoles and derivatives
Azoles
Piperidines
Diazines
Pyridines and derivatives
Organic oxygen compounds
Organooxygen compounds
Benzenoids
Benzene and substituted derivatives
Phenols
Phenol ethers
Phenylpropanoids and polyketides
Macrolides and analogues
Macrolide lactams
Stilbenes
Tannins
Organic nitrogen compounds
Organonitrogen compounds
Nucleosides, nucleotides, and analogues
Pyrimidine nucleosides
Organosulfur compounds
Thioethers
B
PC1 (18.2%)
PC2 (14.7%)
Superclass
Lipids and lipid−like molecules
Organic acids and derivatives
Organoheterocyclic compounds
Organic oxygen compounds
Benzenoids
Phenylpropanoids and polyketides
Organic nitrogen compounds
Nucleosides, nucleotides, and analogues
Other
C
superclass
Lipids and lipid−like molecules
Organic acids and derivatives
Organoheterocyclic compounds
Organic oxygen compounds
Benzenoids
Phenylpropanoids and polyketides
Undefined
Organic nitrogen compounds
Nucleosides, nucleotides, and analogues
Organosulfur compounds
Alkaloids and derivatives
Hydrocarbons
count
D
class
Carboxylic acids and derivatives
Fatty Acyls
Glycerophospholipids
Organooxygen compounds
Glycerolipids
Prenol lipids
Benzene and substituted derivatives
Sphingolipids
Undefined
Steroids and steroid derivatives
Organonitrogen compounds
Peptidomimetics
count
E
subclass
Amino acids, peptides, and analogues
Undefined
Triradylcglycerols
Carbohydrates and carbohydrate conjugates
Glycerophosphocholines
Glycerophosphoethanolamines
Fatty acids and conjugates
Fatty acid esters
Glycosphingolipids
Fatty amides
Fatty alcohols
Amines
count
sirius
metfrag
metfrag & sirius
sm
gc-ms
nmr

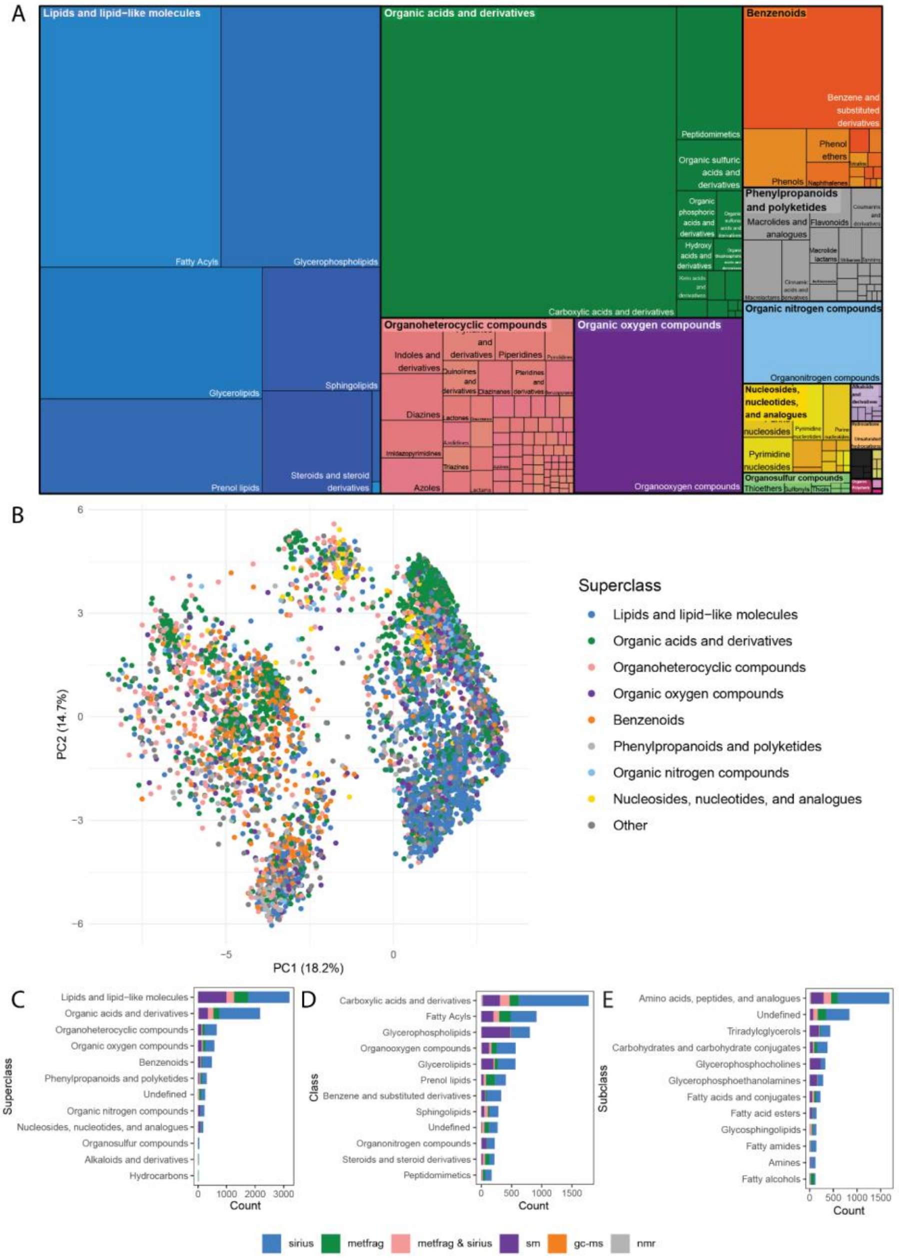


***Figure 54*: Compound classifications for the 8,1818,577 *D. magna* deeply annotated metabolites. A)** *Treemap of metabolite 'superclasses' and 'classes' for the metabolite annotations.* ***B)*** *Principal component analysis of PubChem fingerprints for all unique metabolite annotations: colour represents the superclass*

*classification of the metabolite annotation.* ***C)*** *Count of unique metabolite annotations for 'superclasssubclass' compound classifications. Showing the top 12 'superclassessubclasses'****,*** *colour represents the annotation approach used (annotation was either derived using SIRIUS CSI:FingerID, MetFrag, SIRIUS CSI:FingerID & MetFrag, Spectral matching, GC-EI-HRMS or 1D- & 2D-NMR).* ***D)*** *Count of unique metabolite annotations for 'class' compound classifications. Showing the top 12 'classes'****,*** *colour represents the annotation approach used (annotation was either derived using SIRIUS CSI:FingerID, MetFrag, SIRIUS CSI:FingerID & MetFrag, Spectral matching, GC-EI-HRMS or 1D- & 2D-NMR).* ***E)*** *Count of unique metabolite annotations for 'subsuperclass' compound classifications. Showing the top 12 'subperclasses', colour represents the annotation approach used (annotation was either derived using from SIRIUS CSI:FingerID, MetFrag, SIRIUS CSI:FingerID & MetFrag, Spectral matching, GC-EI-HRMS or 1D- & 2D-NMR).*

*ALT TEXT: Graphs summarising the different compound classifications of metabolites annotated through deep metabolome annotation of D. magna*

### 3.3 Metabolites and compound classes physicochemically separated by DMA experimental workflow

The extent to which the DMA experimental workflow physicochemically separated metabolites was evaluated to determine the effectiveness, or potential redundancy, of components within the workflow (see **Figure 6~~5~~**).

When combining all annotations from either the polar or apolar arm of the workflow (see **Figure 6~~5~~A**), both arms generated a substantial number of unique metabolite annotations (with 4,305~~495~~ and 3,503~~632~~ metabolites unique to the polar and apolar arms respectively, and only 373~~450~~ metabolites shared). This finding highlights the importance of the extraction procedure within the workflow and the necessity of both arms of the workflow to provide a comprehensive view of the metabolome. When combining all annotations from each chromatography approach used (see **Figure ~~5~~6B**), the C30 column yielded the highest number of metabolite annotations (3,876~~4,082~~ in total, of which ~~3,632~~3,503 were unique to the column). The PHE analysis resulted in 3,590~~3,771~~ metabolite annotations (2,404~~66~~ unique to column) while analyses performed using the AMD column resulted in the lowest number of metabolite annotations (~~2,226~~2,049, of which ~~1,171~~1,029 were unique to this column).

Considering the ionisation modes used (see **Figure ~~5~~6C**), analysis using positive ionisation mode ~~produced 6,~~produced ~~099~~5,846 metabolite annotations (~~5,078~~4,902 were unique to this mode), compared to ~~3,490~~ 3,270 metabolite annotations using negative ionisation (2,326 ~~2,469 were~~ unique to this mode). This finding evidences the need to include both ionisation modes in the DMA workflow. Unsurprisingly, for both ionisation modes the higher mass ranges (>600 Da) are dominated by lipids and lipid-like molecules, whereas for mass ranges <600 Da the organic acids and derivatives are most prominent, along with lipids and lipid-like molecules. Other superclasses with mass <600 Da include organoheterocyclic compounds; organic oxygen compounds; benzenoids; phenylpropanoids and polyketides; organic nitrogen compounds; and nucleosides, nucleotides, and analogues. **Supplemental Figure S29** shows the distributions of unique annotations against the exact mass of the annotation.

When examining each assay (see **Figure ~~5~~6D**), in all cases the analysis of the crude *D. magna* extract (without SPE fractionation) resulted in the highest number of annotations compared to the individual SPE fractions. This is to be expected as the SPE fractions were intended to separate the metabolites according to their physicochemical properties and so by design will separate the metabolites across fractions. **Figure ~~5~~6D** also demonstrates that lipids and lipid-like molecules can be seen to dominate the apolar arm, whereas organic acids and derivatives are the most prominent in the polar arm. This is also expected based on the chemistry of the liquid-phase extractions, solid-phase extractions and chromatography used, which favour apolar metabolites.

**Figure ~~5~~6E** shows an UpSet plot detailing the overlap of annotations across assays (with positive and negative ionisation modes combined). The most striking observation is the dissimilar number of annotations per assay, with the SPE fractions from the AMD column providing the lowest number of annotations. This assessment could form a basis for a more streamlined, time-efficient workflow. Given that the measurements of the crude extracts yielded 581, 568 and 513~~577, 565 and 507~~ unique annotations (for apolar crude C30, polar

**Formatted:** Not Highlight
**Formatted:** Not Highlight
**Formatted:** Not Highlight
**Formatted:** Not Highlight
**Formatted:** Not Highlight
**Formatted:** Not Highlight
**Formatted:** Not Highlight

crude AMD and polar crude PHE respectively) illustrates their valuable contribution to the DMA workflow.

To provide added confidence in the ability of the experimental and computational workflow's ability to annotate the metabolome an assessment of the workflow was also made using 48 chemical reference standards covering a wide biochemical space including lipid and lipid-like molecules; organic acids and derivatives; organic oxygen compounds; organoheterocyclic compounds; nucleosides, nucleotides, and analogues; and organic nitrogen compounds. The spread of the compound classes across the experimental workflow mirrors what is observed in the *Daphnia* samples, where lipids and lipid-like molecules dominate the apolar arm and organic acids and derivatives being the most prominent in the polar arm. As the majority of metabolite reference standards were observed by the DMA workflow (89.6%), we deemed that both the experimental and computational workflows were sufficiently reliable at annotating a diverse range of metabolites to be used for annotating *D. magna* metabolome. See **Supplemental Table S11**, **Supplemental Figure S30-31** and **Supplemental Section 2.6** for further details.

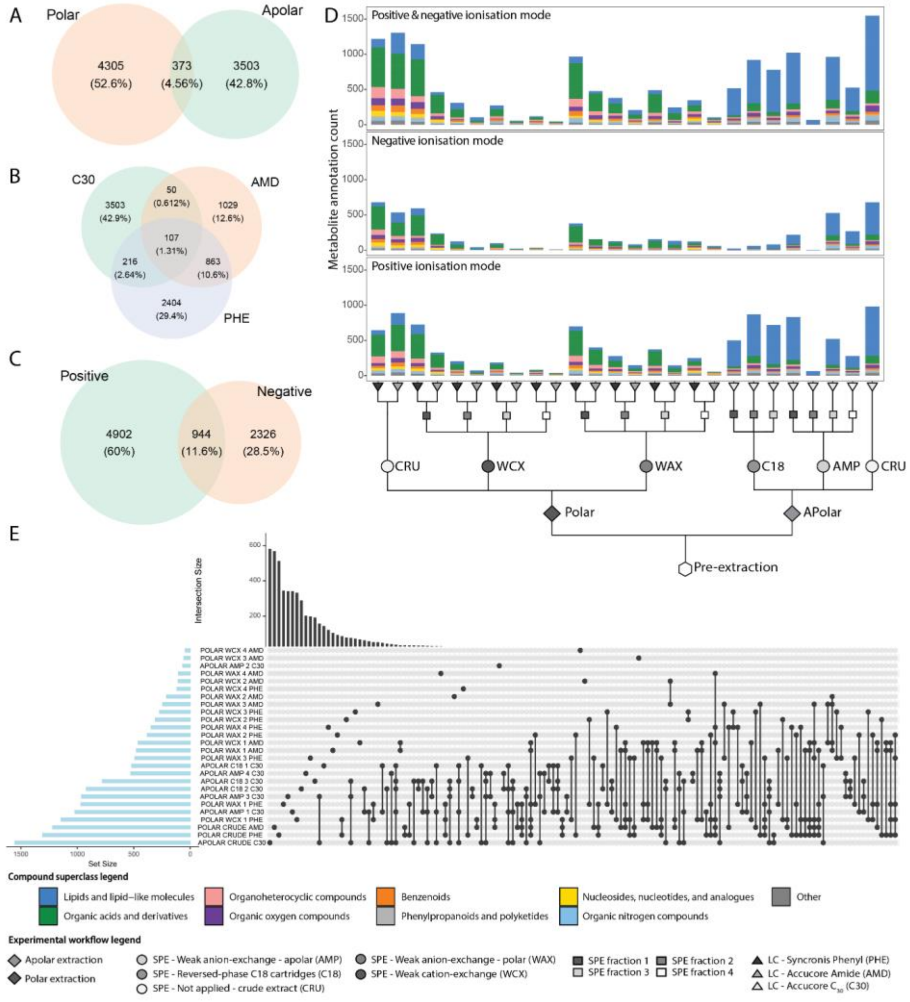


***Figure ~~5~~6: Contributions of extraction, SPE fractionation, LC separation and mass spectrometric ionisation methods to the number of metabolite annotations for D. magna.*** *A~~C~~) Venn diagram of metabolite annotations observed across extraction approaches. B~~D~~) Venn diagram of metabolite annotations observed across all chromatography techniques. C~~E~~) Venn diagram of metabolite annotations observed across positive and negative ionisation modes.*

*~~A~~D) Count of metabolite annotations across experimental workflow components: bar charts shown for positive ionisation mode, negative ionisation mode and combined positive and negative ionisation modes. Colour represents the superclass compound classification of the annotations. See bottom of figure for colour code used for compound superclass. ~~B~~E) UpSet plot summarising the overlap of metabolite annotations between assays (positive and negative assays have been combined). ~~C) Venn diagram of metabolite annotations observed across extraction approaches. D) Venn diagram of metabolite annotations observed across all chromatography techniques. E) Venn diagram of metabolite annotations observed across positive and negative ionisation modes.~~*

*ALT TEXT: Graphs summarising the number of metabolite annotations resulting from each experimental component of the D. magna deep metabolome annotation.*

## 3.4 Comparison to other metabolite databases

The annotation results from the DMA of *D. magna* were compared to public resources of relevant metabolites from different species, as summarised in **Figure 77**. Even considering this relatively limited number of metabolites known for different organisms, it is readily apparent that some metabolites are widely shared across organisms due to the conservation of metabolism (i.e., phylometabolomics). From ChEBI, the top six species that overlap with *D. magna* DMA annotations are *Homo sapiens*, *Saccharomyces cerevisiae*, *Mus musculus, Escherichia coli, D. magna* and *Chlamydomonas reinhardtii (a single celled green algae)*. For *H. sapiens, M. musculus*, *S. cerevisiae* and *E. coli* this is in part explained by these species being the most represented within ChEBI. However, overlap with the previously known *D. magna* and algae metabolites (which are the diet of the cultured *D. magna*) adds confidence to both the existing annotations and the effectiveness of the DMA workflow.

As *D. magna* is used internationally as an ecotoxicology test species, the metabolite annotations reported using the DMA workflow were compared to those in MTox700+, a metabolite list of toxicologically-relevant metabolites derived from mammalian studies. Overlapping metabolites could be used to help interpret the toxicological perturbations measured in *D. magna* metabolomics studies. A total of 311293 of 722 metabolites were matched to a full InChIiKey (or 370346 of 722 if using the first section of the InChiIKey).

In addition to the above, the 8,5778,181 annotated metabolites were investigated to assess coverage of known pathways. Whilst we acknowledge the limitations of this analysis due to the limited knowledge of pathways for *Daphnia*, preliminary analysis using the QIAGEN IPA software were still able to identify 56 molecular pathways ($p \leq 0.05$, Fisher's exact test), with 11345 pathways having ≥ 50% coverage over 50% with ≥ 3 measured metabolites (see **Supplemental Figure S32**), supporting the relevance of the annotated metabolites for pathway-level toxicological interpretation.suggesting new knowledge of the *Daphnia* metabolome may enable deeper toxicological insights.

**Formatted:** Not Highlight

**Formatted:** Not Highlight

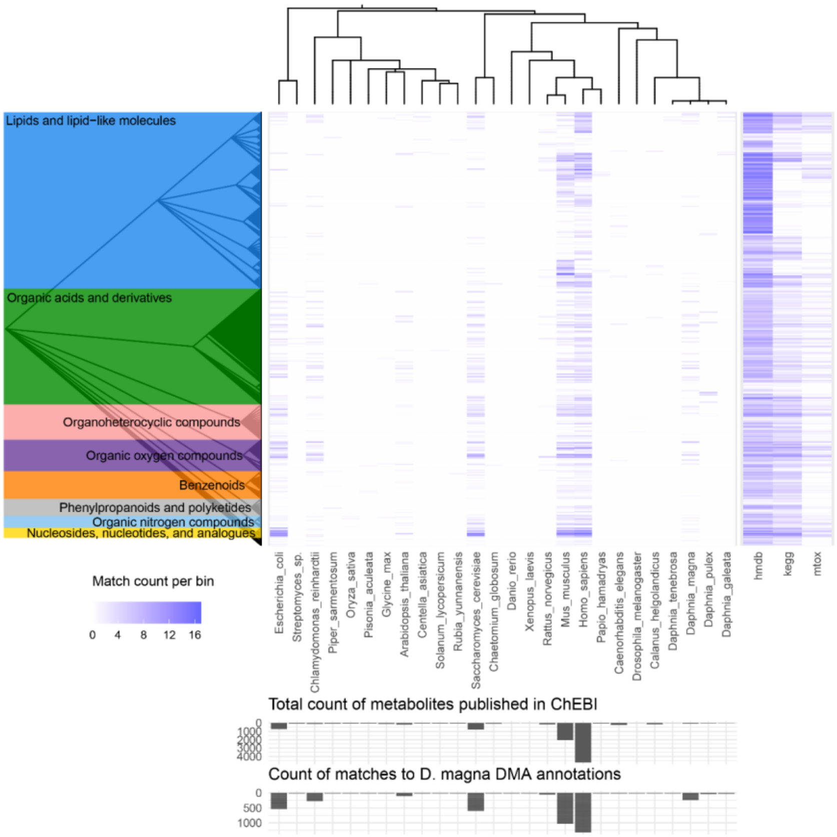

Lipids and lipid−like molecules
Organic acids and derivatives
Organoheterocyclic compounds
Organic oxygen compounds
Benzenoids
Phenylpropanoids and polyketides
Organic nitrogen compounds
Nucleosides, nucleotides, and analogues
Match count per bin
0 4 8 12 16
Escherichia_coli
Streptomyces_sp.
Chlamydomonas_reinhardtii
Piper_sarmentosum
Oryza_sativa
Pisonia_aculeata
Glycine_max
Arabidopsis_thaliana
Centella_asiatica
Solanum_lycopersicum
Rubia_yunnanensis
Saccharomyces_cerevisiae
Chaetomium_globosum
Danio_rerio
Xenopus_laevis
Rattus_norvegicus
Mus_musculus
Homo_sapiens
Papio_hamadryas
Caenorhabditis_elegans
Drosophila_melanogaster
Calanus_helgolandicus
Daphnia_tenebrosa
Daphnia_magna
Daphnia_pulex
Daphnia_galeata
hmdb
kegg
mtox
Total count of metabolites published in ChEBI
0
1000
2000
3000
4000
Count of matches to D. magna DMA annotations
0
500
1000

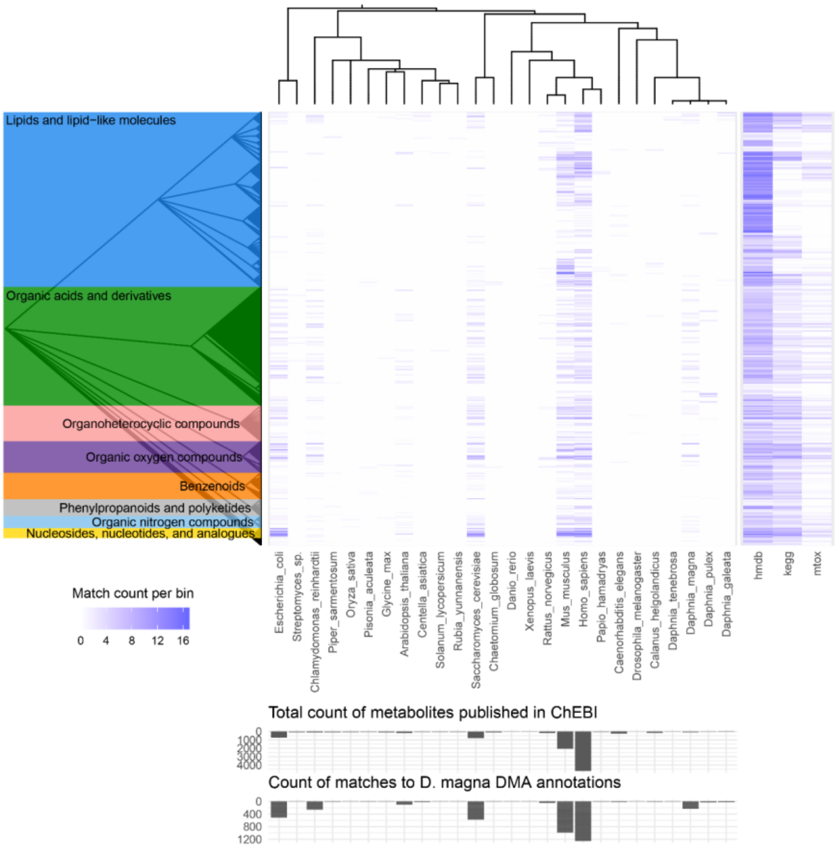


***Figure 76: Overlap of D. magna metabolite annotations reported using the DMA workflow with known metabolites from other species. Left** – all metabolite annotations from the DMA of D. magna presented in a hierarchical tree of superclasses, classes and subclasses. **Top** – phylogenetic tree of known metabolites from 26 species derived from ChEBI. **Centre** – heatmap of the counts of metabolites matched between the species derived from ChEBI with the D. magna metabolites from the DMA workflow (binned into sets of 25 compounds). **Right –** heatmap of matches observed between HMDB, KEGG and MTox700+ and the D. magna metabolite annotations reported using the DMA workflow. **Bottom –** counts of the metabolites published with ChEBI for each organism and below that the counts of the matches to DMA of D. Magna annotations.*

*ALT TEXT: Graphs showing how the D. magna deep metabolome annotations overlap across other species from known metabolomes.*

## 3.5 Molecular network analysis using GNPS

Molecular networks were generated using the GNPS network analysis workflow, with classical molecular networking, MS2LDA, Dereplicator+ and MolNetEnhancer (see **Figure 78** for summary of negative ionisation molecular networks and **Supplemental Figure S33** for positive ionisation molecular networks). These networks provide an overall picture of the diversity of the fragmentation spectra collected (and thus the diversity of metabolites observed in *Daphnia*) while not being wholly dependent on obtaining a compound or compound class annotation. The dataset collected exhibited a large diversity of fragmentation spectra (ca. 31,000 fragmentation clusters for the positive ionisation and ca. 5,000 distinct fragmentation clusters for negative ionisation mode). A similar overview can be achieved using MS2LDA mass-motifs, for which ca. 55,000 “mass motifs” are observed for positive ionisation data and ca. 3,900 for the negative ionisation data. However, only a small subset of the clusters could be annotated (e.g., ca. 1,000 clusters annotated via spectral matching for positive ionisation spectra and ca. 200 clusters annotated for negative ionisation spectra). The remaining unannotated spectra may reveal additional insights into the *Daphnia* metabolome as spectral libraries and computational approaches for annotation improve. In particular, the annotations could be improved with further integration with MetFrag and SIRIUSirius CSI:FingerID, but this was beyond the scope of this analysis. The spectral networks also demonstrate the potential for future analysis where multiple networks created from other model organism DMA projects could be compared for cross-species phylometabolomics analysis that would be driven by spectral similarity as opposed to being reliant on metabolite annotations.

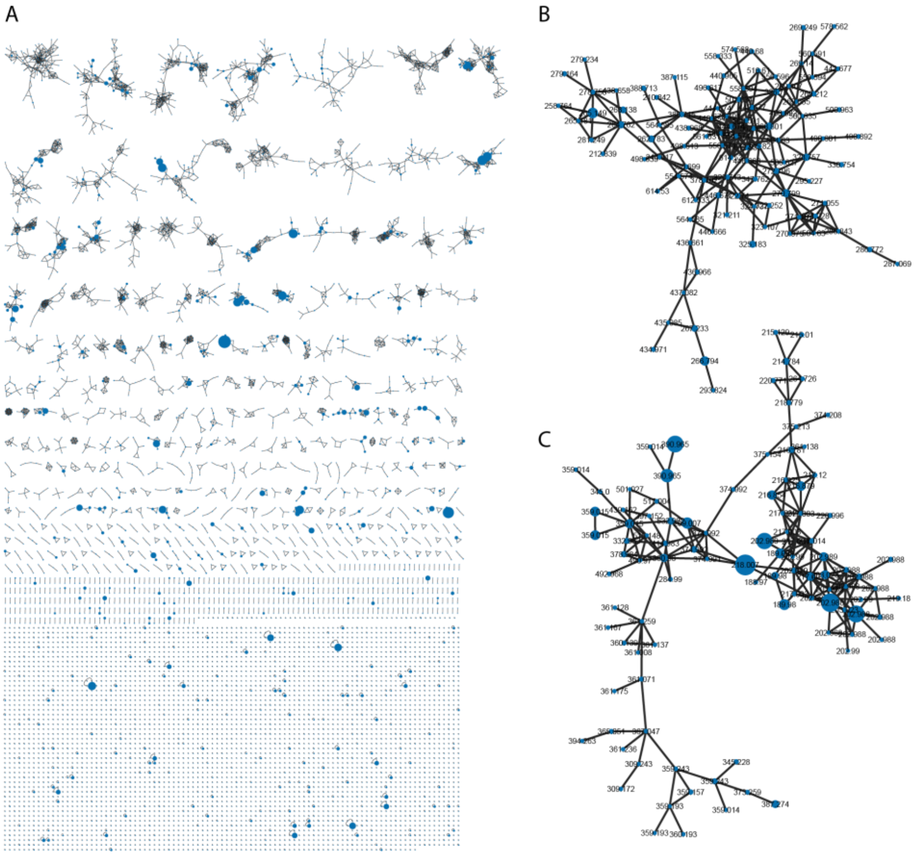


***Figure 8~~7~~*. GNPS molecular network derived from D. magna deep metabolome annotation negative ionisation data. A)** Overview of negative ionisation mode molecular networks generated from GNPS molecular network analysis, showing all 5,320 distinct clusters. The node size is proportional to the number of spectra that contribute to the node. The top 2 clusters (based on how many nodes were observed) are shown in more detail to highlight the precursor *m/z* associated with the node. **B)** Largest cluster observed. **C)** Second largest cluster observed.

*ALT TEXT: Networks of the negative ionisation mass spectrometry fragmentation data for the D.magna deep metabolome annotations.*

## 3.6 Deep metabolome annotation database (DMAdb)

## 3.6 DMAdb: Public Access and Functionalities

DMAdb serves as a data management environment encompassing raw data, processing workflows, and metabolite annotations (Figure 4). This integrated framework enhances reproducibility and supports transparent data provenance. By structuring DMA data according to the ISA (Investigation–Study–Assay) model, DMAdb provides standardised representations of experimental datasets, thereby promoting interoperability and enabling structured exploration of associated metadata. Public users can access selected functionalities without registration (Figure 4), including browsing ISA-organised datasets and performing basic searches of metabolite annotation records. Registered users are provided with extended functionalities, including access to processed datasets at the individual assay level, derived from the corresponding Galaxy histories. Advanced functionalities futherfurther include batch-based exact mass searches across annotated metabolites and MS/MS spectral matching against processed and annotated fragmentation spectra. Although currently implemented in a focused developmental form, DMAdb establishes a structured foundation for ongoing refinement and future expansion. While initially developed to support systematic investigation of the metabolic complexity of *Daphnia magna*, it offers a framework that can be adapted for use with additional model organisms.Although developed specifically for this study to support systematic investigation of the metabolic complexity of *D. magna*, DMAdb establishes a structured foundation for ongoing refinement and future expansion, offering a framework that can be adapted for use with additional model organisms.

**Formatted:** Font: Italic

As a means to internally organise and process the DMA annotations, and to serve as a proof of concept for how deep metabolome annotations could be disseminated in an interactive manner, DMAdb was developed. Accessible via https://dmadb.bham.ac.uk, users can access the data and results through the "ISA (investigation, study & assay)" section or the "Data & Results" section. The (U)HPLC-HRMS(/MS) and DI-HRMS(/MS$^n$) mass spectrometry raw data are available to download, the metabolite annotations for each assay can be explored, the overall metabolite annotations across all assays are summarised, and functionality is provided to both search by monoisotopic exact mass across the annotated compounds and to search the fragmentation spectra from all assays.

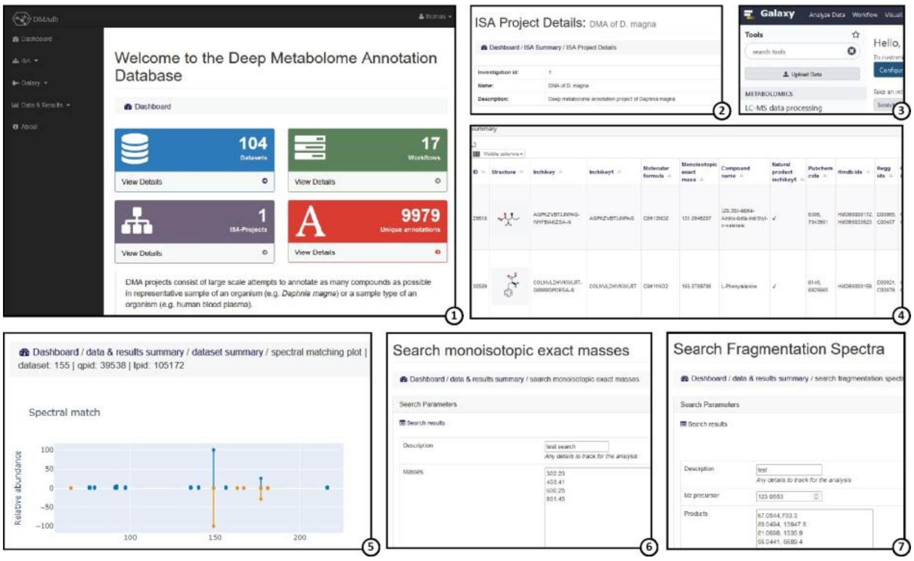


***Figure 8 The Deep Metabolome Annotation database (DMAdb) - overview of its web interface and functionalities.*** *1) Dashboard for the DMAdb. 2) Multiple projects can be handled within the (Investigation/Study/Assay) ISA framework where relevant ontologies, protocols and processes can be recorded. 3) Data processing and annotation analysis was performed via the Galaxy platform (i.e., tools and workflows) and the resulting outputs were uploaded and stored in DMAdb. 4) The metabolite annotations and raw data files can be browsed (4), visualised (5), and searched either by one or more monoisotopic exact masses (6) or via fragmentation spectra (7).*

*ALT TEXT: Summary of the different web interfaces of the Deep Metabolome Annotation database (DMAdb).*

# 4 Conclusion

The extensive experimental and computational tools and workflows developed and applied here have generated one of the largest metabolite annotation datasets ever published and provide the first comprehensive list of metabolites thought to be present in the ecotoxicologically important model organism, *D. magna*. The reported 8,5778,181 metabolites (1,365 to 3,8931,301 to 3,601 if using more stringent filtering criteria), covering predominantly the endogenous *Daphnia* metabolites and potentially some amount of the algae food source and gut microbiome (e.g. the 329306 phenylpropanoids and polyketides and the 3932 alkaloids and derivatives*)*, is a significant step forward in understanding the metabolic complexity of *Daphnia*.

-The dataset generated here also provides a large resource of mass spectrometry fragmentation data focused on a single organism, which can be used for both re-analysis and as a source of annotations when compared to other fragmentation datasets, e.g. for cross-species metabolome comparisons (phylometabolomics). Additionally, we have established Galaxy workflows and tools to process and annotate not just this dataset, but other mass spectrometry fragmentation datasets. Indeed, as many of the tools have already been made public, the benefit of the Galaxy tools developed here have already been demonstrated [43], [44]. R-eproducing the complex, multi-step workflow within Galaxy would require substantial effort, particularly for users unfamiliar with the workflow management platform or with the specific configuration requirements of this study. However, our intention in sharing the full Galaxy histories and workflows is to provide transparency and enable methodological inspection and reuse where appropriate. We also note that the version of SIRIUS CSI:FingerID used here (v4) is no longer readily publicly accessible, as it relied on an API endpoint that is no longer supported. Despite these caveats, the Galaxy tools developed here have already been applied in subsequent metabolomics studies, demonstrating their practical utility [43], [44].

We also present DMAdb as a data management environment for raw data, processing steps, and metabolite annotations, providing controlled public access to ISA-organised datasets alongside extended functionalities for registered users. Although developed specifically for this study, it establishes a potential foundation for the organisation and exploration of future DMA datasets, with application beyond *D. magna.*

While our computational workflow is extensive, encompassing multiple techniques and approaches, we do acknowledge it does have someseveral limitationschallenges. In particular, the bias arisinges from the choice of software, software versions, software parameters settings, weightings, library choice and filtering approaches used, all of which influence the final set of reported metabolite annotations. and their impact on the final set of reported metabolite annotations . The degree of lenience lenience setused for thesese software parameters and choices does impacts the reliability of the annotations, and it is anticipated that some false positive annotations are reported here, i.e., not actual endogenous *Daphnia* metabolites but structurally similar enough to generate an annotation. Purchasing thousands of metabolite reference standards to confirm the annotations is not currently possible, due to the limited commercial availability of such standards. Also, whilst

**Formatted:** Not Highlight

**Formatted:** Not Highlight

steps were put in place to minimise influence of background or contaminant signals, further work could investigate more stringent criteria. As more metabolite annotations are described for model organisms, assuming that they are definitive, the reliability of the annotations from the *Daphnia* DMA project can be tested. Improvements to the computational workflow could include ~~a~~ more extensive use of isotopic~~es~~ patterns and in-source fragmentation to filter out spurious annotations [45]; further integration of the underlying software packages into other mass spectrometry-based data analysis suites [46]; and ~~further~~ integration with GNPS networking tools where networks could be generated from averaged spectra produced via msPurity and MSnPy rather than individual scans, followed by incorporation of annotations from MetFrag and SIRIUS CSI:FingerID. ~~potentially the networks could be generated on the averaged spectra generated via msPurity and MSnPy rather than individual scans, and then incorporating the annotations generated from MetFrag and Sirius CSI:FingerID.~~

The experimental methods described here provide a means to obtain a single homogenous sample representing an organism's metabolome that is then characterised through extensive physicochemical separation and bioanalytical measurements. The experimental workflow developed here could be reused in full or in part, depending on the resources available. For example, where sample material is limited, a subset of the SPE methods and/or (U)HPLC-HRMS(/MS) approaches could be used based on which metabolite classes are of interest, or for a more rapid DMA project the SPE component could be removed from the workflow. We acknowledge that even with this extensive workflow there are limitations in how many metabolites were able to be fragmented, reducing the number of metabolites that can be annotated using fragmentation-based approaches. However, advancements in mass spectrometry technologies can help to address this issue, e.g. the Thermo Scientific Orbitrap ID-X Tribrid mass spectrometer is capable of extensive $MS^n$ analysis, while current generation Time of Flight / Astral instruments support MS/MS acquisition at up to 250 Hz.

It is unlikely that the present study provides an exhaustive list of all possible metabolites produced by or present in *D. magna*. In part, this is due to limitations of current analytical platforms, which typically only detect a subset of an organism's metabolome, e.g. pigment molecules are often difficult to detect using liquid chromatography mass spectrometry. At the same time, while ten *D. magna* strains were included in the present work to maximise the breadth of *D. magna* metabolome annotation achieved, and to capture genetic diversity across the species, many of these strains are derived from European *Daphnia* populations. *D. magna* strains from disparate areas of the globe, such as East Asian, South Africa or North America, may very well produce a plethora of unique metabolites that are currently missing from the annotations described here. Thus, the DMA of *D. magna* would ideally now be expanded to include metabolite annotation data, collated using a DMA-like workflow, both for *Daphnia* originating from a wider variety of geographic locations, and for Daphnia maintained under a greater range of environmental or exposure conditions. In so doing, this would be better able to support the applicability of this work to *D. magna* metabolomics studies performed across the globe, while also better facilitating exploration of the genetic and environmental basis of metabolic diversity across *D. magna* populations.

**Formatted:** Font: Italic

**Formatted:** Font: Italic

**Formatted:** Font: Italic

**Formatted:** Font: Italic

**Formatted:** Font: Italic

**Formatted:** Font: Italic

**Formatted:** Font: Italic

**Formatted:** Font: Italic

**Formatted:** Font: Italic

Even with theese limitations described above, the DMA workflow described here and applied to *D. magna* provides both a resource and a valuable catalyst for future deep metabolome annotation studies of other model organisms.

# 5 Code availability

The Galaxy workflows, histories and details of each tool used in this project are available in https://dma.galaxy.bham.ac.uk/histories/list_published and https://dma.galaxy.bham.ac.uk/workflows/list_published. The Galaxy analysis performed used the following workflows: ~~(workflows also available to download via - https://github.com/computational-metabolomics/dmagna-dma-galaxy-workflows under GPL-3.0 licence).~~

- **W1**: The full (U)HPLC-HRMS(/MS), DI-HRMS(/MS$^n$) and LC fractionation workflow (described in **Figure 3**)
- **W2**: The (U)HPLC-HRMS(/MS) only workflow
- **W3**: The (U)HPLC-HRMS(/MS) workflow used for the metabolite reference standard analysis.

**Supplemental Table S1** provides the links for the relevant histories and workflow used for each assay.

The workflows are also made available on Workflowhub (W1 - https://workflowhub.eu/workflows/2084; W2 - https://workflowhub.eu/workflows/2085; and W3 - https://workflowhub.eu/workflows/2086)

These above resources cover the full Galaxy workflow analysis; however, re-running all analyses, particularly outside of the provided Galaxy instances, would require additional setup due to both the high computational resource demands of this large dataset, as well as software updates to some of the underlying Galaxy tool since the analysis in this manuscript was performed. As such, we also include an example workflow for the most broadly reusable portion of the (U)HPLC-HRMS(/MS) workflow (https://workflowhub.eu/workflows/2083), that can be executed on other public instances (e.g. Workflow4Metabolomics - https://workflow4metabolomics.usegalaxy.fr/).

Workflows are also available to download via - https://github.com/computational-metabolomics/dmagna-dma-galaxy-workflows under GPL-3.0 licence.

See **Table 1** for availability of the Galaxy tools used and developed, and **Supplemental Table S9** for details of each tool.

**Formatted:** Space After: 6 pt

**Formatted:** Font: Bold

**Formatted:** Superscript

**Formatted:** Font: Bold

**Formatted:** Font: Bold

**Formatted:** Font: Bold

**Formatted:** Normal, Space After: 6 pt, No bullets or numbering

**Formatted:** Space After: 6 pt

**Formatted:** Space After: 6 pt

**Table 1:** Galaxy tools - code availability

| **Project name (biotools id)** | **Galaxy tools** | **Galaxy tool code home page** | **Underlying software code home page** | **Licence** | **Lang** |
|---|---|---|---|---|---|
| Pre-existing software and Galaxy tools | | | | | |
| MSnBase (biotools:msnbase) | MSnBase.readMSData | https://github.com/workflow4metabolomics/tools-metabolomics | https://www.bioconductor.org/packages/release/bioc/html/MSnbase.html | Underlying software: Artistic-2.0<br>Galaxy tool: GPL-3.0 | R |
| XCMS (biotools:xcms) | xcms.findChromPeaks<br>xcms.findChromPeaks Merger<br>xcms.groupChromPeaks | https://github.com/workflow4metabolomics/tools-metabolomics | http://bioconductor.org/packages/release/bioc/html/xcms.html<br><br>[47] | GPL (>= 2) | R |
| CAMERA (biotools:camera) | CAMERA .Annotate | https://github.com/workflow4metabolomics/tools-metabolomics | https://www.bioconductor.org/packages/release/bioc/html/CAMERA.html<br><br>[48] | GPL (>= 2) | R |
| BEAMSpy (Not available) | BEAMSpy | https://github.com/computational-metabolomics/beamspy-galaxy | https://github.com/computational-metabolomics/beamspy<br><br>https://more.bham.ac.uk/beams/ | GPL-3.0 | R |
| DIMSpy (biotools:dimspy) | dimspy.Process scans<br>dimspy.merge peaklists<br>dimspy.align samples<br>dimspy.blank filter<br>dimspy.Get peaklist | https://github.com/computational-metabolomics/dimspy-galaxy | https://github.com/computational-metabolomics/dimspy | GPL-3.0 | Python |
| Software and / or the Galaxy tool was developed by authors | | | | | |
| †**msPurity (biotools:mspurity) | msPurity.purityA<br>msPurity.flagRemove<br>msPurity.frag4feature<br>msPurity.filterFragSpectra<br>msPurity.averageFragSpectra<br>msPurity.createMSP<br>msPurity.createDatabase<br>msPurity.spectralMatching<br>msPurity.combineAnnotations<br>msPurity.dimsPredictPurity | https://github.com/computational-metabolomics/mspurity-galaxy/ | https://www.bioconductor.org/packages/release/bioc/html/msPurity.html<br><br>[26] | GPL-3.0 | R |
| **†MSnPy (biotools:msnpy) | MSnPy.group-scans<br>MSnPy.process-scans<br>MSnPy.create-spectral-trees<br>MSnPy.annotate-trees<br>MSnPy.convert-spectral-trees | https://github.com/computational-metabolomics/msnpy-galaxy | https://github.com/computational-metabolomics/msnpy | GPL-3.0 | Python |
| **†msp2db (Not available) | msp2db | https://github.com/computational-metabolomics/dmatools-galaxy | https://github.com/computational-metabolomics/msp2db | GPL-3.0 | Python |
| †**CAMERA DIMS (Not available) | CAMERA DIMS | https://github.com/computational-metabolomics/dmatools-galaxy | https://github.com/computational-metabolomics/cameraDIMS | GPL (>= 2) | R |

Formatted Table

Formatted: Superscript

| | | | | | |
|---|---|---|---|---|---|
| *SIRIUS CSI:FingerID (biotools:Sirius) | SIRIUS CSI:FingerID | https://github.com/computational-metabolomics/sirius-csifingerid-galaxy/ | https://bio.informatik.uni-jena.de/software/sirius/ <br><br> [30] | Underlying software: GNU AGPL Galaxy tool: GPL-3.0 | Java (and python for Galaxy wrapper) |
| *MetFrag (biotools:metfrag) | MetFrag | https://github.com/computational-metabolomics/metfrag-galaxy/ | https://ipb-halle.github.io/MetFrag/ <br><br> [27], [28], [29] | Underlying software: GPL (>= 2) Galaxy tool: GPL-3.0 | Java (and python for Galaxy wrapper) |
| ~~**~~†LC fractionation processor (Not available) | LC fractionation processor | https://github.com/computational-metabolomics/lcfrac-galaxy | https://github.com/computational-metabolomics/lcfrac-galaxy <br><br> (all functionality within Galaxy tool) | GPL-3.0 | Python |
| ~~**~~†§deconrank (Not available) | deconrank | https://github.com/computational-metabolomics/dmatools-galaxy | https://github.com/computational-metabolomics/deconrank | GPL-3.0 | Python |

**Footnotes**: All tools and software described in table are operating system platform independent. *Galaxy tool developed by authors. †~~**~~New (or updated) underlying software and Galaxy tool developed by authors. §Not used directly in the annotation workflows but was used in the “directed acquisition workflows” described in **Supplemental Section 1.8.** ~~Each of the new tools provided here provide example test data to trial the functionality and analysis.~~The “msp2db”, “CAMERA DIMS”, “LC fractionation processor” and “deconrank” are considered highly specific to this workflow and currently of limited general applicability, and therefore have not yet been registered in bio.tools.

R (v4.4.3) was used for summarising the annotations and generating **Figures 5-7** and the supplemental summary figures. The code used for this is available via Github (https://github.com/computational-metabolomics/dmagna-dma-paper) available under the GPL-3.0 licence – operating system – platform independent. All packages requirements are detailed with the repository and we summarise some of the key packages used here: The R package ggplot2 (v3.5.2) [49] was used throughout the analysis for the generation of plots; UpSetR (v1.4.0) [50] was used to generate UpSet plots; VennDiagram (v1.7.3) was used to generate Venn diagrams; Treemap (v2.4.4) was used to generate treemaps; ggtree (v3.14.0) [51], ape (v5.8.1) [52] and aplot (v0.2.8) [53] were used to generate the plots comparing the DMA of *D. magna* metabolites to the phylogenetic tree of relevant species and map to relevant databases and resources; ChemmineR [54] (v3.58.0) was used to extract the PubChem fingerprints from the PubChem mol files from PubChem [55], principal component analysis (PCA) was then performed on these fingerprints with the R “prcomp” function. Additionally, ~~T~~this repository includes an example demonstrating how data from the Galaxy histories can be accessed programmatically, including downloading the XCMS peak matrices and inspecting their relative peak intensities.

~~The codebase for t~~~~The~~ DMAdb and web portal (https://dmadb.bham.ac.uk) was developed using three Django applications (django-gfiles, django-galaxy and django-mogi) specifically designed for metabolomics data organisation with Galaxy and the ISA- framework. The packages and code used are freely available (Project name: DMAdb; Project home page: https://dmadb.readthedocs.io/en/latest/getting-started.html; Operating system(s): Platform independent; Programming language: Python; License: GPL-3.0).

**Formatted:** Font: 8 pt

**Formatted:** Font: 8 pt

**Formatted:** Font: 8 pt

**Formatted:** Font: 8 pt

**Formatted:** Font: 8 pt

# 6 Additional files

Supplemental information is available in the accompanying Word (.docx) file, with larger tables provided separately in the Excel (.xlsx) file (**Supplemental Tables S1, S2, S11** and **S13**).

**Supplemental section 1: Materials and methods - further details**

- **1.1: Summary of assays and files**
- **1.2: Chemicals**
- **1.3: Solvents and solutions**
- **1.4: Consumables**
- **1.5: *D. magna* culturing and sample preparation**
- **1.6: Metabolite extraction from homogenised D. magna biomass**
- **1.7: Solid phase extraction-based fractionation of metabolite extracts**
- **1.8: DMA (U)HPLC-HRMS(/MS), DI-HRMS(MS$^n$) and LC fractionation**
- **1.9: (U)HPLC-HRMS(/MS) method optimisation**
- **1.10: GC-EI-HRMS**
- **1.11: 1D- & 2D-NMR**
- **1.12: DMA computational workflow overview**
- **1.13: DMA Galaxy workflow**
- **1.14: Combining and summarising all annotations**
- **1.15: Assessment of the computational and experimental DMA workflow with metabolite reference standards**

**Supplemental section 2: Results - further details**

- **2.1: (U)HPLC-HRMS(/MS) method optimisation**
- **2.2: Summary of all DMA of *D. magna* annotations**
- **2.3: (U)HPLC-HRMS(/MS) and DI-HRMS(MS$^n$) derived metabolite annotations**
- **2.4: GC-EI-HRMS derived metabolite annotations**
- **2.5: NMR derived metabolite annotations**
- **2.6: Assessment of the computational and experimental DMA workflow with metabolite reference standards**
- **2.7: Pathway analysis**
- **2.8: Molecular network analysis using GNPS**

**Supplemental Tables:**

- **Table S1:** Assay Summary
- **Table S2:** (U)HPLC-HRMS(/MS) DI-HRMS(MS$^n$) data files
- **Table S3:** *D. magna* ~~cultures~~strains
- **Table S4:** High-hardness COMBO and modified high hardness COMBO medium
- **Table S5:** Bold’s basal medium
- **Table S6:** Liquid chromatography systems utilised in optimisation of (U)HPLC-HRMS(/MS) methods

- **Table S7:** Mass spectrometer operational parameters for optimisation of (U)HPLC-HRMS(/MS) methods
- **Table S8:** Liquid chromatography operational parameters for optimisation of (U)HPLC-HRMS(/MS) methods
- **Table S9:** Summary of Galaxy tools
- **Table S10:** Summary of fragmentation spectra used for spectral matching with msPurity
- **Table S11:** Metabolite reference standard summary
- **Table S12:** Median and interquartile range of retention times for RDMFs recorded in DMA (U)HPLC-HRMS/MS method optimisation experiments
- **Table S13:** *D. magna* metabolite annotation summary
- **Table S14:** GC-EI-HRMS derived metabolite annotations
- **Table S15:** NMR derived metabolite annotations

**Supplemental figures (methods)**

- **Figure S1**: Solid phase extraction-based fractionation of *D. magna* polar extract (WAX, weak anion-exchange; WCX, weak cation-exchange)
- **Figure S2**: Solid phase extraction-based fractionation of *D. magna* apolar extract (C18, a reversed phase-based fractionation procedure; AMP, a weak anion-exchange-based fractionation procedure
- **Figure S3:** Overview of the data acquisition workflow applied for (U)HPLC-HRMS(/MS) analysis and time-based fractionation of DMA samples
- **Figure S4:** Overview of the data acquisition workflow applied for DI-HRMS(/MS$^n$) analysis of the DMA re-suspended LC fractionation samples.
- **Figure S5**: Sample preparation for (U)HPLC-HRMS(/MS) method optimisation
- **Figure S6:** Overview of computational analysis of DMA (U)HPLC-HRMS(/MS) and DI-HRMS(MS$^n$) LC fractionation experiments
- **Figure S7:** (U)HPLC-HRMS(/MS) data processing schematic for msPurity and XCMS
- **Figure S8:** DI-HRMS(MS$^n$) data processing schematic for MSnPy

**Supplemental figures ((U)HPLC-HRMS(/MS) method optimisation results)**

- **Figure S9-26:** Multiple figures detailing the (U)HPLC-HRMS(/MS) method optimisation. Includes 2-dimensional density plot of reproducibly detectable metabolic features (RDMFs) for each method assessed and summary plots of the counts of RDMFsS across the different methods.

**Supplemental figures (*D. magna* annotation results)**

- **Figure S27:** Venn diagram of metabolite annotations observed for 1D- & 2D-NMR, GC-EI-HRMS and (U)HPLC-HRMS(/MS) and DI-HRMS(MS$^n$) measurement techniques
- **Figure S28:** Venn diagram of metabolite annotations observed across computational annotation approach used.

- **Figure S29:** Distribution of unique metabolite annotations across monoisotopic exact mass
- **Figure S30:** Assessment of the DMA experimental and computational workflow
- **Figure S31:** Summary of which annotation approach was able to identify each metabolite standard
- **Figure S32**: Summary of the top canonical pathways derived using QIAGEN Ingenuity Pathway Analysis (IPA) for all annotations obtained from the DMA of D. magna
- **Figure S33:** GNPS spectral network analysis (positive ionisation mode).

## 7 Abbreviations

| | |
|---|---|
| (U)HPLC-HRMS(/MS) | (Ultra)high-performance liquid chromatography-high resolution tandem mass spectrometry |
| 1D- & 2D-NMR | 1- and 2-dimensional nuclear magnetic resonance |
| AMD | Accucore Amide liquid chromatography column |
| AMP | Weak anion-exchange SPE cartridges (apolar arm of workflow) |
| C18 | Reversed-phase C18 SPE cartridges |
| C30 | Accucore C30 RPLC column (C30) |
| CID | Collision-induced dissociation |
| DDA | Data dependent acquisition |
| DI-HRMS(/MS$^n$) | Direct infusion-high resolution mass spectrometry (with multiple-stage fragmentation) |
| DMA | Deep metabolome annotation |
| DMAdb | Deep metabolome annotation database |
| GC-EI-HRMS | Gas chromatography-electron ionisation-high resolution mass spectrometry |
| HCD | Higher energy collisional dissociation |
| HILIC | Hydrophilic interaction liquid chromatography |
| HRMS(/MS) | High resolution mass spectrometry (with tandem mass spectrometry) |
| NCE | Normalised collision energy |
| RPLC | Reverse-phase liquid chromatography |
| PHE | Syncronis Phenyl liquid chromatography column |
| SPE | Solid-phase extraction |
| WAX | Weak anion-exchange SPE cartridges (polar arm of workflow) |
| WCX | Weak-cation exchange SPE cartridges |

## 8 Acknowledgements

We would like to thank several current and former Thermo Fisher Scientific scientists for their helpful advice, including Martin Hornshaw, David Peake, Amanda Souza, Ioanna Ntai and Tim Stratton, as well as Anthony Edge and Alex Adam who co-supervised MRJ's iCASE PhD studentship. We are also grateful to Peter Li from *Gigascience* who co-supervised TNL's iCASE PhD studentship and provided early guidance related to Galaxy and database development. We also thank John Colbourne for his insightful feedback provided throughout his co-supervision of both MRJ's and TNL's PhDs. We thank Karl Burgess for his oversight

as Head of Metabolomics at Glasgow Polyomics, University of Glasgow where the GC-EI-HRMS measurements were performed. Thanks also to the Galaxy community for helpful discussions and contributions regarding the Galaxy tools and workflow development, and to Dominic Wilson and Andrew Edmonds from the Research Software Group, part of Advanced Research Computing at the University of Birmingham [56]~~(https://www.birmingham.ac.uk/bear-software)~~ for help with setting up the IT infrastructure for data storage, DMAdb and the Galaxy platform. Finally, we are particularly grateful to Clement Heude (no longer in the field) and the Biomolecular NMR Facility team at the University of Birmingham for the NMR measurements.

## 9 Author contributions

**TNL:** Formal analysis [lead], Software [lead], ~~Formal analysis [lead],~~ Visualisation [lead], Data curation [lead], Writing – original draft [lead], Writing – review & editing [equal], Conceptualization [equal], Investigation [supporting] and Methodology [supporting].

**MRJ:** Investigation [lead], Methodology [lead], Conceptualization [equal], Formal analysis [equal], Software [equal], Visualisation [equal], Data curation [equal], Writing – original draft [lead] and Writing – review & editing [equal].

~~**TNL:** Software [lead], Formal analysis [lead], Visualisation [lead], Data curation [lead], Writing – original draft [lead], Writing – review & editing [equal], Conceptualization [equal], Investigation [supporting] and Methodology [supporting].~~

**AJC:** Investigation [supporting], Methodology [supporting], and Writing – review & editing [supporting].

**ES:** Formal analysis [supporting], Visualisation [supporting], and Writing – review & editing [supporting].

**SW:** Investigation [supporting], Methodology [supporting], Formal analysis [supporting] and Writing – review & editing [supporting].

**RM:** Supervision [supporting] and Writing – review & editing [supporting].

**WD:** Supervision [supporting], Conceptualization [supporting], and Writing – review & editing [supporting].

**RJMW:** Supervision [equal]; Software [equal], Conceptualization [equal], Writing – original draft [equal], Writing – review & editing [equal]; Methodology [supporting], Formal analysis [supporting] and Data curation [supporting].

**MRV:** Supervision [lead]; Conceptualization [equal]; Writing – original draft [equal] and Writing – review & editing [equal].

## 10 Funding

This work was supported financially through two NERC CASE PhD studentships at the University of Birmingham with GigaScience (NE/L002493/1 – CENTA: Central England

NERC Training Alliance; TNL) and Thermo Fisher Scientific (NE/J017442/1; MRJ). The work was also funded through the Wellcome Trust research grant “MetaboFlow” (202952/Z/16/Z; TNL, MRJ, RJMW, MRV) and funding from the European Union’s Horizon 2020 Research and Innovation programme under Grant Agreement No. 965406 “PrecisionTox” (TNL, MRJ, RJMW, ES, MRV). This output reflects only the authors’ views and the European Union cannot be held responsible for any use that may be made of the information contained therein.

## 11 Data availability

All raw (U)HPLC-HRMS(/MS) and DI-HRMS(/MSn) data and selected annotations supporting the results of this article are available through MetaboLights (MTBLS2273~~MTBLS2273~~). Additionally, the mass spectrometry files containing mass spectrometry gas-phase fragmentation spectra used for the GNPS analysis of the *D. magna* sample (and not equilibration, blank or reference standard samples) are also available through GNPS MassIVE repository (MSV000094957). The assay format was simplified for MetaboLights and MassIVE into four assays (apolar positive, apolar negative, polar positive and polar negative).

~~Mass spectral libraries used for the Galaxy workflow are available via github (github.com/computational-metabolomics/msp2db/releases/tag/v0.0.14-mona-23042021).~~

The GitHub repository “dmagna-dma-paper” (Section 5) additionally includes the consolidated annotation file spanning all assays that was used for figure generation and the derivation of summary information, along with details on accessing data from the associated Galaxy workflow histories.

Additionally, ~~T~~the DMAdb web portal can also be used to access ~~all of~~all the raw and processed (U)HPLC-HRMS(/MS) and DI-HRMS(/MSn) data as well as viewing and searching the fragmentation spectra and metabolite annotations.

## 12 Competing interests

The authors declare no competing interests.

## 13 References

[1] T. M. Keane, L. Goodstadt, P. Danecek, M. A. White, K. Wong, B. Yalcin, *et al.*, ‘Mouse genomic variation and its effect on phenotypes and gene regulation’, *Nature*, vol. 477, no. 7364, pp. 289–294, Sep. 2011, doi: 10.1038/nature10413.
[2] I. Dunham, A. Kundaje, S. F. Aldred, P. J. Collins, C. a. Davis, F. Doyle, *et al.*, ‘An integrated encyclopedia of DNA elements in the human genome’, *Nature*, vol. 489, no. 7414, pp. 57–74, 2012, doi: 10.1038/nature11247.
[3] L. Hood and L. Rowen, ‘The human genome project: big science transforms biology and medicine’, *Genome Med*, vol. 5, no. 9, p. 79, 2013, doi: 10.1186/gm483.
[4] The 1000 Genomes Project Consortium, Corresponding authors, A. Auton, G. R. Abecasis, Steering committee, D. M. Altshuler, *et al.*, ‘A global reference for human

genetic variation', *Nature*, vol. 526, no. 7571, pp. 68–74, Oct. 2015, doi: 10.1038/nature15393.
[5] S. Moco and J. M. Buescher, 'Metabolomics: going deeper, going broader, going further', *Cell-Wide Identification of Metabolite-Protein Interactions*, pp. 155–178, 2022.
[6] E. Puris, Š. Kouřil, L. Najdekr, S. Auriola, S. Loppi, P. Korhonen, *et al.*, 'Metabolomic, Lipidomic and Proteomic Characterisation of Lipopolysaccharide-induced Inflammation Mouse Model', *Neuroscience*, vol. 496, pp. 165–178, Aug. 2022, doi: 10.1016/j.neuroscience.2022.05.030.
[7] A.-T. Ramabulana, D. Petras, N. E. Madala, and F. Tugizimana, 'Mass spectrometry DDA parameters and global coverage of the metabolome: Spectral molecular networks of momordica cardiospermoides plants', *Metabolomics*, vol. 19, no. 3, p. 18, Mar. 2023, doi: 10.1007/s11306-023-01981-4.
[8] D. S. Wishart, D. Tzur, C. Knox, R. Eisner, A. C. Guo, N. Young, *et al.*, 'HMDB: the Human Metabolome Database.', *Nucleic acids research*, vol. 35, no. Database issue, pp. D521--6, Jan. 2007, doi: 10.1093/nar/gkl923.
[9] H. Horai, M. Arita, S. Kanaya, Y. Nihei, T. Ikeda, K. Suwa, *et al.*, 'MassBank: A public repository for sharing mass spectral data for life sciences', *J. Mass Spectrom.*, vol. 45, no. 7, pp. 703–714, 2010, doi: 10.1002/jms.1777.
[10] T. Kind, K.-H. Liu, D. Y. Lee, B. DeFelice, J. K. Meissen, and O. Fiehn, 'LipidBlast in silico tandem mass spectrometry database for lipid identification.', *Nat Methods*, vol. 10, no. 8, pp. 755–758, Aug. 2013, doi: 10.1038/nmeth.2551.
[11] M. Wang, J. J. Carver, V. V. Phelan, L. M. Sanchez, N. Garg, Y. Peng, *et al.*, 'Sharing and community curation of mass spectrometry data with Global Natural Products Social Molecular Networking', *Nat Biotechnol*, vol. 34, no. 8, pp. 828–837, Aug. 2016, doi: 10.1038/nbt.3597.
[12] K. Haug, R. M. Salek, P. Conesa, J. Hastings, P. de Matos, M. Rijnbeek, *et al.*, 'MetaboLights--an open-access general-purpose repository for metabolomics studies and associated meta-data.', *Nucleic acids research*, vol. 41, no. Database issue, pp. D781--6, Jan. 2013, doi: 10.1093/nar/gks1004.
[13] M. Sud, E. Fahy, D. Cotter, K. Azam, I. Vadivelu, C. Burant, *et al.*, 'Metabolomics Workbench: An international repository for metabolomics data and metadata, metabolite standards, protocols, tutorials and training, and analysis tools', *Nucleic Acids Res*, vol. 44, no. D1, p. gkv1042, 2015, doi: 10.1093/nar/gkv1042.
[14] M. R. Viant, I. J. Kurland, M. R. Jones, and W. B. Dunn, 'How close are we to complete annotation of metabolomes?', *Current Opinion in Chemical Biology*, vol. 36, pp. 64–69, Feb. 2017, doi: 10.1016/j.cbpa.2017.01.001.
[15] A. S. Edison, R. D. Hall, C. Junot, P. D. Karp, I. J. Kurland, R. Mistrik, *et al.*, 'The time is right to focus on model organism metabolomes', *Metabolites*, vol. 6, no. 1, p. 8, 2016, doi: 10.3390/metabo6010008.
[16] 'The Precision Toxicology initiative', *Toxicology Letters*, vol. 383, pp. 33–42, Jul. 2023, doi: 10.1016/j.toxlet.2023.05.004.
[17] A. Weismann, *Beiträge zur Naturgeschichte der Daphnoiden*, vol. 2. W. Engelmann, 1876.
[18] D. Ebert, *Introduction to the ecology, epidemiology, and evolution of parasitism in Daphnia*. National Center for Biotechnology Information (US), 2005.
[19] D. Ebert, 'Daphnia as a versatile model system in ecology and evolution', *EvoDevo*, vol. 13, no. 1, p. 16, Aug. 2022, doi: 10.1186/s13227-022-00199-0.
[20] W. Lampert, 'Daphnia: Model herbivore, predator and prey', *Polish Journal of Ecology*, vol. 54, no. 4, pp. 607–620, 2006.
[21] J. K. Colbourne, M. E. Pfrender, D. Gilbert, W. K. Thomas, A. Tucker, T. H. Oakley, *et al.*, 'The ecoresponsive genome of Daphnia pulex.', *Science (New York, N.Y.)*, vol. 331, no. 6017, pp. 555–61, Feb. 2011, doi: 10.1126/science.1197761.
[22] E. Afgan, D. Baker, B. Batut, M. van den Beek, D. Bouvier, M. Čech, *et al.*, 'The Galaxy platform for accessible, reproducible and collaborative biomedical analyses: 2018

update', *Nucleic Acids Research*, vol. 46, no. W1, pp. W537–W544, Jul. 2018, doi: 10.1093/nar/gky379.
[23] F. Giacomoni, G. Le Corguille, M. Monsoor, M. Landi, P. Pericard, M. Petera, *et al.*, 'Workflow4Metabolomics: a collaborative research infrastructure for computational metabolomics', *Bioinformatics*, vol. 31, no. 9, pp. 1493–1495, May 2015, doi: 10.1093/bioinformatics/btu813.
[24] A. D. Southam, R. J. M. Weber, J. Engel, M. R. Jones, and M. R. Viant, 'A complete workflow for high-resolution spectral-stitching nanoelectrospray direct-infusion mass-spectrometry-based metabolomics and lipidomics', *Nature Protocols*, vol. 12, no. 2, pp. 310–328, Feb. 2017, doi: 10.1038/nprot.2016.156.
[25] R. J. M. W. and Jiarui Zhou, *DIMSpy: Python package for processing direct- infusion mass spectrometry-based metabolomics and lipidomics data*. (Apr. 2020). Zenodo. doi: 10.5281/zenodo.3764169.
[26] T. N. Lawson, R. J. M. Weber, M. R. Jones, A. J. Chetwynd, G. A. Rodriguez Blanco, R. Di Guida, *et al.*, 'msPurity: Automated evaluation of precursor ion purity for mass spectrometry based fragmentation in metabolomics', *Anal. Chem.*, vol. 89, no. 4, p. acs.analchem.6b04358, 2017, doi: 10.1021/acs.analchem.6b04358.
[27] C. Ruttkies, E. L. Schymanski, S. Wolf, J. Hollender, and S. Neumann, 'MetFrag relaunched: Incorporating strategies beyond in silico fragmentation', *J Cheminform*, vol. 8, no. 1, pp. 1–16, 2016, doi: 10.1186/s13321-016-0115-9.
[28] C. Ruttkies, S. Neumann, and S. Posch, 'Improving MetFrag with statistical learning of fragment annotations', *BMC Bioinformatics*, vol. 20, no. 1, p. 376, Dec. 2019, doi: 10.1186/s12859-019-2954-7.
[29] S. Wolf, S. Schmidt, M. Müller-Hannemann, and S. Neumann, 'In silico fragmentation for computer assisted identification of metabolite mass spectra', *BMC Bioinformatics*, vol. 11, no. 1, p. 148, 2010, doi: 10.1186/1471-2105-11-148.
[30] K. Dührkop, M. Fleischauer, M. Ludwig, A. A. Aksenov, A. V. Melnik, M. Meusel, *et al.*, 'SIRIUS 4: a rapid tool for turning tandem mass spectra into metabolite structure information', *Nat Methods*, vol. 16, no. 4, pp. 299–302, Apr. 2019, doi: 10.1038/s41592-019-0344-8.
[31] HighChem LLC, 'mzCloud: Advanced mass spectral database'. Accessed: Feb. 12, 2026. [Online]. Available: https://www.mzcloud.org/
[32] Global Natural Products Social Molecular Networking (GNPS), 'GNPS: Global natural products social molecular networking'. Accessed: Feb. 12, 2026. [Online]. Available: https://gnps.ucsd.edu
[33] H. Mohimani, A. Gurevich, A. Mikheenko, N. Garg, L.-F. Nothias, A. Ninomiya, *et al.*, 'Dereplication of peptidic natural products through database search of mass spectra', *Nature Chemical Biology*, vol. 13, no. 1, pp. 30–37, Jan. 2017, doi: 10.1038/nchembio.2219.
[34] J. Wandy, Y. Zhu, J. J. J. van der Hooft, R. Daly, M. P. Barrett, and S. Rogers, 'Ms2lda.org: web-based topic modelling for substructure discovery in mass spectrometry', *Bioinformatics*, vol. 34, no. 2, pp. 317–318, Jan. 2018, doi: 10.1093/bioinformatics/btx582.
[35] Y. Djoumbou Feunang, R. Eisner, C. Knox, L. Chepelev, J. Hastings, G. Owen, *et al.*, 'ClassyFire: automated chemical classification with a comprehensive, computable taxonomy', *J Cheminform*, vol. 8, no. 1, p. 61, Dec. 2016, doi: 10.1186/s13321-016-0174-y.
[36] M. Kanehisa and S. Goto, 'KEGG: Kyoto Encyclopedia of Genes and Genomes', *Nucleic Acids Research*, vol. Vol. 28, no. 1, pp. 27–30, Jan. 2000.
[37] M. Kanehisa, 'Toward understanding the origin and evolution of cellular organisms', *Protein Science*, vol. 28, no. 11, pp. 1947–1951, Nov. 2019, doi: 10.1002/pro.3715.
[38] M. Kanehisa, M. Furumichi, Y. Sato, M. Kawashima, and M. Ishiguro-Watanabe, 'KEGG for taxonomy-based analysis of pathways and genomes', *Nucleic Acids Research*, vol. 51, no. D1, pp. D587–D592, Jan. 2023, doi: 10.1093/nar/gkac963.

[39] J. Hastings, P. de Matos, A. Dekker, M. Ennis, B. Harsha, N. Kale, *et al.*, ‘The ChEBI reference database and ontology for biologically relevant chemistry: enhancements for 2013.’, *Nucleic acids research*, vol. 41, no. Database issue, pp. D456--63, Jan. 2013, doi: 10.1093/nar/gks1146.
[40] E. Sostare, T. N. Lawson, L. R. Saunders, J. K. Colbourne, R. J. M. Weber, T. Sobanski, *et al.*, ‘Knowledge-Driven Approaches to Create the MTox700+ Metabolite Panel for Predicting Toxicity’, *Toxicological Sciences*, vol. 186, no. 2, pp. 208–220, Mar. 2022, doi: 10.1093/toxsci/kfac007.
[41] Biobyte Solutions GmbH, ‘PhyloT: Phylogenetic tree generator’. Accessed: Feb. 12, 2026. [Online]. Available: https://phylot.biobyte.de/index.cgi
[42] S. Böcker and F. Rasche, ‘Towards de novo identification of metabolites by analyzing tandem mass spectra’, *Bioinformatics*, vol. 24, no. 16, pp. 49–55, 2008, doi: 10.1093/bioinformatics/btn270.
[43] K. Peters, S. Herman, P. E. Khoonsari, J. Burman, S. Neumann, and K. Kultima, ‘Metabolic drift in the aging nervous system is reflected in human cerebrospinal fluid’, *Sci Rep*, vol. 11, no. 1, p. 18822, Sep. 2021, doi: 10.1038/s41598-021-97491-1.
[44] L. Colas, A.-L. Royer, J. Massias, A. Raux, M. Chesneau, C. Kerleau, *et al.*, ‘Urinary metabolomic profiling from spontaneous tolerant kidney transplanted recipients shows enrichment in tryptophan-derived metabolites’, *eBioMedicine*, vol. 77, p. 103844, Mar. 2022, doi: 10.1016/j.ebiom.2022.103844.
[45] E. Eysseric, C. Gagnon, and P. A. Segura, ‘Identifying congeners and transformation products of organic contaminants within complex chemical mixtures in impacted surface waters with a top-down non-targeted screening workflow’, *Science of The Total Environment*, vol. 822, p. 153540, May 2022, doi: 10.1016/j.scitotenv.2022.153540.
[46] J. Rainer, A. Vicini, L. Salzer, J. Stanstrup, J. M. Badia, S. Neumann, *et al.*, ‘A Modular and Expandable Ecosystem for Metabolomics Data Annotation in R’, *Metabolites*, vol. 12, no. 2, p. 173, Feb. 2022, doi: 10.3390/metabo12020173.
[47] C. A. Smith, E. J. Want, G. O. Maille, R. Abagyan, and G. Siuzdak, ‘XCMS : Processing Mass Spectrometry Data for Metabolite Profiling Using Nonlinear Peak Alignment , Matching , and Identification’, *Anal. Chem.*, vol. 78, no. 3, pp. 779–787, 2006, doi: 10.1021/ac051437y.
[48] C. Kuhl, R. Tautenhahn, C. Böttcher, T. R. R. Larson, S. Neumann, C. Bo, *et al.*, ‘CAMERA: An integrated strategy for compound spectra extraction and annotation of liquid chromatography/mass spectrometry data sets’, *Anal. Chem.*, vol. 84, no. 1, pp. 283–289, Jan. 2012, doi: 10.1021/ac202450g.
[49] H. Wickham, *ggplot2: Elegant graphics for data analysis*. Springer-Verlag New York, 2016. [Online]. Available: https://ggplot2.tidyverse.org
[50] J. R. Conway, A. Lex, and N. Gehlenborg, ‘UpSetR: An R package for the visualization of intersecting sets and their properties’, *Bioinformatics*, vol. 33, no. 18, pp. 2938–2940, 2017, doi: 10.1093/bioinformatics/btx364.
[51] G. Yu, ‘Using ggtree to visualize data on tree-like structures’, *Current protocols in bioinformatics*, vol. 69, no. 1, p. e96, 2020.
[52] E. Paradis and K. Schliep, ‘ape 5.0: an environment for modern phylogenetics and evolutionary analyses in R’, *Bioinformatics*, vol. 35, no. 3, pp. 526–528, Feb. 2019, doi: 10.1093/bioinformatics/bty633.
[53] S. Xu, Q. Wang, S. Wen, J. Li, N. He, M. Li, *et al.*, ‘aplot: Simplifying the creation of complex graphs to visualize associations across diverse data types’, *The Innovation*, p. 100958, May 2025, doi: 10.1016/j.xinn.2025.100958.
[54] Y. Cao, A. Charisi, L.-C. Cheng, T. Jiang, and T. Girke, ‘ChemmineR: a compound mining framework for R’, *Bioinformatics*, vol. 24, no. 15, pp. 1733–1734, Aug. 2008, doi: 10.1093/bioinformatics/btn307.
[55] S. Kim, J. Chen, T. Cheng, A. Gindulyte, J. He, S. He, *et al.*, ‘PubChem 2025 update’, *Nucleic Acids Research*, vol. 53, no. D1, pp. D1516–D1525, Jan. 2025, doi: 10.1093/nar/gkae1059.

[56] University of Birmingham, ‘BEAR software: Birmingham environment for academic research’. Accessed: Feb. 12, 2026. [Online]. Available: https://www.birmingham.ac.uk/bear-software

## 14 Author notes

Martin R. Jones and Thomas N. Lawson contributed equally to this article.

Publisher’s notes.

© The Author(s) 2026~~5~~. Published by Oxford University Press GigaScience.

This is an Open Access article distributed under the terms of the Creative Commons Attribution License (https://creativecommons.org/licenses/by/4.0/), which permits unrestricted reuse, distribution, and reproduction in any medium, provided the original work is properly cited.

# Experimental and computational approaches for deep metabolome annotation with application to ecotoxicological model organism *Daphnia magna*

## Supplemental information

Thomas N. Lawson[1,2,†], Martin R. Jones[1,†], ~~Thomas N. Lawson[1,2,†],~~ Andrew J. Chetwynd[1,3,α], Elena Sostare[2], Stefan Weidt[5], Robert Mistrik[4,δ], Warwick B. Dunn[1,3,§], Ralf J. M. Weber[1,3,*], Mark R. Viant[1,2,3,*]

[1]School of Biosciences, University of Birmingham, Edgbaston, Birmingham, B15 2TT, UK
[2]Michabo Health Science Limited, Union House, 111 New Union Street, Coventry, CV1 2NT, UK
[3]Phenome Centre Birmingham, University of Birmingham, Edgbaston, Birmingham, B15 2TT, UK
[4]HighChem, Mlynské nivy 5, 821 09 Bratislava, Slovakia
[5]Glasgow Polyomics, University of Glasgow, University Avenue, Glasgow, G12 8QQ, UK

[†]Joint first authors
Present addresses: [α]Centre for Proteome Research, and [§]Centre for Metabolomics Research, Department of Biochemistry, Cell and Systems Biology, Institute of Systems, Molecular and Integrative Biology, University of Liverpool, Liverpool, L69 7ZB, UK; [δ]Bitmoderna, Leskova 11, 81104 Bratislava, Slovakia.

*Correspondence address: School of Biosciences, University of Birmingham, Edgbaston, Birmingham, B15 2TT, UK; E-mail: m.viant@bham.ac.uk; E-Mail: r.j.weber@bham.ac.uk

**Formatted**

**Formatted:** Font: 9 pt, Font color: Auto

**Formatted:** Centered, Space Before: 0 pt, After: 0 pt

**Formatted:** Font: 9 pt, Do not check spelling or grammar, All caps

# Table of contents


1 Supplemental – materials and methods 4

1.1 Summary of assays and files 5

1.2 Chemicals 5

1.3 Solvents and solutions 5

1.4 Consumables 6

1.5 *D. magna* culturing and sample preparation 6

1.6 Metabolite extraction from homogenised *D. magna* biomass 11

1.7 Solid phase extraction-based fractionation of metabolite extracts 12

1.8 DMA (U)HPLC-HRMS(/MS), DI-HRMS(/MS$^n$) and LC fractionation 15

1.9 (U)HPLC-HRMS(/MS) method optimisation 26

1.10 GC-EI-HRMS 5

1.11 1D- & 2D-NMR 7

1.12 DMA computational workflow overview 9

1.13 DMA Galaxy workflow 10

1.14 Combining and summarising all annotations 29

1.15 Assessment of the computational and experimental DMA workflow with metabolite reference standards 30

2 Supplemental - results 31

2.1 (U)HPLC-HRMS(/MS) method optimisation 32

2.2 Summary of all DMA of D. magna annotations 58

2.3 (U)HPLC-HRM(/MS) and DI-HRMS(/MS$^n$) derived metabolite annotations 59

2.4 GC-EI-HRMS derived metabolite annotations 62

2.5 NMR derived metabolite annotations 64

2.6 Assessment of the computational and experimental DMA workflow with metabolite reference standards 71

2.7 Pathway analysis 74

2.8 Molecular network analysis using GNPS 77

3 References 79

~~1 Supplemental – materials and methods 3~~

~~1.1 Summary of assays and files 4~~


**Formatted:** Default Paragraph Font, Check spelling and grammar

**Formatted:** Default Paragraph Font, Check spelling and grammar

**Formatted:** Default Paragraph Font, Check spelling and grammar

**Formatted:** Default Paragraph Font, Check spelling and grammar

**Formatted:** Font: 9 pt, Font color: Auto

**Formatted:** Centered, Space Before: 0 pt, After: 0 pt

**Formatted:** Font: 9 pt, Do not check spelling or grammar, All caps

1.2 Chemicals 4

1.3 Solvents and solutions 4

1.4 Consumables 5

1.5 D. magna culturing and sample preparation 5

1.6 Metabolite extraction from homogenised D. magna biomass 10

1.7 Solid phase extraction-based fractionation of metabolite extracts 11

1.8 DMA (U)HPLC-HRMS(/MS), DI-HRMS(/MSn) and LC fractionation 14

1.9 (U)HPLC-HRMS(/MS) method optimisation 25

1.10 GC-EI-HRMS 6

1.11 1D- & 2D-NMR 8

1.12 DMA computational workflow overview 10

1.13 DMA Galaxy workflow 11

1.14 Combining and summarising all annotations 30

1.15 Assessment of the computational and experimental DMA workflow with metabolite reference standards 31

2 Supplemental - results 32

2.1 (U)HPLC-HRMS(/MS) method optimisation 33

2.2 Summary of all DMA of D. magna annotations 59

2.3 (U)HPLC-HRM(/MS) and DI-HRMS(/MSn) derived metabolite annotations 60

2.4 GC-EI-HRMS derived metabolite annotations 62

2.5 NMR derived metabolite annotations 64

2.6 Assessment of the computational and experimental DMA workflow with metabolite reference standards 71

2.7 Pathway analysis 74

2.8 Molecular network analysis using GNPS 75

3 References 77

# 1 Supplemental – materials and methods

Formatted: Font: 9 pt, Font color: Auto
Formatted: Centered, Space Before: 0 pt, After: 0 pt
Formatted: Font: 9 pt, Do not check spelling or grammar, All caps

## 1.1 Summary of assays and files

See **Supplemental Table S1** (provided in separate excel file) for a summary of all experimental assays performed.

See **Supplemental Table S2** (provided in separate excel file) for the (ultra)-high-performance liquid chromatography-high resolution tandem mass spectrometry ((U)HPLC-HRMS(/MS)) and the direct infusion-high resolution mass spectrometry with multiple-stage fragmentation (DI-HRMS(/$MS^n$)) data files used.

## 1.2 Chemicals

Acetic acid (≥ 99.0% w/w, TraceSelect grade), ammonium acetate (≥ 99.99%, trace metals basis), ammonium formate (≥ 99.9% w/w trace metals basis), ammonium hydroxide (≥ 25.0% w/w), boric acid (98.5% for molecular biology), calcium chloride dihydrate (ReagentPlus, ≥ 99.0%), cobalt (II), nitrate hexahydrate (laboratory grade), copper (II) sulfate pentahydrate (laboratory grade), ethylenediaminetetraacetic acid tetra-sodium salt (≥ 99.0%), formic acid (≥ 98% w/w, TraceSelect grade), lithium chloride anhydrous (BioUltra for molecular biology), magnesium sulfate heptahydrate (BioXtra, ≥ 99.0%), manganese (II) chloride tetrahydrate (ReagentPlus, ≥ 99.0%), potassium phosphate dibasic (ACS reagent, ≥ 98%), potassium phosphate monobasic (≥ 99.0%), potassium chloride (ACS reagent, 99.0-100.5%), potassium hydroxide (≥85.0%, pellets), potassium iodide (BioUltra, ≥99.5%), rubidium chloride (≥99%, AT), sodium bicarbonate, sodium bromide (99%), sodium selenite, strontium chloride hexahydrate (ACS reagent, 99%), sulfuric acid and zinc (II) sulfate were all purchased from Sigma Aldrich. Iron (II) sulfate heptahydrate (≥ 99%) and sodium metasilicate nonahydrate were purchased from Acros Organics. Sodium molybdenum oxide dihydrate (ACS, 99.5-103.0% ) was sourced through Alfa Aesar. Sodium chloride (analytical reagent grade), sodium phosphate monobasic (anhydrous, Enzyme-grade (99%), and sodium phosphate dibasic (anhydrous, Bioreagent, ≥99.0%) were sources from Fisher Scientific. Sodium-3-(trimethylsilyl)-proprionate-2,2,3,3-$d_4$ (98%) was purchased from Cambridge Isotope Laboratories Inc.

## 1.3 Solvents and solutions

All solvents used in metabolite extractions, solid phase extractions, (U)HPLC-HRMS(/MS) and DI-HRMS(/$MS^n$) analyses were of HPLC-grade or better: water (various vendors), methanol (various vendors), chloroform (Fisher Scientific, HPLC grade, 99.8% stabilised with amylenes), propan-2-ol (Fisher Scientific, Optima (U)HPLC-HRMS(/MS), 99.9%),.n-hexane (Acros Organisc, 97%+ for HPLC), diethyl ether (Sigma Aldrich, Chromasolv for HPLC, ≥99.9%, inhibitor free), ethanol (Fisher Scientific, HPLC-grade, 'Ethanol absolute'), and phosphoric acid (Fluka Analytical, for HPLC (85-90%).

All mass spectrometer calibration solutions were purchased from Thermo Fisher Scientific.

**Formatted:** Font: 9 pt, Font color: Auto

**Formatted:** Centered, Space Before: 0 pt, After: 0 pt

**Formatted:** Font: 9 pt, Do not check spelling or grammar, All caps

## 1.4 Consumables

HyperSep WCX (carboxylic acid), HyperSep WAX (aminopropyl) and HyperSep C18 SPE cartridges (3 mL, 500 mg), and Syncronis Phenyl (2.1 x 100 mm, 1.7 µm), Accucore Amide (2.1 x 100 m, 2.6 µm) and Accucore C30 (2.1 x 100 mm, 2.6 µm) LC columns, were provided by Fisher Scientific (Hemel Hempstead, UK).

Easy Pierce foil (20 µm) and 384-well polypropylene well plates (AB-Gene) were purchased from Thermo Scientific. Glass champagne vials (1.5 mL, clear, VZM-1509CC-100) and caps (9-425 Blue Screw Thread with PTFE/silicone/PTFE septa), used in NMR experiments, were purchased from Cronus. ACQUITY UPLC 700 µL 96-well deep well plates, used for LC fraction capture and storage, were purchased from Waters. Polypropylene microfuge tubes (1.5 mL, clear, Safe-Lock) were bought from Eppendorf. Aluminium self-adhesive well plate sealing tape was purchased from Corning.

## 1.5 *D. magna* culturing and sample preparation

Stock cultures of ten *D. magna* isolates (see **Table S3**) were maintained in modified high-hardness COMBO medium (Baer and Goulden, 1998) (see **Table S4**) at 20 +/- 2 °C and under a 16:8 hr light:dark cycle, at a target density of 20 female *D. magna* per 1.2 L of medium. A concentrated isolate of *Chlorella vulgaris,* (equivalent to 0.5 mg carbon per ~~liter~~ litre and cultured under non-axenic conditions in Bold's Basal medium – see **Table S5**) was supplied as feed material, at an equivalent daily dose of*:* 0.5 mL/day for days 1-2; 0.75 mL/day for days 3-7 and; 1 mL/day for days 8 onwards. Culture medium was replaced weekly, between which neonates and any unexpected male daphniids were carefully removed. Each *Daphnia* stock culture was derived from an existing stock culture by transferring twenty < 24 hr neonates (third brood or later) to freshly aerated culture medium. Under these conditions, cultures comprised almost exclusively parthenogenetic females. All cultures were visually inspected daily for potential bacterial or fungal contamination. Where contamination was evident or suspected, cultures were promptly discarded.

*D. magna* used in DMA experiments were derived from corresponding stock cultures by transferring 20 neonates (age < 24 hr, third brood or later) into fresh culturing medium. These experimental cultures were maintained as per stock cultures until day 14, at which point adult *D. magna* were transferred to fresh medium in the absence of food. Adults were then maintained without food for two days, under one of two conditions, either: 1) 20 +/- 2 °C with a 16:8 hr light:dark ratio for 48 hr ('basal' metabolome); or 2) 10 +/- 1 °C and with 16:8 hr light:dark ratio for 24 hr, then 10 +/- 1 °C and with 8:16 hr light:dark ratio for a further 24 hr ('stressed' metabolome). Thereafter, remaining female daphniids were collected and metabolically quenched.

To quench metabolic processes at the end of the culturing period, *Daphnia* were rapidly flash frozen in liquid nitrogen. To do so, *D. magna* cultures were first passed through a coarse gauze mesh to isolate *D. magna* from their culturing medium. *D. magna*, trapped on the mesh, were

**Formatted:** Font: 9 pt, Font color: Auto

**Formatted:** Centered, Space Before: 0 pt, After: 0 pt

**Formatted:** Font: 9 pt, Do not check spelling or grammar, All caps

then blot dried with tissue paper to remove excess media and transferred to polypropylene tubes using a fine-haired paintbrush, wherein they were submerged in liquid nitrogen. Resulting samples were either used immediately or stored at -80 °C, until required. Excess stock culture daphniids used in DMA workflow optimisation experiments (“**Development samples”**), and daphniids included in the final DMA workflow application experiments (“**DMA samples”**), were all collected using this process.

Prior to metabolite extraction, *D. magna* samples were cryogenically cooled in liquid nitrogen and homogenised over dry ice using a ceramic mortar and pestle. Resulting homogenate was transferred to a pre-cooled 28 mL glass vial for metabolite extraction.

DMA samples were homogenised in two batches, with approximately 50% of DMA samples homogenised in each. Approximately 60% of each resulting homogenate was transferred to a single 28 mL glass vial for extraction of “polar” metabolites, with the remainder transferred to two separate 28 mL glass vials for extraction of “apolar” metabolites.

**Table S3**: *D. magna* ~~cultures~~strains

| Geographic origin | Supplier | Strain name |
|---|---|---|
| Antwerp, Belgium | University of Antwerp. | AW |
| Birmingham, UK | University of Birmingham. | B1 |
| Birmingham, UK | University of Birmingham. | B2 |
| Kent, UK | Blades Biological – Commercial Supplier. | BD |
| California, USA | University of California, Berkeley. | BK |
| Minnesota, USA | US Environmental Protection Agency (EPA). | EPA |
| Reading, UK | University of Reading. | RD |
| Okazaki, Japan | Okazaki Institute for Integrative Science, National Institute of Natural Sciences. | NS |
| Alessandria, Italy | Universitá del Piemonte Orientale. | UPO |
| UK | Industrial Strain. | IS |

**Formatted:** Font: 9 pt, Font color: Auto

**Formatted:** Centered, Space Before: 0 pt, After: 0 pt

**Formatted:** Font: 9 pt, Do not check spelling or grammar, All caps

**Table S4**: High-hardness COMBO and modified high hardness COMBO medium

| | Compound | Stock (g/L) | Final medium mg/L | Final medium µmol/L | Component in final medium Symbol | Component in final medium mg/L | Component in final medium µmol/L | Volume added per 1 L of medium |
|---|---|---|---|---|---|---|---|---|
| Major stocks | CaCl2.2H2O | 110.28 | 110.28 | 750 | Ca | 30.1 | 750 | 1 mL |
| | | | | | Cl | 53.2 | 1500 | |
| | MgSO4.7H2O | 55.45 [113.5] | 55.45 [113.5] | 225 [461] | Mg | 5.5 [11.2] | 225 [461] | 1 mL [1 mL] |
| | | | | | SO4 | 21.6 [44.2] | 225 [461] | |
| | K2PO4 | 1.742 | 1.742 | 10 | K | 0.8 | 20 | 1 mL |
| | | | | | P | 0.3 | 10 | |
| | NaNO3 | 17 | 17 | 200 | Na | 4.6 | 200 | 1 mL |
| | | | | | NO3 | 12.4 | 200 | |
| | NaHCO3† | 126 | 126 | 1500 | Na | 34.5 | 1500 | 1 mL |
| | | | | | CO3 | 90 | 1500 | |
| | Na2SiO3.9H2O | 28.42 | 28.42 | 100 | Na | 4.6 | 200 | 1 mL |
| | | | | | Si | 2.8 | 100 | |
| | KCl | 5.96 | 5.96 | 80 | K | 3.1 | 80 | 1 mL |
| | | | | | Cl | 2.8 | 80 | |
| | H3BO3 | 24 | 24 | 388 | B | 4.2 | 388 | 1 mL |
| ANIMATE ‡ | LiCl | 0.31 | 0.31 | 7.31 | Li | 0.05 | 7.313 | 1 mL |
| | | | | | Cl | 0.26 | 7.313 | |
| | RbCl | 0.07 | 0.07 | 0.58 | Rb | 0.05 | 0.579 | |
| | | | | | Cl | 0.02 | 0.579 | |
| | SrCl2.6H2O | 0.15 | 0.15 | 0.56 | Sr | 0.05 | 0.563 | |
| | | | | | Cl | 0.04 | 1.125 | |
| | NaBr | 0.016 | 0.016 | 0.16 | Na | 0.004 | 0.156 | |
| | | | | | Br | 0.0124 | 0.156 | |
| | KI | 0.0033 | 0.0033 | 0.02 | K | 0.0008 | 0.02 | |
| | | | | | I | 0.0025 | 0.02 | |

**Formatted:** Font: 9 pt, Font color: Auto

**Formatted:** Centered, Space Before: 0 pt, After: 0 pt

**Formatted:** Font: 9 pt, Do not check spelling or grammar, All caps

| | Compound | Stock (g/L) | Final medium mg/L | Final medium µmol/L | Component in final medium Symbol | Component in final medium mg/L | Component in final medium µmol/L | Volume added per 1 L of medium |
|---|---|---|---|---|---|---|---|---|
| VIM Ŧ | *d-biotin* | 0.1042 | [0.0005] | [0.002] | - | - | - | [0.5 mL] |
| | Cyanocobalamin (B12) | 0.1124 | [0.00055] | [0.0004 ] | - | - | - | |
| | Thiamine | 0.2 | [0.1] | [0.3] | - | - | - | |
| | Na2SeO3Ŧ | 0.04 | 0.002 | 0.0875 | Na | 0.008 | 0.348 | 50 µL |
| | | | | | Se | 0.0275 | 0.348 | |
| | | | | | | | | |

† Heat to dissolve; ‡ ANIMATE: Animal trace elements; Ŧ VIM: Vitamins – only added to high-hardness COMBO medium; * Sodium selenite was added to high hardness and modified high hardness COMBO medium to enhance Daphnia reproductive output (Keating and Dagbusan, 1984). Values in square braces are for high hardness COMBO medium only (i.e. distinct from the values for modified high hardness COMBO medium). All culturing media was prepared using 15 MΩ deionised water (dH2O), to which was added 1 ml of each major stock and 50 µl of sodium selenite, per one litre of prepared medium. All media were aerated for a minimum of ten hours before adjustment to pH 7.75 ± 0.1 using hydrochloric acid and sodium hydroxide. Finally, 1 ml of ANIMATE stock solution was added per litre of medium, immediately prior to use.

**Formatted:** Font: 9 pt, Font color: Auto

**Formatted:** Centered, Space Before: 0 pt, After: 0 pt

**Formatted:** Font: 9 pt, Do not check spelling or grammar, All caps

**Table S5**: Bold's basal medium

| | | Stock solution | | | Bold's Basal Medium (BBM)† | |
|---|---|---|---|---|---|---|
| **Solution No.** | **Formula** | **g/L** | **mmol/L** | **Volume added per 1 L BBM (mL)** | **g/L** | **mmol/L** |
| 1 | $K_2HPO_4$ | 7.5 | 43.1 | 10 | 0.075 | 0.43 |
| 2 | $KH_2PO_4$ | 17.5 | 128.6 | 10 | 0.175 | 1.29 |
| 3 | $MgSO_4.7H_2O$ | 7.5 | 30.4 | 10 | 0.075 | 0.3 |
| 4 | $NaNO_3$ | 25 | 294.2 | 10 | 0.25 | 2.94 |
| 5 | $CaCl_2.2H_2O$ | 2.5 | 17 | 10 | 0.025 | 0.17 |
| 6 | NaCl | 2.5 | 42.8 | 10 | 0.025 | 0.43 |
| 7 | EDTA - $Na_4$ | 50 | 131.5 | 1 | 0.5 | 1.32 |
| | KOH | 31 | 552.5 | | 0.31 | 5.52 |
| 8 | $FeSO_4.7H_2O$ | 5 | 17.9 | 1 | 0.0498 | 0.18 |
| | $H_2SO_4$ | 1 mL | | | | |
| 9 | $H_3BO_3$ | 11.4 | 184.7 | 1 | 0.1142 | 1.85 |
| 10 | $ZnSO_4.7H_2O$ | 14.1 | 49.1 | 0.1 | 0.1412 | 0.49 |
| 11 | $MnCl_2.4H_2O$ | 2.3 | 11.7 | 0.1 | 0.0232 | 0.12 |
| 12 | $CuSO_4.5H_2O$ | 2.5 | 10.1 | 0.1 | 0.0252 | 0.1 |
| 13 | $Co(NO_3)_2.6H_2O$ | 0.8 | 2.7 | 0.1 | 0.008 | 0.03 |
| 14 | $Na_2MoO_4$-$.2H_2O$ | 1.9 | 7.9 | 0.1 | 0.0192 | 0.08 |

† The pH of the final BBM media was maintained in the range 6.7 ± 0.3 (6.4-7.0) by drop-wise addition of either hydrochloric acid or sodium hydroxide. All BBM was autoclaved (121 °C) before use.

**Formatted:** Font: 9 pt, Font color: Auto

**Formatted:** Centered, Space Before: 0 pt, After: 0 pt

**Formatted:** Font: 9 pt, Do not check spelling or grammar, All caps

## 1.6 Metabolite extraction from homogenised *D. magna* biomass

Two distinct procedures were applied for the extraction of metabolites from homogenised *D. magna* biomass. The first, aimed at extracting polar through to moderately-polar metabolites, involved addition of ice-cold methanol, then ice-cold water, to homogenised *D. magna* biomass to yield a 2.5:1 v/v methanol:water solution (accounting for approximately 80% w/w water content of an adult *D. magna*)**,** at 15 parts solution per 1 part biomass (v/w). The extraction vial was capped, vortex-mixed for 30 seconds (40 Hz, Thermo Scientific) and centrifuged for 10 minutes at 3000 rpm (approximately 1600 *x g*) and 4 °C. Supernatant was carefully transferred to a clean, ice-cold 28 mL glass vial. This "polar extract" was distributed equally across 1.5 mL polypropylene microfuge tubes, such that each tube contained a proportion of the total extract equivalent to 20 *D. magna*. Polar extract aliquots were dried in a SpeedVac at 35 °C and then transferred to a -80 °C freezer for storage.

The "apolar extraction" procedure, derived from that described by Bligh and Dyer (1959) was applied to extract moderately-polar through to highly-apolar metabolites. Ice-cold chloroform was added to homogenate using a graduated glass pipette, followed by ice-cold methanol, to give final solvent ratio of 1:1 v/v chloroform:methanol and a final extraction ratio of 30 parts extraction solution per 1 part homogenate (assumptions as per the polar extraction procedure, detailed above). The extraction vial was then capped and vortex-mixed for 30 seconds (40 Hz), followed by centrifugation for 10 minutes at 4000 rpm (approximately 2800 x *g*) and 4 °C. The supernatant was transferred to an ice-cold, 28 mL glass vial using a glass Pasteur pipette. Next, ice-cold water was added to the supernatant (taking into account the approximate water content of daphniids) to induce phase separation, with the extraction solution consisting of 1:1:0.9 v/v/v chloroform:methanol:water. After vortex mixing for 30 seconds, vials were centrifuged at 3000 rpm (approximately 1600 x *g*) for 10 mins at 4 °C and then allowed to stand on a laboratory bench for 5 minutes at room temperature. Using a 500 µL glass gas-tight syringe (Hamilton), the upper (polar) and lower (apolar) layers were transferred to separate, pre-cooled 28 mL glass vials. The syringe was rinsed before and between transfer of each layer, first using ten full volumes of methanol and then ten full volumes of chloroform. The upper and lower layers were independently split into equivolume aliquots across glass vials and tubes, respectively, such that each contained the equivalent of 20 *D. magna.* The upper 'polar' layer aliquots were dried under vacuum at 35 °C (Speedvac, Thermo Scientific), while the lower 'non-polar' layer aliquots were dried under a gaseous stream of nitrogen and then capped after flushing with nitrogen gas. Dried aliquots were stored frozen at -80 °C.

For both the polar and apolar extraction procedures, methodological blank samples were prepared by replacing *D. magna* biomass with water. The volume of water added was such that it corresponded to 80% of the total wet weight of the extracted *D. magna* biomass (assuming one adult *D. magna* has a wet mass of 1.5 mg).

For DMA samples, dried extracts resulting from the "polar" and "apolar" extraction procedures were termed "crude" extracts.

**Formatted:** Font: 9 pt, Font color: Auto

**Formatted:** Centered, Space Before: 0 pt, After: 0 pt

**Formatted:** Font: 9 pt, Do not check spelling or grammar, All caps

## 1.7 Solid phase extraction-based fractionation of metabolite extracts

Solid phase extraction (SPE) was used to fractionate metabolite extracts prior to analysis. The 'polar extract' was fractionated using weak anion-exchange (WAX; aminopropyl) and weak-cation exchange cartridges (WCX; carboxylic acid) SPE cartridges, while the 'apolar extract' was fractionated with aminopropyl weak anion-exchange (referred to as AMP to distinguish from the polar arm; aminopropyl) and reversed-phase C18 cartridges (C18). All SPE fractionation procedures were applied independently (i.e. not chained) using cartridges of 3 mL total volume and packed with 500 mg sorbent material. Solvents and solutions were passed through cartridges at approximately 1 mL/min.

A total of *n* dried extracts (maximum *n* = 10) were resuspended for SPE-based fractionation over a single SPE cartridge. Extracts, *x*, were ordered from *x* = 1 to *x* = *n*. In all but the final resuspension step, resuspension solvent or solution was added to *x* = 1, vortex mixed for 30 seconds and then transferred to *x* +1. This procedure was repeated until *x* = *n*, at which point, the resuspended extract was transferred to a glass collection vial (8 mL). In the final resuspension step, all *n* extracts received resuspension solvent, followed by vortex mixing for 30 seconds and centrifugation at 3000 x g for 10 min at 4 °C. The resulting supernatants were transferred to the collection vial. All solvents, solutions and extracts were kept of wet ice throughout the resuspension procedure.

**Figures S1** and **S2** provide an overview of each SPE-based fractionation procedure, including: sample resuspension procedures; volumes and types of solvents or solutions used, and; fractions resulting from each stage.

Fractions eluted from SPE cartridges were divided into equivolume aliquots, such that each contained metabolite quantities equivalent to ca. 20 *D. magna*. Aliquots for polar SPE extracts were transferred to polypropylene tubes and dried using a SpeedVac (Thermo Scientific) at 35 °C. Apolar fractions were transferred to 1.75 mL glass vials and dried under a gentle stream of gaseous nitrogen at room temperature. Dried fractions were stored frozen at -80 °C.

**Formatted:** Font: 9 pt, Font color: Auto

**Formatted:** Centered, Space Before: 0 pt, After: 0 pt

**Formatted:** Font: 9 pt, Do not check spelling or grammar, All caps

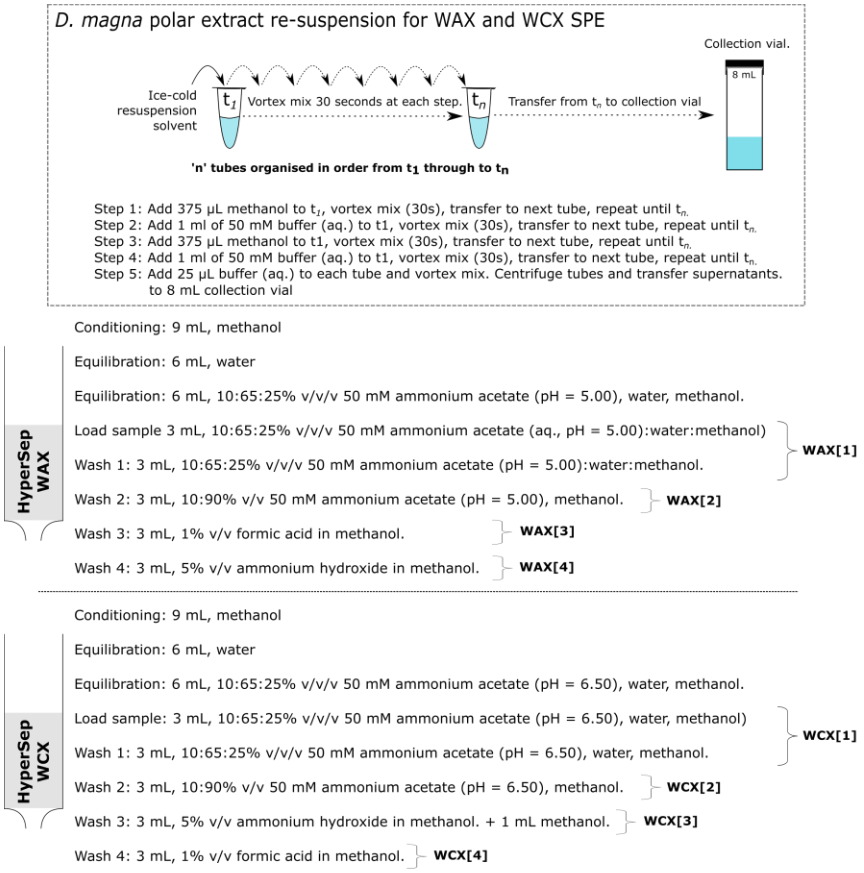


***Figure S1: Solid phase extraction-based fractionation of D. magna*** **polar extract** ***(WAX, weak anion-exchange; WCX, weak cation-exchange)***

**Formatted:** Font: 9 pt, Font color: Auto

**Formatted:** Centered, Space Before: 0 pt, After: 0 pt

**Formatted:** Font: 9 pt, Do not check spelling or grammar, All caps

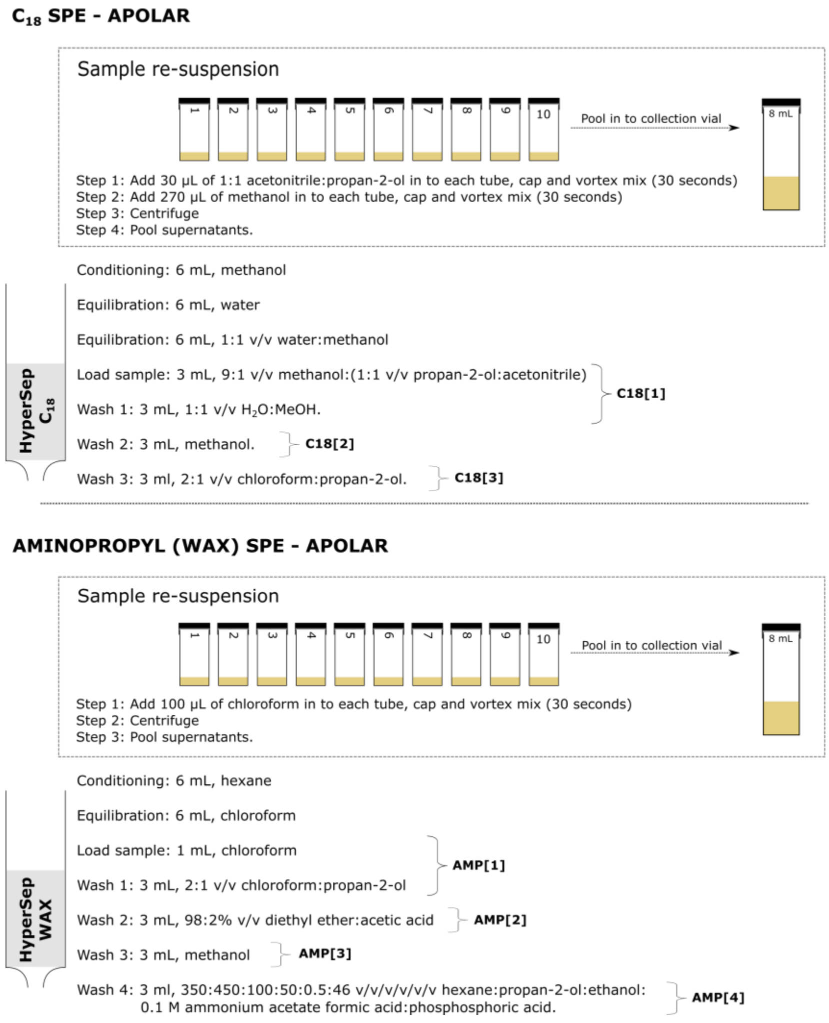


***Figure S2: Solid phase extraction-based fractionation of D. magna apolar extract (C18, a reversed phase-based fractionation procedure); AMP, a weak anion-exchange-based fractionation procedure***

**Formatted:** Font: 9 pt, Font color: Auto

**Formatted:** Centered, Space Before: 0 pt, After: 0 pt

**Formatted:** Font: 9 pt, Do not check spelling or grammar, All caps

# 1.8 DMA (U)HPLC-HRMS(/MS), DI-HRMS(/MS$^n$) and LC fractionation

## 1.8.1 (U)HPLC-HRMS(/MS)

### 1.8.1.1 (U)HPLC-HRMS(/MS) analytical setup and chromatographic methods

Three distinct (U)HPLC-HRMS(/MS) methods were used for untargeted metabolomics analysis of metabolite extracts (‘crude’ extracts) and associated SPE fractions, derived from DMA experimental samples. All chromatographic separations were performed using an Ultimate 3000 liquid chromatograph with in-line degasser, column oven, and fraction collection-enabled autosampler modules (system ‘FC-LCsys’ in **Table S6**; 20 microliter loop and maintained at 10 °C). A Q Exactive mass spectrometer, fitted with a heated electrospray ionisation source, was used for high resolution-full scan (HRMS) and -tandem (HRMS/MS) mass spectrometry analyses of eluted metabolites. All injections were performed using a custom-written injection procedure, in which 10 µL of sample was injected between a plug of mobile phase A.

Samples were resuspended (on wet ice) by vortex mixing for approximately 30 seconds with ice-cold resuspension solution: for Syncronis Phenyl-based RPLC-HRMS(/MS) this was 120 µL mobile phase A; for Accuore Amide-based HILIC-HRMS(/MS) analyses this comprised 24 µL aqueous buffer (i.e. 100 mM ammonium acetate + 2% v/v acetic acid in water), plus 96 µL acetonitrile and 20 µL methanol (see **Supplemental section 1.9.3.3** for resuspension details); for Accucore C30-based RPLC-HRMS(/MS), this was 120 µL of 1:1 v/v acetonitrile:propan-2-ol. Samples were centrifuged at 21885 x g and 4 °C for 10 minutes, followed by transfer of supernatants to glass HPLC vials with glass inserts.

Throughout (U)HPLC-HRMS(/MS) analyses of each DMA sample type, LC fractions were automatically collected. This was achieved by installing a passive tee-piece device between the LC column outlet and mass spectrometer inlet, such that approximately 30-50% of post-column eluent was passed towards the mass spectrometer, while the remainder returned towards the LC autosampler’s dual injector/fraction collector assembly. The latter automated the collection of LC fractions into individual wells of a deep well plate (Eppendorf, 700 µL deep-well polypropylene plates), with each LC fraction spanning a 20 second window. Fraction collection commenced 15 seconds into each (U)HPLC-HRMS(/MS) analysis to allow sufficient time for external rinsing of the injector/fraction collector assembly with needle wash solution. Across replicate analyses of each DMA sample type, under each analysis configuration i.e. positive mode HRMS, negative mode HRMS, positive mode HRMS/MS, and negative mode HRMS/MS, LC fractions were pooled into individual wells of a single fraction collection plate (see **Figure S3**). Upon completion of each (U)HPLC-HRMS(/MS) analysis of each DMA sample type, the corresponding LC fraction collection plates were either dried immediately using a Speedvac system (40 °C), or were briefly stored refrigerated (ca. 4 °C; heat sealed at 174 °C for 2 s with 20 µm EasyPierce foil, Thermo Scientific) until the Speedvac system was available.

HPLC-HRMS(/MS) analyses of apolar extract and associated SPE fractions were performed using an Accucore C30 LC column (C30) (2.1 x 100 mm, 2.6 µm solid-core particle, 150 Å;

**Formatted:** Font: 9 pt, Font color: Auto

**Formatted:** Centered, Space Before: 0 pt, After: 0 pt

**Formatted:** Font: 9 pt, Do not check spelling or grammar, All caps

Thermo Scientific). The column was maintained at 35 °C throughout, with metabolites eluted using a binary linear gradient supplied at 500 µL/min with the following profile (time in minutes, %B in parentheses): 0 (22%), 6 (60%), 14 (85%), 23 (100%), 26 (100%), 26.1 (22%), 30 (22%). Mobile phase A consisted of 49.5:49.5:1% v/v/v water:acetonitrile:buffer, mobile phase B consisted of 88:10:2% v/v/v propan-2-ol:acetonitrile:buffer, with buffer in each case comprising 99:1% v/v 100 mM ammonium acetate (aq.):acetic acid. Full-scan HRMS data were acquired with the following settings: 200-1200 m/z scan range; 70000 resolution (FWHM200m/z); 1e6 AGC target; 100 ms maximum ion injection time; 20 second chromatographic peak width. Data-dependent HRMS/MS scans were performed for the top-10 most abundant ions from each full-scan using the following parameters: minimum m/z fixed at 50 m/z; 35000 resolution ($FWHM_{200\ m/z}$); 1e5 AGC target; 1.3e4 intensity threshold; 1% underfill ratio; fixed first mass of 75 m/z; isolation window of 1 m/z and offset of 0 m/z; stepped normalized collision energies of 20, 30 and 40% in negative ionisation mode, and 25 and 30% in positive ionisation mode; dynamic exclusion of 8 seconds; apex trigger minimum of 4 seconds and maximum of 12 seconds; ‘pick others’ enabled; ‘peptide match’ off; ‘Exclude isotopes’ on; ‘charge exclusion’ off. The H-ESI source was operated as follows: sheath gas 20 arbitrary units (AU), auxiliary gas 5 AU, sweep gas 1 AU, spray voltage ±3 kV, S-lens 45%, capillary temperature 285 °C, and heater temperature 250 °C. S-lens RF level was set at 45%.

‘Polar extract’ and associated SPE fractions derived from DMA samples, underwent untargeted metabolomics analyses using two optimised (U)HPLC-HRMS(/MS) methods. The first, a reversed-phase UHPLC method, was based on a Syncronis Phenyl column (PHE) (2.1 x 100 mm (i.d. x L), 1.7 µm, Thermo Scientific) maintained at 40 °C. Metabolites were eluted from this column using a binary linear gradient supplied at 400 µL/min with the following profile (time in minutes, %B in parentheses): 0 (0%), 1.5 (0%), 22 (100%), 25 (100%), 26 (0%), 30 (0%). Mobile phases A consisted of 90:5:5% v/v/v water:methanol:buffer, while mobile phase B comprised 5:95% v/v buffer:methanol. In each case, buffer comprised an aqueous solution of 100 mM ammonium acetate adjusted to pH 5.8 using 100 mM acetic acid (aq.). The autosampler capillary was cleaned between injections using 100 µL of 80:20% v/v methanol:water solution (needle wash solution). With regards to HRMS(/MS) data acquisition, full-scan data were acquired between 100 and 1000 m/z at a resolution of 70000 ($FWHM_{200\ m/z}$) and with AGC target of 3e6, using a maximum ion injection time of 200 ms, S-lens RF 70%, and a single microscan. Data-dependent HRMS/MS data were collected for the top-3 most abundant ions in each full scan, with: ion intensity threshold ≥ 2e4; minimum m/z fixed at 50 m/z; 35000 resolution ($FWHM_{200m/z}$); 2e5 AGC target; 100 ms maximum ion injection time; 1 m/z isolation window (0 m/z offset); stepped normalized collision energies of 30 and 50%; 8 second dynamic exclusion; ‘pick others’ enabled; apex trigger with minimum of 2 seconds and maximum of 6 seconds; ‘exclude isotopes’ on; ‘charge exclusion’ off; ‘peptide match’ off. The H-ESI source was operated as follows: sheath gas 20 arbitrary units (AU), auxiliary gas 5 AU, sweep gas 0 AU, spray voltage +3.5 kV in positive ionisation mode and -3 kV in negative ionisation mode, S-lens 70%, capillary temperature 320 °C, and heater temperature 200 °C. Chromatographic peak width was set to 15 seconds for full scan and HRMS(/MS) analyses in both positive and negative ionisation modes

**Formatted:** Font: (Default) Arial

**Formatted:** Font: 9 pt, Font color: Auto

**Formatted:** Centered, Space Before: 0 pt, After: 0 pt

**Formatted:** Font: 9 pt, Do not check spelling or grammar, All caps

The second optimised (U)HPLC-HRMS(/MS) method used for untargeted metabolomics analysis of the 'polar extract' of DMA samples and the associated SPE fractions, was based on an Accucore Amide HILIC column (2.1 x 100 mm (i.d. x L), 2.6 µm solid core, Thermo Scientific). The column was maintained at 35 °C throughout analyses, with metabolites eluted using a binary linear gradient, supplied at 400 µL/min, with the following profile (time in minutes, %B in parentheses): 0 (0%), 1.5 (0%), 21 (100%), 25 (100%), 26 (0%), 30 (0%). Mobile phase A consisted of 90:5:5% v/v/v acetonitrile:water:buffer, while mobile phase B comprised 50:45:5% v/v/v acetonitrile:water:buffer. The buffer in each mobile phase consisted of 98:2% v/v 100 mM ammonium acetate (aq.):acetic acid. The autosampler capillary was rinsed with 100 µL of 80:20% v/v water:acetonitrile between samples. Full-scan, data-dependent HRMS(/MS), and H-ESI source parameters were as per the Syncronis Phenyl-based method, described above, with the exception that: chromatographic peak width was set to 25 seconds in positive ionisation mode and 15 seconds in negative ionisation mode; the top-5 ions from each full scan underwent HRMS/MS analysis; the apex trigger range was set with a minimum of 6 seconds and maximum of 15 seconds.

All mobile phases used in optimisation and DMA experiments were prepared using solvents and additives of HPLC-grade, or better. Each mobile phase was degassed in an ultrasonic water bath prior to use.

A directed data acquisition methodology was employed in the DMA experimental workflow for the acquisition of data-dependent (U)HPLC-HRMS(/MS) data. Sometimes referred to as Nearline (Neumann et al., 2013), a hybrid of offline and online fragmentation acquisition, the approach constitutes semi-automated acquisition techniques that can be executed with sufficient speed to minimize shifts in data acquisition, thereby achieving greater efficiency compared to offline fragmentation acquisition. See **Supplemental Section 1.8.1.2-1.8.1.3** for full details.

The collected LC fractions then resuspended underwent extensive DI-HRMS(/MS$^n$) analysis with a Thermo Fisher Obitrap Elite mass spectrometer.An initial DI-HRMS run being used to determine which features should be prioritised for fragmentation based on available sample volume and time constraints. See **Supplemental Sections 1.8.2** for full details.

#### 1.8.1.2 (U)HPLC-HRMS(/MS) data acquisition sequence

U)HPLC-HRMS(/MS) analysis of each DMA sample extract and SPE fraction followed a defined analysis procedure, as depicted in **Figure S3**. At the start of each sequence, quality assurance samples ('QA' samples) consisting of polar or apolar extracts of *D. magna* 'development samples' (the former used for Syncronis Phenyl and Accucore amide based methods, the latter used for the Accucore C30 method) were repeatedly injected to equilibrate the (U)HPLC-HRMS(/MS) system, followed by injection of one or two solvent blanks to minimise potential carryover. Full-scan (U)HPLC-HRMS analyses of DMA samples were then commenced, first in positive and then negative ionisation mode, for both the blank and *D. magna* DMA sample type under consideration. Here, data acquisition followed the same pattern in both ionisation modes, with five consecutive injections of the blank DMA sample and five consecutive injections of the

**Formatted:** Font: 9 pt, Font color: Auto

**Formatted:** Centered, Space Before: 0 pt, After: 0 pt

**Formatted:** Font: 9 pt, Do not check spelling or grammar, All caps

corresponding *D. magna* DMA sample, interspaced by two ‘QA’ samples and a solvent blank. A single sample blank was injected between blocks of positive and negative ionisation full-scan acquisitions. Additional solvent blank and ‘QA’ samples were also injected upon completion of full-scan acquisitions, prior to commencing data-dependent acquisition of HRMS/MS data (DDA-HRMS/MS) in both positive and negative ionisation modes. For DDA-HRMS/MS analyses, four consecutive injections of *D. magna* DMA sample were performed in each ionisation mode, with a single solvent blank injection between. To maximise the number of (U)HPLC-HRMS features selected for data-dependent HRMS/MS analysis, the first two and last two sample injections in each set of four DDA-HRMS/MS injections were configured with distinct inclusion and exclusion lists (see **Supplemental Section 1.8.1.3** for further details).

‘‘QA’ samples injected throughout the analysis sequence were used to ensure stability of (U)HPLC retention times and HRMS signals (in terms of ppm error and intensity). Solvent blanks, meanwhile, were used both to minimise potential carryover and to allow sufficient time for ‘directed acquisition’ data processing required to generate the inclusion and exclusion lists used in DDA-HRMS/MS acquisitions.

#### 1.8.1.3 (U)HPLC-HRMS(/MS) computational methods used for data acquisition

With a view to focussing on the most informative features to acquire fragmentation on, directed data acquisition was applied here to generate the inclusion and exclusion lists for the DDA-HRMS/MS injections. The approach was developed iteratively throughout the project, culminating in an implementation combining the following software packages: XCMS for feature picking (Smith et al., 2006), CAMERA (Kuhl et al., 2012) to annotate features based on their isotopes and adducts; msPurity (Lawson et al., 2017) for peak processing; the ‘Intervals’ R package for interval scheduling; and the Python package ‘deconrank’ (github.com/computational-metabolomics/deconrank) for deconvolution of features, ranking and assigning features for fragmentation.

The following key components, along with parameters – albeit with some variations as the workflow evolved throughout the project, were utilised: The raw data files were converted into mzML format using mzconvert (Proteowizard) into centroid configuration. Subsequently, XCMS was then used for feature detection using centWave peak picking algorithm. The parameters were typically set with ppm value of 5, subject to adjustment for specific assays. The signal-to-noise threshold was established at a minimum of 50, with a prefilter setting of (3, 100), integration set to 1, sigma at 3, mzdiff at 0.001, and peakWidth (low) set to 5 and peakWidth (high) between 20 and 40. For the grouping process in XCMS, the parameters included a minfrac between 0.5 and 0.6, mzwid set to 0.025, and bw set to 5. The blank and corresponding *Daphnia* grouped XCMS features were then compared and if a feature was observed in both blank and *Daphnia* samples, the intensity of the ~~d~~*Daphnia* samples was required to be 10 times that of the blank. The features were also filtered for the relative standard deviation of the retention time and for the ~~d~~*Daphnia* samples their intensity as well. A threshold of intensity of (e.g. 5000) was also applied to the *Daphnia* sample features. In addition, to further avoid acquiring fragmentation on blank features the exclusion list also included the top 100 most

**Formatted:** Font: Italic

**Formatted:** Font: Italic

**Formatted:** Font: 9 pt, Font color: Auto

**Formatted:** Centered, Space Before: 0 pt, After: 0 pt

**Formatted:** Font: 9 pt, Do not check spelling or grammar, All caps

intense (pre-XCMS) peaks within the scan of each mzML across multiple bins within chromatographic run (e.g. 0-90, 90-660, 660-1230 and 1230-1800).The R package CAMERA employed to annotate features based on their isotopes and adducts, used an error tolerance of 2 ppm. A pre-established adduct ranking, determined by the propensity of adduct formation to fragment (e.g., $[M+H]^+$ is ranked higher than $[M+Na]^+$), was used to select each feature from within each feature cluster that is expected to yield the most informative fragmentation data for describing that feature cluster. In cases where features are tied, the feature with the highest intensity is selected. Additionally, the features must elute within the same time range as determined by CAMERA's peak cluster groups. This process generates a list of features ranked in order of preference for fragmentation. The full list of ranked adducts are listed within the deconrank package (negative ionisation adducts: github.com/computational-metabolomics/deconrank/blob/master/deconrank/CAMERA_rules_NegFinal_PlusLi.csv; positive ionisation adducts: github.com/computational-metabolomics/deconrank/blob/master/deconrank/CAMERA_rules_PosFinal_PlusLi.csv)

The final ranked list of Daphnia features was subsequently organized into two inclusion lists for each assay. Features were iteratively added to these lists, ensuring that the minimum number of overlapping intervals of 10 was not surpassed. Furthermore, a corresponding exclusion list was established, comprising features to be excluded based on the detected blank features from XCMS, the top 100 most intense blank features (per chromatographic bin described above) and any features present on the alternate inclusion list.

Formatted: Font: 9 pt, Font color: Auto

Formatted: Centered, Space Before: 0 pt, After: 0 pt

Formatted: Font: 9 pt, Do not check spelling or grammar, All caps

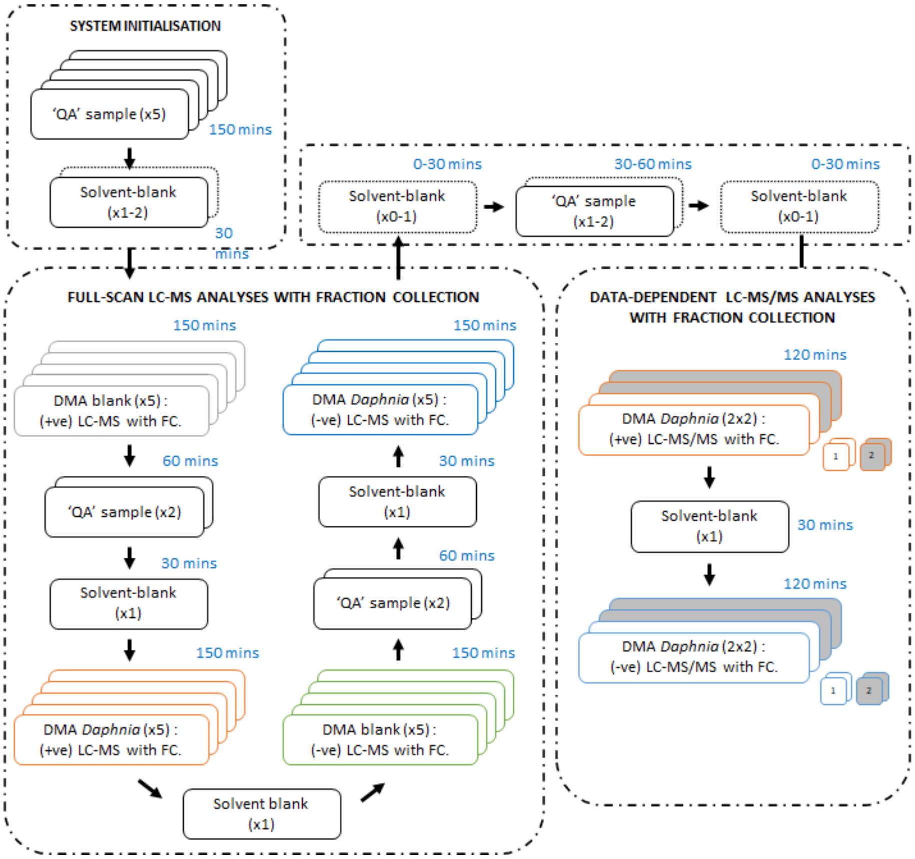


***Figure S3: Overview of the data acquisition workflow applied for (U)HPLC-HRMS(/MS) analysis and time-based fractionation of DMA samples.*** *"'QA' samples" were polar extracts (in the case of the Syncronis Phenyl and Accucore Amide methods) or apolar extractions (in the case of the Accucore C30 method) of D. magna development samples. "(+ve)" and "(-ve)" refer to positive and negative ionisation mode detection, respectively. "FC" means fraction collection. The numbered lists (1 and 2) in the box labelled "Data-dependent LC-MS/MS analysis with fraction collection" representing the inclusion and exclusion lists generated from the directed data acquisition (Nearline) approach.*

**Formatted:** Font: 9 pt, Font color: Auto

**Formatted:** Centered, Space Before: 0 pt, After: 0 pt

**Formatted:** Font: 9 pt, Do not check spelling or grammar, All caps

## 1.8.2 DI-HRMS(/MS$^n$)

### 1.8.2.1 Resuspension of LC fractions for DI-HRMS(/MS$^n$) analyses

LC fractions were resuspended prior to DI-HRMS(/MS$^n$) analyses. Here, 96-well deep well plates containing paired blank- and *Daphnia*-derived LC fractions (dry), were thawed over wet ice, followed by addition of 100 µL resuspension solution to each well. Resuspension solution was adjusted based on both the ionisation mode of DI-HRMS(/MS$^n$) analyses, and the origin of the LC fractions, as follows: 0.25% v/v formic acid in 80:20% v/v methanol:water for analysis of polar LC fractions under positive ionisation conditions; 4:1 v/v methanol:25 mM ammonium acetate (unmodified, aq.) for analysis of polar LC fractions under negative ionisation conditions; 2:1 v/v 7.5 mM ammonium acetate in methanol:chloroform for analysis of apolar LC fractions under positive and negative ionisation conditions. Well plates were then covered with self-adhesive foil and vortex mixed for 15 minutes, before being returned to wet ice. Well contents were subsequently transferred to individual polypropylene PCR tubes (0.5 mL, Sarstedt), from which 15 µL aliquots were in turn transferred to individual wells of a polypropylene 384-well microtiter well plate (Eppendorf twin.tec$^{TM}$) for DI-HRMS(/MS$^n$) analyses. Blank-derived LC fractions were each loaded into a single well on the 384-well plate. *Daphnia*-derived LC fractions meanwhile were each loaded into three wells of the 384-well plate (one well for DI-HRMS analysis, one well for DI-HRMS(/MS$^n$) analysis, and the third well serving as a backup). The 384-well plate was heat-sealed at 174 °C for 2 s using 20 µm EasyPierce$^{TM}$ foil (Thermo Scientific) and finally loaded into the cooled (10 °C) plate holder of a Triversa NanoMate (Advion) in preparation for analysis. Where immediate analysis was not possible, 384-well plates were stored refrigerated until the first possible opportunity for analysis. PCR tubes were stored refrigerated (+4-5 °C) short-term, before transfer to a laboratory freezer (-20 °C) for longer-term storage of remaining LC fraction aliquots.

### 1.8.2.2 DI-HRMS(/MS$^n$) analytical setup and data acquisition sequence

Resuspended LC fractions underwent DI-HRMS(/MS$^n$) analyses using a Triversa NanoMate coupled to an LTQ-Orbitrap Elite mass spectrometer (Thermo Fisher Scientific), i.e. nano-electrospray ionisation-based direct infusion-HRMS(/MS$^n$) analyses. Triversa operational parameters, configured within Chipsoft 8.3.3.1008, were consistent for all scan types: +1.7 kV (positive mode) or -1.7 kV (negative mode) spray voltage; 0.30 psi nitrogen gas pressure; 10 °C interface (plate holder) temperature; 10 µL injection volume; 0.5 µL post-sample air gap; and, with ‘headspace venting’ and ‘air gap before chip’ both enabled. Spray sensing was not used. Infusion flow rate was approximately 300 nL/min. The LTQ-Orbitrap Elite was operated in the profile acquisition mode with 240 °C ion transfer tube temperature and with S-lens RF level set to between 60 and 70%. An automated tuning procedure was applied prior to analyses, using manufacturer recommended calibration mixture. External mass calibration was also performed in accordance with manufacturer recommendations prior to all analyses.

The first step in DI-HRMS(/MS$^n$) analyses was acquisition of full scan HRMS data for all LC fraction types – both blank- and Daphnia-derived – present on a 384-well plate. For LC fractions generated using the Syncronis Phenyl-based UHPLC-HRMS(/MS) method, full scan data were acquired over 1.5 minutes (inclusive of a 24 second spray stabilisation delay) with the following

**Formatted:** Font: 9 pt, Font color: Auto

**Formatted:** Centered, Space Before: 0 pt, After: 0 pt

**Formatted:** Font: 9 pt, Do not check spelling or grammar, All caps

parameters: Orbitrap detection at 120000 resolution ($FWHM_{400m/z}$), 3e6 AGC target, 50-1000 m/z scan range, 1000 ms maximum ion injection time and single microscan. For fractions generated via HILIC-HRMS(/MS) or C30-HRMS(/MS) methods (i.e. based on HPLC columns 'AMD' and 'C30', respectively), full scan data were acquired as overlapping Selected Ion Monitoring (SIM) scan windows (while also having an initial 30 s spray stabilisation delay, during which full scan spectra were acquired). Data from SIM scans was subsequently 'stitched' together, using a bespoke informatics pipeline, to yield pseudo-full scan spectra (Southam et al., 2017). For HILIC-HRMS(/MS) LC fractions (generated using column 'AMD'), SIM scan windows were: 50-125, 105-180, 160-235, 215-290, 270-345, 325-400, 380-455, 435-510, 490-565, 545-620, 600-675, 655-730, 710-785, 765-840, 820-895, 875-950, 930-1005 and 985-1060 m/z. For C30-RPLC-HRMS(/MS) LC fractions (generated using column 'C30'), SIM scan windows were: 190-265, 245-320, 300-375, 355-430, 410-485, 465-540, 520-595, 575-650, 630-705, 685-760, 740-815, 795-870, 850-925, 905-980, 960-1035, 1015-1090, 1070-1145, and 1125-1200 m/z. All SIM scan data were acquired with Orbitrap detection, using a target resolution of 240000 ($FWHM_{400m/z}$) and AGC target of 5e5 (in both positive and negative ionisation modes). All other acquisition parameters were as per those used for DI-HRMS analyses of Syncronis Phenyl-derived LC fractions.

Full scan data, whether collected in full scan or SIM modes, was processed as described in **Supplemental Section 1.8.2.3**, below. Resulting inclusion lists were used to guide data-dependent tandem ($MS^2$) and multiple-stage ($MS^3$) mass spectral analyses of *Daphnia*-derived LC fraction components, during subsequent DI-HRMS(/$MS^n$) analyses. Herein, full scan data was acquired periodically throughout the infusion of each *Daphnia*-derived LC fraction aliquot, using the following parameters: Orbitrap detection at 120000 resolution ($FWHM_{400\ m/z}$) and 3e6 AGC target for LC fractions generated during Syncronis Phenyl-based RPLC-HRMS(/MS) analyses, and at 240000 resolution ($FWHM_{400\ m/z}$) with 5e5 AGC target for LC fractions generated from both HILIC-HRMS(/MS) and C30-RPLC-HRMS(/MS) analyses; scan range 50-1000 m/z, 50-1060 m/z and 190-1200 m/z for LC fractions generated during Syncronis Phenyl-based RPLC-HRMS(/MS), HILIC-HRMS(/MS), and C30-RPLC-HRMS(/MS) analyses, respectively; S-lens RF level between 60-70%; maximum ion injection time of 1000 ms; single microscan. For LC fractions generated during HILIC-HRMS(/MS) and C30-RPLC-HRMS(/MS) analyses, a data-dependent SIM scan followed each full scan (to better evaluate precursor ion purity), with the following parameters: Orbitrap detection at 120000 resolution ($FWHM_{400\ m/z}$), 5e5 AGC target, S-lens RF level between 60-70%; 10 microscans. Precursor ions selected from full scan events then underwent data-dependent (i.e. guided by an inclusion list) HCD-based fragmentation at three distinct normalised collision energy (NCE) values, specifically 20, 40 and 80%, using 0.1 ms activation time and with 50 m/z fixed lower scan-range limit. CID-based $MS^n$ analyses (effected using the linear ion trap) were also attempted for the same precursor ions, with fragmentation energy set to 35% NCE and with 30 ms activation time and 0.25 activation Q. Up to the top-3 most intense CID-induced $MS^2$ product ions, with m/z values < 98% of the original precursor ion, were targeted for CID-based $MS^3$ analyses. All $MS^2$ and $MS^3$ scan types were performed in triplicate, using: 1 Da isolation window; 2e5 AGC target; Orbitrap detection at 120000 resolution ($FWHM_{400\ m/z}$); 1000 ms maximum ion injection time; dynamic exclusion enabled; minimum signal requirement of either 5000 counts (for LC fractions generated using column 'PHE') or 500 counts (for LC fractions generated using methods 'AMD' and 'C30');

**Formatted:** Font: 9 pt, Font color: Auto

**Formatted:** Centered, Space Before: 0 pt, After: 0 pt

**Formatted:** Font: 9 pt, Do not check spelling or grammar, All caps

default charge state of 1. Dynamic exclusion settings were: repeat- and exclusion-time equal to the duration of DI-HRMS(/MS$^n$) method section, maximum exclusion list size of 500 and exclusion width of < ±2.5 ppm. See **Figure S4** for a summary of the data acquisition workflow.

#### 1.8.2.3 DI-HRMS(/MS$^n$) computational methods used for data acquisition

The same principle used for the directed data acquisition (i.e. Nearline (Neumann et al., 2013)) for (U)HPLC-HRMS(/MS) workflow was also applied to DI-HRMS(/MS$^n$) analysis to prioritise the most informative features to acquire fragmentation data on. The workflow was developed iteratively over the course of the project with a final implementation using the Python package ‘DIMSpy’ (github.com/computational-metabolomics/dimspy) for the peak processing or “feature picking”; adducts and isotope peaks putatively assigned using a modified version of the R package CAMERA (github.com/computational-metabolomics/cameraDIMS ) (Kuhl et al., 2012); and the Python package ‘deconrank’ (github.com/computational-metabolomics/deconrank) used to rank and select features for fragmentation, as well as to schedule the chosen features into inclusion lists. Visual scripting was then used to automatically create mass spectrometry instrument methods based on the inclusion lists generated.

As mentioned, the approach was iteratively improved as the project was developed but bellow describes broadly the workflow and parameters used for all assays:

Full-scan HRMS data generated during DI-HRMS analysis of resuspended LC fractions, was extracted in centroid format from associated .RAW data. For data acquired using SIM-type scan windows (i.e. LC fractions generated via HILIC-HRMS/MS and C30-RPLC-HRMS/MS methods), SIM spectra were stitched together to generate pseudo full scan spectra. Resulting peak matrices, each containing the m/z value, intensity and signal-to-noise ratio (as defined by manufacturer algorithm) values for all peaks in all full scan spectra (whether generated using full scan or SIM scan acquisition modes) of a given file, were then filtered. In each matrix, peaks with SNR ≥ 3 underwent hierarchical clustering using typically 5 ppm overlap threshold applied to peak m/z value (exact ppm used dependent on the dataset analysed). Replicate and %RSD filters were then applied followed by an absolute-intensity ≥ 5000. Extract-blank and corresponding *Daphnia* peak matrices were subsequently compared. Peaks present in the both matrices, as defined by a 5 ppm overlap window, were removed from the latter if their intensity was less than 10-times that in the former, yielding a ‘blank filtered’ matrix. The msPurity R package was then used to calculate the anticipated precursor ion purity of the feature (defined as precursor ion intensity / total intensity of all ions within +/- 0.5 m/z units).

To each of these matrices was applied the adapted version of the CAMERA package (compatible with DI-MS datasets), facilitating ion-type and isotope annotations. Each feature could thus have no annotation, an adduct annotation, an isotope annotation, or both. From each resulting feature cluster, the feature expected to yield the most informative fragmentation spectrum was selected based on pre-established adduct ranking rules (e.g. $M+H^+$ ranked above $M+Na^+$); in cases of ties, the most intense feature was selected. These selected features were designated as “first-tier features,” with all other cluster members classified as “second-tier features.”. All features (both first and second tiers) are then scored by peak intensity, full-scan

**Formatted:** Font: 9 pt, Font color: Auto

**Formatted:** Centered, Space Before: 0 pt, After: 0 pt

**Formatted:** Font: 9 pt, Do not check spelling or grammar, All caps

anticipated precursor ion purity, ion-type annotation and number of other associated ions sharing the same candidate neutral molecular mass. Weightings factors summed to a total of 1 and were set as follows: ion type 0.3, intensity 0.3, purity 0.2 and adducts in group 0.2. A subset of the second-tier features (e.g. 50%) being included on the inclusion list but with a penalised overall score (i.e. given a lower priority for fragmentation). Additionally, hard thresholds were used to remove features where the anticipated precursor ion purity was very low (e.g. <0.05) and removal of features where they strongly thought to be isotopes (e.g. [M+1]+ [M+2]+).

Xcalibur (v3.0.63) method files for all DI-HRMS(/$MS^n$) analyses were generated using an automated visual-scripting methodology to automate Windows graphical user interface (GUI) operations (using the python package pywinauto). All method files comprised two distinct sections, the first dedicated to HCD-based HRMS/MS data acquisition, and the second to ion-trap-facilitated CID $MS^3$ data acquisition. The time duration of each section, and therefore the overall method duration (inclusive of a 30 s spray stabilisation delay period), were adapted based on the length of the inclusion list supplied and according to defined limits to keep each acquisition run within approximately 30 minutes. Within these prescribed limits, section 1 duration (i.e. HCD-based HRMS/MS acquisition) was set in accordance with: length of inclusion list multiplied by 10 s (duration required for triplicate analysis at three %NCE values). Duration of section 2 was set in accordance with: length of inclusion list multiplied by 0.33 (i.e. 33% of peaks considered) multiplied by 12 s (duration required for triplicate analysis at single NCE value via ion-trap).

**Formatted:** Font: 9 pt, Font color: Auto

**Formatted:** Centered, Space Before: 0 pt, After: 0 pt

**Formatted:** Font: 9 pt, Do not check spelling or grammar, All caps

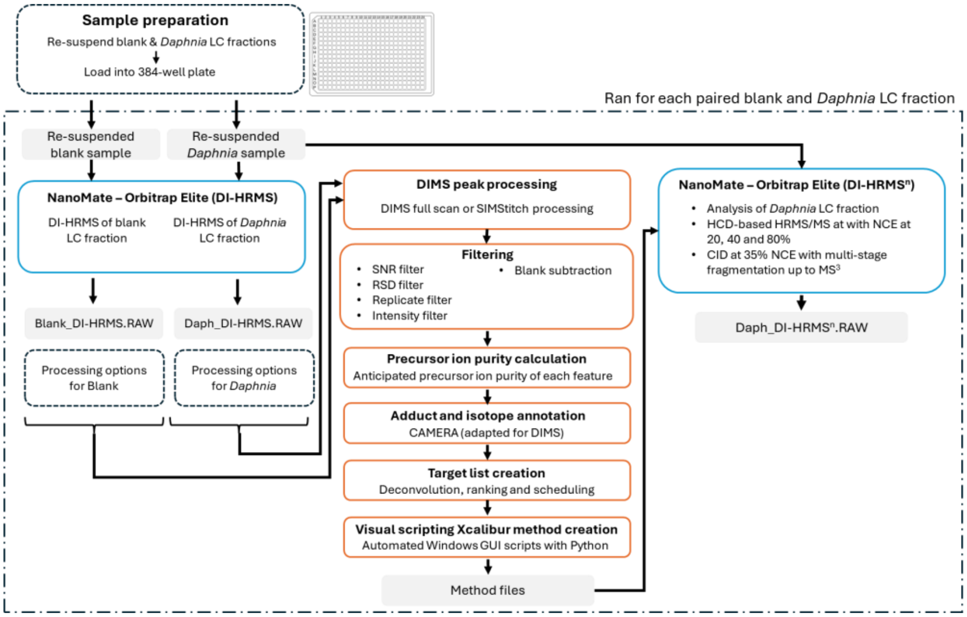


***Figure S4: Overview of the data acquisition workflow applied for DI-HRMS(/MS$^n$) analysis of the DMA re-suspended LC fractionation samples.*** *Demonstrating how the re-suspended LC fractionation samples were analysed by DI-HRMS(/MS$^n$). First DI-HRMS (Orbitrap Elite) analysis is performed on the paired re-suspended blank and Daphnia LC fractions to generate Thermo MS RAW files for each sample. These files are then peak processed with the resulting peak matrices filtered. Then the precursor ion purity is calculated and adduct and isotopes are annotated for the filtered features. A target list of features is then created prioritising features that will be the most informative to obtain fragmentation spectra on within restricted time limits for the acquisition. The target list of features is then used as input to generate Xcalibur methods files (the generation of which was automated using visual scripting with Python). The method files are then used as input for the analysis of the corresponding Daphnia LC fraction for both HCD-based HRMS/MS and CID-based multi-stage fragmentation.*

**Formatted:** Font: 9 pt, Font color: Auto

**Formatted:** Centered, Space Before: 0 pt, After: 0 pt

**Formatted:** Font: 9 pt, Do not check spelling or grammar, All caps

## 1.9 (U)HPLC-HRMS(/MS) method optimisation

Two (U)HPLC-HRMS(/MS) methods were optimized for analysis and (U)HPLC-based fractionation of DMA *D. magna* ‘polar extracts’ and associated SPE fractions. The first, a reversed-phase liquid chromatography (RPLC) method, was based on a Syncronis Phenyl column (2.1 x 100 mm, 1.7 µm; Thermo Scientific). The second, a hydrophilic interaction liquid chromatography (HILIC) method, was established using an Accucore Amide column (2.1 x 100 mm, 2.6 µm; Thermo Scientific).

(U)HPLC-HRMS(/MS) method optimisation sought to maximise the number and chromatographic distribution of ‘reproducibly detectable metabolic features’ (**RDMFs**) recorded in WAX and WCX SPE fractions, derived from polar extracts of *Daphnia magna* “development samples” (see **Supplemental Section 1.5**), under both positive and negative ionisation conditions. RDMFs were defined as any (U)HPLC-HRMS feature that, across technical replicate injections, had percent relative standard deviation (%RSD) ≤ 15% with respect to intensity and ≤ 3% with respect to retention time, and that were not annotated as potential isotopes, adducts or background ions during data processing, i.e. after background filtering and spectral deconvolution. In so doing, the goal was to maximise the number of metabolites for which HRMS/MS data could be collected during (U)HPLC-HRMS/MS analysis of DMA samples, whilst also ensuring that (semi-)purified metabolites could be reproducibly and concurrently fractionated (i.e. collected into well plates) for downstream, in-depth direct infusion-HRMS$^n$ analyses as part of the DMA analytical workflow (see **Supplemental Section 2.1** for details).

**Formatted:** Font: (Default) Arial

**Formatted:** Font: (Default) Arial

To rationalise the number of optimisation experiments performed, SPE fractions were combined based on presumed overlap in the physicochemical properties of their constituent metabolites. Fractions WAX[1], WAX[2], WCX[3] and WCX[4] were combined to generate sample type ‘WAX12WCX34’, whose constituents were expected to be polar-to-moderately polar, including some with (inducible) cationic structural moieties (e.g. amines). Fractions WAX[3], WAX[4], WCX[1] and WCX[2], meanwhile, were combined to yield “WCX12WAX34” containing polar-to-moderately polar metabolites, some of which having anionic structural moieties (e.g. carboxy groups, sulfates etc). *Daphnia*-derived SPE fraction pools are hereafter referred to as ‘DAX12DCX34’ and ‘DCX12DAX34’, while corresponding extraction blank SPE fractions are termed ‘BAX12BCX34’ and ‘BCX12BAX34’.

Each phase of method optimisation used SPE fractions prepared from distinct sets of *D. magna* “development samples”. Hence, RDMF counts were comparable within an optimisation experiment, wherein a single set of “development samples” were used, but not necessarily between optimisation experiments where different sets may have been used.

**Formatted:** Font: 9 pt, Font color: Auto

**Formatted:** Centered, Space Before: 0 pt, After: 0 pt

**Formatted:** Font: 9 pt, Do not check spelling or grammar, All caps

**Table S6**: Liquid chromatography systems used for (U)HPLC-HRMS(/MS) method optimisation

| System identifier(2)(3) | Component | Additional details |
|---|---|---|
| RS-LCsys1 | Ultimate$^{TM}$ SRD-3600<br>Ultimate$^{TM}$ HPG-3400RS<br>Ultimate$^{TM}$ TCC-3000RS<br>Ultimate$^{TM}$ WPS-3000TRS | In-line degasser<br>High-pressure binary gradient pump<br>Thermostatted column compartment<br>Temperature controlled and fitted with 100 µL stainless steel split loop and 6-port high pressure switch valve |
| RS-LCsys2 | Ultimate$^{TM}$ SR-3000<br>Ultimate$^{TM}$ LPG-3400RS<br>Ultimate$^{TM}$ TCC-3000RS<br>Ultimate$^{TM}$ WPS-3000TRS | In-line degasser<br>Quaternary pump<br>Thermostatted column compartment<br>Temperature controlled autosampler unit fitted with a 25 µL syringe, 25 µL stainless steel split loop and 6-port high pressure switch valve |
| FC-LCsys(1) | Ultimate$^{TM}$ SRD-3600<br>Ultimate$^{TM}$ DGP-3600RS<br>Ultimate$^{TM}$ FLM-3100<br>Ultimate$^{TM}$ WPS-3000TFC-ANALYTICAL | Six-channel in-line degasser<br>Dual-gradient pump unit with ternary solvent-mixing capabilities<br>Thermostatted flow control manager (column oven)<br>Temperature-controlled fraction collection-enabled autosampler fitted with 20 µL nanoViper pulled-loop, 2.4 µL needle, 6.4 µL bridge tubing, 25 µL syringe and 50 µL buffer tubing (all constructed from PEEK) |

(1) Injections on 'FC-LCsys' were performed using a custom-written 'User Defined Program', in which a 10 µL sample plug was injected at the centre of a loading solvent plug of volume 10 µL, with composition typically matching mobile-phase start conditions[MJ1] .

(2) All LC systems were configured, from injection loop through to mass spectral detector, with Viper (stainless-steel) or nanoViper (PEEK) capillaries.

(3) All autosampler modules, irrespective of analytical method applied, were operated at 10 °C, with samples loaded in clear-glass 300 µL fixed-insert autosampler vials, sealed using pre-assembled threaded caps with PTFE-silicone septa[MJ2]

**Formatted:** Font: 9 pt, Font color: Auto

**Formatted:** Centered, Space Before: 0 pt, After: 0 pt

**Formatted:** Font: 9 pt, Do not check spelling or grammar, All caps

**Table S7**: Mass spectrometer operational parameters for optimisation of (U)HPLC-HRMS(/MS) methods

<table>
<tr><th></th><th colspan="6">Mass spectrometer scan parameters</th><th colspan="10">MS source parameters</th></tr>
<tr><th rowspan="2">Method ID</th><th rowspan="2">System</th><th rowspan="2">Scan range (m/z)</th><th colspan="2">Full-scan</th><th colspan="2">MS/MS</th><th colspan="3">Gas (AU)</th><th rowspan="2">Aux. gas temp. (°C)</th><th rowspan="2">Spray voltage (kV)</th><th rowspan="2">S-lens level (%)</th><th rowspan="2">Ion transfer tube temp. (°C)</th><th colspan="3">Position</th></tr>
<tr><th>Resolut-ion*</th><th>AGC</th><th>Resolut-ion</th><th>AGC</th><th>Sheath</th><th>Aux.</th><th>Sweep</th><th>x-axis</th><th>y-axis</th><th>z-axis</th></tr>
<tr><td>Ph-CO-1</td><td rowspan="7">Q Exactive</td><td rowspan="7">100-1000</td><td rowspan="7">70000</td><td rowspan="7">3e6</td><td rowspan="7">NA</td><td rowspan="7">NA</td><td rowspan="7">40</td><td rowspan="7">15</td><td rowspan="7">0</td><td rowspan="7">300</td><td rowspan="7">Pos: +3.5, Neg: -3</td><td rowspan="7">70</td><td rowspan="7">320</td><td rowspan="7">+0.5</td><td rowspan="7">C</td><td rowspan="7">1.5</td></tr>
<tr><td>Ph-CO-2</td></tr>
<tr><td>Ph-CO-3</td></tr>
<tr><td>Ph-CO-4</td></tr>
<tr><td>Ph-FO-1</td></tr>
<tr><td>Ph-FO-2</td></tr>
<tr><td>Ph-FO-3</td></tr>
<tr><td>AA-CO-1</td><td rowspan="4">Q Exactive</td><td rowspan="10">100-1000</td><td rowspan="10">70000</td><td rowspan="10">3e6</td><td rowspan="10">NA</td><td rowspan="10">NA</td><td rowspan="4">40</td><td rowspan="4">15</td><td rowspan="10">0</td><td rowspan="3">200</td><td rowspan="4">Pos: +3.5, Neg: -3</td><td rowspan="10">70</td><td rowspan="10">320</td><td rowspan="10">+0.5</td><td rowspan="10">C</td><td rowspan="3">1.25</td></tr>
<tr><td>AA-CO-2</td></tr>
<tr><td>AA-CO-3</td></tr>
<tr><td>AA-FO-1*</td><td>300</td><td>1.5</td></tr>
<tr><td>AA-FO-1**</td><td rowspan="6">Q Exactive Focus</td><td rowspan="6">Pos: 40, Neg: 20</td><td rowspan="6">Pos: 15, Neg: 5</td><td rowspan="6">Pos: 300, Neg: 200</td><td rowspan="6">Pos: +3.5, Neg: -4</td><td rowspan="6">1.75</td></tr>
<tr><td>AA-FO-2</td></tr>
<tr><td>AA-FO-3</td></tr>
<tr><td>AA-FO-4</td></tr>
<tr><td>AA-FO-5</td></tr>
<tr><td>AA-FO-6</td></tr>
<tr><td>C30</td><td>Q Exactive</td><td>200-1200</td><td>70000</td><td>1e6</td><td>35000</td><td>1e5</td><td>40</td><td>15</td><td>1</td><td>250</td><td>Pos: +3, Neg: -3</td><td>45</td><td>285</td><td>+0.5</td><td>C</td><td>1.75</td></tr>
</table>

* parameters used for phases 2 and 3 of HILIC-HRMS(/MS) method optimisation

** parameters used for phase 4 of HILIC-HRMS(/MS) method and for DMA experiments.

**Formatted:** Font: 9 pt, Font color: Auto

**Formatted:** Centered, Space Before: 0 pt, After: 0 pt

**Formatted:** Font: 9 pt, Do not check spelling or grammar, All caps

**Table S8**: Liquid chromatography operational parameters for optimisation of (U)HPLC-HRMS(/MS) methods

| Column | Method ID | A-phase | B-phase | Gradient (%B / time / flow rate) | Column temp. (°C) | Syringe wash | Inj. vol. (µL) | LC system | AS temp. (°C) |
|---|---|---|---|---|---|---|---|---|---|
| Syncronis Phenyl | Ph-CO-1 | 0.1% v/v FA in H2O | 0.1% v/v FA in MeOH | 5/ 5/ 95/ 95/ 5/ 5% B at 0/ 1.5/ 22/ 25/ 26/ 30 min at 0.4 mL/min | 40 | 80:20% v/v MeOH:$H_2O$ | 10 | RS-LCsys1 | 10 |
| | Ph-CO-2 | 0.1% v/v FA in H2O | 0.1% v/v FA in ACN | | | | | | |
| | Ph-CO-3 | 95:5% v/v H2O:100 mM AmAc buffer (aq., pH 5.80) - final pH 5.8 | 5:95% v/v 100 mM AmAc buffer (aq., pH 5.80):MeOH | | | | | | |
| | Ph-CO-4 | 95:5% v/v H2O:100 mM AmAc buffer (aq., pH 5.80) - final pH 5.8 | 5:95% v/v 100 mM AmAc buffer (aq., pH 5.80):ACN | | | | | | |
| | Ph-FO-1 | 90:5:5% v/v/v H2O:MeOH:100 mM AmAc buffer (aq., pH 5.80) | 5:95% v/v 100 mM AmAc buffer (aq., pH 5.80):MeOH | 0/ 0/ 100/ 100/ 0/ 0% B at 0/ 1.5/ 22/ 25/ 26/ 30 min at 0.4 mL/min | | | 10* | FC-LCsys | |
| | Ph-FO-2 | | | 0 /0 / 70/ 100/ 100/ 0/ 0% B at 0 / 1.5/ 22/ 22.1/ 25/ 26/ 30 min at 0.4 mL/min | | | | | |
| | Ph-FO-3 | | | 0/ 0/ 100/ 100/ 100/ 0/ 0/ 0% B at 0/ 1.5/ 22/ 22.1/ 25/ 26/ 29.9/ 30 min at 0.3/ 0.3/ 0.3/ 0.4/ 0.4/ 0.4/ 0.4/0.3 mL/min | | | | | |

**Formatted:** Font: 9 pt, Font color: Auto

**Formatted:** Centered, Space Before: 0 pt, After: 0 pt

**Formatted:** Font: 9 pt, Do not check spelling or grammar, All caps

| Column | Method ID | A-phase | B-phase | Gradient (%B / time / flow rate) | Column temp. (°C) | Syringe wash | Inj. vol. (µL) | LC system | AS temp. (°C) |
|---|---|---|---|---|---|---|---|---|---|
| Accucore amide | AA-CO-1 | 90:10% v/v ACN:50 mM AmFm buffer (aq., pH 3.00) | 40:50:10% v/v/v ACN:$H_2O$:50 mM AmFm buffer (aq., pH 3.00) | 0/ 0/ 100/ 100/ 100/ 100/ 100/ 0/ 0/ 0% B at 0/ 1.5/ 21/ 23/ 23.01/ 25/ 25.01/ 26/ 30/ 30.1 min with 0.2/ 0.2/ 0.2/ 0.2/ 0.3/ 0.3/ 0.4/ 0.4/ 0.4/ 0.2 mL/min | 30 | 80:20% v/v H2O:MeCN | 10 | RS-LCsys1 | 10 |
| | AA-CO-2 | 90:10% v/v ACN:50 mM 50 mM AmAc buffer (aq., pH 6.63) | 40:50:10% v/v/v ACN:H2O:50 mM AmAc buffer (aq., pH 6.63) | | | | | | |
| | AA-CO-3 | 90:10% v/v ACN:50 mM 50 mM AmAc buffer (aq., pH 5.80) | 40:50:10% v/v/v ACN:H2O:50 mM AmAc buffer (aq., pH 5.80) | | | | | | |
| | AA-FO-1 | 90:5:5% v/v ACN:H2O:100 mM AmAc buffer (aq., pH 6.63) | 40:55:5% v/v/v ACN:H2O:100 mM AmAc buffer (aq., pH unmodified at approximately 6.9) | 0/ 0/ 100/ 100/ 100/ 100/ 100/ 0/ 0/ 0 / 0% B at 0/ 1.5/ 21/ 23/ 23.01/ 25/ 25.01/ 26/ 30/ 30.1/ 35 min with 0.2/ 0.2/ 0.2/ 0.2/ 0.3/ 0.3/ 0.4/ 0.4/ 0.4/ 0.2/ 0.2 mL/min | | | 10* | FC-Lcsys and RS-Lcsys2 | |
| | AA-FO-2 | 90:5:5% v/v/v ACN:H2O:100 mM AmAc (unmodified) | 40:55:5% v/v/v ACN:H2O:100 mM AmAc (aq., pH unmodified at approximately 6.9) | 0/ 0/ 100/ 100/ 0/ 0% B at 0/ 1.5/ 21/ 25/ 26/ 30 min at 0.4 mL/min | | | 5 | RS-Lcsys2 | |

**Formatted:** Font: 9 pt, Font color: Auto

**Formatted:** Centered, Space Before: 0 pt, After: 0 pt

**Formatted:** Font: 9 pt, Do not check spelling or grammar, All caps

| Column | Method ID | A-phase | B-phase | Gradient (%B / time / flow rate) | Column temp. (°C) | Syringe wash | Inj. vol. (µL) | LC system | AS temp. (°C) |
|---|---|---|---|---|---|---|---|---|---|
| | AA-FO-3 | | 60:35:5% v/v/v ACN:H2O:100 mM AmAC (aq., pH unmodified at approximately 6.9) | | | | | | |
| | AA-FO-4 | 90:5:5% v/v/v ACN:H2O:100 mM AmAc (pH 5.8) | 40:55:5% v/v/v ACN:H2O:100 mM AmAc (pH 5.8) | | | | | | |
| | AA-FO-5 | | 60:35:5% v/v/v ACN:H2O:100 mM AmAc (pH 5.8) | | | | | | |
| | AA-FO-6 | 90:5:5% v/v/v ACN:H2O:100 mM AmAc + 2% HOAc | 60:35:5% v/v/v ACN:H2O:100 mM AmAc + 2% HOAc | | | | | | |
| Accucore $C_{30}$ | C30-Eval | 10 mM AmFm in 50% ACN + 0.1% FA | 2 mM AmFm in ACN:IPA:H2O 10:88:2% (v/v/v) + 0.02% FA | 22/60/85/100/100/22/22% B at 0/6/14/23/26/26.1/30 minutes at 0.4 mL/min | 35 | 47.5:47.5:5% v/v/v ACN:IPA:H2O | 10* | FC-LCsys | 10 |

* 20 µL injection in which 10 µL of sample is injected between a loading solvent plug (mobile phase A).



**Formatted:** Font: 9 pt, Font color: Auto

**Formatted:** Centered, Space Before: 0 pt, After: 0 pt

**Formatted:** Font: 9 pt, Do not check spelling or grammar, All caps

## 1.9.1 Sample preparation for (U)HPLC-HRMS(/MS) method optimisation

SPE fraction pools used for (U)HPLC-HRMS/MS method optimisation were resuspended to a total volume of 100 µL. The resuspension solution used was equivalent in composition to the (U)HPLC gradient start conditions, unless otherwise specified. In practice, all SPE fractions to be combined were thawed and maintained on wet-ice throughout the resuspension process (see **Figure S5**, below). Thereafter, 100 µL of ice-cold resuspension solution was added into the microfuge tube containing the SPE fraction with lowest elution order number within a given pool, e.g. DAX[1] in 'DAX12DCX34' and DCX[1] in 'DCX12DCX34'. The tube was then vortex mixed for 30 seconds, before centrifugation at 21885 x *g* and 4 °C for 5 mins. The resulting supernatant was entirely transferred to the next highest-numbered fraction, again followed by vortex mixing, centrifugation and transfer – a procedure repeated until reaching the final fraction within a pooling group. Pooled SPE fraction samples, i.e. WAX12WCX34 and WCX12WAX34, were finally centrifuged for 10 minutes at 4 °C and 21885 x *g*, before transfer to 300 µL clear-glass autosampler vials for analysis.

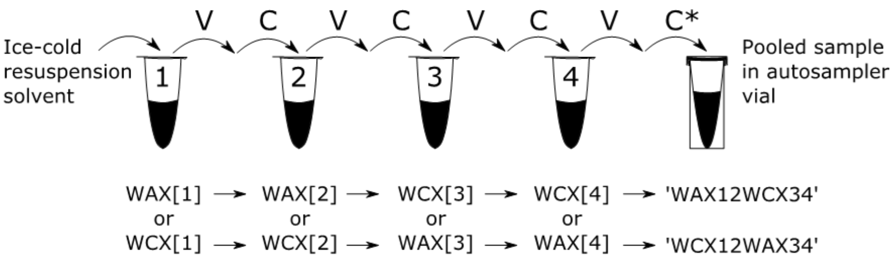


***Figure S5: Sample preparation for (U)HPLC-HRMS(/MS) method optimisation**. V: vortex mix, C: centrifuge, C*: extended centrifugation period, prior to loading in to autosampler vial.*

Quality assurance (QA) samples were prepared by resuspending thawed polar extracts of *D. magna* development samples (see **Supplemental Section 1.5**, above) in 100 µL of ice-cold resuspension solution. QA samples were vortex mixed for 30 s and then centrifuged for 10 min at 21885 x g and 4 °C. The resulting supernatant was transferred to a glass autosampler vial for analysis. Extraction blank 'QA' samples were likewise prepared. *D. magna* QA samples were injected repeatedly at the start of each (U)HPLC-HRMS(/MS) analysis sequence to initialise the LC-MS system, and then intermittently throughout each sequence to monitor system performance. Extract blank QA samples were likewise injected, generally towards the start and end of each sequence, to monitor the extent of LC-MS background signal.

**Formatted**

**Formatted:** Font: 9 pt, Font color: Auto

**Formatted:** Centered, Space Before: 0 pt, After: 0 pt

**Formatted:** Font: 9 pt, Do not check spelling or grammar, All caps

## 1.9.2 Optimisation of a Syncronis Phenyl-based reserved phase UHPLC-HRMS(/MS) method for untargeted analysis of DMA ‘polar’ extracts and associated SPE fractions

### 1.9.2.1 Phase one

Phase one of method optimisation, termed “coarse optimisation” (CO), for the Syncronis Phenyl-based RPLC method, involved exploration of the impact of different organic solvent types and mobile phase modifiers on the number and chromatographic distribution of RDMFs. Four methods were trialled. Methods ‘Ph-CO-1’ and ‘Ph-CO-2’ used acetonitrile as organic solvent, while methods ‘Ph-CO-3’ and ‘Ph-CO-4’ used methanol. Methods ‘Ph-CO-1’ and ‘Ph-CO-4’ included 0.1% v/v formic acid as mobile phase modifier, whereas methods ‘Ph-CO-2’ and ‘Ph-CO-3’ included 5 mM ammonium acetate buffer (5 mM final concentration, aqueous buffer pH 5.8) in each mobile phase. All other chromatography and mass spectrometry settings were kept constant across methods, as summarised in **Tables S7** and **S8**.

Samples DAX12DCX34 and DCX12DAX34 were analysed by consecutive quadruplicate injection in positive ionisation mode, and by consecutive triplicate injection in negative ionisation mode. Between each sample type, a single injection of corresponding blank SPE fraction pool was performed, followed by an injection of a *D. magna* ‘QA’ sample.

### 1.9.2.2 Phase two

Phase two of optimising the Syncronis Phenyl-based RPLC method involved fine-tuning of the eluent gradient shape and flow rate of the best-performing method from the first phase of optimisation, method ‘Ph-CO-3’. Herein, the primary objective was to maximise the chromatographic spread of RDMFs across the available chromatographic elution time to maximise the purity of DMA LC fractions while minimising HRMS(/MS) spectral complexity.

Method ‘Ph-CO-3’ was updated to use a lower initial percent of mobile phase B: 0/0/100/100/0/0% B at 0/1.5/22/25/26/30 min, using 400 µL/min flow rate throughout. This updated method was termed ‘Ph-FO-1’. From this reference method, two additional methodological modifications were explored, the first (‘Ph-FO-2’) utilising a two-step-binary gradient elution profile (0/0/70/100/100/0/0% B at 0/1.5/22/22.1/25/26/30 min) at the original 400 µL/min flow rate, and the second (‘Ph-FO-3’) implementing a reduced mobile phase flow rate of 300 µL/min throughout the gradient ramp period (minutes 0 to 22), before returning to 400 µL/min for the remainder of the elution period.

For each method, quintuplicate injections were performed of both DAX12DCX34 and DCX12DAX34 sample types. Corresponding blank SPE fraction pools were injected in triplicate after their respective *Daphnia* equivalents, followed by a single or duplicate injection of a *D. magna* ‘QA’ sample. This analysis order was applied first in positive and then in negative ionisation modes.

**Formatted:** Font: 9 pt, Font color: Auto

**Formatted:** Centered, Space Before: 0 pt, After: 0 pt

**Formatted:** Font: 9 pt, Do not check spelling or grammar, All caps

### 1.9.3 Optimisation of an Accucore Amide-based hydrophilic interaction liquid chromatography (HILIC)-HRMS(/MS) method for untargeted analysis of DMA ‘polar’ extracts and associated SPE fractions

#### 1.9.3.1 Phase one

Phase one of optimising an Accucore Amide-based HILIC-HRMS(/MS) method for DMA involved exploration of the impact, on RDMF counts and distributions, of altering the pH and additive type of the aqueous buffer used to prepare the binary-gradient mobile phases. As summarised in **Table S8**, method ‘AA-CO-1’ used a 50 mM ammonium formate buffer (aq.) adjusted to pH 3.0 through dropwise addition of 50 mM ammonium formate into 50 mM formic acid (aq.). Method ‘AA-CO-2’ used a 50 mM ammonium acetate solution (aq.), pH unmodified (approximately pH 6.6). Method ‘AA-CO-3’, meanwhile, used a 50 mM ammonium acetate buffer (aq.) adjusted to pH 5.8 using 50 mM acetic acid (aq.). In all methods, mobile phase ‘A’ comprised 90:10% v/v acetonitrile:aqueous buffer, while mobile phase ‘B’ comprised 40:50:10% v/v/v acetonitrile:water:aqueous buffer.

Owing to limited solubility of dried SPE fractions in 100% mobile phase A, a modified version of the SPE-fraction resuspension procedure described in **Supplemental Section 1.9.1** (above) was applied. At each stage of the resuspension process (i.e. for each SPE fraction included in an SPE fraction pool), the following steps were applied: transfer resuspension solution into the microfuge tube containing the dried SPE fraction; vortex mix for 60 seconds; sonicate twice for 10 seconds; vortex mix for a further 30 seconds; sonicate for 30 seconds; vortex mix for a 30 seconds and; finally, centrifuge for 5 minutes at 21885 x g and 4 °C. This procedure is hereafter referred to as ‘AA-Resus-1’. All sonication steps were performed using an ultrasonic bath operated at ambient temperature. Total resuspension volume was 100 µL.

Samples DAX12DCX34 and DCX12DAX34 were each analysed in quadruplicate, in both positive and negative ionisation modes, followed by corresponding extract blank samples. One or two *D. magna* ‘QA’ samples were injected after the latter.

#### 1.9.3.2 Phase two

Phase two of Accucore Amide-based HILIC-HRMS(/MS) method optimisation sought to evaluate the impact of sample resuspension on the number and diversity of RDMFs. Hence, three SPE fraction resuspension strategies were trialled. The first, “AA-Resus-1”, followed the procedure outlined in phase one of HILIC-HRMS(/MS) method optimisation (**Supplemental Section 1.9.3.1**, above) – this served as the reference method. The second, “AA-Resus-2”, replicated the procedure for “AA-Resus-1” but used a pre-mixed solution of 80:20% v/v acetonitrile:dimethyl sulfoxide (DMSO) for resuspension. The final resuspension method evaluated, ‘AA-Resus-3’, used two solutions for resuspension, the first being 50 µL ice-cold 100 mM ammonium acetate (pH unmodified) and the second being 50 µL ice-cold acetonitrile. For both, the resuspension procedure followed that of “AA-Resus-1”, with the final supernatants combined. For all resuspension methods evaluated, the resuspended sample was vortex mixed, centrifuged (4 °C for 10 minutes at 21885 x g) and transferred into an autosampler vial, prior to analysis. Resulting SPE fraction pools were analysed using system ‘LC-FCsys’, (see TableS6,

**Formatted:** Font: 9 pt, Font color: Auto

**Formatted:** Centered, Space Before: 0 pt, After: 0 pt

**Formatted:** Font: 9 pt, Do not check spelling or grammar, All caps

above), in combination with a Q Exactive mass spectrometer. Chromatographic conditions matched those for method ‘AA-CO-3’, save for an additional 5-minute hold at 100% mobile phase A that was added to the end of the method for re-equilibration purposes – hereafter termed method ‘AA-FO-1’. Mobile phase ‘A’ was used as loading solvent for the plugged sample injection procedure.

All SPE fraction pools prepared using a given resuspension protocol were analysed consecutively, in both positive and then negative ionisation modes. DAX12DCX34 and DCX12DAX34 fraction pools were injected in triplicate, each followed by duplicate injection of corresponding BAX12BCX34 and BCX12BAX34 samples. *D. magna* QA samples were injected singly, or in duplicate, following each set of blank SPE fraction pool injections, to monitor and equilibrate the (U)HPLC-HRMS(/MS) system.

#### 1.9.3.3 Phase three

Phase three of optimizing an Accucore Amide-based HILIC method for DMA involved further evaluation of the impact of the SPE fraction resuspension procedure on RDMF counts and distributions. Method “AA-Resus-4” was based on “AA-Resus-3” but used 20 µL of 100 mM ammonium acetate buffer (aq., pH unmodified) for the first part of resuspension, and 80 µL acetonitrile for the second. Following each vortex mixing step depicted in **Figure S5**, above, the SPE fraction undergoing resuspension was centrifuged for 5 minutes at 21885 x *g* and 4 °C, prior to transfer of the resulting supernatant into the next tube. The final supernatants generated using both 20 µL 100 mM ammonium acetate buffer and 80 µL acetonitrile, were combined into a single tube. An additional 20 µL methanol was spiked into this tube to avoid phase separation, followed by vortex mixing, centrifugation (5 minutes at 21885 x g and 4 °C) and transfer of the supernatant into a glass autosampler vial.

For comparison, additional SPE fraction pool samples were prepared as per “AA-Resus-1” (see **Supplemental Section 1.9.3.2**, above) – the leading resuspension method from prior optimisation experiments.

Both DAX12DCX34 and DCX12DAX34 were each analysed in quadruplicate, followed by corresponding extract blanks (BAX12BCX34 and BCX12BAX34, respectively), in both positive and negative ionisation modes using methods ‘AA-FO-1’ (see **Supplemental Section 1.9.3.2**).

#### 1.9.3.4 Phase four

Phase four of optimizing an Accucore Amide-based HILIC-HRMS(/MS) method for DMA involved re-evaluating the method’s chromatographic conditions. This was undertaken due to substantial changes implemented for sample resuspension during earlier phases of method optimisation, which were anticipated to impact both the type and concentration of metabolites accessible through this method. A total of six chromatographic conditions were therefore trialled, as outlined in **Table S7** and **Table S8**, using system ‘LC-RSsys1’ (see **Table S6**) in combination with a Q Exactive Focus mass spectrometer. Method ‘AA-FO-1’ (see **Supplemental Section 1.9.3.2**) was transferred to this system with a 10 µL injection volume defined (no longer using a plugged solvent injection procedure). Methods ‘AA-FO-2’ through ‘AA-FO-6’ all used a 5 µL

Formatted: Font: 9 pt, Font color: Auto

Formatted: Centered, Space Before: 0 pt, After: 0 pt

Formatted: Font: 9 pt, Do not check spelling or grammar, All caps

injection volume. For methods ‘AA-FO-1’ and ‘AA-FO-2’, injection volume (10 versus 5 µL) and flow rate (200 versus 400 µL $min^{-1}$) were the sole technical differences between the methods. Method ‘AA-FO-3’ replicated ‘AA-FO-2’, except for a reduction in the aqueous content of mobile phase B (strong mobile phase) to 40% v/v. Methods ‘AA-FO-4’ and ‘AA-FO-5’ were replicas of methods ‘AA-FO-2’ and ‘AA-FO-3’, respectively, albeit with the aqueous buffer used for mobile phase preparation having been adjusted to pH 5.8 using 100 mM acetic acid. Method ‘AA-FO-6’ was likewise a replica of method ‘AA-FO-2’, albeit with the aqueous buffer used to prepare mobile phases comprising 100 mM ammonium acetate spiked with acetic acid to 2% v/v. Methods ‘AA-FO-[3-6]’ all also used a higher linear flow rate of 400 µL/min.

Across all methods, SPE fraction re-suspension followed closely the two-step procedure described for ‘AA-Resus-4’ (see **Supplemental Section 1.9.3.3**, above), differing only in that 24 µL of aqueous buffer and 96 µL of acetonitrile were used, followed by addition of 10 µL methanol for biphase disruption. Owing to failure to dissipate the resulting biphase in sample vials for methods ‘AA-FO-4 ‘, ‘AA-FO-5’ and ‘AA-FO-6’, it was necessary to spike in an additional 10 µL methanol into the SPE fraction pools.

### 1.9.4 Data processing for (U)HPLC-HRMS(/MS) method optimisation

Data files from (U)HPLC-HRMS(/MS) method optimisation were converted to .mzML format using msconvert (Proteowizard). XCMS was used to read and extract data from .mzML files and to perform feature detection using the centWave peak picking algorithm with the following parameters: ppm = 5, signal to noise threshold = 100, prefilter = (3, 100), sigma = 3, mzdiff = 0.001, peakWidth (low) = 5 and peakWidth (high) = 20. XCMS’s group.density function was then used to group metabolic features within and across sample groups, using the following parameters: ‘bw’ = 5, ‘minfrac’ = 0.6, ‘minsamp’ = 1, ‘mzwid’ = 0.025, ‘max’ = 50. Adduct and isotope annotations were assigned to features in the resulting data matrix using the CAMERA package, with the following functions and parameters applied: function ‘groupFWHM’ used ‘perfwhm’ = 0.6, and ‘sigma’ = 6; function ‘groupCorr’ used ‘cor_eic_th’ = 0.75, ‘calcIso’ = 0, ‘calcCiS’ = 0, ‘calcCaS’ = 0, ‘graphMethod’ = hcs, and ‘pval’ = 0.05; function ‘findIsotopes’ used ‘intval’ = maxo, ‘maxcharge’ = 3, ‘minfrac’ = 0.5, ‘maxiso’ = 4, ‘mzabs’ = 0.01, and ‘ppm’ = 5; function ‘findAdducts’ used ‘max_peaks’ = 100, ‘multiplier’ = 3, ‘mzabs’ = 0.015, and ‘ppm’ = 5. Spectral deconvolution was subsequently performed within Python as per **Supplemental Section 1.8.1.2.**

**Formatted:** Font: 9 pt, Font color: Auto

**Formatted:** Centered, Space Before: 0 pt, After: 0 pt

**Formatted:** Font: 9 pt, Do not check spelling or grammar, All caps

## 1.10 GC-EI-HRMS

Aliquots of the ‘polar extract’ and associated SPE fractions derived from DMA experimental samples, were analysed by gas chromatography-electron ionisation-high resolution mass spectrometry (GC-EI-HRMS). Prior to analysis, each sample underwent a two-step methoximation-silylation derivatization procedure, as follows: 1) add 50 µL of 20 mg/mL O-methoxyamine hydrochloride in pyridine (99.8%) to sample; 2) vortex mix for 30 seconds; 3) heat sample to 80 °C and maintain for 15 minutes; 4) bring sample to room temperature; 5) add 50 µL of N-methyl-N-trimethylsilyltrifluoroacetamide (MSTFA) containing 1% trimethylchlorosilane (TMCS); 6) vortex mix for 20 seconds; 7) heat sample to 80 °C and maintain for 15 minutes; 8) bring sample to room temperature; 9) spike 5 µL of n-alkane retention index solution comprising 0.6 mg/mL of each of decane, dodecane, pentadecane, nonadecane, docosane, pentacosane and nonacosane, in pyridine; 10) vortex mix for 20 seconds; 11) centrifuge at 15800 x *g* and room temperature for 15 minutes; 12) transfer 50 µL of supernatant to 300 µL fixed-insert, amber-glass GC automsampler vial and crimp seal using pre-assembled caps with PTFE septum. All heating was performed using a Reacti-Therm I heat block (Thermo Scientific). All solutions were handled with positive displacement pipettes fitted with disposable glass, microdispenser capillaries. Between transfer procedures, pipettes were rinsed with *n*-hexane and wiped with lint-free tissue paper to minimise cross contamination.

Aliquots of crude polar extracts of *D. magna* ‘development samples’ were prepared according to the above procedure and used for GC-EI-HRMS system equilibration and as intra-study QC samples.

GC-EI-HRMS analyses were performed using a Thermo Scientific TriPlus RSH autosampler coupled, via a wool-lined heated injection port (280 °C; 1:100 split ratio), to a Thermo Scientific TRACE 1310 gas chromatograph and, in turn, to a Q Exactive GC-Orbitrap mass spectrometer, via an Extractabrite electron ionisation/chemical ionisation source. Samples were injected at a volume of 1 µL and separated over a TG-5SilMS capillary column (30 m length x 0.25 mm internal diameter x 0.25 µm film thickness; fitted with 15 cm integrated, non-functionalised guard; selectivity comparable to 5% diphenyl/95% dimethylpolysiloxane columns) using helium as carrier gas (1 mL/min) and with the following temperature gradient elution program: 0/4/12.5/20.5/30 min at 70/70/320/320/70 °C. Column eluates were passed through an MS transfer line (250 °C) in to a heated electron-ionization (EI) source (230 °C), wherein ionisation was induced through interaction with an electron ‘beam’ operated at 70 eV. Positive-mode, full-scan (50-650 m/z) mass spectral data acquisition was performed at 60000 resolution (FWHM 200 m/z) and with AGC target of 1e6, using a dedicated GC-Orbitrap mass spectrometer. Data were not acquired during the first five minutes of elution. Lock-masses of 207.0324 m/z, 281.0511 m/z and 355.0699 m/z were included for internal calibration purposes. The mass spectral system was externally mass-calibrated < 24 hours prior to data acquisition.

Xcalibur software was used to construct and thereafter automate the GC-EI-HRMS analysis sequence. Prior to analysis of DMA SPE fractions, five QA sample injections were performed for the purpose of system stabilisation. Thereafter, DMA SPE fractions were analysed at random, with QA samples injected every fourth sample for system monitoring purposes.

**Formatted:** Font: 9 pt, Font color: Auto

**Formatted:** Centered, Space Before: 0 pt, After: 0 pt

**Formatted:** Font: 9 pt, Do not check spelling or grammar, All caps

GC-EI-HRMS data were processed using TraceFinder v.4.1(beta), including peak-picking, deconvolution and spectral library lookup. Processing parameters were set as follows: accurate mass tolerance of 5 ppm; signal to noise ratio threshold of 10; minimum and maximum retention times bounds were 5.3 and 20.0 min, respectively; TIC intensity threshold of 100000; ion overlap window of 98%; ‘use all ions’ enabled; retention alignment window of 5 seconds and; library search type set to ‘normal’ with ‘golmdb’ selected.

Peak annotations were made through matching of full-scan mass spectra to the NIST 2014 HRAM and GOLMdb libraries, as well as to an internal GC-EI-HRMS library constructed under the same temperature programme as described above, using authentic chemical standards. Annotations did not take into consideration the retention time, nor indices, of the extracted mass spectral features due to software limitations. Quantifier ions were automatically selected via the TraceFinder software.

Annotations were filtered to retain only those with dot-product match scores > 700 (equivalent to the 0.7 dot product cosine threshold used elsewhere in this paper). The threshold has been previously reported as a general guide for considering an acceptable match, where 700-800 is considered a fair match, 800-900 a good match and greater than 900 considered a very good match (Garreta-Lara et al., 2016).



**Formatted:** Font: 9 pt, Font color: Auto

**Formatted:** Centered, Space Before: 0 pt, After: 0 pt

**Formatted:** Font: 9 pt, Do not check spelling or grammar, All caps

## 1.11 1D- & 2D-NMR

SPE fractions derived from DMA polar metabolite extracts were analysed by 1D- & 2D-NMR spectroscopy. *Daphnia* SPE fractions, with corresponding blanks, were thawed over wet ice for 10 minutes before addition of 60 µL of 0.1 M phosphate buffer (pH 7.0, 9:1% v/v HPLC-grade water:deuterium oxide) containing 0.5 mM trimethylsilylpropanoic acid (chemical shift calibrant). Tubes were vortex mixed for 30 seconds, followed by centrifugation at 15,000 rpm and 4 °C, for 10 minutes. For SPE fraction WCX[3], it was necessary to agitate the sample for 60 s in an ultrasonic bath to promote dissolution of the pelleted material, followed by centrifugation. Thereafter, 50 µL of each supernatant was transferred to an individual, clear-glass champagne vial (1.5 mL, Cronus) and stored capped (PTFE-silicone-PTFE, Cronus) in a laboratory refrigerator (5 ± 1 °C) before robot-assisted transfer into 1.7 mm NMR tubes (1.7 x 103.5 mm; SampleJet). Finally, samples were loaded and stored in a cooled (6 °C) NMR SampleJet autosampler for analysis. All NMR analyses were performed at the Biomolecular NMR facility Birmingham, United Kingdom, using a Bruker AVANCE III 600 MHz NMR spectrometer equipped with a 1.7 mm TCI-Cryoprobe (1H, 13C, 19F) and operated at a proton frequency of 600.13 MHz.

A combination of 1D and 2D-NMR experiments were used to study SPE fractions. Each fraction was initially profiled by 1D proton nuclear overhauser effect NMR spectroscopy (1D-$^{1}$H-NOESY). The 1D-$^{1}$H-NOESY pulse sequence (Bruker noesygppr1d pulse sequence) included water suppression and consisted of the following parameters: mixing time 10 ms, spectral width 11.98 ppm, number of points 32k, relaxation delay 4 s and number of scan 128. Total acquisition time per experiment amounted to 14 minutes.

To support metabolite annotation, 2D homonuclear $^{1}$H-$^{1}$H (2D-JRes and TOCSY) and heteronuclear $^{1}$H-$^{13}$C (HSQC) experiments were performed. For 2D $^{1}$H-$^{1}$H J-Resolved (J-Res) NMR experiments, data were acquired using 8 transients for 32 increments, each collected with 16k data points and with a spectral width of 12 ppm in the direct dimension F2 (chemical shift axis) and 50 Hz in the indirect dimension F1 (spin-spin coupling axis) for a total acquisition time of 24 minutes. 2D 1H-1H TOCSY spectra were acquired using a DIPSI-2 (Shaka et al., 1988) mixing sequence with the following parameters: 140 ms acquisition time, 65 ms mixing time, 11.98 ppm spectral width and relaxation delay 2s, number of scans 16 and 512 increments corresponding to a total acquisition time of 2 hours and 34 minutes. 2D 1H-13C Heteronuclear Single Quantum Coherence spectroscopy (HSQC) experiments (Davis et al., 1991) were carried out using a 65 ms acquisition time with GARP 13C decoupling and a 1.5 s relaxation delay. A total of 64 transients were averaged for each of the 256 increments resulting in an acquisition time of 3 hours and 35 minutes.

To shorten the acquisition time of total correlation spectroscopy (TOCSY) and HSQC experiments, a Non-Uniform Sampling (NUS) algorithm (Multi-dimensional Decomposition (Orekhov and Jaravine, 2011)) was applied. Here, only a subset of data points were acquired in a semi-randomised manner. For both experiment types, the amount of sparse sampling was set to 40%.

Automated tuning, matching and shimming was performed prior to data acquisition. Temperature gradients and radiation damping occasionally necessitated manual shimming.

**Formatted:** Font: 9 pt, Font color: Auto

**Formatted:** Centered, Space Before: 0 pt, After: 0 pt

**Formatted:** Font: 9 pt, Do not check spelling or grammar, All caps

Such were the difficulties in achieving an effective shim on the aforementioned NMR system, that polar-arm SPE fractions for the metabolite reference standard mixture were analysed using a Bruker AVANCE III HD 600 MHz IVDr validated NMR spectrometer, fitted with a room temperature double resonance broadband probe (Bruker 5mm BBI).

NMR data analysis was performed using the TopSpin software suite (v.3.5pl2, Bruker BioSpin Germany). For assignment of identities to spectral signals, data were compared to various NMR databases (SpinCouple (Kikuchi et al., 2016)), the Human Metabolome Database (Wishart et al., 2007), the Birmingham Metabolomics Library (Ludwig et al., 2012) and Biological Magnetic Resonance Data Bank (Hoch et al., 2022) and to the metabolite standard mixture components. For putatively annotated compounds, further experiments were performed involving spiking of reference compounds into solution.

**Formatted:** Font: 9 pt, Font color: Auto

**Formatted:** Centered, Space Before: 0 pt, After: 0 pt

**Formatted:** Font: 9 pt, Do not check spelling or grammar, All caps

## 1.12 DMA computational workflow overview

The DMA workflow predominantly consists of the Galaxy workflow, however annotations are also incorporated from external sources (i.e. mzCloud, GNPS workflows, NMR and GC-MS). See **Figure S6** for overview for the full overview of the computational workflow.

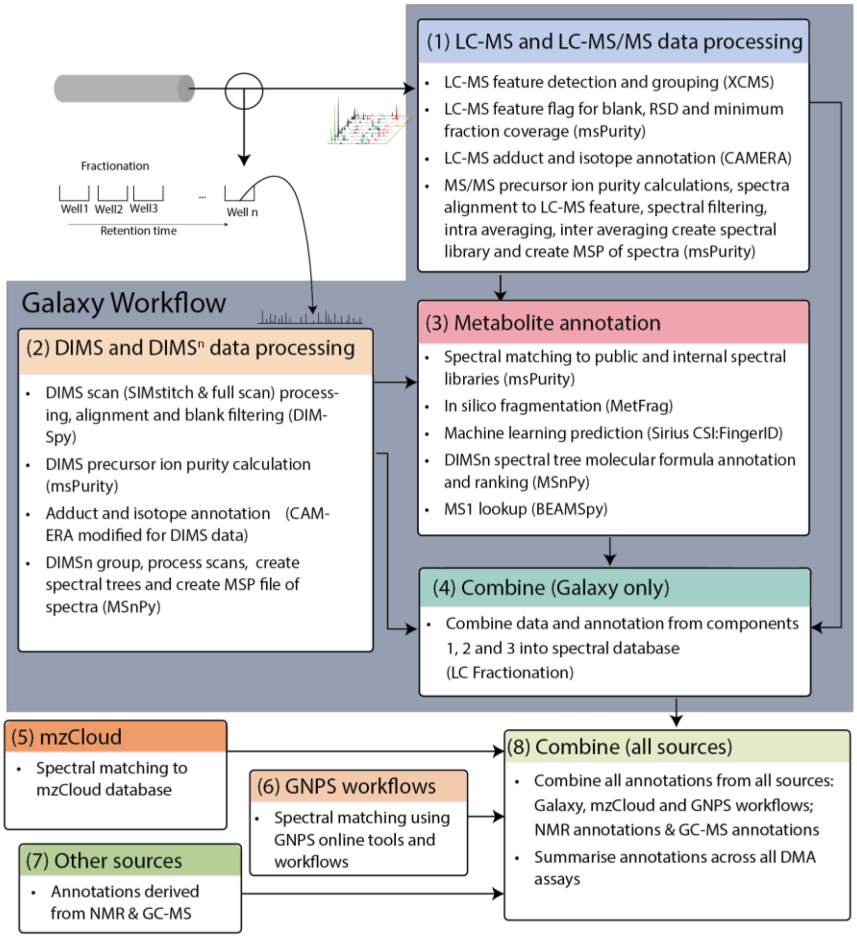


***Figure S6: Overview of computational analysis of DMA (U)HPLC-HRMS(/MS) and DI-HRMS(/MS$^n$) LC fractionation experiments****. Conceptual schematic of the data processing, metabolite annotation, data management and additional data analysis techniques and approaches used.*

**Formatted:** Font: 9 pt, Font color: Auto

**Formatted:** Centered, Space Before: 0 pt, After: 0 pt

**Formatted:** Font: 9 pt, Do not check spelling or grammar, All caps

# 1.13 DMA Galaxy workflow

## 1.13.1 Overview

The “DMA Galaxy Workflow” is split into 5 components: “Data input”, “(U)HPLC-HRMS(/MS) data processing”, “DI-HRMS(MS$^{n}$) data processing”, “Metabolite annotation” and the “Combining” component.

The “Data input” workflow component consists of either the RAW mass spectrometry files, the equivalent mzML file or the sample metadata for each file.

The **“(U)HPLC-HRMS(/MS) data processing”** workflow component uses the Workflow4Metabolomics XCMS Galaxy tools (Giacomoni et al., 2015; Smith et al., 2006) for chromatographic feature picking and grouping of (U)HPLC-HRMS features between multiple files. Galaxy wrappers of the msPurity R package are also used to flag and calculate quality metrics for MS1 features (i.e. flag blank features, calculate RSD of the intensities and retention time of the MS1 data), calculate precursor ion purity of the fragmentation spectra, align fragmentation spectra to the XCMS MS1 features, filter fragmentation data based on signal to noise, average fragmentation spectra within files (intra-averaging), average fragmentation spectra across files (inter-averaging), combine the (U)HPLC-HRMS(/MS) based analysis into a single SQLite database and create MSP files of the averaged fragmentation spectra. Additionally the CAMERA Galaxy tool (Giacomoni et al., 2015; Kuhl et al., 2012) is used as means to determine adducts and isotopes.

The **“DI-HRMS(/MS$^{n}$) data processing”** workflow component uses the Galaxy wrappers for the DIMSpy tools (github.com/computational-metabolomics/dimspy-galaxy) to process the DI-HRMS data - including averaging and grouping spectra within and across files and blank subtraction. Galaxy wrappers of the MSnPy tools are also used to process the fragmentation component of the DI-HRMS(/MS$^{n}$) data – including grouping related scans, processing and averaging the m/z and intensities from each scan group, creating spectral and fragmentation trees of each group and creating MSP files of the averaged fragmentation spectra. Additionally, the msPurity Galaxy tools are used to calculate the precursor ion purity and CAMERA DIMS Galaxy tool is used as means to determine adducts and isotopes.

The **“Metabolite annotation”** workflow components use the Galaxy wrappers of msPurity for spectral matching; the Galaxy wrapper for SIRIUS-CSI:FingerID (Dührkop et al., 2019) for predicting structure based on fragmentation trees and a machine learning model derived from public mass spectrometry datasets; and the Galaxy wrapper for MetFrag (Ruttkies et al., 2019, 2016; Wolf et al., 2010) that annotates features using *in silico* prediction of mass spectral fragmentation patterns. Annotation of the MS1 features is also performed using the Galaxy wrapper for BEAMSpy - using a neutral mass lookup of each feature to a compound database (HMDB) based on a neutral mass calculated from a predefined list of adducts.

**Formatted:** Font: 9 pt, Font color: Auto

**Formatted:** Centered, Space Before: 0 pt, After: 0 pt

**Formatted:** Font: 9 pt, Do not check spelling or grammar, All caps

The **“Combining”** workflow component uses the msPurity combineAnnotation tools and the LC Fractionation Galaxy tool to combine all the annotations and processed data into a single SQLite database. The (U)HPLC-HRMS(/MS) data is first combined using the msPurity combineAnnotation tool, where all annotations from the (U)HPLC-HRMS(/MS) dataset (i.e. spectral matching, MetFrag, SIRIUS CSI:FingerID and MS1 neutral mass lookup) are aligned based on their InChiKeys. Scores from each approach are weighted based on predetermined weights and the annotations are ranked for each annotated feature. The “LC Fractionation” Galaxy tool is then used when fractionation data is available and combines all the DI-HRMS(/$MS^n$) data and annotations in the same way as the combineAnnotation but additionally aligns features observed in the DI-HRMS(/$MS^n$) fractions to the (U)HPLC-HRMS features.

Galaxy workflows and workflow histories available at https://dma.galaxy.bham.ac.uk/. Full parameters for each tool used can be found in the histories and key parameters are also detailed in the individual sections of the methods here.

**Formatted:** Font: 9 pt, Font color: Auto

**Formatted:** Centered, Space Before: 0 pt, After: 0 pt

**Formatted:** Font: 9 pt, Do not check spelling or grammar, All caps

## 1.13.2 Galaxy tool summary

**Table S9:** Summary of Galaxy tools

| Tool Name | Underlying software of tool | Fig 3. DMA Galaxy Work-flow step(s) | Description of tool |
|---|---|---|---|
| **LC-MS and LC-MS/MS data processing** | | | |
| MSnBase.readMSData | MSnBase R Package | 2 | Read in mzML files using the MSnBase R package to be used for XCMS processing |
| xcms.findChromPeaks | XCMS R package | 3 | Data processing tool for feature detection of mass spectrometry datasets |
| xcms.findChromPeaks Merger | XCMS R package | 4 | Data processing tool to merge multiple xcms.xcmsSet data objects into a single object |
| xcms.groupChromPeaks | XCMS R package | 5 | Data processing tool for grouping mass spectrometry peaks between different files |
| CAMERA .Annotate | CAMERA R package | 7 | Annotation tool for adducts and isotopes of LC-MS data |
| **msPurity.purityA | msPurity R package | 1 | Assess the precursor ion purity for fragmentation spectra acquired from either a LC-MS/MS or DI-MS/MS ($MS^n$) experiment |
| **msPurity.flagRemove | msPurity R package | 6 | Tool to flag XCMS grouped peaks from the xcmsSet object based on various thresholds (e.g. RSD of intensity and retention time). The peaks can then be removed from the xcmsSet object and regrouped |
| **msPurity.frag4feature | msPurity R package | 8 | Assign fragmentation spectra (MS/MS) stored within a purityA class object to grouped features within an XCMS xset object. |
| **msPurity .filterFragSpectra | msPurity R package | 9 | Flag and filter features based on signal-to-noise ratio, relative abundance, intensity threshold and precursor ion purity of precursor. |
| **msPurity .averageFragSpectra | msPurity R package | 10, 11 | Average and filter fragmentation spectra for each XCMS feature using an msPurity purityA object. The tool uses the msPurity functions averageAllFragSpectra, averageIntraFragSpectra and averageInterFragSpectra. |
| **msPurity. createMSP | msPurity R package | 12 | This tool will extract the MS/MS spectra data from an msPurity object into a MSP file |
| **msPurity. createDatabase | msPurity R package | 14 | Create SQLite database of LC-MS/MS dataset |
| **DI-HRMS(/$MS^n$) data processing** | | | |
| dimspy .Process scans | dimspy python Package | 22,23 | Process Scans (and SIM-Stitch) - Read, filter and average MS scans |
| dimspy .merge peaklists | dimspy python package | 24 | Merge peaklists produced by the tools 'Process scans (and SIM-Stitch)' or 'Replicate filter' |
| dimspy .align samples | dimspy python package | 25 | Align Samples - Align peaks across Peaklists |
| dimspy. blank filter | dimspy python package | 26 | Blank Filter - Remove 'blank' peaks from the biological mass spectra |
| dimspy. Get peaklist | dimspy python package | 27 | Export a tsv file of the peaklist |

**Formatted:** Font: 9 pt, Font color: Auto

**Formatted:** Centered, Space Before: 0 pt, After: 0 pt

**Formatted:** Font: 9 pt, Do not check spelling or grammar, All caps

| Tool Name | Underlying software of tool | Fig 3. DMA Galaxy Work-flow step(s) | Description of tool |
|---|---|---|---|
| *CAMERA DIMS | CAMERA R package (modified to handle DIMS data) | 29 | Modification of the R package CAMERA to work on DI-MS data |
| **msPurity .dimsPredictPurity(single) | msPurity R package | 28 | Calculate the anticipated precursor ion purity from a DI-MS dataset |
| **MSnPy Group Scans | MSnPy python package | 30 | Group fragmentation events from DI-MS(MS$^n$) data |
| **MSnPy Process Scans | MSnPy python package | 31 | Read, filter and average DI-MS(MS$^n$) scans |
| **MSnPy Create Spectral Trees | MSnPy python package | 32 | Create spectral trees from processed scan data derived from DI-MS(MS$^n$) data |
| **MSnPy Convert Spectral Trees | MSnPy python package | 33 | Convert spectral trees to MSP files or dimspy PeakList objects |
| **Metabolite annotation** | | | |
| BEAMSpy | BEAMSpy python package | 20, 40 | Birmingham mEtabolite Annotation for Mass SpectroMetry - for MS1 metabolite annotations and spectral annotations (e.g. adducts and isotopes) |
| **msPurity .spectralMatching | msPurity R package | 15, 39 | Perform spectral matching to spectral libraries for an LC-MS/MS dataset. |
| *SIRIUS CSI:FingerID | SIRIUS CSI:FingerID | 16, 37 | Run the metabolite annotation software SIRIUS CSI:FingerID on MSP files |
| *MetFrag | MetFrag | 18, 38 | Run the metabolite annotation software MetFrag on MSP files |
| **MSnPy Annotate Spectral Trees | MSnPy python package | 35 | Annotate and/or filter spectral trees derived from DI-MS(MS$^n$) data |
| **MSnPy Rank Spectral Trees | MSnPy python package | 36 | Rank annotated spectral trees derived from DI-MS(MS$^n$) data |
| **Combining** | | | |
| **msPurity.combineAnnotations | msPurity R package | 21 | Combine, score and rank metabolite annotation results |
| **LC fractionation processor | lcfrac python package | 41 | Combine and process spectra and metabolite annotation results from an LC-MS(/MS) & DI-MS(MS$^n$) fractionation experiment |
| **Miscellaneous** | | | |
| **msp_split | Python script | 13 | Split an MSP file into *n* number of files |
| **msp2db | msp2db python package | 34 | create an SQLite database from MSP files |
| **msPurity.purityX | msPurity R package | ***NA | Calculate the anticipated precursor ion purity from a LC-MS XCMS dataset |

**Formatted:** Font: 9 pt, Font color: Auto

**Formatted:** Centered, Space Before: 0 pt, After: 0 pt

**Formatted:** Font: 9 pt, Do not check spelling or grammar, All caps

| Tool Name | Underlying software of tool | Fig 3. DMA Galaxy Work-flow step(s) | Description of tool |
|---|---|---|---|
| **deconrank | Deconrank python package | ***NA | Deconvolute adducts and isotopes then score and rank for fragmentation |

**Footnotes**: *New Galaxy tool developed by authors. **New underlying software and Galaxy tool developed by authors. ***Not used directly as part of the metabolite annotation workflow shown in **Figure 3** of the main paper. The abbreviations of liquid chromatography mass spectrometry (LC-MS), liquid chromatography tandem mass spectrometry (LC-MS/MS), direction infusion mass spectrometry (DI-MS) and direct infusion mass spectrometry with multi stage fragmentation (DI-MS($MS^n$)) are used here when describing approaches that do not require ultra-high performance liquid chromatography (UHPLC) or high resolution mass spectrometry (HRMS).

Formatted: Font: 9 pt, Font color: Auto

Formatted: Centered, Space Before: 0 pt, After: 0 pt

Formatted: Font: 9 pt, Do not check spelling or grammar, All caps

### 1.13.3 (U)HPLC-HRMS(/MS) data processing

#### 1.13.3.1 MSnBase and XCMS

(U)HPLC-HRMS(/MS) mzML files were first processed with MSnBase.readMSData Galaxy tool that creates MSnBase R datatypes that are compatible with the subsequent XCMS tools.

The XCMS  Galaxy tool xcms.findChromPeaks is then used to perform (U)HPLC-HRMS feature detection, the CentWave algorithm was used and the following parameters were used - ppm: 11.5, peakwidth: “3, 30”, mzdiff: -0.00375 and snrthres: 10.

The Galaxy tool xcms.findChromPeaks is ran as a data collection where each MSnBase.readMSData processed mzML file is ran independently to one another. The resulting xcms.findChromPeaks outputs are then merged into a single object and appropriate sample metadata applied using the xcms.findChromPeaks merger Galaxy tool.

The Galaxy tool xcms.groupChromPeaks was then used to group the chromatographic features across each of the files using the PeakDensity approach was used with the following parameters - bw: 0.25, minFraction: 0.5, minSamples: 1 and binSize: 0.0157.

The parameters for xcms.findChromPeaks and xcms.groupChromPeaks determined by running IPO (Libiseller et al., 2015) optimisation of the XCMS parameters on a subset the (U)HPLC-HRMS data files for the majority of the individual assays and taking the medium parameters for each value.

#### 1.13.3.2 msPurity ((U)HPLC-HRMS(/MS))

(U)HPLC-HRMS(/MS) data processing was done primarily through the Galaxy tools of msPurity that has been substantially updated since the original publication.

Within the DMA galaxy workflow, using the Galaxy tool msPurity.purityA the precursor ion purity was calculated for all the fragmentation spectra from the (U)HPLC-HRMS(/MS) mzML files (linear interpolation to derive the precursor ion purity score with, features less than 5% of the peak were removed and C13 isotope peaks were disregarded).

The Galaxy tool msPurity.frag4feature was then used to map the fragmentation spectra to the XCMS chromatographic features using a tolerance of 10 ppm to match between the fragmentation spectra precursor and XCMS features.

The Galaxy tool msPurity.filterFragSpectra was then used to calculate signal-to-noise ratio of all fragmentation spectra. It should be noted though that no features were removed at this stage as further filtering was performed based on averaged spectra in the next step in the workflow.

The Galaxy tool msPurity.averageFragSpectra was then used to average the fragmentation spectra associated with XCMS chromatographic features. The tool was run twice, first to average within each file (intra-averaging) and then again to average across files (inter-averaging). The averaging was performed on the *m/z* values using hierarchical clustering with a cutoff of 5 ppm where the averaged fragment peak consists of the median intensity and median

**Formatted:** Font: 9 pt, Font color: Auto

**Formatted:** Centered, Space Before:  0 pt, After:  0 pt

**Formatted:** Font: 9 pt, Do not check spelling or grammar, All caps

*m/z* value of the contributing peaks that are averaged. Fragment peaks were removed that were not present in at least 50% of the scans (for intra-averaging) or at least 50% of files (for inter averaging). See **Figure S7** for schematic of data processing and averaging strategy.

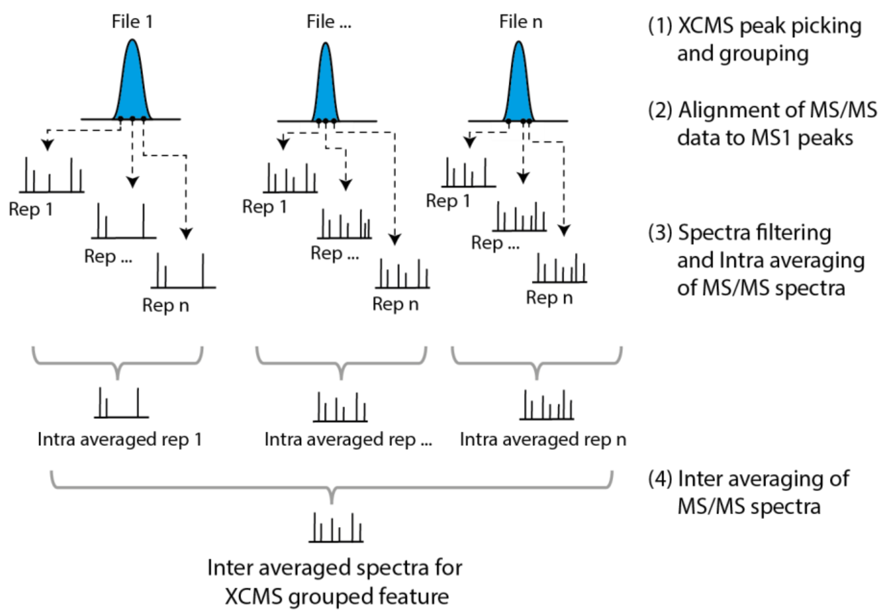


***Figure S7: (U)HPLC-HRMS(/MS) data processing schematic for msPurity and XCMS.*** *Schematic showing how (U)HPLC-HRMS(/MS) data within a file is averaged (intra-averaging) and across a file is averaged (inter-averaging) using msPurity and XCMS.*

The Galaxy tool msPurity.createMSP was then used to create MSP files for the averaged spectra that in turn is used with the MetFrag and SIRIUS CSI:FingerID Galaxy tools, The Galaxy tool msPurity.createDatabase tool was used to create an SQLite database of the (U)HPLC-HRMS(/MS) dataset (schema - https://bioconductor.org/packages/release/bioc/vignettes/msPurity/inst/doc/msPurity-spectral-database-vignette.html) - the SQLite database is used as input for the (U)HPLC-HRMS(/MS) spectral matching and used to map the DI-HRMS(/MS$^n$) data to the (U)HPLC-HRMS(/MS) data.

Additionally, the Galaxy tool 'msPurity.flagRemove' was used to calculate the RSD of the intensity and RSD of the retention time for each feature the blank and non-blank (i.e. "samples") files, flags any features that are not observed in less than a defined minimum fraction of features and flag features that are determined as blanks. We not here that no features were removed at this stage as we intended to obtain all annotations regardless of the feature quality.

**Formatted:** Font: 9 pt, Font color: Auto

**Formatted:** Centered, Space Before: 0 pt, After: 0 pt

**Formatted:** Font: 9 pt, Do not check spelling or grammar, All caps

Instead, blank filtering was performed after the Galaxy workflow at the final combining stage (see section S1.14)

Both the Galaxy tools of msPurity and the underlying R package are developed and maintained by the authors.

#### 1.13.3.3 CAMERA

The Galaxy tool CAMERA.Annotate was used to annotate isotopes and adducts in the LC-MS/MS data using a threshold of 5 ppm and absolute *m/z* error of 0.015, and using the correlation inside samples for peak grouping.

Formatted: Font: 9 pt, Font color: Auto

Formatted: Centered, Space Before: 0 pt, After: 0 pt

Formatted: Font: 9 pt, Do not check spelling or grammar, All caps

### 1.13.4 DI-HRMS(/MS$^n$) data processing

#### 1.13.4.1 DIMSpy

DI-HRMS processing was done via the Galaxy tools of the DIMSpy Python package. For each assay, the workflow processed each of the DI-HRMS raw files generated from every well of the fractionation experiment.

The DIMSpy.Process Scan tool was used to process the scans and perform hierarchical clustering to average the spectra across scans (i.e. the same technique that was used for averaging the fragmentation spectra). The DIMSpy.Process tool was setup to either process SIMstitch DI-HRMS data or full scan DI-HRMS(/MS$^n$) data. Each processing and averaging is performed on each raw file independently and then merged into a single object using the DIMSpy.Merge peaklist tool. A signal to noise threshold of 3 (based on the ThermoFisher noise value extracted from the .RAW file).

We note that the *Daphnia* samples and blank samples were processed using slightly different settings, with the *Daphnia* samples used a 5 ppm tolerance to average across scans and the blank samples used a 2 ppm tolerance. The lower ppm used for blank samples was used as an attempt to provide more robust blank subtraction, the difference however is thought to have minimal overall impact and in any future use of the workflow using the same ppm tolerance between *Daphnia* samples and blank samples would be suggested.

The DIMSpy.Align Samples Galaxy tool is then used to align the blank and *Daphnia* samples together based on a ppm tolerance of 5 ppm, and then blank subtraction of peaks can be performed using the DIMSpy.Blank filter Galaxy tool, again based on a ppm tolerance of 5 and a *Daphnia* feature required to be 10 times the intensity of the blank sample feature.

The Galaxy tool DIMSpy.Get peaklists was used to create a data collection of the processed peaklist as text files and a data collection of the peaklists as hdf5.

Both the Galaxy tools of DIMSpy and the underlying Python package are developed and maintained by the authors.

#### 1.13.4.2 MSnPy

DI-HRMS(/MS$^n$) processing was conducted via several Galaxy tools that cover the functionalities of the MSnPy python package. For each assay, the workflow processed each of the DI-HRMS(/MS$^n$) raw files generated from every well of the analytical fractionation experiment.

The first stage is to group scans that are replicates of one another and map the relationship between scans (e.g. replicates of different collision energies, types and MS levels) using the MSnPy.Group scans Galaxy tool. Next the Galaxy tool MSnPy.Process-scans is used to average the features within each scan group (using the same hierarchical clustering approach as used in DIMSpy and msPurity for averaging) with a ppm tolerance of 5. The averaged scans are then used to create spectral tree networks using the Galaxy tool MSnPy.create-spectral-trees. The averaged fragmentation data of these trees are then exported using the

**Formatted:** Font: 9 pt, Font color: Auto

**Formatted:** Centered, Space Before: 0 pt, After: 0 pt

**Formatted:** Font: 9 pt, Do not check spelling or grammar, All caps

"MSnPy.convert-spectral-trees" tool. This provides outputs of the averaged spectra in multiple configurations: merged fragmentation spectra across all collision energies and MS levels; merged fragmentation spectra but keeping each energy collision energy independent and MS level independent; and finally just the precursors that were targeted for the fragmentation. The outputs are available in hdf5 and MSP formats. See **Figure S8** for a schematic of the data processing and averaging strategy used with MSnPy.

Both the Galaxy tools of MSnPy and the underlying Python packages for DI-HRMS(/$MS^n$) data processing are developed and maintained by the authors.

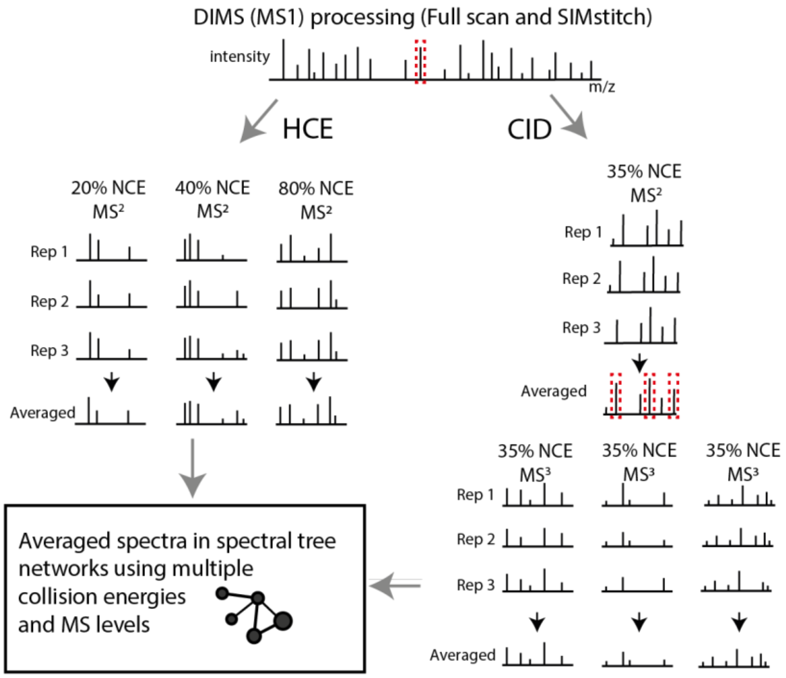


***Figure S8: DI-HRMS(/$MS^n$) data processing schematic for MSnPy.*** *Schematic of how DI-HRMS($MS^n$) data is averaged. HCE and CID data was collected within the same run and is initially processed and averaged separately and then merged at different levels for the creation of relevant spectra for annotation purposes. HCE = Higher energy collisional dissociation. CID = Collision induced dissociation. NCE = normalised collision energy.*

**Formatted:** Font: 9 pt, Font color: Auto

**Formatted:** Centered, Space Before: 0 pt, After: 0 pt

**Formatted:** Font: 9 pt, Do not check spelling or grammar, All caps

#### 1.13.4.3 msPurity (DI-HRMS(/$MS^n$))

The precursor ion purity was calculated for all the $MS^1$ features using the msPurity.dimsPredictPurity Galaxy tool for all features observed from the DIMSpy peaklist generated for every well of the fractionation experiment for each assay.

It should be noted that the precursor ion purity scores used for the DI-HRMS data using the dimsPredictiPurity tool were recalculated for some assays to assess different parameters used from those in the originally submitted workflow. For clarity a separate Galaxy history has been created with the suffix (DI-HRMS predicted precursor ion purity) for each DI-HRMS based assay and within each of these histories are the final DI-HRMS precursor ion purity scores used for filtering the metabolite annotations.

Both the Galaxy tool msPurity.dimsPredictPurity and underlying R package are developed and maintained by the authors.

#### 1.13.4.4 msp2db

To perform spectral matching of the DI-HRMS(/$MS^n$) data we first need to generate an SQLite database of the query spectra to be used as input for msPurity.spectralMatching. This is done via the msp2db Galaxy tool for all the $MS^n$Py-generated MSP files of the DI-HRMS(/$MS^n$) data, generated for every well of the fractionation experiment for each assay.

Both the Galaxy tool msp2db and underlying python package are developed and maintained by the authors.

#### 1.13.4.5 CAMERA-DIMS

Adduct and isotopes were checked using a modified version CAMERA to work on DI-HRMS data using the Galaxy tool CAMERA-DIMS for all the DIMSpy peaklist generated for every well of the fractionation experiment for each assay.

The Galaxy tool CAMERA-DIMS was developed by, and maintained by, the authors.

Formatted: Font: 9 pt, Font color: Auto

Formatted: Centered, Space Before: 0 pt, After: 0 pt

Formatted: Font: 9 pt, Do not check spelling or grammar, All caps

## 1.13.5 Metabolite annotation

### 1.13.5.1 msPurity spectralMatching

The spectral matching for both (U)HPLC-HRMS(/MS) and the DI-HRMS(/MS$^n$) data was performed using Galaxy tool msPurity.spectralMatching.

The library SQLite database used consist of 667,163 fragmentation spectra with 229,936 unique compounds (based on a unique InChiKey) was generated using the package msp2db - using all of the MoNA mass spectrometry database as of 23rd April 2021. The database also included an internal library of mass spectral fragmentation data collected in-house at Phenome Centre Birmingham.

The library spectra was filtered by ionisation type and only included the following instruments types: APCI-ITFT, APCI-ITTOF, CE-ESI-TOF, ESI-ITFT, ESI-ITTOF, ESI-QFT, ESI-QTOF, ESI-TOF, in source CID, in-silico QTOF, Ion trap, LC-APCI-ITFT, LC-APCI-QTOF, LC-APCI-Q, LC-ESI-IT, LC-ESI-ITFT, LC-ESI-ITTOF, LC-ESI-Q, LC-ESI-QFT, LC-ESI-QIT, LC-ESI-QQ, LC-ESI-QTOF, LC-ESI-TOF, LC-Q-TOF/MS, LC-QTOF, Linear Ion Trap, LIT, MALDI-QIT, MALDI-TOF, MALDI-TOFTOF, Orbitrap, QIT, QIT-FT, QIT-TOF, QqQ, Q-TOF, Quattro_QQQ, QTOF, Flow-injection QqQ/MS, LC-APPI-QQ, LC-ESI-QQQ and MALDI-QITTOF. Some of the fragmentation spectra did not have an instrument type defined; in those cases the instrument names were checked and spectra with relevant instruments were included.

After filtering for instrument types, the database consists of 648,243 fragmentation spectra with 221,575 unique compounds (based on a unique InChiKey).

Weighted vectors of the intensity and m/z were used for the spectral matching comparison (see equation 1) - for all analysis in this paper the weighting was as per the MassBank (Horai et al. 2010) approach, i.e. x = 0.5 and y = 2. The dot product cosine (equation 2 – where $Q$ represents the query spectra and $L$ represents the library spectra), was then calculated. Other similarity metrices were calculated (i.e. reverse dot product cosine and composite dot-product cosine) but not used as part of the final filtering and ranking of annotations.

$$w = [peak\_intensity]^x \times [mz]^y \qquad (1)$$

$$dpc = \frac{\sum w_Q \times w_L}{\sqrt{\sum w_Q^2 \times \sum w_L^2}} \qquad (2)$$

For the LC-MS/MS spectral matching the msPurity.spectralMatching used the output of the msPurity.createDatabase tool and spectral matching was performed on all inter-averaged fragmentation spectra.

For the the DI-HRMS(/MS$^n$) spectral matching the msPurity.spectralMatching used the output from msp2db that created an SQLite database of the averaged fragmentation spectra for each collision energy and was ran for every well of the fractionation experiment for each assay.

The precursor of the library and the query spectra need to be within +/- 5 ppm of one another.

Both the Galaxy tools of msPurity and the underlying R package described above are developed and maintained by the authors.

**Formatted:** Font: 9 pt, Font color: Auto

**Formatted:** Centered, Space Before: 0 pt, After: 0 pt

**Formatted:** Font: 9 pt, Do not check spelling or grammar, All caps

Mass spectral libraries used are available via github (github.com/computational-metabolomics/msp2db/releases/tag/v0.0.14-mona-23042021).

**Table S10**: Summary of fragmentation spectra used for spectral matching with msPurity

| MSP files origin | Original source | Spectra count | | | Unique Compound count | | |
|---|---|---|---|---|---|---|---|
| | | **Pos** | **Neg** | **All** | **Pos** | **Neg** | **all** |
| MoNA | embl-mcf | 692 | 601 | 1293 | 256 | 321 | 431 |
| | fahfa | NA | 4290 | 4290 | NA | 4290 | 4290 |
| | fiehn_hilic | 1712 | 1348 | 3060 | 980 | 813 | 1219 |
| | fiehn_plasma | 4439 | 4216 | 8655 | 377 | 295 | 594 |
| | gnps | 19554 | 4247 | 23801 | 10356 | 2747 | 10983 |
| | hmdb | 2088 | 1074 | 3162 | 672 | 271 | 923 |
| | lipidblast | 143342 | 342454 | 485796 | 110833 | 154770 | 197527 |
| | massbank | 40040 | 19062 | 59102 | 5350 | 3934 | 7226 |
| | metabobase | NA | 1254 | 1254 | NA | 290 | 290 |
| | pathogen_box | 392 | NA | 392 | 392 | NA | 392 |
| | respect | 3879 | 2495 | 6374 | 1149 | 907 | 1511 |
| | riken_ims_oxidized_phospholipids | NA | 386 | 386 | NA | 386 | 386 |
| | vaniya_fiehn_natural_products_library | 31944 | 12992 | 44936 | 2677 | 2464 | 2768 |
| Internal library | METASCI_HILIC | 682 | 676 | 1358 | 460 | 538 | 695 |
| | METASCI_RP | 923 | 733 | 1656 | 514 | 442 | 634 |
| | PCB_HILIC | 523 | 196 | 719 | 238 | 123 | 263 |
| | PCB_LIPIDS | 326 | 257 | 583 | 154 | 159 | 214 |
| | PCB_LIPIDS_IPA | 321 | 251 | 572 | 145 | 157 | 213 |
| | PCB_RP | 454 | 400 | 854 | 230 | 187 | 289 |
| **Total** | | 251311 | 396932 | 648243 | 127953 | 168207 | 221575 |
| **Total (without LipidBlast in silico)** | | 107969 | 54478 | 162447 | 17150 | 13951 | 24589 |

**Formatted:** Font: 9 pt, Font color: Auto

**Formatted:** Centered, Space Before: 0 pt, After: 0 pt

**Formatted:** Font: 9 pt, Do not check spelling or grammar, All caps

#### 1.13.5.2 MetFrag

Metabolite annotation using MetFrag was performed using the Galaxy tool of the same name. MetFrag is a combinatorial *in silico* fragmentation tool, that generates *in silico* spectra from potential matches of a hypothetical neutral masses or molecular formula of the fragmentation precursor ion.

When annotating the (U)HPLC-HRMS(/MS) data within the DMA workflow, the MetFrag Galaxy tool takes as input the MSP of the inter-averaged fragmentation spectra generated from msPurity.createMSP. When annotating DI-HRMS(/$MS^n$) data from the DMA workflow, the MetFrag Galaxy tool takes as input the MSP of the combined and averaged spectra from multiple collision energies and MS levels, generated from MSnPy.convert-spectral-trees tools. This was run for every well of the fractionation experiment for each assay.

The analysis in this paper used a PostgreSQL database of all the compounds within PubChem to search against to generate the *in-silico* spectra. An overall weighted score was used of the following: Fragmentor score (which scores the fragmentation based on the intensities, m/z values and bond energies of the matches between the predicted spectra and the query spectra); the OfflineMetFusionScore (based on a predefined spectral library within MetFrag); and the Suspect list score (which ranks annotations against a list of suspected compounds – in our cases a list of natural products). The weights used for the DMA workflow were: FragmentScore: 0.2, OfflineMetFusionScore: 0.3 and SuspectListScore: 0.5. The scores were chosen based on the default values of the Galaxy tool based on the community development of the tool (FragmentScore: 0.4, OfflineMetFusionScore: 0.6 and SuspectListScore: 1) but scaled to equal 1 to easier integrate with other scores. Whilst the combining approach used here is convenient, further MetFrag analysis beyond this paper would benefit of using the scores independent as the overall score is somewhat biassed to both the suspect list and offline metfusion score and led to a rather harsh cutoff being used for final filtering of MetFrag (>0.95) to ensure we were always using a reliable “FragmentScore”.

We also note that use of the PubChem structural database for MetFrag means that the annotations are not restricted to existing metabolome knowledgebases – however this can lead to annotations where there is no prior evidence of either the compound occurring naturally, or of it having been detected using a mass spectrometer. As such, the use of MetFrag’s “SuspectedListScore” and the “OfflineMetFusionScore” means that compounds that have either previously been found as a natural product or that have previously been reported in MassBank, are prioritised, as these were considered more likely to be an observable metabolite in *D. magna*. In addition, the final filtering of MetFrag (>0.95) essentially forces any final reported annotation derived solely from MeFrag to have been on the “SuspectedListScore” and have a high scoring “OfflineMetFusionScore”.

The mass deviation for the database search was set at 5 ppm and the fragment peak match deviation with an absolute mass deviation of 0.001 Da.

**Formatted:** Font: 9 pt, Font color: Auto

**Formatted:** Centered, Space Before: 0 pt, After: 0 pt

**Formatted:** Font: 9 pt, Do not check spelling or grammar, All caps

The following adducts were considered for positive ionisation mode: $[M+H]^+$,$[M+Na]^+$,$[M+NH_4]^+$ and $[M+K]^+$. The following adducts were considered for negative ionisation mode: [M-H]-,[M+Cl]-,$[M+HCOO]^-$,$[M+CH_3COO]^-$ and $[M-H+CH_3COOH]^-$.

The MetFrag Galaxy tool is developed and maintained by the authors (with help within the Galaxy community).

#### 1.13.5.3 SIRIUS CSI:FingerID

SIRIUS is a mass spectrometry analysis and annotation GUI and CLI software that can be used to perform isotope analysis, fragmentation trees analysis and predict metabolite structures using CSI:FingerID. The Galaxy tool SIRIUS CSI:FingerID, provides a wrapper of the SIRIUS CLI specifically to perform the fragmentation tree creation, CSI:FingerID metabolite annotation and the CANOPUS metabolite class annotation

When annotating the (U)HPLC-HRMS(/MS) data within the DMA workflow, the SIRIUS CSI:FingerID Galaxy tool takes as input the MSP of the inter-averaged fragmentation spectra generated from msPurity.createMSP. When annotating the DI-HRMS(/$MS^n$) data from the DMA workflow, the SIRIUS CSI:FingerID tool takes in as input the combined and averaged spectra at each collision energy, converted into MSP format from the from the MSnPy Galaxy tool MSnPy.convert-spectra-trees tool and was run for every well of the fractionation experiment, for each assay.

The annotation workflow used included the “all biological” compound database for the CSI:FingerID database, specifying for Orbitrap based analysis and with 5 ppm mass deviation used. For each fragmentation spectra SIRIUS CSI:FingerID generates a rank list of potential structures - annotated to a partial InChiKey.

The following adducts were considered for positive ionisation mode: $[M+H]^+$,$[M+Na]^+$,$[M+NH_4]^+$ and $[M+K]^+$. The following adducts were considered for negative ionisation mode: [M-H]-,[M+Cl]-,$[M+HCOO]^-$,$[M+CH_3COO]^-$ and $[M-H+CH_3COOH]^-$.

The SIRIUS CSI:FingerID Galaxy tool was primarily developed by the authors (with help within the Galaxy community) and is maintained by the authors.

#### 1.13.5.4 BEAMSpy

The BEAMSpy Galaxy tool was used to annotate metabolites detected during DI-HRMS(/$MS^n$) and (U)HPLC-HRMS analyses. The BEAMSpy tool uses a calculated neutral mass of each MS1 feature and searches this against a Metabolite library of compounds. As there is no *D. magna* list of metabolites to search against, HMDB was used instead as it provides one of the larger resources of known metabolites. Note that no annotation was reported in this paper that was solely dependent on BEAMSpy metabolite annotation of MS1-only data.

**Formatted:** Font: 9 pt, Font color: Auto

**Formatted:** Centered, Space Before: 0 pt, After: 0 pt

**Formatted:** Font: 9 pt, Do not check spelling or grammar, All caps

When annotating the (U)HPLC-HRMS data with BEAMSpy within the DMA workflow, the BEAMSpy Galaxy tool takes as input a text (.tsv) file of the XCMS grouped chromatographic features (from the xcms.groupChemPeaks Galaxy tool). When annotating the DI-HRMS(/MS$^{n}$) data from the DMA workflow, BEAMSpy Galaxy tool takes as input a text (.tsv) file containing DI-HRMS features generated by the DIMSpy.Get peaklist Galaxy tool. This was run for every well of the fractionation experiment for each assay.

#### 1.13.5.5 MSnPy molecular formula annotation

*De novo* metabolite annotation to a molecular formula (i.e. without a predefined compound database) of the DI-HRMS(/MS$^{n}$) data was done via the MSnPy.annotate-trees Galaxy tool. Due to the multiple collision energies and MS levels collected, the DI-HRMS(/MS$^{n}$) data provided an opportunity to calculate more accurate *de novo* molecular formula annotations of mass spectrometry features than would usually be possible with (U)HPLC-HRMS(/MS) data. The MSnPy.annotate-trees used a ppm tolerance of 5, applied a heuristic rules method to calculate the molecular formula, and used the pre-calculated molecular formula database – mfdb (https://mfdb.bham.ac.uk) – consisting of precomputed molecular formulas based on the assumption that the compound would comprise the following chemical elements (CHNOPS) within the measured mass range of the mass spectrometer. The “MSnPy.rank-spectral-trees”Galaxy tool is then used to rank the annotated trees based on the number of neutral losses explained by the molecular formula annotation of the annotated tree. The MSnPy molecular formula annotation and ranking was ran on every well of the fractionation experiment for each assay.

Additionally, it should be noted that, when the annotations described here were summarised in the main paper, only molecular formulae were included in instances where there were ten or fewer top-ranked candidates.

The MSnPy molecular formula annotation Galaxy tool and underlying python package was developed by and maintained by the authors.

### 1.13.6 Combining

#### 1.13.6.1 msPurity combineAnnotation

All (U)HPLC-HRMS(/MS) derived annotations and processed data are combined into a single SQLite database using the msPurity.combineAnnotation Galaxy tool, where the database generated at an earlier stage (msPurity.createDatabase) and updated via the spectral matching analysis (msPurity.spectralMatching) is combined with the annotation results from MetFrag, SIRIUS CSI:FingerID and BEAMSpy of the (U)HPLC-HRMS(/MS) data.

In addition to the annotation approaches above, we also calculated a “biological similarity metric”. Here, a filtered list of HMDB compounds was generated in which entries determined to be exogenous metabolites were removed. The chemical structures of these retained HMDB metabolites was then compared against all structures available in PubChem, to generate

**Formatted:** Font: 9 pt, Font color: Auto

**Formatted:** Centered, Space Before: 0 pt, After: 0 pt

**Formatted:** Font: 9 pt, Do not check spelling or grammar, All caps

tanimoto scores. For Pubchem structures found to be dissimilar to the filtered list of HMDB metabolites based on the calculated Tanimoto scores, i.e. not similar to any ‘endogenous’ compound reported in the largest metabolome database, these were less likely to be accurate annotations for HRMS/MSn spectra – or should at least be deprioritised as candidate annotations.

Compounds are aligned across all approaches based on matching InChIKeys (Heller and McNaught, 2009) and the annotations for specific (U)HPLC-HRMS features are ranked based on a combined score of all the annotation approaches where each approach is given a weight. The following weightings were used for the Galaxy Annotation Workflow: spectral-matching: 0.45, metfrag: 0.15, SIRIUS-CSI:fingerID: 0.25 and MS1 lookup: 0.1 and biosimilarity score 0.05.

Weightings were selected by evaluating annotation performance on the MTBLS749 MetaboLights study using a combination of empirical testing and expert judgment. Sensitivity and specificity were calculated following Chao et al. (2020) to guide selection, alongside expert considerations: spectral matching was prioritized as the most reliable source, with MS/MS-based tools like MetFrag and SIRIUS given greater weight than approaches lacking fragmentation data. Weight combinations that didn’t align with these principles were excluded, and the final set demonstrating the best combined sensitivity and specificity was chosen.

Although the approach of selecting the weights here is subjective, being heavily dependent on both the choice of metabolites to calculate the sensitivity and specificity and the “expert” considerations, following the assessment of the (U)HPLC-HRMS(/MS) component of the experimental & computational DMA workflow, wherein we could annotate 89.6% of reference standards when filtering for the 1st top ranked metabolite – we deemed the weightings sufficient to use.

The msPurity.combineAnnotation tool used the following SQLite database of PubChem compounds: https://doi.org/10.5281/zenodo.7756132

The msPurity.combineAnnotation Galaxy tool and underlying R package was developed by and maintained by the authors.

#### 1.13.6.2 LC-Fractionation processor tool

Both the (U)HPLC-HRMS(/MS) and the DI-HRMS(/$MS^n$) derived annotations and processed data are combined into a single SQLite database using the Galaxy tool “LC Fractionation processor”. This takes as input the combined (U)HPLC-HRMS(/MS) annotations from the msPurity.combineAnnotation Galaxy tool as well as for every well of the fractionation experiment for each assay: the DI-HRMS peaklists from DIMSpy, the DI-HRMS(/$MS^n$) peaklists from MSnPy, the DI-HRMS(/$MS^n$) MetFrag annotations, the DI-HRMS(/$MS^n$) SIRIUS CSI:FingerID annotations, the DI-HRMS(/$MS^n$) spectral matching annotations, the DI-HRMS BEAMSpy $MS^1$ annotations and the molecular formula annotations from MSnPy.

**Formatted:** Font: 9 pt, Font color: Auto

**Formatted:** Centered, Space Before: 0 pt, After: 0 pt

**Formatted:** Font: 9 pt, Do not check spelling or grammar, All caps

Compound annotations were aligned for each DI-HRMS feature using the same approach as msPurity.comebinAnnotation and used the same weights: spectral-matching: 0.45, metfrag: 0.15, SIRIUS-CSI:fingerID: 0.25 and MS1 lookup: 0.1 and biosimilarity score 0.05.

The “LC fractionation processor” Galaxy tool used the following SQLite database of PubChem compounds: https://doi.org/10.5281/zenodo.7756132

The “LC fractionation processor” Galaxy tool was developed by, and is maintained by, the authors.

**Formatted:** Font: 9 pt, Font color: Auto

**Formatted:** Centered, Space Before: 0 pt, After: 0 pt

**Formatted:** Font: 9 pt, Do not check spelling or grammar, All caps

### 1.13.7 Galaxy workflow reproducibility

The Workflows can be viewed directly from our Galaxy instance https://dma.galaxy.bham.ac.uk/.

Manual inspection of Galaxy histories was performed and in multiple cases jobs needed to be either re-run due to faults with API calls to web-services, cluster and server related errors, timeout errors, or specific raw files being problematic. In cases where the job could not be repeated successfully, the failed job output was filtered from its dataset collection and the remaining jobs in the history were restarted. All manual interactions are recorded in each of the analysis Galaxy histories.

Spectral matching was repeated with msPurity.spectralMatching in each history to include a wider range of instrument types and library spectra. The subsequent tools msPurity.combineAnnotations and the LC-Fractionation processor tool were repeated to include the updated spectral matching annotations.

The LC-Fractionation tool was repeated several times in each history whilst issues with problematic files and bugs were resolved. The redundant LC fractionation result files have been deleted from the history (but the metadata can still be viewed for traceability). The LC-Fractionation tool is the last step in the workflow so repeating the analysis has no effect on any other tool in the workflow.

**Formatted:** Font: 9 pt, Font color: Auto

**Formatted:** Centered, Space Before: 0 pt, After: 0 pt

**Formatted:** Font: 9 pt, Do not check spelling or grammar, All caps

## 1.14 Combining and summarising all annotations

Five main sources of annotations were combined into a final list of Metabolite annotations: Galaxy workflow annotations, GNPS workflow annotations, mzCloud annotations, NMR annotations and GC-EI-HRMS annotations. All data were combined into a single table encompassing all annotations across every assay.

Entries in this table were filtered to only include annotations that were ranked as the top 1 annotation with either a spectral matching dot product cosine score >0.7, at least 2 shared peaks between library and query spectra, and at least 10% of peaks explained; or be ranked 1st from SIRIUS CSI:FingerID; or have a MetFrag score >0.95.  All Galaxy workflow annotations also had to be derived from fragmentation spectra acquired from a precursor ion with ~~a precursor ion with a~~ "precursor ion purity" >0.5.

Any annotations where the mass difference between the query precursor *m/z* and the library precursor *m/z* for GNPS and mzCloud was above 10 ppm were disregarded. The SIRIUS CSI:FingerID annotations were also filtered to remove any annotations where the calculated neutral mass of the query was not within 10 ppm of the annotation. This is already done within the Galaxy workflow annotations for spectral matching within *msPurity.spectralMatching* and within the MetFrag tool. From all the approaches only the top ranked annotations were used for the final summary of results.

Following the above filtering, annotations derived from mzCloud and the Galaxy workflow could still have more than one annotation per feature (i.e. when there are multiple top 1 ranked annotations). If all the annotations within the top 1 ranked annotations had the same partial InChiKey (i.e., the first block of the InChiKey that encodes the molecular skeleton) and only differed in stereochemistry, one representation of the structure was carried forward. If the annotations that were top 1 ranked had different molecular formulae, the annotation with the lowest ppm error between the theoretical neutral monoisotopic mass and the calculated monoisotopic mass of the feature (based on the adduct used for the annotation) was selected. Following these steps, if the annotation could still not be distilled to a single metabolite annotation (unique InChiKey) the annotation was not included in the final list of metabolites.

The LC-MS annotations for the *Daphnia* assays were further quality-flagged by extracting the XCMS peaklist objects from the Galaxy workflow and applying blank filtering. For each feature, if a corresponding peak was detected in the blank sample, the intensity in the daphnia samples was required to be at least 10-fold higher than that in the blank for the feature to be retained. All LC-MS-based annotations were required to either be directly linked to an XCMS feature that passed the blank-filtering threshold (when using the Galaxy workflow); or if the annotation was generated through the mzCloud or GNPS workflows, such that both the precursor m/z (+/-10 ppm) and the XCMS feature *m/z* (+/-10 ppm) overlapped and fall within the retention time window defined for a valid XCMS feature.

All annotations were chemically classified using ClassyFire (Djoumbou Feunang et al., 2016).

**Formatted:** Font: Italic

**Formatted:** Font: Italic

**Formatted:** Font: 9 pt, Font color: Auto

**Formatted:** Centered, Space Before:  0 pt, After:  0 pt

**Formatted:** Font: 9 pt, Do not check spelling or grammar, All caps

## 1.15 Assessment of the computational and experimental DMA workflow with metabolite reference standards

Metabolite reference standards were analysed to evaluate the effectiveness of the overall DMA workflow, specifically the (U)HPLC-HRMS(/MS) component.

See **Supplemental Table S11** (provided in separate excel file) for a summary of the metabolite reference standards used.

Formatted: Font: 9 pt, Font color: Auto

Formatted: Centered, Space Before: 0 pt, After: 0 pt

Formatted: Font: 9 pt, Do not check spelling or grammar, All caps

# 2 Supplemental - results

Formatted: Font: 9 pt, Font color: Auto

Formatted: Centered, Space Before: 0 pt, After: 0 pt

Formatted: Font: 9 pt, Do not check spelling or grammar, All caps

## 2.1 (U)HPLC-HRMS(/MS) method optimisation

(U)HPLC-HRMS(/MS) method optimisation was undertaken using Syncronis Phenyl and Accucore Amide LC columns. Here, the objective was to maximise both the number and chromatographic distribution of reproducibly detectable metabolic features (RDMFs) accessible during DMA of *Daphnia magna* 'crude' polar extract and associated SPE fractions.

### 2.1.1 Syncronis Phenyl (PHE)

#### 2.1.1.1 Phase one

Phase one of optimising a Syncronis Phenyl-based UHPLC-HRMS(/MS) method for DMA involved exploration of the impact of altering mobile phase organic solvent (methanol or acetonitrile) and additive (0.1% v/v formic acid or 5 mM ammonium acetate) types on RDMF counts and chromatographic distributions.

**Figures S9** and **S10** provide representative examples of the two-dimensional distribution (*m/z* versus retention time) of RDMFs detected using methods 'Ph-CO-[1-4]'. As demonstrated in the marginal boxplots of these figures, the m/z distribution of RDMFs across methods was found to be highly similar, though clear differences existed with respect to the chromatographic distribution of RDMFs. Methods 'Ph-CO-3' and 'Ph-CO-4', both of which used methanol as mobile phase organic solvent, had superior (i.e. wider) distribution of RDMFs across the chromatographic elution window compared to methods 'Ph-CO-1' and 'Ph-CO-2', where acetonitrile was used as organic solvent. Median retention times for RDMFs (and associated interquartile ranges) are summarised in **Table S12**, with significantly greater values recorded for methods 'Ph-CO-3' and Ph-CO-4' compared to 'Ph-CO-1' and 'Ph-CO-2'. The lower eluotropic strength of methanol compared to acetonitrile under reversed phase LC conditions, coupled with methanol's lack of $\pi$-orbital electrons that could disrupt $\pi$-$\pi$ interactions between metabolites and stationary phase moieties, likely contributed to these observations.

The total number of RDMFs recorded using methods 'Ph-CO-[1-4]' is summarised in **Figure S11**. Method 'Ph-CO-3' consistently outperformed all other methods in this regard, irrespective of the sample type or ionisation mode combination considered. Interestingly, all methods yielded more RDMFs when SPE fraction pool DCX12DAX34 was analysed compared to DAX12DCX34, potentially indicating a greater number of anionic versus cationic metabolites in the polar extracts of *Daphnia magna* (though differences in detection sensitivity and selectivity for anionic and cationic metabolites cannot be excluded as contributory factors in this observation).

Overall, method 'Ph-CO-3' outperformed all other methods evaluated during phase one of Syncronis Phenyl-based (U)HPLC-HRMS(/MS) method optimisation, both in terms of the total number and chromatographic distribution of RDMFs. The next most-performant method, method 'Ph-CO-4', offered similar performance metrics to 'Ph-CO-3', however upon closer inspection of raw chromatographic data (not shown) it was found that peak widths were substantially wider for many of the most intense features recorded during negative ionisation mode analyses. These wider peak widths would have proven deleterious to maximising the number of features for

**Formatted:** Font: 9 pt, Font color: Auto

**Formatted:** Centered, Space Before: 0 pt, After: 0 pt

**Formatted:** Font: 9 pt, Do not check spelling or grammar, All caps

which HRMS/MS data could be acquired, and for post-column fractionation during DMA experiments. Method ‘Ph-CO-3’ was therefore taken forward for further optimisation.

**Table S12**: Median and interquartile range of retention times for RDMFs recorded in DMA (U)HPLC-HRMS/MS method optimisation experiments

| | **RDMF retention time metrics (minutes)** | | | | | | | |
|---|---|---|---|---|---|---|---|---|
| **SPE fraction pool** | **DAX12DCX34** | | | | **DCX12DAX34** | | | |
| **Ionisation mode** | **Positive** | | **Negative** | | **Positive** | | **Negative** | |
| **Method name** | **Median** | **IQR** | **Median** | **IQR** | **Median** | **IQR** | **Median** | **IQR** |
| Ph-CO-1 | 6.3 | 5.3 | 5.3 | 5.8 | 6.6 | 6.9 | 5.9 | 6.9 |
| Ph-CO-2 | 5.7 | 6.0 | 5.0 | 5.3 | 7.0 | 5.1 | 6.1 | 4.8 |
| Ph-CO-3 | 8.6 | 9.6 | 7.8 | 9.0 | 9.1 | 9.9 | 8.7 | 8.5 |
| Ph-CO-4 | 7.0 | 11.2 | 6.2 | 8.2 | 10.3 | 9.6 | 9.0 | 8.4 |
| Ph-FO-1 | 7.6 | 12.5 | 5.1 | 9.7 | 6.5 | 11.9 | 6.3 | 9.5 |
| Ph-FO-2 | 1.2 | 11.6 | 0.9 | 5.5 | 6.8 | 15.4 | 5.6 | 11.3 |
| Ph-FO-3 | 3.1 | 11.7 | 1.5 | 8.6 | 6.7 | 11.8 | 6.6 | 10.4 |
| AA-CO-1 | 2.4 | 5.1 | 2.5 | 5.0 | 3.1 | 5.7 | 2.7 | 5.9 |
| AA-CO-2 | 2.4 | 3.6 | 2.3 | 3.9 | 4.3 | 5.7 | 4.4 | 5.8 |
| AA-CO-3 | 2.8 | 4.4 | 2.6 | 4.2 | 4.1 | 5.4 | 4.0 | 5.4 |
| AA-Resus-1 | 3.9 | 7.5 | 6.6 | 7.8 | 4.2 | 8.2 | 7.1 | 8.3 |
| AA-Resus-2 | 3.0 | 6.7 | 5.0 | 7.3 | 2.6 | 6.8 | 3.1 | 6.7 |
| AA-Resus-3 | 8.8 | 10.6 | 9.3 | 10.2 | 11.2 | 11.6 | 11.5 | 5.4 |
| AA-Resus-4 | 7.5 | 8.7 | 9.1 | 8.0 | 8.8 | 9.9 | 10.2 | 4.6 |
| AA-FO-1 | 3.0 | 8.2 | 2.6 | 6.6 | 7.6 | 9.4 | 8.2 | 9.3 |
| AA-FO-2 | 5.5 | 6.8 | 5.5 | 6.6 | 6.9 | 7.3 | 6.3 | 7.0 |
| AA-FO-3 | 4.6 | 9.2 | 7.6 | 9.4 | 7.1 | 9.7 | 7.3 | 9.2 |
| AA-FO-4 | 5.1 | 7.4 | 5.9 | 6.9 | 6.9 | 8.1 | 7.2 | 7.5 |
| AA-FO-5 | 4.8 | 7.8 | 5.3 | 7.9 | 6.4 | 9.6 | 5.8 | 9.6 |
| AA-FO-6 | 6.4 | 9.5 | 6.0 | 9.5 | 7.0 | 9.7 | 7.0 | 9.6 |

Footnotes: IQR - interquartile range; RDMF - reproducibly detectable metabolic feature (see section 1.9 for details)

**Formatted:** Font: 9 pt, Font color: Auto

**Formatted:** Centered, Space Before: 0 pt, After: 0 pt

**Formatted:** Font: 9 pt, Do not check spelling or grammar, All caps

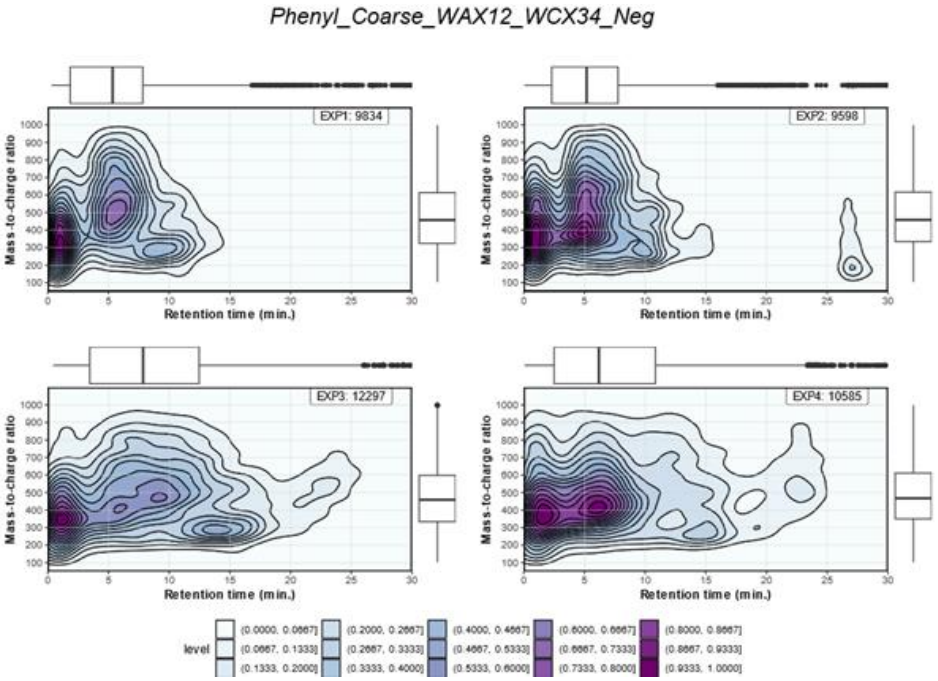


***Figure S9**: 2-dimensional density plot of reproducibly detectable metabolic features detected in sample DAX12DCX34 (a pool of Daphnia magna polar extract SPE fractions) under negative ionisation mode conditions using methods 'Ph-CO-1' (**top-left**), 'Ph-CO-2' (**top-right**), 'Ph-CO-3' (**bottom-left**) and 'Ph-CO-4' (**bottom-right**). Text boxes in the upper-right corner of each subplot indicate the total RDMFs.*



**Formatted:** Font: 9 pt, Font color: Auto

**Formatted:** Centered, Space Before: 0 pt, After: 0 pt

**Formatted:** Font: 9 pt, Do not check spelling or grammar, All caps

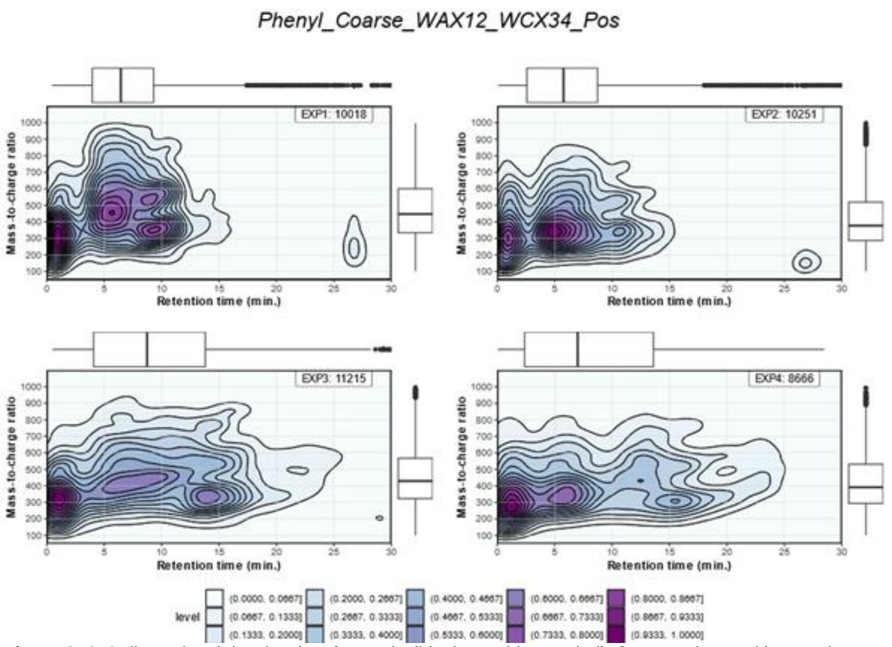


***Figure S10**: 2-dimensional density plot of reproducibly detectable metabolic features detected in sample DAX12DCX34 (a pool of Daphnia magna polar extract SPE fractions) under positive ionisation mode conditions using methods 'Ph-CO-1' (**top-left**), 'Ph-CO-2' (**top-right**), 'Ph-CO-3' (**bottom-left**) and 'Ph-CO-4' (**bottom-right**). Text boxes in the upper-right corner of each subplot indicate the total RDMFs.*

**Formatted:** Font: 9 pt, Font color: Auto

**Formatted:** Centered, Space Before: 0 pt, After: 0 pt

**Formatted:** Font: 9 pt, Do not check spelling or grammar, All caps

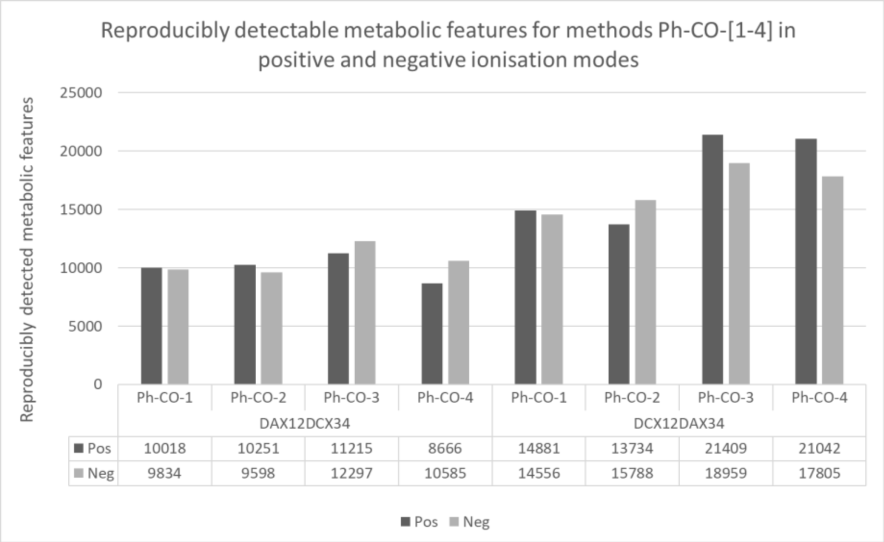


***Figure S11**: Count of reproducibly detectable metabolic features (RDMFs) detected in both positive (pos) and negative (neg) ionisation modes using methods 'Ph-CO-[1-4]' as part of phase one of Syncronis Phenyl-based (U)HPLC-HRMS(/MS) method optimisation.*

**Formatted:** Font: 9 pt, Font color: Auto

**Formatted:** Centered, Space Before: 0 pt, After: 0 pt

**Formatted:** Font: 9 pt, Do not check spelling or grammar, All caps

### 2.1.1.2 Phase two

Phase two of optimising a Syncronis Phenyl-based UHPLC-HRMS(/MS) method for DMA experiments, involved further refinement of the most performant method from the first phase of optimisation, method 'Ph-CO-3'. Three methods, 'Ph-FO-1', 'Ph-FO-2' and 'Ph-FO-3' – each a partially adapted version of method 'Ph-CO-3' (see **Table S7** for details) – were therefore evaluated based on RDMF counts (**Figure S12**) and chromatographic distributions (**Figures S13** and **S14**; representative examples of the 2-dimensional distribution (*m/z* versus retention time) of recorded RDMFs) resulting from positive and negative ionisation mode analyses of samples DAX12DCX34 and DCX12DAX34.

Based on the sum total of RDMF counts recorded across each sample type and ionisation mode combination, as individually summarised in **Figure S12,** method 'Ph-FO-1' was found to outperform methods 'Ph-FO-2' and 'Ph-FO-3', yielding 19687 RDMFs in total compared to 16379 and 16205 RDMFs for methods 'Ph-FO-2' and 'Ph-FO-3', respectively (note, no dereplication was applied to account for features detected under multiple conditions). This result is underpinned by the greater number of RDMFs recorded using method 'Ph-FO-1', compared to 'Ph-FO-2' and 'Ph-FO-3', for all ionisation mode and sample type combinations considered, except for positive mode analysis of sample DCX12DAX34. Method 'Ph-FO-1' was therefore identified as the best performing method in terms of absolute RDMF counts.

With regards to the chromatographic distribution of the RDMFs recorded using methods 'Ph-FO-[1-3]', median and interquartile range values for RDMF retention times were highly similar across all methods for analysis of DCX12DAX34, as summarised in **Table S12**. Under both positive and negative ionisation conditions, median retention times for RDMFs were, respectively: 6.5 (11.9) and 6.3 (9.5) minutes for method 'Ph-FO-1'; 6.8 (15.4) and 5.64 (11.3) minutes for method 'Ph-FO-2', and; 6.7 (11.8) and 6.6 (10.4) minutes for method 'Ph-FO-3 (interquartile ranges in parentheses). Method 'Ph-FO-2', with its larger interquartile range values in both positive and negative ionisation modes, was therefore found to offer superior separation of RDMFs in DCX12DAX34.

Median RDMF retention time values for analysis of DAX12DCX34 were strikingly different to those recorded for DCX12DAX34: 7.6 (12.5) and 5.1 (9.7) minutes for 'Ph-FO-1'; 1.2 (11.6) and 0.9 (5.5) minutes for 'Ph-FO-2', and; 3.1 (11.7) and 1.5 (8.6) minutes for 'Ph-FO-3' (interquartile ranges in parentheses). For methods 'Ph-FO-2' and 'Ph-FO-3' therefore, the first 50% of eluted RDMFs appeared to be highly congested at the start of the chromatographic elution space, a wholly undesirable situation for downstream application in the DMA analytical workflow, wherein extensive metabolome fractionation was sought prior to in-depth DI-HRMS(/MS$^n$) analyses. The exact causes of lower median RDMF retention time values for methods 'Ph-FO-2' and 'Ph-FO-3' were unclear. In part, this could potentially have been linked to the on-average wider peak widths for these methods, being 11.51 s (10.02 s) and 14.42 s (10.77 s) respectively, as compared to method 'Ph-FO-1' at 10.91 s (5.86 s) (parenthesis are IQRs). These wider peak widths may have deleteriously impacted the effectiveness of computational peak picking, resulting in an artificially lower number of RDMFs than were truly present. Nevertheless, even if peaks were accurately detected, their broadness would have been highly undesirable for application in the DMA analytical workflow. Method 'Ph-FO-1', with its superior RDMF counts

**Formatted:** Font: 9 pt, Font color: Auto

**Formatted:** Centered, Space Before: 0 pt, After: 0 pt

**Formatted:** Font: 9 pt, Do not check spelling or grammar, All caps

and acceptable RDMF distributions, was therefore selected for inclusion in the DMA analytical workflow.

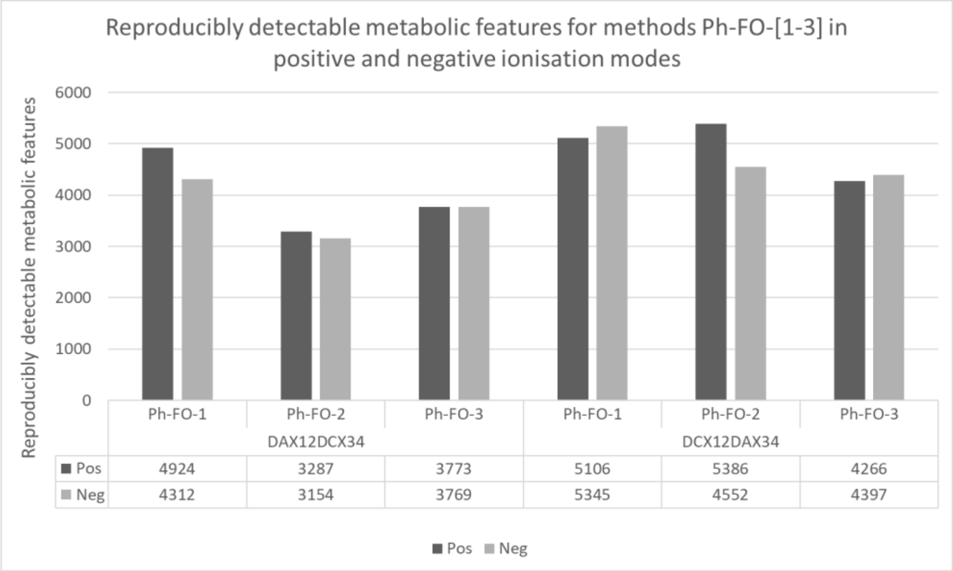


***Figure S12**: Counts of reproducibly detectable metabolic features (RDMFs) detected in both positive ('pos') and negative ('neg') ionisation modes using methods 'Ph-FO-[1-3]' during phase two of Syncronis Phenyl-based (U)HPLC-HRMS(/MS) method optimisation.*

**Formatted:** Font: 9 pt, Font color: Auto

**Formatted:** Centered, Space Before: 0 pt, After: 0 pt

**Formatted:** Font: 9 pt, Do not check spelling or grammar, All caps

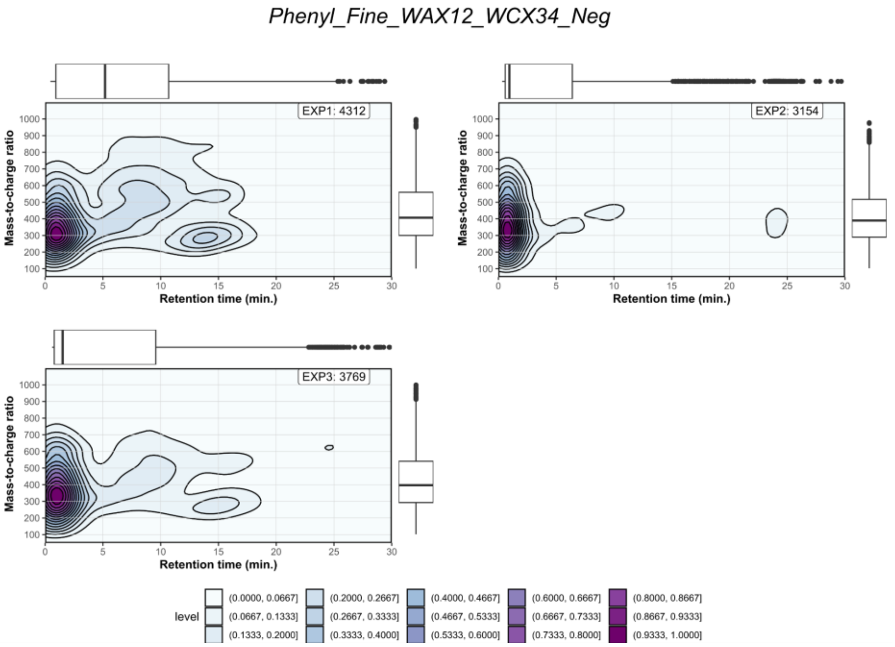


***Figure S13**: 2-dimensional density plot of reproducibly detectable metabolic features detected in sample DAX12DCX34 (a pool of Daphnia magna polar extract SPE fractions) under negative ionisation mode conditions using methods 'Ph-FO-1' (**top-left**), 'Ph-FO-2' (**top-right**) and 'Ph-FO-3' (**bottom-left**). Text boxes in the upper-right corner of each subplot indicate the total RDMFs.*

**Formatted:** Font: 9 pt, Font color: Auto

**Formatted:** Centered, Space Before: 0 pt, After: 0 pt

**Formatted:** Font: 9 pt, Do not check spelling or grammar, All caps

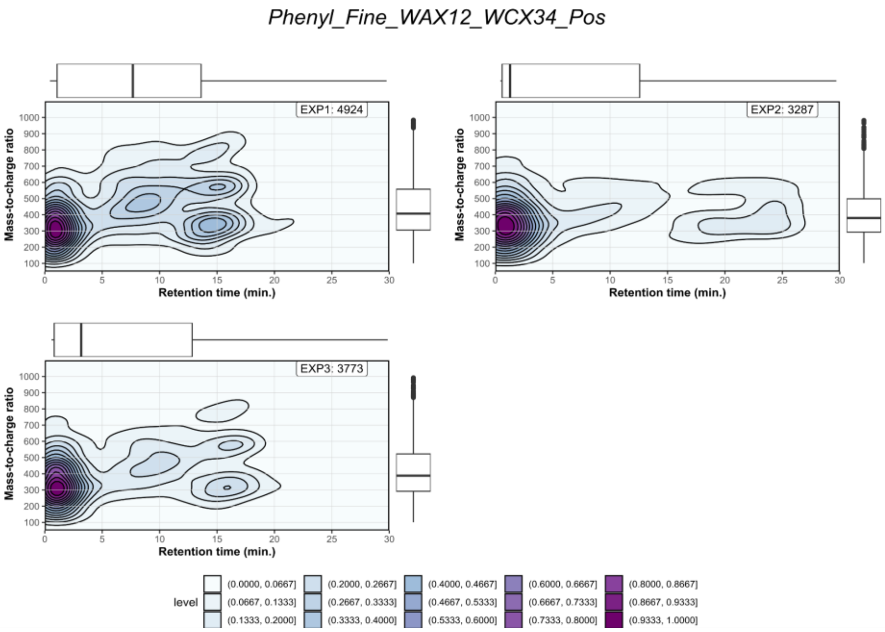


***Figure S14**: 2-dimensional density plot of reproducibly detectable metabolic features detected in sample DAX12DCX34 (a pool of Daphnia magna polar extract SPE fractions) under positive ionisation mode conditions using methods 'Ph-FO-1' (**top-left**), 'Ph-FO-2' (**top-right**) and 'Ph-FO-3' (**bottom-left**). Text boxes in the upper-right corner of each subplot indicate the total RDMFs.*

**Formatted:** Font: 9 pt, Font color: Auto

**Formatted:** Centered, Space Before: 0 pt, After: 0 pt

**Formatted:** Font: 9 pt, Do not check spelling or grammar, All caps

## 2.1.2 Accucore Amide (AMD)

### 2.1.2.1 Phase one

Phase one of optimising an Accucore Amide-based HILIC-HRMS(/MS) method for DMA, involved exploration of the impact of coarse shifts in mobile phase pH (spanning column operational limits) and additive type on RDMF counts and distributions.

**Figure S15**, below, summarises the count of RDMFs recorded in samples DAX12DCX34 and DCX12DAX34, when analysed by methods 'AA-CO-[1-3]' under positive and negative ionisation mode conditions. Sum totals of RDMFs across each combination of sample type and ionisation mode were: 13368 for method 'AA-CO-1' (pH 3.0), 12957 for method 'AA-CO-2' (pH 6.6), and 13476 for method 'AA-CO-3' (pH 5.8). Based on these values, no single method configuration (i.e. mobile phase pH and additive type combination) could be regarded as significantly more performant than any of the other methods tested. Likewise, the individual RDMF values for each combination of ionisation mode and sample type, as presented in **Figure S15**, revealed that no single analytical configuration was substantially superior. Greater numbers of RDMFs were however, evidently recorded when positive mode ionisation was applied, indicating potentially distinct chromatographic distributions of anionic and cationic metabolites.

The chromatographic distribution of RDMFs recorded using methods 'AA-CO-[1-3]' (see **Figures S16** and **S17** for representative examples of the 2-dimensional (*m/z* versus retention time) distribution of RDMFs recorded with these methods) provided additional insights into the relative similarities and differences of each method. As summarised in **Table S12**, median retention times for RDMFs detected in sample DCX12DAX34 were found to be markedly lower for method 'AA-CO-1', at 3.1 min in positive mode and 2.7 min in negative mode, compared to methods 'AA-CO-2' or 'AA-CO-3', where corresponding medians were 4.3 and 4.4 min, and 4.1 and 4.0 min, respectively. For the same sample, interquartile range values of RDMF retention times were similar, being 5.7, 5.7 and 5.4 min in positive mode, and 5.9, 5.8 and 5.4 min in negative mode, for methods 'AA-CO-1', 'AA-CO-2', and 'AA-CO-3', respectively. Together, these values indicated that lower chromatographic resolution was achieved for the first 50% of RDMFs eluted using method 'AA-CO-1', compared to methods 'AA-CO-2' and 'AA-CO-3'. Conversely, the larger span of time between the median and upper quartile of RDMF retention times for method 'AA-CO-1', indicated potential superior separation of metabolites during this elution period compared to 'AA-CO-2' and 'AA-CO-3'. A possible explanation for this observation is that sample DCX12DAX34, by virtue of its constituent SPE fractions, may have been enriched with anionic metabolites. Under the acidic elution conditions of method 'AA-CO-1' (pH 3.0), dissociation of any weakly acidic constituents may have been fully or partially suppressed, in so doing potentially reducing their capacity to interact with the HILIC stationary phase and/or to partition into the aqueous-rich layer thereon, resulting in decreased retention times. With rapid elution of these components, the latter part of the chromatogram may appear to offer greater metabolite separation, due simply to fewer metabolic features remaining to be eluted.

The chromatographic distribution of RDMFs recorded in sample DAX12DCX34 were, unlike those in sample DCX12DAX34, highly similar across methods 'AA-CO-[1-3]'. Median retention

**Formatted:** Font: 9 pt, Font color: Auto

**Formatted:** Centered, Space Before: 0 pt, After: 0 pt

**Formatted:** Font: 9 pt, Do not check spelling or grammar, All caps

time values of RDMFs were 2.4, 2.4 and 2.8 mins in positive ionisation mode, and 2.5, 2.3 and 2.6 mins in negative ionisation mode, respectively. Corresponding interquartile ranges were 5.1, 3.6 and 4.4 mins, and 5.0, 3.9 and 4.2 mins, indicating slightly superior separation of metabolites by method ‘AA-CO-1’.

Given that methods ‘AA-CO-[1-3]’ achieved similar performance in terms of both the distribution and number of RDMFs recorded, method ‘AA-CO-3’ was ultimately selected for further optimisation on the basis that its operational pH, pH 5.8, may facilitate ionisation (and thus promote retention under HILIC conditions) of a broad range of both anionic and cationic metabolites.

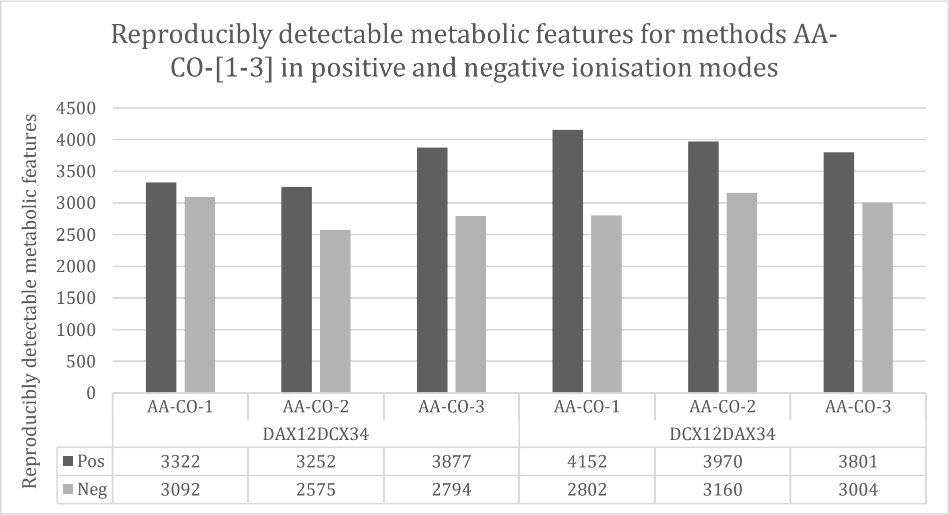


***Figure S15*** ***-*** *Counts of reproducibly detectable metabolic features (RDMFs) detected in samples DAX12DCX34 and DCX12DAX34 using methods ‘AA-CO-[1-3]’, under both positive (‘pos’) and negative (‘neg’) ionisation mode conditions, during phase one of Accucore Amide-based HILIC-HRMS(/MS) method optimisation.*

**Formatted:** Font: 9 pt, Font color: Auto

**Formatted:** Centered, Space Before: 0 pt, After: 0 pt

**Formatted:** Font: 9 pt, Do not check spelling or grammar, All caps

AccucoreAmide_Coarse_WAX12_WCX34_Neg

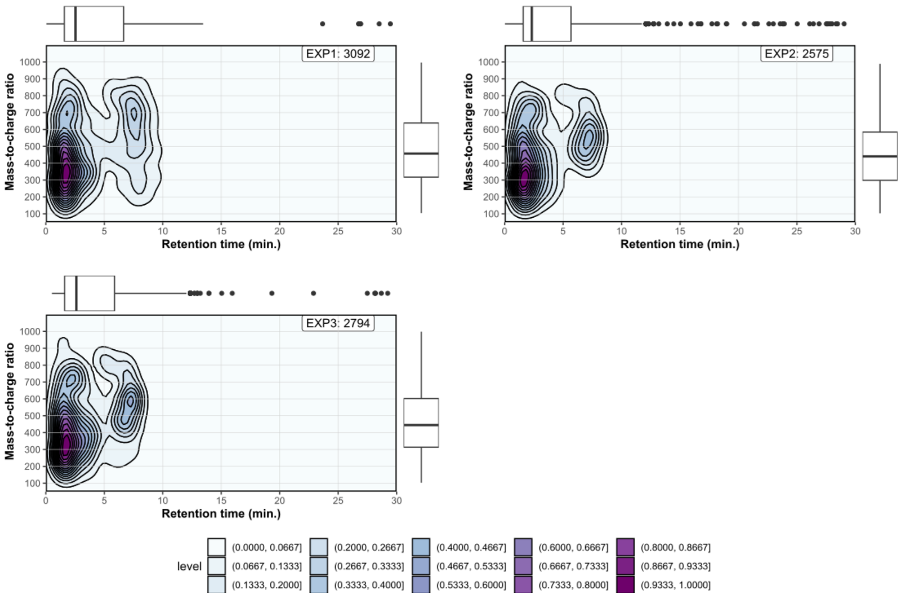


***Figure S16**: 2-dimensional density plot of reproducibly detectable metabolic features (RDMFs) detected through negative ionisation mode analysis of sample DAX12DCX34 using methods 'AA-CO-1' (**top-left**), 'AA-CO-2' (**top-right**) and 'AA-CO-3 (**bottom-left**) during phase one of Accucore Amide-based HILIC-HRMS(/MS) method optimisation. Text boxes in the upper-right corner of each subplot indicate the total RDMFs.*

**Formatted:** Font: 9 pt, Font color: Auto

**Formatted:** Centered, Space Before: 0 pt, After: 0 pt

**Formatted:** Font: 9 pt, Do not check spelling or grammar, All caps

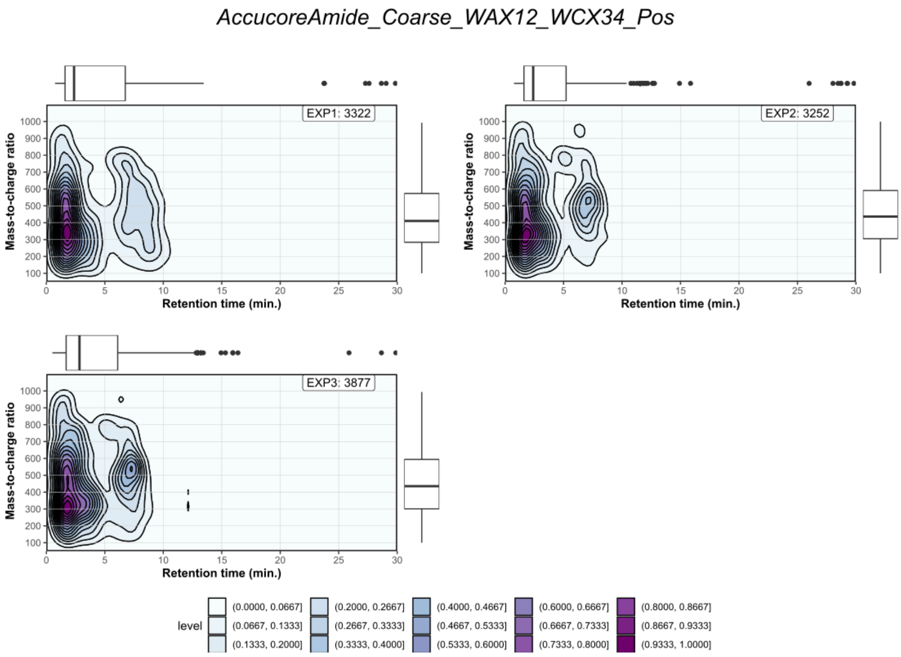


***Figure S17:*** *2-dimensional density plot of reproducibly detectable metabolic features (RDMFs) detected through positive ionisation mode analysis of sample DAX12DCX34 using methods 'AA-CO-1' (**top-left**), 'AA-CO-2' (**top-right**) and 'AA-CO-3 (**bottom-left**) during phase one of Accucore Amide-based HILIC-HRMS(/MS) method optimisation. Text boxes in the upper-right corner of each subplot indicate the total RDMFs.*

**Formatted:** Font: 9 pt, Font color: Auto

**Formatted:** Centered, Space Before: 0 pt, After: 0 pt

**Formatted:** Font: 9 pt, Do not check spelling or grammar, All caps

### 2.1.2.2 Phase two

Resuspension of dried SPE fractions proved a considerable challenge when establishing an effective HILIC-HRMS(/MS) method for DMA. Indeed, during the first phase of HILIC-HRMS(/MS) method optimisation, SPE fraction resuspension required multiple bursts in an ultrasonic bath, interspersed by vigorous vortex mixing, to achieve effective dissolution of the dried pellets. The high salt content of dried SPE fractions, coupled with the potentially poor solubility of 'polar' metabolites in 90% v/v ice-cold acetonitrile:aqueous buffer, were hypothesised as major contributory factors in this regard.

Phase two of HILIC-HRMS(/MS) method optimisation sought to improve the efficiency (i.e. making the process faster and reducing the likelihood of promoting metabolite degradation) and effectiveness (i.e. ensuring as many metabolites as possible are resuspended) of the procedures used for SPE fraction resuspension, prior to DMA by HILIC-HRMS(/MS). A single analytical method, adapted from method 'AA-CO-3' (see **Supplemental Section 1.9.3.2**), was used to evaluate three distinct resuspension processes, 'AA-Resus-[1-3]'.

**Figure S18**, below, demonstrates the number of RDMFs recorded in samples DAX12DCX34 and DCX12DAX34 following application of the three resuspension methods tested, 'AA-RESUS-[1-3]'. Sum totals of RDMFs across sample type and ionisation mode were: 14789 for 'AA-Resus-1', 10580 for 'AA-Resus-2', and 8947 for 'AA-Resus-3'. Method 'AA-Resus-1' – a replica of the method used for fraction resuspension during the first phase of HILIC-HRMS(/MS) method development (see **Supplemental Section 1.9.3.1**) – thus permitted detection of the greatest number of RDMFs. This remained true when considering RDMFs counts for each combination of sample type or MS ionisation mode.

Distributions of RDMFs differed considerably for each of the resuspension methods, with median RDMF retention times consistently lower for 'AA Resus-2' and consistently greater for 'AA-Resus-3', as summarised in **Table S12**. Each method, therefore, appeared to demonstrate differing selectivity with respect to the types of metabolic constituents resuspended and effectively separated under HILIC conditions.

Comparison of RDMF distributions for methods 'AA-Resus 1' and 'AA-Resus-3' (see **Figures S19** and **S20**, below) revealed 48.5 to 62.7% fewer early-eluting (i.e. less-polar or more apolar) RDMFs – here defined as RDMFs with retention times less than 5 * LC retention factor – for 'AA-Resus-3'. The lower volume of ACN used for 'AA-Resus-3' resuspensions likely underpinned this observation. For later eluting RDMFs – defined as RDMFs with retention times > 5 * LC retention factor – 'AA-Resus-3' yielded 42.2% and 45.3% fewer RDMFs than 'AA-Resus-1' from analysis of DAX12DCX34 under negative and positive ionisation conditions, respectively. Similarly, 16.4% fewer RDMFs were recorded using 'AA-Resus-3' compared to 'AA-resus-1' when analysing sample DCX12DAX34 in positive ionisation mode, though 10.2% more RDMFs were recorded during negative ionisation mode analyses of the same sample, following 'AA-Resus-3'.

Comparison of the chromatographic distributions of RDMFs for methods 'AA-Resus-1' and 'AA-Resus-2' (see **Figures S19** and **S20**, below), revealed 10.7 to 26.5% fewer early-eluting

Formatted: Font: 9 pt, Font color: Auto

Formatted: Centered, Space Before: 0 pt, After: 0 pt

Formatted: Font: 9 pt, Do not check spelling or grammar, All caps

RDMFs (i.e. retention time < 5 * LC retention factor) for ‘AA-Resus-2’. The number of later-eluting RDMFs (i.e. retention time > 5 * LC retention factor) was also reduced relative to ‘AA-Resus-1’, with 25.5-27.1% fewer recorded RDMFs during analysis of DAX12DCX34, and 43.5% (positive mode) and 56.2% (negative mode) fewer during analysis of DCX12DAX34. These results build upon the conclusions of Ruta et al. (2010) who, using a limited panel of pre-dissolved probe molecules, suggested that comparable HILIC performance could be achieved where either 80:20% v/v ACN:DMSO or 100% v/v A-phase injection solvents were employed. In the present study, resuspension of dried polar extracts using 80:20% v/v ACN:DMSO mixtures was found to be substantially less effective for sample resuspension than using 100% mobile phase A. Indeed, given that ‘AA-Resus-2’ (resuspension using 80:20% v/v ACN:DMSO) employed the same sonication and vortex mixing procedures used for ‘AA-Resus-1’, but resulted in substantially fewer and less-well distributed RDMFs across all analyses conducted, ‘AA-Resus-2’ was ultimately excluded from further consideration as a method for metabolite resuspension in the DMA workflow.

A mismatch between sample injection solvent and initial LC eluent composition is known to adversely impact chromatographic performance. Chauve et al., (2010) highlight this as ‘the most important cause of performance loss and peak distortion’ in HILIC analyses, leading to peak splitting, tailing and fronting effects, particularly for earlier-eluting peaks. Nevertheless, due to poor solubility of various polar metabolites in ice-cold organic solvent (typically >90% v/v acetonitrile for HILIC analyses), such as those contained in the SPE fractions analysed in this work, mismatches are required to ensure effective solute dissolution. Use of a plugged sample injection procedure (such as that used to good effect by Johnson et al., (2010)), permits partial dilution of aqueous injection solvent – in this work reducing aqueous content from approximately 50% to 30% v/v. Given that method ‘AA-Resus-3’ yielded distinctly fewer early-eluting peaks than ‘AA-Resus-1’, a further reduction in the aqueous composition of the sample injection plug was speculated to be of importance for effective DMA. Indeed, Heaton and McCalley (2016) concluded that >20% v/v aqueous content should be avoided in the sample injection plug of HILIC-based separations, if early eluting peak shape is of importance. Hence, with a view to further improving the effectiveness of HILIC-HRMS(/MS) for DMA, methods ‘AA-Resus-3’ was carried forward for further optimisation in phase three of HILIC-HRMS(/MS) method optimisation, wherein method ‘AA-Resus-1’ was used to benchmark performance.

**Formatted:** Font: 9 pt, Font color: Auto

**Formatted:** Centered, Space Before: 0 pt, After: 0 pt

**Formatted:** Font: 9 pt, Do not check spelling or grammar, All caps

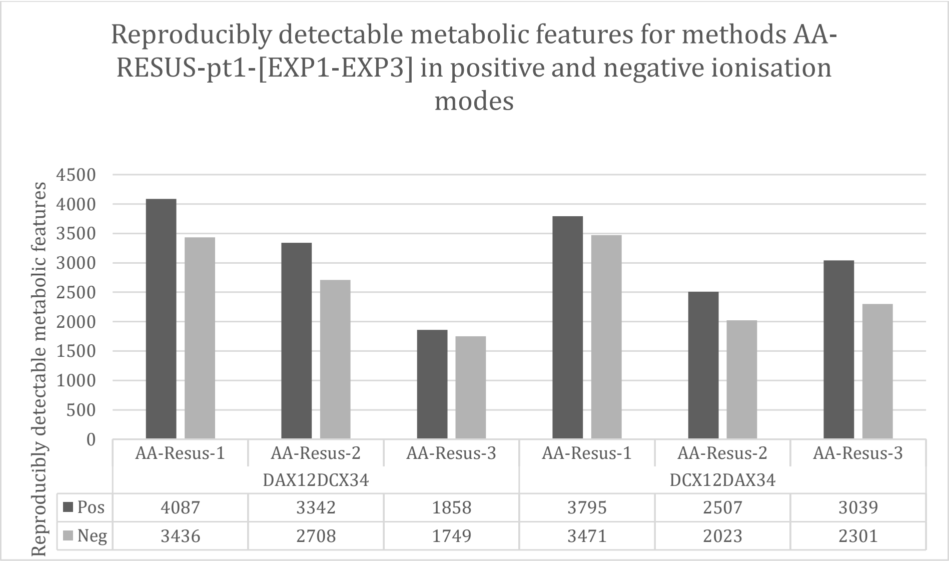


***Figure S18:*** *Counts of reproducibly detectable metabolic features (RDMFs) detected in both positive ('pos') and negative ('neg') ionisation modes using methods 'AA-RESUS-[1-3]' during phase two of Accucore Amide-based HILIC-HRMS(/MS) method optimisation.*

**Formatted:** Font: 9 pt, Font color: Auto

**Formatted:** Centered, Space Before: 0 pt, After: 0 pt

**Formatted:** Font: 9 pt, Do not check spelling or grammar, All caps

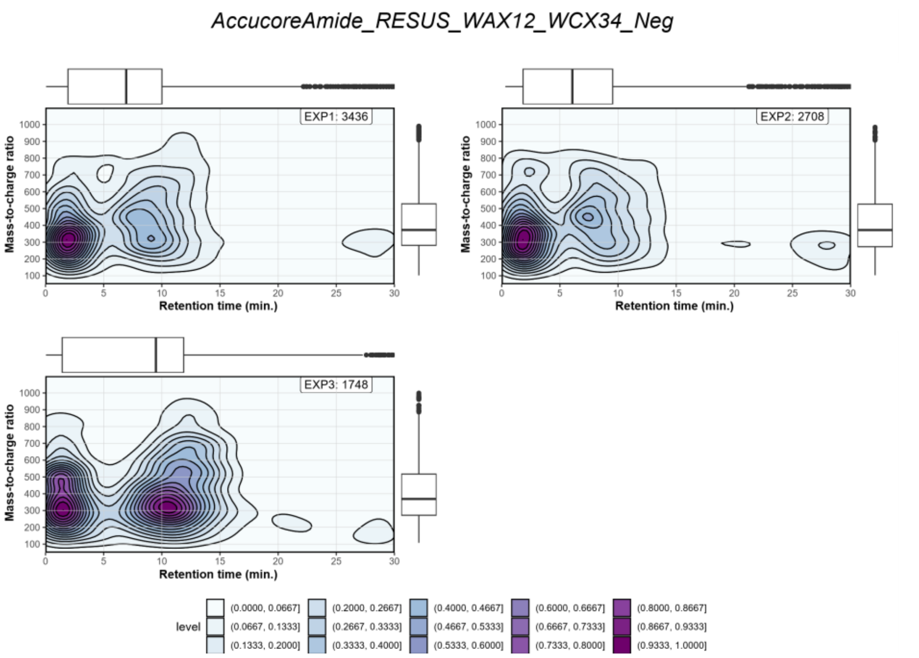


***Figure S19:*** *2-dimensional density plot of reproducibly detectable metabolic features (RDMFs) detected through negative ionisation mode analysis of sample DAX12DCX34 using methods 'AA-Resus-1' (**top-left**), 'AA-Resus-2' (**top-right**) and 'AA-Resus-3 (**bottom-left**), during phase two of Accucore Amide-based HILIC-HRMS(/MS) method optimisation. Text boxes in the upper-right corner of each subplot indicate the total RDMFs.*

**Formatted:** Font: 9 pt, Font color: Auto
**Formatted:** Centered, Space Before: 0 pt, After: 0 pt
**Formatted:** Font: 9 pt, Do not check spelling or grammar, All caps

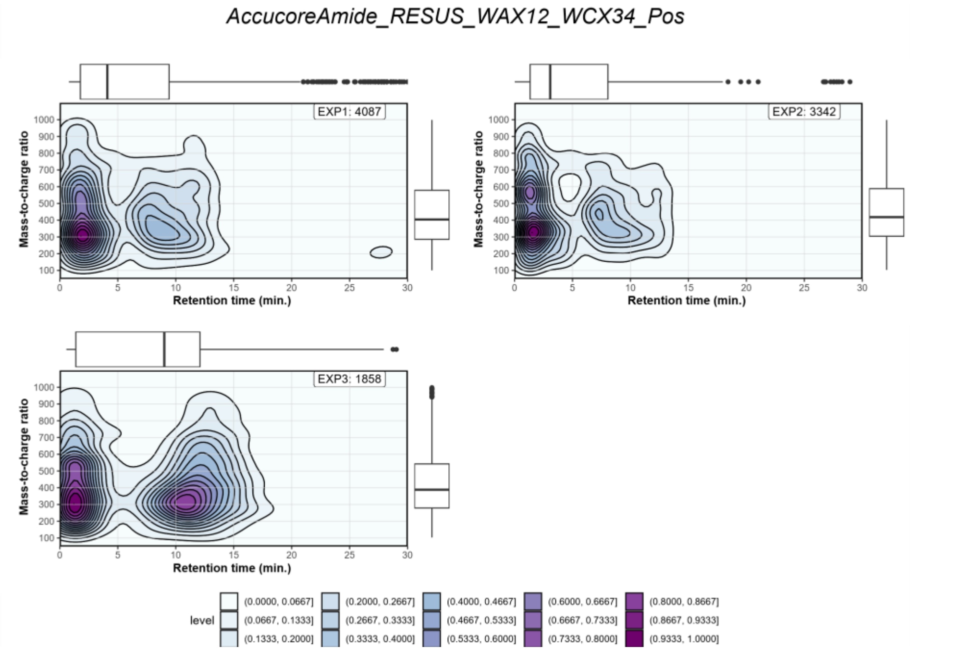


***Figure S20:*** *2-dimensional density plot of reproducibly detectable metabolic features (RDMFs) detected through positive ionisation mode analysis of sample DAX12DCX34 using methods 'AA-Resus-1' (**top-left**), 'AA-Resus-2' (**top-right**) and 'AA-Resus-3 (**bottom-left**), during phase two of Accucore Amide-based HILIC-HRMS(/MS) method optimisation. Text boxes in the upper-right corner of each subplot indicate the total RDMFs.*

**Formatted:** Font: 9 pt, Font color: Auto

**Formatted:** Centered, Space Before: 0 pt, After: 0 pt

**Formatted:** Font: 9 pt, Do not check spelling or grammar, All caps

### 2.1.2.3 Phase three

Phase three of HILIC-HRMS(/MS) method optimisation sought to further improve the procedures used for resuspension of polar extracts and associated SPE fractions in the DMA workflow, to maximise the quantity and quality of resulting HILIC-HRMS(/MS) data. Two distinct resuspension methods were accordingly trialled. Method 'AA-Resus-1' used mobile phase A as resuspension solvent, whereas method 'AA-Resus-4' used 20 µL 100 mM ammonium acetate buffer (aq., pH unadjusted) followed by 80 µL acetonitrile.

Application of 'AA-Resus-4' to the resuspension of samples DAX12DCX34 and DCX12DAX34 resulted in formation of biphasic solutions. Neither warming these solutions to room temperature, nor increasing their acetonitrile or water proportions, served to disrupt their biphasic state. Spiking of 20 µL ice-cold methanol did however dissipate the biphase, as evidenced by the visibly uniform distribution of coloured metabolome constituents (presumably pigments, such as carotenoids) throughout resulting monophasic solutions. Method 'AA-Resus-4' thereby yielded solutions of 20% greater volume than 'AA-Resus-1', i.e. metabolites were potentially more diluted.

Based solely on the number of RDMFs recorded using resuspension methods 'AA-Resus-1' and 'AA-Resus-4', as summarised in **Figure S21,** below, neither resuspension procedure was found to be distinctly more performant for HILIC-HRMS(/MS)-based DMA. Sums of RDMFs counts across each sample type and ionisation mode combination were similar for each method, yielding 6741 and 7001 RDMFs, respectively. Method 'AA-Resus-1' did however outperform method 'AA-Resus-4' in terms of the number of RDMFs recorded in sample DAX12DCX34 under both positive and negative ionisation conditions, though the opposite was true for analysis of sample DCX12DAX34.

Inspection of the chromatographic distribution of RDMFs recorded using methods 'AA-Resus-1' and 'AA-Resus-4', as demonstrated in **Figures S22** and **S23,** below, revealed striking differences between the two methods. The early-eluting region of resulting chromatograms (defined as per **Supplemental Section 2.1.2.2**; equating to < 4.4 to 4.7 minutes) contained between 50 and 67% fewer RDMFs when 'AA-Resus-4' was used for sample resuspension as opposed to 'AA-Resus-1'. Conversely, the later eluting regions (> 4.4 to 4.7 minutes) contained 66.7 and 107.1% more RDMFs for sample DAX12DCX34, and 248 and 363.7% (positive and negative mode values, respectively) for sample DCX12DAX34, when 'AA-Resus-4' was used for sample resuspension instead of 'AA-Resus-1'.

Given that a HILIC-HRMS(/MS) method was included in the DMA workflow to facilitate HRMS(/MS) analysis of as many (predominantly) polar metabolites as possible, resuspension method 'AA-Resus-4', which yielded data on more later-eluting (and thus presumably more polar) metabolites, was selected as the method of choice for sample resuspension in the DMA workflow. Those metabolites missed by method 'AA-Resus-4', i.e. those detected in the early elution window of the HILIC-HRMS(/MS) method, were assumed to have more apolar characteristics and thus likely better suited to analysis under reversed-phase conditions (such as those established in **Supplemental Sections 2.1.1.1** and **2.1.1.2**).

**Formatted:** Font: 9 pt, Font color: Auto

**Formatted:** Centered, Space Before: 0 pt, After: 0 pt

**Formatted:** Font: 9 pt, Do not check spelling or grammar, All caps

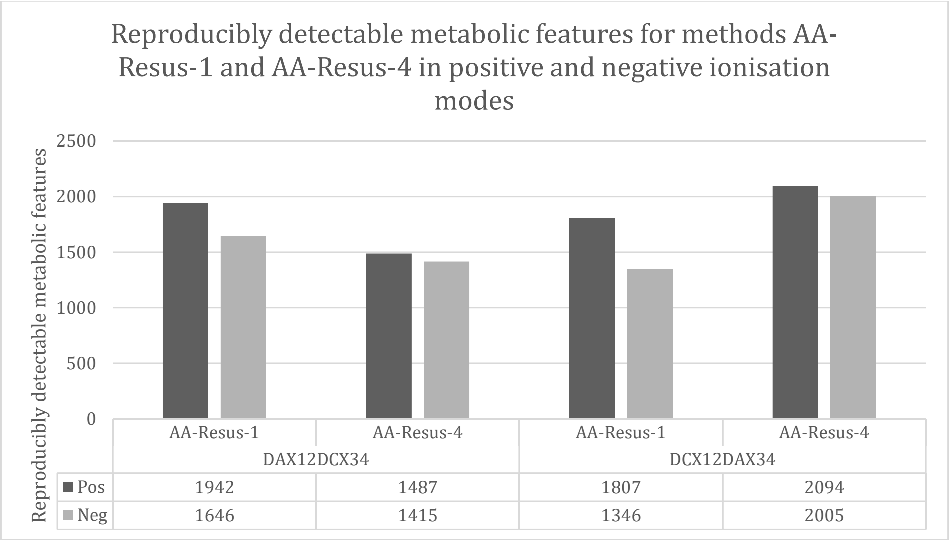


***Figure S21:*** *Counts of reproducibly detectable metabolic features (RDMFs) detected in both positive ('pos') and negative ('neg') ionisation modes using methods 'AA-Resus-1' and 'AA-Resus-4' during phase three of Accucore Amide-based HILIC-HRMS(/MS) method optimisation.*

**Formatted:** Font: 9 pt, Font color: Auto

**Formatted:** Centered, Space Before: 0 pt, After: 0 pt

**Formatted:** Font: 9 pt, Do not check spelling or grammar, All caps

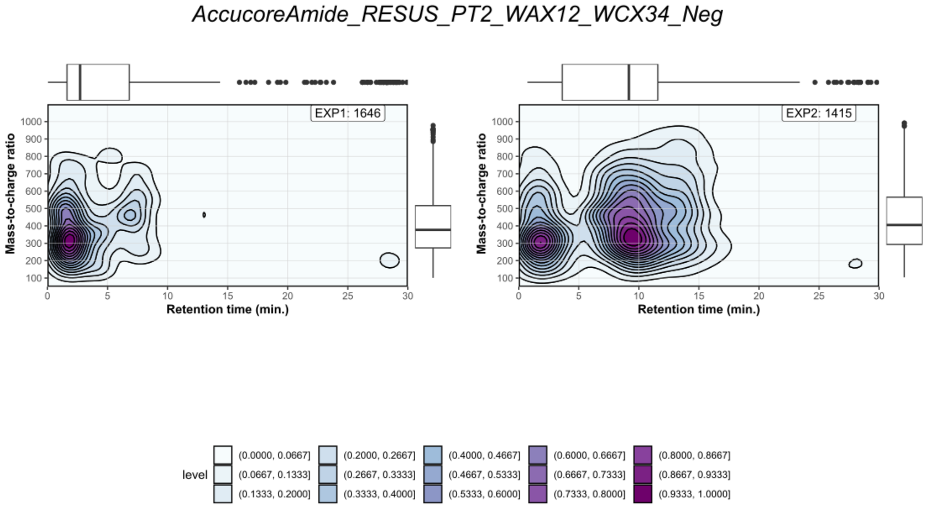


***Figure S22:*** *2-dimensional density plot of reproducibly detectable metabolic features (RDMFs) detected through negative ionisation mode analysis of sample DAX12DCX34 using methods 'AA-Resus-1' (**left**) and 'AA-Resus-4' (**right**), during phase three of Accucore Amide-based HILIC-HRMS(/MS) method optimisation. Text boxes in the upper-right corner of each subplot indicate the total RDMFs.*

**Formatted:** Font: 9 pt, Font color: Auto

**Formatted:** Centered, Space Before: 0 pt, After: 0 pt

**Formatted:** Font: 9 pt, Do not check spelling or grammar, All caps

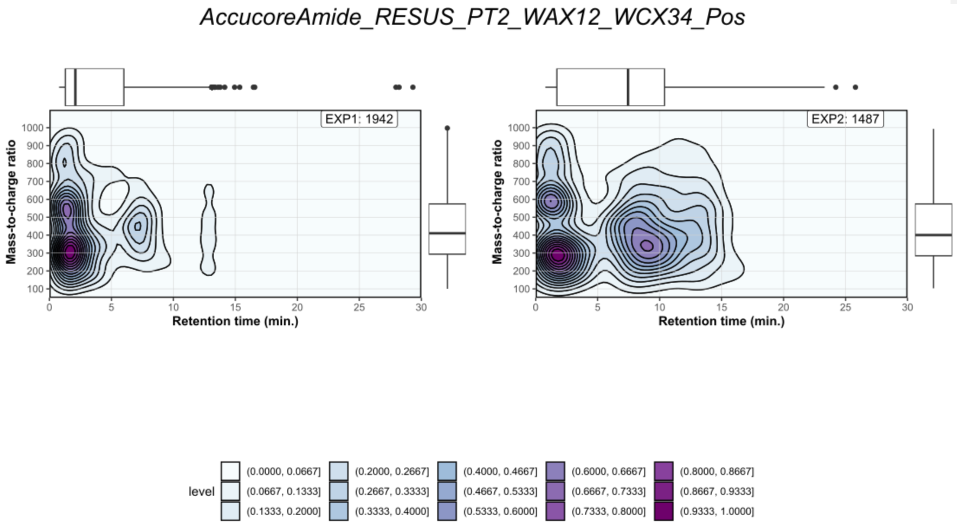


***Figure S23:*** *2-dimensional density plot of reproducibly detectable metabolic features (RDMFs) detected through positive ionisation mode analysis of sample DAX12DCX34 using methods 'AA-Resus-1' (**left**) and 'AA-Resus-4' (**right**), during phase three of Accucore Amide-based HILIC-HRMS(/MS) method optimisation. Text boxes in the upper-right corner of each subplot indicate the total RDMFs.*

**Formatted:** Font: 9 pt, Font color: Auto

**Formatted:** Centered, Space Before: 0 pt, After: 0 pt

**Formatted:** Font: 9 pt, Do not check spelling or grammar, All caps

### 2.1.2.4 Phase four

The fourth and final phase of optimising a HILIC-HRMS(/MS) method for DMA, involved broad reappraisal of the conditions used to effect HILIC separations. This was undertaken in response to the significant changes made to the HILIC-HRMS(/MS) sample resuspension protocol in prior optimisation steps, which substantially altered the types of metabolites accessible to this method. Six methods were ultimately trialled, methods 'AA-FO-[1-6]'.

Counts of RDMFs recorded using methods 'AA-FO-[1-6]' are summarised in **Figure S24**, below. Strikingly, analyses conducted using method 'AA-FO-1' – a partially modified version of the most performant HILIC-HRMS(/MS) method from earlier optimisation experiments (see **Supplemental Sections 1.9.3 and 2.1.2.1-2.1.2.3** for method details) – resulted in between 34.1 and 53.4% fewer RDMFs than method 'AA-FO-2'. The latter was a replica of method 'AA-FO-1', except for injection volume having been halved and flow rate doubled. The substantially lower RDMF counts for 'AA-FO-1' are hypothesised to stem from use of an overly large injection volume, which can deleteriously impact HILIC performance, including through peak fronting, tailing and, in extreme cases, complete peak loss (Chauve et al., 2010; Heaton and McCalley, 2016; Ruta et al., 2010; Vorkas et al., 2015). Such effects have been proposed to arise, as in RPLC, due to the differences in relative strengths and viscosities of the injection and mobile phase solutions (Heaton and McCalley, 2016). The lower injection volume of 'AA-FO-2', in combination with higher flow rate, would have led to greater dilution of the injection solvent and thereby likely helped to minimise solvent mismatch effects, resulting in improved chromatographic performance. For this reason, a 5 µL injection volume and 400 µL $min^{-1}$ flow rate were used in the final DMA HILIC-HRMS(/MS) method.

Comparison of the sum total of RDMFs recorded (across each sample type and ionisation mode) using methods 'AA-FO-4' and 'AA-FO-2', or methods 'AA-FO-5' and 'AA-FO-3', revealed that substantially more RDMFs were recorded where pH-adjusted ammonium acetate buffers (aq., pH 5.8; as in methods 'AA-FO-4' and 'AA-FO-5') were used for mobile phase preparation and sample resuspension, rather than unmodified ammoniate acetate solutions (as in methods 'AA-FO-2' and 'AA-FO-3'). Sum totals were 16869 and 14993, and 14105 and 12776, respectively. Methods 'AA-FO-4' and 'AA-FO-2', and methods 'AA-FO-5' and 'AA-FO-3' were each equivalent, except for the aqueous buffer used. It is noted that methods 'AA-FO-4' and 'AA-FO-5' also required 10 µL of additional methanol to be spiked into resuspended samples to maintain a monophasic state, meaning samples were also somewhat more dilute than those analysed by methods 'AA-FO-2' and 'AA-FO-3'.

Through comparison of RDMFs counts for methods 'AA-FO-4' and 'AA-FO-5', and methods 'AA-FO-2' and 'AA-FO-3', it was observed that the rate at which aqueous volume percentage increased throughout the gradient elution program impacted both the total number and distribution of RDMFs recorded. Unexpectedly, it was the higher aqueous ramp rates applied in methods 'AA-FO-2' and 'AA-FO-4', wherein mobile phase 'B' constituted 60% v/v water, that gave rise to the greater number of RDMFs. In agreement with expectations however, the shallower ramp rates of aqueous volume fraction in methods 'AA-FO-3' or 'AA-FO-5' resulted in wider distributions of RDMFs, as shown in **Figures S25** and **S26**, below. A trade off therefore existed with respect to selecting an 'optimal' set of parameters for HILIC separations.

Formatted: Font: 9 pt, Font color: Auto

Formatted: Centered, Space Before: 0 pt, After: 0 pt

Formatted: Font: 9 pt, Do not check spelling or grammar, All caps

Fortuitously, method ‘AA-FO-6’ afforded an excellent compromise, yielding the second highest RDMF sum total across all evaluated methods (15293 RDMFs recorded across all sample types and ionisation modes) – second only to ‘AA-FO-4’ (16869 RDMFs across all sample types and ionisation mode). Methods ‘AA-FO-6’ also consistently gave rise to the widest distribution of RDMFs, as evidenced by the interquartile range of RDMF retention times (see **Table S12**). Method ‘AA-FO-6’ was therefore selected as the HILIC-HRMS(/MS) method for inclusion in the DMA analytical workflow.

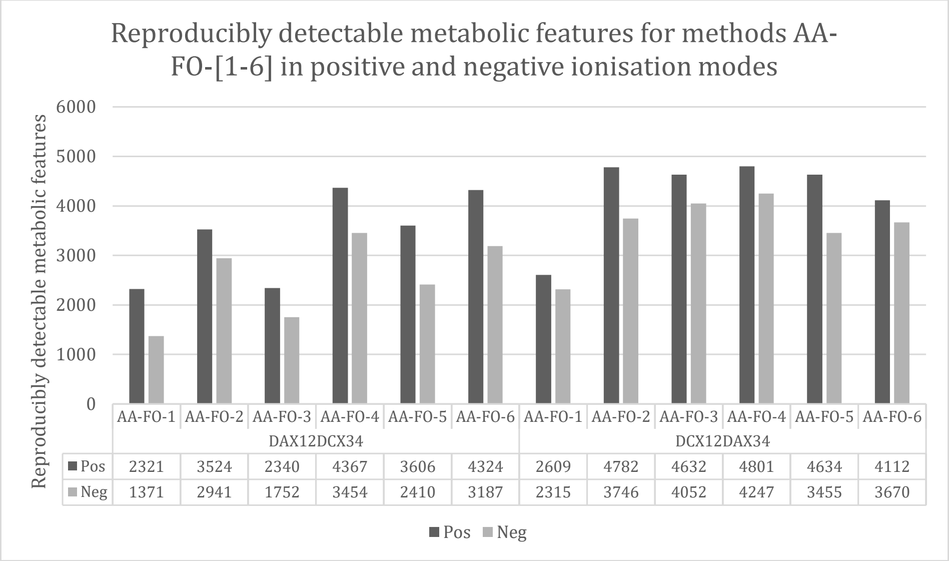


***Figure S24:*** *Counts of reproducibly detectable metabolic features (RDMFs) detected in both positive (‘pos’) and negative (‘neg’) ionisation modes using methods ‘AA-FO-[1-6]’ during phase four of Accucore Amide-based HILIC-HRMS(/MS) method optimisation.*

**Formatted:** Font: 9 pt, Font color: Auto

**Formatted:** Centered, Space Before: 0 pt, After: 0 pt

**Formatted:** Font: 9 pt, Do not check spelling or grammar, All caps

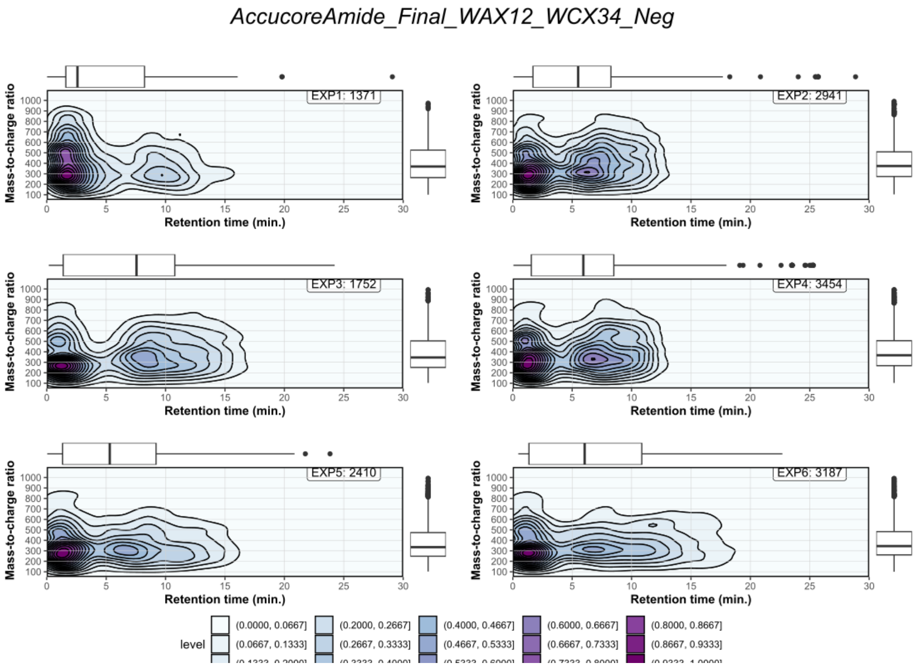


***Figure S25**: 2-dimensional density plot of reproducibly detectable metabolic features (RDMFs) detected through negative ionisation mode analysis of sample DAX12DCX34 using methods 'AA-FO-1' (**top-left**), 'AA-FO-2 (**top-right**), 'AA-FO-3' (**centre-left**), 'AA-FO-4' (**centre-right**), 'AA-FO-5' (**bottom-left**) and 'AA-FO-6' (**bottom-right**) during phase four of Accucore Amide-based HILIC-HRMS(/MS) method optimisation. Text boxes in the upper-right corner of each subplot indicate the total RDMFs.*



Formatted: Font: 9 pt, Font color: Auto
Formatted: Centered, Space Before: 0 pt, After: 0 pt
Formatted: Font: 9 pt, Do not check spelling or grammar, All caps

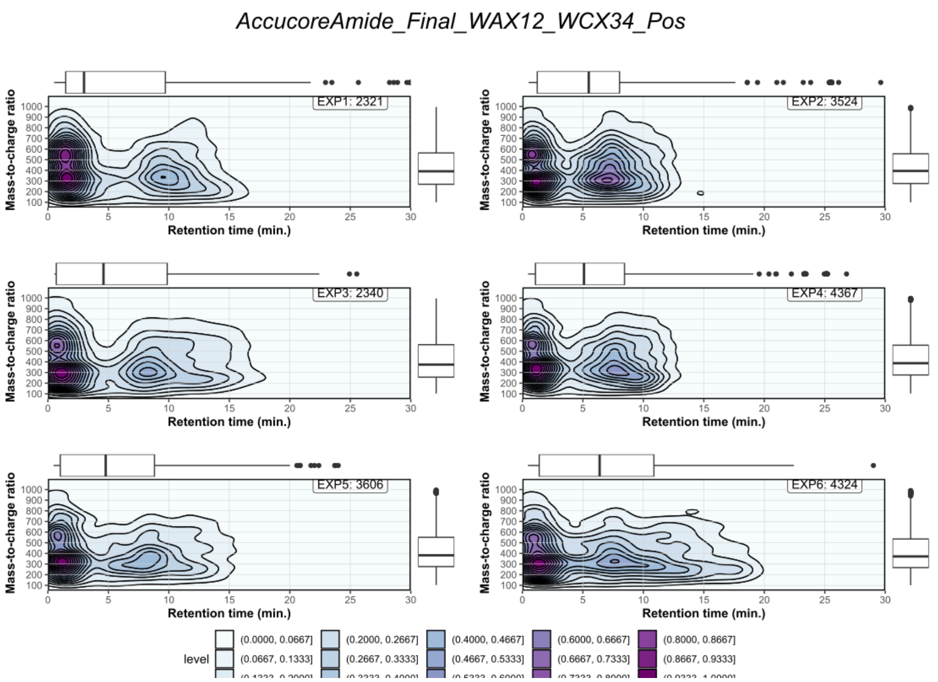


***Figure S26****: : 2-dimensional density plot of reproducibly detectable metabolic features (RDMFs) detected through positive ionisation mode analysis of sample DAX12DCX34 using methods 'AA-FO-1' (****top-left****), 'AA-FO-2 (****top-right****), 'AA-FO-3' (****centre-left****), 'AA-FO-4' (****centre-right****), 'AA-FO-5' (****bottom-left****) and 'AA-FO-6' (****bottom-right****) during phase four of Accucore Amide-based HILIC-HRMS(/MS) method optimisation. Text boxes in the upper-right corner of each subplot indicate the total RDMFs.*

**Formatted:** Font: 9 pt, Font color: Auto

**Formatted:** Centered, Space Before: 0 pt, After: 0 pt

**Formatted:** Font: 9 pt, Do not check spelling or grammar, All caps

## 2.2 Summary of all DMA of D. magna annotations

See **Supplementary Table S13** (provided in separate excel file) for a summary of all annotations for *D. magna.*

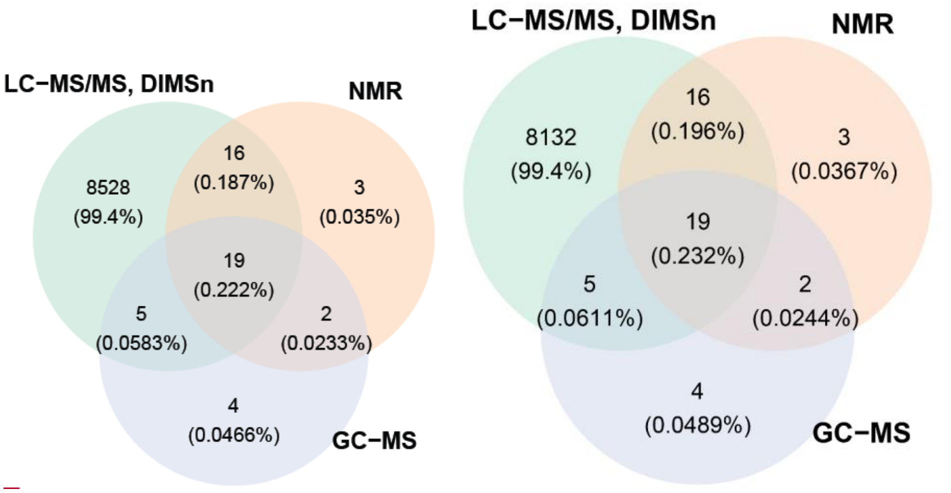


***Figure S27: Venn diagram of metabolite annotations observed for 1D- & 2D-NMR, GC-EI-HRMS and (U)HPLC-HRMS(/MS) and DI-HRMS(/MS$^n$) measurement techniques***

**Formatted:** Font: 9 pt, Font color: Auto

**Formatted:** Centered, Space Before: 0 pt, After: 0 pt

**Formatted:** Font: 9 pt, Do not check spelling or grammar, All caps

## 2.3 (U)HPLC-HRMS(/MS) and DI-HRMS(/MS$^n$) derived metabolite annotations

We report 8,1728,568 MSI level 2 annotated metabolites across all (U)HPLC-HRM(/MS) & DI-HRMS(/MS$^n$) assays.

(U)HPLC-HRMS(/MS) & DI-HRMS(/MS$^n$) annotations were derived from three main computational annotation approaches: spectral matching, SIRIUS CSI:FingerID and MetFrag. SIRIUS CSI:FingerID consistently provided more annotations than any other approach (5,817 5,980 unique annotations in total, 4,581 4,685 of which were unique to the approach), followed by MetFrag (1,9512,091 unique annotations, 1,1741,075 unique to the approach) and then spectral matching (1,8802,043 unique annotations, 1,3591,230 unique to the approach). The higher number of annotations observed from SIRIUS CSI:FingerID is partly down to the filtering used for this approach (choosing the top ranked annotation) whereas MetFrag and Spectral Matching enabled much more stringent filtering criteria to be applied based on the multiple scoring outputs. We note that SIRIUS CSI;FingerID now has the COSMOS score but that was not implemented within Galaxy Workflow tool at the time of analysis. Additionally, with the version of SIRIUS used, the COSMOS score was only provided for the top ranked CSI:FingerID annotation so could not be applied to all annotations derived from SIRIUS CSI:FingerID Galaxy tool. The primary reason for MetFrag not exceeding the number of annotations of the other approaches is due to the very stringent filtering criteria on the MetFrag annotations (chosen based on manual inspection of annotations observed at different thresholds of the MetFrag score) and the use of a wide range of libraries for spectral matching.

Spectral matching was performed using 3 different workflows or tools: The "Galaxy workflow" approach – where spectral matching was performed via the Galaxy workflow tool msPurity.spectralMatching applied to averaged fragmentation spectra; The "mzCloud" approach – spectral matching performed directly from the raw mass spectrometry files against the mzCloud spectral database; and the "GNPS workflow" approach – where the mzML files are searched against the GNPS public spectral databases. The "Galaxy workflow" spectral matching approach gave 1,0441,065 unique annotations (775784 unique to the approach), the GNPS workflow spectral matching approach gave 822963 unique annotations (619532 unique to the approach) and the mzCloud approach gave 415477 unique annotations (288255 unique to the approach). The Galaxy workflow and GNPS workflow were expected to have a higher number of annotations due to the larger spectral databases used (derived from various sources and instrument types) whereas the mzCloud library uses a smaller but more specific library for Thermo Scientific's instruments, including Orbitrap mass spectrometers (i.e. the same instrument type used for the experimental analyses performed here).

Annotation to a full InChiKey is difficult using only mass spectrometry fragmentation analysis without additional chromatography investigations and can be challenging to distinguish between the stereochemistry encoded in the second component of an InChiKey. If we only consider the first component of the InChiKey (encoding the "molecular skeleton" of the structure) we observe 7,6678,029 annotations from the (U)HPLC-MS/MS & DI-HRMS(/MS$^n$) data sets.

**Formatted:** Not Highlight

**Formatted:** Not Highlight

**Formatted:** Not Highlight

**Formatted:** Not Highlight

**Formatted:** Not Highlight

**Formatted:** Not Highlight

**Formatted:** Not Highlight

**Formatted:** Not Highlight

**Formatted:** Not Highlight

**Formatted:** Font: 9 pt, Font color: Auto

**Formatted:** Centered, Space Before: 0 pt, After: 0 pt

**Formatted:** Font: 9 pt, Do not check spelling or grammar, All caps

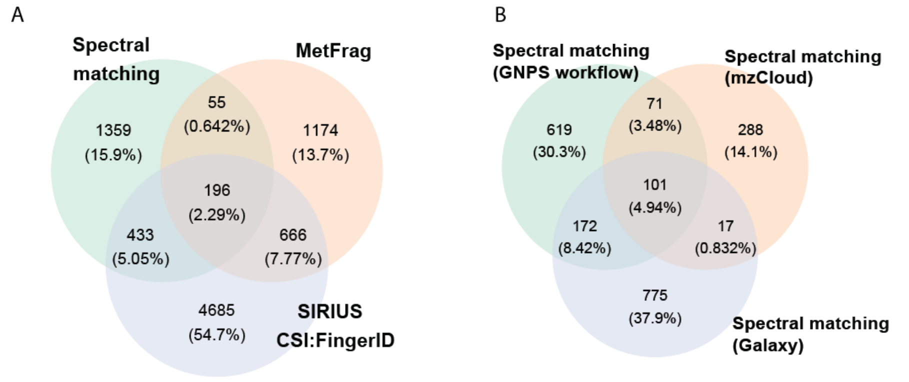


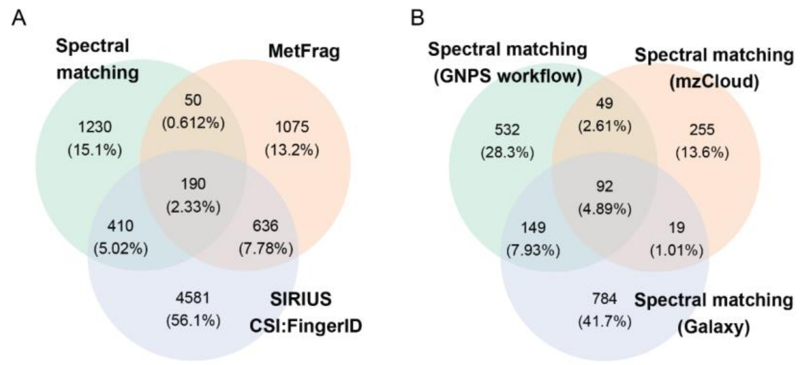


***Figure S28: Venn diagram of metabolite annotations observed across computational annotation approach used.*** *A) Comparison of spectral matching, MetFrag and SIRIUS CSI:FingerID. B) Comparison of the annotations specifically from the three spectral matching approaches used.*

**Formatted:** Font: 9 pt, Font color: Auto

**Formatted:** Centered, Space Before: 0 pt, After: 0 pt

**Formatted:** Font: 9 pt, Do not check spelling or grammar, All caps

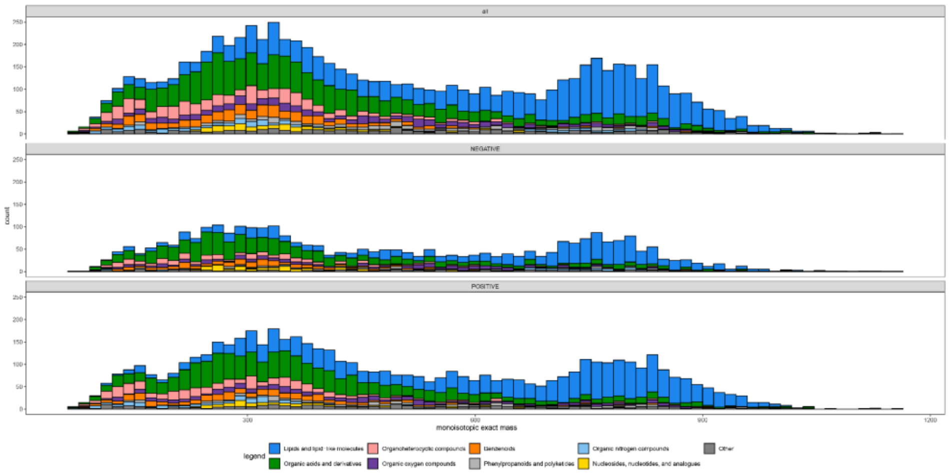


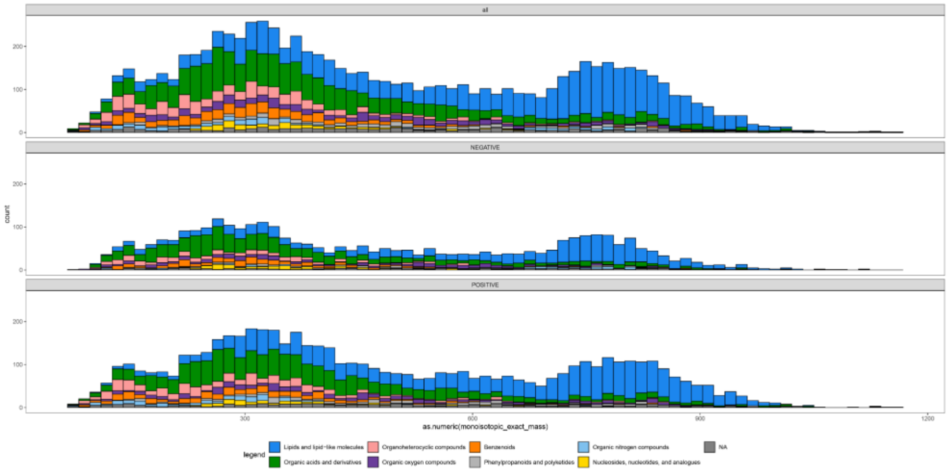


***Figure S29: Distribution of unique metabolite annotations across monoisotopic exact mass:*** *Histograms shown for positive ionisation mode, negative ionisation mode and the combined annotation of positive and negative ionisation mode. Colour represents the superclass compound classification of the annotations.*

**Formatted:** Font: 9 pt, Font color: Auto

**Formatted:** Centered, Space Before: 0 pt, After: 0 pt

**Formatted:** Font: 9 pt, Do not check spelling or grammar, All caps

## 2.4 GC-EI-HRMS derived metabolite annotations

GC-EI-HRMS analysis of the crude polar extract and polar SPE fractions lead to the putative annotation of 31 metabolites (MSI level 2). The GC-EI-HRMS annotations included 19 organic acids and derivatives, including 15 of the 21 proteinogenic amino acids present in eukaryotes (as well as several derivatives thereof); 2 purine nucleotides; 2 pyrimidine nucleotides; 1 glycerophospholipids, 1 steroid (cholesterol), 1 amine (putrescine), 1 carbohydrate (D-glucose), 1 ~~C~~cyclohexanols (myo-inositol); 1 indole derivative (L-tryptophan~~3-Indol-3-ylalanine~~), 1 glycoside (m~~M~~ethyl-galactoside) and 1 inorganic compound (~~S~~sulfuric acid). The 4 organic metabolites unique to the GC-EI-HRMS analysis were: ~~alpha-Oxovaleric acid, L-2-Amino-n-butyric acid, L-2-Amino-n-butyric acid, myo-inositol and Methyl-galactoside.~~ Myo-inositol, 2-oxopentanoic acid, methyl-galactoside and 2-aminobutanoic acid.

**Commented [TL1]:** Updated to use common names rather than chemical names from pubchem/CTS

**Formatted:** Font: 9 pt, Font color: Auto

**Formatted:** Centered, Space Before: 0 pt, After: 0 pt

**Formatted:** Font: 9 pt, Do not check spelling or grammar, All caps

**Table S14:** GC-EI-HRMS derived metabolite annotations

| Compound | Quantifier ion | Retention time | SI/Dot product | SPE fractions |
|---|---|---|---|---|
| 2-aminobutanoic acid | 130.1047 | 7.84 | 772 | WAX[1], WCX[1], Crude |
| 2-oxopentanoic acid | 73.04683 | 10.13 | 714 | WAX[1], WAX[2], WAX[3], WAX[4], WCX[1], Crude |
| Adenosine 5'- | 169.0679 | 17.53 | 757 | WAX[4], WCX[1], Crude |
| Asparagine | 73.04683 | 11.38 | 804 | WAX[1], WCX[1], WCX[4], Crude |
| Cholesterol | 129.073 | 18.13 | 858 | WAX[2], WCX[1], Crude |
| D-glucose | 73.04683 | 12.63 | 852 | WAX[1]. WCX[1], Crude |
| Glycerol-3-phosphate | 299.0713 | 11.87 | 830 | WAX[1], WAX[4], WCX[1], Crude |
| Glycine | 174.113 | 9.01 | 827 | WAX[1], WCX[1], Crude |
| | 102.0735 | 7.33 | 798 | |
| Inosine | 217.1074 | 15.65 | 801 | WAX[1], WCX[1], Crude |
| Lactic acid | 149.0449 | 6.61 | 786 | WAX[1], WAX[2], WAX[3], WCX[1], Crude |
| L-alanine | 116.089 | 7.12 | 849 | WAX[1], WCX[1], Crude |
| L-cystine | 218.1025 | 14.52 | 718 | WAX[1], WCX[1], Crude |
| L-glutamic acid | 246.1339 | 11.08 | 817 | WAX[1], WAX[3], WCX[1], Crude |
| L-glutamine | 156.0837 | 11.97 | 786 | WAX[1], WCX[1], Crude |
| L-isoleucine | 158.136 | 8.89 | 858 | WAX[1], WCX[1], Crude |
| L-leucine | 158.1359 | 8.72 | 735 | WAX[1], WCX[1], Crude |
| L-threonine | 73.04683 | 9.57 | 883 | WAX[1], WCX[1], Crude |
| L-tryptophan | 79.05426 | 14.2 | 762 | WAX[1], WCX[1], WCX[2], Crude |
| L-tyrosine | 218.1026 | 12.86 | 857 | WAX[1], WCX[1], Crude |
| L-valine | 144.1203 | 8.24 | 869 | WAX[1], WCX[1], Crude |
| Lysine | 174.113 | 12.77 | 723 | WAX[1], WCX[1], WCX[3], Crude |
| L-5-oxoproline | 156.0839 | 10.51 | 840 | WAX[1], WAX[3], WCX[1], Crude |
| L-proline | 142.1046 | 8.94 | 858 | WAX[1], WCX[1], Crude |
| Methyl-galactoside | 204.0997 | 12.54 | 744 | WAX[1], WCX[1], Crude |
| Myo-inositol | 217.1074 | 13.62 | 790 | WAX[1], WCX[1], Crude |
| Phenylalanine | 218.1021 | 11.17 | 881 | WAX[1], WCX[1], Crude |
| Putrescine | 174.1129 | 11.41 | 839 | WAX[1], WAX[2], WCX[1], WCX[4], Crude |
| Sulfuric acid | 147.0657 | 7.71 | 796 | WAX[4], WCX[1], Crude |
| Serine | 116.0528 | 8.59 | 726 | WAX[1], WCX[1], Crude |
| | 204.1236 | 9.38 | 851 | |
| Uridine | 73.04683 | 12.27 | 833 | WAX[1], WAX[4], WCX[1], Crude |
| Uridine 5'-monophosphate | 169.0679 | 16.67 | 714 | WAX[4], WCX[1], Crude |

**Formatted:** Font: 9 pt, Font color: Auto

**Formatted:** Centered, Space Before: 0 pt, After: 0 pt

**Formatted:** Font: 9 pt, Do not check spelling or grammar, All caps

## 2.5 NMR derived metabolite annotations

1D and 2D NMR analysis of the crude polar extract and polar SPE fractions lead to the identification of 39 metabolites (MSI level 1) and putative annotation of 2 metabolites. The NMR annotations included 24 organic acids and derivatives; 4 pyrimidine nucleotides; 2 purine nucleosides; 3 carbohydrates; 4 organic nitrogen compounds; 1 imidazopyrimidine (~~6(1H)-Purinone~~hypoxanthine); 1 indole derivative (~~3-Indol-3-ylalanine~~L-tryptophan); 1 glycerophosphocholine (~~sn-3-GPC~~GlyceroPhosphoCholine - GPCho) and 1 benzenoid (~~2,4-bis(azanyl)phenol~~diaminopropane) were observed. The 3 metabolites unique to the NMR analysis were: ~~beta-alanyl-Npi-methyl-L-histidine, Inosinic acid and 2,4-bis(azanyl)phenol.~~ inosinic acid (IMP), anserine and diaminopropane.

**Commented [TL2]:** Updated chemical names to common form rather than the puibchem/cts names

**Table S15:** NMR derived metabolite annotations

| Peak | Metabolites | Group | δ 1H (ppm) | Multiplicity: J (Hz) | Assignment data | Fraction (s) |
|---|---|---|---|---|---|---|
| 1 | L-Alanine | β-$CH_3$ | 1.48 | d | $^1$H, HSQC (18.99), TOCSY (3.79) | WCX1, WAX1 |
| | | α-CH | 3.79 | q | $^1$H, HSQC (53.38), TOCSY (1.48) | |
| 2 | Anserine | β-$CH_2$(Ala) | 2.69 | m | $^1$H, JRES, TOCSY (3.20) | WCX2 |
| | | β-$CH_2$ (His) | 3.05 | dd | $^1$H, JRES, TOCSY (3.20, 4.48, 7.11) | |
| | | α-$CH_2$(Ala) | 3.20 | m | $^1$H, JRES, TOCSY (2.68, 4.48, 7.11) | |
| | | N-$CH_3$ | 3.78 | s | $^1$H, JRES | |
| | | α-CH(His) | 4.48 | dd | $^1$H, JRES, TOCSY (ND*) | |
| | | CH-5(His) | 7.11 | d | $^1$H, JRES, TOCSY (3.05, 3.20, 8.28) | |
| | | CH-2(His) | 8.28 | d | $^1$H, JRES, TOCSY (7.11) | |
| 3 | L-Arginine/Phosphoarginine | γ-$CH_2$ | 1.68 | m | $^1$H, HSQC (26.40), TOCSY (1.92, 3.25) | WCX1, WCX2, WCX3, WCX4 ,WAX1, WAX4 |
| | | β-$CH_2$ | 1.92 | m | $^1$H, HSQC (30.40), TOCSY (1.68, 3.25, 3.76) | |
| | | δ-$CH_2$ | 3.25 | t | $^1$H, HSQC (43.37), TOCSY (1.68, 1.92, 3.76) | |
| | | α-CH | 3.76 | t | $^1$H, HSQC (57.21), TOCSY (1.68, 1.92, 3.25) | |
| 4 | L-Asparagine | β-$CH_2$ | 2.88 | m | $^1$H, HSQC (37.36), TOCSY (2.95, 4.01) | WCX1, WAX1 |
| | | β'-$CH_2$ | 2.95 | m | $^1$H, HSQC (37.43) , TOCSY (2.88, 4.01) | |
| | | α-CH | 4.01 | dd | $^1$H, HSQC (54.16), TOCSY (2.88, 2.95) | |
| 5 | L-Aspartic acid | β-$CH_2$ | 2.69 | dd | $^1$H, TOCSY (2.78, 3.90) | WCX1, WAX3 |
| | | β'-$CH_2$ | 2.78 | dd | $^1$H, TOCSY (2.69, 3.90) | |

**Formatted:** Font: 9 pt, Font color: Auto

**Formatted:** Centered, Space Before: 0 pt, After: 0 pt

**Formatted:** Font: 9 pt, Do not check spelling or grammar, All caps

| Peak | Metabolites | Group | δ 1H (ppm) | Multiplicity: J (Hz) | Assignment data | Fraction (s) |
|---|---|---|---|---|---|---|
| | | α-CH | 3.90 | dd | $^{1}$H, TOCSY (2.69, 2.78) | |
| 6 | Betaine | $CH_3$ | 3.26 | s | $^{1}$H, HSQC (56.09) | WCX1, WAX1 |
| | | $CH_2$ | 3.89 | s | $^{1}$H, HSQC (68.72) | |
| 7 | Choline | $CH_3$ | 3.22 | s | $^{1}$H, HSQC (56.66) | WCX1, WCX4, WAX1 |
| | | β-$CH_2$ | 3.51 | dd | $^{1}$H, HSQC (70.16), TOCSY (4.06) | |
| | | α-$CH_2$ | 4.06 | ddd | $^{1}$H, HSQC (58.52), TOCSY (3.51) | |
| 8 | L-Cystine | $CH_2$ | 3.20 | dd | $^{1}$H, HSQC (40.56), TOCSY (3.39, 4.12) | WCX1, WAX1 |
| | | $CH_2$ | 3.39 | dd | $^{1}$H, HSQC (40.71), TOCSY (3.20, 4.12) | |
| | | CH | 4.12 | dd | $^{1}$H, HSQC (56.19), TOCSY (3.20, 3.39) | |
| 9 | Diaminopropane | β-$CH_2$ | 2.06 | m | $^{1}$H, HSQC (27.86), TOCSY (3.11) | WCX3, WCX4, WAX1 |
| | | α-$CH_2$ | 3.11 | t | $^{1}$H, HSQC (39.59), TOCSY (2.06) | |
| 10 | Fatty acids (FA) | $CH_3$ | 0.90 | t | $^{1}$H, TOCSY (1.30) | WCX1, WCX2, WCX3, WCX4, WAX1, WAX2, WAX3, WAX4 |
| | | (n)$CH_2$ | 1.27 | Broad | $^{1}$H, TOCSY (1.58, 2.25) | |
| | | (1)$CH_2$ | 1.58 | Broad | $^{1}$H, TOCSY (1.27, 2.25) | |
| | | (2)$CH_2$ | 2.25 | Broad | $^{1}$H, TOCSY (1.27, 1.58) | |
| 11 | γ-AminoButyric Acid (GABA) | β-$CH_2$ | 1.89 | m | $^{1}$H, JRES, spiking, TOCSY (2.29, 3.02) | WAX1 |
| | | α-$CH_2$ | 2.29 | t | $^{1}$H, JRES, spiking, TOCSY (1.89, 3.02) | |
| | | γ-$CH_2$ | 3.02 | t | $^{1}$H, JRES, spiking, TOCSY (1.89, 2.29) | |
| 12 | α-Glucose | CH-4 | 3.40 | m | $^{1}$H, HSQC (72.29), TOCSY | WCX1, WAX1 |
| | | CH-2 | 3.54 | dd | $^{1}$H, HSQC(74.26), TOCSY | |
| | | CH-3 | 3.72 | m | $^{1}$H, HSQC (75.7), TOCSY | |
| | | CH-6; CH'-6 | 3.83 | m | $^{1}$H, HSQC (63.45), TOCSY | |
| | | CH-5 | 3.83 | m | $^{1}$H, HSQC (74.13), TOCSY | |
| | | CH-1 | 5.23 | d | $^{1}$H, HSQC (94.80), TOCSY | |
| 13 | β-Glucose | CH-2 | 3.24 | dd | $^{1}$H, HSQC (76.98), TOCSY | WCX1, WAX1 |
| | | CH-4 | 3.40 | m | $^{1}$H, HSQC (72.29), TOCSY | |
| | | CH-3; CH-5 | 3.47 | m | $^{1}$H,HSQC (78.61), TOCSY | |

**Formatted:** Font: 9 pt, Font color: Auto

**Formatted:** Centered, Space Before: 0 pt, After: 0 pt

**Formatted:** Font: 9 pt, Do not check spelling or grammar, All caps

| Peak | Metabolites | Group | δ 1H (ppm) | Multiplicity: J (Hz) | Assignment data | Fraction (s) |
|---|---|---|---|---|---|---|
| | | CH-6 | 3.72 | m | $^{1}$H, HSQC (63.38), TOCSY | |
| | | CH'-6 | 3.89 | dd | $^{1}$H, HSQC (63.42), TOCSY | |
| | | CH-1 | 4.64 | d | $^{1}$H, HSQC (98.64), TOCSY(ND*) | |
| 14 | L-Glutamic acid | β-CH$_2$ | 2.06 | m | $^{1}$H, TOCSY (2.36, 3.77) | WCX1, WAX1, WAX3 |
| | | β'-CH$_2$ | 2.14 | m | $^{1}$H, TOCSY (2.36, 3.77) | |
| | | γ-CH$_2$ | 2.36 | m | $^{1}$H, HSQC (36.12), TOCSY (2.10, 3.77) | |
| | | α-CH | 3.77 | dd | $^{1}$H, HSQC (57.53), TOCSY (2.10, 2.36) | |
| 15 | L-Glutamine | β-CH$_2$ | 2.14 | m | $^{1}$H, HSQC (29.15), TOCSY (2.44, 3.77) | WCX1, WAX1 |
| | | γ-CH$_2$ | 2.44 | m | $^{1}$H, HSQC (33.52), TOCSY (2.14, 3.77) | |
| | | α-CH | 3.77 | t | $^{1}$H, HSQC (57.28), TOCSY (2.14, 2.44) | |
| 16 | Glycerol | CH$_2$ | 3.57 | m | $^{1}$H, HSQC (65.41), TOCSY (3.66, 3.79) | WCX1, WAX1 |
| | | CH$_2$' | 3.66 | m | $^{1}$H, HSQC (65.26), TOCSY (3.57, 3.79) | |
| | | CH | 3.79 | tt | $^{1}$H, HSQC (74.95), TOCSY (3.57, 3.66) | |
| 17 | GlyceroPhosphoCholine (GPCho) | CH$_3$ | 3.22 | s | $^{1}$H, HSQC (56.66), | WCX1, WAX1 |
| | | CH$_2$ | 3.63 | m | $^{1}$H, HSQC (64.63), TOCSY (3.91, 4.33) | |
| | | CH ; CH$_2$ | 3.91 | m | $^{1}$H, HSQC (69.16), TOCSY (3.63) | |
| | | CH$_2$ | 4.33 | m | $^{1}$H, HSQC (62.13), TOCSY (3.63) | |
| 18 | L-Glycine | α-CH$_2$ | 3.56 | s | $^{1}$H, HSQC (44.31) | WCX1, WAX1 |
| 19 | L-Histidine | β-CH$_2$ | 3.18 | dd | $^{1}$H, HSQC (30.24), TOCSY (3.26, 4.01) | WCX1, WAX1 |
| | | β-CH$_2$ | 3.26 | dd | $^{1}$H, HSQC (30.24), TOCSY (3.18, 4.01) | |
| | | α-CH | 4.01 | dd | $^{1}$H, HSQC (57.44), TOCSY (3.18, 3.26, 7.11 ) | |
| | | δ-CH | 7.11 | d | $^{1}$H, HSQC (119.98), TOCSY (4.01) | |
| | | ε-CH | 7.93 | d | $^{1}$H, HSQC (138.41) | |
| 20 | Hypoxanthine | CH | 7.96 | s | $^{1}$H, HSQC (144.51) | WCX1 |
| | | CH | 8.11 | s | $^{1}$H, HSQC (140.15) | |
| 21 | L-Isoleucine | δ-CH$_3$ | 0.93 | t | $^{1}$H, HSQC (13.68), TOCSY(1.01, 1.47, 1.98) | WCX1, WAX1 |
| | | γ-CH$_3$ | 1.01 | d | $^{1}$H, HSQC (17.27), TOCSY(0.93, 1.47, 1.98) | |
| | | γ-CH$_2$ | 1.47 | m | $^{1}$H, HSQC (27.11, TOCSY(0.93, 1.01, 1.98) | |



**Formatted:** Font: 9 pt, Font color: Auto

**Formatted:** Centered, Space Before: 0 pt, After: 0 pt

**Formatted:** Font: 9 pt, Do not check spelling or grammar, All caps

| Peak | Metabolites | Group | δ 1H (ppm) | Multiplicity: J (Hz) | Assignment data | Fraction (s) |
|---|---|---|---|---|---|---|
| | | β-CH | 1.98 | m | $^{1}$H, HSQC (38.68), TOCSY (0.93, 1.01, 1.47) | |
| | | α-CH | 3.68 | d | $^{1}$H, HSQC (62.44), TOCSY(1.01, 1.47, 1.98) | |
| 22 | Inosine | $CH_2$ | 3.84 | dd | $^{1}$H, HSQC (ND), TOCSY(4.26, 4.43) | WCX1, WAX1 |
| | | $CH_2$ | 3.91 | dd | $^{1}$H, HSQC (ND), TOCSY(4.26, 4.43) | |
| | | CH | 4.26 | dd | $^{1}$H, HSQC (88.39), TOCSY(3.91, 4.43, 4.8, 6.06) | |
| | | CH | 4.43 | dd | $^{1}$H, HSQC (73.11), TOCSY(3.91, 4.26, 4.8, 6.06) | |
| | | CH | 4.8* | s | $^{1}$H, HSQC (ND), TOCSY(ND) | |
| | | CH | 6.06 | d | $^{1}$H, HSQC (91.05), TOCSY(4.26, 4.43, 4.8) | |
| | | CH | 8.19 | s | $^{1}$H, HSQC (ND), TOCSY | |
| | | CH | 8.31 | s | $^{1}$H, HSQC (142.94) | |
| 23 | Inosinic acid (IMP) | $CH_2$ | 4.02 | m | $^{1}$H, HSQC (66.35), TOCSY (4.37, 4.51) | WCX1, WAX1, WAX4 |
| | | CH | 4.37 | m | $^{1}$H, HSQC (87.31), TOCSY (4.02, 4.51) | |
| | | CH | 4.51 | m | $^{1}$H, HSQC (73.37), TOCSY (ND*) | |
| | | CH | 6.15 | d | $^{1}$H, HSQC (89.80), TOCSY (4.51) | |
| | | CH | 8.24 | s | $^{1}$H, HSQC (149.20) | |
| | | CH | 8.55 | s | $^{1}$H, HSQC (142.72) | |
| 24 | L-Lactic acid | $CH_3$ | 1.33 | d | $^{1}$H, HSQC (22.98), TOCSY (4.11) | WCX1, WAX1, WAX2, WAX3 |
| | | CH | 4.11 | q | $^{1}$H, HSQC (71.31), TOCSY (1.33) | |
| 25 | L-Leucine | δ-$CH_3$ | 0.95 | t | $^{1}$H, HSQC (23.83), TOCSY (1.71, 3.75) | WCX1, WAX1 |
| | | δ-$CH_3$ | 0.98 | t | $^{1}$H, HSQC (24.77), TOCSY (1.71, 3.75) | |
| | | γ-CH | 1.69 | m | $^{1}$H, HSQC (42.59), TOCSY (0.97, 3.75) | |
| | | β-$CH_2$ | 1.73 | m | $^{1}$H, HSQC (42.59), TOCSY (0.97, 3.75) | |
| | | α-CH | 3.75 | m | $^{1}$H, HSQC (56.34), TOCSY (0.97, 1.71) | |
| 26 | L-Lysine | γ-$CH_2$ | 1.47 | m | $^{1}$H, HSQC(24.15), TOCSY (1.72, 1.91, 3.02, 3.77 ) | WCX1, WCX2, WCX3, WAX1 |
| | | δ-$CH_2$ | 1.72 | m | $^{1}$H, HSQC (29.30), TOCSY (1.47, 1.91, 3.02 ) | |
| | | β-$CH_2$ | 1.91 | m | $^{1}$H, HSQC (32.75), TOCSY (1.47, 1.72, 3.77 ) | |
| | | ε-$CH_2$ | 3.02 | t | $^{1}$H, HSQC (42.12), TOCSY (1.47, 1.72, 1.91) | |
| | | α-CH | 3.77 | t | $^{1}$H, HSQC (57.28), TOCSY (1.47,1.72, 1.91 ) | |

**Formatted:** Font: 9 pt, Font color: Auto

**Formatted:** Centered, Space Before: 0 pt, After: 0 pt

**Formatted:** Font: 9 pt, Do not check spelling or grammar, All caps

| Peak | Metabolites | Group | δ 1H (ppm) | Multiplicity: J (Hz) | Assignment data | Fraction (s) |
|---|---|---|---|---|---|---|
| 27 | L-Methionine | $CH_3$-S | 2.12 | s | $^{1}H$, HSQC (32.76) | WCX1 |
| | | β-$CH_2$ | 2.15 | m | $^{1}H$, TOCSY (2.64, 3.86) | |
| | | δ-$CH_2$ | 2.64 | t | $^{1}H$, HSQC (31.49), TOCSY (2.15, 3.86) | |
| | | α-CH | 3.86 | dd | $^{1}H$, HSQC (56.81), TOCSY (2.64, 2.15) | |
| 28 | L-Ornithine | γ-$CH_2$ | 1.81 | m | $^{1}H$, HSQC (ND), TOCSY (1.93, 3.05, 3.77), spiking | WAX1 |
| | | β-$CH_2$ | 1.93 | m | $^{1}H$, HSQC (30.43), TOCSY (1.81, 3.05, 3.77), spiking | |
| | | δ-$CH_2$ | 3.05 | t | $^{1}H$, HSQC (41.84), TOCSY (1.81, 1.93, 3.77), spiking | |
| | | α-CH | 3.77 | t | $^{1}H$, HSQC (57.28), TOCSY (1.83, 1.93, 3.05), spiking | |
| 29 | L-Phenylalanine | β-$CH_2$ | 3.14 | m | $^{1}H$, HSQC (39.15), TOCSY (4.00 ) | WCX1, WAX1, WAX3 |
| | | α-CH | 4.00 | dd | $^{1}H$, HSQC (58.85), TOCSY (3.14) | |
| | | δ-CH | 7.33 | d | $^{1}H$, HSQC (132.16), TOCSY (7.38, 7.43) | |
| | | ζ-CH | 7.38 | m | $^{1}H$, HSQC (130.44), TOCSY (7.33, 7.43 ) | |
| | | ε-CH | 7.43 | m | $^{1}H$, HSQC (132.00), TOCSY (7.33, 7.38) | |
| 30 | PhosphorylCholine (PCho) | $CH_3$ | 3.22 | s | $^{1}H$, HSQC (39.15) | WCX1, WAX1, WAX4 |
| | | β-$CH_2$ | 3.59 | t | $^{1}H$, HSQC (69.32), TOCSY (4.18 ) | |
| | | α-$CH_2$ | 4.18 | ddd | $^{1}H$, HSQC (60.88), TOCSY (3.59 ) | |
| 31 | Putrescine | β-$CH_2$ | 1.73 | m | $^{1}H$, HSQC (26.80) , TOCSY (3.03) | WCX1, WAX1 |
| | | α-$CH_2$ | 3.03 | t | $^{1}H$, HSQC (41.81) , TOCSY (1.73) | |
| 32 | L-Serine | α-CH | 3.84 | dd | $^{1}H$, HSQC (59.16), TOCSY (3.96) | WCX1, WAX1 |
| | | β-$CH_2$ | 3.96 | m | $^{1}H$, HSQC (63.22), TOCSY (3.84) | |
| 33 | L-Threonine | γ-$CH_3$ | 1.33 | d | $^{1}H$, HSQC (22.27), TOCSY (3.61, 4.25) | WCX1, WAX1 |
| | | α-CH | 3.61 | d | $^{1}H$, HSQC (63.38), TOCSY (1.33, 4.25) | |
| | | β-CH | 4.25 | m | $^{1}H$, HSQC (69.01), TOCSY (1.33, 3.61) | |
| 34 | L-Tryptophan | β-CH | 3.29 | dd | $^{1}H$, HSQC (29.14), TOCSY (3.47, 4.06) | WCX1, WAX1, WAX3 |
| | | β'-CH | 3.47 | dd | $^{1}H$, HSQC (29.14), TOCSY (3.29, 4.06) | |
| | | α-CH | 4.06 | dd | $^{1}H$, HSQC (57.9), TOCSY (3.29, 3.47) | |
| | | ζ'-CH | 7.20 | m | $^{1}H$, HSQC (122.15), TOCSY (7.28, 7.53, 7.73) | |

**Formatted:** Font: 9 pt, Font color: Auto

**Formatted:** Centered, Space Before: 0 pt, After: 0 pt

**Formatted:** Font: 9 pt, Do not check spelling or grammar, All caps

| Peak | Metabolites | Group | δ 1H (ppm) | Multiplicity: J (Hz) | Assignment data | Fraction (s) |
|---|---|---|---|---|---|---|
| | | ε-CH | 7.28 | m | 1H, HSQC (124.81), TOCSY (7.20, 7.53, 7.73) | |
| | | δ-CH | 7.33 | s | 1H, HSQC (128.09), TOCSY (2.28, 3.62) | |
| | | ζ-CH | 7.53 | d | 1H, HSQC (114.81), TOCSY (7.20, 7.28, 7.73) | |
| | | η-CH | 7.73 | d | 1H, HSQC (121.22), TOCSY (7.20, 7.28, 7.53) | |
| 35 | L-Tyrosine | β-$CH_2$ | 3.05 | dd | 1H, HSQC (38.37), TOCSY(3.17, 3.94) | WCX1, WAX1, WAX3 |
| | | β'-$CH_2$ | 3.17 | dd | 1H, HSQC (38.37), TOCSY (3.05, 3.94) | |
| | | α-CH | 3.94 | dd | 1H, HSQC (59.00), TOCSY (3.05, 3.17) | |
| | | ε-CH | 6.89 | m | 1H, HSQC (118.72), TOCSY (7.19) | |
| | | δ-CH | 7.19 | m | 1H, HSQC (133.57), TOCSY (6.89) | |
| 36 | Uridine | CH2 | 3.81 | dd | 1H, JRES, TOCSY (3.91, 4.13, 4.23) | WCX1, WAX1 |
| | | CH2’ | 3.91 | dd | 1H, JRES, TOCSY (3.81, 4.13, 4.23) | |
| | | CH | 4.13 | m | 1H, JRES, TOCSY (3.81, 3.91, 4.23) | |
| | | CH | 4.23 | dd | 1H, JRES, TOCSY (3.81, 3.91, 4.13, 5.9) | |
| | | CH | 4.35 | dd | 1H, JRES, TOCSY (4.13, 4.23, 5.9) | |
| | | CH | 5.89 | d | 1H, JRES, TOCSY (4.13, 4.23, 4.35, 7.97) | |
| | | CH | 5.91 | d | 1H, JRES, TOCSY (4.13, 4.23, 4.35, 7.97) | |
| | | CH | 7.97 | d | 1H, JRES, TOCSY (5.90) | |
| 37 | Uridine MonoPhosphate (UMP) | $CH_2$ | 3.97 | m | 1H, HSQC (ND), TOCSY (4.25, 4.33, 4.39) | WCX1, WAX1, WAX4 |
| | | CH | 4.25 | m | 1H, HSQC (ND), TOCSY (3.97, 4.33, 4.39, 5.98) | |
| | | CH | 4.33 | t | 1H, HSQC (72.60), TOCSY (3.97, 4.25, 4.39, 5.98) | |
| | | CH | 4.39 | t | 1H, HSQC (76.67), TOCSY (3.97, 4.25, 4.33, 5.98) | |
| | | CH | 5.97 | m | 1H, HSQC (105.43), TOCSY (4.25, 4.33, 4.39) | |
| | | CH | 5.98 | m | 1H, HSQC (91.20), TOCSY (4.25, 4.33, 4.39) | |
| | | CH | 8.08 | d | 1H, HSQC (ND), TOCSY (5.98) | |
| 38 | Uridine DiPhosphate (UDP) | $CH_2$ | 4.21 | dd | 1H, HSQC (ND), TOCSY (4.27, 4.38, 4.42) | WCX1, WAX4 |
| | | CH | 4.27 | m | 1H, HSQC (ND), TOCSY (4.21, 4.38, 4.42) | |
| | | CH | 4.38 | t | 1H, HSQC (ND), TOCSY (4.21, 4.27, 4.42) | |
| | | CH | 4.42 | t | 1H, HSQC (91.39), TOCSY (4.21, 4.27, 4.38) | |

Formatted: Font: 9 pt, Font color: Auto

Formatted: Centered, Space Before: 0 pt, After: 0 pt

Formatted: Font: 9 pt, Do not check spelling or grammar, All caps

| Peak | Metabolites | Group | δ 1H (ppm) | Multiplicity: J (Hz) | Assignment data | Fraction (s) |
|---|---|---|---|---|---|---|
| | | CH | 5.96 | s | $^1$H, HSQC (ND) | |
| | | CH | 5.97 | d | $^1$H, HSQC (105.32), TOCSY ( 7.98) | |
| | | CH | 7.98 | d | $^1$H, HSQC (ND), TOCSY (5.96, 5.97) | |
| 39 | UDP-N-acetylglucosamine | CH3 | 2.07 | s | $^1$H, HSQC (ND), JRES | WCX1 |
| | | CH | 3.56 | dd | $^1$H, HSQC (ND), TOCSY (3.80, 3.87, 3.92, 4.00) | |
| | | CH | 3.80 | m | $^1$H, HSQC (ND), TOCSY (3.56, 3.87,3.92, 4.00) | |
| | | CH2 | 3.87 | dd | $^1$H, HSQC (ND), TOCSY (3.56, 3.80, 3.92) | |
| | | CH | 3.92 | m | $^1$H, HSQC (ND), TOCSY (3.56, 3.80, 3.87, 4.00) | |
| | | CH | 4.00 | ddd | $^1$H, HSQC (56.03), TOCSY (3.56, 3.8, 3.92, 5.51) | |
| | | CH2 | 4.19 | m | $^1$H, HSQC (67.76), TOCSY (4.37, 5.96) | |
| | | CH2’ | 4.24 | m | $^1$H, HSQC (67.76), TOCSY (4.37, 5.96) | |
| | | CH | 4.29 | m | $^1$H, HSQC (85.89), TOCSY (4.26, 5.96) | |
| | | CH | 4.37 | m | $^1$H, HSQC (76.67), TOCSY (4.26, 5.96) | |
| | | CH | 5.51 | dd | $^1$H, HSQC (97.30), TOCSY (3.56, 3.80, 4.00) | |
| | | CH | 5.95 | d | $^1$H, HSQC (105.43), TOCSY (4.37, 7.94) | |
| | | CH | 5.97 | d | $^1$H, HSQC (91.20), TOCSY (4.37, 7.94) | |
| | | CH | 7.94 | d | $^1$H, HSQC (ND), TOCSY (5.96) | |
| 40 | L-Valine | γ-$CH_3$ | 0.99 | d | $^1$H, HSQC (19.45), TOCSY (2.28, 3.62) | WCX1, WAX1 |
| | | γ'-$CH_3$ | 1.04 | d | $^1$H, HSQC (20.70), TOCSY (2.28, 3.62) | |
| | | β-CH | 2.28 | m | $^1$H, HSQC (31.96), TOCSY (1.02, 3.62) | |
| | | α-CH | 3.62 | d | $^1$H, HSQC (63.38), TOCSY (1.02, 2.28) | |

*: Not detected because of the water suppression

**Formatted:** Font: 9 pt, Font color: Auto

**Formatted:** Centered, Space Before: 0 pt, After: 0 pt

**Formatted:** Font: 9 pt, Do not check spelling or grammar, All caps

## 2.6 Assessment of the computational and experimental DMA workflow with metabolite reference standards

Metabolite reference standards were analysed using the DMA workflow to check that they were separated and annotated correctly – see **Figures S30-31**. The reference standards covered a wide biochemical space including lipid and lipid-like molecules; organic acids and derivatives; organic oxygen compounds; organoheterocyclic compounds; nucleosides, nucleotides, and analogues; and organic nitrogen compounds.

Forty-three of the 48 (89.6%) reference standards were successfully annotated as top ranked. For the apolar components of the workflow, the majority of annotations were for lipid and lipid-like molecules, while for the polar component of the workflow the chemical space was more diverse, with lipid and lipid-like molecules, organic acids and derivatives, alongside other superclasses and classes of compounds observed. The five missing standards, when only using the top ranked metabolite annotations, were: Adenosine triphosphate; pyruvate; 1,2-dioctadecanoyl-sn-glycero-3-phospho-L-serine (PS 18:0/18:0); Nicotinamide adenine dinucleotide phosphate; and spermine.

Our observations indicate that the majority of the missing metabolites were phosphorylated. Enhancements to the workflow could potentially increase their coverage, as recent advancements in liquid chromatography (LC) hardware and column technology offer significant improvements for the analysis of phosphorylated compounds and other compounds that interact with metal surfaces in the LC flow path (DeLano et al., 2021; Gilar et al., 2021; McCalley, 2022).

Spectral matching was able to annotate 39 out of the 48 (81.3 %) reference standard compounds. SIRIUS CSI:FingerID was able to annotate 39 out of the 48 (81.3%) and MetFrag was able to annotate 25 out of the 48 (52.1). The lower percentage of annotation from MetFrag is in part due to the high threshold (>0.95 MetFrag weighted score) used for the final filtering of the MetFrag annotations.

See **Supplemental Table S11** (provided in separate excel file) for a summary of the reference standard annotations.

**Formatted:** Font: 9 pt, Font color: Auto

**Formatted:** Centered, Space Before: 0 pt, After: 0 pt

**Formatted:** Font: 9 pt, Do not check spelling or grammar, All caps

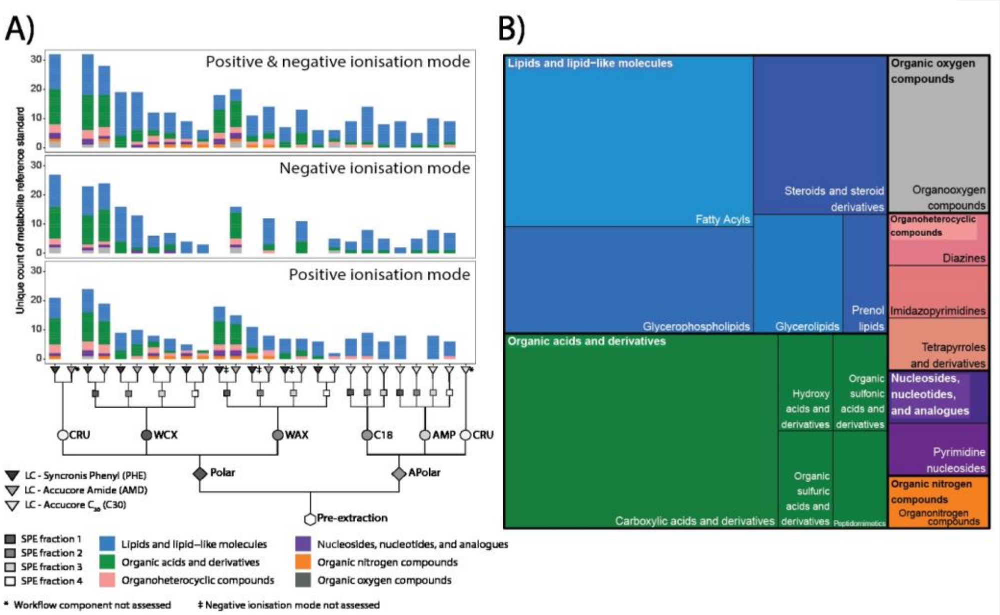


***Figure S30: Assessment of the DMA experimental and computational workflow.*** ***A)*** *Count of matched reference standards across the different components of the experimental workflow confirming that, for example, organic acids and derivatives were detected in the polar arm while the apolar arm predominantly detected lipids and lipid-like molecules.* ***B)*** *Treemap of metabolite standard compound classes and superclasses.*

Formatted: Font: 9 pt, Font color: Auto

Formatted: Centered, Space Before: 0 pt, After: 0 pt

Formatted: Font: 9 pt, Do not check spelling or grammar, All caps

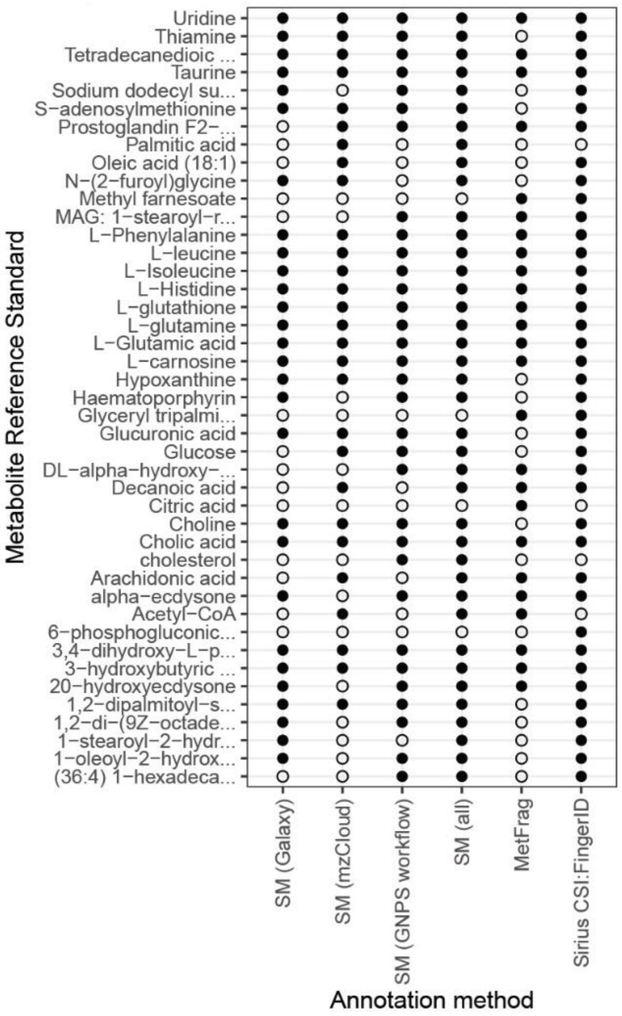


***Figure S31: Summary of which annotation approach was able to identify each metabolite standard.*** *Black circles represent a successful annotation to the standard and white/empty circles represent where the annotation was unsuccessful. SM = spectral matching; SM (all) = a successful annotation using any of the spectral matching approaches.*

**Formatted:** Font: 9 pt, Font color: Auto

**Formatted:** Centered, Space Before: 0 pt, After: 0 pt

**Formatted:** Font: 9 pt, Do not check spelling or grammar, All caps

## 2.7 Pathway analysis

**Formatted:** Font: 9 pt, Font color: Auto

**Formatted:** Centered, Space Before: 0 pt, After: 0 pt

**Formatted:** Font: 9 pt, Do not check spelling or grammar, All caps

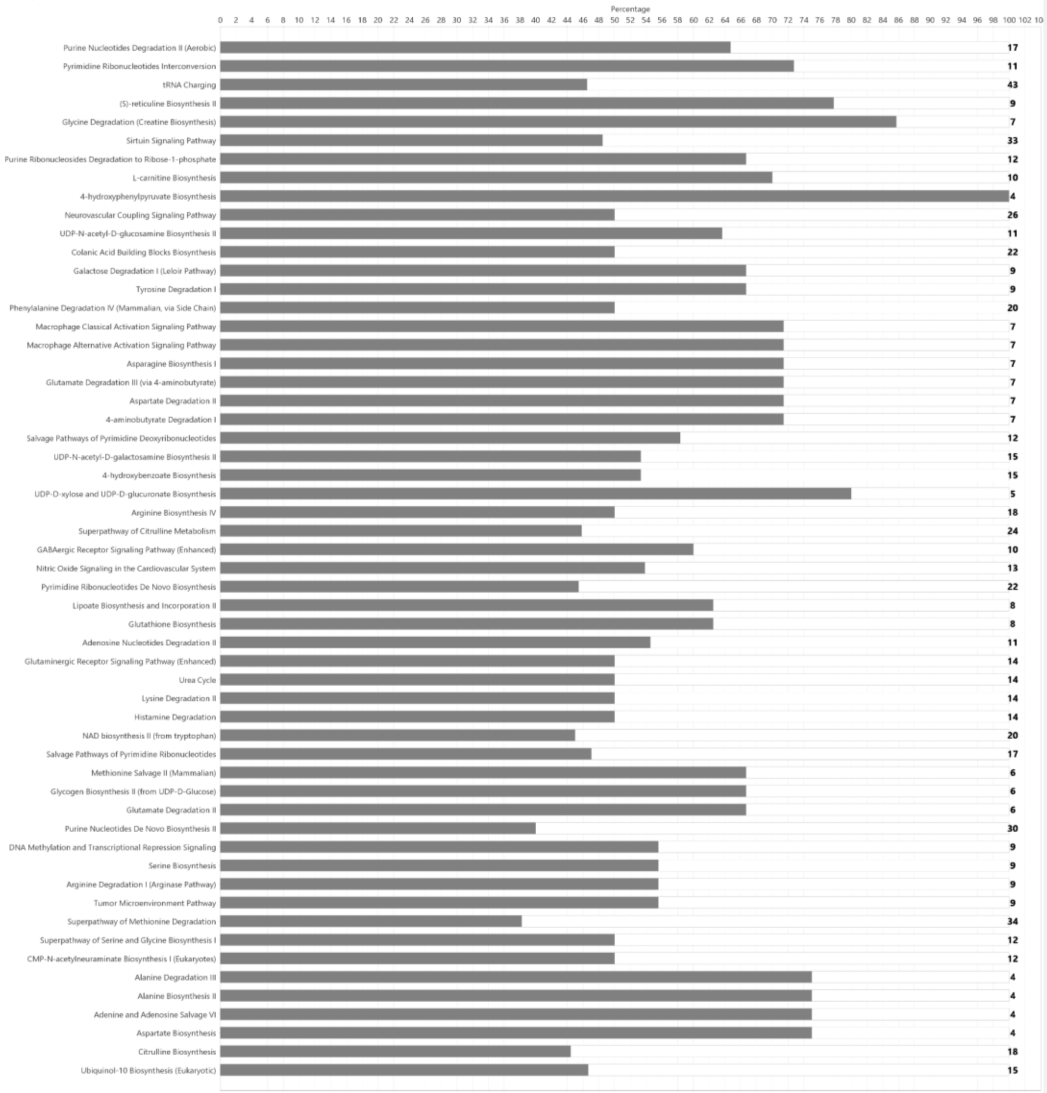


**Formatted:** Font: 9 pt, Font color: Auto

**Formatted:** Centered, Space Before: 0 pt, After: 0 pt

**Formatted:** Font: 9 pt, Do not check spelling or grammar, All caps

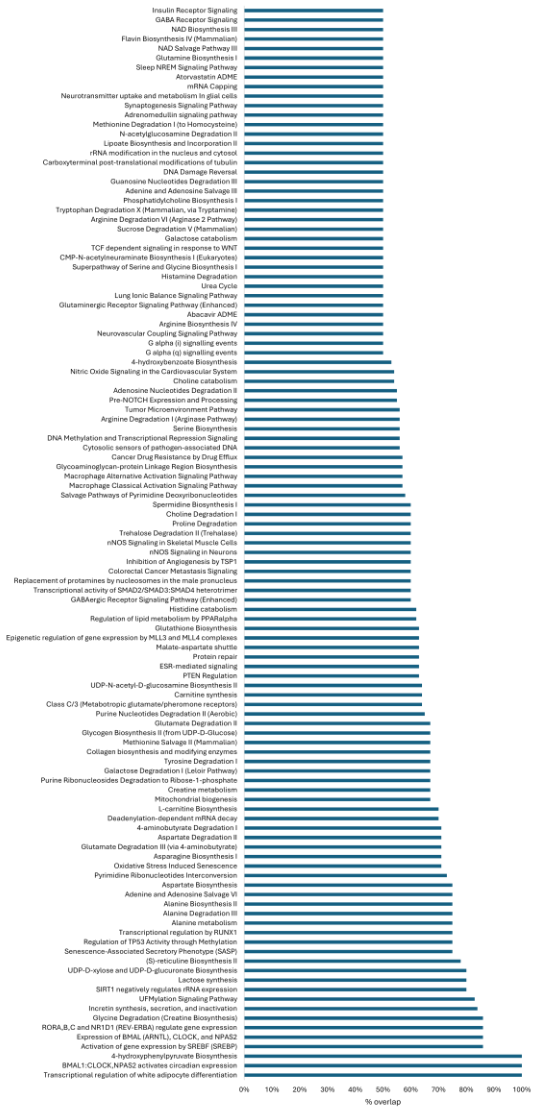


***Figure S32: Summary of the top canonical pathways derived using QIAGEN Ingenuity Pathway Analysis (IPA) for all annotations obtained from the DMA of D. magna.*** *Pathway coverage is shown as a percentage at the top and the numbers on the right represent metabolites in the particular pathway. Only showing pathways >=3 metabolites (measured) and >=50% coverage.~~with <= 0.05 p-value from Fisher's exact test where the background is derived from the IPA database.~~*

**Formatted:** Font: 9 pt, Font color: Auto

**Formatted:** Centered, Space Before: 0 pt, After: 0 pt

**Formatted:** Font: 9 pt, Do not check spelling or grammar, All caps

## 2.8 Molecular network analysis using GNPS

Spectral networks generated using the GNPS network analysis workflow were performed across all of the *D. magna* data files separated into positive and negative ionisation data.

For the positive ionisation mode the network contains 616,499 spectra of which 78,353 of the spectra are annotated using the GNPS spectral matching libraries. The GNPS workflow determined 31,323 distinct clusters of the network (including singlets) of which 1,052 had some level of annotation to GNPS spectral matching libraries. MS2LDA analysis on the positive ionisation network determined 47,853 mass motifs.

For the negative ionisation mode the network contains 165,430 spectra of which 17,666 of the spectra are annotated using the GNPS spectral matching libraries. The GNPS workflow determined 5,320 distinct clusters of the network (including singlets) of which 187 had some level of annotation to GNPS spectral matching libraries. MS2LDA analysis on the negative ionisation network determined 8,681 mass motifs.

**Formatted:** Font: 9 pt, Font color: Auto
**Formatted:** Centered, Space Before: 0 pt, After: 0 pt
**Formatted:** Font: 9 pt, Do not check spelling or grammar, All caps

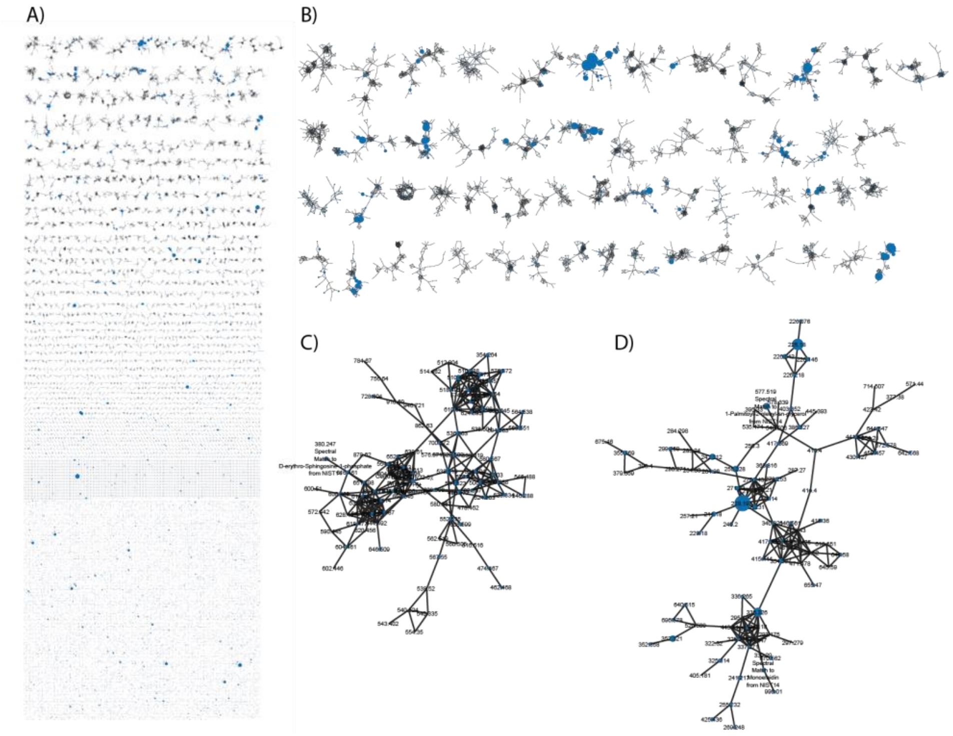


**Figure S33: GNPS spectral network analysis (positive ionisation mode).** A) Overview of positive ionisation mode spectral networks generated from GNPS molecular network analysis showing all 31,323 distinct clusters. B) the top 55 clusters (based on how many nodes were observed). C) and D) show the top 2 clusters (again based on how many nodes were observed) which further display the precursor m/z associated with the node and the compound name of any spectral annotation derived from spectral matching within GNPS. For A), the largest cluster observed, two spectral matching annotations were observed for D-erythro-Sphingosine-1-phosphate and Cer(d18:1/22:0). For B), the second largest cluster observed, two spectral matching annotations for Monoelaidin and 1-Palmitoyl-2-oleoyl-sn-glycerol.

**Formatted:** Font: 9 pt, Font color: Auto

**Formatted:** Centered, Space Before: 0 pt, After: 0 pt

**Formatted:** Font: 9 pt, Do not check spelling or grammar, All caps

# 3 References


Baer, K.N., Goulden, C.E., 1998. Evaluation of a high-hardness COMBO medium and frozen algae for Daphnia magna. Ecotoxicology and environmental safety 39, 201–206. https://doi.org/10.1006/eesa.1997.1627

Bligh, E.G., Dyer, W.J., 1959. A rapid method of total lipid extraction and purification. Canadian Journal of Biochemistry and Physiology 37, 911–917.

Chao, A., Al-Ghoul, H., McEachran, A.D., Balabin, I., Transue, T., Cathey, T., Grossman, J.N., Singh, R.R., Ulrich, E.M., Williams, A.J., Sobus, J.R., 2020. In silico MS/MS spectra for identifying unknowns: a critical examination using CFM-ID algorithms and ENTACT mixture samples. Anal Bioanal Chem 412, 1303–1315. https://doi.org/10.1007/s00216-019-02351-7

Chauve, B., Guillarme, D., Cléon, P., Veuthey, J.-L., 2010. Evaluation of various HILIC materials for the fast separation of polar compounds. Journal of separation science 33, 752–764.

Davis, A.L., Laue, E.D., Keeler, J., Moskau, D., Lohman, J., 1991. Absorption-mode two-dimensional NMR spectra recorded using pulsed field gradients. Journal of Magnetic Resonance (1969) 94, 637–644. https://doi.org/10.1016/0022-2364(91)90154-L

DeLano, M., Walter, T.H., Lauber, M.A., Gilar, M., Jung, M.C., Nguyen, J.M., Boissel, C., Patel, A.V., Bates-Harrison, A., Wyndham, K.D., 2021. Using Hybrid Organic–Inorganic Surface Technology to Mitigate Analyte Interactions with Metal Surfaces in UHPLC. Anal. Chem. 93, 5773–5781. https://doi.org/10.1021/acs.analchem.0c05203

Djoumbou Feunang, Y., Eisner, R., Knox, C., Chepelev, L., Hastings, J., Owen, G., Fahy, E., Steinbeck, C., Subramanian, S., Bolton, E., Greiner, R., Wishart, D.S., 2016. ClassyFire: automated chemical classification with a comprehensive, computable taxonomy. J Cheminform 8, 61. https://doi.org/10.1186/s13321-016-0174-y

Dührkop, K., Fleischauer, M., Ludwig, M., Aksenov, A.A., Melnik, A.V., Meusel, M., Dorrestein, P.C., Rousu, J., Böcker, S., 2019. SIRIUS 4: a rapid tool for turning tandem mass spectra into metabolite structure information. Nat Methods 16, 299–302. https://doi.org/10.1038/s41592-019-0344-8

Garreta-Lara, E., Campos, B., Barata, C., Lacorte, S., Tauler, R., 2016. Metabolic profiling of Daphnia magna exposed to environmental stressors by GC–MS and chemometric tools. Metabolomics 12, 86. https://doi.org/10.1007/s11306-016-1021-x

Giacomoni, F., Le Corguille, G., Monsoor, M., Landi, M., Pericard, P., Petera, M., Duperier, C., Tremblay-Franco, M., Martin, J.-F., Jacob, D., Goulitquer, S., Thevenot, E.A., Caron, C., 2015. Workflow4Metabolomics: a collaborative research infrastructure for computational metabolomics. Bioinformatics 31, 1493–1495. https://doi.org/10.1093/bioinformatics/btu813

Gilar, M., DeLano, M., Gritti, F., 2021. Mitigation of analyte loss on metal surfaces in liquid chromatography. Journal of Chromatography A 1650, 462247. https://doi.org/10.1016/j.chroma.2021.462247

Heaton, J.C., McCalley, D.V., 2016. Some factors that can lead to poor peak shape in hydrophilic interaction chromatography, and possibilities for their remediation. Journal of Chromatography A 1427, 37–44. https://doi.org/10.1016/j.chroma.2015.10.056


**Formatted:** Font: 9 pt, Font color: Auto

**Formatted:** Centered, Space Before: 0 pt, After: 0 pt

**Formatted:** Font: 9 pt, Do not check spelling or grammar, All caps

Heller, S.R., McNaught, A.D., 2009. The IUPAC International Chemical Identifier (InChI). Chemistry International 31, 7–9. https://doi.org/10.1515/ci.2009.31.1.7

Hoch, J.C., Baskaran, K., Burr, H., Chin, J., Eghbalnia, H.R., Fujiwara, T., Gryk, M.R., Iwata, T., Kojima, C., Kurisu, G., Maziuk, D., Miyanoiri, Y., Wedell, J.R., Wilburn, C., Yao, H., Yokochi, M., 2022. Biological Magnetic Resonance Data Bank. Nucleic Acids Research 51, D368–D376. https://doi.org/10.1093/nar/gkac1050

Johnson, J.R., Karlsson, D., Dalene, M., Skarping, G., 2010. Determination of aromatic amines in aqueous extracts of polyurethane foam using hydrophilic interaction liquid chromatography and mass spectrometry. Analytica Chimica Acta 678, 117–123. https://doi.org/10.1016/j.aca.2010.08.020

Kikuchi, J., Tsuboi, Y., Komatsu, K., Gomi, M., Chikayama, E., Date, Y., 2016. SpinCouple: Development of a Web Tool for Analyzing Metabolite Mixtures via Two-Dimensional J-Resolved NMR Database. Anal Chem 88, 659–665. https://doi.org/10.1021/acs.analchem.5b02311

Kuhl, C., Tautenhahn, R., Böttcher, C., Larson, T.R.R., Neumann, S., Bo, C., Larson, T.R.R., Neumann, S., 2012. CAMERA: An integrated strategy for compound spectra extraction and annotation of liquid chromatography/mass spectrometry data sets. Anal. Chem. 84, 283–289. https://doi.org/10.1021/ac202450g

Lawson, T.N., Weber, R.J.M., Jones, M.R., Chetwynd, A.J., Rodriguez Blanco, G.A., Di Guida, R., Viant, M.R., Dunn, W.B., 2017. msPurity: Automated evaluation of precursor ion purity for mass spectrometry based fragmentation in metabolomics. Anal. Chem. 89, acs.analchem.6b04358. https://doi.org/10.1021/acs.analchem.6b04358

Libiseller, G., Dvorzak, M., Kleb, U., Gander, E., Eisenberg, T., Madeo, F., Neumann, S., Trausinger, G., Sinner, F., Pieber, T., others, 2015. IPO: a tool for automated optimization of XCMS parameters. BMC bioinformatics 16, 118.

Ludwig, C., Easton, J.M., Lodi, A., Tiziani, S., Manzoor, S.E., Southam, A.D., Byrne, J.J., Bishop, L.M., He, S., Arvanitis, T.N., Günther, U.L., Viant, M.R., 2012. Birmingham Metabolite Library: a publicly accessible database of 1-D 1H and 2-D 1H J-resolved NMR spectra of authentic metabolite standards (BML-NMR). Metabolomics 8, 8–18. https://doi.org/10.1007/s11306-011-0347-7

McCalley, D.V., 2022. Influence of metals in the column or instrument on performance in hydrophilic interaction liquid chromatography. Journal of Chromatography A 1663, 462751. https://doi.org/10.1016/j.chroma.2021.462751

Neumann, S., Thum, A., Böttcher, C., 2013. Nearline acquisition and processing of liquid chromatography-tandem mass spectrometry data. Metabolomics 9, 84–91. https://doi.org/10.1007/s11306-012-0401-0

Orekhov, V.Y., Jaravine, V.A., 2011. Analysis of non-uniformly sampled spectra with multi-dimensional decomposition. Progress in nuclear magnetic resonance spectroscopy 59, 271–292. https://doi.org/10.1016/j.pnmrs.2011.02.002

Ruta, J., Rudaz, S., McCalley, D.V., Veuthey, J.-L., Guillarme, D., 2010. A systematic investigation of the effect of sample diluent on peak shape in hydrophilic interaction liquid chromatography. Journal of Chromatography A 1217, 8230–8240. https://doi.org/10.1016/j.chroma.2010.10.106

**Formatted:** Font: 9 pt, Font color: Auto

**Formatted:** Centered, Space Before:  0 pt, After:  0 pt

**Formatted:** Font: 9 pt, Do not check spelling or grammar, All caps

Ruttkies, C., Neumann, S., Posch, S., 2019. Improving MetFrag with statistical learning of fragment annotations. BMC Bioinformatics 20, 376. https://doi.org/10.1186/s12859-019-2954-7

Ruttkies, C., Schymanski, E.L., Wolf, S., Hollender, J., Neumann, S., 2016. MetFrag relaunched: Incorporating strategies beyond in silico fragmentation. J Cheminform 8, 1–16. https://doi.org/10.1186/s13321-016-0115-9

Shaka, A., Lee, C., Pines, A., 1988. Iterative schemes for bilinear operators; application to spin decoupling. Journal of Magnetic Resonance (1969) 77, 274–293.

Smith, C.A., Want, E.J., Maille, G.O., Abagyan, R., Siuzdak, G., 2006. XCMS : Processing Mass Spectrometry Data for Metabolite Profiling Using Nonlinear Peak Alignment , Matching , and Identification. Anal. Chem. 78, 779–787. https://doi.org/10.1021/ac051437y

Southam, A.D., Weber, R.J.M., Engel, J., Jones, M.R., Viant, M.R., 2017. A complete workflow for high-resolution spectral-stitching nanoelectrospray direct-infusion mass-spectrometry-based metabolomics and lipidomics. Nature Protocols 12, 310–328. https://doi.org/10.1038/nprot.2016.156

Vorkas, P.A., Isaac, G., Anwar, M.A., Davies, A.H., Want, E.J., Nicholson, J.K., Holmes, E., 2015. Untargeted UPLC-MS Profiling Pipeline to Expand Tissue Metabolome Coverage: Application to Cardiovascular Disease. Anal. Chem. 87, 4184–4193. https://doi.org/10.1021/ac503775m

Wishart, D.S., Tzur, D., Knox, C., Eisner, R., Guo, A.C., Young, N., Cheng, D., Jewell, K., Arndt, D., Sawhney, S., Fung, C., Nikolai, L., Lewis, M., Coutouly, M.-A., Forsythe, I., Tang, P., Shrivastava, S., Jeroncic, K., Stothard, P., Amegbey, G., Block, D., Hau, D.D., Wagner, J., Miniaci, J., Clements, M., Gebremedhin, M., Guo, N., Zhang, Y., Duggan, G.E., Macinnis, G.D., Weljie, A.M., Dowlatabadi, R., Bamforth, F., Clive, D., Greiner, R., Li, L., Marrie, T., Sykes, B.D., Vogel, H.J., Querengesser, L., 2007. HMDB: the Human Metabolome Database. Nucleic acids research 35, D521--6. https://doi.org/10.1093/nar/gkl923

Wolf, S., Schmidt, S., Müller-Hannemann, M., Neumann, S., 2010. In silico fragmentation for computer assisted identification of metabolite mass spectra. BMC Bioinformatics 11, 148. https://doi.org/10.1186/1471-2105-11-148

**Formatted:** Font: 9 pt, Font color: Auto

**Formatted:** Centered, Space Before: 0 pt, After: 0 pt

**Formatted:** Font: 9 pt, Do not check spelling or grammar, All caps
