# Supplementary material for: Experimental and computational approaches for deep metabolome annotation with application to the ecotoxicological model organism Daphnia magna
Source: Gigascience. 2026 May 9;15:giag055. doi: 10.1093/gigascience/giag055 (PMC13254479; doi:10.1093/gigascience/giag055)
Supplement: giag055_Reviewer_1_Report_original_submission — Reviewer 1 -- 11/21/2025 [file giag055_reviewer_1_report_original_submission.pdf]

Reviewer Report

Title: Experimental and computational approaches for deep metabolome annotation with application to the ecotoxicological model organism Daphnia magna

Version: Original Submission **Date:** 11/21/2025

Reviewer name: Reviewer 1

Reviewer Comments to Author:

Jones et al describes a deep metabolome annotation of Daphnia magna. The array of analytical chemistry analyses is impressive. The reported annotation is of very high coverage. This is clearly a high-value resource for metabolomics and for environmental toxicology.As a resource, it will be more useful if feature annotation from each method is made accessible. That is, when another lab generates data using a similar method, the m/z and retention time can be used for their annotation. As is, the supplemental tables and the website only provide compound centered results, which are not clearly linked to experimental data. Annotation per experiment method will also help others assess annotation confidence and how to best combine annotations, which are, as the authors rightfully acknowledged, not fully evaluated. Concentration levels, even just signal intensity as proxy, would be very useful to others. The project website hosts a searchable table, but direct dataset downloads should be available and important.It will also be important to archive the high-quality features without annotation. As the authors have invested so much in generating the data, listing the unknown features will make it possible for them to become known in the future.It is understood that readers can reproduce such features and annotation by running the various Galaxy histories. But this reproducibility requires a high level of commitment to access a Galaxy server and look up many granular details, where the underlying software functions have many design limitations. This makes reproducibility for the sake of reproducibility, not necessarily for scientific progress. We appreciate the efforts from the authors, but wish that the paper includes some discussion of the older software tools versus newer options.The IPA pathway analysis appears to be incorrect, because Fisher's exact test is for overrepresentation of a category/pathway. This work concerns how the annotated metabolites map to metabolic pathways, not involving an overrepresentation test. For this, one can use a generic pathway definition from an open resource. IPA is not needed but adds complications.The website, https://dmadb.bham.ac.uk/, is nice. But we have spotted a few glitches. For some functions, errors returned that user privilege is not granted. Filtering worked for some values, not others. E.g. Error 502 Gateway Error when 390-399 was applied to mono mass filtering.It'd be nice to mention in the main text of how the NMR data overlap with MS data.Under section 3.4, reference to Figure 7 should be Figure 6.The stress conditions in this paper were rather limited. It'd be good to add to discussions how this may be broadened in the future. How to link to genomics would be worthy discussions too.

Level of Interest

Please indicate how interesting you found the manuscript: Choose an item.

Quality of Written English

Please indicate the quality of language in the manuscript: Choose an item.

Declaration of Competing Interests

Please complete a declaration of competing interests, considering the following questions:

- Have you in the past five years received reimbursements, fees, funding, or salary from an organisation that may in any way gain or lose financially from the publication of this manuscript, either now or in the future?
- Do you hold any stocks or shares in an organisation that may in any way gain or lose financially from the publication of this manuscript, either now or in the future?
- Do you hold or are you currently applying for any patents relating to the content of the manuscript?
- Have you received reimbursements, fees, funding, or salary from an organization that holds or has applied for patents relating to the content of the manuscript?
- Do you have any other financial competing interests?
- Do you have any non-financial competing interests in relation to this paper?

If you can answer no to all of the above, write 'I declare that I have no competing interests' below. If your reply is yes to any, please give details below.

I declare that I have no competing interests.

I agree to the open peer review policy of the journal. I understand that my name will be included on my report to the authors and, if the manuscript is accepted for publication, my named report including any attachments I upload will be posted on the website along with the authors' responses. I agree for my report to be made available under an Open Access Creative Commons CC-BY license (http://creativecommons.org/licenses/by/4.0/). I understand that any comments which I do not wish to be included in my named report can be included as confidential comments to the editors, which will not be published.

Choose an item.

To further support our reviewers, we have joined with Publons, where you can gain additional credit to further highlight your hard work (see: https://publons.com/journal/530/gigascience). On publication of this paper, your review will be automatically added to Publons, you can then choose whether or not to claim your Publons credit. I understand this statement.

Yes Choose an item.
