# Supplementary material for: Experimental and computational approaches for deep metabolome annotation with application to the ecotoxicological model organism Daphnia magna
Source: Gigascience. 2026 May 9;15:giag055. doi: 10.1093/gigascience/giag055 (PMC13254479; doi:10.1093/gigascience/giag055)
Supplement: giag055_Reviewer_2_Report_original_submission — Reviewer 2 -- 11/30/2025 [file giag055_reviewer_2_report_original_submission.pdf]

Reviewer Report

Title: Experimental and computational approaches for deep metabolome annotation with application to the ecotoxicological model organism Daphnia magna

Version: Original Submission **Date:** 11/30/2025

Reviewer name: Reviewer 2

Reviewer Comments to Author:

Comments on "Experimental and computational approaches for deep metabolome annotation with application to the ecotoxicological model organism Daphnia magna"This manuscript reports on a large-scale metabolomics approach to characterize the metabolome of the model organism D. magna. This study is very timely, as more and more omics approaches are applied to Daphnia and in combination, they are a powerful tool to understand the biology of this ecologically important organism. Being a powerful experimental system, mainly used in ecological context, D. magna is very widely used. The current study adds to the resources available for this system. The study appears well done and the different datasets created are all made available. Thus, future generations of software can even drive this further.I am in favour for the publication of this study.While this comment is too late now (the study is already done), it would have been good to use a proper method for gut evacuation of the animals. Starving is known not to do this. Daphnia digest slowly. For gut evacuation, feeding the Daphnia with dextran beats, replaces the entire gut content with these beats. This would reduce the problem with metabolites potentially derived from the food (Chlorella algae) and microbiota (fungi and bacteria).Where the Daphnia checked for parasites before the 10 genotypes were used? Many D. magna clone harbour parasites in their gut epithelium.I appreciate that the authors tried to diversify across D. magna genotypes, but the sample of 10 D. magna clones used seems a bit arbitrary. Most strain collections around the world, use central European D. magna clones, e.g. the NIES clone from Japan is from Europe (via the US). But I think this is ok, as most work on this model species is done in Europe anyway. Future studies may include confirmed isolates from the East Asian, South African and North American D. magna lineage.The references cited need some curation:Book chapter are often incorrectly formatted and incomplete.Several references miss the year of publication (e.g. ref. 17, 18, 20, 32, 38, 46), other miss the doi (e.g ref. 6, most book chapters).Incorrect formatting in ref. 16.Section 2.1.3, third line Type: "polar polar"

Level of Interest

Please indicate how interesting you found the manuscript: Choose an item.

Quality of Written English

Please indicate the quality of language in the manuscript: Choose an item.

Declaration of Competing Interests

Please complete a declaration of competing interests, considering the following questions:

- Have you in the past five years received reimbursements, fees, funding, or salary from an organisation that may in any way gain or lose financially from the publication of this manuscript, either now or in the future?
- Do you hold any stocks or shares in an organisation that may in any way gain or lose financially from the publication of this manuscript, either now or in the future?
- Do you hold or are you currently applying for any patents relating to the content of the manuscript?
- Have you received reimbursements, fees, funding, or salary from an organization that holds or has applied for patents relating to the content of the manuscript?
- Do you have any other financial competing interests?
- Do you have any non-financial competing interests in relation to this paper?

If you can answer no to all of the above, write 'I declare that I have no competing interests' below. If your reply is yes to any, please give details below.

I declare no competing interests.

I agree to the open peer review policy of the journal. I understand that my name will be included on my report to the authors and, if the manuscript is accepted for publication, my named report including any attachments I upload will be posted on the website along with the authors' responses. I agree for my report to be made available under an Open Access Creative Commons CC-BY license (http://creativecommons.org/licenses/by/4.0/). I understand that any comments which I do not wish to be included in my named report can be included as confidential comments to the editors, which will not be published.

Choose an item.

To further support our reviewers, we have joined with Publons, where you can gain additional credit to further highlight your hard work (see: https://publons.com/journal/530/gigascience). On publication of this paper, your review will be automatically added to Publons, you can then choose whether or not to claim your Publons credit. I understand this statement.

Yes Choose an item.
